# Photochemical Deracemization of Aza-1-isoindolinones: Critical Influence of Catalyst Substitution and the Nature of Its Resting State

**DOI:** 10.1021/jacs.6c05440

**Published:** 2026-05-30

**Authors:** Philip Freund, Mike Pauls, Miriam Jänchen, Julian Zuber, Jürgen Hauer, Christoph Bannwarth, Thorsten Bach

**Affiliations:** † Department Chemie and Catalysis Research Center (CRC), School of Natural Sciences, Technische Universität München, Garching D-85747, Germany; ‡ Institut für Physikalische Chemie, 9165 RWTH Aachen University, Aachen D-52074, Germany

## Abstract

The photochemical deracemization of aza-1-isoindolinones has been investigated. A chiral *para*-methoxy-substituted phenyl ketone was discovered that promotes the conversion of racemic 4-aza-1-isoindolinones into their enantiopure form upon irradiation. Products were obtained in yields of 76–96% and with an enantiomeric excess of 86–99% (23 examples). Quantum chemical calculations suggest that the catalyst acts by removing a hydrogen atom at the stereogenic center of the substrate and returning it via its N4 nitrogen atom. It was found that the intermediately formed radicals combine to form a 1:1 adduct that acts as a resting state of the catalyst. Crossover experiments and transient absorption spectroscopy provided evidence that the radical–radical recombination is reversed upon irradiation, restoring the active catalyst. Due to a notable absorption at λ = 350 nm, *para*-methoxy substitution seems to facilitate the cleavage, which accounts for the better performance of the substituted vs the unsubstituted phenyl ketone catalyst. When applying the catalyst to the photochemical deracemization of other aza-1-isoindolinones, it was observed that the success of the reaction depends on the stability of the intermediate formed by back hydrogen atom transfer (bHAT) from the catalyst to the substrate. Racemic 6-aza-3-benzyl-1-isoindolinone and 7-aza-3-benzyl-1-isoindolinone could be successfully employed in a photochemical deracemization, because they offer either a nitrogen atom at N6 (6-aza) or the isoindolinone oxygen atom (7-aza) to form enamine and enol intermediates upon bHAT. 5-Aza-3-benzyl-1-isoindolinone leads, in a nonpolar solvent, only to high-energy intermediates and delivers a low enantiomeric excess under standard deracemization conditions.

## Graphical abstract

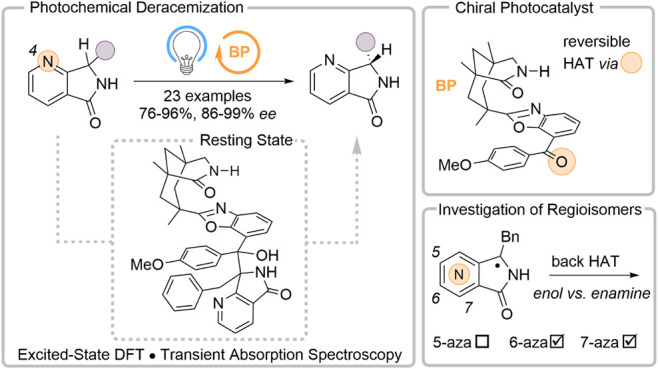

## Introduction

The term deracemization describes a transformation in which a racemate of a given chiral compound “is made nonracemic by increasing the quantity of one enantiomer at the expense of the other”.^1^ Ideally, a deracemization converts a racemate to a single enantiomer in quantitative yield.^2^ In homogeneous solution under thermal conditions, the process is impossible, because the racemate is entropically favored, and a catalyst cannot alter a given thermodynamic preference.^3^ Photochemically,^4^ the boundaries of microscopic reversibility^5^ can be overcome by converting a given enantiomer to a short-lived intermediate in a photochemical step and by reforming the same compound in a thermal reaction. Ever since the first successful photochemical deracemization was described in 2018,^6^ the field has been rapidly growing, and several compound classes have been found to be accessible in enantiomerically pure or enriched form from their respective racemates.^4^ The most straightforward approach toward a photochemical deracemization requires a single chiral catalyst and no other additives or reagents.^7^ Toward this goal, our group has pursued an approach in which a chiral photocatalyst^8^ acts on a given substrate with a stereogenic carbon atom by a reversible hydrogen atom transfer (HAT) mechanism (Scheme 1).^9,10^

**Scheme 1:**
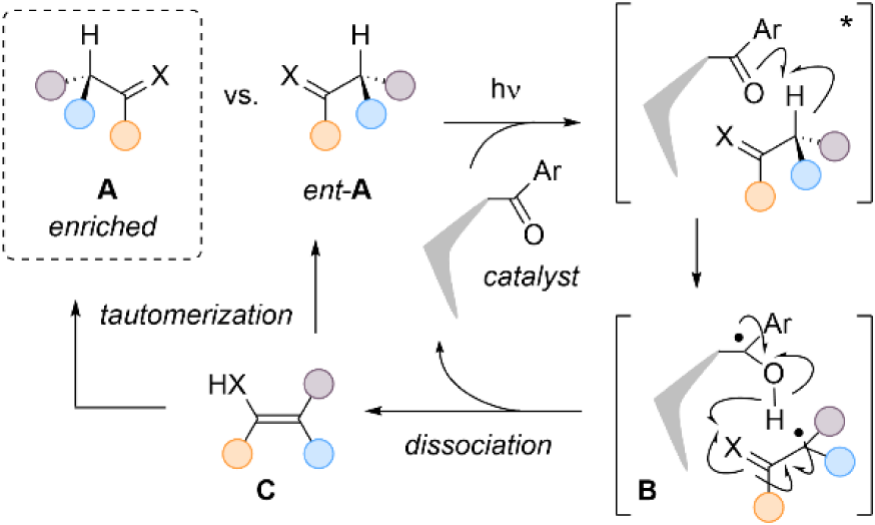
General Mechanistic Scheme of a Photochemical Deracemization by Reversible Hydrogen Atom Transfer (*X* = O, NR)

In this scenario, the catalyst features a photochemically active aromatic ketone fragment (Ar = aryl) and is competent to selectively address one of the two enantiomers **A** and *ent*-**A**. Excitation of the carbonyl chromophore populates by rapid intersystem crossing (ISC) its lowest triplet state (T_1_), which has nπ* character and invites a HAT to the carbonyl oxygen atom.^11^ If enantiomer recognition is enabled by proper binding to one of the two enantiomers, here *ent*-**A**, and if the hydrogen atom at the stereogenic center is appropriately positioned, the HAT process occurs with high selectivity. The ensuing radical pair **B** comprises a protonated ketyl radical and an achiral radical derived from the substrate. It has been found that back HAT (bHAT) to the substrate is efficient to a heteroatom *X* (oxygen or nitrogen atom) that is in conjugation with the radical center.^9^ The process avoids regeneration of the same substrate enantiomer by reforming the broken C–H bond within the chiral catalyst-substrate assembly. Rather, the achiral enol or enamine product **C** dissociates from the catalyst before statistically undergoing tautomerization to either enantiomer **A** or *ent*-**A**. Since enantiomer **A** is not processed by the catalyst, it is gradually enriched and remains as the only product once the enantiomer *ent*-**A** is consumed.

A critical parameter to achieve the desired reaction pertains to the proper positioning of the carbonyl chromophore and the targeted hydrogen atom in the substrate. For the deracemization of 4,7-diaza-1-isoindolinones *rac*-**1** ^12^ it was found that a chiral benzoxazole with a lactam hydrogen bonding motif and a benzoyl group in 5-position was unsuited to achieve the desired hydrogen abstraction at carbon atom C3 of substrate enantiomer *ent*-**1**. It was required to position the benzoyl group at carbon atom C7 of the benzoxazole core and the resulting 7-substituted catalyst (+)-**2a** showed an excellent performance in the desired deracemization reaction (Scheme 2). Products **1** were obtained in high yields and with excellent enantiomeric excess (*ee*). In the complex of this catalyst with the processed enantiomer *ent*-**1**, the oxygen atom of the carbonyl group was found by quantum chemical calculations to be as close as 251 pm to the hydrogen atom at C3 while the less efficient catalyst displayed a distance of 316 pm in the analogous complex.

**Scheme 2:**
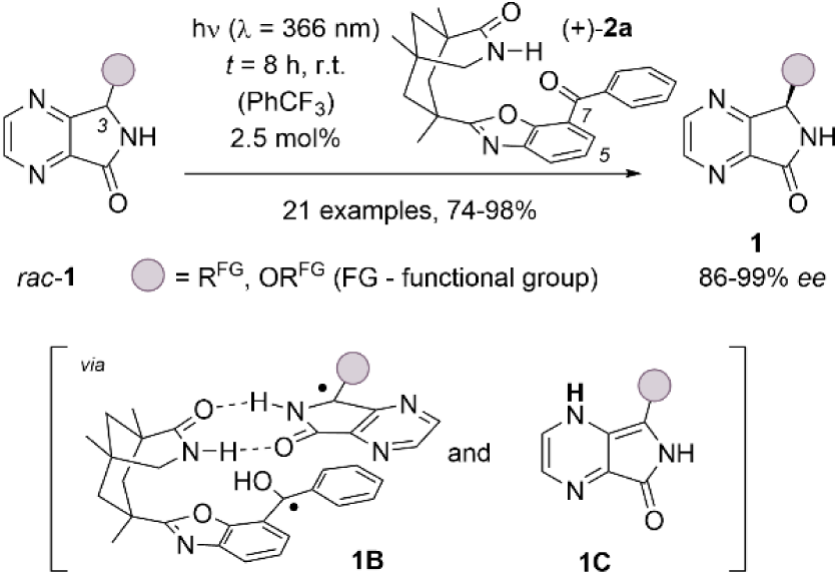
Previous Work on the Photochemical Deracemization of 4,7-Diaza-1-isoindolinones *rac*-**1** Promoted by Photocatalyst (+)-**2a**

Key intermediates in the process are the achiral radical, which is derived from *ent*-**1** by HAT and which forms a radical pair **1B** with the protonated ketyl radical (cf. also Scheme 1), and the achiral enamine intermediate **1C**, which is generated by bHAT from the protonated ketyl radical of the catalyst. In addition to the initial HAT, the bHAT to the nitrogen atom could also be nicely visualized by quantum chemical calculations, which revealed a low energy trajectory for the process.^12^

In the present study, we attempted to apply the deracemization protocol established for 4,7-diaza-1-isoindolinones to other aza-1-isoindolinones. The latter compounds are relevant for drug development,^13^ but there is no general route to access them in enantiomerically pure form. Surprisingly, it was found that catalyst **2a** was inefficient in catalyzing the desired photochemical deracemization of 4-aza-1-isoindolinones. Instead, it was required to modulate the electronic properties of the catalyst by appropriate substitution within the phenyl ring of the benzoyl group. A catalyst was identified which delivered the desired transformation with high enantioselectivity and in high yields, enabling an access to various substituted pyridine derivatives as consecutive products. By analyzing the time course of the reaction, we found that the catalyst-substrate complex was completely converted to a new compound after only 1 h of irradiation. Since the photochemical deracemization continued upon continued irradiation, the new compound appears to serve as a photochemically activatable resting state of the catalyst. In this report, we provide full details on our synthetic, mechanistic, and quantum chemical studies on 4-aza-1-isoindolinones and we disclose our results on a possible application of the process to other aza-1-isoindolinones.

## Results and Discussion

### Catalyst Discovery, Product Scope, and Consecutive Reactions

Preliminary studies on the deracemization of 4-aza-1-isoindolinones commenced with the 3-benzyl-substituted compound *rac*-**3a**. For supply reasons, all reactions performed in the present study were performed with the (−)-enantiomer of the respective catalyst, initially with (−)-**2a**. Under the conditions optimized for the deracemization of 4,7-diaza-1-isoindolinones, the desired enantiomer **3a** with the expected (*S*)-configuration at carbon atom C3 was obtained in a low *ee* (Scheme 3). Several other conditions were applied but the outcome was equally disappointing. At a shorter irradiation wavelength (λ = 350 nm), the selectivity increased, but the *ee* remained below 50%.

**Scheme 3:**
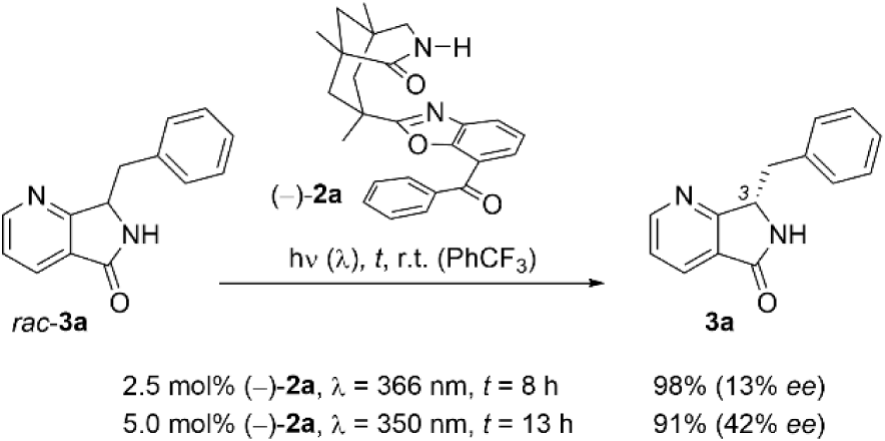
Attempted Photochemical Deracemization of 4-Aza-1-isoindolinone *rac*-**3a** Employing Catalyst (−)-**2a**

Although it was not immediately apparent which parameter was responsible for the decrease in enantioselectivity, we felt, supported by quantum chemical calculations, that the trajectory for the forward and return HAT remained unchanged when going from compounds *rac*-**1** to the 4-aza derivatives *rac*-**3**. In other words, it made no sense to change the position of the benzoyl group nor the structure of the recognition motif. Rather, we attempted to modulate the photophysical parameters of the benzoyl group by attaching substituents to its *para* position. A successful synthesis campaign furnished ketones with a *para*-methoxy (**2b**) and a *para*-trifluoromethyl substituent (**2c**) at the phenyl ring. After separation of the two enantiomers by preparative chiral HPLC, the enantiomerically pure compounds were obtained, and it was confirmed that the (−)-enantiomers display the same absolute configuration as catalyst (−)-**2a** (see the Supporting Information for details). Comparison of the UV–vis spectra revealed that catalyst (−)-**2b** features a more intense long-wavelength absorption than (−)-**2a** and (−)-**2c** (Figure 1). Absorption coefficients at the local maxima were determined as ε = 105 M^–1^ cm^–1^ (λ = 360 nm) for (−)-**2a**, ε = 221 M^–1^ cm^–1^ (λ = 353 nm) for (−)-**2b**, and ε = 92 M^–1^ cm^–1^ (λ = 367 nm) for (−)-**2c**. The observation aligns with the more pronounced ππ*-character of *para*-methoxy-substituted phenyl ketones.^14^ For example, 4,4′-dimethoxybenzophenone displays an absorption maximum at λ = 331 nm with an absorption coefficient ε = 890 M^–1^ cm^–1^ while the long wavelength absorption of benzophenone is weaker with ε = 150 M^–1^ cm^–1^ (λ = 338 nm).^15^

**Figure 1:**
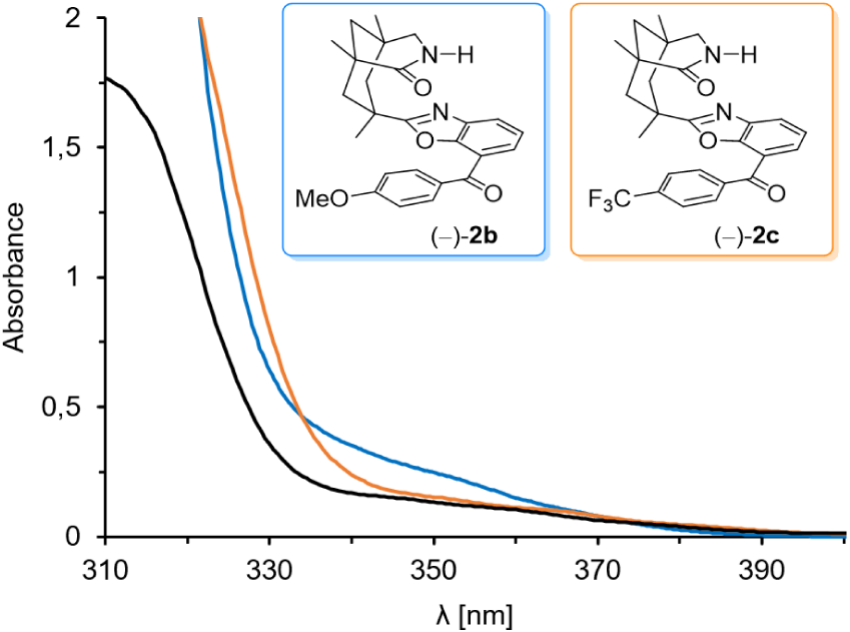
UV–vis spectra of aromatic ketones (−)-**2a** (black), (−)-**2b** (blue), and (−)-**2c** (orange) in the long wavelength region λ > 310 nm (*c* = 1.0 mM in CH_2_Cl_2_).

The enhanced ππ*-character of 4,4′-dimethoxybenzophenone can also been accounted responsible for the lower reactivity of the compound in hydrogen abstraction reactions.^16^ The bimolecular rate constants for hydrogen abstraction from isopropanol in acetonitrile had been found to be 2.83 (±0.15) × 10^5^ L mol^–1^ s^–1^ for 4,4′-dimethoxybenzophenone, 2.3 (±0.3) × 10^6^ L mol^–1^ s^–1^ for benzophenone, and 1.45 (±0.05) × 10^7^ L mol^–1^ s^–1^ for 4,4′-bis­(trifluoromethyl)­benzophenone.^16b^ Against this background, it came as a major surprise that catalyst (−)-**2b** outperformed dramatically the other two benzophenones in the deracemization reaction of substrate *rac*-**3a**. Under the conditions given in Scheme 3 for the reaction catalyzed by (−)-**2a** at λ = 350 nm, catalyst (−)-**2b** provided the desired enantiomer **3a** in 84% yield and with 98% *ee*. Catalyst (−)-**2c** remained in the same regime as the unsubstituted phenyl ketone, providing the product in 91% yield and with 36% *ee*. A rate profile recorded for the deracemization of compound *rac*-**3a** (Figure 2) illustrates impressively the outstanding performance of catalyst (−)-**2b** vs (−)-**2a**.

**Figure 2:**
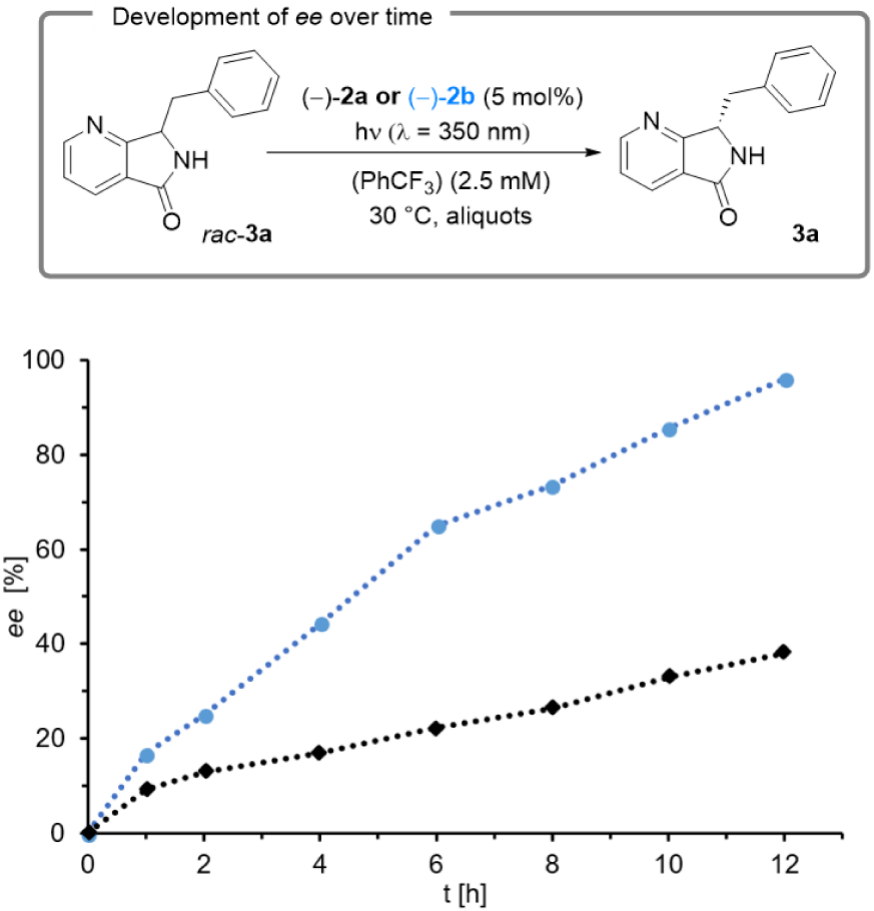
Rate profile for the photochemical deracemization of 4-aza-1-isoindolinone *rac*-**3a** depending on the photocatalyst: (−)-**2a** (black) vs (−)-**2b** (blue).

The reaction was monitored by taking aliquots from the mixture and analyzing them by chiral HPLC. While the unsubstituted catalyst (−)-**2a** displays low activity and does not lead to a satisfactory *ee* increase within an irradiation time of 12 h, the *para*-methoxy-substituted ketone (−)-**2b** continues to turn over the substrate until a high enantiomeric excess is reached in the same time period. For all preparative experiments the reaction was terminated after 13 h, and the products were isolated by column chromatography. The substrate scope of the transformation was found to be wide and structurally diverse (Scheme 4). It not only includes 3-benzyl-substituted 4-aza-1-isoindolones *rac*-**3a** to *rac*-**3e** but also 3-alkyl-substituted derivatives *rac*-**3f** to *rac*-**3k**. The size of the substituent is not relevant for the success of the deracemization, and even 3-ethyl-4-aza-1-isoindolinone (**3i**) was isolated in 98% *ee*. Substituents within the benzo core of the 4-aza-1-isoindolinone were tolerated well (products **3k** to **3q**).

**Scheme 4:**
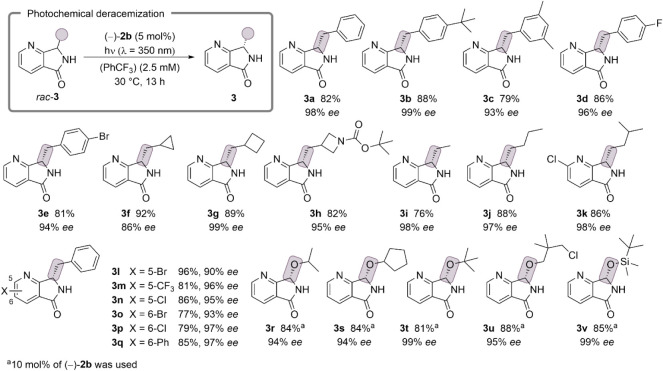
Substrate Scope for the Photochemical Deracemization of 4-Aza-1-isoindolinones *rac*-**3** Catalyzed by Benzophenone (−)-**2b**

In general, the reaction is compatible with the halogen substituents fluorine (**3d**), chlorine (**3k**, **3n**, **3p**, **3u**), and bromine (**3e**, **3l**, **3o**). Additional functional groups, which were stable under the reaction conditions, include a *tert*-butyloxycarbonyl-protected amine (**3h**), a phenyl group (**3q**) and a silyl group (**3v**). The 3-oxysubstituted 4-aza-1-isoindolinones *rac*-**3r** to *rac*-**3v** required a higher catalyst loading (10 mol %) to reach consistently enantioselectivities >90% *ee* within a reasonable time span (13 h). Typically, the reactions were performed on a scale of 25 μmol. On larger scale, product **3a** was obtained in a 1 mmol experiment in 84% yield and with 95% *ee* at an expanded reaction time of *t* = 30 h. Product **3l** was obtained in a 0.2 mmol reaction in 85% yield and with 91% *ee* (*t* = 18 h). The absolute configuration of the products was assigned based on single-crystal X-ray analysis performed with 4-aza-1-isoindolinone **3e** (>99% *ee*). Anomalous dispersion effects in the diffraction experiment revealed the compound to be (*S*)-configured, which matched our expectations and which aligned with previous results on 4,7-diaza-1-isoindolinones.^12^ The deracemization protocol can be used to edit a stereogenic center at C3 even if other stereogenic centers are present (Scheme 5).

**Scheme 5:**
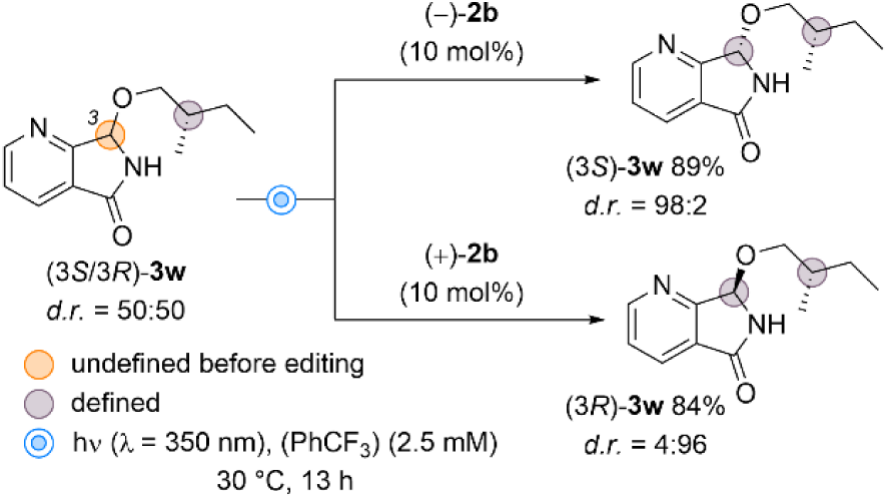
Stereochemical Editing at Position C3 of 4-Azaisoindolinone **3w**

Substrate **3w** was obtained from (*S*)-2-methylbutanol and 3-hydroxy-4-aza-1-isoindolinone as a mixture of two diastereoisomers at position C3. The stereogenic center could be edited with high diastereoselectivity (d.r. = diastereomeric ratio) toward product (3*S*)-**3w** if catalyst enantiomer (−)-**2b** was employed. With the opposite catalyst enantiomer (+)-**2b**, diastereoisomer (3*R*)-**3w** was formed.

Since the ring opening of 4-aza-1-isoindolinones is facile, the photochemical deracemization reaction is a useful tool to prepare pyridines with a stereogenic carbon center attached to the C2 carbon atom of pyridines. To demonstrate the feasibility of the described approach, product **3a** (95% *ee*) obtained from the mmol scale reaction (vide supra) was protected with a *tert*-butoxycarbonyl (Boc) group (DMAP = *N*,*N*-dimethylaminopyridine) to yield product **4** (Scheme 6). Lactam ring opening was achieved by treatment with lithium hydroxide delivering the desired 2,3-disubstituted pyridine **5**. Since the enantiomeric purity of the compound could not be accessed by chiral HPLC, the carboxylic acid was reduced to alcohol **6**,^17^ which was isolated in high yield and with almost perfect retention of the chiral information (93% *ee*).

**Scheme 6:**
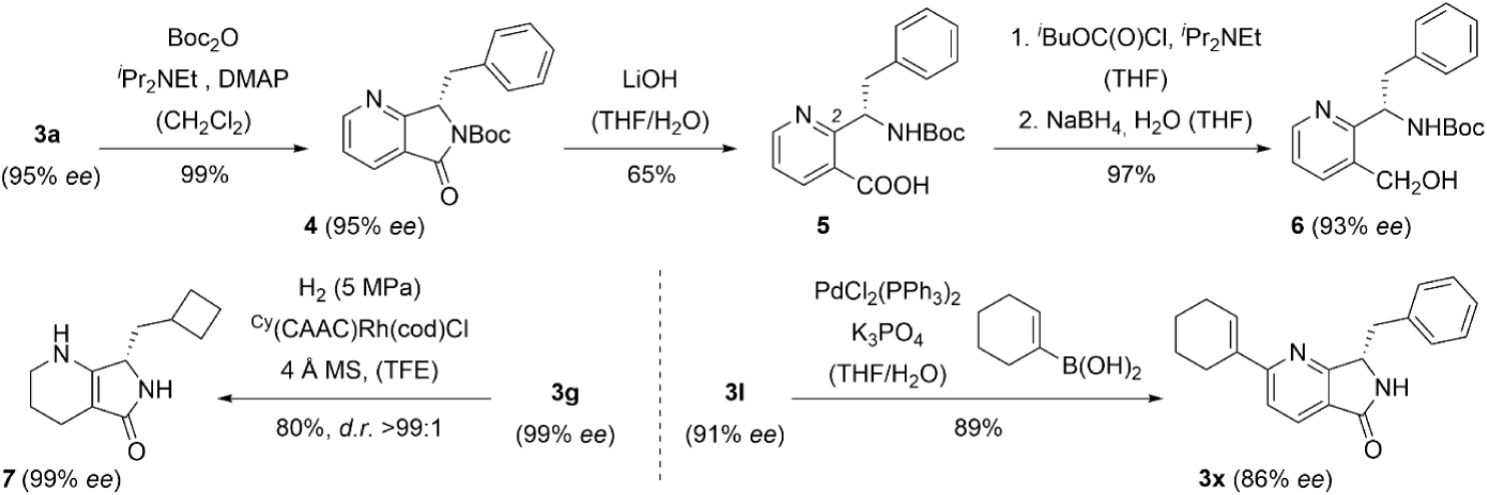
Follow-Up Reactions Performed with Enantioenriched 4-Azaisoindolinones **3a**, **3g**, and **3l**

The stereochemical integrity of the products was further probed in a hydrogenation reaction of the pyridine core within 4-aza-1-isoindolinone **3g** (99% *ee*). The reaction was performed under hydrogen pressure (MPa = megapascal) and was mediated by 2.5 mol % of a rhodium catalyst [^Cy^(CAAC) = cyclic (diisopropylphenyl)­(amino)­carbene; (CAAC)­cod = 1,5-cyclooctadiene].^18^ The hydrogenation product **7** was isolated and showed complete retention of configuration (99% *ee*). A Suzuki cross-coupling^19^ performed with 5-bromo-4-aza-1-isoindolinone **3l** (91% *ee*) gave the desired coupling product **3x** (86% *ee*).

### Quantum Chemical Calculations and Mechanistic Studies

Although the use of catalyst (−)-**2b** had led to a synthetically useful protocol for the deracemization of 4-aza-1-isoindolinones, its mode of action and the reason for its superior performance compared to ketone (−)-**2a** remained unclear. Phosphorescence studies performed at 77 K (PhCF_3_) revealed for (−)-**2b** the typical signature of an aromatic ketone. The spectrum does not significantly differ from the spectrum of (−)-**2a**, and it also displays the regular approximately 1600 cm^–1^ spacing between vibronic bands, which is one of the hallmarks of an nπ* triplet state.^20^ From the shortest wavelength emission at λ = 428 nm, the triplet energy was determined as *E*_T_ = 280 kJ mol^–1^. The value is almost identical to the triplet energy determined previously for (−)-**2a** (*E*_T_ = 276 kJ mol^–1^, 77 K, PhCF_3_).^12^ In line with this analogy, quantum chemical calculations revealed the expected behavior for catalyst (−)-**2b** in the photochemical deracemization of substrate *rac*-**3a** (Figure 3).

**Figure 3:**
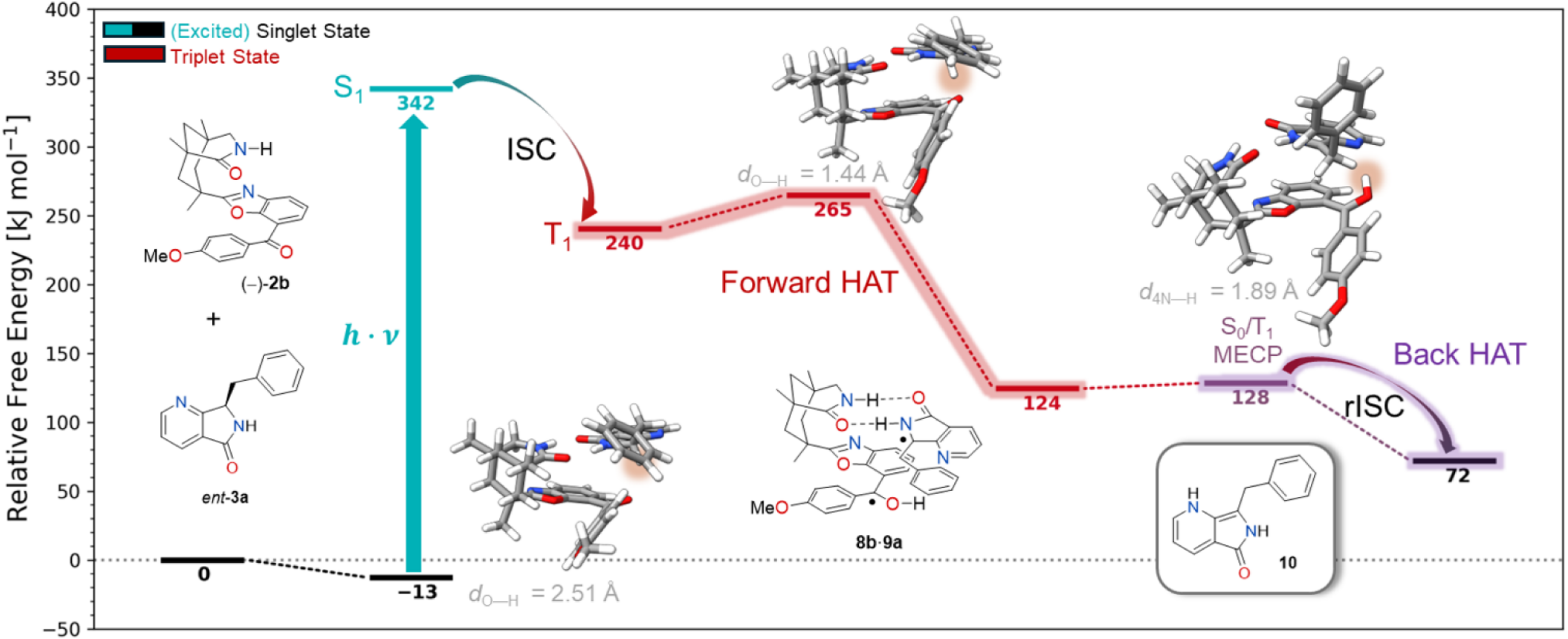
Computed free energy reaction profile for the photocatalytic deracemization of *rac*-**3a**. Electronic energies are calculated at the PW6B95-D4/def2-QZVP//PBEh-3c+CPCM^21^ level of theory (see the Supporting Information for details on the Gibbs free energy contributions).^22^ Free energies are given relative to the free monomers *ent*-**3a** and (−)-**2b**. The forward (red) and back HAT (purple) steps are highlighted, and the relevant distances (*d*_O–H_ and *d*_4N–H_, respectively) are given. The free energy at the S_0_/T_1_ minimum energy crossing point is approximated by an additive scheme (see the Supporting Information for details) using the electronic energy at the crossing point, and a proximate T_1_ minimum. Likewise, the S_1_ excited-state energy is computed vertically at the p-DFT/MRCI^23^ level of theory.

Formation of the noncovalent substrate-catalyst complex between the enantiomer *ent*-**3a** and catalyst (−)-**2b** is favorable with the exergonic association free energy determined as Δ*G* = −13 kJ mol^–1^. Photoexcitation of the catalyst into the excited state S_1_, followed by ISC to the T_1_ triplet state enables the forward hydrogen atom transfer from *ent*-**3a** to take place overcoming a barrier of only 25 kJ mol^–1^. At the T_1_ transition state geometry, the distance between the benzophenone oxygen atom and the hydrogen atom at the stereogenic center is reduced to 144 pm. After forward HAT, the biradical intermediate **8b**·**9a** is formed, which is associated with a decrease in free energy of Δ*G* = (124 – 240) kJ mol^–1^ = −116 kJ mol^–1^. The successive bHAT is associated with a small energetic barrier $\left(\Delta G_{\mathrm{bHAT}}^{‡}=4\mathrm{kJ}{\mathrm{mol}}^{-1}\right)$ to reach a nearby S_0_/T_1_ minimum energy crossing point (MECP). After returning to the S_0_ state, enamine intermediate **10** is formed which undergoes tautomerization^24^ to either one of the substrate enantiomers. The relevant distance for this second HAT step between the nitrogen atom in 4-position and the OH unit of **8b** is small (189 pm), thus, providing the prerequisites for a suitable coordinate.

From the quantum chemical calculations there was no immediate evidence explaining the improved performance of catalyst (−)-**2b** vs catalyst (−)-**2a**. The somewhat linear rate profile (Figure 2) led us to speculate that the stability of the respective catalyst and its concentration might be responsible for the different efficiency. Employing (−)-**2b** as the photocatalyst, we, thus, monitored the reaction of *rac*-**3a** → **3a** over time by HPLC analysis. At the beginning of the reaction (*t* = 0 h), the peaks of the two enantiomers **3a** and *ent*-**3a** were equal in intensity, and catalyst (−)-**2b** was clearly visible with a retention time *t*_R_ = 7.49 min. Much to our surprise, the catalyst completely disappeared after 1 h (*t* = 1 h) of irradiation (Figure 4). The observation was highly unexpected, because it is evident from the time profile (Figure 2) that the deracemization proceeds up to a reaction time of *t* = 12 h.

**Figure 4:**
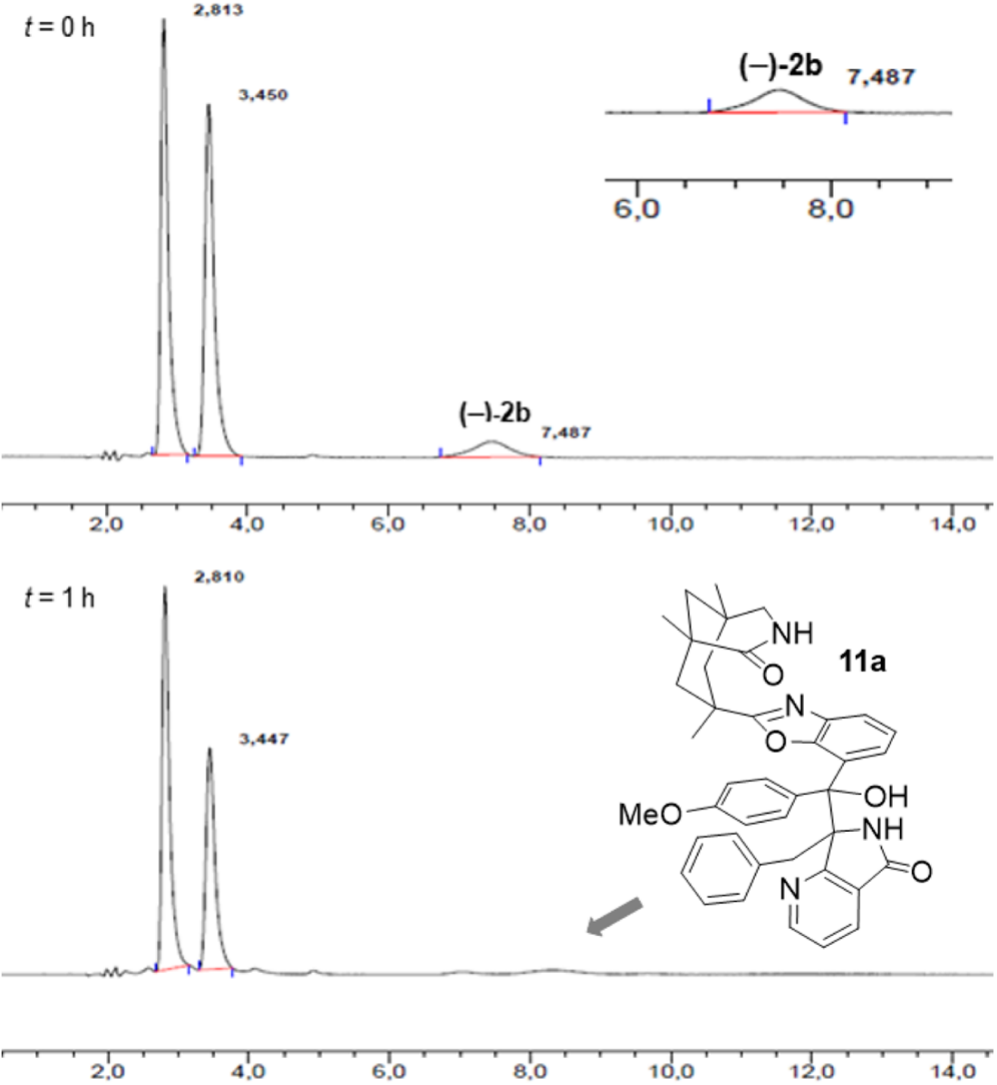
HPLC traces of the reaction mixture for the deracemization *rac*-**3a** → **3a** at the beginning of the reaction (*t* = 0 h) and after 1 h (*t* = 1 h). Structure of catalyst resting state **11a** formed by combination of radicals **8b** and **9a**.

In a preparative run, the reaction was stopped after 1 h, and analysis of the reaction mixture indicated the presence of material with a retention time *t*_R_ > 8.00 min. Careful separation led to the isolation of a new compound with an exact electrospray ionization (ESI) mass *m*/*z* = 657.3071 [M + H^+^]. Based on NMR analysis, the compound was a mixture of diastereoisomers with both signals of the catalyst skeleton and the 4-aza-1-isoindolinone present. IR analysis revealed the presence of the indolinone lactam band at approximately 1690 cm^–1^ and the absence of an aromatic carbonyl band at approximately 1645 cm^–1^ confirming the structure assignment for compound **11a** (calcd. *m*/*z* = 657.3059). In fact, when revisiting the quantum chemical calculations (see the Supporting Information), the radical recombination of protonated ketyl radical **8b** and aza-1-isoindolinone radical **9a** was clearly indicated in an unbiased search as a viable pathway.^25^ We had discarded the idea of a radical–radical recombination, because we had believed it to be unproductive. As it turned out, compound **11a** seems to serve as a resting state for the catalyst from which the active form is regenerated photolytically. Upon irradiation at λ = 350 nm the C–C bond at the tertiary alcohol appears to be cleaved to form radicals **8b** and **9a**, and the catalyst (−)-**2b** is regenerated by bHAT to the substrate (Scheme 7A). The calculated barrier $\left(\Delta G_{\mathrm{calc}}^{‡}\right)$ for the cleavage is low, and related chromophores^26^ are known to absorb in the wavelength region λ > 300 nm (vide infra).

**Scheme 7:**
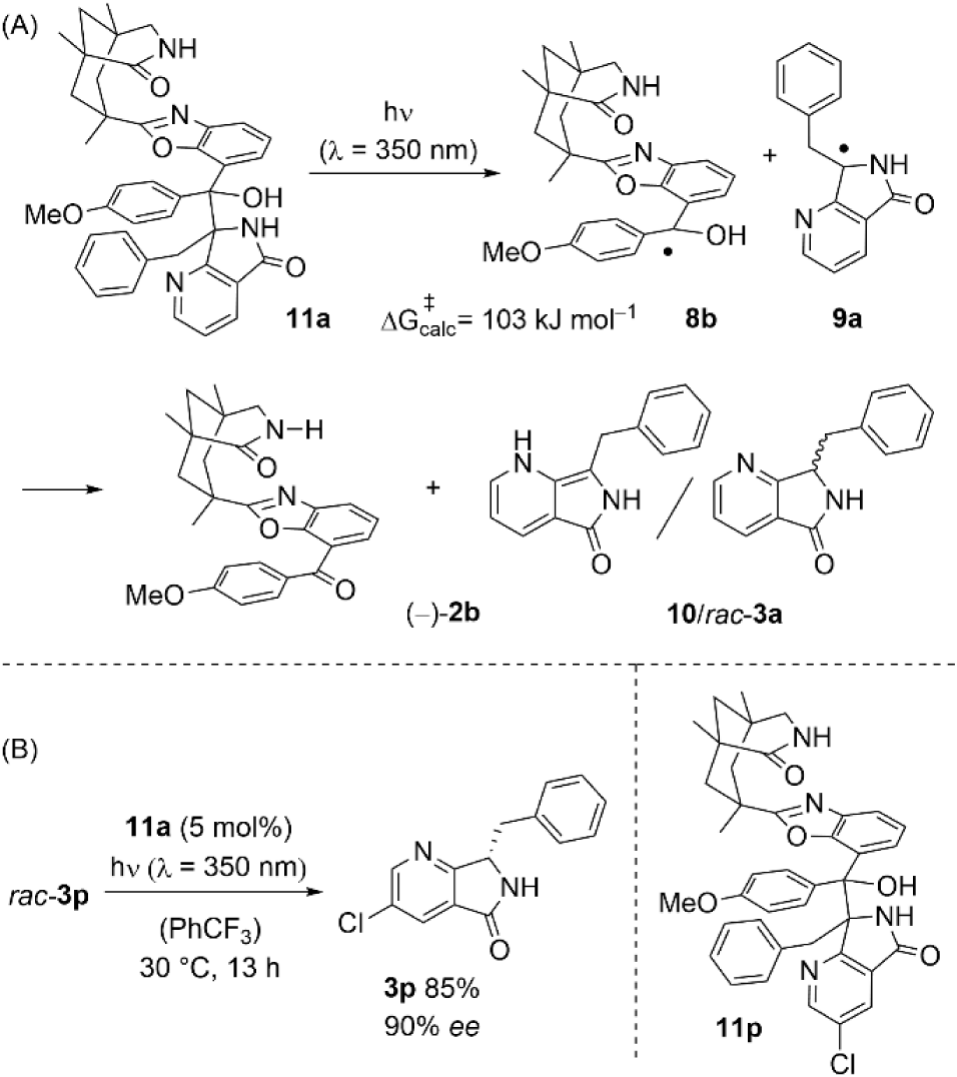
A. Cleavage of Compound **11a** upon Irradiation at λ = 350 nm and Formation of Active Catalyst (−)-**2b.** B. Photochemical Deracemization Mediated by Catalyst Resting State **11a** and Structure of Crossover Product **11p**

The hypothesis of a reversible C–C bond formation was substantiated experimentally by performing the photochemical deracemization of chlorinated 4-aza-1-isoindolinone *rac*-**3p** with catalytic quantities of compound **11a**. Under otherwise unchanged conditions, the reaction delivered the desired product **3p** with 90% *ee*. In addition, a new compound was isolated from the reaction mixture that displayed in full accordance with structure **11p** an exact ESI mass *m*/*z* = 691.2670 [M­(^35^Cl) + H^+^, calcd. 691.2682]. Its formation demonstrates that the C–C bond within **11a** was cleaved during the reaction and a new C–C bond was formed, most likely via radical intermediate **8b** (Scheme 7B).

As quantum chemical calculations suggested an immediate cleavage of compound **11a** into its components **8b** and **9a** on the singlet hypersurface (see Figure S20 in the Supporting Information), we subjected the compound to transient absorption spectroscopy at the fs/ps time scale (Figure 5). The central wavelength of the pump pulse was set to λ = 323 nm in dichloromethane solution, which is resonant with the red edge of the main absorption of **11a** (see the Supporting Information). Pulses were compressed to 30 fs, as estimated from the duration of the coherent artifact in a solvent-only transient absorption measurement.^27^

**Figure 5:**
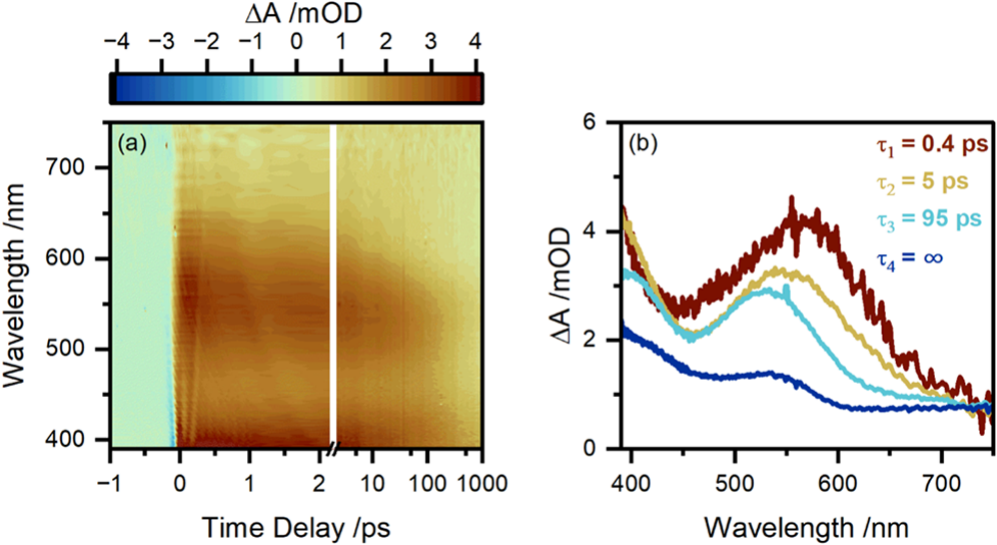
Transient absorption data for **11a** in dichloromethane solution after 323 nm/30 fs excitation. (a) Transient data after excitation at 323 nm, resolved in detection wavelength and pump–probe delay. (b) Evolution-associated decay spectra and related time constants extracted by a global kinetic fit for the data set shown in (a).

Figure 5a shows the measured transient absorption data in differential absorption (Δ*A*), resolved in probe wavelength and pump–probe delay. Immediately upon excitation, there are two excited-state absorption bands observable (positive signals), one below 450 nm and one between 450 and 650 nm, which do not decay completely within the measured time window. Based on literature precedence,^9a,16b,28^ we assign the excited state absorption (ESA) feature between 450 and 650 nm to the protonated ketyl radical **8b** (Scheme 7A), which is formed immediately upon excitation of **11a**. For further insights, we performed a global kinetic fitting analysis, as described in the Supporting Information. Figure 5b shows the resulting evolution-associated spectra (EAS) and the respective time constants. The EAS show a blueshift and narrowing in the ESA band between 450 and 600 nm on picosecond time scales (components with τ_1_ to τ_3_) which we suggest to be due to vibrational cooling processes in the excited radical.^29^ As for the assignment of the last EAS component (τ_4_), its spectral similarities to the other components suggest that it also stems from the radical, with typical lifetimes beyond the 1 ns measurement window. The changed relative intensities between the third and fourth components are in agreement with the progress of the bHAT from **8b** to radical **9a** which regenerates catalyst (−)-**2b** (cf. Scheme 7) and appears to remain incomplete within the time frame of the transient absorption experiment. Although the spectral features of the longer-lived EAS components would also match the spectra of a triplet benzophenone,^28a^ we rule out that bHAT from **8b** to radical **9a** leads to the triplet state of (−)-**2b** given that the process is more than 100 kJ mol^–1^ uphill (cf. Figure 3). The absorption spectrum of (−)-**2b**, shown in Figure 1, is too far blue-shifted to be detected in the probing window of our transient absorption experiment.

The cleavage of a putative catalyst resting state provides a straightforward explanation for the improved performance of catalyst (−)-**2b** vs (−)-**2a**. The 1:1 adduct of the latter catalyst shows a significantly weaker absorption at λ = 350 nm as compared to intermediate **11a** (see the Supporting Information for details). As a result, the recovery of the catalyst is slower, and the reaction progresses with a significantly decreased reaction rate (cf. Figure 2). The explanation aligns with the wavelength-dependent performance of catalyst (−)-**2a** (cf. Scheme 3). Although the adduct of catalyst (−)-**2c** has not been isolated in this study, it is reasonable to assume that its UV–vis properties are similar to those of the (−)-**2a** adduct. In previous work on 4,7-aza-1-isoindolinones (cf. Scheme 2),^12^ it was not attempted to isolate any 1:1 adduct of catalyst (−)-**2a** and the substrate. Given the findings of the current study, it appears likely that a related adduct forms but is readily cleaved at λ = 366 nm. In fact, the UV–vis spectra of 4,7-aza-1-isoindolinones are markedly red-shifted compared to 4-aza-1-isoindolinones.

### Photochemical Deracemization of Regioisomeric Azaisoindolinones

When expanding the photochemical deracemization to other aza-1-isoindolinones, we were faced with the question of a viable pathway for bHAT in any of the intermediates. Quantum chemical calculations indicated that a rotation around the C–C bond that links the benzoyl-substituted benzoxazole to the chiral 1,5,7-trimethyl-3-azabicyclo[3.3.1]­nonan-2-one scaffold is feasible ( $\Delta G_{T1}^{‡}=50\mathrm{kJ}{\mathrm{mol}}^{-1}$ for free (−)-**2b**, $\Delta G_{S0}^{‡}=69\mathrm{kJ}{\mathrm{mol}}^{-1}$ in the adduct, see the Supporting Information for details). The rotation enables the intermediate **8b** to deliver the hydrogen atom to a nitrogen atom in the ring but also to the oxygen atom of the isoindolinone, which is involved in the two-point hydrogen bonding to the catalyst. In other words, the forward HAT to photoexcited catalyst (−)-**2b** was expected to occur in the same fashion as for 4-aza-1-isoindolinones *ent*-**3**, but the respective intermediates **13** formed by this process can decay by a bHAT to enamine intermediates **14** or to enol intermediates **15**. Since we suspected the thermodynamic driving force toward these intermediates to play a key role for the success of the deracemization, we constructed a heat map that provides the quantum chemically determined relative energies depending on the substitution pattern. Here, the relative energies of possible enol and enamine intermediates originating from deracemization of the 4-aza derivative, as well as other aza derivatives, are shown. The map provides insight into the thermodynamically preferred intermediate to be formed in the bHAT process (Scheme 8).

**Scheme 8:**
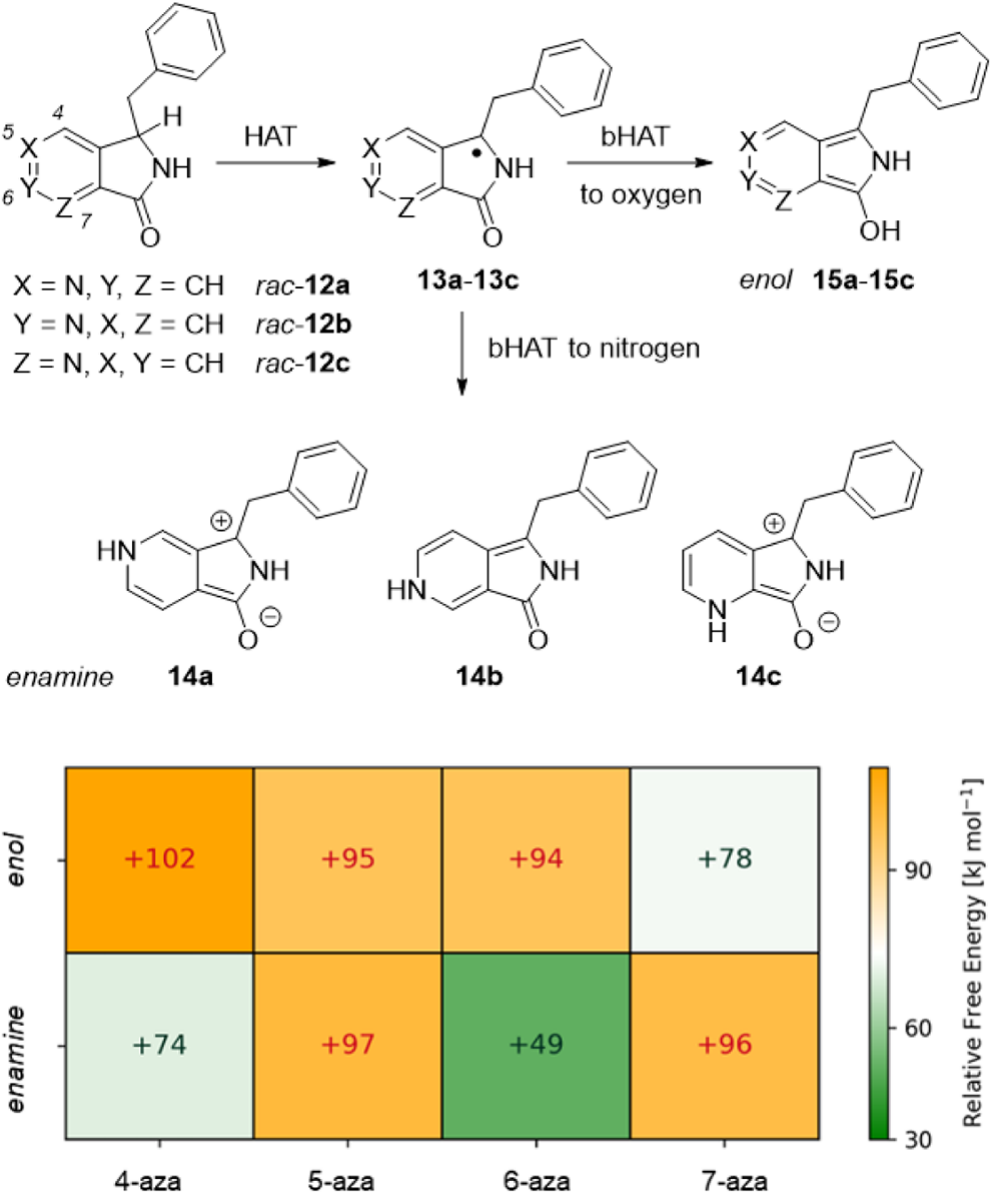
Mechanistic Alternatives for the Back HAT (bHAT) to a Radical Intermediate **13** Formed from Different Aza-1-isoindolinones **12** and Heat Map (See the Supporting Information for Computational Details on the Gibbs Free Energies^22^) for the Various Intermediates

It can be immediately seen that the enamine form is clearly preferred over the enol form (+74 kJ mol^–1^ vs +102 kJ mol^–1^) for the 4-aza compound supporting our previous mechanistic suggestion and explaining the facile deracemization. Likewise, the 6-aza-1-isoindolinone *rac*-**12b** is expected to take this pathway with a strong preference for the enamine intermediate **14b** (+49 kJ mol^–1^ vs +94 kJ mol^–1^). The zwitterionic nature of intermediate **14a** (+97 kJ mol^–1^) and **14c** (+96 kJ mol^–1^) seems to disfavor the bHAT to the nitrogen atom for substrates *rac*-**12a** and *rac*-**12c**. However, 7-aza-1-isoindolinone *rac*-**12c** appears to have an alternative option for bHAT via the oxygen atom and enol intermediate **15c** (+78 kJ mol^–1^). The situation for 5-aza-1-isoindolinone *rac*-**12a** is the least favorable because both the enamine intermediate **14a** (+97 kJ mol^–1^) and the enol intermediate **15a** (+95 kJ mol^–1^) are relatively high in energy, making a bHAT energetically costly and possibly stalling the reaction by irreversible formation of a catalyst-substrate adduct.

The MECP geometries^21d,21e^ for bHAT from radical **13b** (6-aza derivative) to enamine **14b** and from radical **13c** (7-aza derivative) to enol **15c** are illustrated in Figure 6. They demonstrate that a geometric rearrangement of the noncovalent complexes is required to give access to a suitable coordinate for bHAT. In the noncovalent complex **13b**·**8b**, the hydrogen bonding motif needs to be cleaved, and rotation of the oxazole unit has to occur to access the required bHAT coordinate. In the rearranged conformation, the bHAT distance at the MECP is reduced to 178 pm only, thereby enabling formation of enamine **14b**. In the other example, bHAT in **13c**·**8b** can occur to enol **15c** upon rotation of the oxazole unit of **8b** without the need to cleave the hydrogen bonds. Once the conformational change is established, the distance for bHAT, *d*_bHAT_ = 214 pm is sufficiently small for HAT to proceed to the carbonyl oxygen of radical **13c** leading to the thermodynamically favorable enol **15c** (see also Scheme 8).

**Figure 6:**
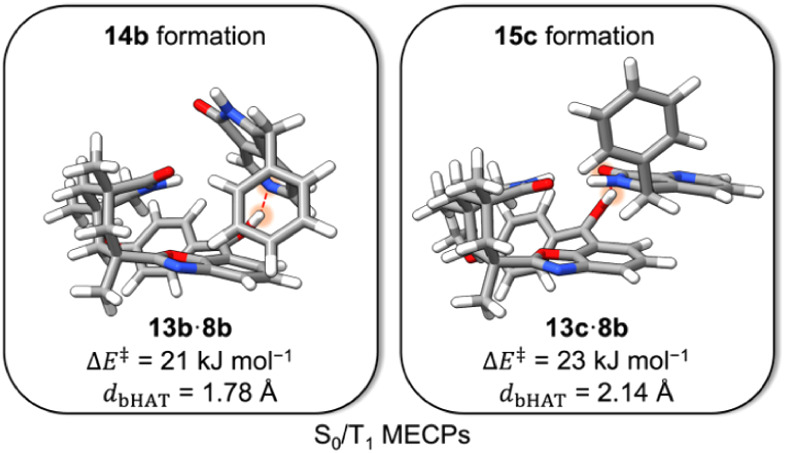
Selected S_0_/T_1_ MECP geometries of **13b**·**8b** (left) and **13c**·**8b** (right) illustrating suitable backward hydrogen atom transfer (bHAT) coordinates to form the enamine **14b** and enol photoproduct **15c**, respectively. MECPs are calculated at the UKS-PBEh-3c+CPCM^21d,21e^ level of theory (see the Supporting Information for details). The distance for bHAT *d*_bHAT_ is given for the involved atoms and highlighted in orange color. In addition, the relative energy difference Δ*E* ^‡^ between the T_1_ minimum after HAT and the MECP is provided.

The predictions based on the quantum chemical calculations qualitatively agreed with the experimental results. The three aza-1-isoindolinones *rac*-**12a**, *rac*-**12b**, and *rac*-**12c** were subjected to the optimized reaction conditions identified for 4-aza-1-isoindolinone *rac*-**3a**. The 5-aza derivative *rac*-**12a** performed poorly, and the desired product **12a** was isolated in only 23% *ee* (Figure 7). In stark contrast, the 6-aza-1-isoindolinone *rac*-**12b** could be smoothly deracemized and was obtained as compound **12b** in almost enantiomerically pure form (98% *ee*). The 7-aza compound resulted in a good enantioselectivity (82% *ee*) for enantiomer **12c** but did not reach the high enantioselectivities achieved with the 4- and 6-aza-1-isoindolinones.

**Figure 7:**
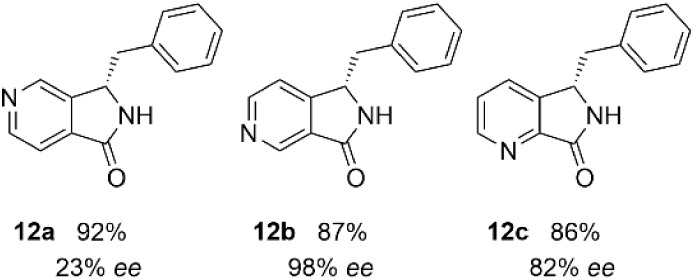
Aza-1-isoindolinones **12** obtained by photochemical deracemization under the conditions optimized for 4-aza-1-isoindolinone *rac*-**3a**.

Since additional quantum chemical calculations of the 5-aza zwitterionic intermediate **14a** revealed a significantly stronger stabilization in polar solvents (see Figure S17 in the Supporting Information), we concluded the study on the aza-1-isoindolinones by subjecting compound *rac*-**12a** to a photochemical deracemization in acetonitrile solution. The reaction was not as clean as the reactions performed in trifluorotoluene solution but product **12a** could be isolated in a moderate yield of 56%. Remarkably, its enantioselectivity was significantly higher (96% *ee*), however, corroborating the relevance of the bHAT step for the outcome of the photochemical deracemization.

## Conclusion

In summary, the present study has disclosed a broadly applicable route for the generation of 4-aza-, 6-aza-, and 7-aza-isoindolinones in enantiomerically pure form. Since ring opening of the five-membered lactam ring is facile, six-membered nitrogen heterocycles with an adjacent stereogenic center are readily accessible. Even more importantly, the mechanistic work on the photochemical deracemization has provided pivotal insights into the mode of action of chiral benzophenone catalysts. The formation of a 1:1 adduct **11** between the catalyst and the substrate had previously been considered an irreversible decomposition pathway but has now been found to lead to a catalyst resting state from which the active catalyst can be regenerated. In accordance with quantum chemical calculations, the photolytic cleavage of the C–C bond within adducts **11** has been established by crossover experiments and by transient absorption spectroscopy. It is conceivable that previously unsuccessful deracemization reactions may be viable upon proper electronic modulation of the chromophore at the benzophenone catalyst. A second mechanistic key finding relates to the nature of the intermediate formed by bHAT from the protonated ketyl radical **8b** to the radical intermediate. Here, we have identified the calculated thermodynamic stability of the formed intermediate (enol, enamine) as a helpful marker to predict the course of this critical event. The linkage of the catalytically active aromatic ketone to its chiral 1,5,7-trimethyl-3-azabicyclo[3.3.1]­nonan-2-one backbone appears to allow for more conformational freedom than previously anticipated and enables bHAT also to positions which are not immediately accessible from the conformer that accounts for the forward HAT.

## Supplementary Material



## Data Availability

The data that support the findings of this study are available in the Supporting Information of this article. Primary research data are openly available in the Chemotion Repository at: 10.14272/collection/PFR_2026-02-18. The primary data from the computational study (minimum and crossing point geometries, input, and output files) generated and used in this study are openly available in the Zenodo repository at: 10.5281/zenodo.19016974.

## Notes

Experimental details; materials; and methods; analytical data and NMR spectra for all new compounds; details and supplementary results of the quantum chemical calculations; optimized geometries of the molecules considered in the computational study; input and output files (PDF)
