# Supplementary material for: Photochemical Deracemization of Aza-1-isoindolinones: Critical Influence of Catalyst Substitution and the Nature of Its Resting State
Source: J Am Chem Soc. 2026 May 30;148(22):23133–43. doi: 10.1021/jacs.6c05440 (PMC13266994; doi:10.1021/jacs.6c05440)
Supplement: Supplementary file 1 [file ja6c05440_si_001.pdf]

Supporting Information for

Photochemical Deracemization of Aza-1-isoindolinones: Critical Influence of Catalyst Substitution and the Nature of its Resting State

Philip Freund,[a‡] Mike Pauls,[b‡] Miriam Jänchen,[a] Julian Zuber,[a] Jürgen Hauer,[a] Christoph Bannwarth,[b*] and Thorsten Bach[a*]

[a] Department Chemie and Catalysis Research Center (CRC), School of Natural Sciences, Technische Universität München, D-85747 Garching, Germany;

[b] Institut für Physikalische Chemie, RWTH Aachen University, D-52074 Aachen, Germany.

Correspondence to: bannwarth@pc.rwth-aachen.de, thorsten.bach@ch.tum.de

Table of Contents

1. General Information 3

2. Analytical Methods 7

3. General Procedures 9

4. Synthesis of Benzophenone Catalysts 14

5. Characterization of **(–)-2b** 29

6. Condition Optimization and Kinetic Studies 34

7. Crystallographic Data 37

9. Synthesis of Racemic Substrates 45

10. Photochemical Deracemization Reactions 85

11. Downstream Synthetic Transformations 99

12. Isolation and Analysis of **11a** and **11p** 104

13. Transient Absorption Spectroscopy of Compound **11a** 108

14. NMR Spectra 111

15. Chiral HPLC Traces 176

16. References 211

1. General Information

All reactions sensitive to air or moisture, were carried out in dried glassware (600 °C) under positive pressure of argon using standard Schlenk techniques.

Commercially available chemicals were used without further purification, unless otherwise mentioned. For moisture sensitive reactions, dichloromethane (CH_2Cl_2) and tetrahydrofuran (THF) were purified using a MBSPS 800 *MBraun* solvent purification system. The following columns were used:

CH_2Cl_2: 2 × MB-KOL-A type (aluminum oxide)

Et_2O: 1 × MB-KOL-A type 2 (aluminum oxide), 1 × MB-KOL-M type 2 (3 Å molecular sieve)

THF: 2 × MB-KOL-M type 2 (3 Å molecular sieve)

Anhydrous *α,α,α*-trifluorotoluene ($PhCF_3$) was purchased from *Sigma Aldrich (Merck).* $PhCF_3$ used for photochemical deracemization reactions was additionally stored over 3 Å molecular sieves. Anhydrous acetonitrile (MeCN), chloroform ($CHCl_3$), dichloroethane (DCE), dimethylformamide (DMF), methanol (MeOH) and toluene ($PhCH_3$), were purchased from *Thermo Fisher Scientific* and stored over 3 Å molecular sieves.

Technical solvents for column chromatography [chloroform (CH_3Cl), diethyl ether (Et_2O), ethyl acetate (EtOAc), methanol (MeOH), *n*-hexane (Hex)] were used after simple distillation. Normal-phase flash column chromatography (FCC) was performed on silica 60 (*Merck*, 230-400 mesh) with the indicated eluent mixture.

Commercially available chemicals were purchased either from *Sigma Aldrich (Merck)*, *TCI Chemicals*, *ABCR* or *BLDpharm* and, were used without further purification, if not further mentioned.

Unless otherwise stated, photochemical reactions at $\lambda = 350$ nm were carried out in Duran phototubes (10 or 200 mL) under argon atmosphere in a positive geometry setup with a cylindrical array of 16 fluorescent light tubes, UV-A, $\lambda_{max} = 350$ nm (Figures **S1** and **S2**).

Prior to the start of a photoreaction, each reaction mixture was degassed by being sparged with argon under ultrasonication for at least 15 min.

Lehrstuhl OC 1 - TUM

200 nm | 250 nm | 300 nm | 350 nm | 400 nm | 450 nm | 500 nm | 550 nm | 600 nm | 650 nm

Datasheet FLT024 — Philipps-BLB-365

Basic Information

Type	Fluorescent light tube
Description	Philipps TL 8W BLB
Manufacturer / Supplier	Philipps / Beleuchtungdirekt.de
Order number / Date of purch.	n/a / 02/2021
Internal lot / serial number	2021-02 / FLT024

Specification Manufacturer

Type / size	T5 tube, G5 socket
Mechanical specification	16 mm diameter, 288 mm length
Electrical specification	8 W
Wavelength (range, typ.)	350 - 400 nm
Spectral width (FWHM)	~ 16 nm
Datasheet	n/a

Characterization

Description of measurement	Measured with Ocean-optics USB4000 spectrometer using a calibrated setup (cosine corrector/fibre). The cosine corrector was placed at 20 mm distance from a single fluorescent tube at half height.	
Measured dominant wavelength / Int.	365 nm	168 $\mu W/mm^2nm$
Measured spectral width (FWHM)	16 nm	
Integral Reference intensity / range	3059 $\mu W/cm^2$	300-450 nm

Spectrum

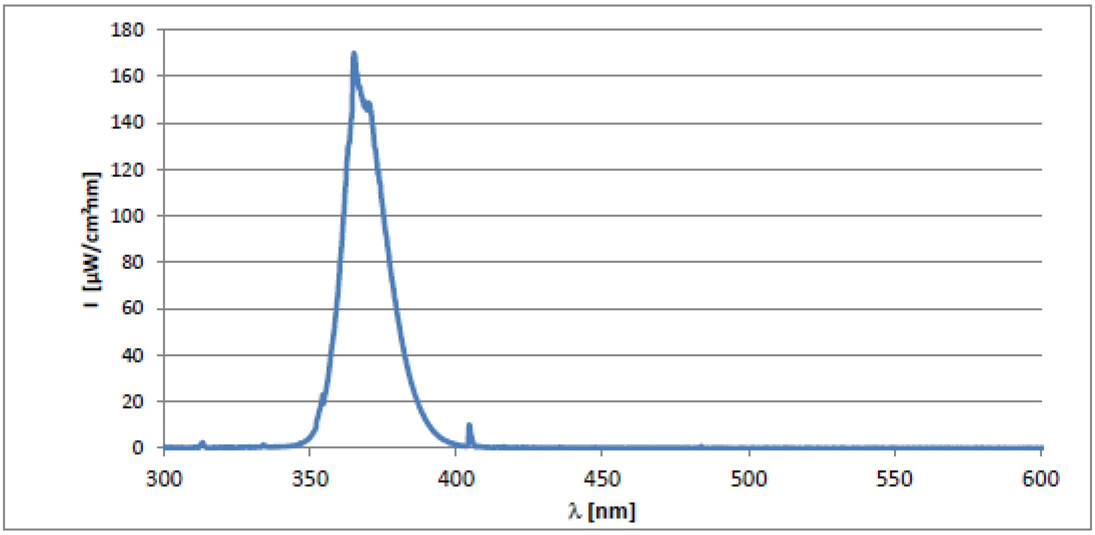

Figure S1: Emission spectrum of the 366 nm fluorescent light tube.

Lehrstuhl OC 1 - TUM

200 nm | 250 nm | 300 nm | 350 nm | 400 nm | 450 nm | 500 nm | 550 nm | 600 nm | 650 nm

Datasheet FLT021 LZC-UVA

Basic Information

Type	Fluorescent light tube
Description	Luzchem LZC-UVA
Manufacturer / Supplier	Hitachi / Luzchem
Order number / Date of purch.	LZC-UVA / 09/2015
Internal lot / serial number	2015-09 / FLT021

Specification Manufacturer

Type / size	T5 tube, G5 socket
Mechanical specification	16 mm diameter, 288 mm length
Electrical specification	8 W
Wavelength (range, typ.)	300 - 400 nm, 350m nm, UV-A
Spectral width (FWHM)	~ 40 nm
Datasheet	

Characterization

Description of measurement	Measured with Ocean-optics USB4000 spectrometer using a calibrated setup (cosine corrector/fibre). The cosine corrector was placed at 20 mm distance from a single fluorescent tube at half height.	
Measured dominant wavelength / Int.	350 nm	115 µW/mm²nm
Measured spectral width (FWHM)	40 nm	
Integral Reference intensity / range	5017 µW/cm²	300-425 nm

Spectrum

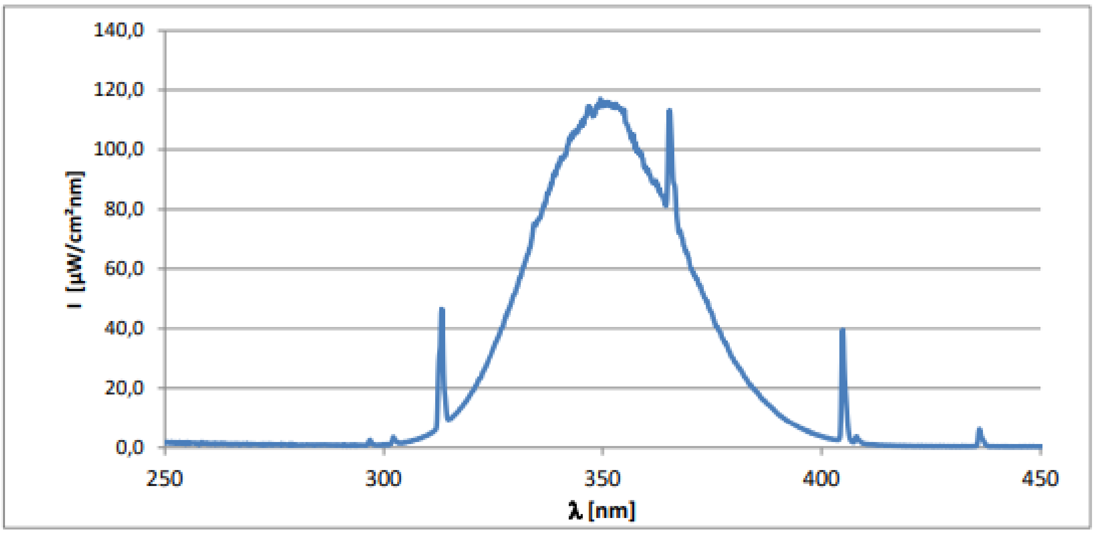

Figure S2: Emission spectrum of the 350 nm fluorescent light tube.

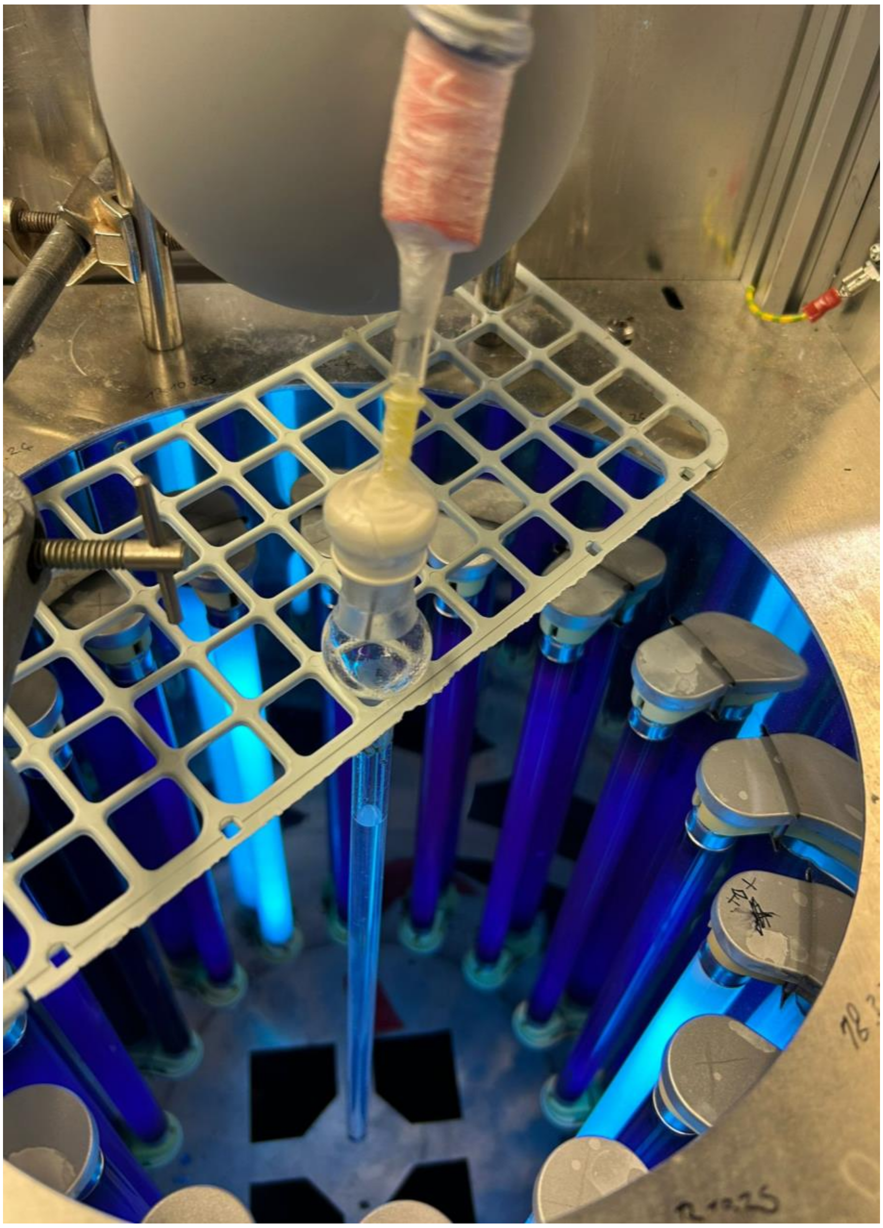

Figure S3: Typical setup for a photoreaction.

2. Analytical Methods

Thin layer chromatography (**TLC**) was performed on silica coated glass plates (silica gel 60 F_{254}) with detection by UV-light ($\lambda = 254$ nm), potassium permanganate stain [potassium permanganate (3.00 g), potassium carbonate (20.0 g), aqueous NaOH solution (5%, 5.0 mL), water (300 mL)]. or a ninhydrine stain [2,2-dihydroxy-1*H*-indene-1,3(2*H*)-dione (0.30 g), acetic acid (3 mL), *n*-butanol (100 mL)].

Infrared spectra (**IR**) were recorded on a *Perkin Elmer* Frontier IR-FTR spectrometer by ATR technique. The signal intensity is assigned using the following abbreviations: s (strong), m (medium), w (weak). The following abbreviations were used: aliph = aliphatic, arom = aromatic.

Melting points (**M.p.**) were determined using a Kofler ("Thermopan", Fs *Reichert*, Wien) apparatus.

Nuclear magnetic resonance (**NMR**) (^{1}H, ^{13}C and ^{19}F-NMR) spectra were recorded at room temperature (r.t.) on either a *Bruker* AVHD-400, AVHD-500, or a *Bruker* AV-II-500 equipped with cryo probe head. Chemical shifts of the NMR spectra are reported relative to $CHCl_3$ (^{1}H-NMR: $\delta = 7.26$ ppm, ^{13}C-NMR: $\delta = 77.16$ ppm), MeOH (^{1}H-NMR: $\delta = 3.31$ ppm, ^{13}C-NMR: $\delta = 49.00$ ppm) or DMSO (^{1}H-NMR: $\delta = 2.50$ ppm, ^{13}C-NMR: $\delta = 39.52$ ppm). The data are reported as follows: chemical shift (δ) [multiplicity, coupling constant *J* (Hz), relative integral, number of protons] where multiplicity is defined as: m = multiplet, s = singlet, d = doublet, t = triplet, q = quartet, br = broad. Apparent multiplets which occur because of coupling constant equality between magnetically non-equivalent protons are marked as virtual (*virt.*).

Mass spectrometry (**MS**) and **high-resolution mass spectrometry** (**HRMS**) were measured on a *Thermo Scientific* LTQ-FT Ultra (ESI).

Specific Rotation was determined using an ADP440+ polarimeter (Fa *Bellingham+Stanley*) and is reported as follows: $[\alpha]_{D}^{T}$ (c in g per 100 mL solvent). The polarimeter has a variance of ± 0.001 which translates to a variance of ± 2 for the measured rotation at a concentration of c = 1.0 and a cuvette path length of l = 0.5 cm.

High Performance Liquid Chromatography (**HPLC**) was performed using a chiral stationary phase [ChiralPak AD-H (250 × 4.6 mm), ChiralPak AS-H (250 × 4.6 mm), Chiralpak IC (250 × 4.6 mm), Chiralpak IA (250 × 4.6 mm), *Daicel Chemical Industries*] with UVD 340 Photodiode Array Detector, P580 Pump and an ASI-100 Automated Sample Injector at 20 °C. For normal-phase HPLC a *Daicel* ChiralPak AD-H, ChiralCel OJ-H, ChiralPak IC, ChiralPak IA and a ChiralPak AS-H was used as stationary phase, and a mixture of *n*-heptane/*i*-propanol was used as mobile phase.

Luminescence spectroscopy was performed on a Horiba Scientific FluoroMax-4P spectrofluorometer equipped with a continuous Xe source for steady state spectra and a Xe flashlight source for the observation of phosphorescence spectra. Spectra were recorded in Horiba quartz tubes (inner ⌀ = 4 mm) in a small quartz Dewar vessel which was filled with liquid nitrogen (77 K) with a delay of 50 µs. Trifluorotoluene was used as the solvent, and solutions prepared and handled under dry nitrogen atmosphere.

3. General Procedures

General Procedure A (GP A): Synthesis of methyl 2-(bromomethyl)nicotinates

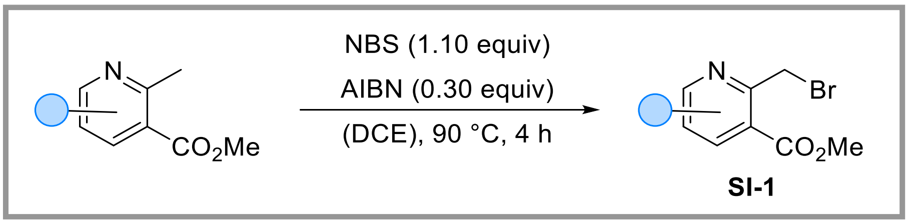

According to a modified procedure,[1] *N*-bromosuccinimide (NBS) (1.10 equiv.) and azobisisobutyronitrile (AIBN) (0.30 equiv.) were added to a solution of methyl 2-methylnicotinate derivative (1.00 equiv.) in DCE (0.64 M) at room temperature. The mixture was refluxed for four hours while the solution turned dark orange or dark red depending on the substrate. Subsequently, the mixture was cooled to room temperature, poured into a mixture of *n*-pentane:EtOAc (9:1) and stirred for ten minutes. The resulting precipitate was removed by filtration through a plug of Celite© and the solvent was removed under reduced pressure. The crude product was subjected to FCC (SiO_2, Hex/Et_2O) to yield the desired product **SI-1**.

General Procedure B (GP B): Synthesis of 6-(4-methoxybenzyl)-6,7-dihydro-5*H*-pyrrolo[3,4-*b*]pyridin-5-ones

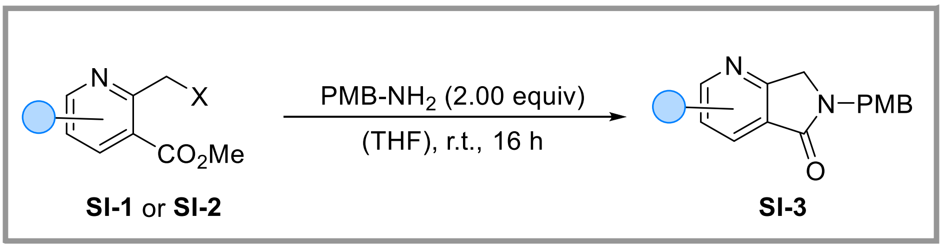

According to a modified procedure,[2] 4-methoxybenzylamine (PMB-NH_2) (2.00 equiv.) was added to a solution of **SI-1** or **SI-2** (1.00 equiv.) in THF (0.3 M) at room temperature in one portion. The resulting suspension was stirred for 16 hours at the same temperature. The reaction was quenched by addition of a sat. ammonium chloride solution and water and the mixture was extracted with EtOAc. The combined organic layers were washed with brine and dried over Na_2SO_4. After filtration, the solvent was removed under reduced pressure, and the crude product was subjected to FCC (SiO_2, Hex/EtOAc) to yield the desired product **SI-3.**

General Procedure C (GP C): Direct synthesis of 6-(4-methoxybenzyl)-6,7-dihydro-5*H*-pyrrolo[3,4-*b*]pyridin-5-ones

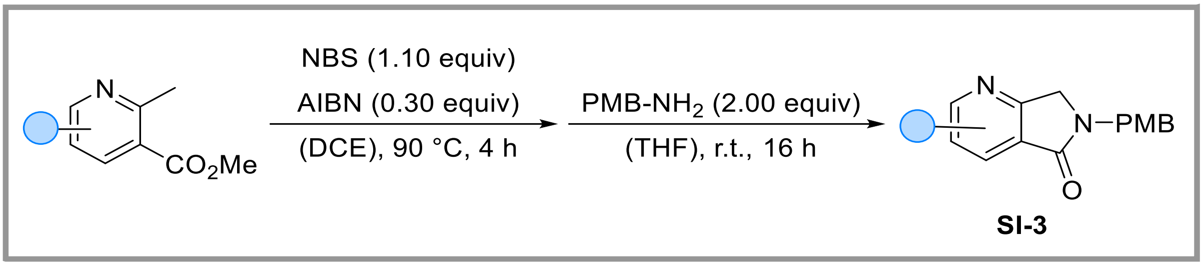

According to a modified procedure,[1,2] NBS (1.10 equiv.) and AIBN (0.30 equiv.) were added to a solution of methyl 2-methylnicotinate derivative (1.00 equiv.) in DCE (0.64 M) room temperature. The mixture was refluxed for four hours while the solution turned dark orange or dark red depending on the substrate. Afterwards, the mixture was cooled to room temperature, poured into a mixture of *n*-pentane:EtOAc (9:1) and stirred for ten minutes. The resulting precipitate was filtered off over a plug of Celite© and the solvent was removed under reduced pressure. Due to insufficient separation according to TLC, the crude product was dissolved in THF (0.3 M) and (PMB-NH_2) (2.00 equiv.) was added at room temperature in one portion. The resulting suspension was stirred for 16 hours at the same temperature. The reaction was quenched by the addition of sat. ammonium chloride solution and water and the mixture was extracted with EtOAc. The combined organic layers were washed with brine and dried over Na_2SO_4. After filtration, the solvent was removed under reduced pressure, and the crude product was subjected to FCC (SiO_2, Hex/EtOAc) to yield the desired product **SI-3.**

General Procedure D (GP D): Synthesis of racemic 3-benzyl-substituted 4-azaisoindolinones

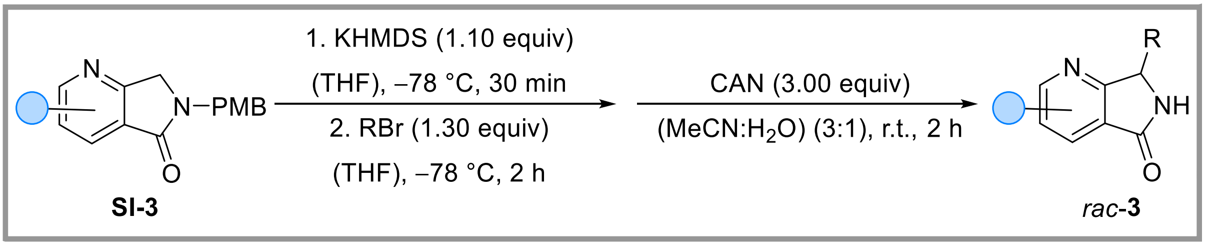

Following a modified procedure,[2] a 1 M solution of KHMDS in THF (1.10 equiv.) was added dropwise to a solution of 6-(4-methoxybenzyl)-6,7-dihydro-5*H*-pyrrolo[3,4-*b*]pyridin-5-one derivative (**SI-3**) (1.00 equiv.) in anhydrous THF (0.08 M) at –78 °C, upon which the reaction mixture changed color depending on the substitution at 4- or 5- position of the substrate. After 20 minutes, the respective bromide (1.30 equiv.) was added in one portion. The reaction was left to stir for two hours at –78 °C. Subsequently, the solution was allowed to warm up to room

temperature and quenched by the addition of sat. NH_4Cl and distilled water. The aqueous layer was extracted with EtOAc, and the combined organic phases were dried over Na_2SO_4, filtered and concentrated under reduced pressure. The crude product was then dissolved in MeCN:H_2O (3:1, 0.03 M), and diammonium cerium(IV) nitrate (CAN) (3.00 equiv.) was added to the solution at room temperature. The yellow solution was stirred for two hours at the same temperature. Subsequently, water was added, and the mixture was extracted with EtOAc. The combined organic layers were washed with brine and dried over Na_2SO_4. After filtration, the solvent was removed under reduced pressure, and the residual crude product was subjected to FCC (SiO_2, Hex/EtOAc) to yield the desired racemic substrate *rac*-**3.**

General Procedure E (GP E): Synthesis of racemic 3-aliphatic-substituted 4-azaisoindolinones

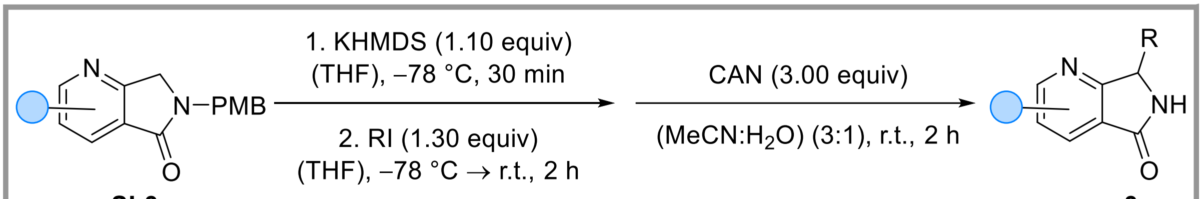

SI-3 *rac*-**3**

Following modified procedure,[2] a 1 M solution of KHMDS in THF (1.10 equiv.) was added dropwise to a solution of 6-(4-methoxybenzyl)-6,7-dihydro-5*H*-pyrrolo[3,4-*b*]pyridin-5-one derivative (**SI-3**) (1.00 equiv.) in anhydrous THF (0.08 M) at –78 °C, upon which the reaction mixture changed color depending on the substitution at 4- or 5- position of the substrate. After 20 minutes, the respective bromide (1.30 equiv.) was added in one portion. The reaction was left to stir for two hours at room temperature. Subsequently, the reaction was quenched by the addition of sat. NH_4Cl and distilled water. The aqueous layer was extracted with EtOAc, and the combined organic phases were dried over Na_2SO_4, filtered and concentrated under reduced pressure. The crude product was then dissolved in MeCN:H_2O (3:1, 0.03 M), and CAN (3.00 equiv.) was added to the solution at room temperature. The yellow solution was stirred for two hours at the same temperature. Subsequently, water was added, and the mixture was extracted with EtOAc. The combined organic layers were washed with brine and dried over Na_2SO_4. After filtration, the solvent was removed under reduced pressure, and the residual crude product was subjected to FCC (SiO_2, Hex/EtOAc) to yield the desired racemic substrate *rac*-**3.**

General Procedure F (GP F): Synthesis of racemic 3-alkoxy-substituted 4-azaisoindolinones

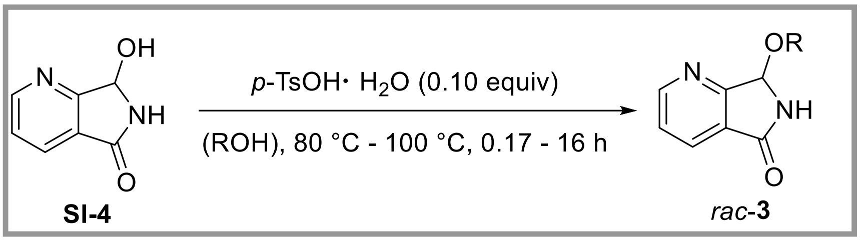

Following a modified procedure,[2] *para*-toluenesulfonic acid (*p*-TsOH) (0.10 equiv.) was added to a suspension of 7-hydroxy-6,7-dihydro-5*H*-pyrrolo[3,4-*b*]pyridin-5-one (**SI-4**) (1.00 equiv.) in the respective alcohol (20.0 equiv.). The resulting mixture was then stirred between 80 °C and 100 °C until a clear solution prevailed. Afterwards, the mixture was allowed to cool to room temperature and was quenched by addition of a sat. bicarb solution. The mixture was extracted with CH_2Cl_2, and the combined organic layers were washed with brine and dried over Na_2SO_4. After filtration, the solvent was removed under reduced pressure, and the residual crude product was subjected to FCC (SiO_2, Hex/EtOAc) to yield the desired racemic substrate *rac*-**3.**

General Procedure G (GP G): Synthesis of racemic 3-silyloxy-substituted 4-azaisoindolinones

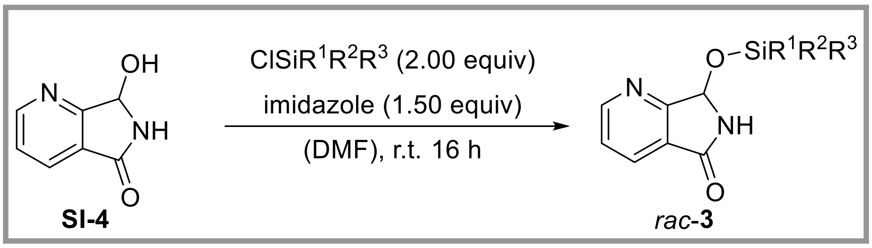

Following a modified procedure,[2] imidazole (1.50 equiv.) and the respective chlorosilane (2.00 equiv.) were added to a solution of 7-hydroxy-6,7-dihydro-5*H*-pyrrolo[3,4-*b*]pyridin-5-one (**SI-4**) (1.00 equiv.) in DMF (0.2 M) at room temperature. The resulting mixture was stirred for 16 hours at the same temperature. Subsequently, the mixture was quenched by the addition of a sat. NH_4Cl solution. The mixture was extracted with CH_2Cl_2, and the combined organic layers were washed with brine and dried over Na_2SO_4. After filtration, the solvent was removed under reduced pressure, and the residual crude product was subjected to FCC (SiO_2, Hex/EtOAc) to yield the desired racemic substrate *rac*-**3**.

General Procedure H (GP H): Suzuki coupling

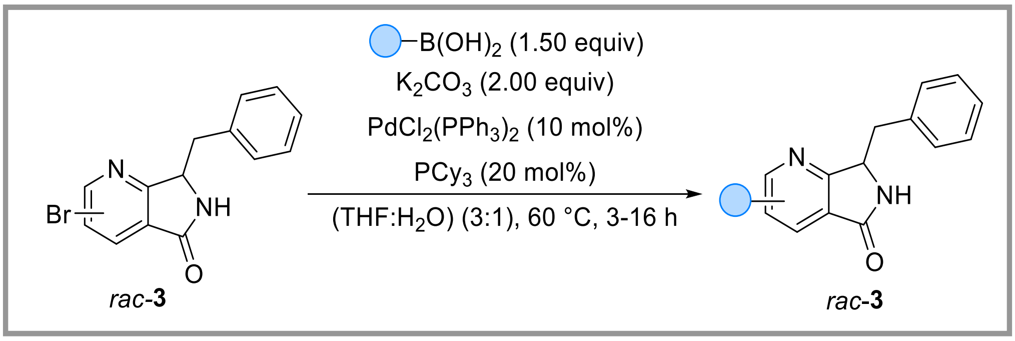

Following a modified procedure,[3] the respective boronic acid (1.50 equiv.), K_2CO_3 (2.00 equiv.), $PdCl_2(PPh_3)_2$ (10 mol%) and PCy_3 (20 mol%) were added to a solution of bromide **3l** or *rac*-**3o** (1.00 equiv.) in THF/water (3:1, 0.04 M). The resulting mixture was degassed by freeze-pump thaw cycling thrice and then stirred at 60 °C for three to 16 hours. Afterwards, the mixture was allowed to cool to room temperature water was added and the aqueous layer was extracted with EtOAc. The combined organic layers were washed with brine and dried over Na_2SO_4. After filtration, the solvent was removed under reduced pressure, and the residual crude product was subjected to FCC (SiO_2, Hex/EtOAc) to yield the desired racemic substrate *rac*-**3q** or **3x**.

General Procedure I (GP I): Photochemical deracemization

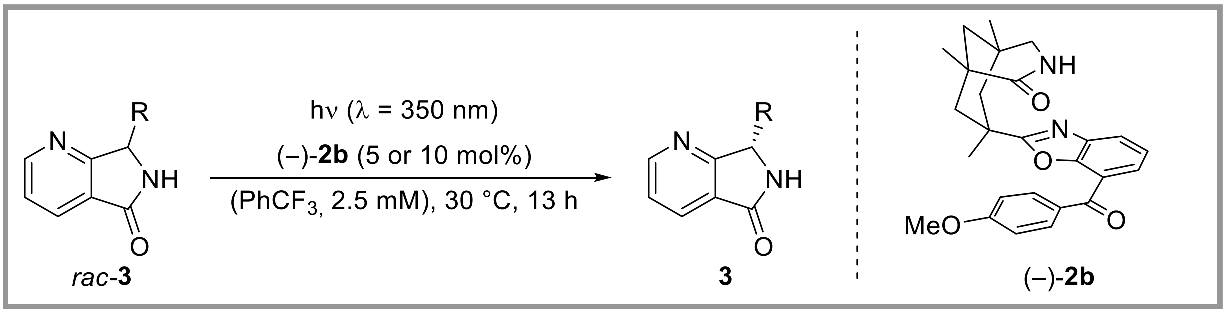

The corresponding racemic 3-substituted 4-azaisoindolinones *rac*-**3** (25.0 µmol, c = 2.5 mM, 1.00 equiv.) and enantiomerically pure (–)-benzophenone **2b** (5 or 10 mol%) were dissolved in 10 mL α,α,α-trifluorotoluene ($PhCF_3$) in a dried phototube (Ø = 1 cm). The solution was degassed by bubbling argon through the solution for 15 minutes under ultrasonication and then irradiated at λ = 350 nm for 13 hours. The solvent was evaporated under reduced pressure, and the crude product was subjected to flash column chromatography (SiO_2, Hex/EtOAc) to yield the enantiomerically enriched products **3**. The enantiomeric excess as well as the specific rotation were determined from the purified enantioenriched/enantiopure products, which were obtained as white solids.

4. Synthesis of Benzophenone Catalysts

2-Methoxy-3-nitrobenzaldehyde (SI-5)

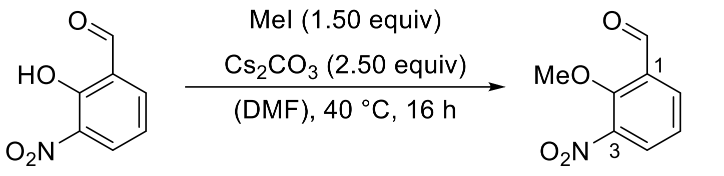

SI-5
$C_8H_7NO_4$
M = 181.15 g mol^{-1}

Following a modified procedure,[4] cesium carbonate (19.5 g, 59.8 mmol, 2.50 equiv.) was added to a solution of 2-hydroxy-3-nitrobenzaldehyde (4.00 g, 23.9 mmol, 1.00 equiv.) in DMF (60 mL) at room temperature. The resulting, orange-colored suspension was stirred ten minutes at the same temperature. Subsequently, methyl iodide (2.23 mL, 5.10 g, 35.9 mmol, 1.50 equiv.) was added and the resulting mixture was stirred for 16 hours at 40°C. Afterwards, the mixture was allowed to cool to room temperature and then was poured into 400 mL ice water and stirred for ten minutes. The precipitate was filtered off and washed with water (3 × 50 mL) until the pH of the filtrate was neutral. The remaining solid was dried in the airflow and *in vacuo* to yield the desired 2-methoxy-3-nitrobenzaldehyde (**SI-5**) (3.65 g, 20.5 mmol, 84%) as a light-yellow solid.

TLC (Hex:EtOAc = 4:1): R_f = 0.59 [UV] [$KMnO_4$].

M.p.: 59 °C.

^{1}H-NMR (400 MHz, $CDCl_3$, 300 K): δ [ppm] = 10.41 (d, ^5J = 0.7 Hz, 1H, CHO), 8.11 – 8.06 (m, 2H, H4, H6), 7.36 (*virt.* td, $^3J \approx {}^3J \approx$ 8.0 Hz, ^5J = 0.7 Hz, 1H, H5), 4.08 (s, 3H, OCH_3).

^{13}C-NMR (101 MHz, $CDCl_3$, 300 K): δ [ppm] = 187.7 (CHO), 156.4 (C2), 144.3 (C1), 133.5 (C4), 131.5 (C3), 131.0 (C6), 124.5 (C5), 65.5 (OCH_3).

HRMS (**ESI**) *m/z* $[M+H]^+$ calculated for $[C_8H_8NO_4]^+$: 182.0448, found: 182.0445.

IR (film): $\tilde{\nu}$ max/cm^{-1} = 3085 (w, CH_{arom}), 2892 (w, $COCH_3$) 1691 (C=O), 1525 (s, $C_{arom}NO_2$), 1599 (m, $C=C_{arom}$), 1457 (m, $C=C_{arom}$).

(2-Methoxy-3-nitrophenyl)(4-methoxyphenyl)methanol (SI-6)

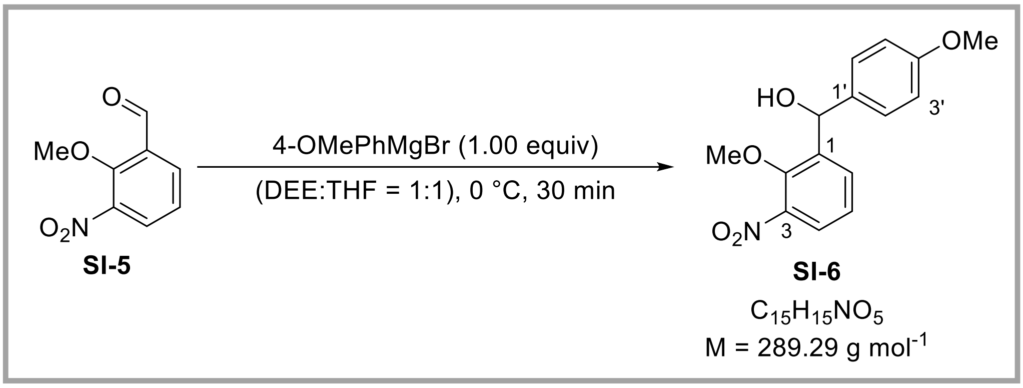

Following a modified procedure,[5] a solution of 4-methoxyphenylmagnesium bromide (22.1 mL, 2.33 g, 11.0 mmol, 1.00 equiv.) was added dropwise to a solution of 2-methoxy-3-nitrobenzaldehyde (**SI-5**) (2.00 g, 11.0 mmol, 1.00 equiv.) in Et_2O:THF (1:1, 70 mL) at 0 °C. The resulting dark red solution was stirred for 30 minutes at the same temperature. Afterwards, the reaction was quenched by addition of sat. NH_4Cl solution (100 mL) and water (50 mL) and the aqueous layer was extracted with EtOAc (3 × 100 mL). The combined organic layers were washed with brine (100 mL) and dried over Na_2SO_4. The solvent was removed under reduced pressure, and the residual crude product was subjected to FCC (SiO_2, Hex:EtOAc = 4:1 → 3:1) to yield the desired (2-methoxy-3-nitrophenyl)(4-methoxyphenyl)methanol (**SI-6**) (2.99 g, 10.3 mmol, 94%) as a yellow oil.

TLC (Hex:EtOAc = 3:1): R_f = 0.50 [UV] [$KMnO_4$].

^{1}H-NMR (400 MHz, $CDCl_3$, 300 K): δ [ppm] = 7.88 – 7.79 (m, 2H, H4, H6), 7.34 – 7.24 (m, 3H, H5, H2', H6'), 6.95 – 6.87 (m, 2H, H3', H5'), 6.15 (d, ^3J = 3.3 Hz, 1H, C*H*OH), 3.83 (s, 3H, C4'-OCH_3), 3.68 (s, 3H, C2-OCH_3), 2.58 (bs, 1H, OH).

^{13}C-NMR (101 MHz, $CDCl_3$, 300 K): δ [ppm] = 159.5 (C4'), 151.1 (C2), 143.5 (C3), 140.4 (C1), 135.0 (C1'), 132.5 (C4), 128.1 (C2', C6'), 124.9 (C6), 124.1 (C5), 114.2 (C3', C5'), 70.7 (C-OH), 62.6 (C4'-O*C*H_3), 55.4 (C2-O*C*H_3).

HRMS (**ESI**) *m/z* $[M-OH]^+$ calculated for $[C_{15}H_{14}NO_4]^+$: 272.0917, found: 272.0915.

IR (film): $\tilde{v}$ max/cm^{-1} = 3415 (w, OH), 3082 (m, CH_{arom}), 2837 (m, OCH_3), 1607 (m, $C{=}C_{arom}$), 1524 (s, $C_{arom}NO_2$), 1459 (m, $C{=}C_{arom}$), 1242 (s, $C_{arom}NO_2$).

(2-Methoxy-3-nitrophenyl)(4-methoxyphenyl)methanone (SI-7)

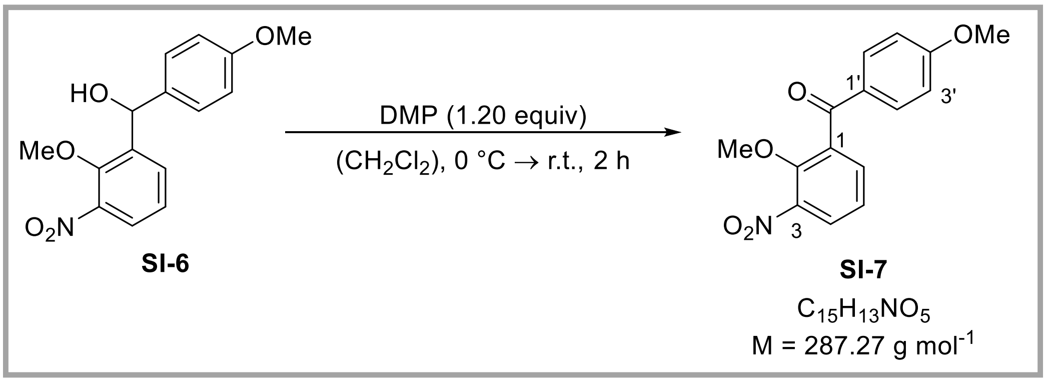

Following a modified procedure,[6] Dess–Martin periodinane (DMP) (3.52 g, 8.30 mmol, 1.20 equiv.) was added in one portion to a solution of (2-methoxy-3-nitrophenyl)(4-methoxyphenyl)methanol (**SI-6**) (2.00 g, 6.91 mmol, 1.00 equiv.) in CH_2Cl_2 (51 mL) at 0 °C. The ice bath was removed, and the resulting red solution was stirred for two hours at room temperature. Afterwards, the reaction was quenched by addition of sat. $Na_2S_2O_3$ solution (20 mL) and sat. bicarb (20 mL) and the aqueous layer was extracted with CH_2Cl_2 (3 × 50 mL). The combined organic layers were washed with brine (50 mL) and dried over Na_2SO_4. The solvent was removed under reduced pressure and the residual crude product was subjected to FCC (SiO_2, Hex:EtOAc = 6:1 → 3:1) to yield the desired (2-methoxy-3-nitrophenyl)(4-methoxyphenyl)methanone (**SI-7**) (1.44 g, 5.01 mmol, 73%) as a yellow oil.

TLC (Hex:EtOAc = 6:1): R_f = 0.29 [UV] [$KMnO_4$].

^{1}H-NMR (400 MHz, $CDCl_3$, 300 K): δ [ppm] = 7.92 (dd, ^3J = 8.1 Hz, ^4J = 1.7 Hz, 1H, H4), 7.85 – 7.75 (m, 2H, H2’, H6’), 7.56 (dd, ^3J = 7.6 Hz, ^4J = 1.7 Hz, 1H, H6), 7.30 (*virt.* t, $^3J \approx {}^3J \approx$ 7.9 Hz, 1H, H5), 6.99 – 6.91 (m, 2H, H3’, H5’), 3.88 (s, 3H, C4’-OCH_3), 3.78 (s, 3H, C2-OCH_3).

^{13}C-NMR (101 MHz, $CDCl_3$, 300 K): δ [ppm] = 192.7 (CO), 164.6 (C4’), 151.3 (C2), 144.2 (C3), 136.5 (C1), 133.6 (C6), 132.6 (C2’, C6’), 129.5 (C1’), 126.7 (C4), 123.9 (C5), 114.1 (C3’, C5’), 64.0 (C2-OCH_3), 55.7 (C4’-OCH_3).

HRMS (**ESI**) *m/z* $[M+H]^+$ calculated for $[C_{15}H_{14}NO_5]^+$: 288.0866, found: 288.0864.

IR (film): $\tilde{\nu}$ max/cm^{-1} = 3078 (m, CH_{arom}), 2951 (m, CH_{aliph}), 1659 (s, C=O), 1595 (m, C=C_{arom}), 1528 (s, $C_{arom}NO_2$), 1455 (m, C=C_{arom}).

(2-Hydroxy-3-nitrophenyl)(4-methoxyphenyl)methanone (SI-8)

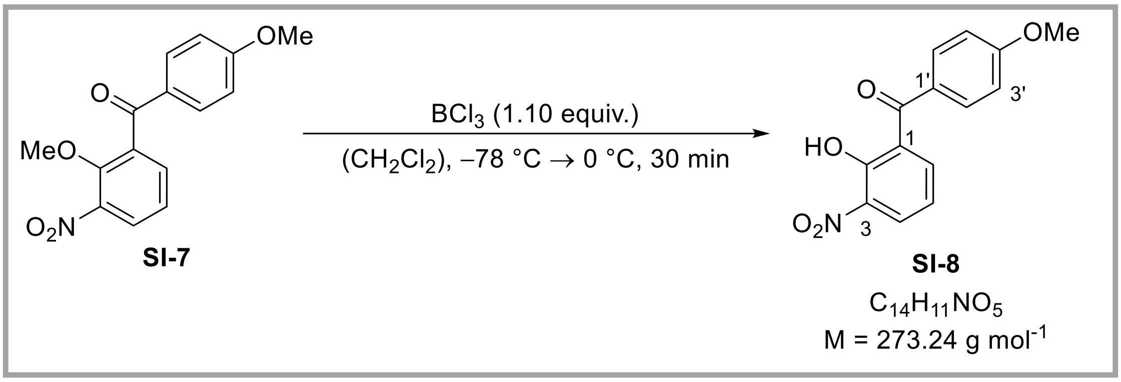

Following a modified procedure,[7] BCl_3 in CH_2Cl_2 (2.76 mL, 323 mg, 2.76 mmol, 1.10 equiv.) was added to a solution of benzophenone (2-methoxy-3-nitrophenyl)(4-methoxyphenyl)methanone (**SI-7**) (720 mg, 2.51 mmol, 1.00 equiv.) in CH_2Cl_2 (25 mL) dropwise at −78 °C, then the mixture was warmed to 0 °C and stirred for 30 minutes. Afterwards, the reaction was quenched by careful addition of water (20 mL), and the aqueous layer was extracted with CH_2Cl_2 (3 × 25 mL). The combined organic layers were washed with brine (25 mL) and dried over Na_2SO_4. The solvent was removed under reduced pressure, and the residual crude product was subjected to FCC (SiO_2, Hex:EtOAc = 4:1) to yield the desired (2-hydroxy-3-nitrophenyl)(4-methoxyphenyl)methanone (**SI-8**) (656 mg, 2.40 mmol, 96%) as a beige solid.

TLC (Hex:EtOAc = 6:1): R_f = 0.27 [UV] [$KMnO_4$].

M.p.: 110 °C.

^{1}H-NMR (400 MHz, $CDCl_3$, 300 K): δ [ppm] = 11.46 (s, 1H, OH), 8.24 (dd, ^3J = 8.4 Hz, ^4J = 1.7 Hz, 1H, H4), 7.84 – 7.75 (m, 2H, H2’, H6’), 7.75 (dd, ^3J = 7.6 Hz, ^4J = 1.7 Hz, 1H, H6), 7.08 (dd, ^3J = 8.4 Hz, ^3J = 7.6 Hz, 1H, H5), 7.02 – 6.95 (m, 2H, H3’, H5’), 3.90 (s, 3H, OCH_3).

^{13}C-NMR (101 MHz, $CDCl_3$, 300 K): δ [ppm] = 194.0 (CO), 164.3 (C4’), 154.0 (C2), 137.5 (C6), 135.5 (C3), 132.4 (C2’, C6’), 129.7 (C1’), 129.1 (C1), 128.3 (C4), 119.4 (C5), 114.1 (C3’, C5’), 55.7 (OCH_3).

HRMS (ESI) *m/z* $[M+H]^+$ calculated for $[C_{14}H_{12}NO_5]^+$: 274.0710, found: 274.0709.

IR (film): $\tilde{v}$max/cm^{-1} = 3212 (m, OH), 3082 (w, CH_{arom}), 2842 (w, OCH_3), 1642 (s, C=O), 1594 (s, , $C{=}C_{arom}$), 1532 (s, $C_{arom}NO_2$), 1446 (m, $C{=}C_{arom}$), 1257 (C=O).

(3-Amino-2-hydroxyphenyl)(4-methoxyphenyl)methanone (SI-9)

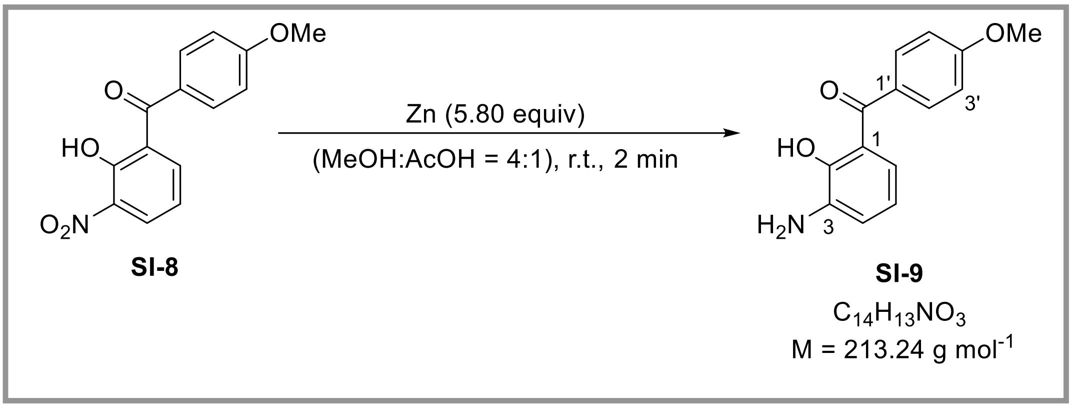

According to a modified procedure,[2] zinc dust (902 mg, 13.8 mmol, 5.80 equiv.) was added in one portion to a solution of (2-hydroxy-3-nitrophenyl)(4-methoxyphenyl)methanone (**SI-8**) (650 mg, 2.38 mmol, 1.00 equiv.) in MeOH:AcOH (57 mL, 4:1) at room temperature. The suspension was stirred for 2 minutes, and the excess zinc dust was filtered through cotton. Water (100 mL) was added to the filtrate, and the aqueous layer was extracted with Et_2O (4 × 60 mL). The combined organic layers were washed with sat. bicarb (150 mL), brine (40 mL) and dried over Na_2SO_4. The solvent was removed under reduced pressure, and the residual crude product was subjected to FCC (SiO_2, Hex:EtOAc = 4:1 → 3:1) to yield the desired (3-amino-2-hydroxyphenyl)(4-methoxyphenyl)methanone (**SI-9**) (354 mg, 1.46 mmol, 61%) as a red oil.

TLC (Hex:EtOAc = 4:1): R_f = 0.26 [UV] [Ninhydrin].

^{1}H-NMR (400 MHz, $CDCl_3$, 300 K): δ [ppm] = 12.15 (s, 1H, OH), 7.78 – 7.68 (m, 2H, H2’, H6’), 7.03 (dd, ^3J = 7.9 Hz, ^4J = 1.5 Hz, 1H, H6), 7.00 – 6.95 (m, 2H, H3’, H5’), 6.92 (dd, ^3J = 7.9 Hz, ^3J = 1.5 Hz, 1H, H6), 6.72 (*virt.* t, $^3J \approx {}^3J \approx$ 7.9 Hz, 1H, H5, 1H, H5), 3.89 (s, 3H), 3.79 (bs, 2H, NH_2).

^{13}C-NMR (101 MHz, $CDCl_3$, 300 K): δ [ppm] = 200.6 (CO), 163.0 (C4’), 150.9 (C2), 136.6 (C1), 132.0 (C2’, C6’), 130.8 (C1’), 122.7 (C6), 119.9 (C4), 118.9 (C3), 118.5 (C5), 113.7 (C3’, C5’), 55.7 (OCH_3).

HRMS (ESI) *m/z* $[M+H]^+$ calculated for $[C_{14}H_{14}NO_3]^+$: 244.0968 found: 244.0962.

IR (film) $\tilde{v}$max/cm^{-1} = 3419 (m, OH), 3560 (m, NH), 3344 (m, NH), 3057 (m, CH_{arom}), 1620 (s, C=O), 1592 (m, $C=C_{arom}$), 1592 (s, NH), 1451 (m, $C=C_{arom}$).

2-Amino-6-(4-methoxybenzoyl)phenyl (1*SR*,5*RS*,7*RS*)-1,5,7-trimethyl-2-oxo-3-azabicyclo[3.3.1]nonane-7-carboxylate (*rac*-SI-11)

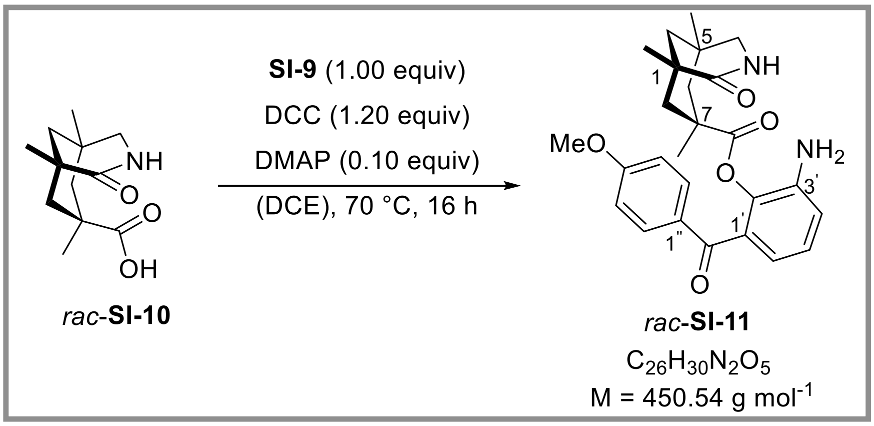

According to a modified procedure,[2] *N*,*N'*-dicyclohexylmethanediimine (DCC) (305 mg, 1.48 mmol, 1.20 equiv.) and *N*,*N*-dimethylpyridin-4-amine (DMAP) (15.1 mg, 123 µmol, 0.10 equiv.) were added to a mixture of *rac*-(1*SR*,5*RS*,7*RS*)-1,5,7-trimethyl-2-oxo-3-azabicyclo[3.3.1]nonane-7-carboxylic acid (**SI-10**)[8] (278 mg, 1.23 mmol, 1.00 equiv.) and (3-amino-2-hydroxyphenyl)(4-methoxyphenyl)methanone (**SI-5**) (300 mg, 1.23 mmol, 1.00 equiv.) in DCE (8.3 mL). The resulting solution was stirred for 16 hours at 70 °C. Afterwards, the reaction was allowed to cool to room temperature, and the formed precipitate was removed by filtration. The solvent was removed under reduced pressure, and the residual crude product was subjected to FCC (SiO_2, Hex:EtOAc = 3:1 → 1:1→ 1:2) to yield the desired 2-amino-6-(4-methoxybenzoyl)phenyl (1*SR*,5*RS*,7*RS*)-1,5,7-trimethyl-2-oxo-3-azabicyclo[3.3.1]nonane-7-carboxylate (*rac*-**SI-11**) (261 mg, 579 µmol, 47%) as a light-yellow foam.

TLC (Hex:EtOAc = 1:1): R_f = 0.10 [UV] [Ninhydrin].

M.p.: >260 °C.

^{1}H-NMR (400 MHz, $CDCl_3$, 300 K): δ [ppm] = 7.86 – 7.77 (m, 2H, H2”, H6”), 7.01 (*virt.* t, ^3J = 7.7 Hz, 1H), 6.93 – 6.86 (m, 2H, H3”, H5”), 6.83 (dd, ^3J = 7.7 Hz, ^4J = 1.6 Hz, 1H, H4’), 6.57 (dd, ^3J = 7.7 Hz, ^4J = 1.6 Hz, 1H, H6’), 5.79 (bs, 1H, CONH), 4.66 (bs, 2H, NH_2), 3.86 (s, 3H, OCH_3), 3.17 (ddd, ^2J = 11.7 Hz, ^3J = 3.2 Hz, ^4J = 2.0 Hz, 1H, H_a4), 2.98 (dd, ^2J = 11.7, ^4J = 1.8 Hz, 1H, H_b4), 2.70 (*virt.* dt, ^2J = 14.3, ^4J = 2.0 Hz, 1H, H_a8), 2.40 (dt, ^2J = 14.3 Hz, ^4J = 2.2 Hz, 1H, H_a6), 1.73 (*virt.* dt, ^2J = 13.0 Hz, ^4J = 2.2 Hz, 1H, H_a9), 1.25 – 1.20 (m, 1H, H_b9), 1.17 (s, 3H, C1CH_3), 1.13 (d, ^2J = 14.4 Hz, 1H, H_b8), 1.00 – 0.89 (m, 7H, C5CH_3, C7CH_3, H_b6).

^{13}C-NMR (101 MHz, $CDCl_3$, 300 K): δ [ppm] = 193.4 (CO), 176.7 (CONH), 174.7 (COO), 163.9 (C4''), 141.0 (C3'), 135.1 (C1'), 134.4 (C2'), 133.0 (C2'', C6''), 129.9 (C1''), 126.0 (C5'), 118.8 (C4'), 117.7 (C6'), 113.7 (C3'', C5''), 55.6 ($COCH_3$), 52.9 (C4), 46.4 (C6), 45.4 (C8), 44.7 (C9), 42.9 (C7), 38.7 (C1), 31.5 (C7*C*H_3), 30.4 (C5), 28.9 (C5*C*H_3), 24.8 (C1*C*H_3).

HRMS (**ESI**) *m/z* $[M+H]^+$ calculated for $[C_{26}H_{31}N_2O_5]^+$: 451.2227 found: 451.2224.

IR (film): $\tilde{v}max/cm^{-1}$ = 3335 (m, NH), 3226 (m, NH), 2957 (m, CH_{aliph}), 1740 (s, C=O), 1651 (s, NHC=O), 1596 (m, $C{=}C_{arom}$), 1574 (s, NH), 1468 (m, $C{=}C_{arom}$).

(1*SR*,5*SR*,7*RS*)-7-(7-(4-Methoxybenzoyl)benzo[*d*]oxazol-2-yl)-1,5,7-trimethyl-3-azabicyclo[3.3.1]nonan-2-one (*rac*-**2b**)

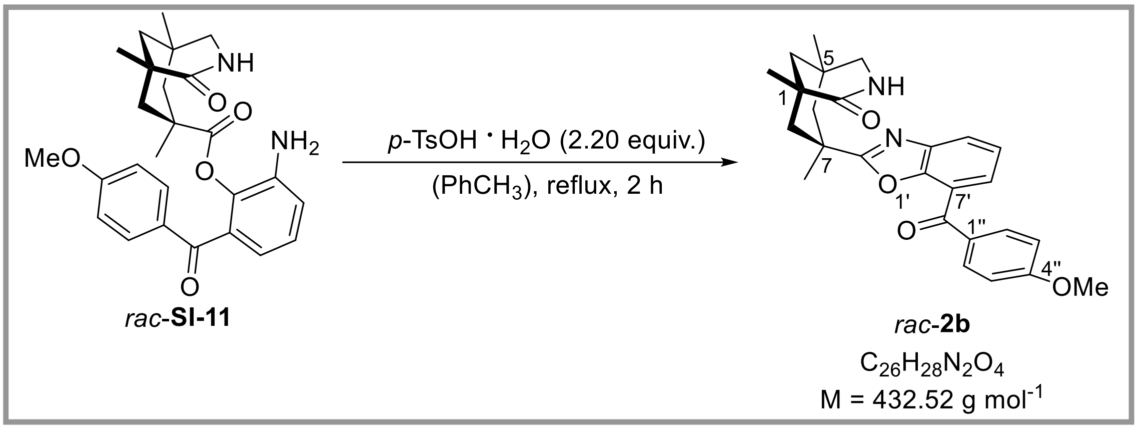

According to a modified procedure,[2] *p*-toluenesulfonic acid monohydrate (223 mg, 1.17 mmol, 2.20 equiv.) was heated to 100 °C in a schlenk flask under vacuum for 20 minutes. Afterwards, the tube was flushed with argon. Toluene (41 mL) and (1*SR*,5*RS*,7*RS*)-1,5,7-trimethyl-2-oxo-3-azabicyclo[3.3.1]nonane-7-carboxylate (*rac*-**SI-11**) (240 mg, 533 µmol, 1.00 equiv.) were added and the resulting suspension was refluxed for two hours. Subsequently, the mixture was allowed to cool to room temperature and sat. bicarb solution (40 mL) was added. The organic layer was separated, and the aqueous layer was extracted with EtOAc (3 × 30 mL). The combined organic layers were washed with brine (15 mL) and dried over Na_2SO_4. After filtration, the solvent was removed under reduced pressure and the residual crude product was subjected to FCC (SiO_2, Hex:EtOAc = 1:1→ 1:2) to yield the desired *rac*-(1*SR*,5*SR*,7*RS*)-7-(7-(4-methoxybenzoyl)benzo[*d*]oxazol-2-yl)-1,5,7-trimethyl-3-azabicyclo[3.3.1]nonan-2-one (*rac*-**2b**) (195 mg, 451 µmol, 85%) as a light-yellow foam.

TLC (Hex:EtOAc = 1:2): R_f = 0.13 [UV] [$KMnO_4$].

M.p.: >260 °C.

^{1}H-NMR (400 MHz, $CDCl_3$, 300 K): δ [ppm] = 7.94 (m, 2H, H2", H6"), 7.83 (dd, ^3J = 7.7 Hz, ^4J = 1.1 Hz, 1H, H4'), 7.63 (dd, ^3J = 7.7 Hz, ^4J = 1.1 Hz, 1H, H6'), 7.39 (*virt.* t, $^3J \approx {}^3J \approx$ 7.7 Hz, 1H, H5'), 7.10 – 7.02 (m, 2H, H3", H5"), 5.61 (bs, 1H, CONH), 3.91 (s, 3H, OCH_3), 3.46 (d, ^2J = 11.2 Hz, 1H, H_a4), 3.01 (d, ^2J = 11.8 Hz, 1H, H_b4), 2.83 (m, 2H, H_a6, H_a8), 1.76 (d, ^3J = 12.8 Hz, 1H, H_a9), 1.42 – 1.33 (m, 3H, H_b6, H_b8, H_b9), 1.31 (s, 3H, $C7CH_3$), 1.22 (s, 3H, $C1CH_3$), 1.08 (s, 3H, $C5CH_3$)

^{13}C-NMR (101 MHz, $CDCl_3$, 300 K): δ [ppm] = 191.5 (CO), 175.9 (CONH), 171.6 (C2'), 163.9 (C4"), 148.3 (C7a'), 142.1 (C3a'), 133.0 (C2", C6"), 130.3 (C1"), 126.7 (C6'), 124.2 (C5'), 123.5 (C4'), 123.0 (C7'), 113.9 (C3", C5"), 55.7 (OCH_3), 53.1 (C4), 46.5 (C6/8), 46.4 (C6/8), 44.8 (C9), 38.4 (C1), 37.7 (C7), 33.5 (C7*C*H_3), 30.8 (C5), 29.3 (C5*C*H_3), 25.1 (C1*C*H_3).

HRMS (**ESI**) *m/z* $[M+H]^+$ calculated for $[C_{26}H_{28}N_2O_4]^+$: 433.2122 found: 433.2122.

IR (film): $\tilde{\nu}$ max/cm^{-1} = 3332 (m, NH), 3070 (m, CH_{arom}), 2961 (m, CH_{aliph}), 2929 (m, CH_{aliph}), 2844 (m, OCH_3), 1645 (s, C=O), 1617 (m, $C{=}C_{arom}$), 1598 (m, $C{=}C_{arom}$), 1489 (m, $C{=}C_{arom}$), 1256 (s, C-O-C).

Chiral HPLC (AD-H, 250 × 4.6 mm, *n*-Hep:*iso*-PrOH = 70:30, 1 mL/min, λ = 210 nm): 6.33 min [(+)-**2b**], 31.6 min [(–)-**2b**].

The two enantiomers of *rac*-**2b** were separated on semi-preparative HPLC (*Daicel* Chiralpak AD, 250 × 20 mm, *n*-Hep:*iso*-PrOH = 70:30, 15 mL/min).

Specific Rotation: $[\alpha]_D^{25}$: −102 [c = 1.0, CH_2Cl_2, (–)-**2b**].

(2-Methoxy-3-nitrophenyl)(4-(trifluoromethyl)phenyl)methanol (SI-12)

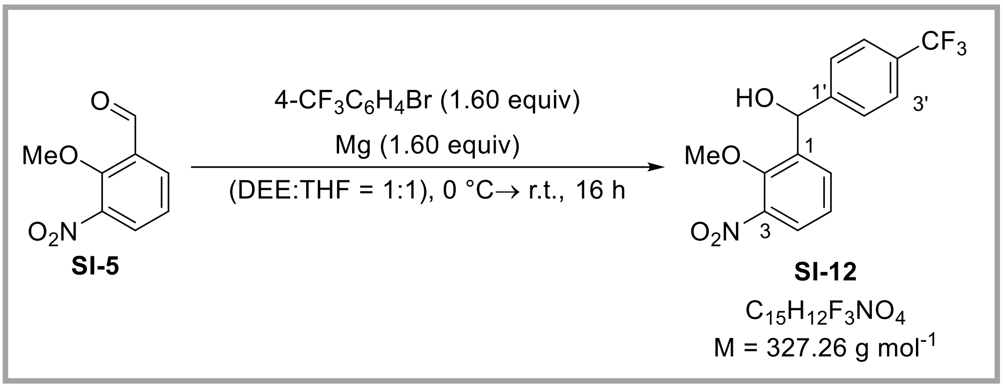

Following a modified procedure,[5] magnesium turnings (429 mg, 17.7 mmol, 1.60 equiv.) and one crystal of iodine were added to a three-neck flask connected to an addition funnel and a findenser©. The mixture was heated upon strong stirring until violet vapor appeared. After allowing the flask to cool to room temperature, 1-bromo-4-(trifluoromethyl)benzene (2.47 mL, 17.7 mmol, 1.60 equiv.) dissolved in Et_2O (10 mL) was added dropwise over 25 minutes, resulting in the mixture to boil. The suspension was stirred until the magnesium was fully consumed and the exothermic reaction stopped. The resulting solution was transferred into a new addition funnel and was added dropwise to a solution of 2-methoxy-3-nitrobenzaldehyde (**SI-5**) (2.00 g, 11.0 mmol, 1.00 equiv.) in Et_2O:THF (1:1, 70 mL) at 0 °C. The resulting dark red solution was stirred for 16 hours at room temperature. Afterwards, the reaction was quenched by addition of sat. NH_4Cl solution (100 mL) and water (50 mL) and the aqueous layer was extracted with EtOAc (3 × 100 mL). The combined organic layers were washed with brine (100 mL) and dried over Na_2SO_4. The solvent was removed under reduced pressure, and the residual crude product was subjected to FCC (SiO_2, Hex:EtOAc = 8:1 → 4:1) to yield the desired (2-methoxy-3-nitrophenyl)(4-(trifluoromethyl)phenyl)methanol (**SI-12**) (2.87 g, 8.76 mmol, 79%) as a yellow oil.

TLC (Hex:EtOAc = 8:1): R_f = 0.36 [UV] [$KMnO_4$].

^{1}H-NMR (400 MHz, $CDCl_3$, 300 K): δ [ppm] = 7.81 (dd, ^3J = 8.1 Hz, ^3J = 1.8 Hz, 1H, H4), 7.69 (dd, ^3J = 7.7 Hz, ^3J = 1.8 Hz, 1H, H6), 7.64 – 7.60 (m, 2H, H3', H5'), 7.55 – 7.48 (m, 2H, H2', H6'), 7.25 (*virt.* t, $^3J \approx {}^3J \approx$ 8.0 Hz, 1H, H5), 6.22 (s, 1H, CH), 3.74 (s, 3H, OCH_3), 2.62 (bs, 1H, OH).

^{13}C-NMR (101 MHz, $CDCl_3$, 300 K): δ [ppm] = 151.2 (C2), 146.7 (C1'), 143.5 (C3), 139.4 (C1), 132.7 (C6), 130.4 (q, $^2J_{C\text{-}F}$ = 32.4 Hz, C4'), 126.9 (C2', C6'), 125.8 (q, $^3J_{C\text{-}F}$ = 3.8 Hz,

C3’, C5’), 125.6 (C4), 124.3 (C5), 124.1 (q, $^{1}J_{C\text{-}F}$ = 272.1 Hz, CF_3), 70.6 (HCOH), 62.7 (OCH_3).

^{19}F-NMR (376MHz, $CDCl_3$, 300 K): δ[ppm] = −62.4.

HRMS (**ESI**) *m/z* [M-OH]$^+$ calculated for $[C_{15}H_{11}F_3NO_3]^+$: 310.0686, found: 310.0684.

IR (film): $\tilde{v}$max/cm^{-1} = 3412 (w, OH), 2952 (m, OCH_3), 1526 (s, $C_{arom}NO_2$), 1322 (s, $C_{arom}NO_2$), 1066 (s, CF_3).

(2-Methoxy-3-nitrophenyl)(4-(trifluoromethyl)phenyl)methanone (SI-13)

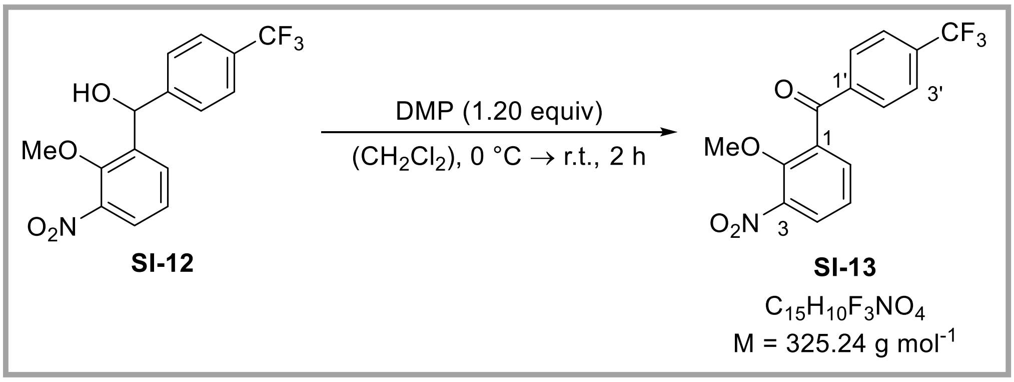

Following a modified procedure,[6] DMP (4.08 g, 9.62 mmol, 1.20 equiv.) was added to a solution of (2-methoxy-3-nitrophenyl)(4-(trifluoromethyl)phenyl)methanol (**SI-12**) (2.62 g, 8.02 mmol, 1.00 equiv.) in CH_2Cl_2 (65 mL) in one portion at 0 °C. The ice bath was removed, and the resulting red solution was stirred for two hours at room temperature. Afterwards, the reaction was quenched by addition of sat. $Na_2S_2O_3$ solution (20 mL) and sat. bicarb (20 mL) and the aqueous layer was extracted with CH_2Cl_2 (3 × 50 mL). The combined organic layers were washed with brine (50 mL) and dried over Na_2SO_4. The solvent was removed under reduced pressure and the residual crude product was subjected to FCC (SiO_2, Hex:EtOAc = 10:1 → 6:1) to yield the desired (2-methoxy-3-nitrophenyl)(4-(trifluoromethyl)phenyl)methanone (**SI-13**) (2.42 g, 7.44 mmol, 93%) as an orange-beige solid.

TLC (Hex:EtOAc = 8:1): R_f = 0.67 [UV] [$KMnO_4$].

M.p.: 60 °C.

^{1}H-NMR (400 MHz, $CDCl_3$, 300 K): δ [ppm] = 8.01 (dd, ^3J = 8.2 Hz, ^4J = 1.7 Hz, 1H, H4), 7.96 – 7.88 (m, 2H, H2’, H6’), 7.79 – 7.71 (m, 2H, H3’, H5’), 7.64 (dd, ^3J = 7.7 Hz, ^4J = 1.7 Hz, 1H, H6), 7.36 (*virt.* t, $^3J \approx {}^3J \approx$ 7.7 Hz, 1H, H5), 3.75 (s, 3H, OCH_3).

^{13}C-NMR (101 MHz, $CDCl_3$, 300 K): δ [ppm] = 193.4 (CO), 151.8 (C2), 144.3 (C3), 139.3 (C1’), 135.3 (s, C1), 135.3 (q, $^{2}J_{C\text{-}F}$ = 32.7 Hz, C4’), 134.0 (C6), 130.2 (C2’, C6’), 127.9 (C4), 126.3 (q, $^{3}J_{C\text{-}F}$ = 3.8 Hz, C3’, C5’), 125.4 (q, $^{1}J_{C\text{-}F}$ = 270.1 Hz, CF_3), 124.4 (C5), 64.2 (OCH_3).

^{19}**F-NMR** (376MHz, $CDCl_3$, 300 K): δ[ppm] = −63.2.

IR (film): $\tilde{v}$max/cm^{-1} = 3077 (m, CH_{arom}), 2964 (m, CH_{aliph}), 1678 (s, C=O), 1601 (m, $C{=}C_{arom}$), 1530 (s, $C_{arom}NO_2$), 1455 (m, $C{=}C_{arom}$), 1066 (s, CF_3).

(2-Hydroxy-3-nitrophenyl)(4-(trifluoromethyl)phenyl)methanone (SI-14)

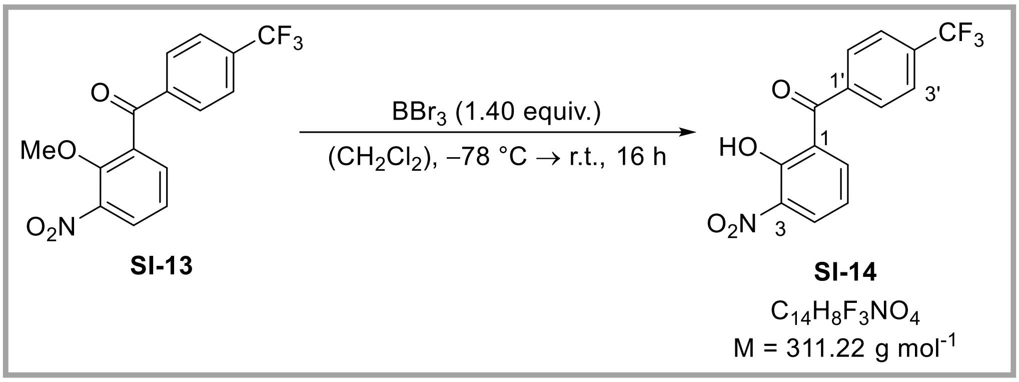

Following a modified procedure,[6] BBr_3 (3.40 mL, 2.56 g, 10.2 mmol, 1.40 equiv.) was added dropwise to a solution of benzophenone (2-methoxy-3-nitrophenyl)(4-(trifluoromethyl)phenyl)methanone (**SI-13**) (2.38 g, 7.30 mmol, 1.00 equiv.) in CH_2Cl_2 (31 mL) at −78 °C. The mixture was allowed to warm to room temperature and stirred for 16 hours. Afterwards, the reaction was quenched by careful addition of water (60 mL), and the aqueous layer was extracted with CH_2Cl_2 (3 × 60 mL). The combined organic layers were washed with brine (60 mL) and dried over Na_2SO_4. The solvent was removed under reduced pressure and the residual crude product was subjected to FCC (SiO_2, Hex:EtOAc = 10:1) to yield the desired (2-hydroxy-3-nitrophenyl)(4-(trifluoromethyl)phenyl)methanone (**SI-14**) (1.74 g, 5.59 mmol, 77%) as a brown-red solid.

TLC (Hex:EtOAc = 8:1): R_f = 0.24 [UV] [$KMnO_4$].

M.p.: 82 °C.

^{1}**H-NMR** (400 MHz, $CDCl_3$, 300 K): δ [ppm] = 11.42 (s, 1H, OH), 8.31 (dd, ^3J = 8.4 Hz, ^4J = 1.7 Hz, 1H, H4), 7.94 – 7.85 (m, 2H, H2', H6'), 7.80 (dd, ^3J = 7.6 Hz, ^4J = 1.7 Hz, 1H, H6), 7.78 – 7.74 (m, 2H, H3', H5'), 7.13 (dd, ^3J = 8.4, ^3J = 7.6 Hz, 1H).

^{13}**C-NMR** (101 MHz, $CDCl_3$, 300 K): δ [ppm] = 194.6 (CO), 154.2 (C2), 139.9 (C1'), 138.1 (C6), 135.5 (C3), 134.9 (q, $^2J_{C\text{-}F}$ = 33.0 Hz, C4'), 129.9 (C2', C6'), 129.4 (C4), 128.0 (C1), 125.9 (q, $^3J_{C\text{-}F}$ = 3.8 Hz, C3', C5'), 123.7 (q, $^1J_{C\text{-}F}$ = 270.0 Hz, CF_3), 119.8 (C5).

^{19}**F-NMR** (376MHz, $CDCl_3$, 300 K): δ[ppm] = −63.2.

IR (film): $\tilde{v}$max/cm^{-1} = 3224 (m, OH), 3097 (w, CH_{arom}), 1667 (s, C=O), 1607 (s, , $C{=}C_{arom}$), 1539 (s, $C_{arom}NO_2$), 1443 (m, $C{=}C_{arom}$), 1065 (s, CF_3).

(3-Amino-2-hydroxyphenyl)(4-(trifluoromethyl)phenyl)methanone (SI-15)

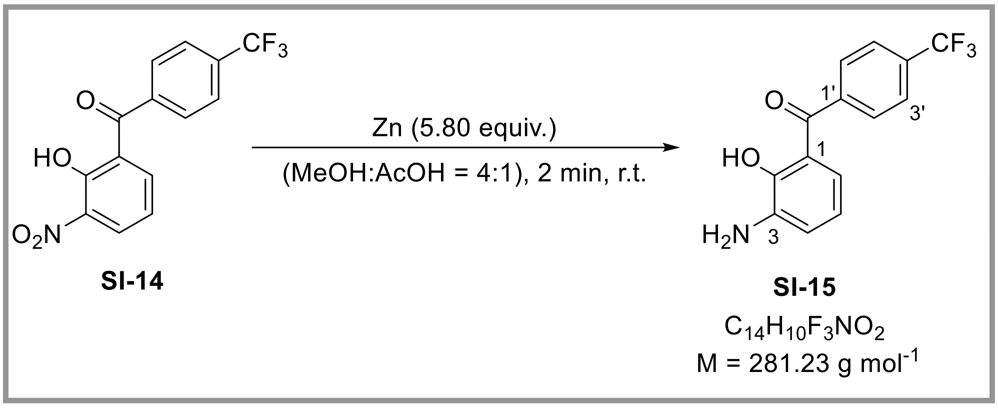

According to a modified procedure,[2] zinc dust (1.83 g, 28.0 mmol, 5.80 equiv.) was added in one portion to a solution of (2-methoxy-3-nitrophenyl)(4-(trifluoromethyl)phenyl)methanone (**SI-14**) (1.50 g, 4.82 mmol, 1.00 equiv.) in MeOH:AcOH (110 mL, 4:1) at room temperature. The suspension was stirred for 2 minutes, and the excess zinc dust was filtered through cotton. Water (150 mL) was added to the filtrate, and the aqueous layer was extracted with Et_2O (4 × 100 mL). The combined organic layers were washed with sat. bicarb (150 mL), brine (40 mL) and dried over Na_2SO_4. The solvent was removed under reduced pressure, and the residual crude product was subjected to FCC (SiO_2, Hex:EtOAc = 8:1) to yield the desired (3-amino-2-hydroxyphenyl)(4-(trifluoromethyl)phenyl)methanone (**SI-15**) (681 mg, 2.42 mmol, 50%) as a red oil.

TLC (Hex:EtOAc = 8:1): R_f = 0.21 [UV] [Ninhydrin].

^{1}H-NMR (400 MHz, $CDCl_3$, 300 K): δ [ppm] = 12.04 (s, 1H, OH), 7.81 – 7.72 (m, 4H, H2’, H3’, H5’, H6’), 6.96 (dd, ^3J = 7.7 Hz, ^4J = 1.4 Hz, 1H, H4), 6.88 (dd, ^3J = 8.1 Hz, ^4J = 1.4 Hz, 1H, H6), 6.73 (*virt.* t, $^3J \approx {}^3J \approx$ 7.9 Hz, 1H, H5), 3.93 (bs, 2H, NH_2).

^{13}C-NMR (101 MHz, $CDCl_3$, 300 K): δ [ppm] = 201.0 (CO), 151.2 (C2), 141.5 (C1’), 136.9 (C1), 133.4 (q, $^2J_{C\text{-}F}$ = 32.8 Hz, C4’), 129.4 (C2’, C6’), 125.4 (q, $^3J_{C\text{-}F}$ = 3.8 Hz C3’, C5’), 123.8 (q, $^1J_{C\text{-}F}$ = 272.5 Hz, CF_3), 122.5 (C6), 120.6 (C4), 119.0 (C5), 118.3 (C3).

^{19}F-NMR (376MHz, $CDCl_3$, 300 K): δ[ppm] = −63.0.

HRMS (ESI) *m/z* $[M+H]^+$ calculated for $[C_{14}H_{11}F_3NO_2]^+$: 282.0736 found: 282.0734.

IR (film) $\tilde{v}$max/cm^{-1} = 3484 (m, NH), 3382 (m, NH), 1615 (s, C=O), 1599 (m, $C{=}C_{arom}$), 1454 (m, $C{=}C_{arom}$), 1063 (s, CF_3).

2-Amino-6-(4-(trifluoromethyl)benzoyl)phenyl (1*SR*,5*RS*,7*RS*)-1,5,7-trimethyl-2-oxo-3-azabicyclo[3.3.1]nonane-7-carboxylate (*rac*-SI-16)

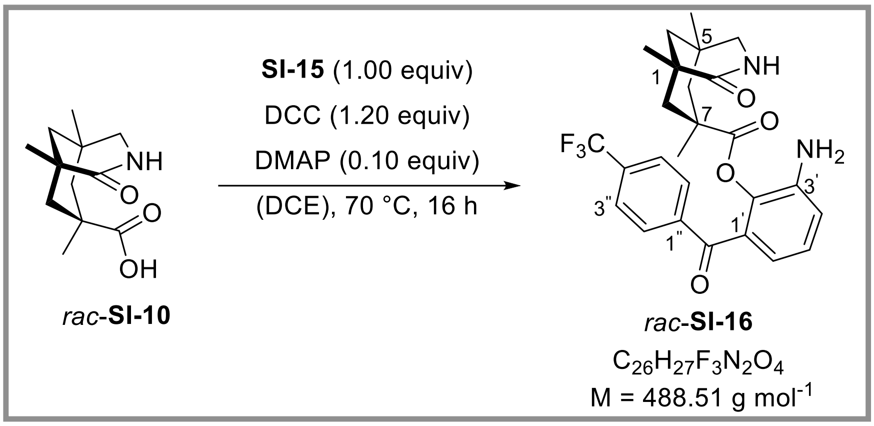

According to a modified procedure,[2] DCC (121 mg, 586 µmol, 1.20 equiv.) and DMAP (5.97 mg, 48.8 µmol, 0.10 equiv.) were added to a mixture of *rac*-(1*SR*,5*RS*,7*RS*)-1,5,7-trimethyl-2-oxo-3-azabicyclo[3.3.1]nonane-7-carboxylic acid (**SI-10**)[8] (110 mg, 488 µmol, 1.00 equiv.) and (3-amino-2-hydroxyphenyl)(4-(trifluoromethyl)phenyl)methanone (**SI-15**) (137 mg, 488 µmol, 1.00 equiv.) in DCE (3.3 mL). The resulting solution was stirred for 16 hours at 70 °C. Afterwards, the reaction was allowed to cool to room temperature, and the formed precipitate was removed by filtration. The solvent was removed under reduced pressure, and the residual crude product was subjected to FCC (SiO_2, Hex:EtOAc = 3:1 → 2:1→ 1:1) to yield the desired 2-amino-6-(4-(trifluoromethyl)benzoyl)phenyl (1*SR*,5*RS*,7*RS*)-1,5,7-trimethyl-2-oxo-3-azabicyclo[3.3.1]nonane-7-carboxylate (*rac*-**SI-16**) (139 mg, 285 µmol, 58%) as a light-yellow foam.

TLC (Hex:EtOAc = 1:1): R_f = 0.28 [UV] [Ninhydrin].

M.p.: >260 °C.

^{1}H-NMR (400 MHz, $CDCl_3$, 300 K): δ [ppm] = 8.00 – 7.91 (m, 2H, H2", H6"), 7.77 – 7.65 (m, 2H, H3", H5"), 7.01 (*virt.* t, ^3J = 7.8 Hz, 1H, H5'), 6.90 (dd, ^3J = 7.8 Hz, ^4J = 1.6 Hz, 1H, H4'), 6.54 (dd, ^3J = 7.8 Hz, ^4J = 1.6 Hz, 1H, H6'), 5.96 (bs, 1H, NH), 4.84 (bs, 2H, NH_2), 3.12 (*virt.* dt, ^2J = 11.7 Hz, ^4J = 2.5 Hz, 1H, H_a4), 2.97 (d, ^2J = 11.9 Hz, 1H, H_b4), 2.73 (*virt.* dt, ^2J = 14.4 Hz, 2.0 Hz, 1H, H_a8), 2.38 (*virt.* dt, ^2J = 14.3 Hz, 2.2 Hz, 1H, H_a6), 1.72 (*virt.* dt, ^2J = 13.0 Hz, 2.2 Hz, 1H, H_a9), 1.27 – 1.15 (m, 5H, H_b8 H_b9, C1CH_3) 1.03 (s, 3H, C7CH_3), 0.96 (dd, ^2J = 14.3 Hz, 1.7 Hz, 1H, H_b6), 0.91 (s, 3H, C5CH_3).

^{13}C-NMR (101 MHz, $CDCl_3$, 300 K): δ [ppm] = 193.5 (CO), 176.8 (CONH), 174.7 (COO), 140.6 (C3'), 139.7 (C1"), 135.2 (C2'), 134.4 (q, $^2J_{C\text{-}F}$ = 32.5 Hz, C4"), 133.1 (C1'), 130.8 (C2', C6'), 125.9 (C5'), 125.4 (q, $^3J_{C\text{-}F}$ = 32.5 Hz, C3", C5"), 123.8 ($^1J_{C\text{-}F}$ = 272.7 Hz, CF_3), 120.0

(C4’), 118.1 (C6’), 52.9 (C4), 46.3 (C6), 45.3 (C8), 44.5 (C9), 42.9 (C7), 38.6 (C1), 31.5 (C7*C*H_3), 30.4 (C5), 28.8 (C5*C*H_3), 24.8 (C1*C*H_3).

^{19}F-NMR (376MHz, $CDCl_3$, 300 K): δ[ppm] = −63.1.

HRMS (**ESI**) *m/z* $[M+H]^+$ calculated for $[C_{26}H_{28}F_3N_2O_4]^+$: 489.1996 found: 489.1993.

IR (film): $\tilde{v}$ max/cm^{-1} = 3338 (m, NH), 2930 (m, CH_{aliph}), 1740 (s, C=O), 1651 (s, NHC=O), 1471 (m, $C{=}C_{arom}$), 1064 (s, CF_3).

(1*SR*,5*SR*,7*RS*)-1,5,7-trimethyl-7-(7-(4-(trifluoromethyl)benzoyl)benzo[*d*]oxazol-2-yl)-3-azabicyclo[3.3.1]nonan-2-one (*rac*-**2c**)

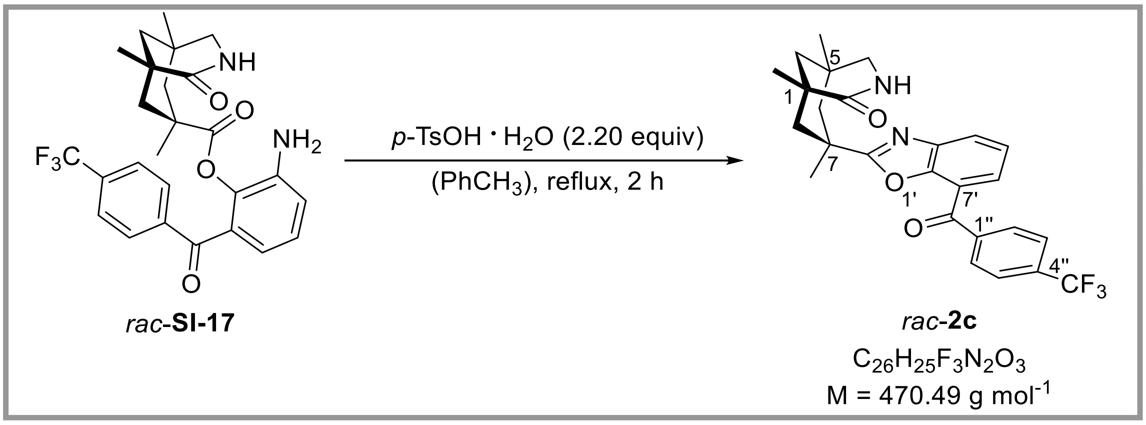

According to a modified procedure,[2] *p*-toluenesulfonic acid monohydrate (115 mg, 602 µmol, 2.20 equiv.) was heated to 100 °C in a schlenk flask under vacuum for 20 minutesutes. Afterwards, the tube was flushed with argon. Toluene (20 mL) and 2-amino-6-(4-(trifluoromethyl)benzoyl)phenyl (1*SR*,5*RS*,7*RS*)-1,5,7-trimethyl-2-oxo-3-azabicyclo[3.3.1]nonane-7-carboxylate (*rac*-**SI-16**) (133 mg, 272 µmol, 1.00 equiv.) were added and the resulting suspension was refluxed for two hours. Subsequently, the mixture was allowed to cool to room temperature and sat. bicarb solution (15 mL) was added. The organic layer was separated, and the aqueous layer was extracted with EtOAc (3 × 20 mL). The combined organic layers were washed with brine (20 mL) and dried over Na_2SO_4. After filtration, the solvent was removed under reduced pressure and the residual crude product was subjected to FCC (SiO_2, Hex:EtOAc = 1:1 → 1:2) to yield the desired *rac*-(1*SR*,5*SR*,7*RS*)-1,5,7-trimethyl-7-(7-(4-(trifluoromethyl)benzoyl)benzo[*d*]oxazol-2-yl)-3-azabicyclo[3.3.1]nonan-2-one (*rac*-**2c**) (90.0 mg, 191µmol, 70%) as a light-yellow foam.

TLC (Hex:EtOAc = 1:2): R_f = 0.19 [UV] [$KMnO_4$].

M.p.: >260 °C.

^{1}H-NMR (400 MHz, $CDCl_3$, 300 K): *δ* [ppm] = 8.03 – 7.98 (m, 2H, H2", H6"), 7.90–7.86 (m, 3H, H3", H4', H5"), 7.78 (dd, ^3J = 7.8 Hz, ^4J = 1.2 Hz, 1H, H6'), 7.44 (*virt*.t, $^3J \approx {}^3J \approx$ 7.8 Hz, 1H, H5'), 5.35 (s, 1H, CONH), 3.44 (d, ^2J = 11.6 Hz, 1H, H_a4), 3.01 (d, ^2J = 11.6 Hz, 1H, H_b4), 2.81 (d, ^2J = 14.4 Hz, 1H, H_a8), 2.53 (d, ^2J = 14.2 Hz, 1H, H_a6), 1.76 (d, ^2J = 12.9 Hz, 1H, H_a9), 1.40 – 1.26 (m, 3H, H_b6, H_b8, H_b9), 1.23 (s, 3H, $C7CH_3$), 1.19 (s, 3H, $C1CH_3$), 1.08 (s, 3H, $C5CH_3$)

^{13}C-NMR (101 MHz, $CDCl_3$, 300 K): *δ* [ppm] = 191.8 (CO), 175.6 (CONH), 171.9 (C2'), 148.6 (C7a'), 142.3 (C3a'), 141.0 (C1"), 134.0 (q, $^2J_{C\text{-}F}$ = 32.5 Hz, C4"), 130.4 (C2", C6"), 126.9 (C6'), 125.7 (q, $^3J_{C\text{-}F}$ = 3.8 Hz, C3", C5"), 124.8 (C4'), 124.6 (C5'), 123.9 (q, $^1J_{C\text{-}F}$ = 271.3 Hz, CF_3), 121.6 (C7'), 53.0 (C4), 46.5 (C8), 46.1 (C6), 44.8 (C9), 38.3 (C1), 37.7 (C7), 33.5 ($C7CH_3$), 30.8 (C5), 29.2 ($C5CH_3$), 25.0 ($C1CH_3$).

^{19}F-NMR (376MHz, $CDCl_3$, 300 K): δ[ppm] = −63.0.

HRMS (**ESI**) *m/z* $[M+H]^+$ calculated for $[C_{26}H_{26}F_3N_2O_3]^+$: 471.1890 found: 471.1890.

IR (film): $\tilde{v}$max/cm^{-1} = 3263 (m, NH), 2963 (m, CH_{aliph}), 2930 (m, CH_{aliph}), 1660 (s, C=O), 1616 (m, $C{=}C_{arom}$), 1064 (s, CF_3).

Chiral HPLC (AD-H, 250 × 4.6 mm, *n*-Hep:*iso*-PrOH = 70:30, 1mL/min, λ = 210 nm): 4.78 min [(+)-**2c**]., 25.7 min [(–)-**2c**].

The two enantiomers of *rac*-**2c** were separated on semi-preparative HPLC (*Daicel* Chiralpak AD, 250 × 20 mm, *n*-Hep:*iso*-PrOH = 70:30).

Specific Rotation: $[a]_D^{25}$: −104 (c = 1.0, CH_2Cl_2, (–)-**2b**).

5. Characterization of (–)-2b

Determination of the absolute configuration of catalyst 2b

To determine the absolute configuration of the newly synthesized catalyst **2b**, substrate **3a** was subjected to reactions under otherwise identical conditions using the enantiomerically pure reference catalysts (+)-**2a** and (–)-**2a** with a loading of 10 mol%. Irradiation in the presence of (+)-**2a** led to the predominant formation of *ent*-**3a** (Figure S4), whereas the use of (–)-**2a** resulted in the preferential formation of substrate enantiomer **3a** (Figure S5). When the reaction was conducted with the newly synthesized catalyst (–)-**2b** in enantiomerically pure form, the accumulation of substrate enantiomer **3a** was observed, indicating that the catalyst possesses the absolute configuration (–)-**2b** (Figure S6). This assignment is further supported by the specific optical rotation of the employed catalyst enantiomer, which was determined to be negative ($[\alpha]_D^{25}$ = –102), in agreement with our expectations.

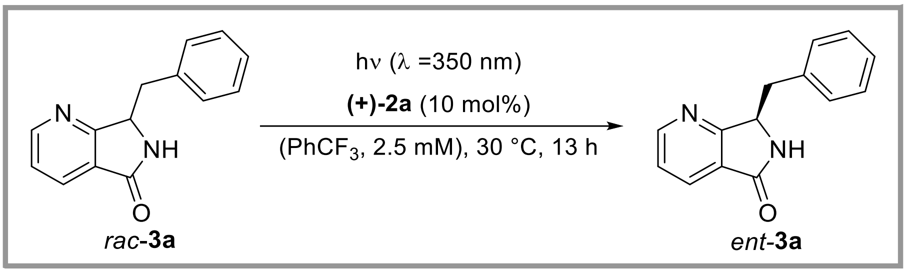

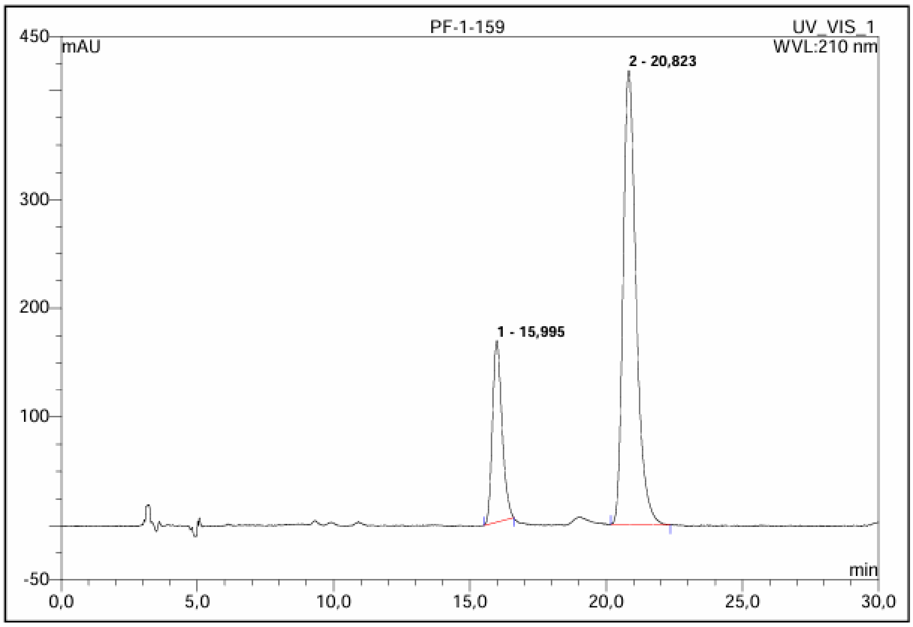

No.	Ret.Time min	Peak Name	Height mAU	Area mAU*min	Rel.Area %	Amount	Type
1	16,00	n.a.	166,659	67,491	22,37	n.a.	BMB*
2	20,82	n.a.	417,327	234,262	77,63	n.a.	BMB*
Total:			583,986	301,752	100,00	0,000	

Figure S4: Chiral HPLC trace of **3a** after reaction with 10 mol% (+)-**2a** for 13 hours at λ = 350 nm.

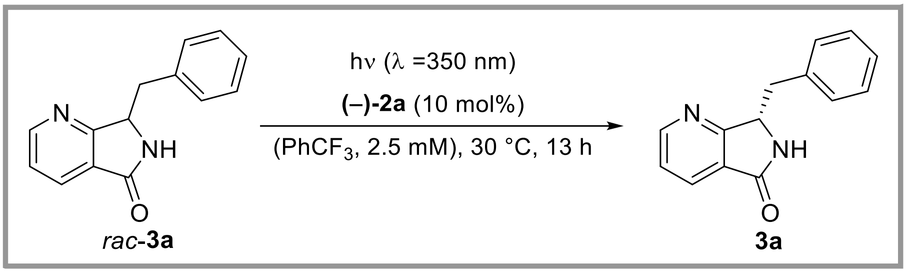

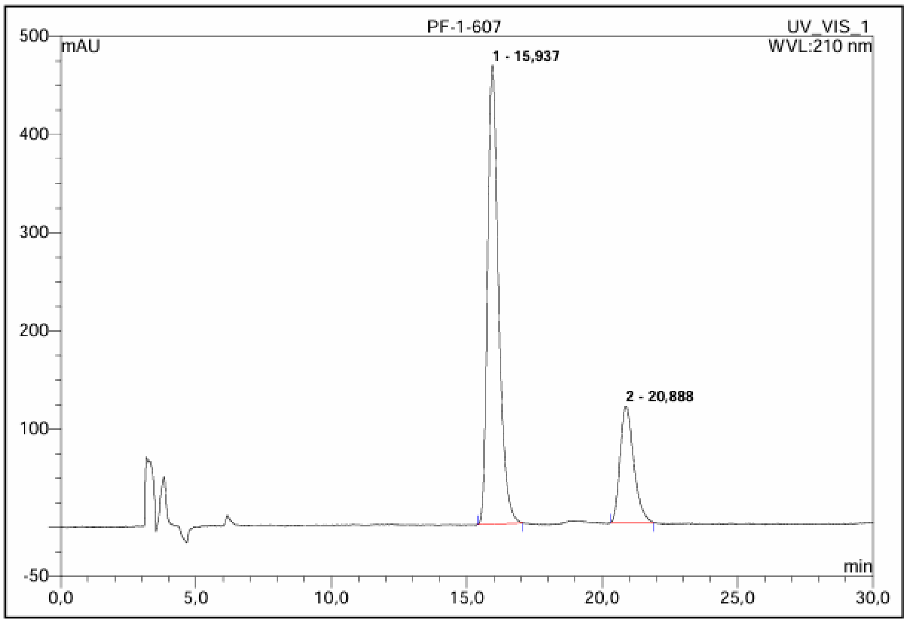

No.	Ret.Time min	Peak Name	Height mAU	Area mAU*min	Rel.Area %	Amount	Type
1	15,94	n.a.	466,915	217,804	76,03	n.a.	BMB
2	20,89	n.a.	118,404	68,669	23,97	n.a.	BMB
Total:			585,319	286,473	100,00	0,000	

Figure S5: Chiral HPLC trace of **3a** after reaction with 10 mol% (**–**)-**2a** for 13 hours at λ = 350 nm.

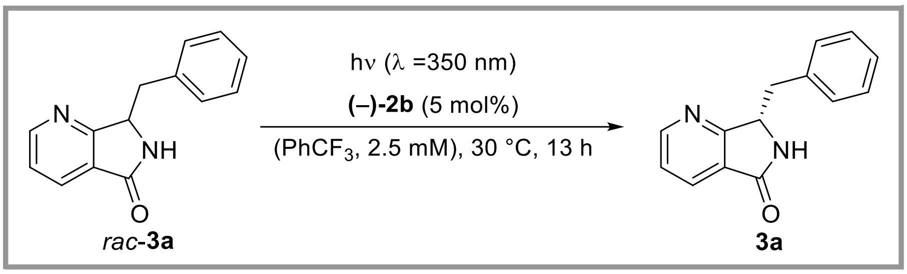

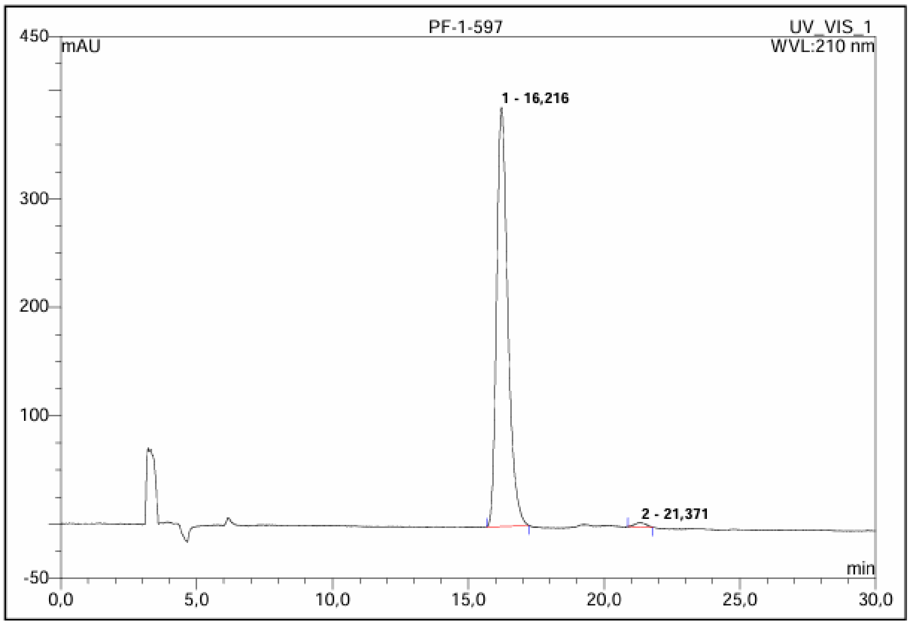

No.	Ret.Time min	Peak Name	Height mAU	Area mAU*min	Rel.Area %	Amount	Type
1	16,22	n.a.	386,675	183,745	99,03	n.a.	BMB
2	21,37	n.a.	3,986	1,792	0,97	n.a.	BMB*
Total:			390,661	185,537	100,00	0,000	

Figure S6: Chiral HPLC trace of **3a** after reaction with 5 mol% **(–)-2b** for 13 hours at λ = 350 nm.

UV-Vis Spectra of (–)-2a

The UV-Vis absorption spectrum of a 1 mM solution of (–)-**2a-c** in CH_2Cl_2 was measured in *Hellma* precision cells made of quartz SUPRASIL® with a light pathway of 10 mm on a Perkin Elmer Lambda 35 UV-Vis spectrometer (Figure **S7**). The extinction coefficient ε at 353 nm for the forbidden first electronic nπ*-transition of (–)-**2b** was calculated to be ε = 221 $M^{-1}cm^{-1}$. The extinction coefficient ε at 367 nm for the forbidden first electronic nπ*-transition of (–)-**2c** was calculated to be ε = 92 $M^{-1}cm^{-1}$.

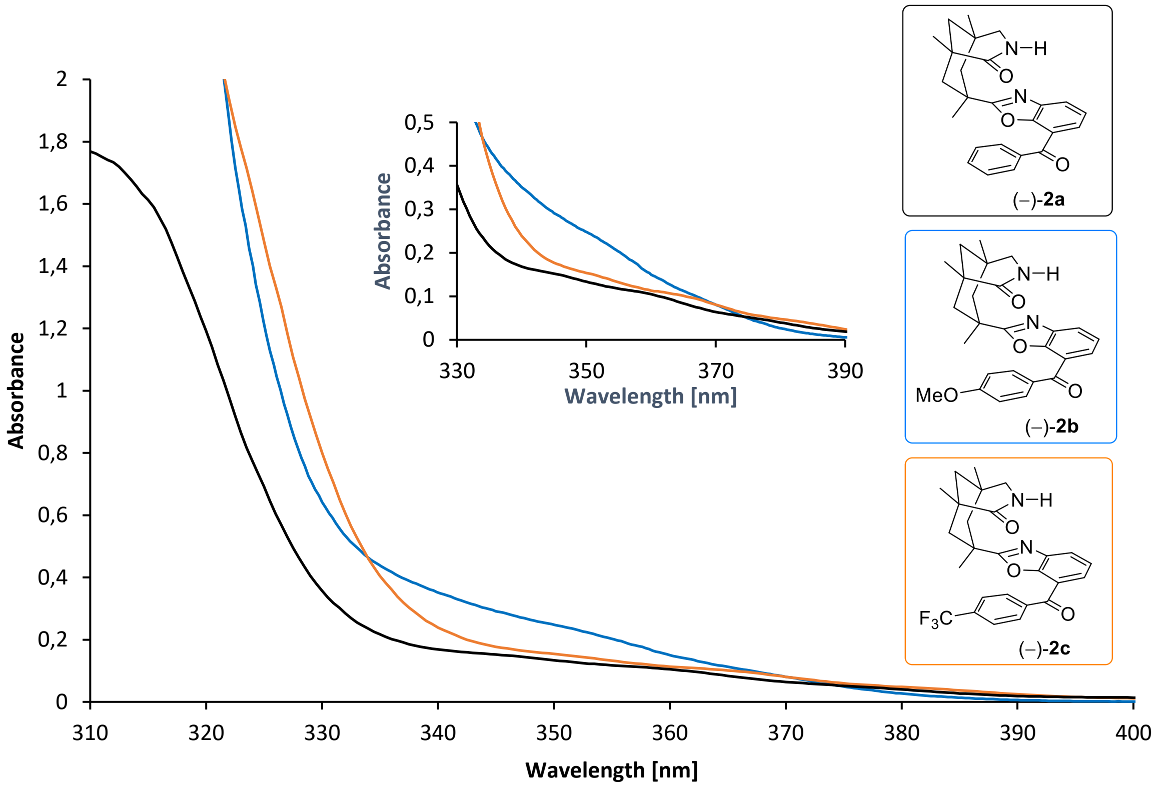

Figure S7: UV-Vis Spectra of (–)-**2a-c** in CH_2Cl_2, (c = 1mM), 10 mm pathway.

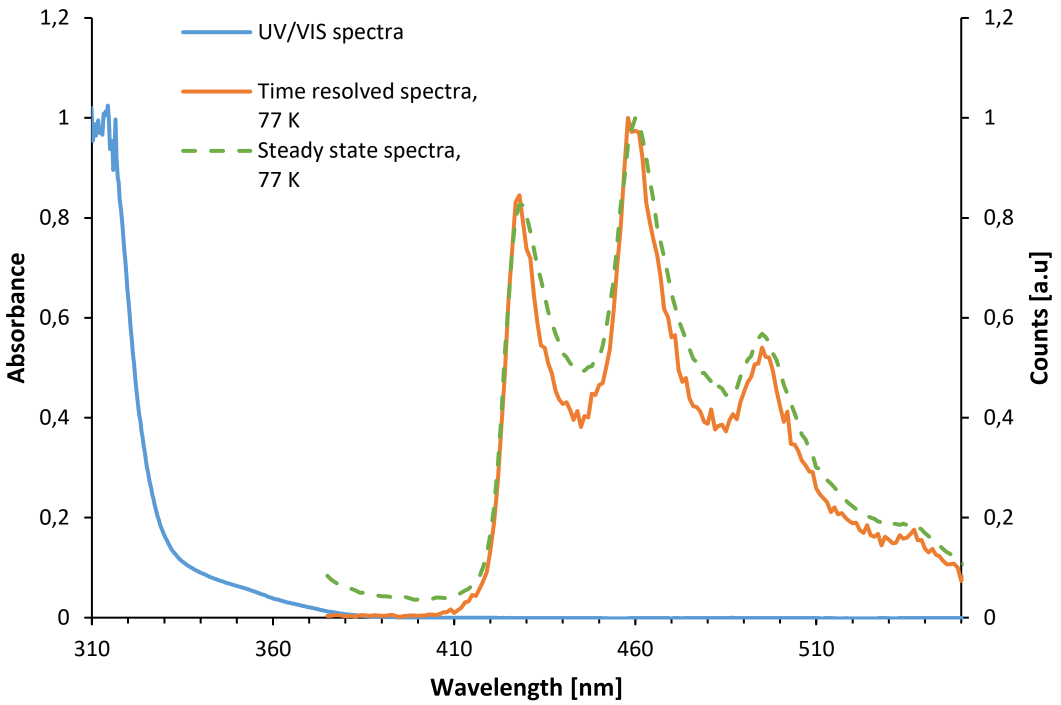

Figure S8: Recorded UV-Vis of (–)-**2b** in $PhCF_3$ (c=100 µM) normalized to A313 nm; steady state spectrum of **1b** in $PhCF_3$ (c=100 µM) at 77 K (dashed lines); time resolved spectrum of (–)-**2b** in $PhCF_3$ (c=100 µM) at 77K after 50 µs delay (solid line). The marked (0,0) transition at λ = 428 nm results in a calculated T_1 energy of E_T = 280 kJ mol^{-1}.

6. Condition Optimization and Kinetic Studies

The reaction conditions were optimized using substrate *rac*-**3a**. The reported yields and *ee*s refer to the isolated product **3a** (Table S1).

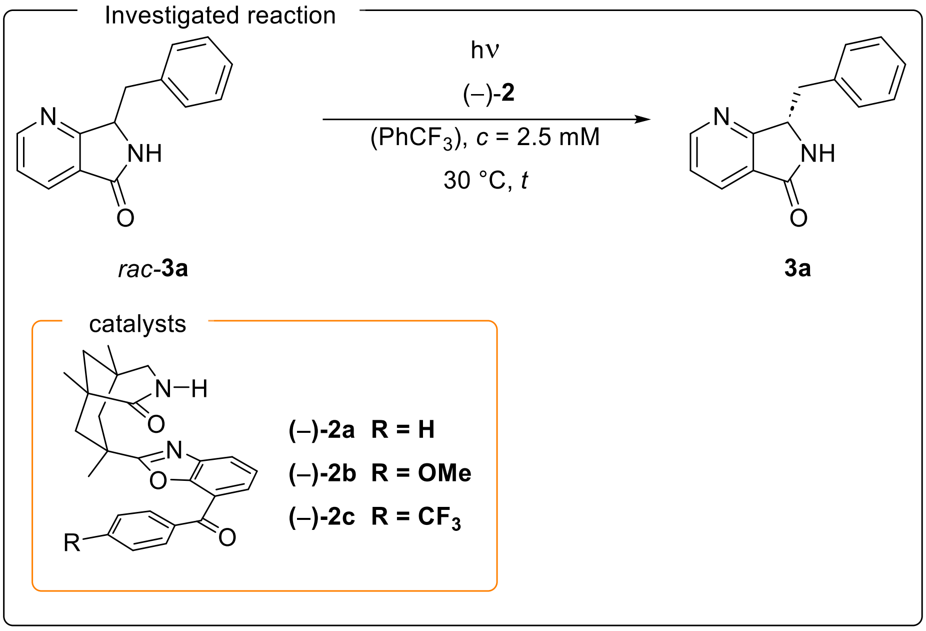

Table S1: Optimization of reaction conditions for the deracemization of *rac*-**3a**. All reactions were performed on a 25.0 µmol scale.

Entry	λ [nm]	Catalyst	[mol%]	*t* [h]	Yield [%]	*ee* [%]
1	366	(–)-**2a**	2.5	8	98	13
2	366	(–)-**2a**	10	13	83	55
3	366	(–)-**2a**	10	18	66	73
4	300	(–)-**2a**	10	13	48	79
5	350	(–)-**2a**	10	13	73	66
6	350	(–)-**2a**	10	18	63	99
7	350	(–)-**2a**	5	13	91	42
8	350	(–)-**2b**	5	13	84	98
9[a]	350	(–)-**2b**	5	13	81	93
10	366	(–)-**2b**	5	13	90	19
11	350	(–)-**2b**	5	10	90	77
12	350	(–)-**2b**	2.5	13	87	40
13	350	(–)-**2c**	5	13	91	36

[a] Reaction was performed at c = 5 mM.

To validate the optimal conditions identified in the initial screening, and to show the differences in performance of catalyst (–)-**2a** and (–)-**2b**, a kinetic study was performed (Figure S9). Samples of the two separate reaction mixture (0.1 mL each) were taken at defined time intervals, using an argon-flushed syringe, followed by solvent evaporation. The resulting crude product was then dissolved in MeOH and analyzed using chiral HPLC. Care was taken to ensure that catalyst (–)-**2a** and (–)-**2b** had a distinct retention time from the enantiomers of substrate *rac*-**3a**, facilitating clear separation and accurate quantification of the enantiomeric ratio without interference from the catalyst.

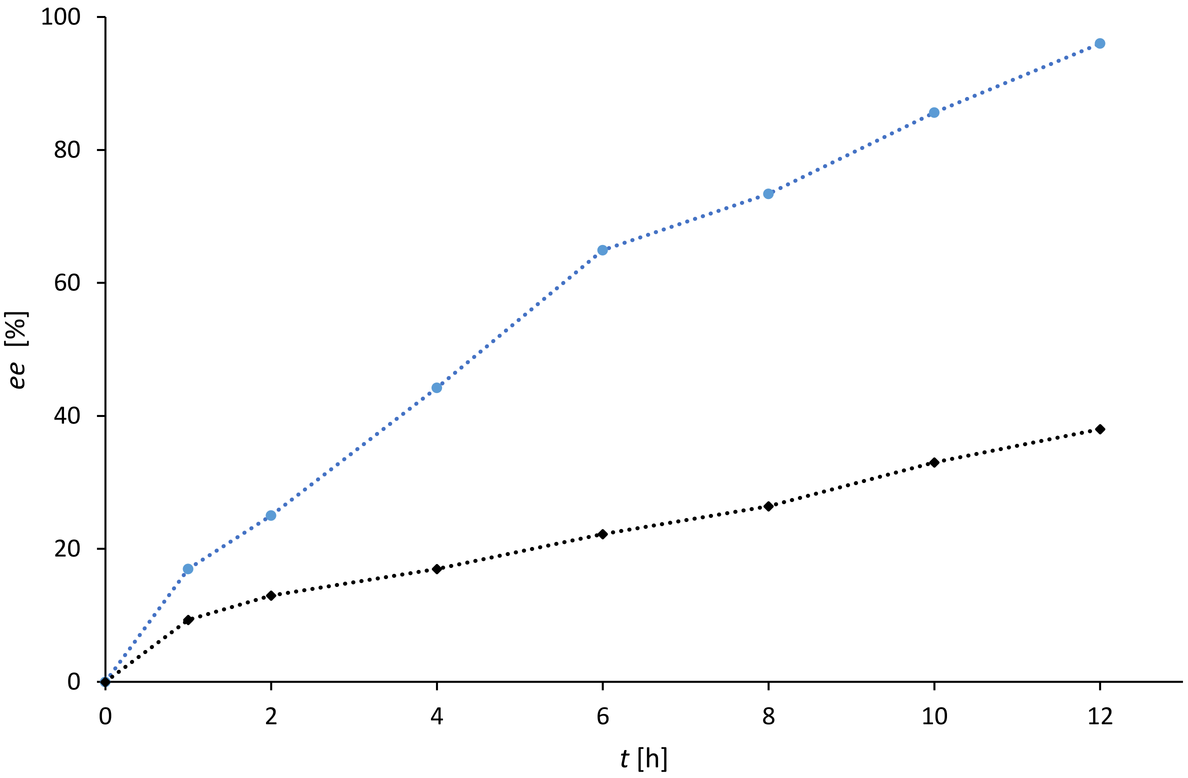

Figure S9: Kinetic profile for the deracemization of *rac*-**3a** using catalyst (–)-**2a** (5 mol%) (black line) and catalyst (–)-**2b** (5 mol%) (blue line) in $PhCF_3$ (2.5 mM) irradiating at 350 nm.

During the studies, it was observed that alkoxy substituted substrates *rac*-**3r** to *rac*-**3v** did not reach the photo stationary state as smoothly compared to the alky substituted ones. Therefore, a screening was conducted with *rac*-**3r** as standard substrate. The screening indicates the photo stationary state is reached after a longer irradiation time or with a higher catalyst loading. However, to achieve high *ee* consistently, substrates *rac*-**3r** to *rac*-**3v** were photochemically deracemized with a higher catalyst loading of (–)-**2b.** The reported yields and *ee*s refer to the isolated product **3r** (Table S2).

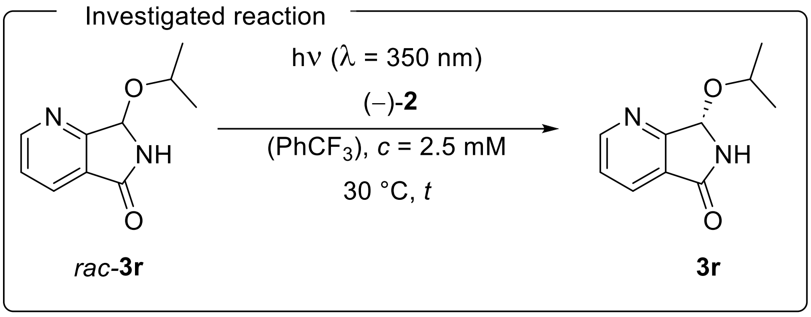

Table S2: Optimization of reaction conditions for the deracemization of *rac*-**3r**. All reactions were performed on a 25.0 µmol scale.

Entry	Catalyst	[mol%]	*t* [h]	Yield [%]	*ee* [%]
1	(–)-**2a**	5	13	94	34
2	(–)-**2b**	5	13	93	56
3	(–)-**2b**	10	13	84	94
4	(–)-**2b**	5	18	73	99

7. Crystallographic Data

SC-XRD structure report for compound **3e**.

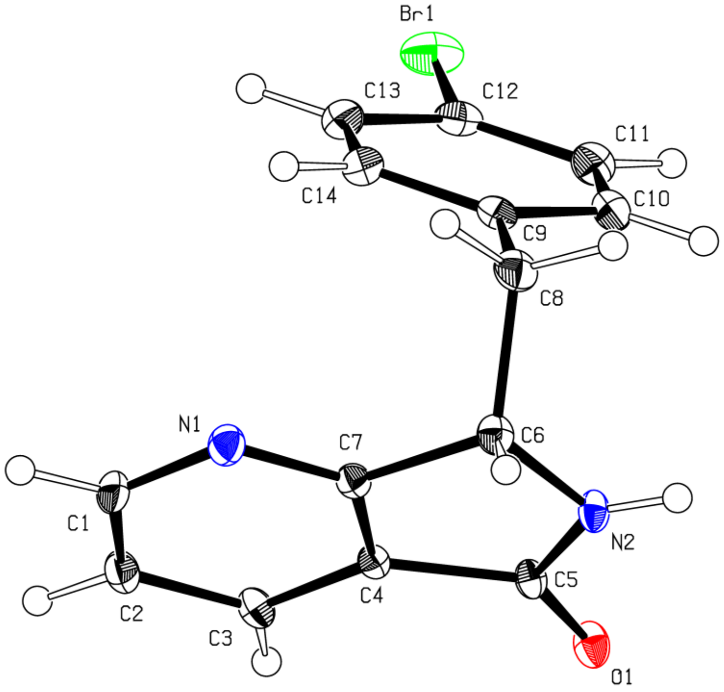

Figure S10: ORTEP representation of the solid-state structure of compound **3e** (C = black, N = blue, O = red and Br = green, Br = green) shown with 50 % probability displacement ellipsoids.

A colourless, needle-shaped crystal of $C_{14}H_{11}BrN_2O$ coated with perfluorinated ether and fixed on top of a Kapton micro sampler was used for X-ray crystallographic analysis. The X-ray intensity data were collected at 100(2) K on a Bruker D8 VENTURE three-angle diffractometer with a TXS rotating anode with MoK_α radiation (λ=0.71073 Å) using APEX4.[9] The diffractometer was equipped with a Helios optic monochromator, a Bruker PHOTON III detector, and an Oxford Cryostreamlow temperature device.

A matrix scan was used to determine the initial lattice parameters. All data were integrated with the Bruker SAINT V8.41 software package using a narrow-frame algorithm and the reflections were corrected for Lorentz and polarisation effects, scan speed, and background.[10] The

integration of the data using a orthorhombic unit cell yielded a total of 63575 reflections within a 2θ range [°] of 4.77 to 72.72 (0.60 Å), of which 6154 were independent. Data were corrected for absorption effects including odd and even ordered spherical harmonics by the multi-scan method (SADABS 2016/2).[11] Space group assignment was based upon systematic absences, E statistics, and successful refinement of the structure.

The structure was solved by dual space methods using SHELXT and refined by full-matrix least-squares methods against F^2 by minimizing $\Sigma w(F_o^2 - F_c^2)^2$ using SHELXL in conjunction with SHELXLE.[12-14] All non-hydrogen atoms were refined with anisotropic displacement parameters. Hydrogen atoms were refined isotropically on calculated positions using a riding model with their U_{iso} values constrained to 1.5 times the U_{eq} of their pivot atoms for terminal sp^3 carbon atoms and a C–H distance of 0.98 Å. Non-methyl hydrogen atoms were refined using a riding model with methylene, aromatic, and other C–H distances of 0.99 Å, 0.95 Å, and 1.00 Å, respectively, and U_{iso} values constrained to 1.2 times the U_{eq} of their pivot atoms.

Neutral atom scattering factors for all atoms and anomalous dispersion corrections for the non-hydrogen atoms were taken from International Tables for Crystallography.[15] Supplementary crystallographic data reported in this paper have been deposited with the Cambridge Crystallographic Data Centre (CCDC 2534803) and can be obtained free of charge from The Cambridge Crystallographic Data Centre via www.ccdc.cam.ac.uk/structures.[16] This report and the CIF file were generated using FinalCif.[17]

Table S3: Crystal data and structure refinement for compound **3e**.

CCDC number	2534803
Empirical formula	$C_{14}H_{11}BrN_2O$
Formula weight	303.16
Temperature [K]	100(2)
Crystal system	orthorhombic
Space group (number)	$P2_12_12_1$ (19)
a [Å]	6.1549(3)
b [Å]	9.2444(4)
c [Å]	22.2176(9)
α [°]	90
β [°]	90
γ [°]	90
Volume [Å^3]	1264.15(10)
Z	4
ρ_{calc} [gcm^{-3}]	1.593
μ [mm^{-1}]	3.240
F(000)	608
Crystal size [mm^3]	0.035×0.056×0.093

Crystal colour	colourless
Crystal shape	needle
Radiation	MoK_α (λ=0.71073 Å)
2θ range [°]	4.77 to 72.72 (0.60 Å)
Index ranges	$-10 \leq h \leq 10$<br>$-15 \leq k \leq 15$<br>$-37 \leq l \leq 36$
Reflections collected	63575
Independent reflections	6154<br>R_{int} = 0.0691<br>R_{sigma} = 0.0436
Completeness to<br>θ = 25.242°	99.9
Data / Restraints /<br>Parameters	6154 / 0 / 166
Goodness-of-fit on F^2	1.013
Final R indexes<br>[I≥2σ(I)]	R_1 = 0.0351<br>wR_2 = 0.0715
Final R indexes<br>[all data]	R_1 = 0.0566<br>wR_2 = 0.0790
Largest peak/hole [eÅ^{-3}]	0.44/−0.30
Flack X parameter	0.038(4)

Atom	x	y	z	U_{eq}
Br1	−0.00889(4)	0.29065(3)	0.22123(2)	0.03050(7)
O1	0.1655(2)	0.40256(15)	0.50380(7)	0.0188(3)
N1	0.6652(3)	0.72494(19)	0.40883(8)	0.0182(3)
N2	0.5318(3)	0.39025(18)	0.48077(8)	0.0153(3)
C1	0.5252(4)	0.8339(2)	0.39883(9)	0.0198(4)
H1	0.579961	0.918873	0.380191	0.024
C2	0.3043(4)	0.8310(2)	0.41394(10)	0.0185(4)
H2	0.214111	0.911687	0.405126	0.022
C3	0.2180(3)	0.7093(2)	0.44195(9)	0.0163(3)
H3	0.068829	0.703412	0.452704	0.020
C4	0.3619(3)	0.5971(2)	0.45328(9)	0.0129(3)
C5	0.3335(3)	0.4532(2)	0.48203(9)	0.0142(3)
C6	0.6982(3)	0.4719(2)	0.44855(9)	0.0160(3)
H6	0.824939	0.491654	0.475468	0.019
C7	0.5770(3)	0.6095(2)	0.43543(9)	0.0139(3)
C8	0.7737(3)	0.3950(2)	0.39051(10)	0.0210(4)
H8A	0.883532	0.455710	0.369982	0.025
H8B	0.843448	0.301984	0.401246	0.025
C9	0.5877(4)	0.3666(2)	0.34802(9)	0.0182(4)
C10	0.4603(4)	0.2426(2)	0.35354(10)	0.0224(4)
H10	0.495814	0.172687	0.383305	0.027
C11	0.2824(4)	0.2194(3)	0.31628(10)	0.0244(4)
H11	0.196250	0.134754	0.320664	0.029
H1BB	0.553(5)	0.309(3)	0.4899(15)	0.037
C12	0.2322(4)	0.3212(2)	0.27264(10)	0.0215(4)
C13	0.3560(4)	0.4454(3)	0.26600(10)	0.0234(4)
H13	0.319782	0.514795	0.236117	0.028
C14	0.5339(4)	0.4673(2)	0.30360(10)	0.0221(4)
H14	0.619962	0.551914	0.298969	0.027

U_{eq} is defined as 1/3 of the trace of the orthogonalized U_{ij} tensor.

Table S4: Anisotropic displacement parameters (Å^2) for compound 33. The anisotropic displacement factor exponent takes the form: $-2\pi^2[\, h^2(a^*)^2U_{11} + k^2(b^*)^2U_{22} + \ldots + 2hka^*b^*U_{12}\,]$.

**Atom**	U_{11}	U_{22}	U_{33}	U_{23}	U_{13}	U_{12}
Br1	0.02408(11)	0.04375(14)	0.02367(10)	−0.00813(9)	−0.00131(11)	0.00334(12)
O1	0.0137(7)	0.0145(6)	0.0281(8)	0.0023(6)	0.0024(6)	−0.0019(5)
N1	0.0178(8)	0.0166(7)	0.0203(8)	0.0023(7)	0.0026(6)	−0.0012(6)
N2	0.0129(8)	0.0131(6)	0.0199(7)	0.0042(6)	−0.0008(6)	0.0024(6)
C1	0.0270(11)	0.0135(7)	0.0189(8)	0.0034(6)	0.0017(8)	−0.0031(8)
C2	0.0208(9)	0.0138(8)	0.0209(9)	0.0007(7)	−0.0025(8)	0.0025(7)
C3	0.0144(7)	0.0139(8)	0.0208(8)	−0.0015(7)	−0.0016(6)	0.0013(7)
C4	0.0133(8)	0.0115(7)	0.0138(8)	−0.0006(7)	−0.0010(6)	0.0003(6)
C5	0.0138(8)	0.0111(7)	0.0178(9)	0.0003(7)	−0.0016(7)	−0.0012(6)
C6	0.0122(8)	0.0162(8)	0.0197(9)	0.0015(7)	−0.0001(7)	0.0015(6)
C7	0.0141(8)	0.0136(8)	0.0141(8)	−0.0006(7)	−0.0012(6)	0.0002(6)
C8	0.0161(9)	0.0216(10)	0.0254(10)	−0.0003(8)	0.0034(8)	0.0049(8)
C9	0.0197(9)	0.0169(9)	0.0179(9)	−0.0029(7)	0.0035(7)	0.0042(7)
C10	0.0302(12)	0.0160(8)	0.0211(9)	−0.0015(7)	0.0002(8)	0.0035(7)
C11	0.0301(11)	0.0194(9)	0.0236(10)	−0.0030(8)	0.0021(8)	−0.0020(9)
C12	0.0217(9)	0.0251(10)	0.0178(9)	−0.0053(8)	0.0022(8)	0.0033(7)
C13	0.0299(11)	0.0231(10)	0.0172(9)	0.0017(8)	0.0018(8)	0.0052(8)
C14	0.0268(12)	0.0208(9)	0.0188(9)	0.0007(7)	0.0048(8)	0.0014(8)

Table S5: Bond lengths and angles for compound **3e**.

**Atom–Atom**	**Length [Å]**
Br1–C12	1.893(2)
O1–C5	1.234(2)
N1–C7	1.335(3)
N1–C1	1.344(3)
N2–C5	1.352(3)
N2–C6	1.460(3)
N2–H1BB	0.79(3)
C1–C2	1.400(3)
C1–H1	0.9500
C2–C3	1.391(3)
C2–H2	0.9500
C3–C4	1.387(3)
C3–H3	0.9500
C4–C7	1.387(3)
C4–C5	1.487(3)
C6–C7	1.503(3)
C6–C8	1.544(3)
C6–H6	1.0000
C8–C9	1.507(3)
C8–H8A	0.9900
C8–H8B	0.9900
C9–C10	1.394(3)
C9–C14	1.397(3)
C10–C11	1.389(3)
C10–H10	0.9500
C11–C12	1.386(3)
C11–H11	0.9500
C12–C13	1.386(3)
C13–C14	1.392(3)
C13–H13	0.9500
C14–H14	0.9500
**Atom–Atom–Atom**	**Angle [°]**
C7–N1–C1	114.30(17)
C5–N2–C6	114.90(16)
C5–N2–H1BB	124(2)
C6–N2–H1BB	120(2)
N1–C1–C2	124.66(18)
N1–C1–H1	117.7
C2–C1–H1	117.7
C3–C2–C1	119.56(18)
C3–C2–H2	120.2
C1–C2–H2	120.2
C4–C3–C2	116.21(18)
C4–C3–H3	121.9
C2–C3–H3	121.9
C7–C4–C3	119.75(18)

C7–C4–C5	108.01(17)
C3–C4–C5	132.23(18)
O1–C5–N2	126.98(18)
O1–C5–C4	127.35(18)
N2–C5–C4	105.65(16)
N2–C6–C7	100.64(15)
N2–C6–C8	112.50(17)
C7–C6–C8	112.16(17)
N2–C6–H6	110.4
C7–C6–H6	110.4
C8–C6–H6	110.4
N1–C7–C4	125.49(18)
N1–C7–C6	124.10(18)
C4–C7–C6	110.39(18)
C9–C8–C6	111.99(17)
C9–C8–H8A	109.2
C6–C8–H8A	109.2
C9–C8–H8B	109.2
C6–C8–H8B	109.2
H8A–C8–H8B	107.9
C10–C9–C14	118.5(2)
C10–C9–C8	121.03(19)
C14–C9–C8	120.4(2)
C11–C10–C9	121.2(2)
C11–C10–H10	119.4
C9–C10–H10	119.4
C12–C11–C10	119.2(2)
C12–C11–H11	120.4
C10–C11–H11	120.4
C13–C12–C11	120.9(2)
C13–C12–Br1	119.35(17)
C11–C12–Br1	119.71(17)
C12–C13–C14	119.3(2)
C12–C13–H13	120.3
C14–C13–H13	120.3
C13–C14–C9	120.9(2)
C13–C14–H14	119.6
C9–C14–H14	119.6

Table S6: Torsion angles for compound **3e**.

Atom–Atom–Atom–Atom	Torsion Angle [°]
C7–N1–C1–C2	−0.3(3)
N1–C1–C2–C3	0.7(3)
C1–C2–C3–C4	0.3(3)
C2–C3–C4–C7	−1.6(3)
C2–C3–C4–C5	179.6(2)
C6–N2–C5–O1	176.3(2)
C6–N2–C5–C4	−5.2(2)
C7–C4–C5–O1	179.7(2)
C3–C4–C5–O1	−1.3(4)
C7–C4–C5–N2	1.2(2)
C3–C4–C5–N2	−179.8(2)
C5–N2–C6–C7	6.7(2)
C5–N2–C6–C8	−112.89(19)
C1–N1–C7–C4	−1.1(3)
C1–N1–C7–C6	177.00(19)
C3–C4–C7–N1	2.1(3)
C5–C4–C7–N1	−178.77(19)
C3–C4–C7–C6	−176.20(18)
C5–C4–C7–C6	2.9(2)
N2–C6–C7–N1	176.13(19)
C8–C6–C7–N1	−64.1(3)
N2–C6–C7–C4	−5.5(2)
C8–C6–C7–C4	114.29(19)
N2–C6–C8–C9	57.6(2)
C7–C6–C8–C9	−55.0(2)
C6–C8–C9–C10	−85.9(2)
C6–C8–C9–C14	91.6(2)
C14–C9–C10–C11	−0.6(3)
C8–C9–C10–C11	176.9(2)
C9–C10–C11–C12	0.4(3)
C10–C11–C12–C13	−0.3(3)
C10–C11–C12–Br1	179.67(17)
C11–C12–C13–C14	0.4(3)
Br1–C12–C13–C14	−179.63(17)
C12–C13–C14–C9	−0.5(3)
C10–C9–C14–C13	0.6(3)
C8–C9–C14–C13	−176.9(2)

9. Synthesis of Racemic Substrates

Methyl 2-(chloromethyl)nicotinate (SI-2)

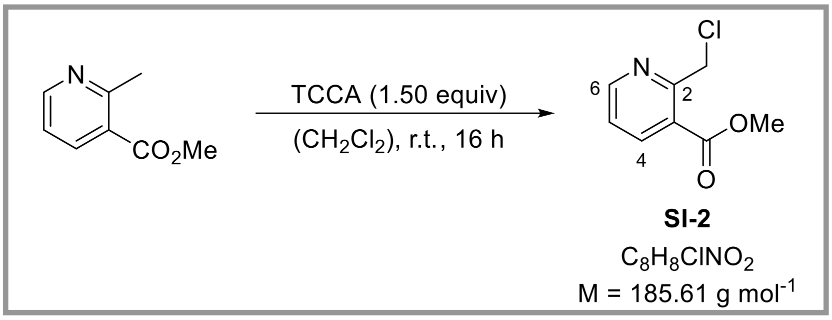

According to a known procedure,[18] trichloroisocyanuric acid (TCCA) (10.4 g, 44.7 mmol, 1.50 equiv.) was added to a solution of methyl 2-methylnicotinate (4.50 g, 29.8 mmol, 1.00 equiv.) in CH_2Cl_2 (25 mL) at room temperature. The suspension was stirred for 16 hours at the same temperature. The mixture was diluted by addition of CH_2Cl_2 (20 mL) and the excess TCCA was filtered off over a plug of Celite©. The filtrate was washed with sat. bicarb solution (100 mL), the organic phase was washed with brine (100 mL) and dried over Na_2SO_4. The solvent was removed under reduced pressure to yield the desired methyl 2-(chloromethyl)nicotinate (**SI-2**) (5.32 g, 28.7 mmol, 96%) without further purification as a pale-yellow oil**.**

TLC (Hex:EtOAc = 9:1): R_f = 0.20 [UV] [$KMnO_4$].

^{1}H-NMR (400 MHz, $CDCl_3$, 300 K): δ [ppm] = 8.72 (dd, ^3J = 4.8 Hz, ^4J = 1.8 Hz, 1H), 8.28 (dd, ^3J = 7.9 Hz, ^4J = 1.8 Hz, 1H), 7.36 (dd, ^3J = 7.9 Hz, ^3J = 4.8 Hz, 1H), 5.12 (s, 2H), 3.96 (s, 3H).

^{13}C-NMR (101 MHz, $CDCl_3$, 300 K): δ [ppm] = 166.0 (COO), 157.4 (C2), 152.4 (C6), 139.3 (C4), 125.6 (C3), 123.4 (C5), 52.8 (CH_3), 45.5 (CH_2Cl).

Spectral data matches those reported in the literature. [18]

Methyl 6-bromo-2-(bromomethyl)nicotinate (SI-1a)

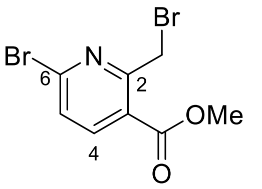

SI-1a

$C_8H_7Br_2NO_2$

M = 308.96 g mol^{-1}

According to **GP A**, NBS (4.22 g, 23.7 mmol, 1.10 equiv.) and AIBN (1.06 g, 6.47 mmol, 0.30 equiv.) were added to a solution of methyl 6-bromo-2-methylpyridine-3-carboxylate (4.96 g, 21.6 mmol, 1.00 equiv.) in DCE (36 mL) at room temperature. The mixture was refluxed for four hours while the solution turned dark orange or dark red depending on the substrate. Afterwards, the mixture was cooled to room temperature, poured into a mixture of *n*-pentane:EtOAc (9:1, 65 mL) and stirred for ten minutes. The resulting precipitate was filtered off over a plug of Celite© and the solvent was removed under reduced pressure. The crude product was subjected to FCC (SiO_2, Hex:Et_2O = 50:1 → 25:1) to yield the desired methyl 6-bromo-2-(bromomethyl)nicotinate (**SI-1a**) (1.68 g, 5.44 mmol, 25%) as a white solid.

TLC (Hex:Et_2O = 50:1): R_f = 0.12 [UV] [$KMnO_4$].

M.p.: 93 °C.

^{1}H-NMR (400 MHz, $CDCl_3$, 300 K): δ [ppm] = 8.12 (d, ^3J = 8.2 Hz, 1H, H4), 7.53 (d, ^3J = 8.3 Hz, 1H, H5), 4.97 (s, 2H, CH_2Cl), 3.98 (s, 3H, CH_3).

^{13}C-NMR (101 MHz, $CDCl_3$, 300 K): δ [ppm] = 165.1 (COO), 159.2 (C2), 144.9 (C6), 141.4 (C4), 128.0 (C5), 124.2 (C3), 53.0 (CH_3), 31.5 (CH_2Br).

HRMS (**ESI**) *m/z* $[M+H]^+$ calculated for $[C_8H_8{}^{79}Br_2NO_2]^+$: 307.8916 found: 307.8914.

IR (film): $\tilde{v}_{max}/cm^{-1}$ = 3074 (w, CH_{arom}), 2953 (w, CH_{aliph}), 1719 (s, C=O), 1567 (m, $C{=}C_{arom}$), 1431 (m, $C{=}C_{arom}$).

Methyl 2-(bromomethyl)-6-(trifluoromethyl)nicotinate (SI-1b)

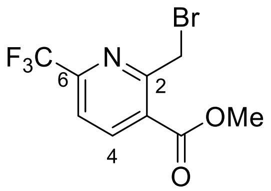

SI-1b

$C_9H_7BrF_3NO_2$

M = 298.06 g mol^{-1}

According to **GP A**, NBS (4.22 g, 23.7 mmol, 1.10 equiv.) and AIBN (1.06 g, 6.47 mmol, 0.30 equiv.) were added to a solution of methyl 2-methyl-6-(trifluoromethyl)nicotinate (4.72 g, 21.6 mmol, 1.00 equiv.) in DCE (36 mL) at room temperature. The mixture was refluxed for four hours while the solution turned dark orange or dark red depending on the substrate. Afterwards, the mixture was cooled to room temperature, poured into a mixture of *n*-pentane:EtOAc (9:1, 65 mL) and stirred for ten minutes. The resulting precipitate was filtered off over a plug of Celite© and the solvent was removed under reduced pressure. The crude product was subjected to FCC (SiO_2,

Hex:Et_2O = 95:5) to yield the desired methyl 2-(bromomethyl)-6-(trifluoromethyl)nicotinate (**SI-1b**) (2.71 g, 9.09 mmol, 42%) as a colorless oil.

TLC (Hex:Et_2O = 95:5): R_f = 0.39 [UV] [$KMnO_4$].
^{1}H-NMR (400 MHz, $CDCl_3$, 300 K): δ [ppm] = 8.44 (d, ^3J = 8.1 Hz, 1H, H4), 7.70 (d, ^3J = 8.1 Hz, 1H, H5), 5.04 (s, 2H, CH_2Br), 4.02 (s, 3H, $COOCH_3$).
^{13}C-NMR (101 MHz, $CDCl_3$, 300 K): δ [ppm] = 165.0 (CO), 158.7 (C2), 150.2 (q, $^2J_{C\text{-}F}$ = 35.5 Hz, C6), 141.1 (C4), 128.0 (C3), 122.2 (C5), 120.9 (q, $^1J_{C\text{-}F}$ = 274.5 Hz, CF_3) 120.0 (q, $^3J_{C\text{-}F}$ = 2.6 Hz, C5), 53.3 ($COOCH_3$), 31.5 (CH_2Br).
^{19}F-NMR (376 MHz, $CDCl_3$, 298K): δ [ppm] = –68.4 (CF_3).
HRMS (ESI) *m/z* $[M+H]^+$ calculated for $[C_9H_8{}^{79}BrF_3NO_2]^+$: 297.9685 found: 297.9687.
IR (film): $\tilde{v}_{max}/cm^{-1}$ = 2959 (m, C_{aliph}), 1733 (s, C=O), 1593 (m, $C{=}C_{arom}$), 1432 (m, $C{=}C_{arom}$), 1286 (m, C-O), 1110 (s, C-F).

6-(4-Methoxybenzyl)-6,7-dihydro-5*H*-pyrrolo[3,4-*b*]pyridin-5-one (SI-3a)

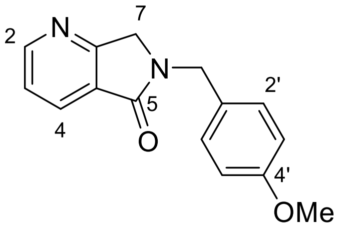

SI-3a
$C_{15}H_{14}N_2O_2$
M = 254.29 g mol^{-1}

According to **GP B**, 4-methoxybenzylamine (PMB-NH_2) (1.31 mL, 1.37 g, 10.0 mmol, 2.00 equiv.) was added in one portion to a solution of methyl 2-(chloromethyl)nicotinate (**SI-2**) (928 mg, 5.00 mmol, 1.00 equiv.) in THF (17 mL) at room temperature. The resulting suspension was stirred for 16 hours at the same temperature. The reaction was quenched by the addition of sat. ammonium chloride solution (60 mL) and water (60 mL) and the mixture was extracted with EtOAc (3 × 70 mL). The combined organic layers were washed with brine (70 mL) and dried over Na_2SO_4. After filtration, the solvent was removed under reduced pressure and the residual crude product was subjected to FCC (SiO_2, Hex:EtOAc = 1:2 → 0:1) to yield the desired 6-(4-methoxybenzyl)-6,7-dihydro-5*H*-pyrrolo[3,4-*b*]pyridin-5-one (**SI-3a**) (791 mg, 3.11 mmol, 62%) as an off-white solid.

TLC (EtOAc): R_f = 0.33 [UV] [$KMnO_4$].
M.p.: 128 °C.
^{1}H-NMR (400 MHz, $CDCl_3$, 300 K): δ [ppm] = 8.65 (dd, ^3J = 4.9 Hz, ^4J = 1.7 Hz, 1H, H2), 8.09 (dd, ^3J = 7.7 Hz, ^4J = 1.7 Hz, 1H, H4), 7.35 (dd, ^3J = 7.7 Hz, 4.9 Hz, 1H, H3), 7.24 – 7.16 (m, 2H, H2', H6'), 6.88 – 6.80 (m, 2H, H3', H5'), 4.74 (s, 2H, N-CH_2-C1'), 4.28 (s, 2H, H7), 3.76 (s, 3H, OCH_3).

^{13}C-NMR (101 MHz, $CDCl_3$, 300 K): δ [ppm] = 166.6 (CO), 162.2 (C7a), 159.4 (C4'), 152.5 (C2), 131.9 (C4), 129.6 (C2', C6'), 128.7 (C1'), 126.5 (C4a), 123.2 (C3), 114.3 (C3', C5'), 55.3 (OCH_3), 51.0 (C7), 45.8 (N-CH_2-C1').

HRMS (**ESI**) *m/z* $[M+H]^+$ calculated for $[C_{15}H_{15}N_2O_2]^+$: 255.1128 found: 255.1155.

IR (film): $\tilde{v}_{max}/cm^{-1}$ = 3074 (w, CH_{arom}), 2937 (w, CH_{aliph}), 1671 (s, C=O), 1606 (m, $C=C_{arom}$), 1512 (m, $C=C_{arom}$).

2-Bromo-6-(4-methoxybenzyl)-6,7-dihydro-5*H*-pyrrolo[3,4-*b*]pyridin-5-one (**SI-3b**)

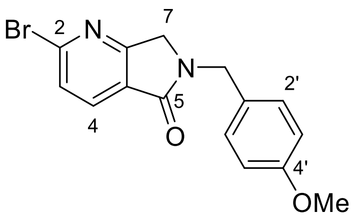

SI-3b
$C_{15}H_{13}BrN_2O_2$
M = 333.19 g mol^{-1}

According to **GP B**, 4-methoxybenzylamine (PMB-NH_2) (1.31 mL, 1.37 g, 10.0 mmol, 2.00 equiv.) was added in one portion to a solution of methyl 2-(chloromethyl)nicotinate (**SI-1a**) (1.55 g, 5.00 mmol, 1.00 equiv.) in THF (17 mL) at room temperature. The resulting suspension was stirred for 16 hours at the same temperature. The reaction was quenched by the addition of sat. ammonium chloride solution (60 mL) and water (60 mL) and the mixture was extracted with EtOAc (3 × 70 mL). The combined organic layers were washed with brine (70 mL) and dried over Na_2SO_4. After filtration, the solvent was removed under reduced pressure and the residual crude product was subjected to FCC (SiO_2, Hex:EtOAc = 2:1) to yield the desired 2-bromo-6-(4-methoxybenzyl)-6,7-dihydro-5*H*-pyrrolo[3,4-*b*]pyridin-5-one (**SI-3b**) (383 mg, 1.15 mmol, 23%) as an off-white solid.

TLC (Hex:EtOAc = 2:1): R_f = 0.35 [UV] [$KMnO_4$].

M.p.: 177 °C.

^{1}H-NMR (400 MHz, $CDCl_3$, 300 K): δ [ppm] = 7.95 (d, ^3J = 8.0 Hz, 1H, H4), 7.57 (d, ^3J = 8.0 Hz, 1H, H3), 7.24 – 7.18 (m, 2H, H2', H6'), 6.90 – 6.81 (m, 2H, H3', H5'), 4.73 (s, 2H, N-CH_2-C1'), 4.28 (s, 2H, H7), 3.78 (s, 3H, OCH_3).

^{13}C-NMR (101 MHz, $CDCl_3$, 300 K): δ [ppm] = 165.8 (C5), 163.4 (C7a), 159.5 (C4'), 145.8 (C2), 133.9 (C4), 129.7 (C2', C6'), 128.4 (C1'), 127.9 (C3), 125.8 (C4a), 114.5 (C3', C5'), 55.4 (OCH_3), 50.6 (C7), 45.9 (N-CH_2-C1').

HRMS (**ESI**) *m/z* $[M+H]^+$ calculated for $[C_{15}H_{14}{}^{79}BrN_2O_2]^+$: 333.0233; found: 333.0226.

IR (film): $\tilde{v}_{max}/cm^{-1}$ = 3107 (w, CH_{arom}), 2930 (w, CH_{aliph}), 1697 (s, C=O), 1655 (m, $C=C_{arom}$), 1593 (m, $C=C_{arom}$), 1247 (m, C-O).

6-(4-Methoxybenzyl)-2-(trifluoromethyl)-6,7-dihydro-5*H*-pyrrolo[3,4-*b*]pyridin-5-one (SI-3c)

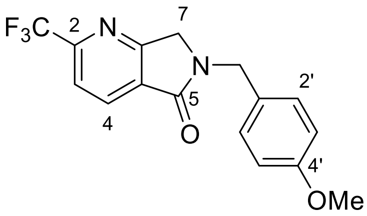

SI-3c
$C_{16}H_{13}F_3N_2O_2$
M = 322.29 g mol^{-1}

According to **GP B**, 4-methoxybenzylamine (PMB-NH_2) (1.31 mL, 1.37 g, 10 mmol, 2.00 equiv.) was added in one portion to a solution of methyl 2-(chloromethyl)nicotinate (**SI-1b**) (1.49 g, 5.00 mmol, 1.00 equiv.) in THF (17 mL) at room temperature. The resulting suspension was stirred for 16 hours at the same temperature The reaction was quenched by the addition of sat. ammonium chloride solution (60 mL) and water (60 mL) and the mixture was extracted with EtOAc (3 × 70 mL). The combined organic layers were washed with brine (70 mL) and dried over Na_2SO_4. After filtration, the solvent was removed under reduced pressure and the residual crude product was subjected to FCC (SiO_2, Hex:EtOAc = 4:1 → 2:1) to yield the desired 6-(4-methoxybenzyl)-2-(trifluoromethyl)-6,7-dihydro-5*H*-pyrrolo[3,4-*b*]pyridin-5-one (**SI-3c**) (1.34 g, 4.16 mmol, 83%) as an off-white solid.

TLC (Hex:EtOAc = 4:1): R_f = 0.32 [UV] [$KMnO_4$].
M.p.: 111 °C.
^{1}H-NMR (400 MHz, $CDCl_3$, 300 K): δ [ppm] = 8.33 (d, ^3J = 8.0 Hz, 1H, H4), 7.81 (d, ^3J = 8.0 Hz, 1H, H3), 7.27 (m, 2H, H2', H6'), 6.89 (m 2H, H3', H5'), 4.80 (s, 2H, N-CH_2-C1'), 4.41 (s, 2H, H7), 3.81 (s, 3H, OCH_3).
^{13}C-NMR (101 MHz, $CDCl_3$, 300 K): δ [ppm] = 165.2 (C5), 162.6 (C7a), 159.6 (C4'), 150.9 (q, $^2J_{C-F}$ = 34.5 Hz, C2), 133.5 (C4), 129.8 (C2', C6'), 129.4 (C1'), 128.2 (C4a), 121.4 (q, $^1J_{C-F}$ = 274.8 Hz, CF_3), 120.3 (q, $^3J_{C-F}$ = 2.6 Hz C3), 114.5 (C3', C5'), 55.5 (OCH_3), 50.9 (C7), 46.1 (N-*C*H_2-C1').
^{19}F-NMR (376 MHz, $CDCl_3$, 298K): δ [ppm] = -67.6 (CF_3).
HRMS (**ESI**) *m/z* [M+H]$^+$ calculated for $[C_{16}H_{14}F_3N_2O_2]^+$: 323.1002; found: 323.1008.
IR (film): $\tilde{v}_{max}$/cm^{-1} = 3064 (w, CH_{arom}), 2967 (w, CH_{aliph}), 1686 (s, C=O), 1609 (m, $C=C_{arom}$), 1513 (m, $C=C_{arom}$).

2-Chloro-6-(4-methoxybenzyl)-6,7-dihydro-5*H*-pyrrolo[3,4-*b*]pyridin-5-one (SI-3d)

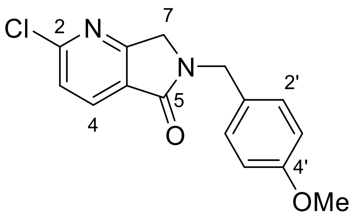

SI-3d
$C_{15}H_{13}ClN_2O_2$
M = 288.73 g mol^{-1}

According to **GP C**, NBS (4.22 g, 23.7 mmol, 1.10 equiv.) and AIBN (1.06 g, 6.47 mmol, 0.30 equiv.) were added to a solution of methyl 6-chloro-2-methylpyridine-3-carboxylate (4.00 g, 21.6 mmol, 1.00 equiv.) in DCE (36 mL) at room temperature. The mixture was refluxed for four hours while the solution turned dark orange. Afterwards, the mixture was cooled to room temperature, poured into a mixture of *n*-pentane:EtOAc (9:1, 65 mL) and stirred for ten minutes. The resulting precipitate was filtered off over a plug of Celite© and the solvent was removed under reduced pressure. Due to insufficient separation according to TLC, the crude product was dissolved in THF (72 mL) and (PMB-NH_2) (5.63 mL, 5.91 g, 43.1 mmol, 2.00 equiv.) was added at room temperature in one portion. The resulting suspension was stirred for 16 hours at the same temperature. The reaction was quenched by the addition of sat. ammonium chloride solution (60 mL) and water (60 mL) and the mixture was extracted with EtOAc (3 × 70 mL). The combined organic layers were washed with brine (70 mL) and dried over Na_2SO_4. After filtration, the solvent was removed under reduced pressure and the residual crude product was subjected to FCC (SiO_2, Hex:EtOAc = 2:1 → 0:1) to yield the desired 2-chloro-6-(4-methoxybenzyl)-6,7-dihydro-5*H*-pyrrolo[3,4-*b*]pyridin-5-one (**SI-3d**) (2.05 g, 7.10 mmol, 33%) as an off-white solid.

TLC (Hex:EtOAc = 2:1): R_f = 0.25 [UV] [$KMnO_4$].

M.p.: 167 °C.

^{1}H-NMR (400 MHz, $CDCl_3$, 300 K): δ [ppm] = 8.06 (d, ^3J = 8.1 Hz, 1H, H4), 7.41 (d, ^3J = 8.1 Hz, 1H, H3), 7.25 – 7.20 (m, 2H, H2', H6'), 6.88 – 6.83 (m, 2H, H3', H5'), 4.73 (s, 2H, N-CH_2-C1'), 4.27 (s, 2H, H7), 3.78 (s, 3H, OCH_3).

^{13}C-NMR (101 MHz, $CDCl_3$, 300 K): δ [ppm] = 165.8 (C5), 163.0 (C7a), 159.5 (C4'), 154.9 (C2), 134.3 (C4), 129.7 (C2', C6'), 128.4 (C1'), 125.5 (C4a), 124.1 (C3), 114.4 (C3', C5'), 55.4 (OCH_3), 50.6 (C7), 45.9 (N-*C*H_2-C1').

HRMS (**ESI**) *m/z* $[M+H]^+$ calculated for $[C_{15}H_{14}{}^{35}ClN_2O_2]^+$: 289.0738; found: 289.0735.

IR (film): $\tilde{\nu}_{max}/cm^{-1}$ = 3066 (w, CH_{arom}), 2928 (w, CH_{aliph}), 1676 (s, C=O), 1611 (m, $C{=}C_{arom}$), 1511 (m, $C{=}C_{arom}$), 1241 (m, C-O).

3-Bromo-6-(4-methoxybenzyl)-6,7-dihydro-5*H*-pyrrolo[3,4-*b*]pyridin-5-one (**SI-3e**)

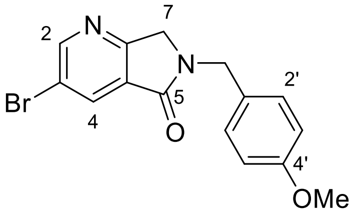

SI-3e
$C_{15}H_{13}BrN_2O_2$
M = 333.19 g mol^{-1}

According to **GP C**, NBS (4.22 g, 23.7 mmol, 1.10 equiv.) and AIBN (1.06 g, 6.47 mmol, 0.30 equiv.) were added to a solution of methyl 5-bromo-2-methylpyridine-3-carboxylate (4.96 g, 21.6 mmol, 1.00 equiv.) in DCE (36 mL) at room temperature. The mixture was refluxed for four hours while the solution turned dark red. Afterwards, the mixture was cooled to room temperature, poured into a mixture of *n*-pentane:EtOAc (9:1, 65 mL) and stirred for ten minutes. The resulting precipitate was filtered off over a plug of Celite© and the solvent was removed under reduced pressure. Due to insufficient separation according to TLC, the crude product was dissolved in THF (72 mL) and (PMB-NH_2) (5.63 mL, 5.91 g, 43.1 mmol, 2.00 equiv.) was added at room temperature in one portion. The resulting suspension was stirred for 16 hours at the same temperature. The reaction was quenched by the addition of sat. ammonium chloride solution (60 mL) and water (60 mL) and the mixture was extracted with EtOAc (3 × 70 mL). The combined organic layers were washed with brine (70 mL) and dried over Na_2SO_4. After filtration, the solvent was removed under reduced pressure and the residual crude product was subjected to FCC (SiO_2, Hex:EtOAc = 2:1 → 0:1) to yield the desired 3-bromo-6-(4-methoxybenzyl)-6,7-dihydro-5*H*-pyrrolo[3,4-*b*]pyridin-5-one (**SI-3e**) (2.60 g, 7.80 mmol, 36%) as an off-white solid.

TLC (Hex:EtOAc = 2:1): R_f = 0.26 [UV] [$KMnO_4$].
M.p.: 160 °C.
^{1}H-NMR (400 MHz, $CDCl_3$, 300 K): δ [ppm] = 8.74 (d, ^4J = 2.2 Hz, 1H, H2), 8.24 (d, ^4J = 2.2 Hz, 1H, H4), 7.26 – 7.21 (m, 2H, H2’, H6’), 6.89 – 6.85 (m, 2H, H3’, H5’), 4.75 (s, 2H, N-CH_2-C1’), 4.26 (s, 2H, H7), 3.79 (s, 3H, OCH_3).
^{13}C-NMR (101 MHz, $CDCl_3$, 300 K): δ [ppm] = 165.3 (C5), 160.4 (C7a), 159.6 (C4’), 153.6 (C2), 134.6 (C4), 129.8 (C2’, C6’), 128.4 (C1’), 128.2 (C4a), 120.6 (C3), 114.5 (C3’, C5’), 55.4 (OCH_3), 50.7 (C7), 46.0 (N-CH_2-C1’).
HRMS (**ESI**) *m/z* [M+H]$^+$ calculated for $[C_{15}H_{14}{}^{79}BrN_2O_2]^+$: 333.0233; found: 333.0230.
IR (film): $\tilde{\nu}_{max}$/cm^{-1} = 3050 (w, CH_{arom}), 2930 (w, CH_{aliph}), 1678 (s, C=O), 1608 (m, $C=C_{arom}$), 1510 (m, $C=C_{arom}$), 1246 (m, C-O).

3-Chloro-6-(4-methoxybenzyl)-6,7-dihydro-5*H*-pyrrolo[3,4-*b*]pyridin-5-one (SI-3f)

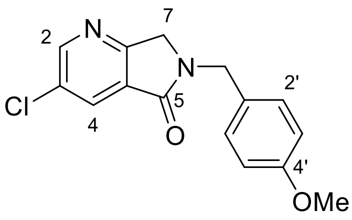

SI-3f

$C_{15}H_{13}ClN_2O_2$

M = 288.73 g mol^{-1}

According to **GP C**, NBS (4.22 g, 23.7 mmol, 1.10 equiv.) and AIBN (1.06 g, 6.47 mmol, 0.30 equiv.) were added to a solution of methyl 5-chloro-2-methylpyridine-3-carboxylate (4.00 g, 21.6 mmol, 1.00 equiv.) in DCE (36 mL) at room temperature. The mixture was refluxed for four hours while the solution turned dark orange. Afterwards, the mixture was cooled to room temperature, poured into a mixture of *n*-pentane:EtOAc (9:1, 65 mL) and stirred for ten minutes. The resulting precipitate was filtered off over a plug of Celite© and the solvent was removed under reduced pressure. Due to insufficient separation according to TLC, the crude product was dissolved in THF (72 mL) and (PMB-NH_2) (5.63 mL, 5.91 g, 43.1 mmol, 2.00 equiv.) was added at room temperature in one portion. The resulting suspension was stirred for 16 hours at the same temperature. The reaction was quenched by the addition of sat. ammonium chloride solution (60 mL) and water (60 mL) and the mixture was extracted with EtOAc (3 × 70 mL). The combined organic layers were washed with brine (70 mL) and dried over Na_2SO_4. After filtration, the solvent was removed under reduced pressure and the residual crude product was subjected to FCC (SiO_2, Hex:EtOAc = 2:1 → 0:1) to yield the desired 3-chloro-6-(4-methoxybenzyl)-6,7-dihydro-5*H*-pyrrolo[3,4-*b*]pyridin-5-one (**SI-3f**) (2.33 g, 8.07 mmol, 37%) as an off-white solid.

TLC (Hex:EtOAc = 2:1): R_f = 0.26 [UV] [$KMnO_4$].

M.p.: 153 °C.

^{1}H-NMR (400 MHz, $CDCl_3$, 300 K): δ [ppm] = 8.63 (d, ^4J = 2.3 Hz, 1H, H2), 8.08 (d, ^4J = 2.3 Hz, 1H, H4), 7.25 – 7.21 (m, 2H, H2’, H6’), 6.89 – 6.84 (m, 2H, H3’, H5’), 4.74 (s, 2H, N-CH_2-C1’), 4.28 (s, 2H, H7), 3.78 (s, 3H, OCH_3).

^{13}C-NMR (101 MHz, $CDCl_3$, 300 K): δ [ppm] = 165.4 (C5), 160.0 (C7a), 159.5 (C4’), 151.5 (C2), 132.3 (C3), 131.6 (C4), 129.7 (C2’, C6’), 128.4 (C1’), 127.7 (C4a), 114.4 (C3’, C5’), 55.4 (OCH_3), 50.6 (C7), 46.0 (N-*C*H_2-C1’).

HRMS (**ESI**) *m/z* $[M+H]^+$ calculated for $[C_{15}H_{14}{}^{35}ClN_2O_2]^+$: 289.0738; found: 289.0735.

IR (film): $\tilde{v}_{max}/cm^{-1}$ = 3054 (w, CH_{arom}), 2934 (w, CH_{aliph}), 1679 (s, C=O), 1610 (m, $C{=}C_{arom}$), 1511 (m, $C{=}C_{arom}$), 1242 (m, C-O).

7-Benzyl-6,7-dihydro-5*H*-pyrrolo[3,4-*b*]pyridin-5-one (*rac*-**3a**)

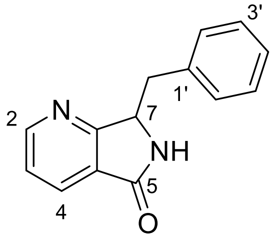

rac-**3a**
$C_{14}H_{12}N_2O$
M = 224.26 g mol^{-1}

According to **GP D**, a 1 M solution of KHMDS in THF (2.16 mL, 432 mg, 2.16 mmol, 1.10 equiv.) was added dropwise to a solution of 6-(4-methoxybenzyl)-6,7-dihydro-5*H*-pyrrolo[3,4-*b*]pyridin-5-one (**SI-3a**) (500 mg, 1.97 mmol, 1.00 equiv.) in anhydrous THF (25 mL) at –78 °C, upon which the reaction mixture turned violet. After 20 minutes, benzyl bromide (304 µL, 437 mg, 2.56 mmol, 1.30 equiv.) was added in one portion. The reaction was stirred for two hours at –78 °C. Subsequently, the solution was allowed to warm up to room temperature and quenched by the addition of sat. NH_4Cl (20 mL) and distilled water (20 mL). The aqueous layer was extracted with EtOAc (3 × 30 mL), and the combined organic phases were dried over Na_2SO_4, filtered and concentrated under reduced pressure. The crude product was then dissolved in MeCN:H_2O (3:1, 58 mL), and diammonium cerium(IV) nitrate (3.23 g, 5.90 mmol, 3.00 equiv.) was added to the solution at room temperature. The yellow solution was stirred for two hours at the same temperature. Subsequently, water (30 mL) was added, and the mixture was extracted with EtOAc (3 × 30 mL). The combined organic layers were washed with brine (50 mL) and dried over Na_2SO_4. After filtration, the solvent was removed under reduced pressure and the residual crude product was subjected to FCC (SiO_2, EtOAc) to yield the desired racemic substrate 7-benzyl-6,7-dihydro-5*H*-pyrrolo[3,4-*b*]pyridin-5-one (*rac*-**3a**) (231 mg, 1.03 mmol, 52%) as a white solid.

TLC (EtOAc): R_f = 0.29 [UV] [$KMnO_4$].
M.p.: 198 °C.
^{1}H-NMR (400 MHz, $CDCl_3$, 300 K): δ [ppm] = 8.82 (dd, ^3J = 5.0 Hz, ^4J = 1.6 Hz, 1H, H2), 8.12 (dd, ^3J = 7.7, ^4J = 1.6 Hz, 1H, H4), 7.45 (dd, ^3J = 7.7 Hz, ^3J = 5.0 Hz, 1H, H3), 7.36 – 7.19 (m, 5H, H1', H2', H3', H4', H5'), 6.71 (bs, 1H, NH), 4.87 (dd, ^3J = 9.5 Hz, ^3J = 3.7 Hz, 1.2 Hz, 1H, H7), 3.65 (dd, ^2J = 13.8 Hz, 3.7 Hz, 1H, C7-CH_a-C1'), 2.77 (dd, ^2J = 13.8, ^3J = 9.5 Hz, 1H, C7-CH_b-C1').
^{13}C-NMR (101 MHz, $CDCl_3$, 300 K): δ [ppm] = 168.3 (C5), 166.3 (C7a), 152.9 (C2), 136.8 (C1'), 132.4 (C4), 129.3 (C3', C5'), 128.9 (C2', C6'), 127.3 (C4'), 126.0 (C4a), 123.6 (C3), 59.7 (C7), 39.7 (C7-CH_2-C1').
HRMS (**ESI**) *m/z* $[M+H]^+$ calculated for $[C_{14}H_{13}N_2O]^+$: 225.1022; found: 225.1017.
IR (film): $\tilde{v}$ max/cm^{-1} = 3167 (m, NH), 3065 (m, CH_{arom}), 2957 (m, CH_{aliph}), 1680 (s, C=O) 1606 (m, C=C_{arom}).

The UV-Vis absorption spectrum of a 1 mM solution of *rac*-**3a** in CH_2Cl_2 was measured in *Hellma* precision cells made of quartz SUPRASIL® with a light pathway of 1 mm on a Perkin Elmer Lambda 35 UV-Vis spectrometer. $PhCF_3$ could not be used due to a significant overlap of its UV-Vis spectrum with the spectrum of *rac*-**3a**.

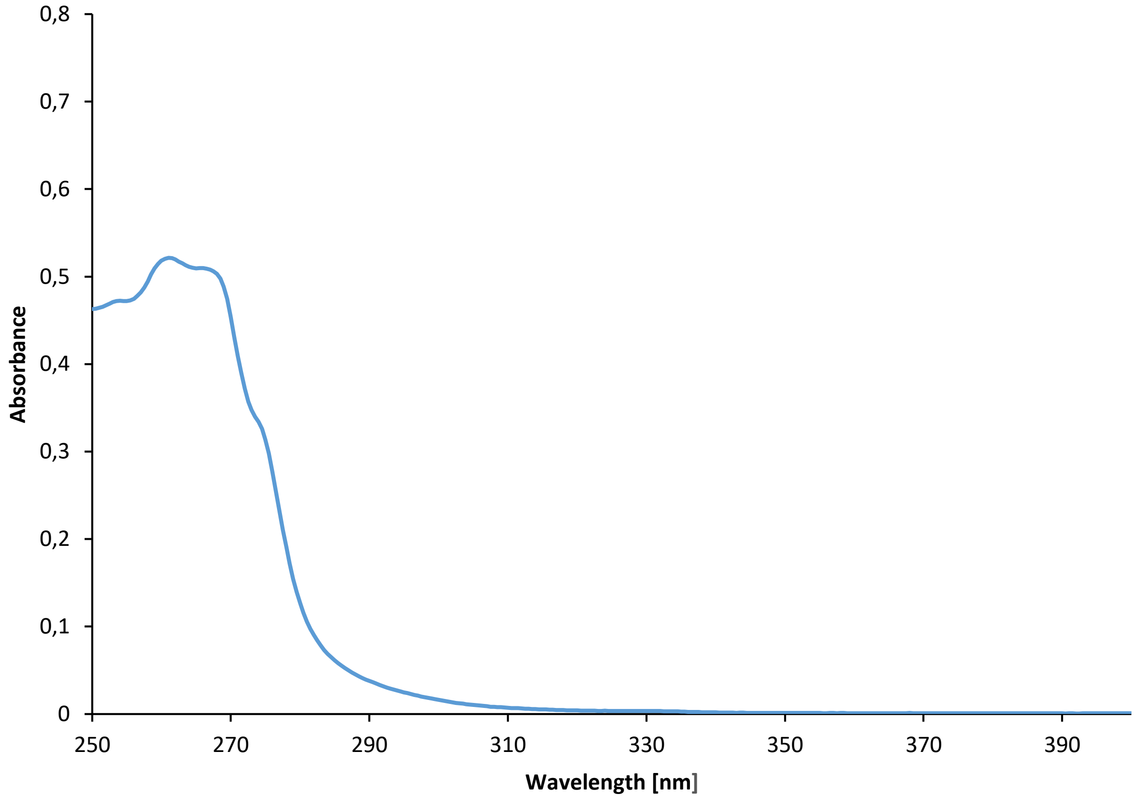

7-(4-(*tert*-Butyl)benzyl)-6,7-dihydro-5*H*-pyrrolo[3,4-*b*]pyridin-5-one (*rac*-**3b**)

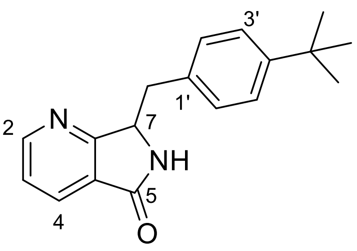

rac-**3b**
$C_{18}H_{20}N_2O$
M = 280.37 g mol^{-1}

According to **GP D**, a 1 M solution of KHMDS in THF (2.16 mL, 432 mg, 2.16 mmol, 1.10 equiv.) was added dropwise to a solution of 6-(4-methoxybenzyl)-6,7-dihydro-5*H*-pyrrolo[3,4-*b*]pyridin-5-one (**SI-3a**) (500 mg, 1.97 mmol, 1.00 equiv.) in anhydrous THF (25 mL) at –78 °C, upon which the reaction mixture turned violet. After 20 minutes, 1-(bromomethyl)-4-(*tert*-butyl)benzene (470 µL, 581 mg, 2.56 mmol, 1.30 equiv.) was added in one portion. The reaction was stirred for two hours at –78 °C. Subsequently, the solution was allowed to warm up to room temperature and quenched by the addition of sat. NH_4Cl (20 mL) and distilled water (20 mL). The aqueous layer was extracted with EtOAc (3 × 30 mL), and the combined organic phases were dried over Na_2SO_4, filtered and concentrated under reduced pressure. The crude product was then dissolved in MeCN:H_2O (3:1, 58 mL), and diammonium cerium(IV) nitrate (3.23 g, 5.90 mmol, 3.00 equiv.) was added to the solution at room temperature. The yellow solution was stirred for two hours at the same temperature. Subsequently, water (30 mL) was added, and the mixture was extracted with EtOAc (3 × 30 mL). The combined organic layers were washed with brine (50 mL) and dried over Na_2SO_4. After filtration, the solvent was removed under reduced pressure and the residual crude product was subjected to FCC (SiO_2, EtOAc) to yield the desired racemic substrate 7-(4-(*tert*-butyl)benzyl)-6,7-dihydro-5*H*-pyrrolo[3,4-*b*]pyridin-5-one (*rac*-**3b**) (300 mg, 1.07 mmol, 54%) as a white solid.

TLC (EtOAc): R_f = 0.31 [UV] [$KMnO_4$].
M.p.: 195 °C.
^{1}H-NMR (400 MHz, $CDCl_3$, 300 K): *δ* [ppm] = 8.83 (dd, ^3J = 5.0 Hz, ^4J = 1.6 Hz, 1H, H2), 8.15 (dd, ^3J = 7.7 Hz, ^4J = 1.6 Hz, 1H, H4), 7.46 (dd, ^3J = 7.7 Hz, ^3J = 5.0 Hz, 1H, H3), 7.42 – 7.37 (m, 2H, H3', H5'), 7.26 – 7.19 (m, 2H, H2', H6'), 6.19 (bs, 1H, NH), 4.82 (dt, ^3J = 10.6 Hz, ^3J = 2.1 Hz, 1H, H7), 3.67 (dd, ^2J = 13.7 Hz, ^3J = 3.4 Hz, 1H, C7-CH$_a$-C1'), 2.61 (dd, ^3J = 13.7 Hz, ^3J = 10.6 Hz, 1H, C7-CH$_b$-C1'), 1.35 [s, 9H, C(CH_3)$_3$].
^{13}C-NMR (101 MHz, $CDCl_3$, 300 K)**:** *δ* [ppm] = 168.1 (C5), 166.4 (C7a), 153.1 (C2), 150.4 (C4'), 134.1 (C1'), 132.4 (C4), 128.9 (C2', C6'), 126.1 (C3', C5'), 125.8 (C4a), 123.6 (C3), 59.9 (C7), 39.5 (C7-CH_2-C1'), 34.7 [*C*(CH_3)$_3$], 31.5 [C(*C*H_3)$_3$],.
HRMS (**ESI**) *m/z* [M+H]$^+$ calculated for [$C_{18}H_{21}N_2O$]$^+$: 281.1648; found: 281.1646.

IR (film) $\tilde{\nu}_{max}/cm^{-1}$**:** 3192 (m, NH), 3086 (m, CH_{arom}), 2964 (m, CH_{aliph}), 1684 (s, C=O) 1606 (m, $C{=}C_{arom}$).

7-(3,5-Dimethylbenzyl)-6,7-dihydro-5*H*-pyrrolo[3,4-*b*]pyridin-5-one (*rac*-**3c**)

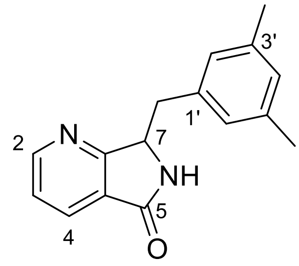

rac-**3c**
$C_{16}H_{16}N_2O$
M = 252.32 g mol^{-1}

According to **GP D**, a 1 M solution of KHMDS in THF (2.16 mL, 432 mg, 2.16 mmol, 1.10 equiv.) was added dropwise to a solution of 6-(4-methoxybenzyl)-6,7-dihydro-5*H*-pyrrolo[3,4-*b*]pyridin-5-one (**SI-3a**) (500 mg, 1.97 mmol, 1.00 equiv.) in anhydrous THF (25 mL) at –78 °C, upon which the reaction mixture turned violet. After 20 minutes, 1-(bromomethyl)-3,5-dimethylbenzene (509 mg, 2.56 mmol, 1.30 equiv.) was added in one portion. The reaction was stirred for two hours at –78 °C. Subsequently, the solution was allowed to warm up to room temperature and quenched by the addition of sat. NH_4Cl (20 mL) and distilled water (20 mL). The aqueous layer was extracted with EtOAc (3 × 30 mL), and the combined organic phases were dried over Na_2SO_4, filtered and concentrated under reduced pressure. The crude product was then dissolved in MeCN:H_2O (3:1, 58 mL), and diammonium cerium(IV) nitrate (3.23 g, 5.90 mmol, 3.00 equiv.) was added to the solution at room temperature. The yellow solution was stirred for two hours at the same temperature. Subsequently, water (30 mL) was added, and the mixture was extracted with EtOAc (3 × 30 mL). The combined organic layers were washed with brine (50 mL) and dried over Na_2SO_4. After filtration, the solvent was removed under reduced pressure and the residual crude product was subjected to FCC (SiO_2, EtOAc) to yield the desired racemic substrate 7-(3,5-dimethylbenzyl)-6,7-dihydro-5*H*-pyrrolo[3,4-*b*]pyridin-5-one (*rac*-**3c**) (170 mg, 678 µmol, 34%) as a white solid.

TLC (EtOAc): $R_f = 0.27$ [UV] [$KMnO_4$].
M.p.: 180 °C.
^{1}H-NMR (400 MHz, $CDCl_3$, 300 K): δ [ppm] = 8.79 (dd, $^3J = 5.0$ Hz, $^4J = 1.6$ Hz, 1H, H2), 8.12 (dd, $^3J = 7.7$ Hz, $^4J = 1.6$ Hz, 1H, H4), 7.42 (dd, $^3J = 7.7$ Hz, $^3J = 5.0$ Hz, 1H, H3), 6.89 (s, 1H, H4'), 6.86 (s, 2H, H2', H6'), 6.47 (bs, 1H, NH), 4.78 (dd, $^3J = 10.1$ Hz, $^3J = 3.5$ Hz, 1H, H7), 3.58 (dd, $^2J = 13.7$ Hz, $^3J = 3.5$ Hz, 1H, C7-CH_a-C1'), 2.54 (dd, $^3J = 13.7$ Hz, $^3J = 10.1$ Hz, 1H, C7-CH_b-C1'), 2.28 (s, 6H, C3'-CH_3, C5'-CH_3).

^{13}C-NMR (101 MHz, $CDCl_3$, 300 K)**:** δ [ppm] = 168.2 (C5), 166.4 (C7a), 152.9 (C2), 138.6 (C3', C5'), 137.0 (C1'), 132.4 (C4), 128.9 (C4'), 127.0 (C2', C6'), 125.9 (C4a), 123.6 (C3), 60.0 (C7), 39.8 (C7-*C*H_2-C1'), 21.4 (C3'-*C*H_3, C5'-*C*H_3).

HRMS (**ESI**) *m/z* $[M+H]^+$ calculated for $[C_{16}H_{17}N_2O]^+$: 253.1335; found: 253.1332.

IR (film) $\tilde{\nu}_{max}/cm^{-1}$**:** 3212 (m, NH), 3082 (m, CH_{arom}), 2982 (m, CH_{aliph}), 1710 (s, C=O) 1609 (m, $C{=}C_{arom}$).

7-(4-Fluorobenzyl)-6,7-dihydro-5*H*-pyrrolo[3,4-*b*]pyridin-5-one (*rac*-**3d**)

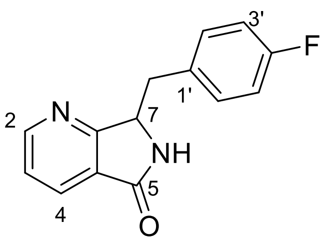

rac-**3d**
$C_{14}H_{11}FN_2O$
M = 242.25 g mol^{-1}

According to **GP D**, a 1 M solution of KHMDS in THF (2.16 mL, 432 mg, 2.16 mmol, 1.10 equiv.) was added dropwise to a solution of 6-(4-methoxybenzyl)-6,7-dihydro-5*H*-pyrrolo[3,4-*b*]pyridin-5-one (**SI-3a**) (500 mg, 1.97 mmol, 1.00 equiv.) in anhydrous THF (25 mL) at –78 °C, upon which the reaction mixture turned violet. After 20 minutes, 1-(bromomethyl)-4-fluorobenzene (318 µL, 483 mg, 2.56 mmol, 1.30 equiv.) was added in one portion. The reaction was stirred for two hours at –78 °C. Subsequently, the solution was allowed to warm up to room temperature and quenched by the addition of sat. NH_4Cl (20 mL) and distilled water (20 mL). The aqueous layer was extracted with EtOAc (3 × 30 mL), and the combined organic phases were dried over Na_2SO_4, filtered and concentrated under reduced pressure. The crude product was then dissolved in MeCN:H_2O (3:1, 58 mL), and diammonium cerium(IV) nitrate (3.23 g, 5.90 mmol, 3.00 equiv.) was added to the solution at room temperature. The yellow solution was stirred for two hours at the same temperature. Subsequently, water (30 mL) was added, and the mixture was extracted with EtOAc (3 × 30 mL). The combined organic layers were washed with brine (50 mL) and dried over Na_2SO_4. After filtration, the solvent was removed under reduced pressure and the residual crude product was subjected to FCC (SiO_2, EtOAc) to yield the desired racemic substrate 7-(4-fluorobenzyl)-6,7-dihydro-5*H*-pyrrolo[3,4-*b*]pyridin-5-one (*rac*-**3d**) (289 mg, 1.19 mmol, 61%) as a white solid.

TLC (EtOAc): R_f = 0.17 [UV] [$KMnO_4$].

M.p.: 193 °C.

^{1}H-NMR (400 MHz, $CDCl_3$, 300 K): δ [ppm] = 8.81 (d, ^3J = 4.9 Hz, 1H, H2), 8.09 (d, ^3J = 7.5 Hz, 1H, H4), 7.43 (dd, ^3J = 7.5 Hz, ^3J = 4.8 Hz, 1H, H3), 7.14 (m, 2H, H2', H6'), 7.06 (bs, 1H, NH), 6.95 (t, J = 8.4 Hz, 2H, H3', H5'), 4.86 (dd, ^3J = 8.5 Hz, ^3J = 3.7 Hz, 1H, H7),

3.56 (dd, ^3J = 14.0 Hz, 3.4 Hz, 1H, C7-CH_a-C1'), 2.86 (dd, ^3J = 14.0 Hz, ^3J = 8.5 Hz, 1H, C7-CH_b-C1').

^{13}C-NMR (101 MHz, $CDCl_3$, 300 K)**:** δ [ppm] = 168.7 (C5), 166.1 (C7a), 162.10 (d, $^1J_{C\text{-}F}$ = 245.6 Hz), 153.0 (C2), 132.4 (C4), 132.1 (d, $^4J_{C\text{-}F}$ = 3.2 Hz, C1'), 131.0 (d,$^3J_{C\text{-}F}$ = 8.0 Hz, C2', C6'), 126.1 (C4a), 123.6 (C3), 115.7 (d, $^2J_{C\text{-}F}$ = 21.3 Hz, C3', C5'), 59.6 (C7), 38.6 (C7-CH$_2$-C1').

^{19}F-NMR (376MHz, $CDCl_3$, 300 K): δ[ppm] = −115.6.

HRMS (**ESI**) *m/z* $[M+H]^+$ calculated for $[C_{14}H_{12}FN_2O]^+$: 243.0928; found: 243.0926.

IR (film) $\tilde{\nu}_{max}$/cm^{-1}**:** 3380 (m, NH), 3089 (m, CH_{arom}), 2944 (m, CH_{aliph}), 1691 (s, C=O) 1603 (m, $C{=}C_{arom}$).

7-(4-Bromobenzyl)-6,7-dihydro-5*H*-pyrrolo[3,4-*b*]pyridin-5-one (*rac*-**3e**)

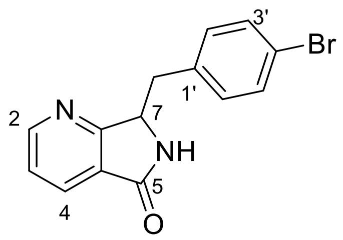

rac-**3e**
$C_{14}H_{11}BrN_2O$
M = 303.16 g mol^{-1}

According to **GP D**, a 1 M solution of KHMDS in THF (2.16 mL, 432 mg, 2.16 mmol, 1.10 equiv.) was added dropwise to a solution of 6-(4-methoxybenzyl)-6,7-dihydro-5*H*-pyrrolo[3,4-*b*]pyridin-5-one (**SI-3a**) (500 mg, 1.97 mmol, 1.00 equiv.) in anhydrous THF (25 mL) at –78 °C, upon which the reaction mixture turned violet. After 20 minutes, 1-bromo-4-(bromomethyl)benzene (639 mg, 2.56 mmol, 1.30 equiv.) was added in one portion. The reaction was stirred for two hours at –78 °C. Subsequently, the solution was allowed to warm up to room temperature and quenched by the addition of sat. NH_4Cl (20 mL) and distilled water (20 mL). The aqueous layer was extracted with EtOAc (3 × 30 mL), and the combined organic phases were dried over Na_2SO_4, filtered and concentrated under reduced pressure. The crude product was then dissolved in MeCN:H_2O (3:1, 58 mL), and diammonium cerium(IV) nitrate (3.23 g, 5.90 mmol, 3.00 equiv.) was added to the solution at room temperature. The yellow solution was stirred for two hours at the same temperature. Subsequently, water (30 mL) was added, and the mixture was extracted with EtOAc (3 × 30 mL). The combined organic layers were washed with brine (50 mL) and dried over Na_2SO_4. After filtration, the solvent was removed under reduced pressure and the residual crude product was subjected to FCC (SiO_2, EtOAc) to yield the desired racemic substrate 7-(4-bromobenzyl)-6,7-dihydro-5*H*-pyrrolo[3,4-*b*]pyridin-5-one (*rac*-**3e**) (349 mg, 1.15 mmol, 59%) as a white solid.

TLC (EtOAc): R_f = 0.20 [UV] [$KMnO_4$].

M.p.: 193 °C.

^{1}H-NMR (400 MHz, $CDCl_3$, 300 K): δ [ppm] = 8.78 (dd, ^{3}J = 4.9 Hz, ^{4}J = 1.6 Hz, 1H, H2), 8.07 (dd, ^{3}J = 7.7 Hz, ^{4}J = 1.6 Hz, 1H, H4), 7.41 (dd, ^{3}J = 7.7 Hz, ^{3}J = 4.9 Hz, 1H, H3), 7.39 (bs, 1H, NH), 7.35 – 7.31 (m, 2H, H3', H5'), 7.05 – 6.96 (m, 2H, H2', H6'), 4.84 (dd, ^{3}J = 8.2 Hz, ^{3}J = 3.9 Hz, 1H, H7), 3.50 (dd, ^{2}J = 13.9 Hz, ^{3}J = 3.9 Hz, 1H, C7-CH_a-C1'), 2.86 (dd, ^{2}J = 13.9 Hz, ^{3}J = 8.2 Hz, 1H, C7-CH_b-C1').

^{13}C-NMR (101 MHz, $CDCl_3$, 300 K)**:** δ [ppm] = 168.8 (C5), 166.0 (C7a), 152.9 (C2), 135.4 (C1'), 132.4 (C4), 131.8 (C3', C5'), 131.2 (C2', C6'), 126.0 (C4a), 123.6 (C3), 121.2 (C4'), 59.3 (C7), 38.7 (C7-CH_2-C1').

HRMS (**ESI**) *m/z* $[M+H]^+$ calculated for $[C_{14}H_{12}{}^{79}BrN_2O]^+$: 303.0128; found: 303.0121.

IR (film) $\tilde{\nu}_{max}/cm^{-1}$**:** 3180 (m, NH), 3087 (m, CH_{arom}), 2920 (m, CH_{aliph}), 1689 (s, C=O) 1605 (m, $C{=}C_{arom}$).

7-(Cyclopropylmethyl)-6,7-dihydro-5*H*-pyrrolo[3,4-*b*]pyridin-5-one (*rac*-**3f**)

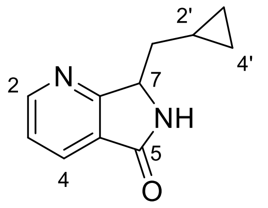

rac-**3f**

$C_{11}H_{12}N_2O$

M = 188.23 g mol^{-1}

According to **GP E**, a 1 M solution of KHMDS in THF (2.16 mL, 432 mg, 2.16 mmol, 1.10 equiv.) was added dropwise to a solution of 6-(4-methoxybenzyl)-6,7-dihydro-5*H*-pyrrolo[3,4-*b*]pyridin-5-one (**SI-3a**) (500 mg, 1.97 mmol, 1.00 equiv.) in anhydrous THF (25 mL) at –78 °C, upon which the reaction mixture turned violet. After 20 minutes, (iodomethyl)cyclopropane (264 µL, 465 mg, 2.56 mmol, 1.30 equiv.) was added in one portion and the reaction was left to stir for two hours at room temperature. Subsequently, the reaction was quenched by the addition of sat. NH_4Cl (20 mL) and distilled water (20 mL). The aqueous layer was extracted with EtOAc (3 × 30 mL), and the combined organic phases were dried over Na_2SO_4, filtered and concentrated under reduced pressure. The crude product was then dissolved in MeCN:H_2O (3:1, 58 mL), and diammonium cerium(IV) nitrate (3.23 g, 5.90 mmol, 3.00 equiv.) was added to the solution at room temperature. The yellow solution was stirred for two hours at the same temperature. Subsequently, water (30 mL) was added, and the mixture was extracted with EtOAc (3 × 30 mL). The combined organic layers were washed with brine (50 mL) and dried over Na_2SO_4. After filtration, the solvent was removed under reduced pressure and the residual crude product was subjected to FCC (SiO_2, EtOAc) to yield the desired racemic substrate 7-(cyclopropylmethyl)-6,7-dihydro-5*H*-pyrrolo[3,4-*b*]pyridin-5-one (*rac*-**3f**) (116 mg, 611 µmol, 31%) as a white solid.

TLC (EtOAc): R_f = 0.21 [UV] [$KMnO_4$].

M.p.: 153 °C.

^{1}H-NMR (400 MHz, $CDCl_3$, 300 K): δ [ppm] = 8.74 (dd, ^3J = 4.9 Hz, ^4J = 1.6 Hz, 1H, H2), 8.13 (dd, ^3J = 7.7 Hz, ^4J = 1.6 Hz, 1H, H4), 7.46 – 7.35 (m, 2H, H3, NH), 4.73 (dd, ^3J = 8.4 Hz, ^3J = 4.0 Hz, 1H, H7), 2.15 (ddd, ^2J = 14.2 Hz, ^3J = 6.7 Hz, ^3J = 4.0 Hz, 1H, H_a1'), 1.59 – 1.47 (m, 1H, H_b1'), 0.91 – 0.75 (m, 1H, H2'), 0.57 – 0.40 (m, 2H, H_a3', H_a4'), 0.21 – 0.13 (m, 1H, H_b3'), 0.13 – 0.05 (m, 1H, H_b4').

^{13}C-NMR (101 MHz, $CDCl_3$, 300 K)**:** δ [ppm] = 168.9 (C5), 167.1 (C7a), 152.7 (C2), 132.3 (C4), 125.8 (C4a), 123.3 (C3), 59.2 (C7), 38.2 (C1'), 7.7 (C2'), 5.1 (C4'), 4.0 (C3').

HRMS (**ESI**) *m/z* $[M+H]^+$ calculated for $[C_{11}H_{13}N_2O]^+$: 189.1022; found: 189.1021.

IR (film) $\tilde{\nu}_{max}/cm^{-1}$**:** 3175 (m, NH), 3073 (m, CH_{arom}), 2956 (m, CH_{aliph}), 2920 (m, CH_{aliph}), 1697 (s, C=O) 1607 (m, $C{=}C_{arom}$).

7-(Cyclobutylmethyl)-6,7-dihydro-5*H*-pyrrolo[3,4-*b*]pyridin-5-one (*rac*-**3g**)

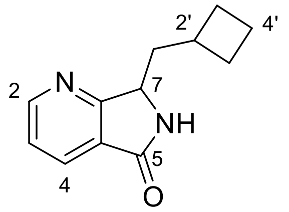

rac-**3g**

$C_{12}H_{14}N_2O$

M = 202.26 g mol^{-1}

According to **GP E**, a 1 M solution of KHMDS in THF (2.16 mL, 432 mg, 2.16 mmol, 1.10 equiv.) was added dropwise to a solution of 6-(4-Methoxybenzyl)-6,7-dihydro-5*H*-pyrrolo[3,4-*b*]pyridin-5-one (**SI-3a**) (500 mg, 1.97 mmol, 1.00 equiv.) in anhydrous THF (25 mL) at –78 °C, upon which the reaction mixture turned violet. After 20 minutes, (iodomethyl)cyclobutane (501 mg, 2.56 mmol, 1.30 equiv.) was added in one portion and the reaction was left to stir for two hours at room temperature. Subsequently, the reaction was quenched by the addition of sat. NH_4Cl (20 mL) and distilled water (20 mL). The aqueous layer was extracted with EtOAc (3 × 30 mL), and the combined organic phases were dried over Na_2SO_4, filtered and concentrated under reduced pressure. The crude product was then dissolved in MeCN:H_2O (3:1, 58 mL), and diammonium cerium(IV) nitrate (3.23 g, 5.90 mmol, 3.00 equiv.) was added to the solution at room temperature. The yellow solution was stirred for two hours at the same temperature. Subsequently, water (30 mL) was added, and the mixture was extracted with EtOAc (3 × 30 mL). The combined organic layers were washed with brine (50 mL) and dried over Na_2SO_4. After filtration, the solvent was removed under reduced pressure and the residual crude product was subjected to FCC (SiO_2, EtOAc) to yield the desired racemic substrate 7-(cyclobutylmethyl)-6,7-dihydro-5*H*-pyrrolo[3,4-*b*]pyridin-5-one (*rac*-**3g**) (226 mg, 1.11 mmol, 56%) as a white solid.

TLC (EtOAc): R_f = 0.24 [UV] [$KMnO_4$].

M.p.: 135 °C.

^{1}H-NMR (400 MHz, $CDCl_3$, 300 K): δ [ppm] = 8.74 (dd, ^3J = 4.9 Hz, ^4J = 1.6 Hz, 1H, H2), 8.12 (dd, ^3J = 7.7 Hz, ^4J = 1.6 Hz, 1H, H4), 7.46 (bs, 1H, NH), 7.38 (dd, ^3J = 7.7 Hz, ^3J = 4.9 Hz, 1H, H3), 4.60 (dd, ^3J = 7.8 Hz, ^3J = 4.2 Hz, 1H, H7), 2.53 – 2.37 (m, 1H, H2'), 2.28 – 2.19 (m, 1H, H_a1'), 2.16 – 1.98 (m, 1H, H_a3'), 1.93 – 1.70 (m, 5H, H_b1', H4', H5'), 1.68 – 1.52 (m, 1H, H_b3').

^{13}C-NMR (101 MHz, $CDCl_3$, 300 K)**:** δ [ppm] = 169.1 (C5), 167.3 (C7a), 152.8 (C2), 132.2 (C4), 125.8 (C4a), 123.2 (C3), 57.6 (C7), 39.9 (C1'), 32.9 (C2'), 29.0 (C5'), 28.6 (C3'), 18.9 (C4').

HRMS (**ESI**) *m/z* $[M+H]^+$ calculated for $[C_{12}H_{15}N_2O]^+$: 203.1179; found: 203.1175.

IR (film) $\tilde{\nu}_{max}/cm^{-1}$**:** 3180 (m, NH), 3089 (m, CH_{arom}), 2955 (m, CH_{aliph}), 2931 (m, CH_{aliph}), 1689 (s, C=O) 1609 (m, $C=C_{arom}$).

***tert*-Butyl 3-((5-oxo-6,7-dihydro-5*H*-pyrrolo[3,4-*b*]pyridin-7-yl)methyl)azetidine-1-carboxylate** (*rac*-**3h**)

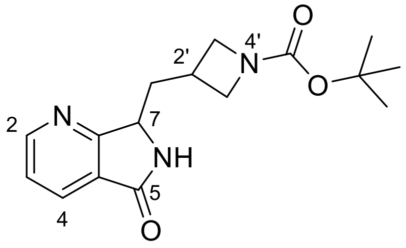

rac-**3h**
$C_{16}H_{21}N_3O_3$
M = 303.36 g mol^{-1}

According to **GP E**, a 1 M solution of KHMDS in THF (2.16 mL, 432 mg, 2.16 mmol, 1.10 equiv.) was added dropwise to a solution of 6-(4-methoxybenzyl)-6,7-dihydro-5*H*-pyrrolo[3,4-*b*]pyridin-5-one (**SI-3a**) (500 mg, 1.97 mmol, 1.00 equiv.) in anhydrous THF (25 mL) at –78 °C, upon which the reaction mixture turned violet. After 20 minutes, *tert*-butyl 3-(iodomethyl)azetidine-1-carboxylate (760 mg, 2.56 mmol, 1.30 equiv.) was added in one portion and the reaction was left to stir for two hours at room temperature. Subsequently, the reaction was quenched by the addition of sat. NH_4Cl (20 mL) and distilled water (20 mL). The aqueous layer was extracted with EtOAc (3 × 30 mL), and the combined organic phases were dried over Na_2SO_4, filtered and concentrated under reduced pressure. The crude product was then dissolved in MeCN:H_2O (3:1, 58 mL), and diammonium cerium(IV) nitrate (3.23 g, 5.90 mmol, 3.00 equiv.) was added to the solution at room temperature. The yellow solution was stirred for two hours at the same temperature. Subsequently, water (30 mL) was added, and the mixture was extracted with EtOAc (3 × 30 mL). The combined organic layers were washed with brine (50 mL) and dried over Na_2SO_4. After filtration, the solvent was removed under reduced pressure and the residual crude product was subjected to FCC (SiO_2, Hex:EtOAc = 2:1 → 0:1) to yield the desired

racemic substrate *tert*-butyl 3-((5-oxo-6,7-dihydro-5*H*-pyrrolo[3,4-*b*]pyridin-7-yl)methyl)azetidine-1-carboxylate (*rac*-**3h**) (44.5 mg, 147 µmol, 7%) as a white solid.

TLC (EtOAc): R_f = 0.16 [UV] [$KMnO_4$].
M.p.: 205 °C.
^{1}H-NMR (400 MHz, $CDCl_3$, 300 K): δ [ppm] = 8.76 (dd, ^3J = 5.0 Hz, ^4J = 1.6 Hz, 1H, H2), 8.15 (dd, ^3J = 7.7 Hz, ^4J = 1.6 Hz, 1H, H4), 7.44 (dd, ^3J = 7.7 Hz, ^3J = 5.0 Hz, 1H, H3), 7.24 (bs, 1H, NH), 4.67 (*virt*. t, ^3J = 5.8 Hz, 1H, H7), 4.05 (*virt*. t, ^3J = 8.5 Hz, 1H, H_a3'), 3.87 (*virt*. t, ^3J = 8.4 Hz, 1H, H_a5'), 3.68 (dd, ^3J = 8.5 Hz, ^3J = 5.8 Hz, 1H, Hb3'), 3.52 (dd, ^3J = 8.4 Hz, ^3J = 5.8 Hz, 1H, H_b5'), 2.75 – 2.60 (m, 1H, H2'), 2.44 – 2.32 (m, 1H, H_a1'), 2.24 – 2.13 (m, 1H, H_b1'), 1.41 [s, 9H, $C(CH_3)_3$].
^{13}C-NMR (101 MHz, $CDCl_3$, 300 K)**:** δ [ppm] = 168.9 (C5), 166.4 (C7a), 156.3 (COO), 153.0 (C2), 132.5 (C4), 125.8 (C4a), 123.7 (C3), 79.6 [*C*(CH_3)$_3$], 56.9 (C7), 54.8 (C3'), 54.6 (C5'), 37.4 (C1'), 28.5 [C(*C*H_3)$_3$], 25.5 (C2').
HRMS (**ESI**) *m/z* $[M+H]^+$ calculated for $[C_{16}H_{22}N_3O_3]^+$: 304.1656; found: 304.1649.
IR (film) $\tilde{\nu}_{max}$/cm^{-1}**:** 3214 (m, NH), 3083 (m, CH_{arom}), 2959 (m, CH_{aliph}), 2925 (m, CH_{aliph}), 1705 (s, C=O), 1682 (s, C=O), 1611 (m, $C{=}C_{arom}$), 1133 (s, C-O).

7-Ethyl-6,7-dihydro-5*H*-pyrrolo[3,4-*b*]pyridin-5-one (*rac*-**3i**)

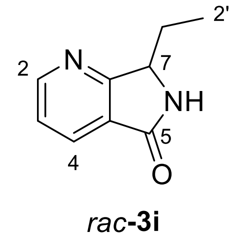

rac-**3i**
$C_9H_{10}N_2O$
M = 162.19 g mol^{-1}

According to **GP E**, a 1 M solution of KHMDS in THF (2.16 mL, 432 mg, 2.16 mmol, 1.10 equiv.) was added dropwise to a solution of 6-(4-methoxybenzyl)-6,7-dihydro-5*H*-pyrrolo[3,4-*b*]pyridin-5-one (**SI-3a**) (500 mg, 1.97 mmol, 1.00 equiv.) in anhydrous THF (25 mL) at –78 °C, upon which the reaction mixture turned violet. After 20 minutes, iodoethane (207 µL, 399 mg, 2.56 mmol, 1.30 equiv.) was added in one portion and the reaction was left to stir for two hours at room temperature. Subsequently, the reaction was quenched by the addition of sat. NH_4Cl (20 mL) and distilled water (20 mL). The aqueous layer was extracted with EtOAc (3 × 30 mL), and the combined organic phases were dried over Na_2SO_4, filtered and concentrated under reduced pressure. The crude product was then dissolved in MeCN:H_2O (3:1, 58 mL), and diammonium cerium(IV) nitrate (3.23 g, 5.90 mmol, 3.00 equiv.) was added to the solution at room temperature. The yellow solution was stirred for two hours at the same temperature. Subsequently, water (30 mL) was added, and the mixture was extracted with EtOAc (3 × 30 mL). The combined organic layers were washed with brine (50 mL) and dried over

Na_2SO_4. After filtration, the solvent was removed under reduced pressure and the residual crude product was subjected to FCC (SiO_2, EtOAc) to yield the desired racemic substrate 7-ethyl-6,7-dihydro-5*H*-pyrrolo[3,4-*b*]pyridin-5-one (*rac*-**3i**) (38.5 mg, 237 µmol, 12%) as an off-white solid.

TLC (EtOAc): $R_f = 0.20$ [UV] [$KMnO_4$].
M.p.: 167 °C.
^{1}H-NMR (400 MHz, $CDCl_3$, 300 K): δ [ppm] = 8.75 (dd, $^3J = 5.0$ Hz, $^4J = 1.6$ Hz, 1H, H2), 8.13 (dd, $^3J = 7.7$ Hz, $^4J = 1.6$ Hz, 1H, H4), 7.80 (bs, 1H, NH), 7.39 (dd, $^3J = 7.7$ Hz, $^4J = 5.0$ Hz, 1H, H3), 4.64 (dd, $^3J = 7.4$ Hz, $^3J = 4.5$ Hz, 1H, H7), 2.17 (ddq, $^2J = 14.7$ Hz, $^3J = 7.4$ Hz, $^3J = 4.5$ Hz, 1H, C1'H_a), 1.83 (*virt.* dp, $^2J = 14.7$ Hz, $^3J = 7.4$ Hz, 1H, C1'H_b), 0.96 (t, $^3J = 7.4$ Hz, 3H. H2').
^{13}C-NMR (101 MHz, $CDCl_3$, 300 K)**:** δ [ppm] = 169.5 (C5), 167.1 (C7a), 152.9 (C2), 132.0 (C4), 126.0 (C4a), 123.2 (C3), 60.0 (C7), 26.1 (C1'), 9.4 (C2').
HRMS (**ESI**) *m/z* $[M+H]^+$ calculated for $[C_9H_{11}N_2O]^+$: 163.0866; found: 163.0861.
IR (film) $\tilde{\nu}_{max}/cm^{-1}$**:** 3154 (m, NH), 3078 (m, CH_{arom}), 2970 (m, CH_{aliph}), 2930 (m, CH_{aliph}), 1690 (s, C=O) 1584 (m, $C{=}C_{arom}$).

7-Butyl-6,7-dihydro-5*H*-pyrrolo[3,4-*b*]pyridin-5-one (*rac*-**3j**)

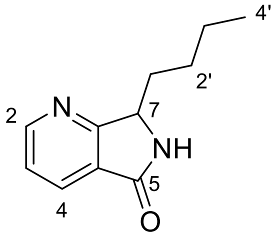

rac-**3j**
$C_{11}H_{14}N_2O$
M = 190.25 g mol^{-1}

According to **GP E**, a 1 M solution of KHMDS in THF (2.16 mL, 432 mg, 2.16 mmol, 1.10 equiv.) was added dropwise to a solution of 6-(4-methoxybenzyl)-6,7-dihydro-5*H*-pyrrolo[3,4-*b*]pyridin-5-one (**SI-3a**) (500 mg, 1.97 mmol, 1.00 equiv.) in anhydrous THF (25 mL) at –78 °C, upon which the reaction mixture turned violet. After 20 minutes, 1-iodobutane (290 µL, 470 mg, 2.56 mmol, 1.30 equiv.) was added in one portion and the reaction was left to stir for two hours at room temperature. Subsequently, the reaction was quenched by the addition of sat. NH_4Cl (20 mL) and distilled water (20 mL). The aqueous layer was extracted with EtOAc (3 × 30 mL), and the combined organic phases were dried over Na_2SO_4, filtered and concentrated under reduced pressure. The crude product was then dissolved in MeCN:H_2O (3:1, 58 mL), and diammonium cerium(IV) nitrate (3.23 g, 5.90 mmol, 3.00 equiv.) was added to the solution at room temperature. The yellow solution was stirred for two hours at the same temperature. Subsequently, water (30 mL) was added, and the mixture was extracted with EtOAc (3 × 30 mL). The combined organic layers were washed with brine (50 mL) and dried

over Na_2SO_4. After filtration, the solvent was removed under reduced pressure and the residual crude product was subjected to FCC (SiO_2, Hex:EtOAc = 1:3 → 0:1) to yield the desired racemic substrate 7-butyl-6,7-dihydro-5*H*-pyrrolo[3,4-*b*]pyridin-5-one (*rac*-**3j**) (189 mg, 994 µmol, 51%) as a white solid.

TLC (EtOAc): R_f = 0.21 [UV] [$KMnO_4$].
M.p.: 141 °C.
^{1}H-NMR (400 MHz, $CDCl_3$, 300 K): δ [ppm] = 8.75 (dd, ^3J = 4.9 Hz, ^4J = 1.6 Hz, 1H, H2), 8.12 (dd, ^3J = 7.7 Hz, ^4J = 1.6 Hz, 1H, H4), 7.39 (dd, ^3J = 7.7 Hz, ^3J = 4.9 Hz, 1H, H3), 7.22 (bs, 1H, NH), 4.68 – 4.60 (m, 1H, H7), 2.18 – 2.08 (m, 1H, H_a1'), 1.80 – 1.66 (m, 1H, H_b1'), 1.55 – 1.43 (m, 1H, H_a2'), 1.41 – 1.21 (m, 3H, H_b2', H3'), 0.90 (t, ^3J = 7.1 Hz, 3H, H4').
^{13}C-NMR (101 MHz, $CDCl_3$, 300 K): δ [ppm] = 169.1 (C5), 167.3 (C7a), 153.0 (C2), 132.2 (C4), 125.7 (C4a), 123.3 (C3), 58.6 (C7), 32.9 (C1'), 27.7 (C2'), 22.7 (C3'), 14.0 (C4').
HRMS (ESI) *m/z* $[M+H]^+$ calculated for $[C_{11}H_{15}N_2O]^+$: 191.1179; found: 191.1177.
IR (film) $\tilde{\nu}_{max}/cm^{-1}$: 3175 (m, NH), 3101 (m, CH_{arom}), 2953 (m, CH_{aliph}), 2869 (m, CH_{aliph}), 1703 (s, C=O) 1612 (m, $C{=}C_{arom}$).

2-Chloro-7-isopentyl-6,7-dihydro-5*H*-pyrrolo[3,4-*b*]pyridin-5-one (*rac*-**3k**)

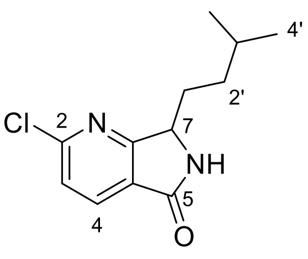

rac-**3k**
$C_{12}H_{15}ClN_2O$
M = 238.72 g mol^{-1}

According to **GP E**, a 1 M solution of KHMDS in THF (2.16 mL, 432 mg, 2.16 mmol, 1.10 equiv.) was added dropwise to a solution of 2-chloro-6-(4-methoxybenzyl)-6,7-dihydro-5*H*-pyrrolo[3,4-*b*]pyridin-5-one (**SI-3d**) (569 mg, 1.97 mmol, 1.00 equiv.) in anhydrous THF (25 mL) at –78 °C, upon which the reaction mixture turned light-red. After 20 minutes, 1-iodo-3-methylbutane (339 µL, 507 mg, 2.56 mmol, 1.30 equiv.) was added in one portion and the reaction was left to stir for two hours at room temperature. Subsequently, the solution was allowed to warm up to room temperature and quenched by the addition of sat. NH_4Cl (20 mL) and distilled water (20 mL). The aqueous layer was extracted with EtOAc (3 × 30 mL), and the combined organic phases were dried over Na_2SO_4, filtered and concentrated under reduced pressure. The crude product was then dissolved in MeCN:H_2O (3:1, 58 mL), and diammonium cerium(IV) nitrate (3.23 g, 5.90 mmol, 3.00 equiv.) was added to the solution at room temperature. The yellow solution was stirred for two hours at the same temperature. Subsequently, water (30 mL) was added, and the mixture was extracted with EtOAc (3 × 30 mL). The combined organic layers were washed with brine (50 mL) and dried over

Na_2SO_4. After filtration, the solvent was removed under reduced pressure and the residual crude product was subjected to FCC (SiO_2, Hex:EtOAc = 2:1 → 1:1) to yield the desired racemic substrate 2-chloro-7-isopentyl-6,7-dihydro-5*H*-pyrrolo[3,4-*b*]pyridin-5-one (*rac*-**3k**) (132 mg, 553 µmol, 28%) as a white solid.

TLC (Hex:EtOAc = 2:1): R_f = 0.16 [UV] [$KMnO_4$].
M.p.: 130 °C.
^{1}H-NMR (400 MHz, $CDCl_3$, 300 K): δ [ppm] = 8.05 (d, ^3J = 8.0 Hz, 1H, H4), 7.97 (bs, 1H, NH), 7.41 (d, ^3J = 8.0 Hz, 1H, C3), 4.59 (dd, ^3J = 8.1 Hz, ^3J = 4.1 Hz, 1H), 2.21 – 2.08 (m, 1H, H_a1'), 1.73 – 1.61 (m, 1H, H_b1'), 1.60 – 1.50 (m, 1H, H3'), 1.43 – 1.32 (m, 1H, H_a2'), 1.26 – 1.13 (m, 1H, H_b2'), 0.87 (*virt. t*, ^3J = 6.9 Hz, 6H, H4').
^{13}C-NMR (101 MHz, $CDCl_3$, 300 K): δ [ppm] = 168.4 (C5), 168.2 (C7a), 155.3 (C2), 134.4 (C4), 124.7 (C4a), 124.1 (C3), 58.7 (C7), 34.5 (C2'), 30.9 (C1'), 28.1 (C3'), 22.6 (C4'), 22.4 (C4').
HRMS (**ESI**) *m/z* $[M+H]^+$ calculated for $[C_{12}H_{16}{}^{35}ClN_2O]^+$: 239.0946; found: 239.0943.
IR (film): $\tilde{v}_{max}/cm^{-1}$ = 3188 (m, NH), 3088 (m, CH_{arom}), 2956 (m, CH_{aliph}), 2924 (m, CH_{aliph}), 1686 (s, C=O) 1572 (m, $C{=}C_{arom}$), 1097 (m, C-Cl).

7-Benzyl-2-bromo-6,7-dihydro-5*H*-pyrrolo[3,4-*b*]pyridin-5-one (*rac*-**3l**)

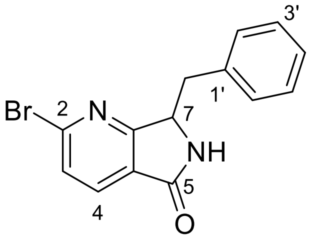

rac-**3l**
$C_{14}H_{11}BrN_2O$
M = 303.16 g mol^{-1}

According to **GP D**, a 1 M solution of KHMDS in THF (2.16 mL, 432 mg, 2.16 mmol, 1.10 equiv.) was added dropwise to a solution of 2-bromo-6-(4-methoxybenzyl)-6,7-dihydro-5*H*-pyrrolo[3,4-*b*]pyridin-5-one (**SI-3b**) (656 mg, 1.97 mmol, 1.00 equiv.) in anhydrous THF (25 mL) at –78 °C, upon which the reaction mixture turned dark red. After 20 minutes, benzyl bromide (304 µL, 437 mg, 2.56 mmol, 1.30 equiv.) was added in one portion. The reaction was stirred for two hours at –78 °C. Subsequently, the solution was allowed to warm up to room temperature and quenched by the addition of sat. NH_4Cl (20 mL) and distilled water (20 mL). The aqueous layer was extracted with EtOAc (3 × 30 mL), and the combined organic phases were dried over Na_2SO_4, filtered and concentrated under reduced pressure. The crude product was then dissolved in MeCN:H_2O (3:1, 58 mL), and diammonium cerium(IV) nitrate (3.23 g, 5.90 mmol, 3.00 equiv.) was added to the solution at room temperature. The yellow solution was stirred for two hours at the same temperature. Subsequently, water (30 mL) was added, and the mixture was extracted with EtOAc

(3 × 30 mL). The combined organic layers were washed with brine (50 mL) and dried over Na_2SO_4. After filtration, the solvent was removed under reduced pressure and the residual crude product was subjected to FCC (SiO_2, Hex:EtOAc = 2:1 → 1:2) to yield the desired racemic substrate 7-benzyl-2-bromo-6,7-dihydro-5*H*-pyrrolo[3,4-*b*]pyridin-5-one (*rac*-**3l**) (321 mg, 1.06 mmol, 54%) as an off-white solid.

TLC (Hex:EtOAc = 1:1): R_f = 0.27 [UV] [$KMnO_4$].

M.p.: 180 °C.

^{1}H-NMR (400 MHz, $CDCl_3$, 300 K): δ [ppm] = 7.93 (d, ^3J = 8.0 Hz, 1H, H4), 7.62 (d, ^3J = 8.0 Hz, 1H), 7.35 – 7.25 (m, 3H, H2', H4', H6'), 7.23 – 7.17 (m, 2H, H3', H5'), 6.60 (bs, 1H, NH), 4.82 (dd, ^3J = 9.7 Hz, ^3J = 3.5 Hz, 1H, H7), 3.64 (dd, ^2J = 13.8, ^3J = 3.5 Hz, 1H, C7-CH_a-C1'), 2.73 (dd, ^3J = 13.8, ^3J = 9.7 Hz, 1H, C7-CH_b-C1').

^{13}C-NMR (101 MHz, $CDCl_3$, 300 K): δ [ppm] = 167.5 (C5), 167.4 (C7a), 146.5 (C2), 136.4 (C1'), 134.3 (C4), 129.3 (C2', C6'), 129.0 (C3', C5'), 128.2 (C3), 127.4 (C4'), 125.1 (C4a), 59.5 (C7), 39.5 (C7-CH_2-C1').

HRMS (**ESI**) *m/z* $[M+H]^+$ calculated for $[C_{14}H_{12}{}^{79}BrN_2O]^+$: 303.0128; found: 303.0121.

IR (film): $\tilde{v}_{max}/cm^{-1}$ = 3148 (m, NH), 3108 (m, CH_{arom}), 2930 (m, CH_{aliph}), 1698 (s, C=O) 1656 (m, $C{=}C_{arom}$), 1574 (m, $C{=}C_{arom}$), 1090 (m, C-Br).

7-Benzyl-2-(trifluoromethyl)-6,7-dihydro-5*H*-pyrrolo[3,4-*b*]pyridin-5-one (*rac*-**3m**)

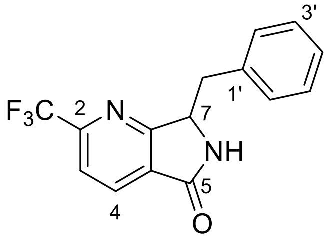

rac-**3m**

$C_{15}H_{11}F_3N_2O$

M = 292.26 g mol^{-1}

According to **GP D**, a 1 M solution of KHMDS in THF (2.16 mL, 432 mg, 2.16 mmol, 1.10 equiv.) was added dropwise to a solution of 6-(4-methoxybenzyl)-2-(trifluoromethyl)-6,7-dihydro-5*H*-pyrrolo[3,4-*b*]pyridin-5-one (**SI-3c**) (634 mg, 1.97 mmol, 1.00 equiv.) in anhydrous THF (25 mL) at –78 °C, upon which the reaction mixture turned violet-blue. After 20 minutes, benzyl bromide (304 µL, 437 mg, 2.56 mmol, 1.30 equiv.) was added in one portion. The reaction was stirred for two hours at –78 °C. Subsequently, the solution was allowed to warm up to room temperature and quenched by the addition of sat. NH_4Cl (20 mL) and distilled water (20 mL). The aqueous layer was extracted with EtOAc (3 × 30 mL), and the combined organic phases were dried over Na_2SO_4, filtered and concentrated under reduced pressure. The crude product was then dissolved in MeCN:H_2O (3:1, 58 mL), and diammonium cerium(IV) nitrate (3.23 g, 5.90 mmol, 3.00 equiv.) was added to the solution at room temperature. The yellow solution was stirred for two hours at the same

temperature. Subsequently, water (30 mL) was added, and the mixture was extracted with EtOAc (3 × 30 mL). The combined organic layers were washed with brine (50 mL) and dried over Na_2SO_4. After filtration, the solvent was removed under reduced pressure and the residual crude product was subjected to FCC (SiO_2, Hex:EtOAc = 2:1 → 1:1) to yield the desired racemic substrate 7-benzyl-2-(trifluoromethyl)-6,7-dihydro-5*H*-pyrrolo[3,4-*b*]pyridin-5-one (*rac*-**3m**) (367 mg, 1.26 mmol, 64%) as a white solid.

TLC (Hex:EtOAc = 1:1): R_f = 0.43 [UV] [$KMnO_4$].
M.p.: 139 °C.
^{1}H-NMR (400 MHz, $CDCl_3$, 300 K): δ [ppm] = 8.25 (d, ^3J = 7.9 Hz, 1H, H4), 7.81 (d, ^3J = 7.9 Hz, 1H, H3), 7.30 – 7.21 (m, 3H, H2', H4', H6'), 7.20 – 7.15 (m, 2H, H3', H5'), 6.95 (bs, 1H, NH), 4.98 – 4.90 (dd, ^3J = 9.2 Hz, ^3J = 3.6 Hz, 1H, H7), 3.64 (dd, ^2J = 13.8 Hz, ^3J = 3.6 Hz, 1H, C7-CH_a-C1'), 2.82 (dd, ^2J = 13.8 Hz, ^3J = 9.2 Hz, 1H, C7-CH_b-C1').
^{13}C-NMR (101 MHz, $CDCl_3$, 300 K): δ [ppm] = 166.9 (C5), 166.5 (C7a), 151.2 (q, $^2J_{C\text{-}F}$ = 35.1 Hz, C2), 136.1 (C1'), 133.9 (C4), 129.4 (C2', C6'), 129.0 (C3', C5'), 128.6 (C4a), 127.4 (C4'), 121.4 (q, $^1J_{C\text{-}F}$ = 274.4 Hz, CF_3), 120.5 (q, $^3J_{C\text{-}F}$ = 3.0 Hz, C3), 59.7 (C7), 39.3 (C7-CH_2-C1').
^{19}F-NMR (376MHz, $CDCl_3$, 300 K): δ[ppm] = −67.5.
HRMS (**ESI**) *m/z* $[M+H]^+$ calculated for $[C_{15}H_{12}F_3N_2O]^+$: 293.0902; found: 293.0908.
IR (film): $\tilde{v}_{max}/cm^{-1}$ = 3242 (m, NH), 3034 (m, CH_{arom}), 2932 (m, CH_{aliph}), 1663 (s, C=O) 1346 (m, $C{=}C_{arom}$), 1130 (m, CN).

7-Benzyl-2-chloro-6,7-dihydro-5*H*-pyrrolo[3,4-*b*]pyridin-5-one (*rac*-**3n**)

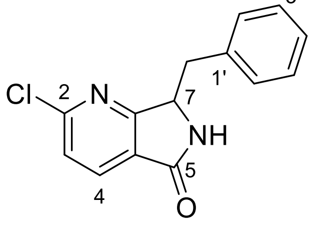

rac-**3n**
$C_{14}H_{11}ClN_2O$
M = 258.71 g mol^{-1}

According to **GP D**, a 1 M solution of KHMDS in THF (2.16 mL, 432 mg, 2.16 mmol, 1.10 equiv.) was added dropwise to a solution of 2-chloro-6-(4-methoxybenzyl)-6,7-dihydro-5*H*-pyrrolo[3,4-*b*]pyridin-5-one (**SI-3d**) (569 mg, 1.97 mmol, 1.00 equiv.) in anhydrous THF (25 mL) at –78 °C, upon which the reaction mixture turned light-red. After 20 minutes, benzyl bromide (304 µL, 437 mg, 2.56 mmol, 1.30 equiv.) was added in one portion. The reaction was stirred for two hours at –78 °C. Subsequently, the solution was allowed to warm up to room temperature and quenched by the addition of sat. NH_4Cl (20 mL) and distilled water (20 mL). The aqueous layer was extracted with EtOAc (3 × 30 mL), and the combined organic phases were dried over Na_2SO_4, filtered and concentrated under reduced pressure. The crude product

was then dissolved in MeCN:H_2O (3:1, 58 mL), and diammonium cerium(IV) nitrate (3.23 g, 5.90 mmol, 3.00 equiv.) was added to the solution at room temperature. The yellow solution was stirred for two hours at the same temperature. Subsequently, water (30 mL) was added, and the mixture was extracted with EtOAc (3 × 30 mL). The combined organic layers were washed with brine (50 mL) and dried over Na_2SO_4. After filtration, the solvent was removed under reduced pressure and the residual crude product was subjected to FCC (SiO_2, Hex:EtOAc = 1:1) to yield the desired racemic substrate 7-benzyl-2-chloro-6,7-dihydro-5*H*-pyrrolo[3,4-*b*]pyridin-5-one (*rac*-**3n**) (210 mg, 812 µmol, 41%) as a white solid.

TLC (Hex:EtOAc = 1:1): R_f = 0.23 [UV] [$KMnO_4$].
M.p.: 165 °C.
^{1}H-NMR (400 MHz, $CDCl_3$, 300 K): δ [ppm] = 8.03 (d, ^3J = 8.1 Hz, 1H, H4), 7.45 (d, ^3J = 8.0 Hz, 1H, H3), 7.34 – 7.23 (m, 3H, H2', H4', H6'), 7.22 – 7.18 (m, 2H, H3', H5'), 6.67 (bs, 1H, NH), 4.80 (dd, ^3J = 9.6 Hz, ^3J = 3.5 Hz, 1H, H7), 3.62 (dd, ^2J = 13.7, ^3J = 3.5 Hz, 1H, C7-CH_a-C1'), 2.72 (dd, ^3J = 13.7 Hz, ^3J = 9.6 Hz, 1H, C7-CH_b-C1').
^{13}C-NMR (101 MHz, $CDCl_3$, 300 K): δ [ppm] = 167.3 (C5), 167.1 (C7a), 155.4 (C2), 136.4 (C1'), 134.7 (C4), 129.3 (C3', C5'), 129.0 (C2', C6'), 127.4 (C4'), 124.8 (C4a), 124.5 (C3), 59.5 (C7), 39.5 (C7-CH_2-C1').
HRMS (**ESI**) *m/z* $[M+H]^+$ calculated for $[C_{14}H_{12}{}^{35}ClN_2O]^+$: 259.0633; found: 259.0631.
IR (film): $\tilde{v}_{max}/cm^{-1}$ = 3182 (m, NH), 3088 (m, CH_{arom}), 2915 (m, CH_{aliph}), 1683 (s, C=O) 1596 (m, $C{=}C_{arom}$), 1090 (m, C-Cl).

7-Benzyl-3-bromo-6,7-dihydro-5*H*-pyrrolo[3,4-*b*]pyridin-5-one (*rac*-**3o**)

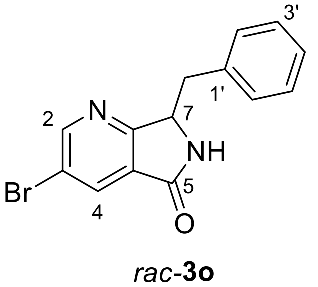

rac-**3o**
$C_{14}H_{11}BrN_2O$
M = 303.16 g mol^{-1}

According to **GP D**, a 1 M solution of KHMDS in THF (2.16 mL, 432 mg, 2.16 mmol, 1.10 equiv.) was added dropwise to a solution of 3-bromo-6-(4-methoxybenzyl)-6,7-dihydro-5*H*-pyrrolo[3,4-*b*]pyridin-5-one (**SI-3e**) (656 mg, 1.97 mmol, 1.00 equiv.) in anhydrous THF (25 mL) at –78 °C, upon which the reaction mixture turned dark red. After 20 minutes, benzyl bromide (304 µL, 437 mg, 2.56 mmol, 1.30 equiv.) was added in one portion. The reaction was stirred for two hours at –78 °C. Subsequently, the solution was allowed to warm up to room temperature and quenched by the addition of sat. NH_4Cl (20 mL) and distilled water (20 mL). The aqueous layer was extracted with EtOAc (3 × 30 mL), and the combined organic phases were dried over Na_2SO_4, filtered and concentrated under reduced pressure. The crude product

was then dissolved in MeCN:H_2O (3:1, 58 mL), and diammonium cerium(IV) nitrate (3.23 g, 5.90 mmol, 3.00 equiv.) was added to the solution at room temperature. The yellow solution was stirred for two hours at the same temperature. Subsequently, water (30 mL) was added, and the mixture was extracted with EtOAc (3 × 30 mL). The combined organic layers were washed with brine (50 mL) and dried over Na_2SO_4. After filtration, the solvent was removed under reduced pressure and the residual crude product was subjected to FCC (SiO_2, Hex:EtOAc = 1:1 → 1:2) to yield the desired racemic substrate 7-benzyl-3-bromo-6,7-dihydro-5*H*-pyrrolo[3,4-*b*]pyridin-5-one (*rac*-**3o**) (281 mg, 927 µmol, 47%) as a white solid.

TLC (Hex:EtOAc = 1:1): R_f = 0.22 [UV] [$KMnO_4$].
M.p.: 212 °C.
^{1}H-NMR (400 MHz, $CDCl_3$, 300 K): δ [ppm] = 8.84 (d, ^4J = 2.2 Hz, 1H, H2), 8.20 (d, ^4J = 2.2 Hz, 1H, H4), 7.34 – 7.26 (m, 3H, H2', H4', H6')), 7.23 – 7.17 (m, 2H, H3', H5'), 6.49 (bs, 1H, NH), 4.79 (ddd, ^3J = 9.7 Hz, ^3J = 3.7 Hz, ^3J = 1.2 Hz, 1H, H7), 3.59 (dd, ^3J = 13.8 Hz, ^3J = 3.6 Hz, 1H, C7-CH_a-C1'), 2.71 (dd, ^3J = 13.8 Hz, ^3J = 9.6 Hz, 1H, C7-CH_b-C1').
^{13}C-NMR (101 MHz, $CDCl_3$, 300 K): δ [ppm] = 166.8 (C5), 164.4 (C7a), 154.1 (C2), 136.5 (C1'), 134.9 (C4), 129.3 (C3', C5'), 129.1 (C2', C6'), 127.5 (C4'), 127.4 (C4a), 120.9 (C3), 59.5 (C7), 39.6 (C7-CH_2-C1').
HRMS (**ESI**) *m/z* $[M+H]^+$ calculated for $[C_{14}H_{12}{}^{79}BrN_2O]^+$: 303.0128; found: 303.0126.
IR (film): $\tilde{v}_{max}/cm^{-1}$ = 3210 (m, NH), 3086 (m, CH_{arom}), 2927 (m, CH_{aliph}), 1705 (s, C=O) 1446 (m, $C{=}C_{arom}$), 1064 (m, C-Br).

7-Benzyl-3-chloro-6,7-dihydro-5*H*-pyrrolo[3,4-*b*]pyridin-5-one (*rac*-**3p**)

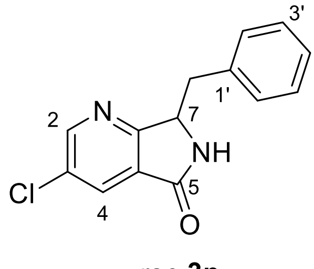

rac-**3p**
$C_{14}H_{11}ClN_2O$
M = 258.71 g mol^{-1}

According to **GP D**, a 1 M solution of KHMDS in THF (2.16 mL, 432 mg, 2.16 mmol, 1.10 equiv.) was added dropwise to a solution of 3-chloro-6-(4-methoxybenzyl)-6,7-dihydro-5*H*-pyrrolo[3,4-*b*]pyridin-5-one (**SI-3f**) (569 mg, 1.97 mmol, 1.00 equiv.) in anhydrous THF (25 mL) at –78 °C, upon which the reaction mixture turned dark-red. After 20 minutes, benzyl bromide (304 µL, 437 mg, 2.56 mmol, 1.30 equiv.) was added in one portion. The reaction was stirred for two hours at –78 °C. Subsequently, the solution was allowed to warm up to room temperature and quenched by the addition of sat. NH_4Cl (20 mL) and distilled water (20 mL). The aqueous layer was extracted with EtOAc (3 × 30 mL), and the combined organic phases were dried over Na_2SO_4, filtered and concentrated under reduced pressure. The crude product

was then dissolved in MeCN:H_2O (3:1, 58 mL), and diammonium cerium(IV) nitrate (3.23 g, 5.90 mmol, 3.00 equiv.) was added to the solution at room temperature. The yellow solution was stirred for two hours at the same temperature. Subsequently, water (30 mL) was added, and the mixture was extracted with EtOAc (3 × 30 mL). The combined organic layers were washed with brine (50 mL) and dried over Na_2SO_4. After filtration, the solvent was removed under reduced pressure and the residual crude product was subjected to FCC (SiO_2, Hex:EtOAc = 1:1) to yield the desired racemic substrate 7-benzyl-3-chloro-6,7-dihydro-5*H*-pyrrolo[3,4-*b*]pyridin-5-one (*rac*-**3p**) (160 mg, 619 µmol, 31%) as a white solid.

TLC (Hex:EtOAc = 1:1): R_f = 0.24 [UV] [$KMnO_4$].
M.p.: 205 °C.
^{1}H-NMR (400 MHz, $CDCl_3$, 300 K): δ [ppm] = 8.75 (d, ^4J = 2.3 Hz, 1H, H2), 8.05 (d, ^4J = 2.3 Hz, 1H, H4), 7.34 – 7.24 (m, 3H, H2', H4', H6'), 7.22 – 7.16 (m, 2H, H3', H5'), 6.80 (bs, 1H, NH), 4.84 (ddd, ^3J = 9.4 Hz, ^3J = 3.7 Hz, ^3J = 1.2 Hz, 1H, H7), 3.59 (dd, ^3J = 13.8 Hz, ^3J = 3.7 Hz, 1H, C7-CH_a-C1'), 2.76 (dd, ^3J = 13.8 Hz, ^3J = 9.4 Hz, 1H, C7-CH_b-C1').
^{13}C-NMR (101 MHz, $CDCl_3$, 300 K): δ [ppm] = 167.0 (C5), 164.1 (C7a), 152.0 (C2), 136.4 (C1'), 132.5 (C3), 131.9 (C4), 129.3 (C3', C5'), 129.0 (C2', C6'), 127.4 (C4'), 127.0 (C4a), 59.4 (C7), 39.5 (C7-CH_2-C1').
HRMS (**ESI**) *m/z* $[M+H]^+$ calculated for $[C_{14}H_{12}{}^{35}ClN_2O]^+$: 259.0633; found: 259.0631.
IR (film): $\tilde{v}_{max}/cm^{-1}$ = 3210 (m, NH), 3062 (m, CH_{arom}), 2929 (m, CH_{aliph}), 1705 (s, C=O) 1452 (m, $C=C_{arom}$), 1097 (m, C-Cl).

7-Benzyl-3-phenyl-6,7-dihydro-5*H*-pyrrolo[3,4-*b*]pyridin-5-one (*rac*-**3q**)

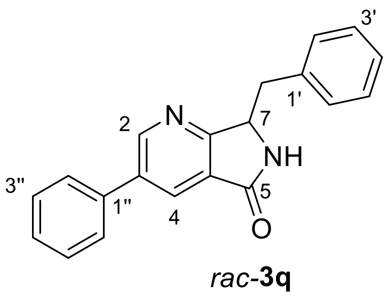

rac-**3q**
$C_{20}H_{16}N_2O$
M = 300.36 g mol^{-1}

According to **GP H**, to a solution of 7-benzyl-3-bromo-6,7-dihydro-5*H*-pyrrolo[3,4-*b*]pyridin-5-one (*rac*-**3o**) (100 mg, 330 µmol, 1.00 equiv.) in THF/water (3:1) (8 mL) was added phenylboronic acid (60.3 mg, 495 µmol, 1.50 equiv.), K_2CO_3 (91.2 mg, 660 µmol, 2.00 equiv.), $PdCl_2(PPh_3)_2$ (23.2 mg, 33.0µmol, 10 mol%) and PCy_3 (18.5 mg, 66.0µmol, 20 mol%). The resulting mixture was degassed by freeze-pump thaw cycling thrice and then stirred at 60 °C for 16 hours. Afterwards, the mixture was allowed to cool to room temperature water (10 mL) was added and the aqueous layer was extracted with EtOAc (3 × 20 mL).). The combined organic layers were washed with brine (20 mL) and dried over Na_2SO_4. After filtration, the solvent was removed under reduced

pressure and the residual crude product was subjected to FCC (SiO_2, Hex/EtOAc) to yield the desired racemic substrate **7**-benzyl-3-phenyl-6,7-dihydro-5*H*-pyrrolo[3,4-*b*]pyridin-5-one *rac*-**3q** (82.0 mg, 273 µmol, 83%) as a white solid.

TLC (Hex:EtOAc = 1:1): R_f = 0.20 [UV] [$KMnO_4$].

M.p.: 218 °C.

^{1}H-NMR (400 MHz, $CDCl_3$, 300 K): δ [ppm] = 9.01 (d, ^4J = 2.2 Hz, 1H, H2), 8.28 (d, ^4J = 2.2 Hz, 1H), 7.66 – 7.59 (m, 2H, H2'', H6''), 7.58 – 7.49 (m, 2H, H3'', H5''), 7.50 – 7.41 (m, 1H, H4''), 7.37 – 7.30 (m, 2H, H3', H5'), 7.29 – 7.23 (m, 3H, H2', H4', H6'), 6.48 (bs, 1H, NH), 4.87 (ddd, ^3J = 9.9 Hz, ^3J = 3.6 Hz, ^3J = 1.2 Hz, 1H, H7), 3.68 (dd, ^3J = 13.8 Hz, ^3J = 3.6 Hz, 1H, C7-CH_a-C1'), 2.74 (dd, ^3J = 13.8, ^3J = 9.9 Hz, 1H, C7-CH_b-C1').

^{13}C-NMR (101 MHz, $CDCl_3$, 300 K): δ [ppm] = 168.3 (C5), 165.0 (C7a), 151.9 (C2), 137.3 (C3/C1'/C1''), 137.1 (C3/C1'/C1''), 137.0 (C3/C1'/C1''), 130.4 (C4), 129.5 (C3'', C5''), 129.3 (C2', C6'), 129.1 (C3', C5'), 128.8 (C4''), 127.5 (C2'', C6''), 127.4 (C4'), 125.9 (C4a), 59.7 (C7), 39.9 (C7-CH_2-C1').

HRMS (**ESI**) *m/z* $[M+H]^+$ calculated for $[C_{20}H_{17}N_2O]^+$: 301.1335; found: 301.1332.

IR (film): $\tilde{v}_{max}/cm^{-1}$ = 3171 (m, NH), 3047 (m, CH_{arom}), 3028 (m, CH_{arom}), 2931 (m, CH_{aliph}), 1698 (s, C=O), 1573 (m, $C=C_{arom}$), 1406 (m, $C=C_{arom}$).

7-Hydroxy-6,7-dihydro-5H-pyrrolo[3,4-b]pyridin-5-one (**SI-4**)

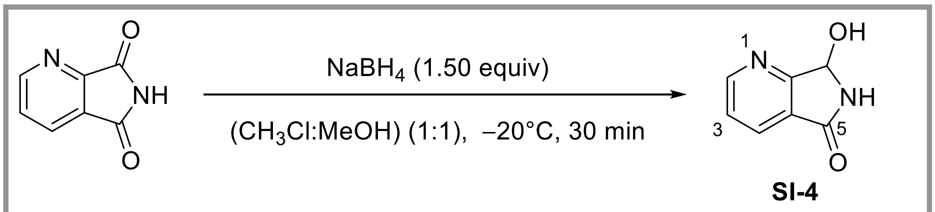

According to a literature known procedure,[19] sodium borohydride (1.34 g, 35.4 mmol, 1.50 equiv.) was added portion wise to a solution of 5*H*-pyrrolo[3,4-*b*]pyridine-5,7(6*H*)-dione (3.50 g, 23.6 mmol, 1.00 equiv.) in CH_3Cl:MeOH (1:1, 236 mL) at –20°C. The mixture was stirred for 30 minutes at the same temperature. Afterwards, the reaction was quenched by addition of 1 M HCl until the pH reached a value of approximately 2. The mixture was stirred for 10 minutes at this pH value at –20 °C. Subsequently, 1 M NaOH was added until the pH reached a value of approximately 9. Subsequently, the solvents were removed under reduced pressure and the crude reaction mixture was directly subjected to FCC (SiO_2, CH_3Cl:MeOH = 10:1 → 5:1) to yield 7-hydroxy-6,7-dihydro-5*H*-pyrrolo[3,4-*b*]pyridin-5-one

(**SI-4**) (2.10 g, 14.0 mmol, 59%) as a white solid. The product was obtained as an inseparable mixture of two regioisomers with a ratio of 9:1 in favor of the wanted regioisomer and was used without further purification.

TLC (CH_3Cl:MeOH = 5:1): R_f = 0.33 [UV] [$KMnO_4$].
^{1}H-NMR (400 MHz, DMSO-d6, 300 K): δ [ppm] = 9.56 (s, 1H, NH), 9.00 (m, 2H, H2, H3), 6.66 (d, ^3J = 9.2 Hz, 1H, OH), 5.91 (d, ^3J = 9.2 Hz, 1H, H7).

The analytical data is in accordance with the literature.[19]

7-Isopropoxy-6,7-dihydro-5*H*-pyrrolo[3,4-*b*]pyridin-5-one (*rac*-**3r**)

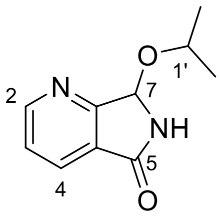

rac-**3r**
$C_{10}H_{12}N_2O_2$
M = 192.22 g mol^{-1}

According to **GP F**, *p*-TsOH (38.0 mg, 200 µmol, 0.10 equiv.) was added to a suspension of 7-hydroxy-6,7-dihydro-5*H*-pyrrolo[3,4-*b*]pyrazin-5-one (**SI-4**) (300 mg, 2.00 mmol 1.00 equiv.) in propan-2-ol (3.06 mL, 2.40 g, 40.0 mmol, 20.0 equiv.). The resulting mixture was then stirred at 80 °C for two hours. Afterwards, the mixture was allowed to cool to room temperature and was quenched by addition of a sat. bicarb solution (10 mL). The mixture was extracted with EtOAc (3 × 30 mL), and the combined organic layers were washed with brine (20 mL) and dried over Na_2SO_4. After filtration, the solvent was removed under reduced pressure and the residual crude product was subjected to FCC (SiO_2, Hex:EtOAc = 1:2 → 0:1) to yield the desired racemic substrate 7-isopropoxy-6,7-dihydro-5*H*-pyrrolo[3,4-*b*]pyridin-5-one (*rac*-**3r**) (202 mg, 1.05 mmol, 53%) as a white solid.

TLC (EtOAc): R_f = 0.23 [UV] [$KMnO_4$].
M.p.: 176 °C.
^{1}H-NMR (400 MHz, $CDCl_3$, 300 K): δ [ppm] = 8.80 (dd, ^3J = 4.9, ^3J = 1.6 Hz, 1H, H2), 8.10 (dd, ^3J = 7.7, 1.6 Hz, 1H, H4), 7.63 (bs, 1H, NH), 7.44 (dd, ^3J = 7.7, ^3J = 4.9 Hz, 1H, H3), 5.90 (d, ^3J = 1.3 Hz, 1H, H7), 4.07 (*virt.* hept, ^3J = 6.2 Hz, 1H, H1'), 1.33 (d, ^3J = 6.1 Hz, 3H, H_a2'), 1.26 (d, ^3J = 6.1 Hz, 3H, H_b2').
^{13}C-NMR (101 MHz, $CDCl_3$, 300 K): δ [ppm] = 168.4 (C5), 163.8 (C7a), 153.6 (C2), 132.1 (C4), 125.8 (C4a), 124.6 (C3), 83.3 (C7), 71.4 (C1'), 23.2 (C_a2'), 22.9 (C_b2').
HRMS (**ESI**) *m/z* [M+H]$^+$ calculated for $[C_{10}H_{13}N_2O_2]^+$: 193.0972; found: 193.0971.

IR (film): $\tilde{\nu}_{max}$/cm^{-1} = 3184 (m, NH), 3047 (m, CH_{arom}), 2979 (m, CH_{aliph}), 2921 (m, CH_{aliph}), 1677 (s, C=O), 1585 (m, $C=C_{arom}$), 1413 (m, $C=C_{arom}$), 1192 (m, C-O).

7-(Cyclopentyloxy)-6,7-dihydro-5*H*-pyrrolo[3,4-*b*]pyridin-5-one (*rac*-**3s**)

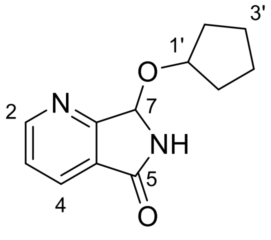

rac-**3s**
$C_{12}H_{14}N_2O_2$
M = 218.26 g mol^{-1}

According to **GP F**, *p*-TsOH (38.0 mg, 200 µmol, 0.10 equiv.)was added to a suspension of 7-hydroxy-6,7-dihydro-5*H*-pyrrolo[3,4-*b*]pyrazin-5-one (**SI-4**) (300 mg, 2.00 mmol 1.00 equiv.) in cyclopentanol (3.62 mL, 3.44 g, 40.0 mmol, 20.0 equiv.). The resulting mixture was then stirred at 80 °C for two hours. Afterwards, the mixture was allowed to cool to room temperature and was quenched by addition of a sat. bicarb solution (10 mL). The mixture was extracted with EtOAc (3 × 30 mL), and the combined organic layers were washed with brine (20 mL) and dried over Na_2SO_4. After filtration, the solvent was removed under reduced pressure and the residual crude product was subjected to FCC (SiO_2, Hex:EtOAc = 1:2) to yield the desired racemic substrate 7-(cyclopentyloxy)-6,7-dihydro-5*H*-pyrrolo[3,4-*b*]pyridin-5-one (*rac*-**3s**)
(244 mg, 1.12 mmol, 56%) as a white solid.

TLC (EtOAc): R_f = 0.41 [UV] [$KMnO_4$].
M.p.: 169 °C.
^{1}H-NMR (400 MHz, $CDCl_3$, 300 K): δ [ppm] = 8.81 (dd, ^3J = 5.0 Hz, ^4J = 1.6 Hz, 1H, H2), 8.11 (dd, ^3J = 7.7 Hz, ^4J = 1.6 Hz, 1H, H4), 7.45 (dd, ^3J = 7.7 Hz, ^3J = 5.0 Hz, 1H, H3), 6.84 (bs, 1H, NH), 5.88 (d, ^3J = 1.3 Hz, 1H, H7), 4.31 (tt, ^3J = 5.9 Hz, ^3J = 3.9 Hz, 1H, H1'), 1.92 – 1.82 (m, 2H, H_a2', H_a5'), 1.81 – 1.66 (m, 4H, H_b2', H_b5', H_a3', H_a4'), 1.61 – 1.48 (m, 2H, H_b3', H_b4').
^{13}C-NMR (101 MHz, $CDCl_3$, 300 K): δ [ppm] = 167.8 (C5), 163.7 (C7a), 153.6 (C2), 132.2 (C4), 125.9 (C4a), 124.7 (C3), 83.9 (C7), 80.8 (C1', 33.3 (C2'/C5'), 33,4 (C2'/C5'), 23.6 (C3'/C4'), 23.5 (C3'/C4').
HRMS (**ESI**) *m/z* $[M+H]^+$ calculated for $[C_{12}H_{15}N_2O_2]^+$: 219.1128; found: 219.1125.
IR (film): $\tilde{\nu}_{max}$/cm^{-1} = 3212 (m, NH), 3091 (m, CH_{arom}), 2953 (m, CH_{aliph}), 2917(m, CH_{aliph}), 1706 (s, C=O), 1589 (m, $C=C_{arom}$), 1416 (m, $C=C_{arom}$), 1105 (m, C-O).

7-(*tert*-Butoxy)-6,7-dihydro-5*H*-pyrrolo[3,4-*b*]pyridin-5-one (*rac*-**3t**)

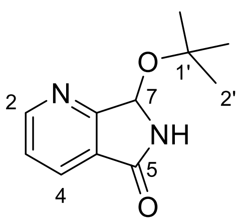

rac-**3t**

$C_{11}H_{14}N_2O_2$

M = 206.25 g mol^{-1}

According to a literature known procedure,[20] a suspension of 7-hydroxy-6,7-dihydro-5*H*-pyrrolo[3,4-*b*]pyrazin-5-one (**SI-4**) (300 mg, 2.00 mmol, 1.00 equiv.) in trifluoroacetic anhydride (TFAA) (1.00 ml, 1.51 g, 7.18 mmol, 3.60 equiv.) was stirred for 20 minutes at room temperature. Afterwards the excess TFAA was removed under reduced pressure and *tert*-butanol (4.00 mL, 3.20 g, 43.1 mmol, 21.6 equiv.) was added at room temperature. The resulting mixture was stirred for one hour at the same temperature. The reaction was quenched by addition of a sat. bicarb solution (10 mL), the mixture was extracted with CH_2Cl_2 (3 × 15 mL), and the combined organic layers were washed with brine (20 mL) and dried over Na_2SO_4. After filtration, the solvent was removed under reduced pressure and the residual crude product was subjected to FCC (SiO_2, EtOAc) to yield the desired racemic substrate 7-(*tert*-butoxy)-6,7-dihydro-5*H*-pyrrolo[3,4-*b*]pyridin-5-one (*rac*-**3t**) (168 mg, 815 µmol, 41%) as a white solid.

TLC (Hex:EtOAc = 1:2): R_f = 0.31 [UV] [$KMnO_4$].

^{1}H-NMR (400 MHz, $CDCl_3$, 300 K): δ [ppm] = 8.80 (dd, ^3J = 5.0 Hz, ^4J = 1.6 Hz, 1H, H2), 8.07 (dd, ^3J = 7.6 Hz, ^4J = 1.6 Hz, 1H, H4), 7.41 (dd, ^3J = 7.6 Hz, ^3J = 5.0 Hz, 1H, H3), 7.06 (bs, 1H, NH), 5.96 (d, ^3J = 1.1 Hz, 1H, H7), 1.45 (s, 9H, H2').

^{13}C-NMR (101 MHz, $CDCl_3$, 300 K): δ [ppm] = 168.2 (C5), 164.5 (C7a), 153.7 (C2), 131.9 (C4), 125.6 (C4a), 124.4 (C3), 79.5 (C7), 76.3 (C1'), 28.8 (C2').

The spectroscopic data matches those reported in literature.[20]

7-(3-Chloro-2,2-dimethylpropoxy)-6,7-dihydro-5*H*-pyrrolo[3,4-*b*]pyridin-5-one (*rac*-**3u**)

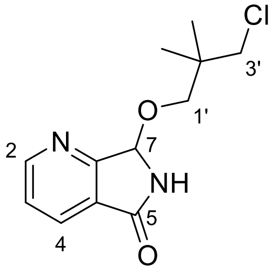

rac-**3u**

$C_{12}H_{15}ClN_2O_2$

M = 254.71 g mol^{-1}

According to **GP F**, *p*-TsOH (38.0 mg, 200 µmol, 0.10 equiv.)was added to a suspension of 7-hydroxy-6,7-dihydro-5*H*-pyrrolo[3,4-*b*]pyrazin-5-one (**SI-4**) (300 mg, 2.00 mmol 1.00 equiv.) in 3-chloro-2,2-dimethylpropan-1-ol (4.90 g, 40.0 mmol, 20.0 equiv.). The resulting mixture was then stirred at 100 °C for 30 minutes. Afterwards, the mixture was allowed to cool to room temperature and was quenched by addition of a sat. bicarb solution (10 mL). The mixture was extracted with EtOAc (3 × 30 mL), and the combined organic layers were washed with brine (20 mL) and dried over Na_2SO_4. After filtration, the solvent was removed under

reduced pressure and the residual crude product was subjected to FCC (SiO_2, Hex:EtOAc = 1:2) to yield the desired racemic substrate 7-(3-chloro-2,2-dimethylpropoxy)-6,7-dihydro-5*H*-pyrrolo[3,4-*b*]pyridin-5-one *rac*-**3u** (305 mg, 1.20 mmol, 60%) as a white solid.

TLC (Hex:EtOAc = 1:2): R_f = 0.23 [UV] [$KMnO_4$].

M.p.: 172 °C.

^{1}H-NMR (400 MHz, $CDCl_3$, 300 K): δ [ppm] = 8.81 (dd, ^3J = 4.9 Hz, ^4J = 1.6 Hz, 1H, H2), 8.12 (dd, ^3J = 7.7 Hz, ^4J = 1.6 Hz, 1H, H4), 7.46 (dd, ^3J = 7.7 Hz, ^3J = 5.0 Hz, 1H, H3), 7.24 (bs, 1H, NH), 5.93 (d, ^3J = 1.3 Hz, 1H, H7), 3.51 – 3.38 (m, 3H, H_a1', H3'), 3.16 (d, ^3J = 8.9 Hz, 1H, H_b1'), 0.97 (s, 3H, H_a4'), 0.96 (s, 3H, H_b4').

^{13}C-NMR (101 MHz, $CDCl_3$, 300 K): δ [ppm] = 168.0 (C5), 163.0 (C7a), 153.6 (C2), 132.1 (C4), 126.1 (C4a), 124.8 (C3), 84.5 (C7), 71.1 (C1'), 52.3 (C3'), 36.7 (C2'), 22.8 (C_a4'), 22.8 (C_b4').

HRMS (**ESI**) *m/z* $[M+H]^+$ calculated for $[C_{12}H_{16}{}^{35}ClN_2O_2]^+$: 255.0895; found: 255.0893.

IR (film): $\tilde{v}_{max}/cm^{-1}$ = 3190 (m, NH), 3120 (m, CH_{arom}), 2958 (m, CH_{aliph}), 2880 (m, CH_{aliph}), 1708 (s, C=O), 1673 (m, $C=C_{arom}$), 1415 (m, $C=C_{arom}$), 768 (m, C-Cl).

7-((*tert*-Butyldimethylsilyl)oxy)-6,7-dihydro-5*H*-pyrrolo[3,4-*b*]pyridin-5-one (*rac*-**3v**)

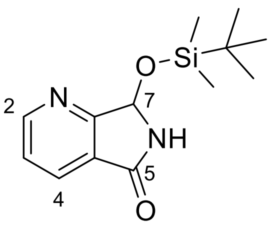

rac-**3v**
$C_{13}H_{20}N_2O_2Si$
M = 264.40 g mol^{-1}

According to **GP G**, imidazole (2.04 mg, 3.00 mmol, 1.50 equiv.) and *tert*-butylchlorodimethylsilane (602 mg, 4.00 mmol, 2.00 equiv.) were added to a solution of 7-hydroxy-6,7-dihydro-5*H*-pyrrolo[3,4-*b*]pyridin-5-one (**SI-4**) (300 mg, 2.00 mmol, 1.00 equiv.) in DMF (10 mL) at room temperature. The resulting mixture was stirred for 16 hours at the same temperature. Subsequently, the mixture was quenched by the addition of a sat. NH_4Cl solution (20mL). The mixture was extracted with CH_2Cl_2 (3 × 20 mL), and the combined organic layers were washed with brine (20 mL) and dried over Na_2SO_4. After filtration, the solvent was removed under reduced pressure and the residual crude product was subjected to FCC (SiO_2, Hex:EtOAc = 2:1 → 1:1) to yield the desired racemic substrate 7-((*tert*-butyldimethylsilyl)oxy)-6,7-dihydro-5*H*-pyrrolo[3,4-*b*]pyridin-5-one (*rac*-**3v**) (325 mg, 1.23 mmol, 62%) as a white solid.

TLC (Hex:EtOAc = 2:1): R_f = 0.31 [UV] [$KMnO_4$].

M.p.: 168 °C.

^{1}H-NMR (400 MHz, $CDCl_3$, 300 K): δ [ppm] = 8.78 (dd, ^3J = 4.9 Hz, ^4J = 1.6 Hz, 1H, H2), 8.08 (dd, ^3J = 7.6 Hz, ^4J = 1.6 Hz, 1H, H4), 7.42 (dd, ^3J = 7.6 Hz, ^3J = 4.9 Hz, 1H, H3), 6.70 (bs, 1H, NH), 6.07 (d, ^3J = 1.3 Hz, 1H, H7), 0.94 [s, 9H, $C(CH)_3$], 0.20 (s, 3H, CH_3), 0.17 (s, 3H, CH_3).

^{13}C-NMR (101 MHz, $CDCl_3$, 300 K): δ [ppm] = 167.8 (C5), 165.1 (C7a), 153.6 (C2), 132.0 (C4), 125.2 (C4a), 124.4 (C3), 79.8 (C7), 25.8 [C(*C*H)$_3$], 18.3 [*C*(CH)$_3$], –4.0 (CH_3), –4.3 (CH_3).

HRMS (**ESI**) *m/z* $[M+H]^+$ calculated for $[C_{12}H_{21}N_2O_2Si]^+$: 265.1367; found: 265.1365.

IR (film): $\tilde{v}_{max}/cm^{-1}$ = 3186 (m, NH), 3117 (m, CH_{arom}), 2929 (m, CH_{aliph}), 2858 (m, CH_{aliph}), 1708 (s, C=O), 1587 (m, $C{=}C_{arom}$), 1412 (m, $C{=}C_{arom}$), 1083 (m, C-O).

7-((*S*)-2-methylbutoxy)-6,7-dihydro-5*H*-pyrrolo[3,4-*b*]pyridin-5-one (*3S/3R*-**2w**)

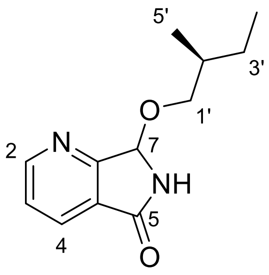

3S/3R-**3w**
$C_{12}H_{16}N_2O_2$
M = 220.27g mol^{-1}

According to **GP F**, *p*-TsOH (38.0 mg, 200 µmol, 0.10 equiv.) was added to a suspension of **SI-4** (300 mg, 2.00 mmol 1.00 equiv.) in (*S*)-2-methylbutan-1-ol (4.30 mL, 3.52 g, 40.0 mmol, 20.0 equiv.). The resulting mixture was then stirred at 100 °C for 30 minutes. Afterwards, the mixture was allowed to cool to room temperature and was quenched by addition of a sat. bicarb solution (10 mL). The mixture was extracted with EtOAc (3 × 30 mL), and the combined organic layers were washed with brine (20 mL) and dried over Na_2SO_4. After filtration, the solvent was removed under reduced pressure and the residual crude product was subjected to FCC (SiO_2, Hex:EtOAc = 1:2) to yield the desired racemic substrate 7-((*S*)-2-methylbutoxy)-6,7-dihydro-5*H*-pyrrolo[3,4-*b*]pyridin-5-one (*3S/3R*-**2w**) (304 mg, 1.38 mmol, 69%) as a white solid.

TLC (Hex:EtOAc = 1:2): R_f = 0.23 [UV] [$KMnO_4$].

M.p.: 119 °C (mixture of diastereoisomers).

^{1}H-NMR (400 MHz, $CDCl_3$, 300 K): δ [ppm] = 8.80 (dd, ^3J = 4.9 Hz, ^4J = 1.6 Hz, 1H, H2), 8.11 (dd, ^3J = 7.7, ^3J = 1.5 Hz, 1H, H4), 7.49 – 7.41 (m, 2H, H3, NH), 5.90 (s, 1H, H7), 3.55-3.14 (m, 2H, H1’), 1.74 – 1.58 (m, 1H, H2’), 1.52 – 1.36 (m, 1H, H_a3’), 1.21 – 1.04 (m, 1H, H_b3’), 0.92 – 0.87 (m, 3H, H5’), 0.87 – 0.80 (m, 3H, H4’).

^{13}C-NMR (101 MHz, $CDCl_3$, 300 K): δ [ppm] = 168.2 (C5), 163.4 (C7a), 153.6 (C2), 132.1 (C4), 126.1 (C4a), 124.7 (C3), 84.6 (C7), 84.6 (C7), 71.9 (C1’), 71.9 (C1’), 35.1 (C2’), 26.2 (C3’), 16.6 (C5’), 16.6 (C5’), 11.3 (C4’), 11.3 (C4’).

HRMS (**ESI**) *m/z* [M+H]$^+$ calculated for $[C_{12}H_{17}N_2O_2]^+$: 221.1285; found: 221.1282.

IR (film): $\tilde{v}_{max}/cm^{-1}$ = 3219 (m, NH), 3051 (m, CH_{arom}), 2962 (m, CH_{aliph}), 2876 (m, CH_{aliph}), 1709 (s, C=O), 1589 (m, $C=C_{arom}$), 1415 (m, $C=C_{arom}$), 1103 (m, C-O).

2-(4-Methoxybenzyl)-2,3-dihydro-1*H*-pyrrolo[3,4-*c*]pyridin-1-one (SI-17)

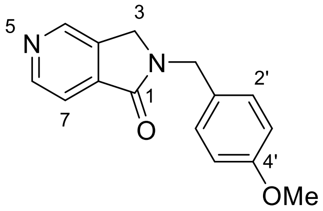

SI-17

$C_{15}H_{14}N_2O_2$

M = 254.29 g mol^{-1}

According to a modified procedure,[21] glacial acetic acid (946 µL, 993 mg, 16.5 mmol, 1.00 equiv.), 1-chloropyrrolidine-2,5-dione (NCS) (1.33 g, 9.92 mmol, 0.60 equiv.) and azobis(isobutyonitril) (AIBN) (543 mg, 3.31 mmol, 0.20 equiv.) were added to a solution of methyl 3-methylpyridine-4-carboxylate (2.50 g, 16.5 mmol, 1.00 equiv.) in DCE (83 mL). The resulting mixture was stirred for five hours at 60 °C before AIBN (543 mg, 3.31 mmol, 0.20 equiv.) was added again. This process was repeated after nine hours, 24 hours, 28 hours, 3two hours and 50 hours with stirring at the same temperature. The reaction was quenched by the addition of sat. bicarb solution (50 mL) and the mixture was extracted with EtOAc (3 × 30 mL). The combined organic layers were washed with brine (50 mL) and dried over Na_2SO_4. After filtration, the solvent was removed under reduced pressure, and the yellow oil was used in the next step without further purification as the intermediate chloride was not bench stable.

To the above mentioned crude mixture in THF (80 mL) was added 4-methoxybenzylamine ($PMB-NH_2$) (4.32 mL, 4.54 g, 33.1 mmol, 2.00 equiv.) at room temperature in one portion. The resulting mixture was stirred for 16 hours at the same temperature. The reaction was quenched by the addition of sat. bicarb solution (100 mL) and the mixture was extracted with CH_2Cl_2 (3 × 100 mL). The combined organic layers were washed with brine (100 mL) and dried over Na2SO4. After filtration, the solvent was removed under reduced pressure and the residual crude product was subjected to FCC (SiO_2, Hex:EtOAc = 1:2 → 0:1) to yield the desired 2-(4-methoxybenzyl)-2,3-dihydro-1*H*-pyrrolo[3,4-*c*]pyridin-1-one (**SI-17**) (565 mg, 2.22 mmol, 13%) as an off-white solid.

TLC (EtOAc): R_f = 0.20 [UV] [$KMnO_4$].

M.p.: 125 °C

^{1}H-NMR (400 MHz, $CDCl_3$, 300 K): δ [ppm] = 8.78 – 8.72 (m, 2H, H4, H6), 7.75 (dd, ^3J = 4.9, Hz, ^5J = 1.2 Hz, 1H), 7.25 – 7.21 (m, 2H, H2', H6'), 6.89 – 6.85 (m, 2H, H3', H5'), 4.75 (s, 2H, N-C*H*2-C1'), 4.33 (s, 2H, H3), 3.79 (s, 3H, OCH_3).

^{13}**C-NMR** (101 MHz, $CDCl_3$, 300 K): δ [ppm] = 166.7 (C1), 159.6 (C4'), 149.5 (C4), 145.1 (C6), 140.5 (C3a), 135.8 (C7a), 129.8 (C2', C6'), 128.5 (C1'), 117.9 (C7), 114.5 (C3', C5'), 55.4 (OCH_3), 48.1 (C3), 46.2 (N-CH_2-C1').

HRMS (**ESI**) *m/z* $[M+H]^+$ calculated for $[C_{15}H_{15}N_2O_2]^+$: 255.1128 found: 255.1123.

IR (film) $\tilde{\nu}_{max}/cm^{-1}$**:** 3038 (m, CH_{arom}), 2929 (m, CH_{aliph}), 1695 (s, C=O), 1583 (m, $C{=}C_{arom}$), 1451 (m, $C{=}C_{arom}$).

3-Benzyl-2,3-dihydro-1*H*-pyrrolo[3,4-*c*]pyridin-1-one (*rac*-**12a**)

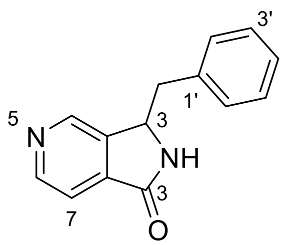

rac-**12a**
$C_{14}H_{12}N_2O$
M = 224.26 g mol^{-1}

According to a modified **GP E**, a 1 M solution of KHMDS in THF (1.08 mL, 217 mg, 1.08 mmol, 1.10 equiv.) was added dropwise to a solution of 2-(4-methoxybenzyl)-2,3-dihydro-1*H*-pyrrolo[3,4-*c*]pyridin-1-one (**SI-19**) (250 mg, 983 µmol, 1.00 equiv.) in anhydrous THF (13 mL) at –78 °C, upon which the reaction mixture turned violet. After 20 minutes, benzyl bromide (153 µL, 219 mg, 1.28 mmol, 1.30 equiv.) was added in one portion. The reaction was stirred for two hours at –78 °C. Subsequently, the solution was allowed to warm up to room temperature and quenched by the addition of sat. NH_4Cl (10 mL) and distilled water (10 mL). The aqueous layer was extracted with EtOAc (3 × 20 mL), and the combined organic phases were dried over Na_2SO_4, filtered and concentrated under reduced pressure. The crude product was then dissolved in $MeCN:H_2O$ (3:1, 29 mL), and diammonium cerium(IV) nitrate (1.08 g, 1.97 mmol, 2.00 equiv.) was added to the solution at room temperature. The yellow solution was stirred for two hours at the same temperature. Subsequently, water (20 mL) was added, and the mixture was extracted with EtOAc (3 × 20 mL). The combined organic layers were washed with brine (30 mL) and dried over Na_2SO_4. After filtration, the solvent was removed under reduced pressure and the residual crude product was subjected to FCC (SiO_2, EtOAc) to yield the desired racemic substrate 3-benzyl-2,3-dihydro-1*H*-pyrrolo[3,4-*c*]pyridin-1-one (*rac*-**12a**) (78 mg, 348 µmol, 35%) as a white solid.

TLC (EtOAc): R_f = 0.16 [UV] [$KMnO_4$].

M.p.: 221 °C.

^{1}**H-NMR** (400 MHz, $CDCl_3$, 300 K): δ [ppm] = 8.79 (d, ^3J = 4.9 Hz, 1H, H6), 8.68 (d, ^4J = 1.2 Hz, 1H, H4), 7.73 (dd, ^3J = 4.9 Hz, ^4J = 1.2 Hz, 1H, H7), 7.41 – 7.29 (m, 3H, H2', H4' H6'), 7.25 – 7.21 (m, 2H, H3', H5'), 6.53 (bs, 1H, NH), 4.93 (dd, ^3J = 9.0 Hz, ^3J = 5.4 Hz, 1H,

H3), 3.26 (dd, ^3J = 13.6 Hz, ^3J = 5.5 Hz, 1H, C7-CH$_a$-C1'), 2.88 (dd, J = 13.6 Hz, 8.9 Hz, 1H, C7-CH$_b$-C1').

^{13}C-NMR (101 MHz, CDCl$_3$, 300 K): δ [ppm] = 168.4 (C3), 149.8 (C6), 145.4 (C4), 141.1 (C7a), 139.6 (C3a), 136.3 (C1'), 129.3 (C2', C6'), 129.3 (C4'), 127.7 (C3', C5'), 117.9 (C7), 57.4 (C3), 41.3 (C3-*C*H$_2$-C1').

HRMS (**ESI**) *m/z* [M+H]$^+$ calculated for [C$_{14}$H$_{13}$N$_2$O]$^+$: 225.1022; found: 225.1017.

IR (film) $\tilde{\nu}_{max}$/cm^{-1}**:** 3164 (m, NH), 3054 (m, CH$_{arom}$), 2923 (m, CH$_{aliph}$), 1705 (s, C=O), 1586 (m, C=C$_{arom}$), 1432 (m, C=C$_{arom}$).

3-(Methoxycarbonyl)-4-methylpyridine 1-oxide (**SI-18**)

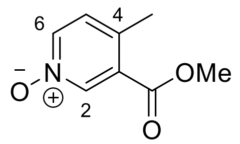

SI-18

$C_8H_9NO_3$

M = 167.16 g mol^{-1}

According to a modified procedure,[21] *meta*-chloroperoxybenzoic acid (8.90 g, 39.7 mmol, 77 w%, 1.20 equiv.) was added to a solution of methyl methyl 4-methylnicotinate (5.00 g, 33.1 mmol, 1.00 equiv.) in CH$_2$Cl$_2$ (167 mL) at 0 °C. The reaction was allowed to warm to room temperature and was stirred 16 hours at the same temperature. Afterwards, the reaction was quenched by the addition of sat. sodium sulfite solution (100 mL) and sat. bicarb solution (100 mL) and the mixture was extracted with CH$_2$Cl$_2$ (3 × 100 mL). The combined organic layers were washed with brine (100 mL) and dried over Na$_2$SO$_4$. After filtration, the solvent was removed under reduced pressure and the residual crude product was subjected to FCC (SiO$_2$, EtOAc:MeOH = 9:1 → 5:1) to yield the desired 3-(methoxycarbonyl)-4-methylpyridine 1-oxide (**SI-18**) (3.51 g, 21.0 mmol, 63%) as a white solid.

TLC (EtOAc:MeOH = 9:1): R_f = 0.13 [UV] [KMnO$_4$].

M.p.: 114 °C

^{1}H-NMR (400 MHz, CDCl$_3$, 300 K): δ [ppm] = 8.71 (d, ^4J = 2.0 Hz, 1H, H2), 8.18 (dd, ^3J = 6.7 Hz, ^4J = 2.0 Hz, 1H, H6), 7.15 (d, ^3J = 6.7 Hz, 1H, H5), 3.93 (s, 3H, COOCH$_3$), 2.59 (s, 3H, CH$_3$).

^{13}C-NMR (101 MHz, CDCl$_3$, 300 K): δ [ppm] = 164.1 (COO), 141.2 (C6), 140.7 (C2), 139.6 (C4), 129.0 (C5), 128.5 (C3), 52.9 (*C*OOCH$_3$), 20.5 (CH$_3$).

HRMS (**ESI**) *m/z* [M+H]$^+$ calculated for [C$_8$H$_{10}$NO$_3$]$^+$: 168.0655 found: 168.0653.

IR (film): $\tilde{v}_{max}$/cm^{-1} = 3107 (m, CH$_{arom}$), 2957 (m, CH$_{aliph}$), 1731 (s, C=O), 1080 (s, CO).

Methyl 4-(chloromethyl)nicotinate (SI-19)

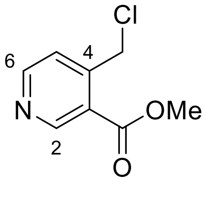

SI-19
$C_8H_8ClNO_2$
M = 185.61 g mol^{-1}

According to a modified procedure,[21] tosyl chloride (7.98 g, 41.9 mmol, 2.00 equiv.) was added to a solution of methyl 3-(methoxycarbonyl)-4-methylpyridine 1-oxide (3.50 g, 20.9 mmol, 1.00 equiv.) in 1,4-dioxane (21 mL) in one portion. The mixture was then stirred at 110 °C for 90 minutes upon which the reaction turned orange. The reaction was allowed to cool to room temperature and HCL (1M, 40 mL) was added. The resulting mixture was washed with Et_2O (3 × 60 mL) and the aqueous phase was neutralized by addition of solid $NaHCO_3$. The aqueous phase was then extracted with Et_2O (3 × 100 mL) The combined organic layers were washed with brine (100 mL) and dried over Na_2SO_4. After filtration, the solvent was removed under reduced pressure and the residual crude product was subjected to FCC (SiO_2, Hex:EtOAc = 3:1) to yield the desired methyl 4-(chloromethyl)nicotinate (**SI-19**) (1.76 g, 9.48 mmol, 45%) as an orange solid.

TLC (Hex:EtOAc = 3:1): R_f = 0.32 [UV] [$KMnO_4$].

M.p.: 54 °C

^{1}H-NMR (400 MHz, $CDCl_3$, 300 K): δ [ppm] = 9.16 (d, ^4J = 0.7 Hz, 1H, H2), 8.76 (d, ^3J = 5.2 Hz, 1H, H6), 7.61 (*virt.* dq, ^3J = 5.1 Hz, $^4J \approx {}^5J \approx$ 0.7 Hz, 1H, H5), 5.05 (d, ^4J = 0.7 Hz, 2H, CH_2Cl), 3.96 (s, 3H, $COOCH_3$).

^{13}C-NMR (101 MHz, $CDCl_3$, 300 K): δ [ppm] = 165.7 (COO), 153.4 (C6), 151.8 (C2), 147.5 (C4), 124.1 (C3), 124.0 (C5), 52.5 (COO*C*H_3), 43.0 (CH_3).

HRMS (ESI) *m/z* $[M+H]^+$ calculated for $[C_8H_9{}^{35}ClNO_2]^+$: 186.0316 found: 186.0315.

IR (film) $\tilde{\nu}_{max}$/cm^{-1}**:** 3081 (m, CH_{arom}), 2962 (m, CH_{aliph}), 1712 (s, C=O), 1590 (m, $C{=}C_{arom}$), 1439 (m, $C{=}C_{arom}$), 1285 (m, C-O), 841 (m, C-Cl).

2-(4-Methoxybenzyl)-1,2-dihydro-3*H*-pyrrolo[3,4-*c*]pyridin-3-one (SI-20)

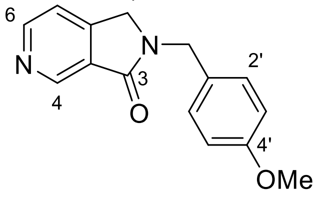

SI-20
$C_{15}H_{14}N_2O_2$
M = 254.29 g mol^{-1}

According to a modified **GP B**, 4-methoxybenzylamine (PMB-NH_2) (2.37 mL, 2.51 g, 18.3 mmol, 2.00 equiv.) was added in one portion to a solution of methyl 4-(chloromethyl)nicotinate (**SI-21**) (1.70 g, 9.16 mmol, 1.00 equiv.) in THF (47 mL) at room temperature. The resulting suspension was stirred for 16 hours at the same temperature. The reaction was quenched by the addition of sat. ammonium chloride solution (60 mL) and water (60 mL) and the mixture was extracted with EtOAc (3 × 70 mL). The combined organic layers were washed with brine (70 mL) and dried over Na_2SO_4. After filtration, the solvent was removed under

reduced pressure and the residual crude product was subjected to FCC (SiO_2, EtOAc) to yield the desired 2-(4-methoxybenzyl)-1,2-dihydro-3*H*-pyrrolo[3,4-*c*]pyridin-3-one (**SI-20**) (1.45 g, 5.70 mmol, 62%) as an off-white solid.

TLC (EtOAc): R_f = 0.12 [UV] [$KMnO_4$].
M.p.: 134 °C.
^{1}H-NMR (400 MHz, $CDCl_3$, 300 K): δ [ppm] = 9.12 (d, ^5J = 1.0 Hz, 1H, H4), 8.72 (d, ^3J = 5.1 Hz, 1H, H6), 7.35 (*virt.* dq, ^3J = 5.1 Hz, $^4J \approx {}^5J \approx$ 1.0 Hz, 1H, H7), 7.25 – 7.21 (m, 2H. H2', H6'), 6.91 – 6.84 (m, 2H, H3', H5'), 4.73 (s, 2H, N-CH_2-C1'), 4.28 (d, ^3J = 1.0 Hz, 2H, H1), 3.79 (s, 3H, OCH_3).
^{13}C-NMR (101 MHz, $CDCl_3$, 300 K): δ [ppm] = 166.7 (C3) 159.5 (C4'), 151.5 (C6), 149.8 (C7a), 146.3 (C4), 129.8 (C2', C6'), 129.0 (C3a), 128.6(C1'), 118.2 (C7), 114.4 (C3', C5'), 55.5 (OCH_3), 49.2 (C1), 45.9 (N-*C*H_2-C1').
HRMS (**ESI**) *m/z* $[M+H]^+$ calculated for $[C_{15}H_{15}N_2O_2]^+$: 255.1128 found: 255.1124.
IR (film): $\tilde{v}_{max}/cm^{-1}$ = 3063 (w, CH_{arom}), 2934 (w, CH_{aliph}), 1672 (s, C=O), 1610 (m, $C{=}C_{arom}$), 1511 (m, $C{=}C_{arom}$).

1-Benzyl-1,2-dihydro-3*H*-pyrrolo[3,4-*c*]pyridin-3-one (*rac*-**12b**)

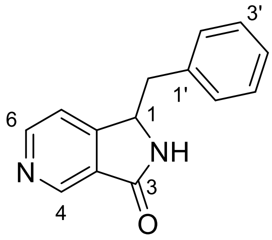

rac-**12b**
$C_{14}H_{12}N_2O$
M = 224.26 g mol^{-1}

According to a modified **GP E**, a 1 M solution of KHMDS in THF (2.16 mL, 434 mg, 2.16 mmol, 1.10 equiv.) was added dropwise to a solution of 2-(4-methoxybenzyl)-1,2-dihydro-3*H*-pyrrolo[3,4-*c*]pyridin-3-one (**SI-20**) (500 mg, 1.97 mmol, 1.00 equiv.) in anhydrous THF (25 mL) at –78 °C, upon which the reaction mixture turned violet. After 20 minutes, benzyl bromide (304 µL, 437 mg, 2.56 mmol, 1.30 equiv.) was added in one portion. The reaction was stirred for two hours at –78 °C. Subsequently, the solution was allowed to warm up to room temperature and quenched by the addition of sat. NH_4Cl (20 mL) and distilled water (20 mL). The aqueous layer was extracted with EtOAc (3 × 20 mL), and the combined organic phases were dried over Na_2SO_4, filtered and concentrated under reduced pressure. The crude product was then dissolved in MeCN:H_2O (3:1, 58 mL), and diammonium cerium(IV) nitrate (1.08 g, 1.97 mmol, 1.00 equiv.) was added to the solution at room temperature. The yellow solution was stirred for two hours at the same temperature. Subsequently, water (40 mL) was added, and the mixture was extracted with EtOAc (3 × 40 mL). The combined organic layers were washed with brine (30 mL) and dried over Na_2SO_4. After filtration, the solvent was removed under

reduced pressure and the residual crude product was subjected to FCC (SiO_2, EtOAc:MeOH = 1:0 → 95:5) to yield the desired racemic substrate 1-benzyl-1,2-dihydro-3*H*-pyrrolo[3,4-*c*]pyridin-3-one (*rac*-**12b**) (20 mg, 89.2 µmol, 5%) as a white solid.

TLC (EtOAc:MeOH =95:5): R_f = 0.16 [UV] [$KMnO_4$].
M.p.: 195 °C.
^{1}H-NMR (400 MHz, $CDCl_3$, 300 K): δ [ppm] = 9.08 (s, 1H, H4), 8.75 (d, ^3J = 5.1 Hz, 1H, H6), 7.39 – 7.28 (m, 3H, H2', H4' H6'), 7.25 – 7.18 (m, 3H, H7, H3', H5'), 6.75 (bs, 1H, NH), 4.86 (dd, ^3J = 8.7 Hz, ^3J = 5.8 Hz, 1H, H1), 3.18 (dd, ^2J = 13.6 Hz, ^3J = 5.8 Hz, 1H, C1-CH_a-C1'), 2.90 (dd, ^2J = 13.6 Hz, ^3J = 8.7 Hz, 1H, C1-CH_b-C1').
^{13}C-NMR (101 MHz, $CDCl_3$, 300 K): δ [ppm] = 168.8 (C3), 155.0 (C7a), 152.0 (C6), 146.6 (C4), 136.1 (C1'), 129.3 (C3', C5'), 129.2 (C2', C6'), 128.0 (C3a), 127.7 (C4'), 118.1 (C7), 58.1 (C1), 40.9 (C1-*C*H_2-C1').
HRMS (ESI) *m/z* $[M+H]^+$ calculated for $[C_{14}H_{13}N_2O]^+$: 225.1022; found: 225.1019.
IR (film): $\tilde{v}_{max}/cm^{-1}$ = 3164 (m, NH), 3029 (m, CH_{arom}), 2919 (m, CH_{aliph}), 1689 (s, C=O) 1614 (m, $C=C_{arom}$).

6-(4-Methoxybenzyl)-5,6-dihydro-7*H*-pyrrolo[3,4-*b*]pyridin-7-one (SI-21)

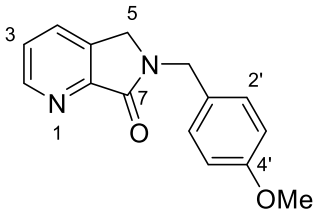

SI-21
$C_{15}H_{14}N_2O_2$
M = 254.29 g mol^{-1}

According to a modified **GP B**, 4-methoxybenzylamine (PMB-NH_2) (1.14 mL, 1.19 g, 8.69 mmol, 2.00 equiv.) was added in one portion to a solution of methyl 3-(bromomethyl)picolinate (1.00 g, 4.35 mmol, 1.00 equiv.) in THF:DMF (1:1, 22 mL) at room temperature. The resulting suspension was stirred for 16 hours at the same temperature. The reaction was quenched by the addition of sat. ammonium chloride solution (50 mL) and water (50 mL) and the mixture was extracted with CH_2Cl_2 (3 × 100 mL). The combined organic layers were washed with brine (100 mL) and dried over Na_2SO_4. After filtration, the solvent was removed under reduced pressure and the residual crude product was subjected to FCC (SiO_2, EtOAc) to yield the desired 6-(4-methoxybenzyl)-5,6-dihydro-7*H*-pyrrolo[3,4-*b*]pyridin-7-one (**SI-21**) (848 mg, 3.34 mmol, 77%) as an off-white solid.

TLC (EtOAc): R_f = 0.14 [UV] [$KMnO_4$].
M.p.: 189 °C.

^{1}H-NMR (400 MHz, $CDCl_3$, 300 K): *δ* [ppm] = 8.79 – 8.74 (m, 1H, H2), 7.73 (ddt, ^3J = 7.7 Hz, ^4J = 1.5 Hz, ^4J = 0.7 Hz, 1H, H4), 7.38 (dd, ^3J = 7.7, ^3J = 4.8 Hz, 1H, H3), 7.28 – 7.21 (m, 2H, H2', H6'), 6.89 – 6.81 (m, 2H, H3', H5'), 4.79 (s, 2H, N-CH_2-C1'), 4.24 (s, 2H, H5), 3.78 (s, 3H, OCH_3).

^{13}C-NMR (101 MHz, $CDCl_3$, 300 K): *δ* [ppm] = 166.4 (C7), 159.5 (C4'), 151.0 (C2), 135.2 (C7a), 131.2 (C4), 129.9 (C2', C6'), 128.7 (C1'), 125.2 (C3), 114.4 (C3', C5'), 55.4 (OCH_3), 47.1 (C5), 46.4 (N-CH_2-C1').

HRMS (**ESI**) *m/z* $[M+H]^+$ calculated for $[C_{15}H_{15}N_2O_2]^+$: 255.1128 found: 255.1123.

IR (film): $\tilde{v}_{max}/cm^{-1}$ = 3089 (w, CH_{arom}), 2929 (w, CH_{aliph}), 1686 (s, C=O), 1587 (m, $C=C_{arom}$), 1511 (m, $C=C_{arom}$).

5-Benzyl-5,6-dihydro-7*H*-pyrrolo[3,4-*b*]pyridin-7-one (*rac*-**13c**)

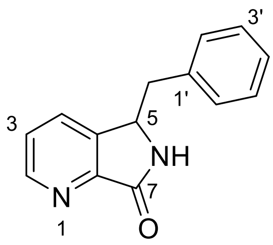

rac-**13c**
$C_{14}H_{12}N_2O$
M = 224.26 g mol^{-1}

According to a modified **GP E**, a 1 M solution of KHMDS in THF (2.16 mL, 434 mg, 2.16 mmol, 1.10 equiv.) was added dropwise to a solution of 6-(4-methoxybenzyl)-5,6-dihydro-7*H*-pyrrolo[3,4-*b*]pyridin-7-one (**SI-21**) (500 mg, 1.97 mmol, 1.00 equiv.) in anhydrous THF (25 mL) at –78 °C, upon which the reaction mixture turned violet. After 20 minutes, benzyl bromide (304 µL, 437 mg, 2.56 mmol, 1.30 equiv.) was added in one portion. The reaction was stirred for two hours at –78 °C. Subsequently, the solution was allowed to warm up to room temperature and quenched by the addition of sat. NH_4Cl (20 mL) and distilled water (20 mL). The aqueous layer was extracted with EtOAc (3 × 20 mL), and the combined organic phases were dried over Na_2SO_4, filtered and concentrated under reduced pressure. The crude product was then dissolved in MeCN:H_2O (3:1, 58 mL), and diammonium cerium(IV) nitrate (1.08 g, 1.97 mmol, 1.00 equiv.) was added to the solution at room temperature. The yellow solution was stirred for two hours at the same temperature. Subsequently, water (40 mL) was added, and the mixture was extracted with EtOAc (3 × 40 mL). The combined organic layers were washed with brine (30 mL) and dried over Na_2SO_4. After filtration, the solvent was removed under reduced pressure and the residual crude product was subjected to FCC (SiO_2, EtOAc) to yield the desired racemic substrate 5-benzyl-5,6-dihydro-7*H*-pyrrolo[3,4-*b*]pyridin-7-one (*rac*-**13c**) (41 mg, 183 µmol, 9%) as a white solid.

TLC (EtOAc:): R_f = 0.18 [UV] [$KMnO_4$].

M.p.: 196 °C.

^{1}H-NMR (400 MHz, $CDCl_3$, 300 K): δ [ppm] = 8.79 (dd, ^{3}J = 4.8 Hz, ^{4}J = 1.5 Hz, 1H, H2), 7.39 – 7.28 (m, 2H, H4, NH), 7.42 – 7.28 (m, 4H, H3, H2', H4' H6'), 7.24 – 7.18 (m, 2H, H3', H5'), 4.86 (*virt.* t., $^{3}J \approx {}^{3}J \approx$ 7.2 Hz, 1H, H5), 3.14 – 2.99 (m, 2H, C1-CH_a-C1', C1-CH_b-C1').
^{13}C-NMR (101 MHz, $CDCl_3$, 300 K): δ [ppm] = 168.6 (C7), 151.3 (C2), 150.3 (C7a), 140.5 (C4a), 136.2 (C1'), 131.5 (C4), 129.4 (C3', C5'), 129.1 (C2', C6'), 127.6 (C4'), 125.4 (C3), 56.0 (C5), 41.0 (C1-*C*H_2-C1').
HRMS (**ESI**) *m/z* $[M+H]^+$ calculated for $[C_{14}H_{13}N_2O]^+$: 225.1022; found: 225.1019.

10. Photochemical Deracemization Reactions

(*S*)-7-Benzyl-6,7-dihydro-5*H*-pyrrolo[3,4-*b*]pyridin-5-one (3a)

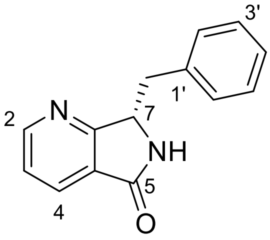

3a
$C_{14}H_{12}N_2O$
M = 224.26 g mol^{-1}

According to **GP I**, a degassed solution of *rac*-**3a** (5.61 mg, 25.0 µmol, 1.00 equiv.) and enantiomerically pure (–)-benzophenone (–)-**2b** (541 µg, 1.25 µmol, 5 mol%) in 10 mL α,α,α-trifluorotoluene was irradiated at λ = 350 nm for 13 hours. After irradiation, the solvent was evaporated and the residue was purified by FCC (SiO_2, EtOAc) to obtain (*S*)-7-benzyl-6,7-dihydro-5*H*-pyrrolo[3,4-*b*]pyridin-5-one (**3a**) (4.70 mg, 21.0 µmol, 84%, 98% *ee*) as a colorless solid.

TLC (EtOAc): R_f = 0.29 [UV] [$KMnO_4$].

Specific Rotation: $[\alpha]_D^{25}$: –102 (*c* = 1.0, CH_2Cl_2) [98% *ee*].

Chiral HPLC: 98% *ee* (AD-H 250 × 4.6 mm, *n*-Hep/*iso*-PrOH = 90/10, 1 mL/min, λ = 210 nm); t_R = 16.21 min (major, **3a**), 21.37 min (minor, *ent*-**3a**).

0.5 mmol scale

According to a modified **GP I**, a degassed solution of *rac*-**3a** (112 mg, 0.50 mmol, 1.00 equiv.) and enantiomerically pure (–)-benzophenone (–)-**2b** (10.8 mg, 25.0 µmol, 5 mol%) in 200 mL α,α,α-trifluorotoluene was irradiated at λ = 350 nm for 30 hours. After irradiation, the solvent was evaporated and the residue was purified by FCC (SiO_2, EtOAc) to obtain **3a** (102 mg, 45.5 µmol, 91%, 95% *ee*) as a colorless solid.

(*S*)-7-(4-(*tert*-Butyl)benzyl)-6,7-dihydro-5*H*-pyrrolo[3,4-*b*]pyridin-5-one (3b)

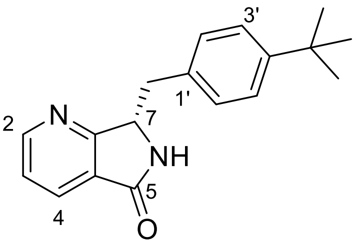

3b
$C_{18}H_{20}N_2O$
M = 280.37 g mol^{-1}

According to **GP I**, a degassed solution of *rac*-**3b** (7.00 mg, 25.0 µmol, 1.00 equiv.) and enantiomerically pure (–)-benzophenone (–)-**2b** (541 µg, 1.25 µmol, 5 mol%) in 10 mL α,α,α-trifluorotoluene was irradiated at λ = 350 nm for 13 hours. After irradiation, the solvent was evaporated and the residue was purified by FCC (SiO_2, EtOAc) to obtain (*S*)-7-(4-(*tert*-butyl)benzyl)-6,7-dihydro-5*H*-pyrrolo[3,4-*b*]pyridin-5-one (**3b**) (6.19 mg, 22.1 µmol, 88%, 99% *ee*) as a colorless solid.

TLC (EtOAc): R_f = 0.31 [UV] [$KMnO_4$].

Specific Rotation: $[\alpha]_D^{25}$: –94 (*c* = 1.0, CH_2Cl_2) [99% *ee*].

Chiral HPLC: 99% *ee* (AD-H 250 × 4.6 mm, *n*-Hep/*iso*-PrOH = 90/10, 1 mL/min, λ = 210 nm); t_R = 8.94 min (major, **3b**), 9.92 min (minor, *ent*-**3b**).

(*S*)-7-(3,5-Dimethylbenzyl)-6,7-dihydro-5*H*-pyrrolo[3,4-*b*]pyridin-5-one (**3c**)

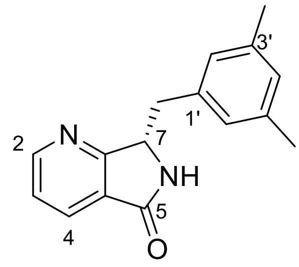

3c
$C_{16}H_{16}N_2O$
M = 252.32 g mol^{-1}

According to **GP I**, a degassed solution of *rac*-**3c** (6.31 mg, 25.0 µmol, 1.00 equiv.) and enantiomerically pure (–)-benzophenone (–)-**2b** (541 µg, 1.25 µmol, 5 mol%) in 10 mL α,α,α-trifluorotoluene was irradiated at λ = 350 nm for 13 hours. After irradiation, the solvent was evaporated and the residue was purified by FCC (SiO_2, EtOAc) to obtain (*S*)-7-(3,5-dimethylbenzyl)-6,7-dihydro-5*H*-pyrrolo[3,4-*b*]pyridin-5-one (**3c**) (4.98 mg, 19.7 µmol, 79%, 93% *ee*) as a colorless solid.

TLC (EtOAc): R_f = 0.27 [UV] [$KMnO_4$].
Specific Rotation: $[\alpha]_D^{25}$: –100 (*c* = 1.0, CH_2Cl_2) [93% *ee*].
Chiral HPLC: 93% *ee* (IC 250 × 4.6 mm, *n*-Hep/*iso*-PrOH = 90/10, 1 mL/min, λ = 210 nm); t_R = 8.35 min (minor, *ent*-**3c**), 10.38 min (major, **3c**).

(*S*)-7-(4-Fluorobenzyl)-6,7-dihydro-5*H*-pyrrolo[3,4-*b*]pyridin-5-one (**3d**)

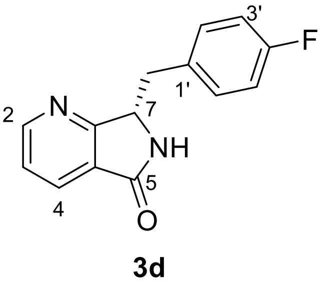

3d
$C_{14}H_{11}FN_2O$
M = 242.25 g mol^{-1}

According to **GP I**, a degassed solution of *rac*-**3d** (6.06 mg, 25.0 µmol, 1.00 equiv.) and enantiomerically pure (–)-benzophenone (–)-**2b** (541 µg, 1.25 µmol, 5 mol%) in 10 mL α,α,α-trifluorotoluene was irradiated at λ = 350 nm for 13 hours. After irradiation, the solvent was evaporated and the residue was purified by FCC (SiO_2, EtOAc) to obtain (*S*)-7-(4-fluorobenzyl)-6,7-dihydro-5*H*-pyrrolo[3,4-*b*]pyridin-5-one (**3d**) (5.21 mg, 21.5 µmol, 86%, 96% *ee*) as a colorless solid.

TLC (EtOAc): R_f = 0.27 [UV] [$KMnO_4$].
Specific Rotation: $[\alpha]_D^{25}$: –132 (*c* = 1.0, CH_2Cl_2) [96% *ee*].
Chiral HPLC: 96% *ee* (AD-H 250 × 4.6 mm, *n*-Hep/*iso*-PrOH = 90/10, 1 mL/min, λ = 210 nm); t_R = 12.03 min (major, **3d**), 22.22 min (minor, *ent*-**3d**).

(*S*)-7-(4-Bromobenzyl)-6,7-dihydro-5*H*-pyrrolo[3,4-*b*]pyridin-5-one (3e)

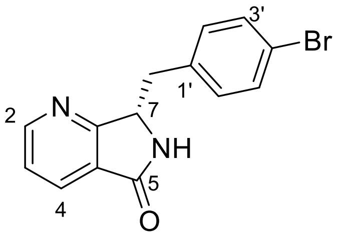

3e

$C_{14}H_{11}BrN_2O$

M = 303.16 g mol^{-1}

According to **GP I**, a degassed solution of *rac*-**3e** (7.58 mg, 25.0 µmol, 1.00 equiv.) and enantiomerically pure (–)-benzophenone (–)-**2b** (541 µg, 1.25 µmol, 5 mol%) in 10 mL α,α,α-trifluorotoluene was irradiated at λ = 350 nm for 13 hours. After irradiation, the solvent was evaporated and the residue was purified by FCC (SiO_2, EtOAc) to obtain (*S*)-7-(4-bromobenzyl)-6,7-dihydro-5*H*-pyrrolo[3,4-*b*]pyridin-5-one (**3e**) (6.12 mg, 20.2 µmol, 81%, 94% *ee*) as a colorless solid.

TLC (EtOAc): R_f = 0.20 [UV] [$KMnO_4$].

Specific Rotation: $[\alpha]_D^{25}$: –34 (*c* = 1.0, CH_2Cl_2) [94% *ee*].

Chiral HPLC: 94% *ee* (AD-H 250 × 4.6 mm, *n*-Hep/*iso*-PrOH = 90/10, 1 mL/min, λ = 210 nm); t_R = 13.83 min (major, **3e**), 22.62 min (minor, *ent*-**3e**).

(*S*)-7-(Cyclopropylmethyl)-6,7-dihydro-5*H*-pyrrolo[3,4-*b*]pyridin-5-one (3f)

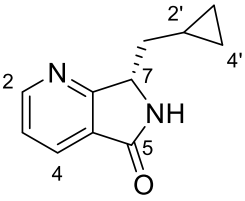

3f

$C_{11}H_{12}N_2O$

M = 188.23 g mol^{-1}

According to **GP I**, a degassed solution of *rac*-**3f** (4.71 mg, 25.0 µmol, 1.00 equiv.) and enantiomerically pure (–)-benzophenone (–)-**2b** (541 µg, 1.25 µmol, 5 mol%) in 10 mL α,α,α-trifluorotoluene was irradiated at λ = 350 nm for 13 hours. After irradiation, the solvent was evaporated and the residue was purified by FCC (SiO_2, EtOAc) to obtain (*S*)-7-(cyclopropylmethyl)-6,7-dihydro-5*H*-pyrrolo[3,4-*b*]pyridin-5-one (**3f**) (4.35 mg, 23.1 µmol, 92%, 86% *ee*) as a colorless solid.

TLC (EtOAc): R_f = 0.21 [UV] [$KMnO_4$].

Specific Rotation: $[\alpha]_D^{25}$: –22 (*c* = 1.0, CH_2Cl_2) [86% *ee*].

Chiral HPLC: 86% *ee* (IC 250 × 4.6 mm, *n*-Hep/*iso*-PrOH = 90/10, 1 mL/min, λ = 210 nm); t_R = 24.04 min (minor, *ent*-**3f**), 25.51 min (major, **3f**).

(*S*)-7-(Cyclobutylmethyl)-6,7-dihydro-5*H*-pyrrolo[3,4-*b*]pyridin-5-one (3g)

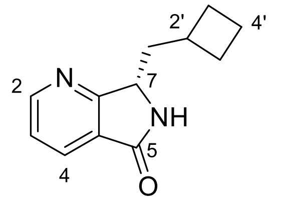

3g

$C_{12}H_{14}N_2O$

M = 202.26 g mol^{-1}

According to **GP I**, a degassed solution of *rac*-**3g** (5.06 mg, 25.0 µmol, 1.00 equiv.) and enantiomerically pure (–)-benzophenone (–)-**2b** (541 µg, 1.25 µmol, 5 mol%) in 10 mL α,α,α-trifluorotoluene was irradiated at λ = 350 nm for 13 hours. After irradiation, the solvent was evaporated and the residue was purified by FCC (SiO_2, EtOAc) to obtain (*S*)-7-(cyclobutylmethyl)-6,7-dihydro-5*H*-pyrrolo[3,4-*b*]pyridin-5-one (**3g**) (4.51 mg, 22.3 µmol, 89%, 99% *ee*) as a colorless solid.

TLC (EtOAc): R_f = 0.24 [UV] [$KMnO_4$].

Specific Rotation: $[\alpha]_D^{25}$: –12 (*c* = 1.0, CH_2Cl_2) [99% *ee*].

Chiral HPLC: 99% *ee* (IC 250 × 4.6 mm, *n*-Hep/*iso*-PrOH = 90/10, 1 mL/min, λ = 210 nm); t_R = 23.80 min (major, **3g**), 25.26 min (minor, *ent*-**3g**).

0.20 mmol scale

According to a modified **GP I**, a degassed solution of *rac*-**3g** (40.5 mg, 200 µmol, 1.00 equiv.) and enantiomerically pure (–)-benzophenone (–)-**2b** (4.33 mg, 10.0 µmol, 5 mol%) in 80 mL α,α,α-trifluorotoluene was irradiated at λ = 350 nm for 18 hours. After irradiation, the solvent was evaporated and the residue was purified by FCC (SiO_2, EtOAc) to obtain **3g** (32.0 mg, 158 µmol, 79%, 99% *ee*) as a colorless solid.

(*S*)-*tert*-Butyl 3-((5-oxo-6,7-dihydro-5*H*-pyrrolo[3,4-*b*]pyridin-7-yl)methyl)azetidine-1-carboxylate (3h)

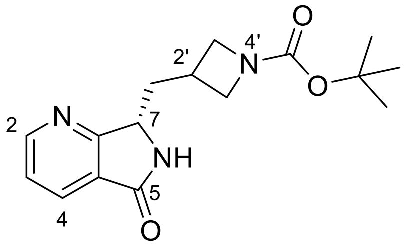

3h

$C_{16}H_{21}N_3O_3$

M = 303.36 g mol^{-1}

According to **GP I**, a degassed solution of *rac*-**3h** (7.58 mg, 25.0 µmol, 1.00 equiv.) and enantiomerically pure (–)-benzophenone (–)-**2b** (541 µg, 1.25 µmol, 5 mol%) in 10 mL α,α,α-trifluorotoluene was irradiated at λ = 350 nm for 13 hours. After irradiation, the solvent was evaporated and the residue was purified by FCC (SiO_2, EtOAc) to obtain (*S*)-*tert*-butyl 3-((5-oxo-6,7-dihydro-5*H*-pyrrolo[3,4-*b*]pyridin-7-yl)methyl)azetidine-1-carboxylate (**3h**) (6.21 mg, 20.5 µmol, 82%, 95% *ee*) as a colorless solid.

TLC (EtOAc): R_f = 0.16 [UV] [$KMnO_4$].

Specific Rotation: $[\alpha]_D^{25}$: –22 (*c* = 1.0, CH_2Cl_2) [95% *ee*].

Chiral HPLC: 95% *ee* (AD-H 250 × 4.6 mm, *n*-Hep/*iso*-PrOH = 90/10, 1 mL/min, λ = 210 nm); t_R = 20.87 min (major, **3h**), 37.83 min (minor, *ent*-**3h**).

(*S*)-7-Ethyl-6,7-dihydro-5*H*-pyrrolo[3,4-*b*]pyridin-5-one (**3i**)

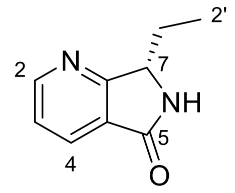

3i
$C_9H_{10}N_2O$
M = 162.19 g mol^{-1}

According to **GP I**, a degassed solution of *rac*-**3i** (4.06 mg, 25.0 µmol, 1.00 equiv.) and enantiomerically pure (–)-benzophenone (–)-**2b** (541 µg, 1.25 µmol, 5 mol%) in 10 mL α,α,α-trifluorotoluene was irradiated at λ = 350 nm for 13 hours. After irradiation, the solvent was evaporated and the residue was purified by FCC (SiO_2, EtOAc) to obtain (*S*)-7-ethyl-6,7-dihydro-5*H*-pyrrolo[3,4-*b*]pyridin-5-one (**3i**) (3.10 mg, 19.1 µmol, 76%, 98% *ee*) as a colorless solid.

TLC (EtOAc): R_f = 0.20 [UV] [$KMnO_4$].
Specific Rotation: $[a]_D^{25}$: –38 (*c* = 1.0, CH_2Cl_2) [98% *ee*].
Chiral HPLC: 98% *ee* (IC 250 × 4.6 mm, *n*-Hep/*iso*-PrOH = 90/10, 1 mL/min, λ = 210 nm); t_R = 8.96 min (minor, *ent*-**3i**), 9.70 min (major, **3i**).

(*S*)-7-Butyl-6,7-dihydro-5*H*-pyrrolo[3,4-*b*]pyridin-5-one (**3j**)

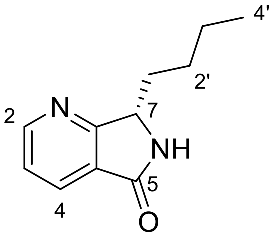

3j
$C_{11}H_{14}N_2O$
M = 190.25 g mol^{-1}

According to **GP I**, a degassed solution of *rac*-**3j** (4.76 mg, 25.0 µmol, 1.00 equiv.) and enantiomerically pure (–)-benzophenone (–)-**2b** (541 µg, 1.25 µmol, 5 mol%) in 10 mL α,α,α-trifluorotoluene was irradiated at λ = 350 nm for 13 hours. After irradiation, the solvent was evaporated and the residue was purified by FCC (SiO_2, EtOAc) to obtain (*S*)-7-butyl-6,7-dihydro-5*H*-pyrrolo[3,4-*b*]pyridin-5-one (**3j**) (4.18 mg, 22.0 µmol, 88%, 97% *ee*) as a colorless solid.

TLC (EtOAc): R_f = 0.21 [UV] [$KMnO_4$].
Specific Rotation: $[a]_D^{25}$: –42 (*c* = 1.0, CH_2Cl_2) [97% *ee*].
Chiral HPLC: 97% *ee* (IC 250 × 4.6 mm, *n*-Hep/*iso*-PrOH = 90/10, 1 mL/min, λ = 210 nm); t_R = 8.86 min (major, **3j**), 40.85 min (minor, *ent*-**3j**).

(*S*)-2-Chloro-7-isopentyl-6,7-dihydro-5*H*-pyrrolo[3,4-*b*]pyridin-5-one (3k)

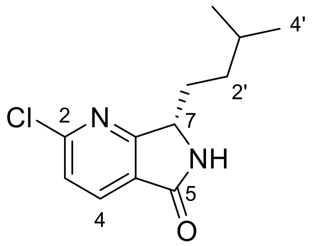

3k

$C_{12}H_{15}ClN_2O$

M = 238.72 g mol^{-1}

According to **GP I**, a degassed solution of *rac*-**3k** (5.97 mg, 25.0 µmol, 1.00 equiv.) and enantiomerically pure (–)-benzophenone (–)-**2b** (541 µg, 1.25 µmol, 5 mol%) in 10 mL α,α,α-trifluorotoluene was irradiated at λ = 350 nm for 13 hours. After irradiation, the solvent was evaporated and the residue was purified by FCC (SiO_2, EtOAc) to obtain (*S*)-2-chloro-7-isopentyl-6,7-dihydro-5*H*-pyrrolo[3,4-*b*]pyridin-5-one (**3k**) (5.14 mg, 21.5 µmol, 86%, 97% *ee*) as a colorless solid.

TLC (Hex:EtOAc = 2:1): R_f = 0.16 [UV] [$KMnO_4$].

Specific Rotation: $[\alpha]_D^{25}$: –82 (*c* = 1.0, CH_2Cl_2) [97% *ee*].

Chiral HPLC: 97% *ee* (AS-H 250 × 4.6 mm, *n*-Hep/*iso*-PrOH = 70/30, 1 mL/min, λ = 210 nm); t_R = 11.43 min (major, **3k**), 15.30 min (minor, *ent*-**3k**).

(*S*)-7-Benzyl-2-bromo-6,7-dihydro-5*H*-pyrrolo[3,4-*b*]pyridin-5-one (3l)

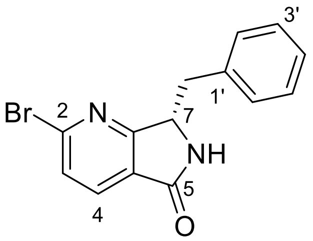

3l

$C_{14}H_{11}BrN_2O$

M = 303.16 g mol^{-1}

According to **GP I**, a degassed solution of *rac*-**3l** (7.58 mg, 25.0 µmol, 1.00 equiv.) and enantiomerically pure (–)-benzophenone (–)-**2b** (541 µg, 1.25 µmol, 5 mol%) in 10 mL α,α,α-trifluorotoluene was irradiated at λ = 350 nm for 13 hours. After irradiation, the solvent was evaporated and the residue was purified by FCC (SiO_2, EtOAc) to obtain (*S*)-7-benzyl-2-bromo-6,7-dihydro-5*H*-pyrrolo[3,4-*b*]pyridin-5-one (**3l**) (7.31 mg, 24.1 µmol, 96%, 90% *ee*) as a colorless solid.

TLC (Hex:EtOAc = 1:1): R_f = 0.27 [UV] [$KMnO_4$].

Specific Rotation: $[\alpha]_D^{25}$: –104 (*c* = 1.0, CH_2Cl_2) [90% *ee*].

Chiral HPLC: 90% *ee* (IA 250 × 4.6 mm, *n*-Hep/*iso*-PrOH = 90/10, 1 mL/min, λ = 210 nm); t_R = 13.80 min (major, **3l**), 19.39 min (minor, *ent*-**3l**).

0.20 mmol scale

According to a modified **GP I**, a degassed solution of *rac*-**3l** (60.6 mg, 200 µmol, 1.00 equiv.) and enantiomerically pure (-)-benzophenone **2b** (4.33 mg, 10.0 µmol, 5 mol%) in 80 mL α,α,α-trifluorotoluene was irradiated at λ = 350 nm for 18 hours. After irradiation, the solvent was

evaporated and the residue was purified by FCC (SiO_2, EtOAc) to obtain (*S*)-7-Benzyl-2-bromo-6,7-dihydro-5*H*-pyrrolo[3,4-*b*]pyridin-5-one (**3l**) (51.5 mg, 170 µmol, 85%, 92% *ee*) as a colorless solid.

(*S*)-7-Benzyl-2-(trifluoromethyl)-6,7-dihydro-5*H*-pyrrolo[3,4-*b*]pyridin-5-one (**3m**)

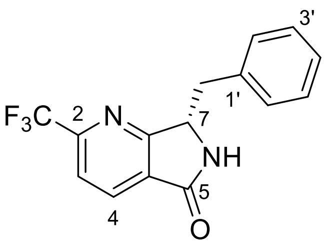

3m
$C_{15}H_{11}F_3N_2O$
M = 292.26 g mol^{-1}

According to **GP I**, a degassed solution of *rac*-**3m** (7.31 mg, 25.0 µmol, 1.00 equiv.) and enantiomerically pure (–)-benzophenone (–)-**2b** (541 µg, 1.25 µmol, 5 mol%) in 10 mL α,α,α-trifluorotoluene was irradiated at λ = 350 nm for 13 hours. After irradiation, the solvent was evaporated and the residue was purified by FCC (SiO_2, EtOAc) to obtain (*S*)-7-benzyl-2-(trifluoromethyl)-6,7-dihydro-5*H*-pyrrolo[3,4-*b*]pyridin-5-one (**3m**) (5.94 mg, 20.3 µmol, 81%, 96% *ee*) as a colorless solid.

TLC (Hex:EtOAc = 1:1): R_f = 0.43 [UV] [$KMnO_4$].
Specific Rotation: $[\alpha]_D^{25}$: –24 (*c* = 1.0, CH_2Cl_2) [96% *ee*].
Chiral HPLC: 96% *ee* (AD-H 250 × 4.6 mm, *n*-Hep/*iso*-PrOH = 90/10, 1 mL/min, λ = 210 nm); t_R = 10.59 min (major, **3m**), 14.92 min (minor, *ent*-**3m**).

(*S*)-7-Benzyl-2-chloro-6,7-dihydro-5*H*-pyrrolo[3,4-*b*]pyridin-5-one (**3n**)

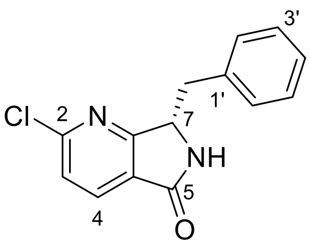

3n
$C_{14}H_{11}ClN_2O$
M = 258.71 g mol^{-1}

According to **GP I**, a degassed solution of *rac*-**3n** (6.47 mg, 25.0 µmol, 1.00 equiv.) and enantiomerically pure (–)-benzophenone (–)-**2b** (541 µg, 1.25 µmol, 5 mol%) in 10 mL α,α,α-trifluorotoluene was irradiated at λ = 350 nm for 13 hours. After irradiation, the solvent was evaporated and the residue was purified by FCC (SiO_2, EtOAc) to obtain (*S*)-7-benzyl-2-chloro-6,7-dihydro-5*H*-pyrrolo[3,4-*b*]pyridin-5-one (**3n**) (5.55 mg, 21.5 µmol, 86%, 95% *ee*) as a colorless solid.

TLC (Hex:EtOAc = 1:1): R_f = 0.24 [UV] [$KMnO_4$].
Specific Rotation: $[\alpha]_D^{25}$: –152 (*c* = 1.0, CH_2Cl_2) [95% *ee*].
Chiral HPLC: 95% *ee* (AD-H 250 × 4.6 mm, *n*-Hep/*iso*-PrOH = 90/10, 1 mL/min, λ = 210 nm); t_R = 16.86 min (major, **3n**), 24.47 min (minor, *ent*-**3n**).

(*S*)-7-Benzyl-3-bromo-6,7-dihydro-5*H*-pyrrolo[3,4-*b*]pyridin-5-one (3o)

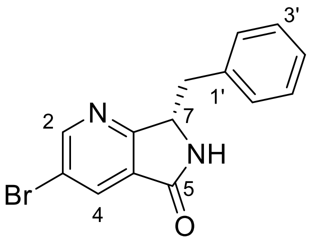

3o

$C_{14}H_{11}BrN_2O$

M = 303.16 g mol^{-1}

According to **GP I**, a degassed solution of *rac*-**3o** (7.58 mg, 25.0 µmol, 1.00 equiv.) and enantiomerically pure (–)-benzophenone (–)**-2b** (541 µg, 1.25 µmol, 5 mol%) in 10 mL α,α,α-trifluorotoluene was irradiated at λ = 350 nm for 13 hours. After irradiation, the solvent was evaporated and the residue was purified by FCC (SiO_2, EtOAc) to obtain (*S*)-7-benzyl-3-bromo-6,7-dihydro-5*H*-pyrrolo[3,4-*b*]pyridin-5-one (**3o**) (5.81 mg, 19.2 µmol, 77%, 93% *ee*) as a colorless solid.

TLC (Hex:EtOAc = 1:1): R_f = 0.22 [UV] [$KMnO_4$].

Specific Rotation: $[\alpha]_D^{25}$: –170 (*c* = 1.0, CH_2Cl_2) [93% *ee*].

Chiral HPLC: 93% *ee* (IC 250 × 4.6 mm, *n*-Hep/*iso*-PrOH = 70/30, 1 mL/min, λ = 210 nm); t_R = 6.90 min (minor, *ent*-**3o**), 8.81 min (major, **3o**).

(*S*)-7-Benzyl-3-chloro-6,7-dihydro-5*H*-pyrrolo[3,4-*b*]pyridin-5-one (*rac*-**3p**)

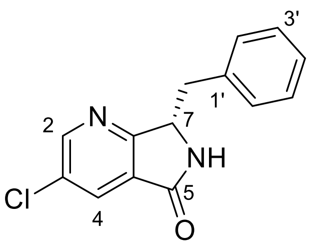

3p

$C_{14}H_{11}ClN_2O$

M = 258.71 g mol^{-1}

According to **GP I**, a degassed solution of *rac*-**3p** (6.47 mg, 25.0 µmol, 1.00 equiv.) and enantiomerically pure (–)-benzophenone (–)**-2b** (541 µg, 1.25 µmol, 5 mol%) in 10 mL α,α,α-trifluorotoluene was irradiated at λ = 350 nm for 13 hours. After irradiation, the solvent was evaporated and the residue was purified by FCC (SiO_2, EtOAc) to obtain (*S*)-7-benzyl-3-chloro-6,7-dihydro-5*H*-pyrrolo[3,4-*b*]pyridin-5-one (**3p**) (5.12 mg, 19.8 µmol, 79%, 97% *ee*) as a colorless solid.

TLC (Hex:EtOAc = 1:1): R_f = 0.24 [UV] [$KMnO_4$].

Specific Rotation: $[\alpha]_D^{25}$: –84 (*c* = 1.0, CH_2Cl_2) [97% *ee*].

Chiral HPLC: 97% *ee* (IC 250 × 4.6 mm, *n*-Hep/*iso*-PrOH = 70/30, 1 mL/min, λ = 210 nm); t_R = 6.56 min (minor, *ent*-**3p**), 8.42 min (major, **3p**).

(*S*)-7-Benzyl-3-phenyl-6,7-dihydro-5*H*-pyrrolo[3,4-*b*]pyridin-5-one (3q)

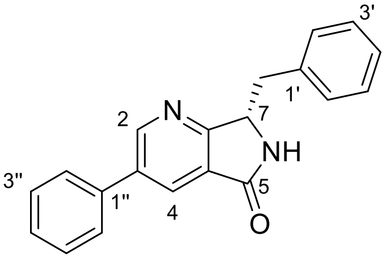

3q
$C_{20}H_{16}N_2O$
M = 300.36 g mol^{-1}

According to **GP I**, a degassed solution of *rac*-**3q** (7.51 mg, 25.0 µmol, 1.00 equiv.) and enantiomerically pure (–)-benzophenone (–)**-2b** (541 µg, 1.25 µmol, 5 mol%) in 10 mL α,α,α-trifluorotoluene was irradiated at λ = 350 nm for 13 hours. After irradiation, the solvent was evaporated and the residue was purified by FCC (SiO_2, EtOAc) to obtain (*S*)-7-benzyl-3-phenyl-6,7-dihydro-5*H*-pyrrolo[3,4-*b*]pyridin-5-one (**3q**) (6.40 mg, 21.3 µmol, 85%, 97% *ee*) as a colorless solid.

TLC (Hex:EtOAc = 1:1): R_f = 0.20 [UV] [$KMnO_4$].

Specific Rotation: $[\alpha]_D^{25}$: –128 (*c* = 1.0, CH_2Cl_2) [97% *ee*].

Chiral HPLC: 97% *ee* (AD-H 250 × 4.6 mm, *n*-Hep/*iso*-PrOH = 70/30, 1 mL/min, λ = 210 nm); t_R = 10.79 min (minor, *ent*-**3q**), 12.30 min (major, **3q**).

(*S*)-7-Isopropoxy-6,7-dihydro-5*H*-pyrrolo[3,4-*b*]pyridin-5-one (3r)

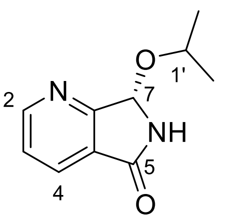

3r
$C_{10}H_{12}N_2O_2$
M = 192.22 g mol^{-1}

According to **GP I**, a degassed solution of *rac*-**3r** (4.81 mg, 25.0 µmol, 1.00 equiv.) and enantiomerically pure (–)-benzophenone (–)**-2b** (1.08 mg, 2.50 µmol, 10 mol%) in 10 mL α,α,α-trifluorotoluene was irradiated at λ = 350 nm for 13 hours. After irradiation, the solvent was evaporated and the residue was purified by FCC (SiO_2, EtOAc) to obtain (*S*)-7-isopropoxy-6,7-dihydro-5*H*-pyrrolo[3,4-*b*]pyridin-5-one (**3r**) (4.02 mg, 20.9 µmol, 84%, 94% *ee*) as a colorless solid.

TLC (EtOAc): R_f = 0.24 [UV] [$KMnO_4$].

Specific Rotation: $[\alpha]_D^{25}$: –88 (*c* = 1.0, CH_2Cl_2) [94% *ee*].

Chiral HPLC: 94% *ee* (IC 250 × 4.6 mm, *n*-Hep/*iso*-PrOH = 50/50, 1 mL/min, λ = 210 nm); t_R = 7.05 min (major, **3r**), 18.78 min (minor, *ent*-**3r**).

(*S*)-7-(Cyclopentyloxy)-6,7-dihydro-5*H*-pyrrolo[3,4-*b*]pyridin-5-one (3s)

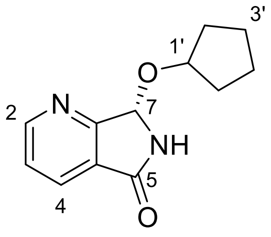

3s

$C_{12}H_{14}N_2O_2$

M = 218.26 g mol^{-1}

According to **GP I**, a degassed solution of *rac*-**3s** (5.46 mg, 25.0 µmol, 1.00 equiv.) and enantiomerically pure (–)-benzophenone (–)-**2b** (1.08 mg, 2.50 µmol, 10 mol%) in 10 mL α,α,α-trifluorotoluene was irradiated at λ = 350 nm for 13 hours. After irradiation, the solvent was evaporated and the residue was purified by FCC (SiO_2, Hex:EtOAc = 1:2) to obtain (*S*)-7-(cyclopentyloxy)-6,7-dihydro-5*H*-pyrrolo[3,4-*b*]pyridin-5-one (**3s**) (4.58 mg, 21.0 µmol, 84%, 94% *ee*) as a colorless solid.

TLC (EtOAc): R_f = 0.41 [UV] [$KMnO_4$].

Specific Rotation: $[a]_D^{25}$: –70 (*c* = 1.0, CH_2Cl_2) [94% *ee*].

Chiral HPLC: 94% *ee* (IC 250 × 4.6 mm, *n*-Hep/*iso*-PrOH = 50/50, 1 mL/min, λ = 210 nm); t_R = 6.83 min (major, **3s**), 21.13 min (minor, *ent*-**3s**).

(*S*)-7-(*tert*-Butoxy)-6,7-dihydro-5*H*-pyrrolo[3,4-*b*]pyridin-5-one (3t)

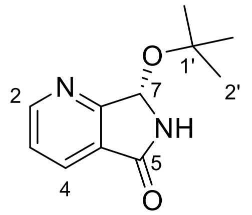

3t

$C_{11}H_{14}N_2O_2$

M = 206.25 g mol^{-1}

According to **GP I**, a degassed solution of *rac*-**3t** (5.16 mg, 25.0 µmol, 1.00 equiv.) and enantiomerically pure (–)-benzophenone (–)-**2b** (1.08 mg, 2.50 µmol, 10 mol%) in 10 mL α,α,α-trifluorotoluene was irradiated at λ = 350 nm for 13 hours. After irradiation, the solvent was evaporated and the residue was purified by FCC (SiO_2, Hex:EtOAc = 1:2) to obtain (*S*)-7-(*tert*-butoxy)-6,7-dihydro-5*H*-pyrrolo[3,4-*b*]pyridin-5-one (**3t**) (4.16 mg, 20.2 µmol, 81%, 99% *ee*) as a colorless solid.

TLC (Hex:EtOAc = 1:2): R_f = 0.31 [UV] [$KMnO_4$].

Specific Rotation: $[a]_D^{25}$: –20 (*c* = 1.0, CH_2Cl_2) [99% *ee*].

Chiral HPLC: 99% *ee* (AD-H 250 × 4.6 mm, *n*-Hep/*iso*-PrOH = 90/10, 1 mL/min, λ = 210 nm); t_R = 10.35 min (minor, *ent*-**3t**), 12.68 min (major, **3t**).

(*S*)- 7-(3-Chloro-2,2-dimethylpropoxy)-6,7-dihydro-5*H*-pyrrolo[3,4-*b*]pyridin-5-one (**3u**)

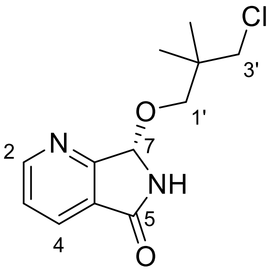

3u

$C_{12}H_{15}ClN_2O_2$

M = 254.71 g mol^{-1}

According to **GP I**, a degassed solution of *rac*-**3u** (6.37 mg, 25.0 µmol, 1.00 equiv.) and enantiomerically pure (–)-benzophenone (–)-**2b** (1.08 mg, 2.50 µmol, 10 mol%) in 10 mL α,α,α-trifluorotoluene was irradiated at λ = 350 nm for 13 hours. After irradiation, the solvent was evaporated and the residue was purified by FCC (SiO_2, Hex:EtOAc = 1:2) to obtain (*S*)- 7-(3-chloro-2,2-dimethylpropoxy)-6,7-dihydro-5*H*-pyrrolo[3,4-*b*]pyridin-5-one (**3u**) (5.60 mg, 22.0 µmol, 88%, 94% *ee*) as a colorless solid.

TLC (Hex:EtOAc = 1:2): R_f = 0.23 [UV] [$KMnO_4$].

Specific Rotation: $[\alpha]_D^{25}$: –10 (*c* = 1.0, CH_2Cl_2) [95% *ee*].

Chiral HPLC: 95% *ee* (AS-H 250 × 4.6 mm, *n*-Hep/*iso*-PrOH = 70/30, 1 mL/min, λ = 210 nm); t_R = 14.53 min (minor, *ent*-**3u**), 18.76 min (major, **3u**).

(*S*)-7-((*tert*-Butyldimethylsilyl)oxy)-6,7-dihydro-5*H*-pyrrolo[3,4-*b*]pyridin-5-one (**3v**)

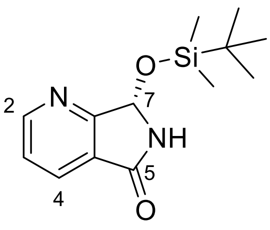

3v

$C_{13}H_{20}N_2O_2Si$

M = 264.40 g mol^{-1}

According to **GP I**, a degassed solution of *rac*-**3v** (6.61 mg, 25.0 µmol, 1.00 equiv.) and enantiomerically pure (–)-benzophenone (–)-**2b** (1.08 mg, 2.50 µmol, 10 mol%) in 10 mL α,α,α-trifluorotoluene was irradiated at λ = 350 nm for 13 hours. After irradiation, the solvent was evaporated and the residue was purified by FCC (SiO_2, Hex:EtOAc = 2:1 → 1:1) to obtain (*S*)-7-((*tert*-butyldimethylsilyl)oxy)-6,7-dihydro-5*H*-pyrrolo[3,4-*b*]pyridin-5-one (**3v**) (5.62 mg, 21.3 µmol, 85%, 99% *ee*) as a colorless solid.

TLC (Hex:EtOAc = 2:1): R_f = 0.31 [UV] [$KMnO_4$].

Specific Rotation: $[\alpha]_D^{25}$: +34 (*c* = 1.0, CH_2Cl_2) [99% *ee*].

Chiral HPLC: 99% *ee* (AD-H 250 × 4.6 mm, *n*-Hep/*iso*-PrOH = 90/10, 1 mL/min, λ = 210 nm); t_R = 6.09 min (major, **3v**), 6.62 min (minor, *ent*-**3v**).

(*S*)-7-((*S*)-2-Methylbutoxy)-6,7-dihydro-5*H*-pyrrolo[3,4-*b*]pyridin-5-one [(*3S*)-3w]

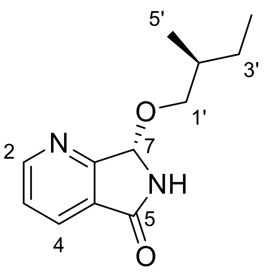

[(*3S*)-**3w**]
$C_{12}H_{16}N_2O_2$
M = 220.27g mol^{-1}

According to **GP I**, a degassed solution of *rac*-**3w** (5.51 mg, 25.0 µmol, 1.00 equiv.) and enantiomerically pure (–)-benzophenone (–)**-2b** (1.08 mg, 2.50 µmol, 10 mol%) in 10 mL α,α,α-trifluorotoluene was irradiated at λ = 350 nm for 13 hours. After irradiation, the solvent was evaporated and the residue was purified by FCC (SiO_2, Hex:EtOAc = 1:1) to obtain (*S*)-7-((*S*)-2-methylbutoxy)-6,7-dihydro-5*H*-pyrrolo[3,4-*b*]pyridin-5-one [(*3S*)-**3w**] (4.91 mg, 22.3 µmol, 89%, d.r. = 98/2) as a colorless solid.

TLC (Hex:EtOAc = 1:2): R_f = 0.48 [UV] [$KMnO_4$].

Specific Rotation: $[\alpha]_D^{25}$: –10 (*c* = 1.0, CH_2Cl_2) [d.r. = 98:2].

Chiral HPLC: d.r. = 98/2 (IC 250 × 4.6 mm, *n*-Hep/*iso*-PrOH = 70/30, 1 mL/min, λ = 210 nm); t_R = 8.04 min [major, (***3S***)**-3w**], 12.99 min [minor, (***3R***)**-3w**].

(*R*)-7-((*S*)-2-Methylbutoxy)-6,7-dihydro-5*H*-pyrrolo[3,4-*b*]pyridin-5-one [(*3R*)-3w]

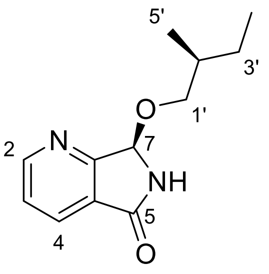

[(*3R*)-**3w**]
$C_{12}H_{16}N_2O_2$
M = 220.27g mol^{-1}

According to **GP I**, a degassed solution of *rac*-**3w** (5.51 mg, 25.0 µmol, 1.00 equiv.) and enantiomerically pure (+)-benzophenone (+)**-2b** (1.08 mg, 2.50 µmol, 10 mol%) in 10 mL α,α,α-trifluorotoluene was irradiated at λ = 350 nm for 13 hours. After irradiation, the solvent was evaporated and the residue was purified by FCC (SiO_2, Hex:EtOAc = 1:1) to obtain (*R*)-7-((*S*)-2-methylbutoxy)-6,7-dihydro-5*H*-pyrrolo[3,4-*b*]pyridin-5-one [(*3R*)-**3w**] (4.91 mg, 21.0 µmol, 84%, d.r. = 96/4) as a colorless solid.

TLC (Hex:EtOAc = 1:2): R_f = 0.48 [UV] [$KMnO_4$].

Specific Rotation: $[\alpha]_D^{25}$: +120 (*c* = 1.0, CH_2Cl_2) [d.r. = 96:4].

Chiral HPLC: d.r. = 96/4 (IC 250 × 4.6 mm, *n*-Hep/*iso*-PrOH = 70/30, 1 mL/min, λ = 210 nm); t_R = 8.06 min [minor, (***3S***)**-3w**], 13.02 min [major, (***3R***)**-3w**].

3-Benzyl-2,3-dihydro-1*H*-pyrrolo[3,4-*c*]pyridin-1-one (**12a**)

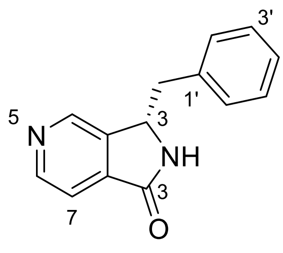

12a
$C_{14}H_{12}N_2O$
M = 224.26 g mol^{-1}

According to **GP I**, a degassed solution of *rac*-**12a** (5.61 mg, 25.0 µmol, 1.00 equiv.) and enantiomerically pure (–)-benzophenone (–)-**2b** (541 µg, 1.25 µmol, 5 mol%) in 10 mL α,α,α-trifluorotoluene was irradiated at λ = 350 nm for 13 hours. After irradiation, the solvent was evaporated and the residue was purified by FCC (SiO_2, EtOAc) to obtain (*S*)-3-benzyl-2,3-dihydro-1*H*-pyrrolo[3,4-*c*]pyridin-1-one (**12a**) (5.15 mg, 23.0 µmol, 92%, 23% *ee*) as a colorless solid.

TLC (EtOAc): R_f = 0.16 [UV] [$KMnO_4$].

Specific Rotation: $[\alpha]_D^{25}$: 22 (*c* = 1.0, CH_2Cl_2) [23% *ee*].

Chiral HPLC: 23% *ee* (AD-H 250 × 4.6 mm, *n*-Hep/*iso*-PrOH = 90/10, 1 mL/min, λ = 210 nm); t_R = 14.10 min (major, **12a**), 17.57 min (minor, *ent*-**12a**).

1-Benzyl-1,2-dihydro-3*H*-pyrrolo[3,4-*c*]pyridin-3-one (**12b**)

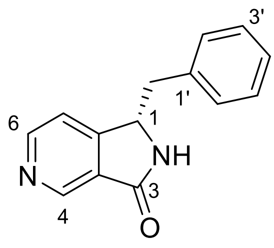

12b
$C_{14}H_{12}N_2O$
M = 224.26 g mol^{-1}

According to **GP I**, a degassed solution of *rac*-**12b** (5.61 mg, 25.0 µmol, 1.00 equiv.) and enantiomerically pure (–)-benzophenone (–)-**2b** (541 µg, 1.25 µmol, 5 mol%) in 10 mL α,α,α-trifluorotoluene was irradiated at λ = 350 nm for 13 hours. After irradiation, the solvent was evaporated and the residue was purified by FCC (SiO_2, EtOAc:MeOH = 95:5) to obtain (*S*)-1-benzyl-1,2-dihydro-3*H*-pyrrolo[3,4-*c*]pyridin-3-one (**12b**) (4.86 mg, 21.7 µmol, 87%, 98% *ee*) as a colorless solid.

TLC (EtOAc:MeOH = 95:5): R_f = 0.16 [UV] [$KMnO_4$].

Specific Rotation: $[\alpha]_D^{25}$: –46 (*c* = 1.0, CH_2Cl_2) [98% *ee*].

Chiral HPLC: 98% *ee* (AD-H 250 × 4.6 mm, *n*-Hep/*iso*-PrOH = 90/10, 1 mL/min, λ = 210 nm); t_R = 20.99 min (major, **12b**), 25.03 min (minor, *ent*-**12b**).

5-Benzyl-5,6-dihydro-7*H*-pyrrolo[3,4-*b*]pyridin-7-one (12c)

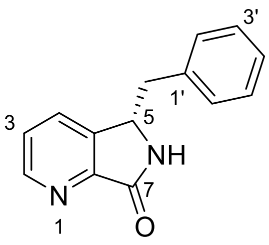

12c

$C_{14}H_{12}N_2O$

M = 224.26 g mol^{-1}

According to **GP I**, a degassed solution of *rac*-**12c** (5.61 mg, 25.0 µmol, 1.00 equiv.) and enantiomerically pure (–)-benzophenone (–)-**2b** (541 µg, 1.25 µmol, 5 mol%) in 10 mL α,α,α-trifluorotoluene was irradiated at λ = 350 nm for 13 hours. After irradiation, the solvent was evaporated and the residue was purified by FCC (SiO_2, EtOAc) to obtain (*S*)-5-benzyl-5,6-dihydro-7*H*-pyrrolo[3,4-*b*]pyridin-7-one (**12c**) (4.81 mg, 21.5 µmol, 86%, 82% *ee*) as a colorless solid.

TLC (EtOAc:): R_f = 0.18 [UV] [$KMnO_4$].

Specific Rotation: $[a]_D^{25}$: –70 (*c* = 1.0, CH_2Cl_2) [82% *ee*].

Chiral HPLC: 82% *ee* (AD-H 250 × 4.6 mm, *n*-Hep/*iso*-PrOH = 90/10, 1 mL/min, λ = 210 nm); t_R = 23.06 min (major, **12c**), 28.56 min (minor, *ent*-**12c**).

11. Downstream Synthetic Transformations

(*S*)-*tert*-Butyl 7-benzyl-5-oxo-5,7-dihydro-6*H*-pyrrolo[3,4-*b*]pyridine-6-carboxylate (4)

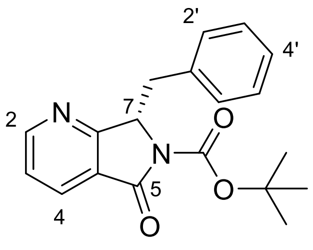

4

$C_{19}H_{20}N_2O_3$

M = 324.38 g mol^{-1}

According to a modified procedure,[2] *N*,*N*-diisopropylethylamine (227 µL, 173 mg, 1.34 mmol, 5.00 equiv.), di-tert-butyl dicarbonate (309 µL, 315 mg, 1.45 mmol, 5.40 equiv.) followed by DMAP (16.3 mg, 134 µmol, 0.50 equiv.) were added to a solution of substrate **3a** (60.0 mg, 268 µmol, 95% *ee*, 1.00 equiv.) in CH_2Cl_2 (300 µL) at room temperature. The resulting mixture was stirred for 20 minutes at the same temperature. Afterwards, the reaction was quenched by addition of sat. NH_4Cl (2 mL) and distilled water (2 mL). The aqueous layer was extracted with CH_2Cl_2 (3 × 20 mL), and the combined organic layers were washed with brine (10 mL), dried over Na_2SO_4. After filtration, the solvent was removed under reduced pressure and the residual crude product was subjected to FCC (SiO_2, Hex:EtOAc = 2:1) to yield the desired product (*S*)-*tert*-butyl 7-benzyl-5-oxo-5,7-dihydro-6*H*-pyrrolo[3,4-*b*]pyridine-6-carboxylate (**4**) (75 mg, 232 µmol, 86%, 95% *ee*) as a colorless oil.

TLC (Hex:EtOAc = 2:1): R_f = 0.29 [UV] [$KMnO_4$].

^{1}H-NMR (400 MHz, $CDCl_3$, 300 K): δ [ppm] = 8.85 (dd, ^3J = 4.9 Hz, ^4J = 1.6 Hz, 1H, H2), 7.90 (dd, ^3J = 7.8 Hz, ^4J = 1.2 Hz, 1H, H4), 7.33 (dd, ^3J = 7.8 Hz, ^3J = 4.9, 1H, H3), 7.08 – 6.95 (m, 3H, H2', H4', H6'), 6.68 – 6.61 (m, 2H, H3', H5'), 5.35 (dd, ^3J = 5.4 Hz, ^3J = 2.7 Hz, 1H), 3.70 (dd, ^2J = 13.8 Hz, ^3J = 5.4 Hz, 1H, C1-CH_a-C1'), 3.54 (dd, ^2J = 13.8 Hz, ^3J = 2.7 Hz, 1H, C1-CH_b-C1'), 1.66 [s, 9H, $(CH_3)_3$].

^{13}C-NMR (101 MHz, $CDCl_3$, 300 K): δ [ppm] = 164.8 (C5), 164.0 (C7a), 154.5 (C2), 150.2 (COO), 134.2 (C1'), 132.8 (C4), 129.7 (C3', C5'), 128.1 (C2', C6'), 126.9 (C4'), 125.4 (C4a), 123.7 (C3), 83.7 [$C(CH_3)_3$], 61.9 (C7), 36.8 (C1-CH_2-C1'), 28.3 [$C(CH_3)_3$].

HRMS (ESI) *m/z* $[M+H]^+$ calculated for $[C_{19}H_{21}N_2O_3]^+$: 325.1547; found: 325.1541.

IR (film): $\tilde{v}_{max}/cm^{-1}$ = 3030 (m, CH_{arom}), 2979 (m, CH_{aliph}), 2860 (m, CH_{aliph}), 1780 (s, C=O), 1747 (s, C=O), 1591 (m, C=Car), 1313 (s, CN), 1154 (s, CO).

Specific Rotation: $[\alpha]_D^{25}$: −54 (c = 1.0, CH_2Cl_2) [95% *ee*].

Chiral HPLC: 95% *ee* OJ-H 250 × 4.6 mm, *n*-Hep/*iso*-PrOH = 90/10, 1 mL/min, λ = 210 nm); t_R = 8.28 min (minor, *ent*-**4**), 9.52 min (major, **4**).

***S*)-2-(1-((*tert*-Butoxycarbonyl)amino)-2-phenylethyl)nicotinic acid (5)**

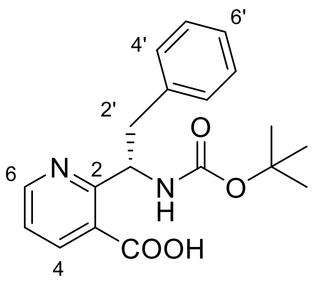

5
$C_{19}H_{22}N_2O_4$
M = 342.40 g mol^{-1}

According to a modified procedure,[2] 1 M aqueous lithium hydroxide solution (11.1 mg, 462 µL, 462 mmol, 2.50 equiv.) was added to a solution of (*S*)-*tert*-butyl 7-benzyl-5-oxo-5,7-dihydro-6*H*-pyrrolo[3,4-*b*]pyridine-6-carboxylate (**4**) (60.0 mg, 185 µmol, 95% *ee*, 1.00 equiv.) in THF (900 µL) at room temperature. The resulting mixture was stirred vigorously for 10 min at the same temperature. Afterwards, the reaction was quenched by the addition of sat. NH_4Cl (1 mL). The pH value of the mixture was adjusted to 2-3 by addition of aqueous 1 M HCl and the mixture was extracted with CH_2Cl_2 (3 × 15 mL). The combined organic layers were washed with brine (10 mL) and dried over Na_2SO_4. After filtration, the solvent was removed under reduced pressure to yield the desired (*S*)-2-(1-((*tert*-butoxycarbonyl)amino)-2-phenylethyl)nicotinic acid (**5**) (41.0 mg, 120 µmol, 65%) as a white solid.

M.p.: 195 °C.

^{1}H-NMR (400 MHz, DMSO-d$_6$, 300 K): δ [ppm] = 13.47 (s, 1H, COOH), 8.71 (dd, ^3J = 4.9 Hz, ^4J = 1.8 Hz, 1H, H6), 8.20 (dd, ^3J = 7.9 Hz, ^4J = 1.8 Hz, 1H, H4), 7.42 (dd, ^3J = 7.9 Hz, 4.7 Hz, 1H, H5), 7.38 – 7.12 (m, 5H, H4’, H5’, H6’, H7’, H8’), 5.78 – 5.64 (m, 1H, H1’), 2.98 (dd, ^2J = 13.5 Hz, ^3J = 3.9 Hz, 1H, H$_a$2’), 2.82 (dd, ^2J = 13.5 Hz, ^3J = 9.9 Hz, 1H, H$_b$2’), 1.23 [s, 9H, $(CH_3)_3$].

^{13}C-NMR (101 MHz, DMSO-d$_6$, 300 K): δ [ppm] = 167.4 (COOH), 161.9 (C3), 155.0 (COON), 151.5 (C6), 138.8 (C3’), 138.3 (C4), 129.3 (C4’, C8’), 127.8 (C5’, C7’), 126.0 (C6’), 125.0 (C2), 122.1 (C5), 77.6 [*C*$(CH_3)_3$], 54.0 (C1’), 40.8 (C2’), 28.1 [C(*C*$H_3)_3$].

HRMS (ESI) *m/z* [M+H]$^+$ calculated for $[C_{19}H_{23}N_2O_4]^+$: 343.1652; found: 343.1649.

IR (film): $\tilde{v}_{max}$/cm^{-1} = 3134 (bs, COO), 3034 (m, CH$_{arom}$), 2979 (m, CH$_{aliph}$), (s, C=O), 1724 (s, C=O), 1526 (m, C=C$_{arom}$), 1134 (s, CN).

tert-Butyl (_S_)-(1-(3-(hydroxymethyl)pyridin-2-yl)-2-phenylethyl)carbamate (6)

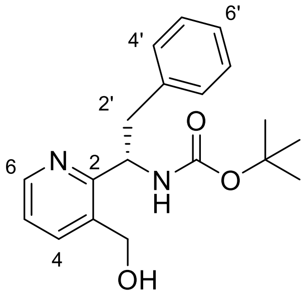

6

$C_{19}H_{24}N_2O_3$

M = 328.41 g mol^{-1}

According to a modified procedure,[22] triethylamine (19.5 µL, 14.2 mg, 140 µmol, 1.20 equiv.) and isobutyl chloroformate (18.2 µL, 19.5 mg, 140 µmol, 1.20 equiv.) were added to a suspension of (*S*)-2-(1-((*tert*-butoxycarbonyl)amino)-2-phenylethyl)nicotinic acid (**5**) (40.0 mg, 117 µmol, 1.00 equiv.) in THF (560 µL) at 0 °C upon which the suspension became clear. The resulting mixture was stirred for 15 min at the same temperature. Subsequently, the mixture was filtrated over a plug of Celite and cotton. The plug was rinsed with a small amount of dry THF and the resulting solution was transferred into a second flask. To this solution sodium borohydride (8.84 mg, 234 µmol, 2.00 equiv.) and one drop of water was added, and the resulting mixture was stirred for two hours at room temperature. Afterwards, the reaction was quenched by addition of water (2 mL) and sat. NH_4Cl (1 mL) and the mixture was extracted with EtOAc (3 × 15 mL). The combined organic layers were washed with brine (15 mL) and dried over Na_2SO_4. After filtration, the solvent was removed under reduced pressure and the residual crude product was subjected to FCC (SiO_2, Hex:EtOAc = 1:1) to yield the desired *tert*-butyl (*S*)-(1-(3-(hydroxymethyl)pyridin-2-yl)-2-phenylethyl)carbamate (**6**) (34.0 mg, 104 µmol, 97%, 93% *ee*) as a white solid.

TLC (Hex:EtOAc = 1:1): R_f = 0.55 [UV] [$KMnO_4$].

M.p.: 149 °C

^{1}H-NMR (400 MHz, $CDCl_3$, 300 K): δ [ppm] = 8.57 (dd, ^3J = 4.7 Hz, ^4J = 1.7 Hz, 1H, H6), 7.54 (dd, ^3J = 7.6, ^4J = 1.7 Hz, 1H, H4), 7.21 – 7.10 (m, 4H, H5, H4', H6', H8'), 6.96 (m, 2H, H5', H7'), 5.69 (d, J = 8.6 Hz, 1H, NH), 5.18 (m, 1H, H1'), 4.52 (dd, ^2J = 12.7 Hz, ^3J = 3.6 Hz, 1H, C3CH$_a$), 4.09 (dd, ^2J = 12.8 Hz, ^3J = 8.6 Hz, 1H, C3CH$_b$), 3.29 (dd, J = 8.5, 3.9 Hz, 1H, OH), 3.24 – 3.04 (m, 2H, H2'), 1.37 [s, 9H, $(CH_3)_3$].

^{13}C-NMR (101 MHz, $CDCl_3$, 300 K): δ [ppm] = 158.2 (C2), 156.1 (COO), 149.1 (C6), 137.5 (C3'), 137.3 (C4), 134.1 (C3), 129.5 (C5', C7'), 128.5 (C4', C8'), 126.7 (C6'), 122.8 (C5), 80.2 [*C*$(CH_3)_3$], 61.7 (CH_2OH), 52.7 (C1'), 42.2 (C2'), 28.5 [C(CH$_3$)$_3$].

HRMS (ESI) *m/z* [M+H]$^+$ calculated for $[C_{19}H_{23}N_2O_4]^+$: 329.1860; found: 329.1856.

IR (film): $\tilde{v}_{max}$/cm^{-1} = 3424 (bm, NH), 3226 (bm, OH), 3065 (m, CH$_{arom}$), 2974 (m, CH$_{aliph}$), 1677 (s, C=O), 1532 (s, NH), 1043 (s, CO).

Specific Rotation: $[\alpha]_D^{25}$: +26 (c = 1.0, CH_2Cl_2) [93% *ee*].

Chiral HPLC: 93% *ee* AD-H 250 × 4.6 mm, *n*-Hep/*iso*-PrOH = 90/10, 1 mL/min, λ = 210 nm); t_R = 8.04 min (major, **6**), 12.78 min (minor, *ent*-**6**).

(*S*)-7-(Cyclobutylmethyl)-1,2,3,4,6,7-hexahydro-5*H*-pyrrolo[3,4-*b*]pyridin-5-one (7)

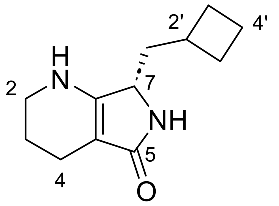

7

$C_{12}H_{18}N_2O$

M = 206.29 g mol^{-1}

According to a modified procedure,[23] ^{Cy}(CAAC)Rh(cod)Cl (2.15 mg, 3.75 µmol, 2.5 mol%), substrate **3g** (30.4 mg, 150 µmol, 99% *ee*, 1.00 equiv.) and powdered molecular sieves (25.0 mg) were filled in a dried screw cap vial. 2,2,2 Trifluoroethanol (1.50 mL) was added under argon atmosphere. The vial was placed in a 60 mL stainless steel autoclave under argon atmosphere. The autoclave was pressurized and depressurized with hydrogen gas three times before the hydrogen pressure was set to 5.0 MPa. The reaction mixture was stirred at 50 °C for 24 h. Afterwards, the autoclave was carefully depressurized. The crude mixture was filtered over Celite and rinsed several times with EtOAc:MeOH, 9:1. After removal of all volatiles under reduced pressure, the crude material was purified by flash column chromatography (SiO_2, EtOAc/MeOH = 1:0 → 9:1) to yield the desired product (*S*)-7-(cyclobutylmethyl)-1,2,3,4,6,7-hexahydro-5*H*-pyrrolo[3,4-*b*]pyridin-5-one (**7**) (24.9 mg, 121 µmol, 81%, 99% *ee*) as a white solid.

TLC (EtOAc:MeOH = 95:5): R_f = 0.18 [UV] [$KMnO_4$].

M.p.: 187 °C

^{1}H-NMR (400 MHz, MeOH-d4, 300 K): δ [ppm] = 3.92 (dd, ^3J = 7.3 Hz, ^3J = 3.7 Hz, 1H, H7), 3.25 (*virt.* q, ^3J = 5.2 Hz, 2H, H2), 2.46 – 2.35 (m, 1H, H2’), 2.18 (t, ^3J = 6.2 Hz, 2H, H4), 2.09 – 1.99 (m, 2H, H3), 1.93 – 1.68 (m, 6H, H_b1’, H3, H3’, H4’, H5’, NH), 1.59 (dd, ^3J = 13.9 Hz, ^3J = 6..9 Hz, 1H, H_b1’).

^{13}C-NMR (101 MHz, MeOH-d4, 300 K): δ [ppm] = 178.5 (C5), 165.1 (C7a), 96.5 (C4a), 56.0 (C7), 42.3 (C2), 40.7 (C1’), 33.5 (C2’), 30.1 (C4), 29.6 (C3), 22.3 (C3’), 19.7 (C5’), 18.6 (C4).

HRMS (ESI) *m/z* $[M+H]^+$ calculated for $[C_{12}H_{19}N_2O]^+$: 207.1492 found: 207.1490.

IR (film): $\tilde{v}_{max}/cm^{-1}$ = 3170 (bm, NH), 2970 (m, CH_{aliph}), 2952 (m, CH_{aliph}), 2847 (m, CH_{aliph}), 1624 (s, C=O), 1395 (s, CN).

Specific Rotation: $[\alpha]_D^{25}$: –24 (*c* = 1.0, CH_2Cl_2) [99% *ee*].

Chiral HPLC: 99% *ee* AS-H 250 × 4.6 mm, *n*-Hep/*iso*-PrOH = 50/50, 1 mL/min, λ = 210 nm); t_R = 7.63 min (major, **7**), 16.98 min (minor, *ent*-**7**).

(*S*)-7-Benzyl-2-(cyclohex-1-en-1-yl)-6,7-dihydro-5*H*-pyrrolo[3,4-*b*]pyridin-5-one (3x)

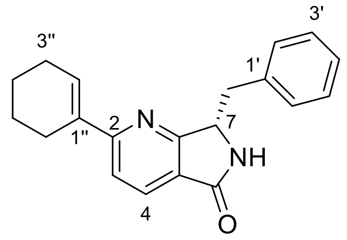

3x

$C_{20}H_{20}N_2O$

M = 304.39 g mol^{-1}

According to **GP H**, cyclohexen-1-ylboronic acid (31.2 mg, 247 µmol, 1.50 equiv.), K_2CO_3 (45.6 mg, 330 µmol, 2.00 equiv.), $PdCl_2(PPh_3)_2$ (11.6 mg, 16.5 µmol, 10 mol%) and PCy_3 (9.25 mg, 33.0 µmol, 20 mol%) were added to a solution of (*S*)-7-benzyl-2-bromo-6,7-dihydro-5*H*-pyrrolo[3,4-*b*]pyridin-5-one (**3l**) (50.0 mg, 165 µmol, 91% *ee*, 1.00 equiv.) in THF/water (4 mL, 3:1). The resulting mixture was degassed by freeze-pump thaw cycling thrice and then stirred at 60 °C for three hours. Afterwards, the mixture was allowed to cool to room temperature, water (10 mL) was added, and the aqueous layer was extracted with EtOAc (3 × 20 mL). The combined organic layers were washed with brine (20 mL) and dried over Na_2SO_4. After filtration, the solvent was removed under reduced pressure and the residual crude product was subjected to FCC (SiO_2, Hex:EtOAc = 1:1) to yield the desired product (*S*)-7-benzyl-2-(cyclohex-1-en-1-yl)-6,7-dihydro-5*H*-pyrrolo[3,4-*b*]pyridin-5-one (**3x**) (44.5 mg, 146 µmol, 86% *ee*, 89%) as a colorless solid.

TLC (Hex:EtOAc = 1:1): R_f = 0.28 [UV] [$KMnO_4$].

M.p.: 182 °C

^{1}H-NMR (400 MHz, $CDCl_3$, 300 K): δ [ppm] = 8.00 (d, ^3J = 8.1 Hz, 1H, H4), 7.45 (d, ^3J = 8.1 Hz, 1H, H3), 7.34 – 7.29 (m, 2H, H3’. H5’), 7.28 – 7.22 (m, 3H, H2’, H4’, H6’), 6.95 (tt, ^3J = 4.0 Hz, ^4J = 1.7 Hz, 1H), 6.24 (s, 1H, NH), 4.77 (dd, ^3J = 9.9 Hz, ^3J = 3.6 Hz, 1H), 3.63 (dd, ^2J = 13.8 Hz, 3.6 Hz, 1H, C7-CH_a-C1’), 2.68 (dd, ^2J = 13.7 Hz, ^3J = 9.9 Hz, 1H, C1-CH_b-C1’), 2.64 – 2.49 (m, 2H, H6’), 2.40 – 2.30 (m, 2H, H3’), 1.89 – 1.80 (m, 2H, H5’), 1.77 – 1.63 (m, 2H, H4’).

^{13}C-NMR (101 MHz, $CDCl_3$, 300 K): δ [ppm] = 168.7 (C2), 165.9 (C5), 162.7 (C7a), 137.3 (C1’), 136.5 (C1’’), 132.3 (C4), 131.9 (C2’’), 129.3 (C2’, C6’), 129.0 (C3’, C5’), 127.2 (C4’), 123.2 (C4a), 118.8 (C3), 59.8 (C7), 39.9 (C1-*C*H_2-C1’), 26.3 (C3’’/C6’’), 26.2 (C3’’/6’’), 22.9 (C5’’), 22.1 (C4’’).

HRMS (ESI) *m/z* [M+H]$^+$ calculated for $[C_{20}H_{21}N_2O]^+$: 305.1648 found: 305.1644.

IR (film): $\tilde{v}_{max}/cm^{-1}$ = 3198 (bm, NH), 3072 (m, CH_{arom}), 2926 (m, CH_{aliph}), 2853 (m, CH_{aliph}), 1707 (s, C=O), 1597 (m, $C{=}C_{arom}$).

Specific Rotation: $[\alpha]_D^{25}$: –120 (*c* = 1.0, CH_2Cl_2) [89% *ee*].

Chiral HPLC: 98% *ee* IA 250 × 4.6 mm, *n*-Hep/*iso*-PrOH = 90/10, 1 mL/min, λ = 210 nm); t_R = 10.97 min (major, **3x**), 21.00 min (minor, *ent*-**3x**).

12. Isolation and Analysis of 11a and 11p

Isolation of 7-Benzyl-7-(hydroxy(4-methoxyphenyl)(2-((7*S*)-1,5,7-trimethyl-2-oxo-3-azabicyclo[3.3.1]nonan-7-yl)benzo[*d*]oxazol-7-yl)methyl)-6,7-dihydro-5*H*-pyrrolo[3,4-*b*]pyridin-5-one (11a)

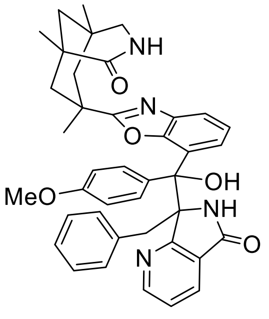

11a
$C_{40}H_{40}N_4O_5$
M = 656.7830 g mol^{-1}

A degassed solution of *rac*-**3a** (44.85 mg, 200 µmol, 4.00 equiv.) and enantiomerically pure (–)-benzophenone (–)-**2b** (21.6 mg, 50.0 µmol, 1.00 equiv.) in 80 mL α,α,α-trifluorotoluene was irradiated at λ = 350 nm for one hour. After irradiation, the solvent was evaporated and the residue was purified by FCC (SiO_2, Hex/EtOAc= 1:1 → 0:1) to obtain 7-benzyl-7-(hydroxy(4-methoxyphenyl)(2-((7*S*)-1,5,7-trimethyl-2-oxo-3-azabicyclo[3.3.1]nonan-7-yl)benzo[*d*]oxazol-7-yl)methyl)-6,7-dihydro-5*H*-pyrrolo[3,4-*b*]pyridin-5-one (**11a**) (31.1 mg, 47.4 µmol, 95%) as a colorless semi solid.

HRMS (**ESI**) *m/z* $[M+H]^+$ calculated for $[C_{40}H_{40}N_4O_5]^+$: 657.3071 found: 657.3059.

As analysis by NMR was not applicable due formation of a mixture of diastereoisomers, infrared spectroscopy was used. While the benzophenone-based catalyst (–)-**2b** showed a characteristic band at 1645 cm^{-1} for aromatic carbonyl compounds (Figure S11),[24] the newly isolated compound did not show that band at 1645 cm^{-1}, but only the one stemming from the lactam of the substrate at 1693 cm^{-1}.

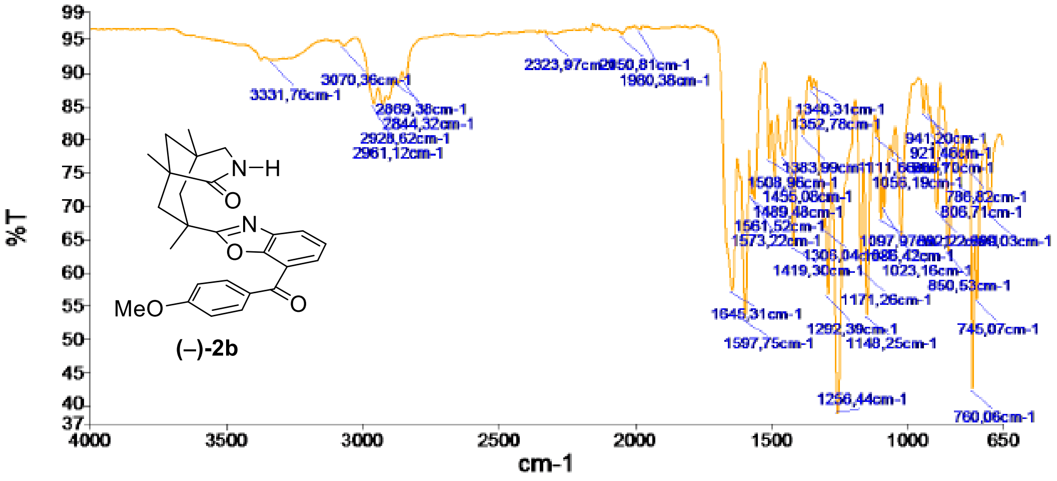

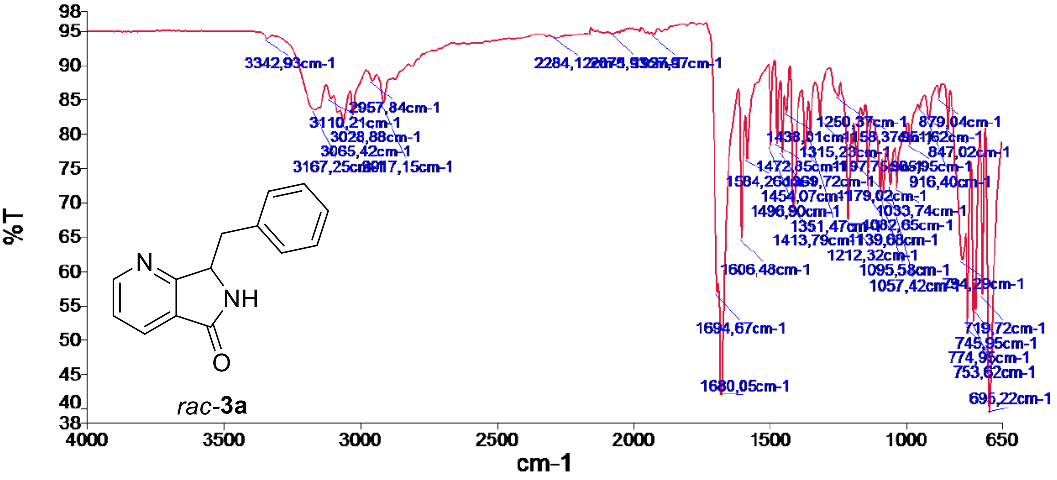

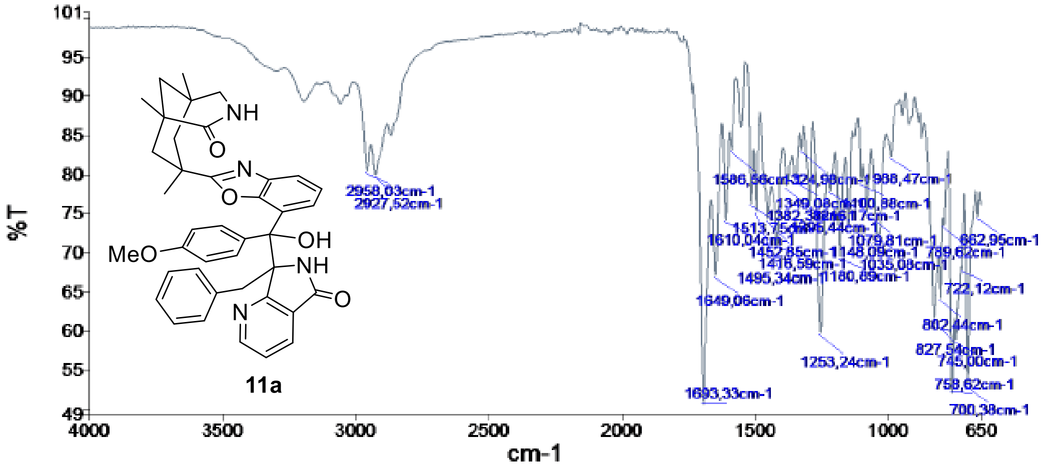

Figure S11: IR Spectra of Compounds (–)-**2b**, *rac*-**3a** and **11a**.

UV-VIS Analysis of 11a

The UV-Vis absorption spectrum of a 1 mM solution of **11a** and **SI-22** in CH_2Cl_2 was measured in *Hellma* precision cells made of quartz SUPRASIL® with a light pathway of 10 mm on a Perkin Elmer Lambda 35 UV-Vis spectrometer (Figure **S12**).

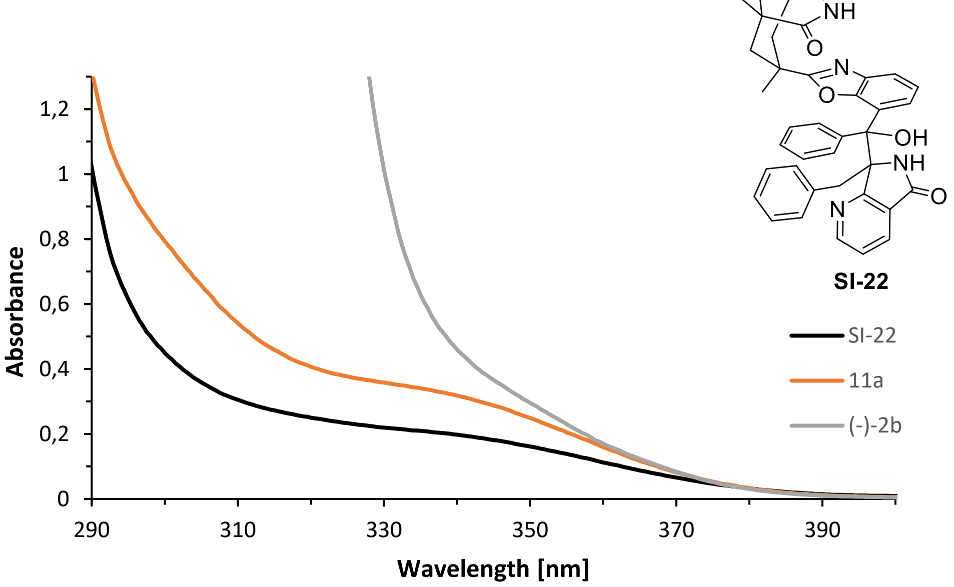

Figure S12: UV-Vis Spectra of **SI-22** (black), **11a** (orange) and (–)-**2b** (grey) in CH_2Cl_2, (c = 1mM), 10 mm pathway.

Scrambling experiment with 11a as replacement for (–)-2b

As compound **11a** was expected to be a catalytically active resting state in the photochemical reaction, a photochemical deracemization with **11**a as a replacement for benzophenone catalyst **(–)-2b** should also result in enrichment of one enantiomer if subjected to the reaction. Therefore, a degassed solution of *rac*-**3p** (38.8 mg, 150 µmol, 1.00 equiv.) and adduct **11a** (4.93 mg, 7.50 µmol, 5 mol%) in 60 mL α,α,α-trifluorotoluene was irradiated at λ = 350 nm for 13 hours. After irradiation, the solvent was evaporated and the residue was purified by FCC (SiO_2, Hex/EtOAc= 1:1 → 0:1) to obtain **3p** (32.8 mg, 127µmol, 85%, 90% *ee*) and the new species **11p** (4.10 mg, 5.93 µmol, 79% with respect to the amount of **11a**). Latter compound was detected by HRMS.

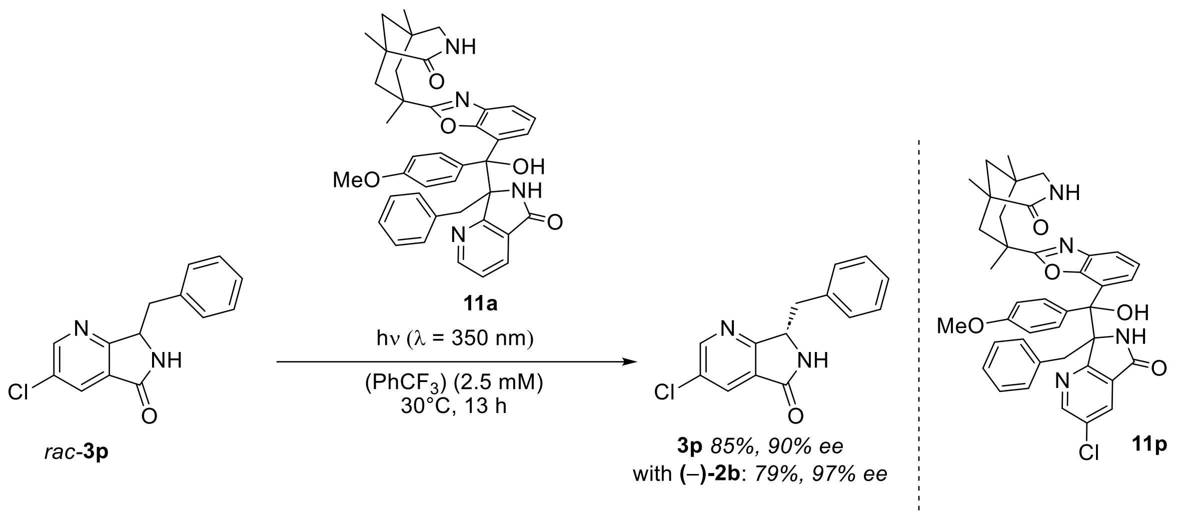

TLC (Hex:EtOAc = 1:1): R_f = 0.24 [UV] [$KMnO_4$].

Specific Rotation: $[\alpha]_D^{25}$: –78 (c = 1.0, CH_2Cl_2) [90% *ee*].

Chiral HPLC: 90% *ee* (IC 250 × 4.6 mm, *n*-Hep/*iso*-PrOH = 70/30, 1 mL/min, λ = 210 nm); t_R = 6.56 min (minor, *ent*-**3p**), 8.42 min (major, **3p**).

HRMS for 11p (ESI) *m/z* $[M+H]^+$ calculated for $[C_{40}H_{40}{}^{35}ClN_4O_5]^+$: 691.2682, found: 691.2670.

13. Transient Absorption Spectroscopy of Compound 11a

UV-Vis Spectroscopy

The absorption spectra before and after transient absorption (TA) measurements were recorded on a Lambda 365 UV–VIS spectrophotometer (PerkinElmer Instruments Inc.). The spectra were recorded in a Schlenk cuvette with a 500 µm pathway, made from quartz glass. All solutions were handled under dry argon.

Transient Absorption Spectroscopy

Femtosecond UV-TA experiments were performed using a home-built transient absorption setup detailed previously.[25,26] Briefly, a 5 kHz train of 800 nm, 36 fs FWHM laser pulses generated by a regenerative amplifier (Coherent Legend Elite Duo HE+) was divided into pump and probe arms. A temporal delay up to 1.5 ns with a minimum step size of 15 fs is achieved via optical path length variations using a mechanical delay stage (Newport, M-IMS300C) equipped with a broadband retroreflector (Newport, M-BBR2.5-5.0).
The pump wavelength of 323 nm is obtained by frequency doubling the output of a tunable NOPA.[27] The NOPA was used to generate 12 nm pulses full width at half maximum (FWHM) centered at 650 nm. The 323 nm pump pulse (5 nm FWHM) duration was estimated to 120 fs (FWHM) by fitting the instrument response function using the program package OPTIMUS.[28] The pump spot size was 250x180 µm, and the used pulse energy was 92 nJ.
A whitelight supercontinuum was generated by focusing 800 nm light into a translated 5 mm thick CaF_2 crystal using a lens with a focal length of 100 mm. The resulting probing range was 390 to 750 nm. The whitelight was split into a reference and a signal part to allow to account for fluctuations.[29] The signal beam was focused into the sample by using a 150 mm focal length spherical mirror. The spot size at the sample position was ~60 µm. The probe spatially overlaps with the pump and is recollimated after the sample using an achromatic lens with a 75 mm focal length. The probe and reference laser pulses were detected using a home-built prism spectrometer in combination with a pair of high-speed CMOS linear-array cameras (Glaz LineScan-I-Gen2, Synertronic Designs). The relative polarization between the pump and the probe pulses was set at magic angle (MA) by using a waveplate in the pump arm (300-470 nm $\lambda/2$ plate, B.Halle Nachfl. GmbH). A sketch of the setup is shown in Figure S13. The measurement was performed in DCM under argon in a Schlenk cuvette with 0.5 mm thickness with an optical density below 0.3 at the excitation wavelength. Figure S14 shows the absorption spectrum of **11a** in DCM together with the used pump spectrum.

To extract lifetimes, we performed a multi-exponential fit (global analysis) using OPTIMUS.[27] Under the assumption of sequential energy relaxation between states, this allows us to extract the spectral shapes (evolution-associated spectra, EAS) and lifetimes of each participating species.

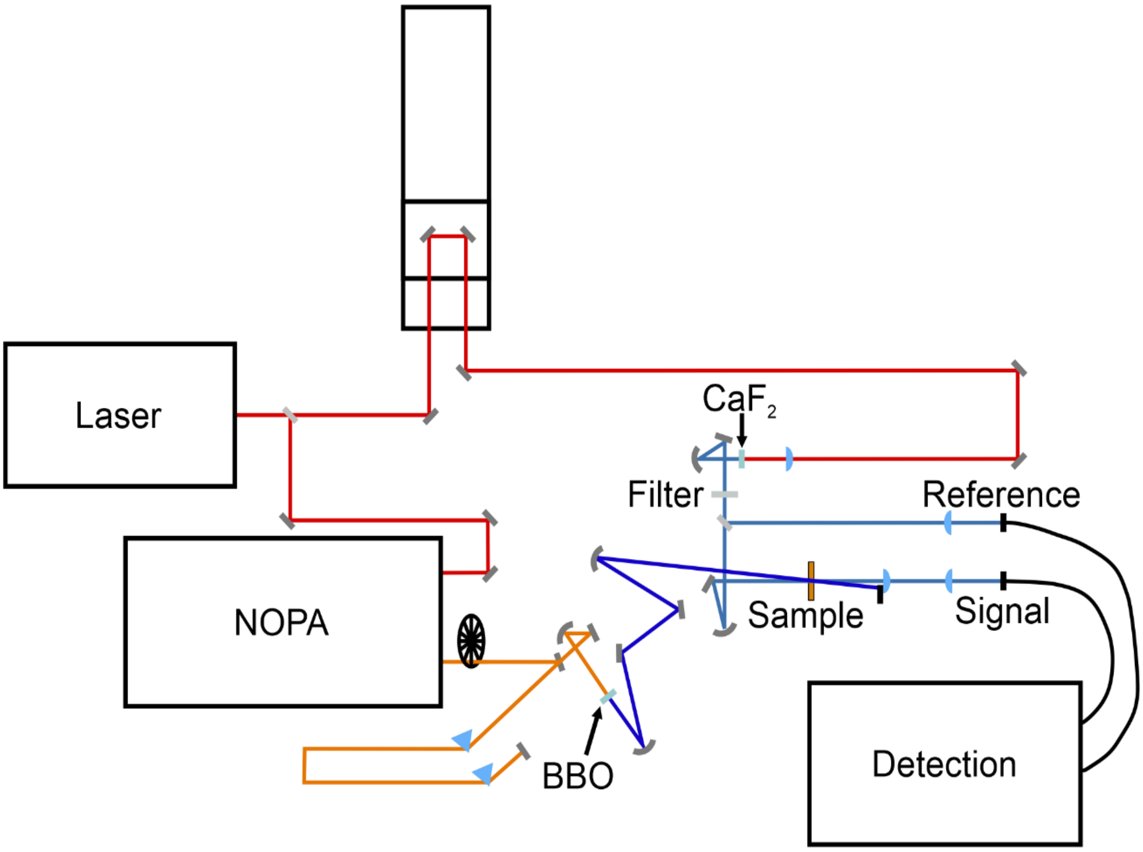

Figure S13: Simplified TA setup where the output from the laser is split into a pump and probe arm. In the former, the fundamental laser pulse pumps a non-collinear parametric amplifier, the output of which is compressed by a prism pair. The compressed pump pulse is then frequency-doubled in a BBO and focused into the sample (orange/blue path). The probe beam passes a delay stage before seeding whitelight generation in CaF_2. The whitelight (blue path) is split into two beams for referencing and probing. The latter is focused into the sample cell (orange) and then detected.

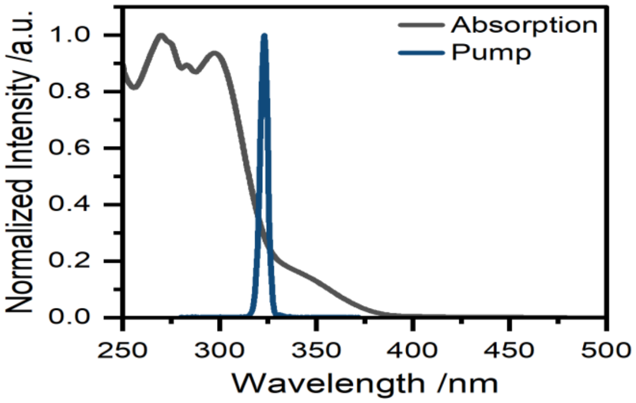

Figure S14: Absorption spectrum of **11a** in DCM (black) and the pump spectrum used for the TA measurement (blue).

14. NMR Spectra

2-Methoxy-3-nitrobenzaldehyde (SI-5)

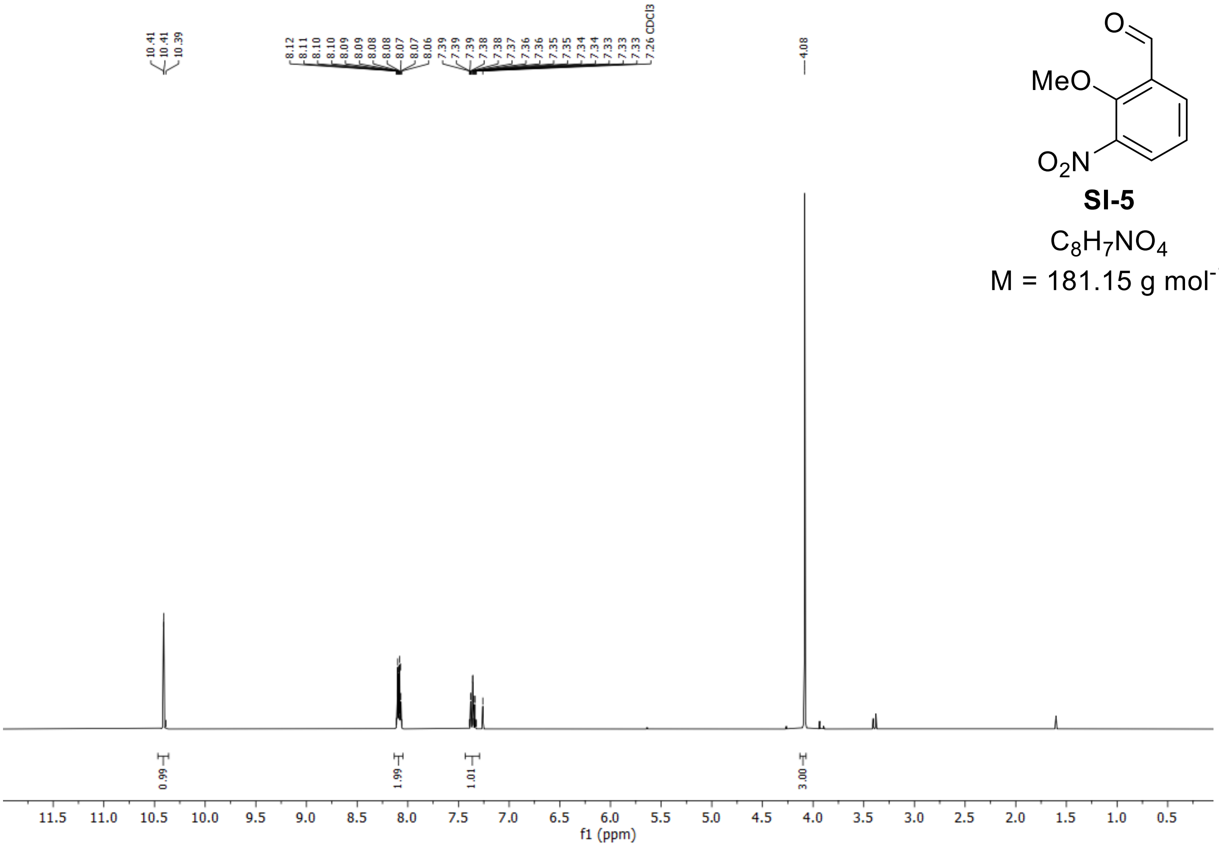

SI-5

$C_8H_7NO_4$

M = 181.15 g mol^{-1}

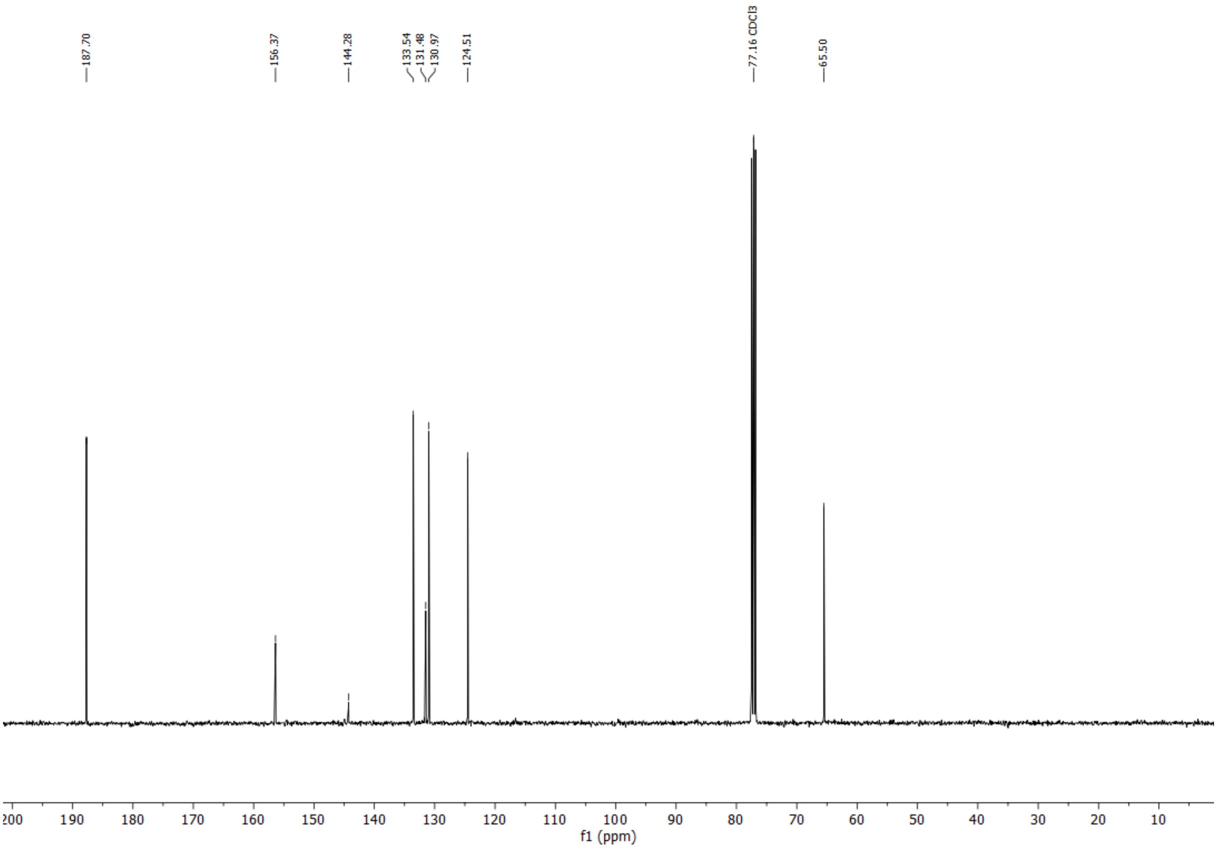

2-Methoxy-3-nitrophenyl)(4-methoxyphenyl)methanol (SI-6)

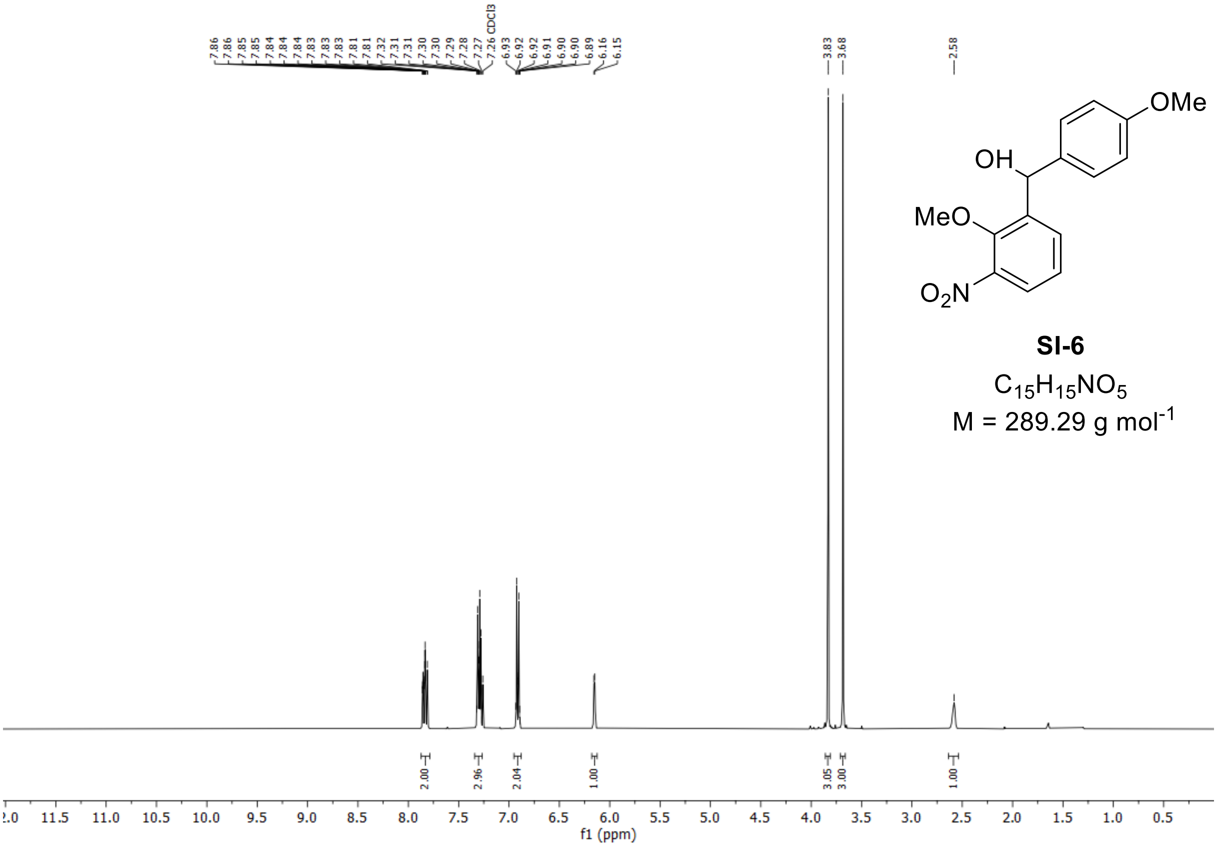

SI-6

$C_{15}H_{15}NO_5$

M = 289.29 g mol^{-1}

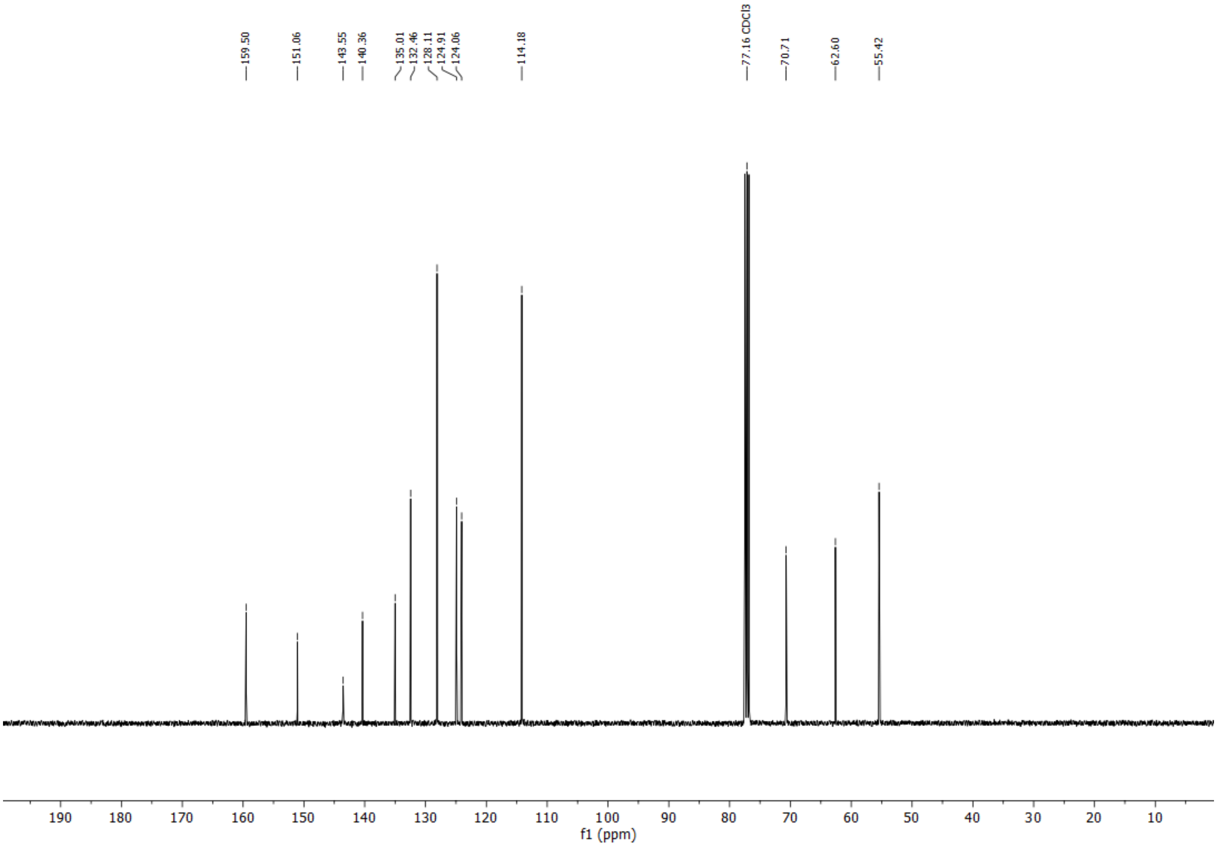

(2-Methoxy-3-nitrophenyl)(4-methoxyphenyl)methanone (SI-7)

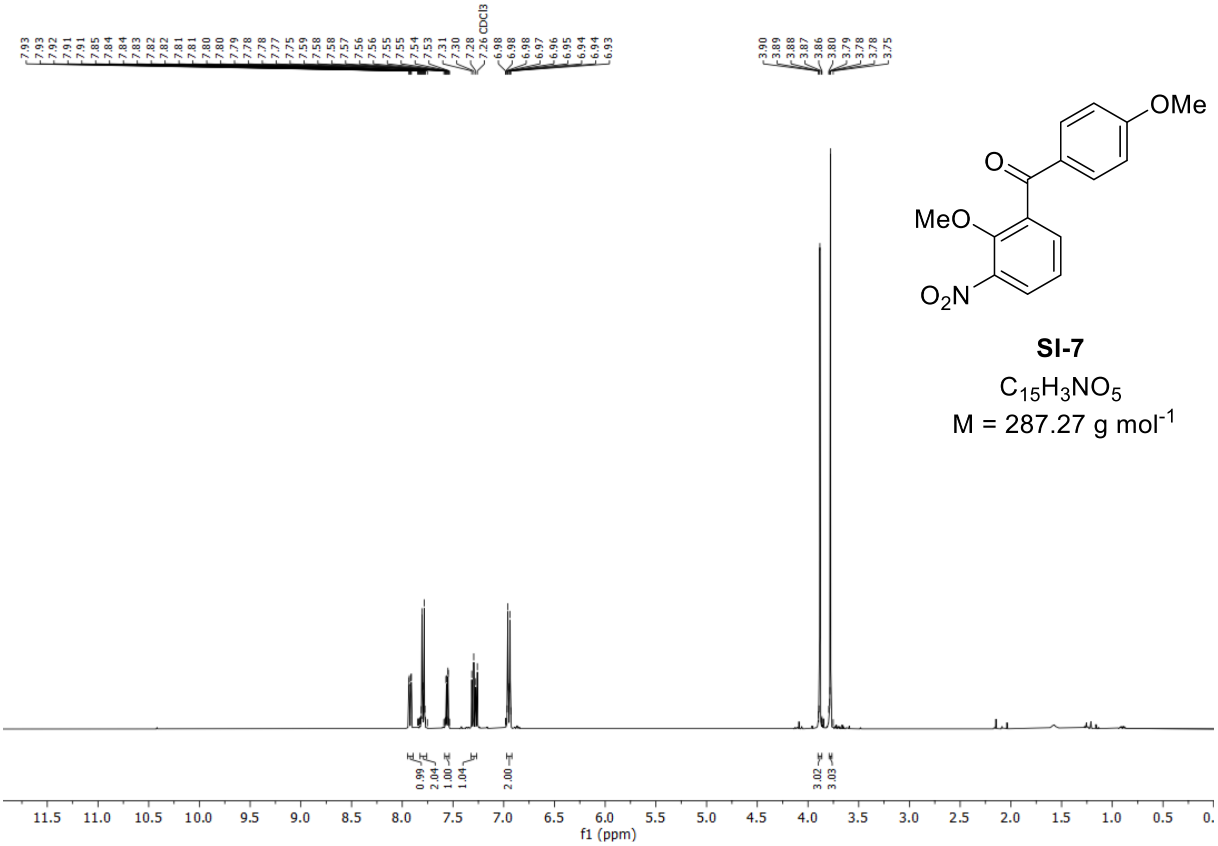

SI-7

$C_{15}H_3NO_5$

M = 287.27 g mol^{-1}

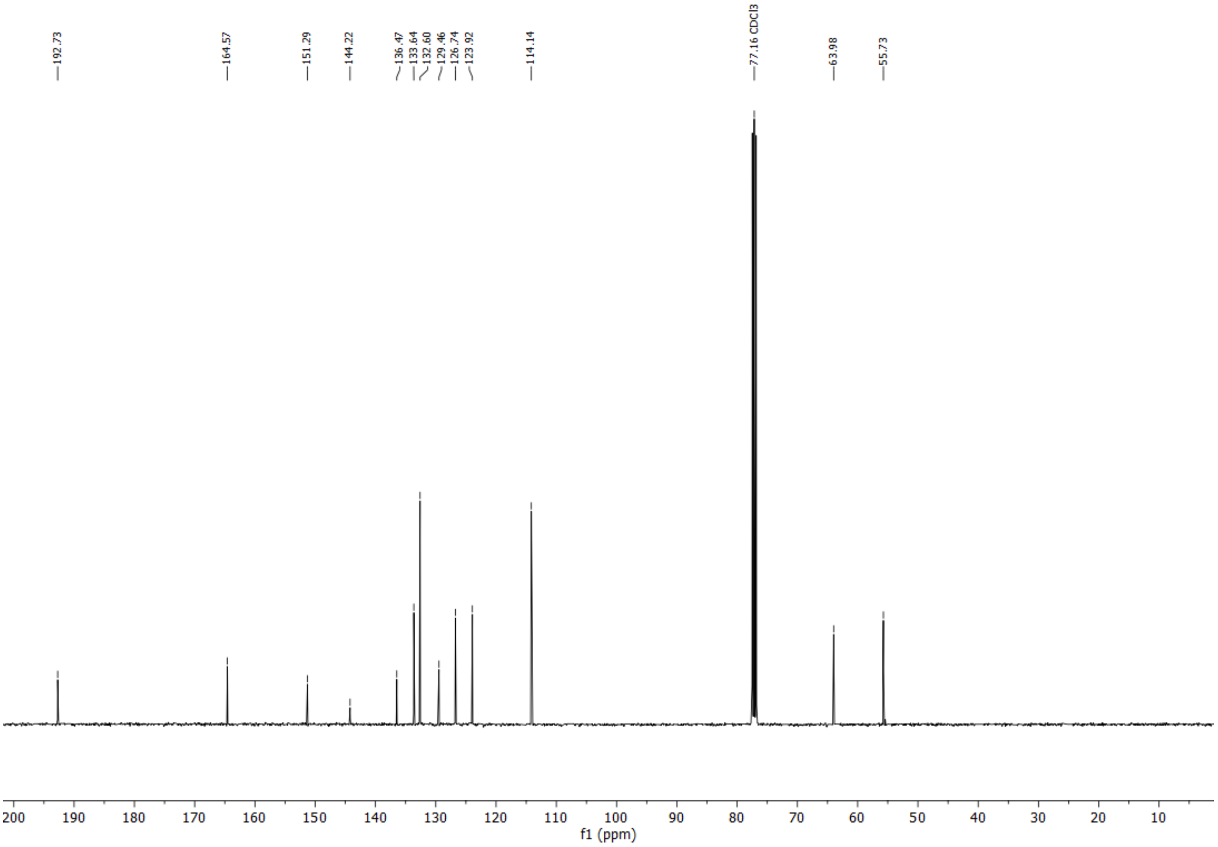

(2-Hydroxy-3-nitrophenyl)(4-methoxyphenyl)methanone (SI-8)

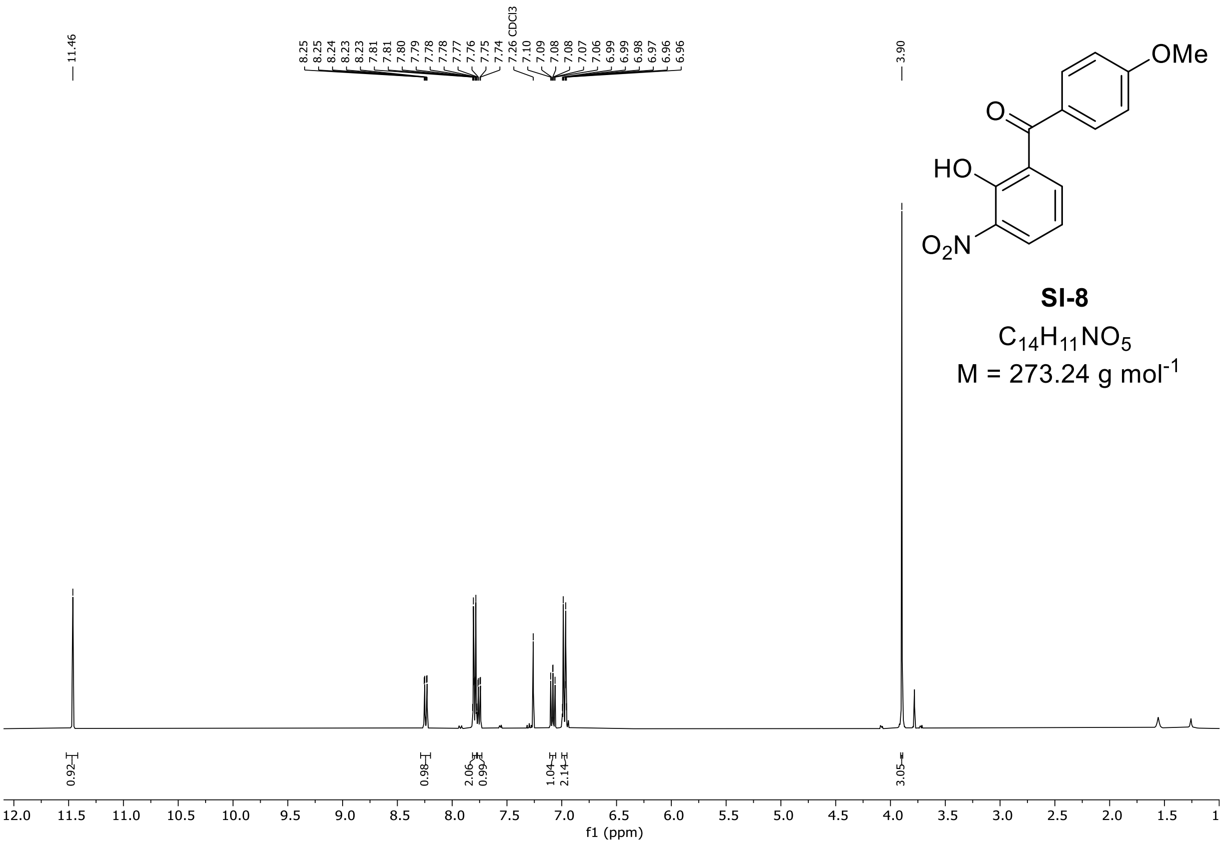

SI-8

$C_{14}H_{11}NO_5$

M = 273.24 g mol^{-1}

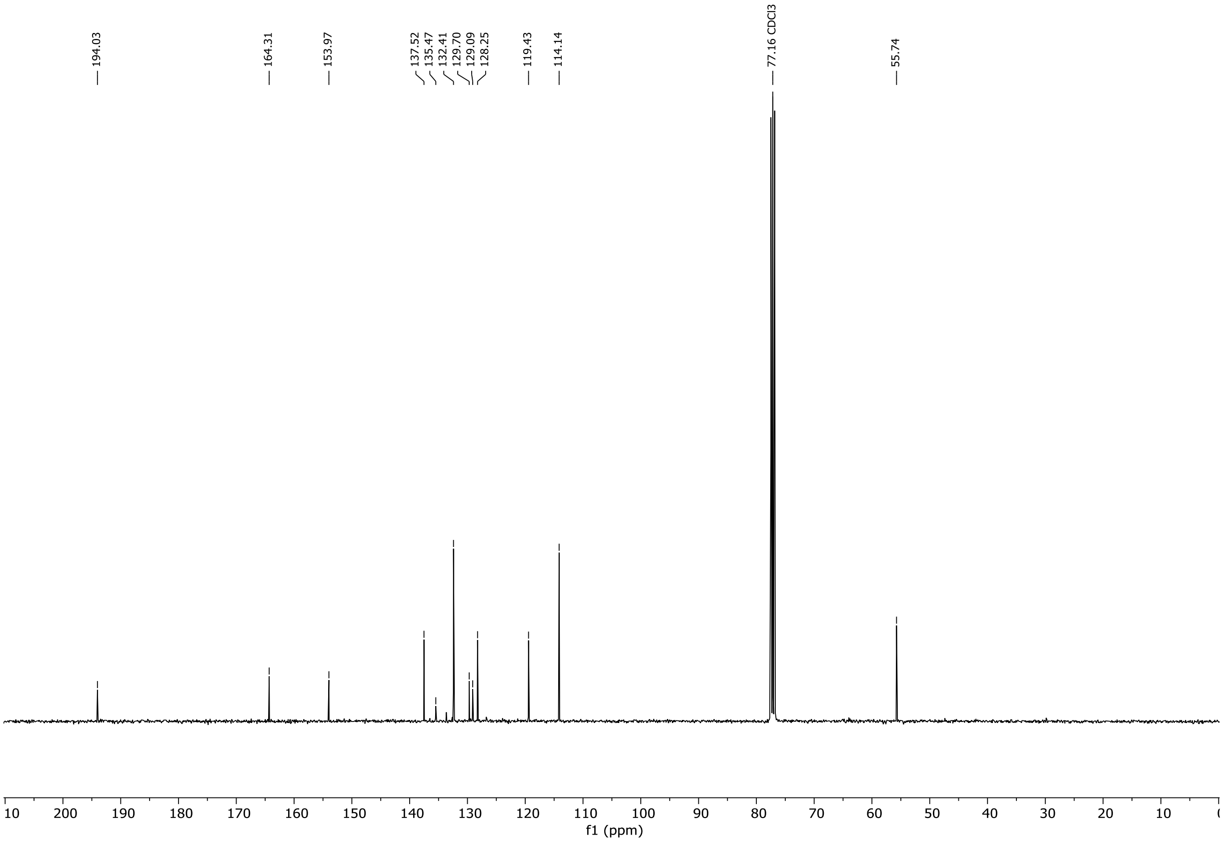

(3-Amino-2-hydroxyphenyl)(4-methoxyphenyl)methanone (SI-9)

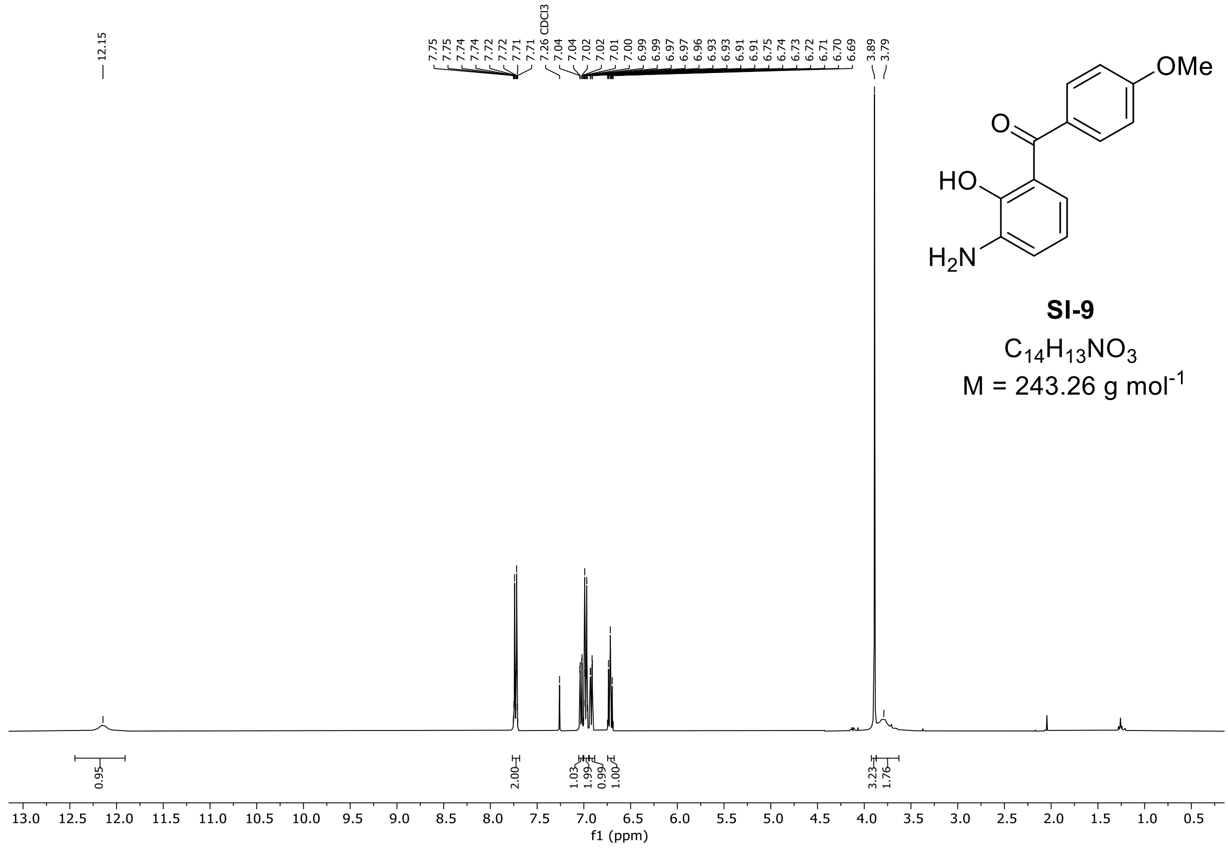

SI-9

$C_{14}H_{13}NO_3$

M = 243.26 g mol^{-1}

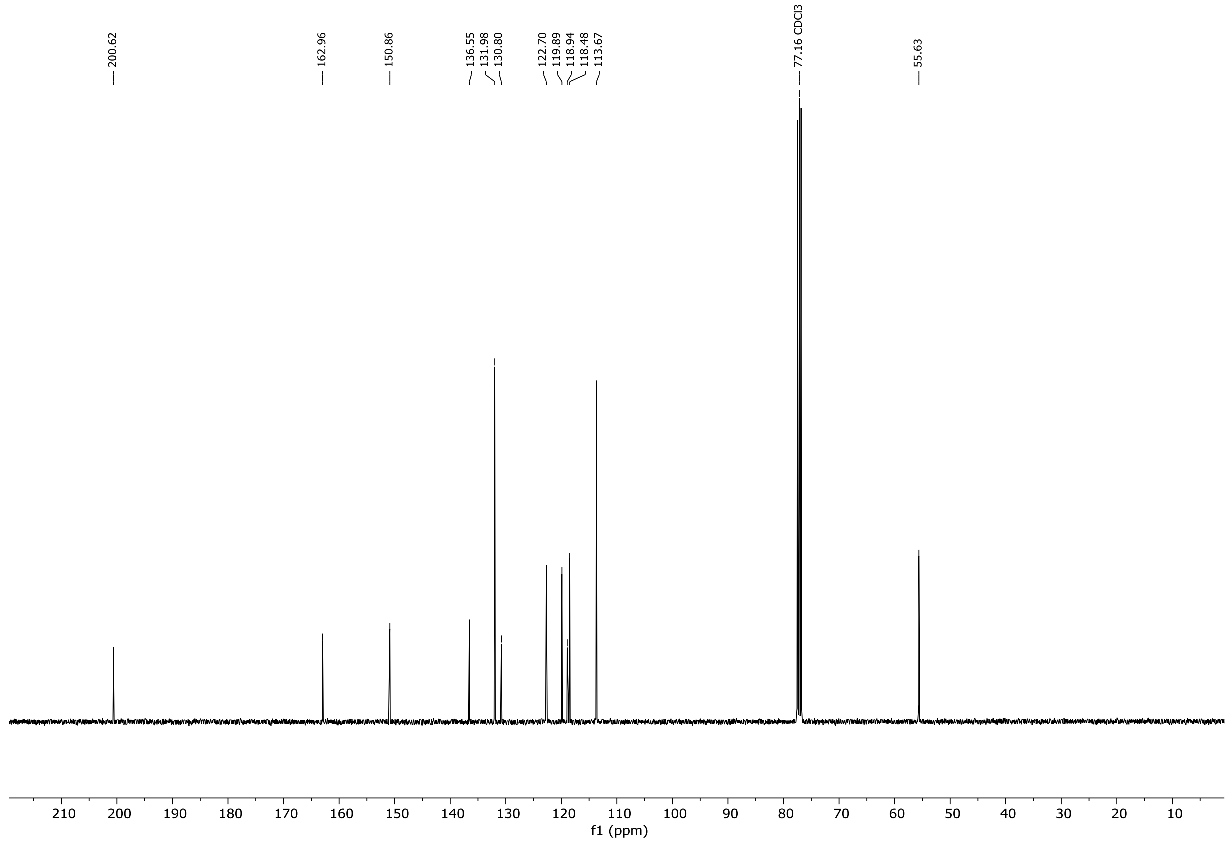

2-Amino-6-(4-methoxybenzoyl)phenyl (1*RS*,5*RS*,7*RS*)-1,5,7-trimethyl-2-oxo-3-azabicyclo[3.3.1]nonane-7-carboxylate (*rac*-SI-11)

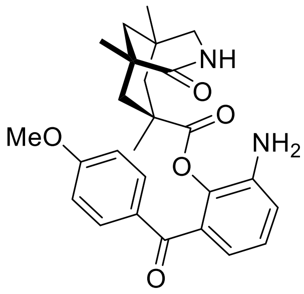

rac-**SI-11**

$C_{26}H_{30}N_2O_5$

M = 450.54 g mol^{-1}

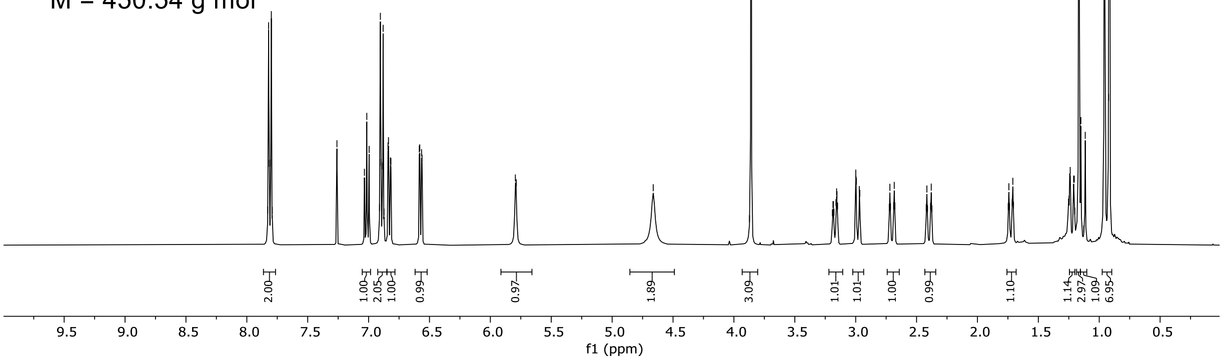

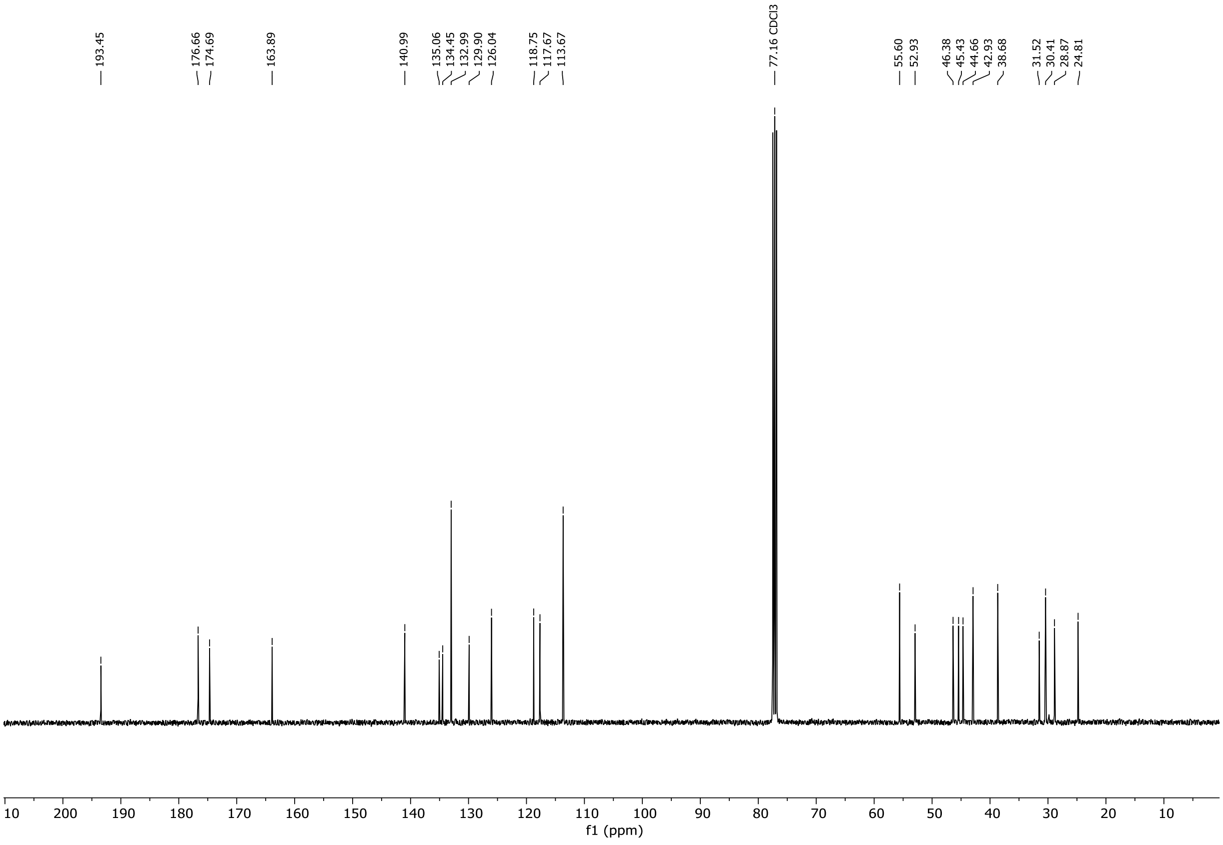

(1*SR*,5*SR*,7*RS*)-7-(7-(4-Methoxybenzoyl)benzo[*d*]oxazol-2-yl)-1,5,7-trimethyl-3-azabicyclo[3.3.1]nonan-2-one (*rac*-2b)

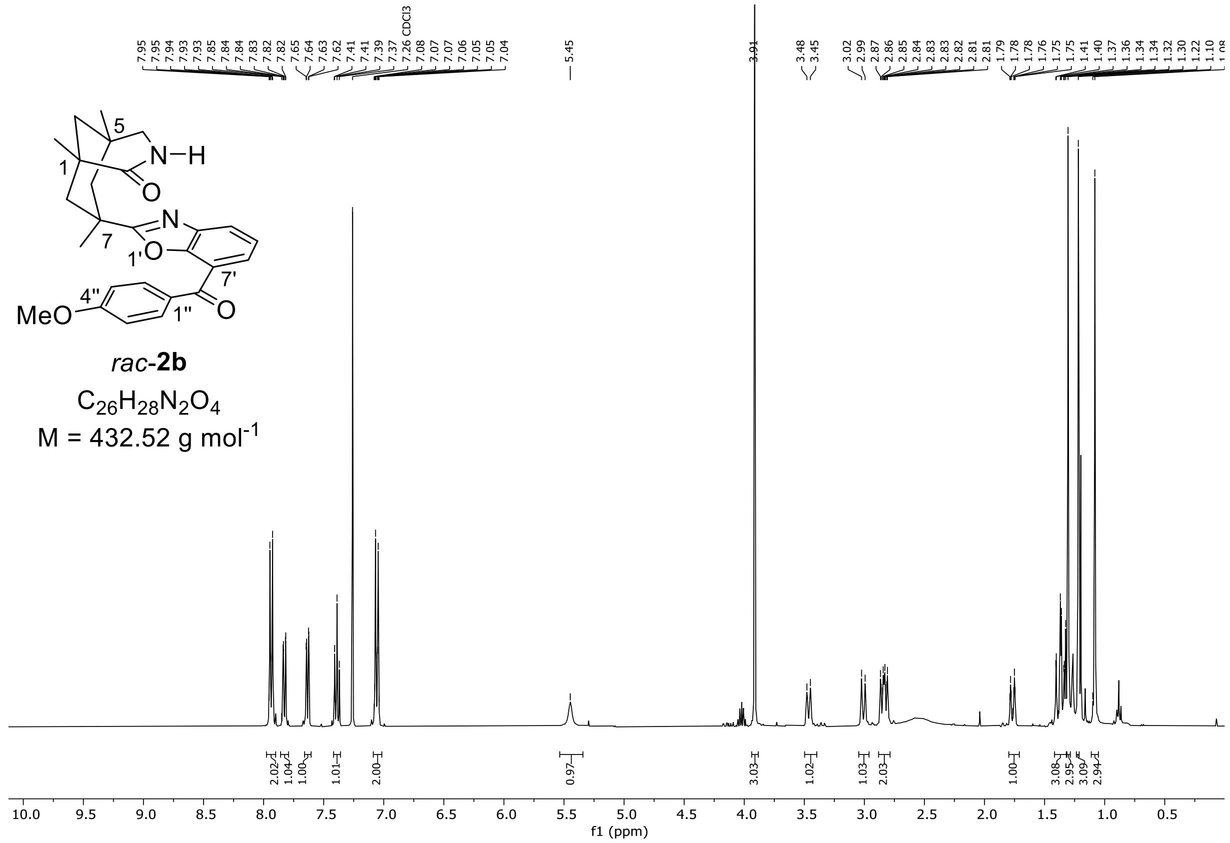

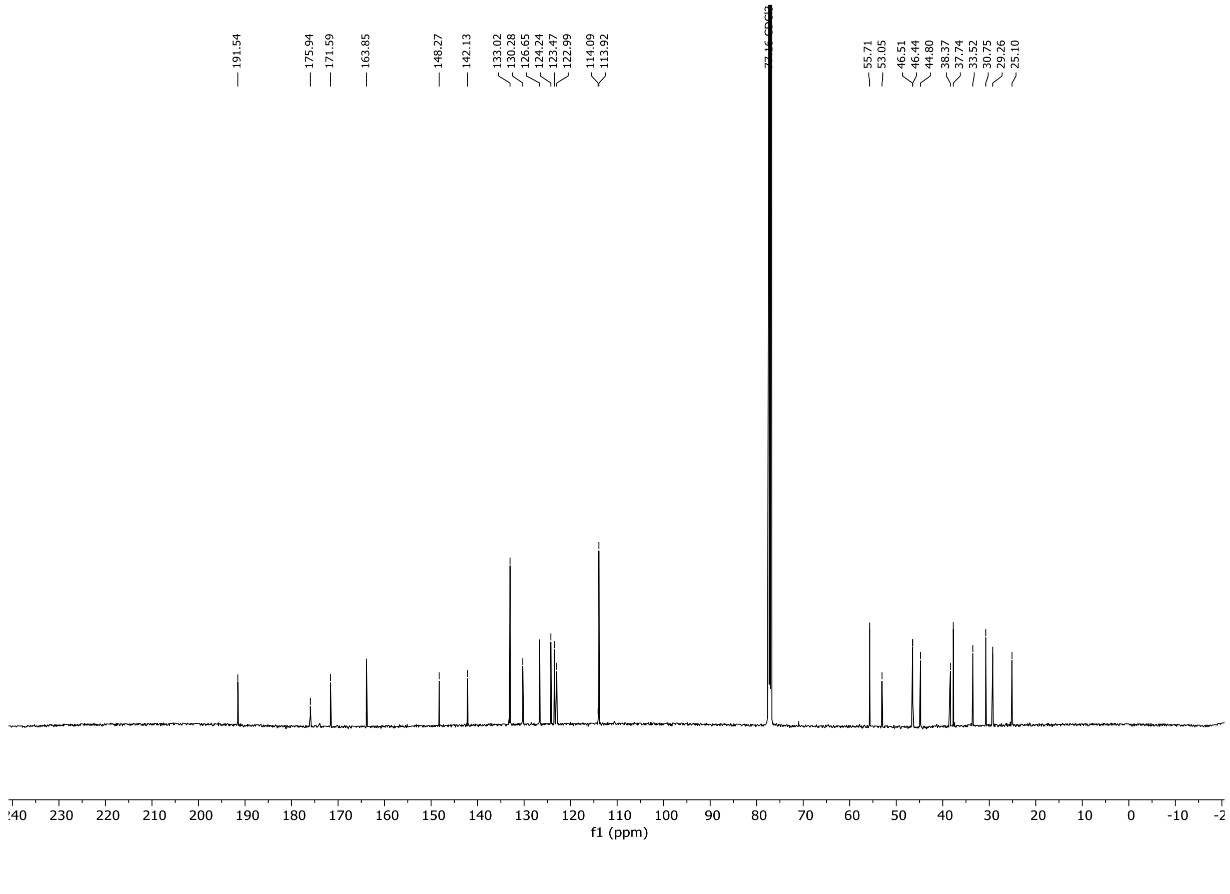

(2-Methoxy-3-nitrophenyl)(4-(trifluoromethyl)phenyl)methanol (SI-12)

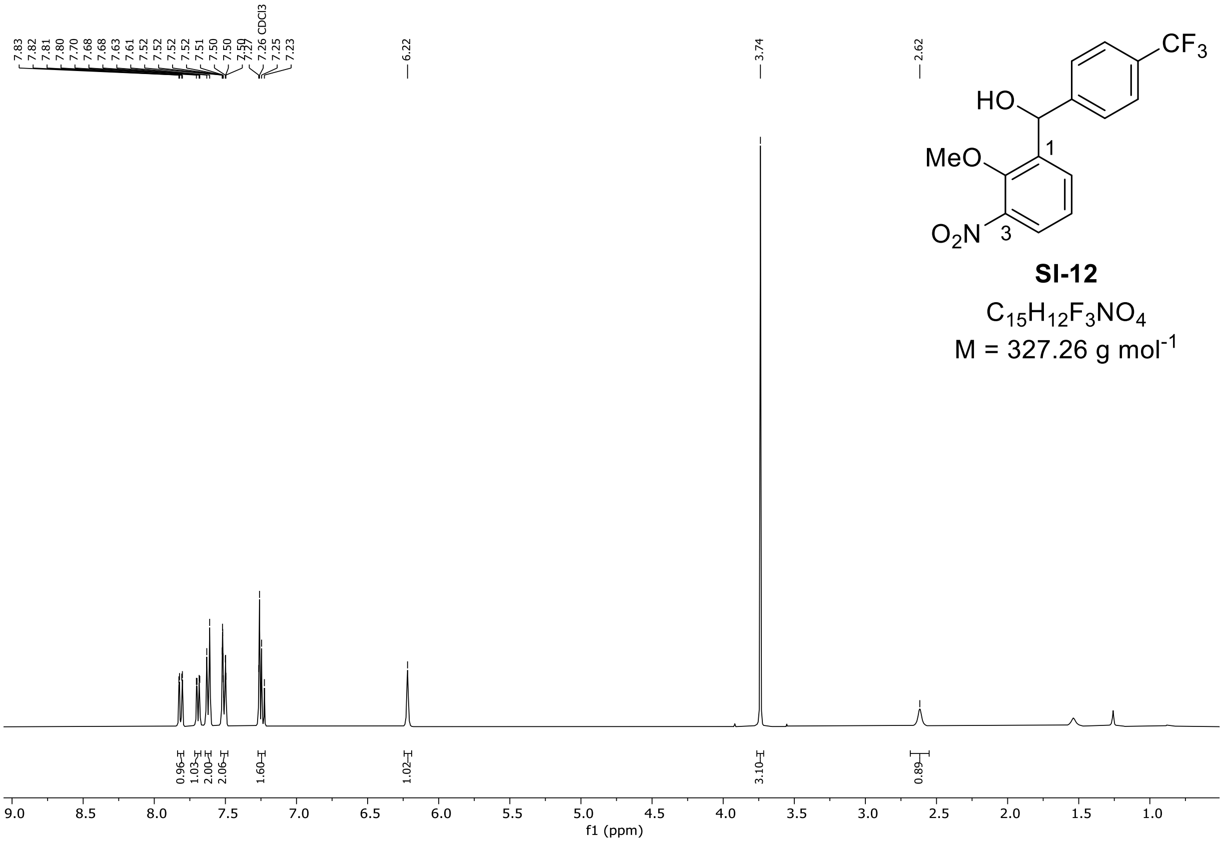

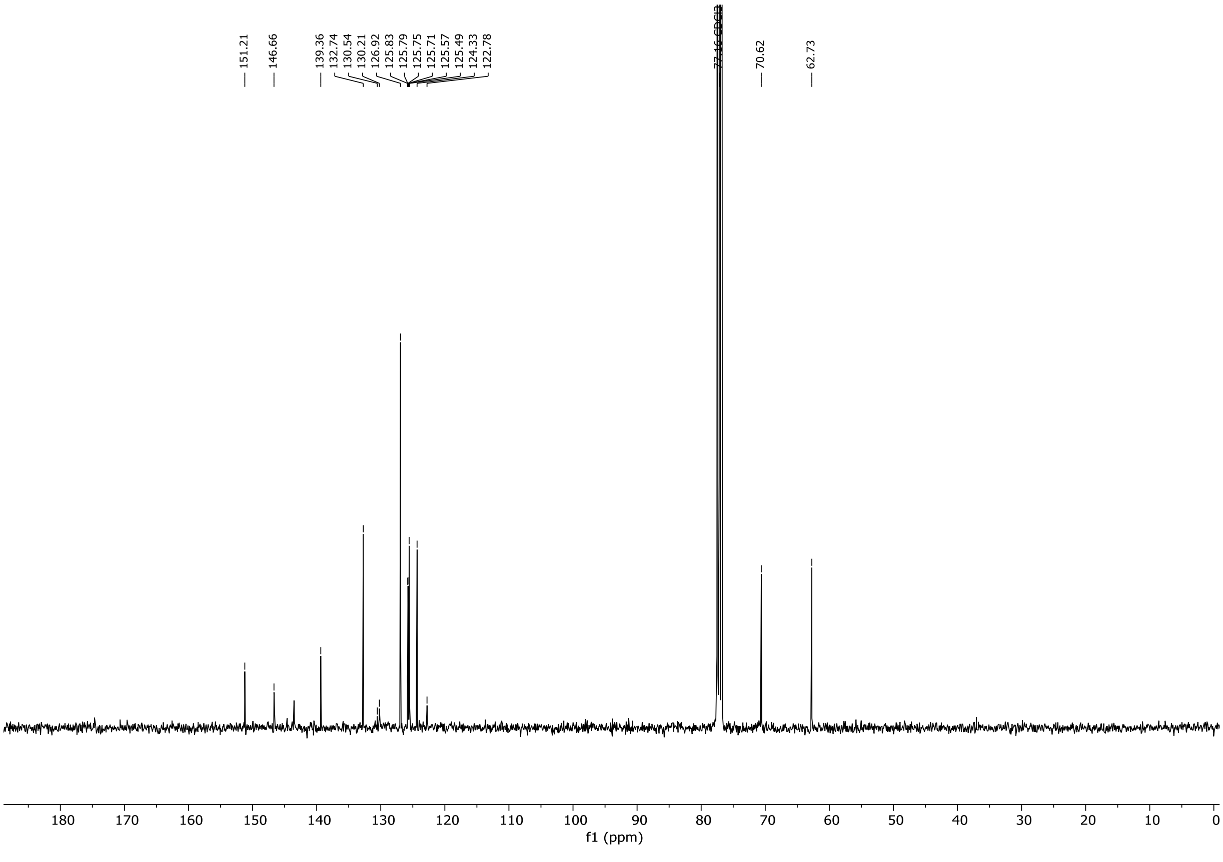

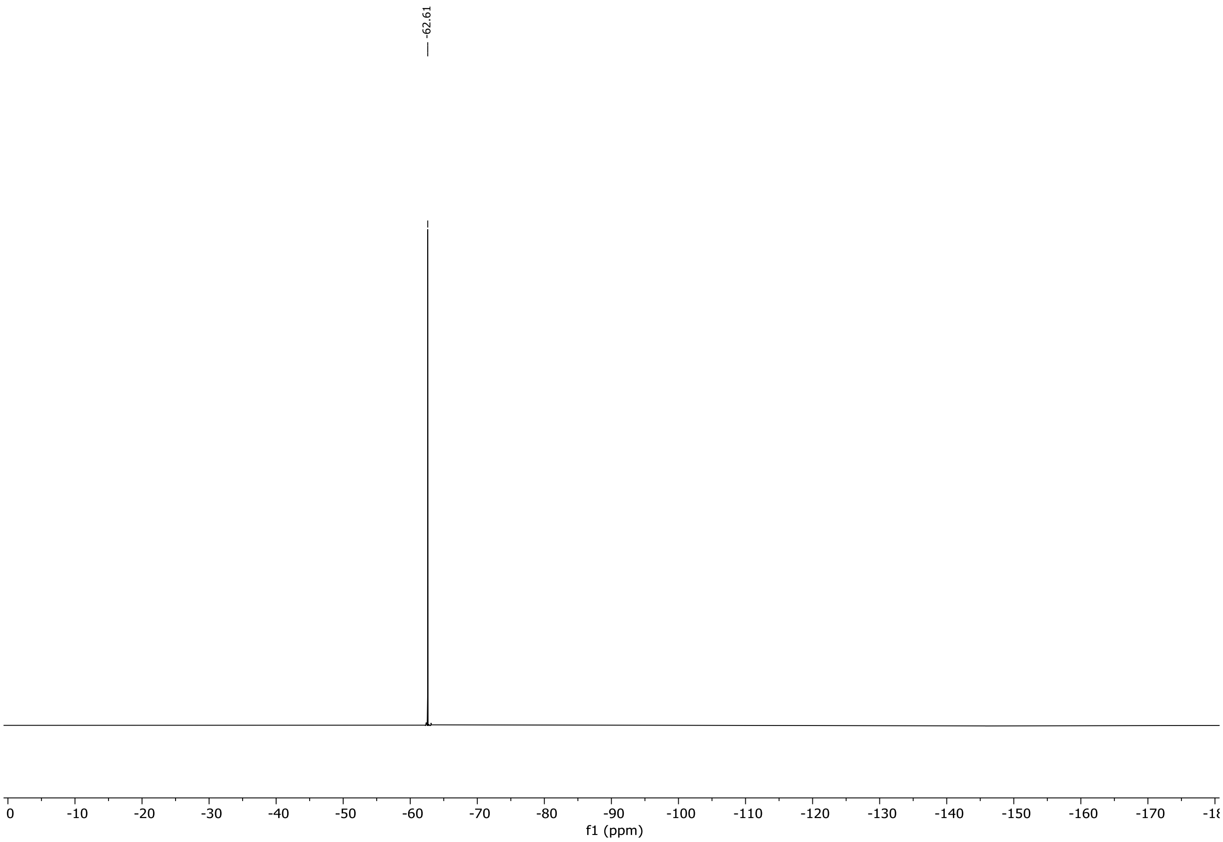
-62.61
0
-10
-20
-30
-40
-50
-60
-70
-80
-90
-100
-110
-120
-130
-140
-150
-160
-170
f1 (ppm)

(2-Methoxy-3-nitrophenyl)(4-(trifluoromethyl)phenyl)methanone (SI-13)

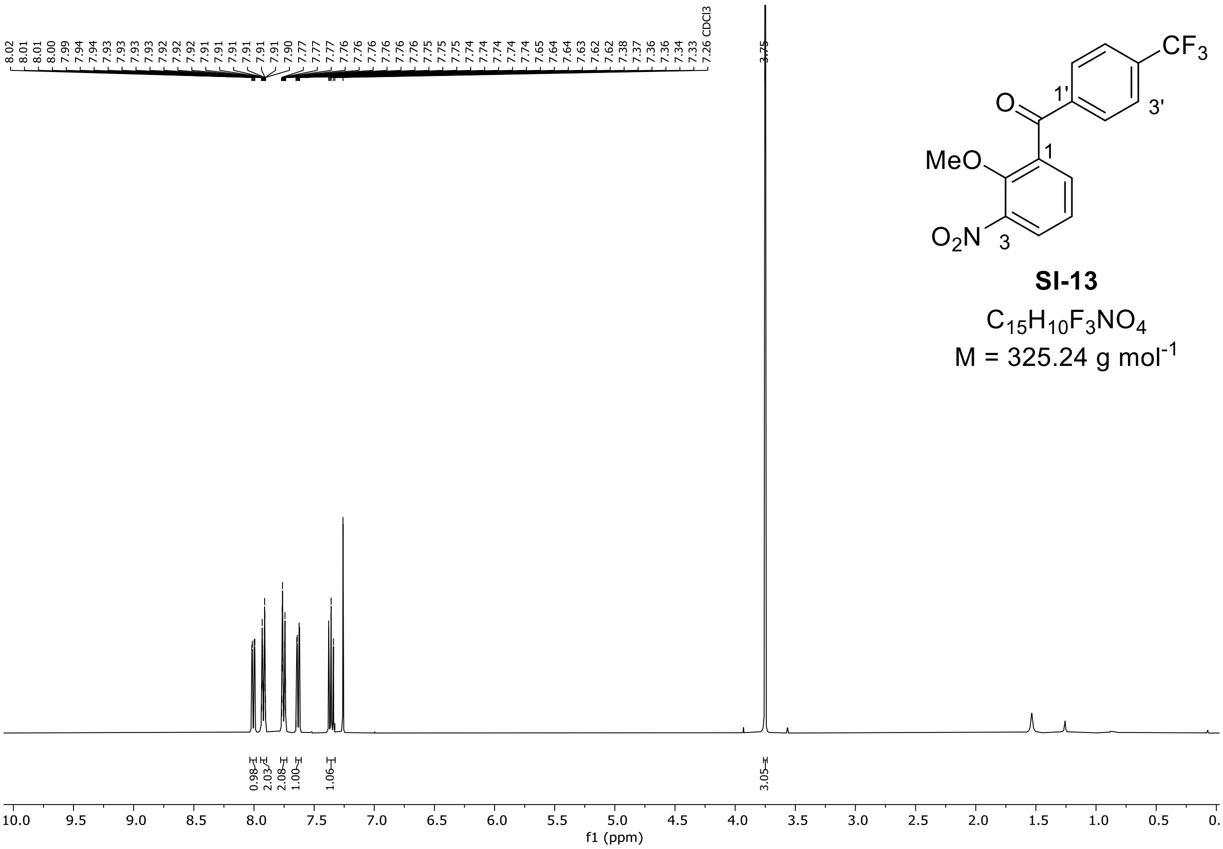

SI-13

$C_{15}H_{10}F_3NO_4$

M = 325.24 g mol^{-1}

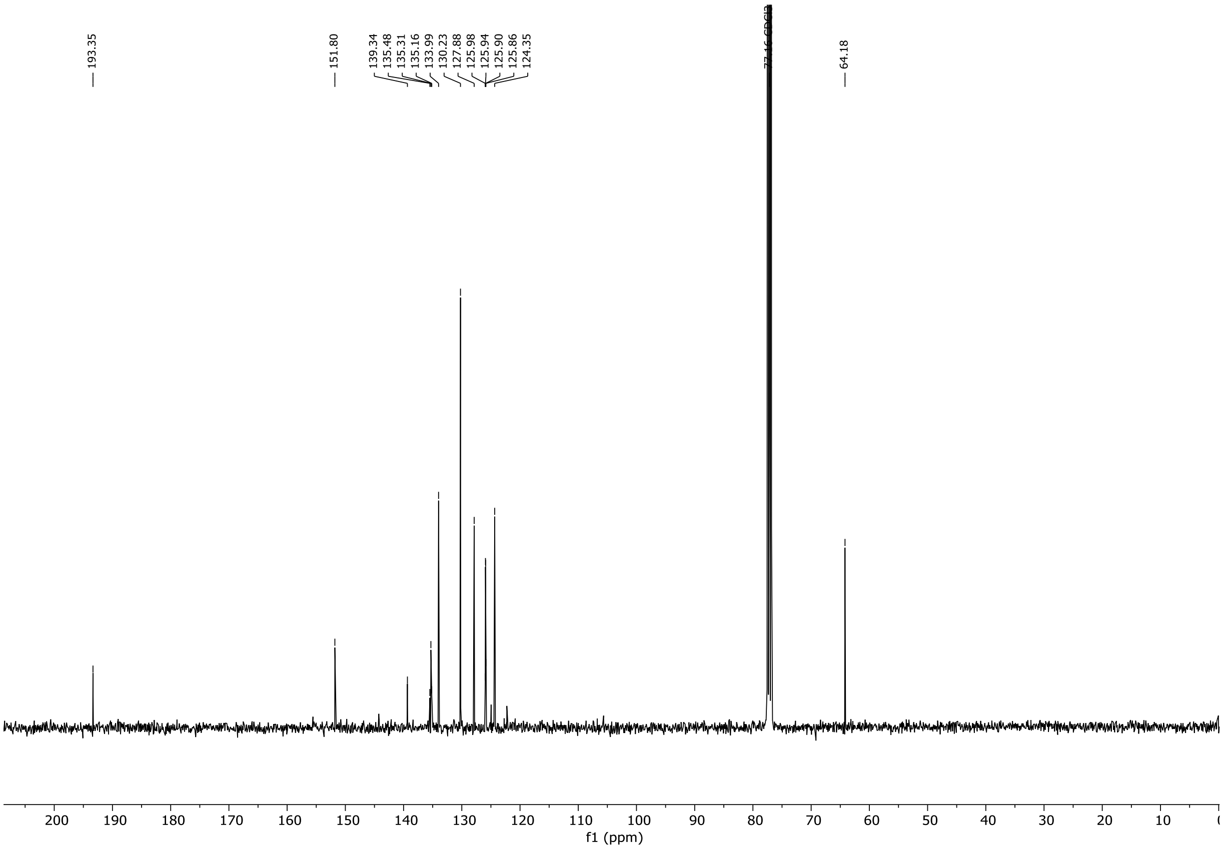

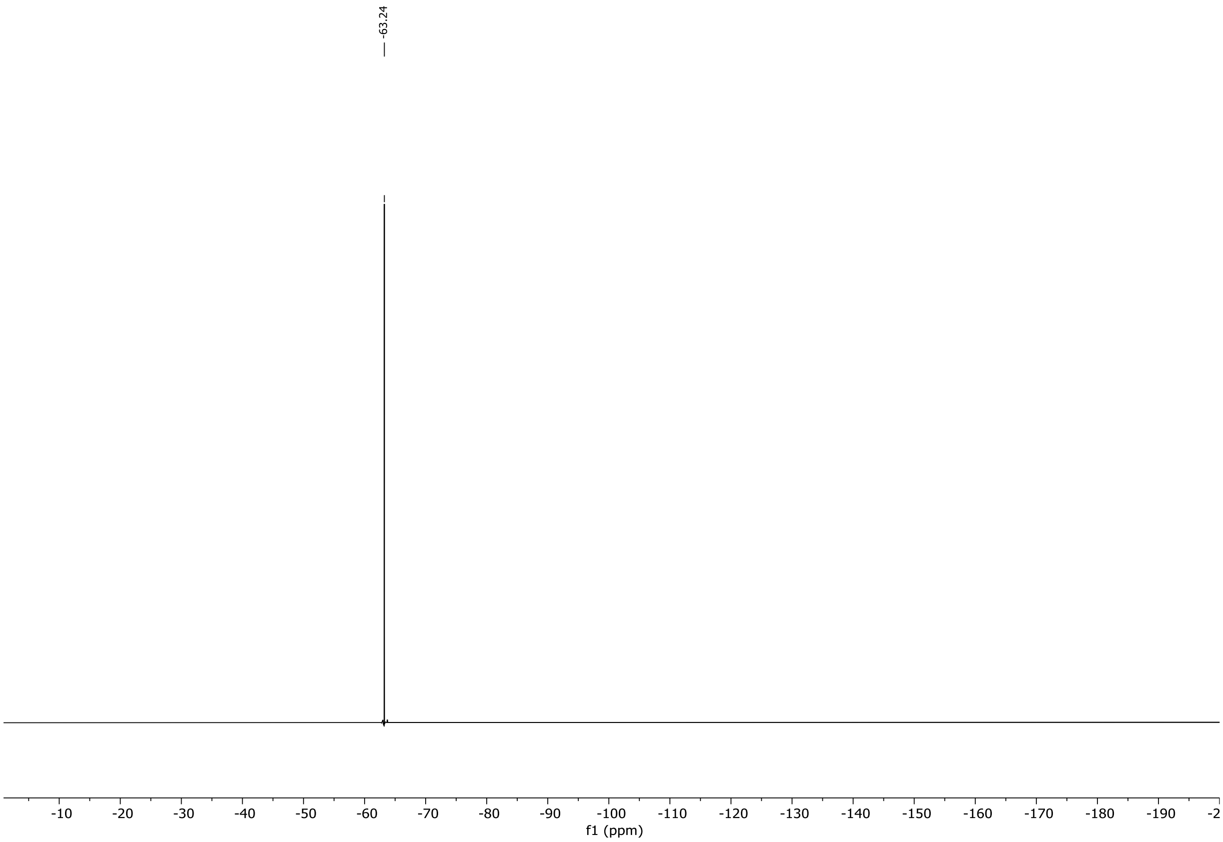
-63.24
-10
-20
-30
-40
-50
-60
-70
-80
-90
-100
-110
-120
-130
-140
-150
-160
-170
-180
-190
-2
f1 (ppm)

(2-Hydroxy-3-nitrophenyl)(4-(trifluoromethyl)phenyl)methanone (SI-14)

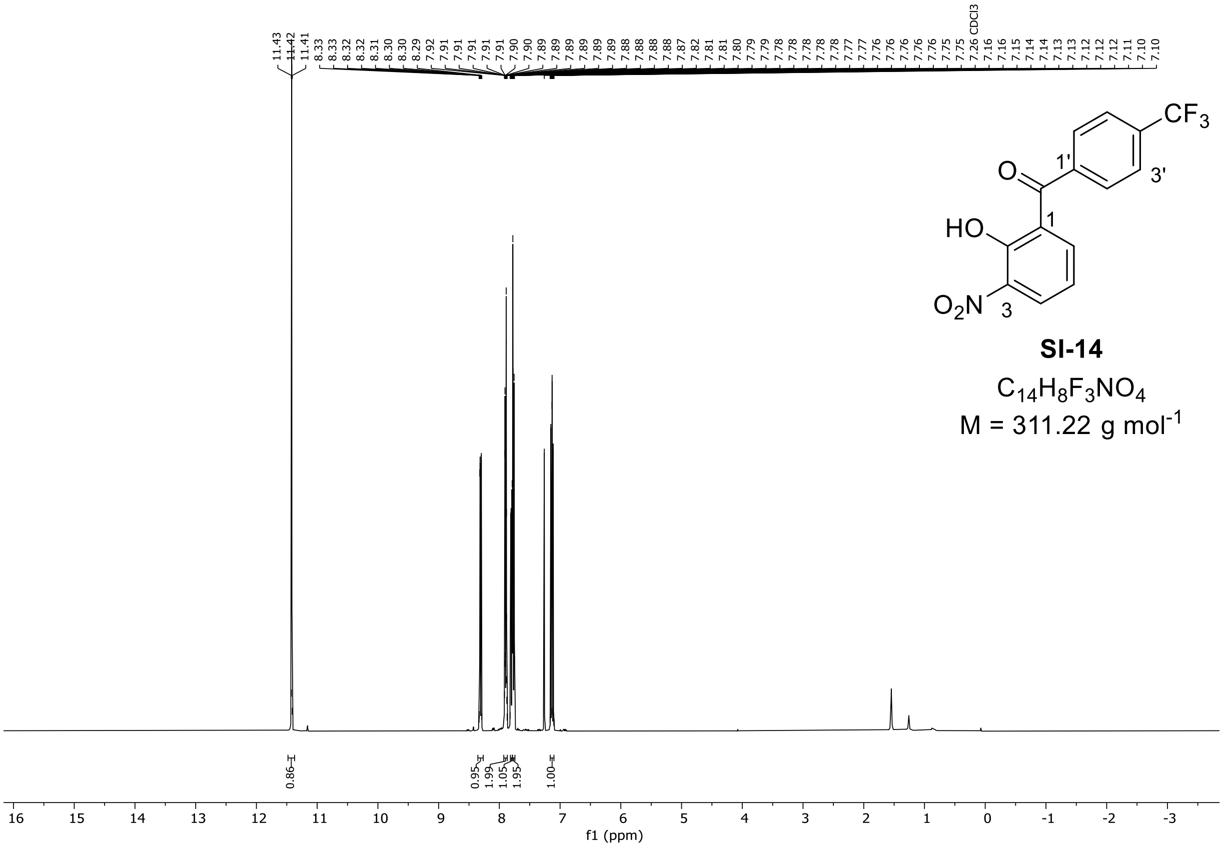

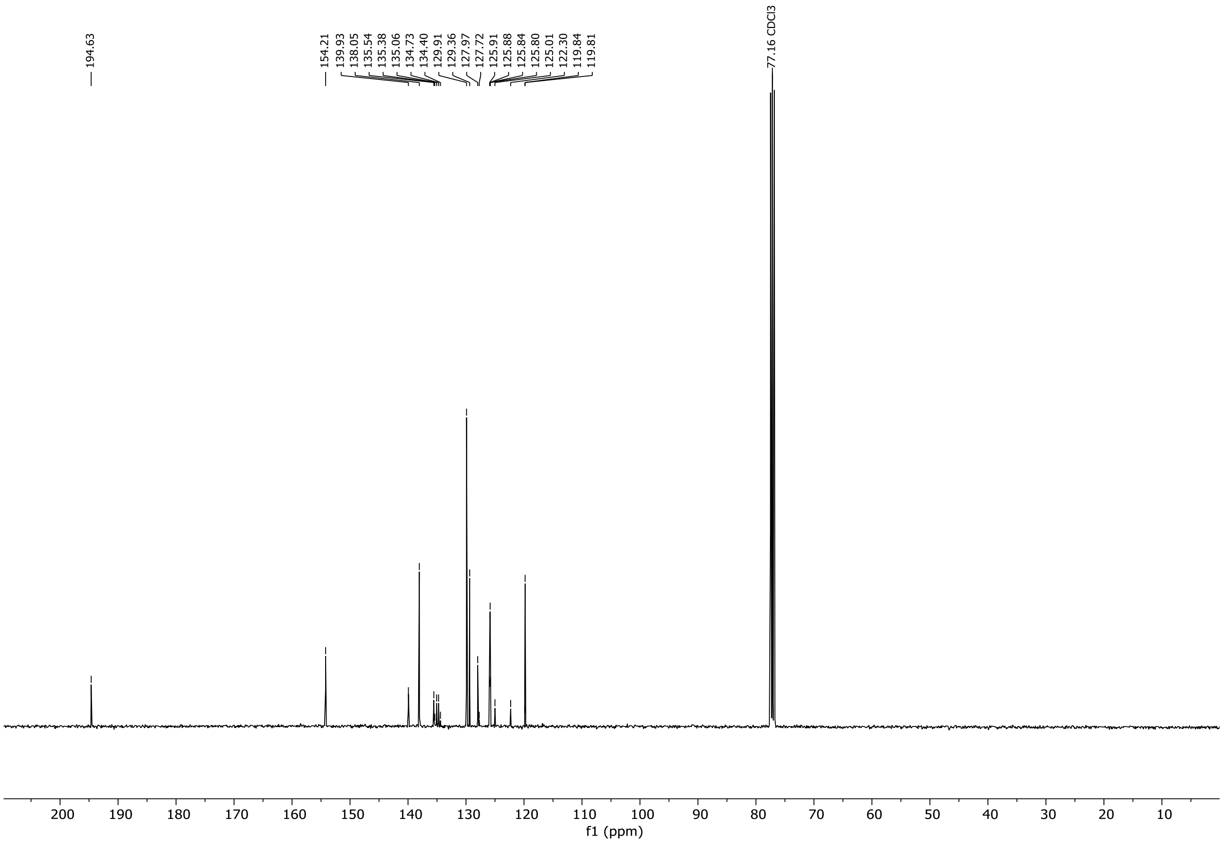

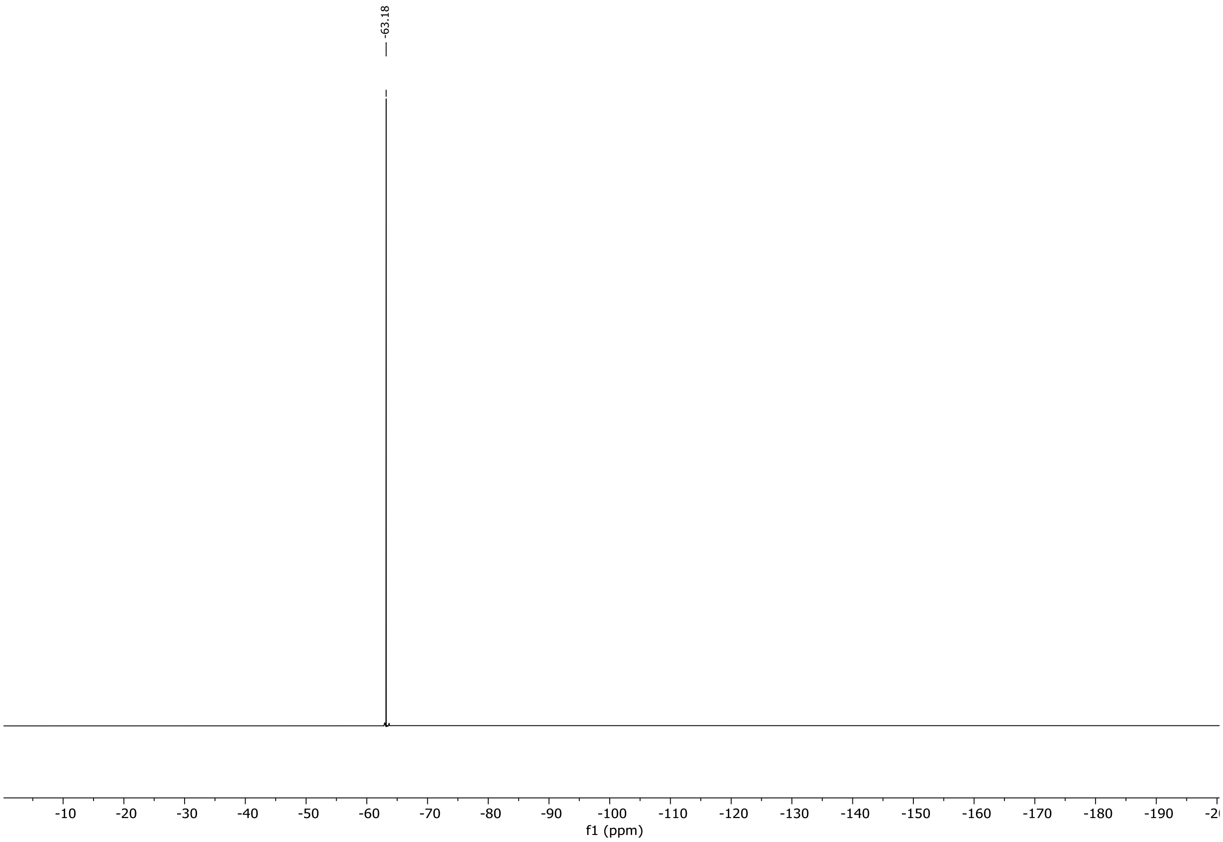
-63.18
-10 -20 -30 -40 -50 -60 -70 -80 -90 -100 -110 -120 -130 -140 -150 -160 -170 -180 -190
f1 (ppm)

(3-Amino-2-hydroxyphenyl)(4-(trifluoromethyl)phenyl)methanone (SI-15)

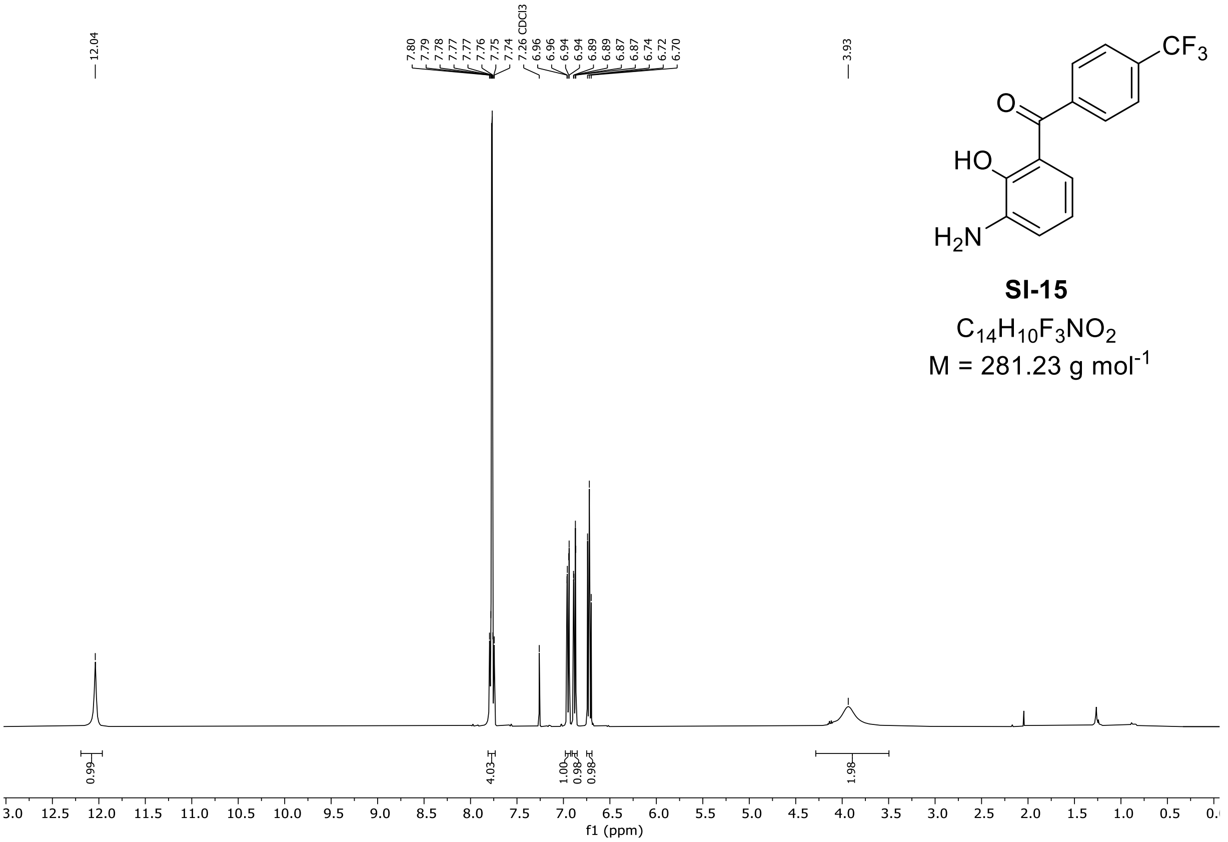

SI-15

$C_{14}H_{10}F_3NO_2$

M = 281.23 g mol^{-1}

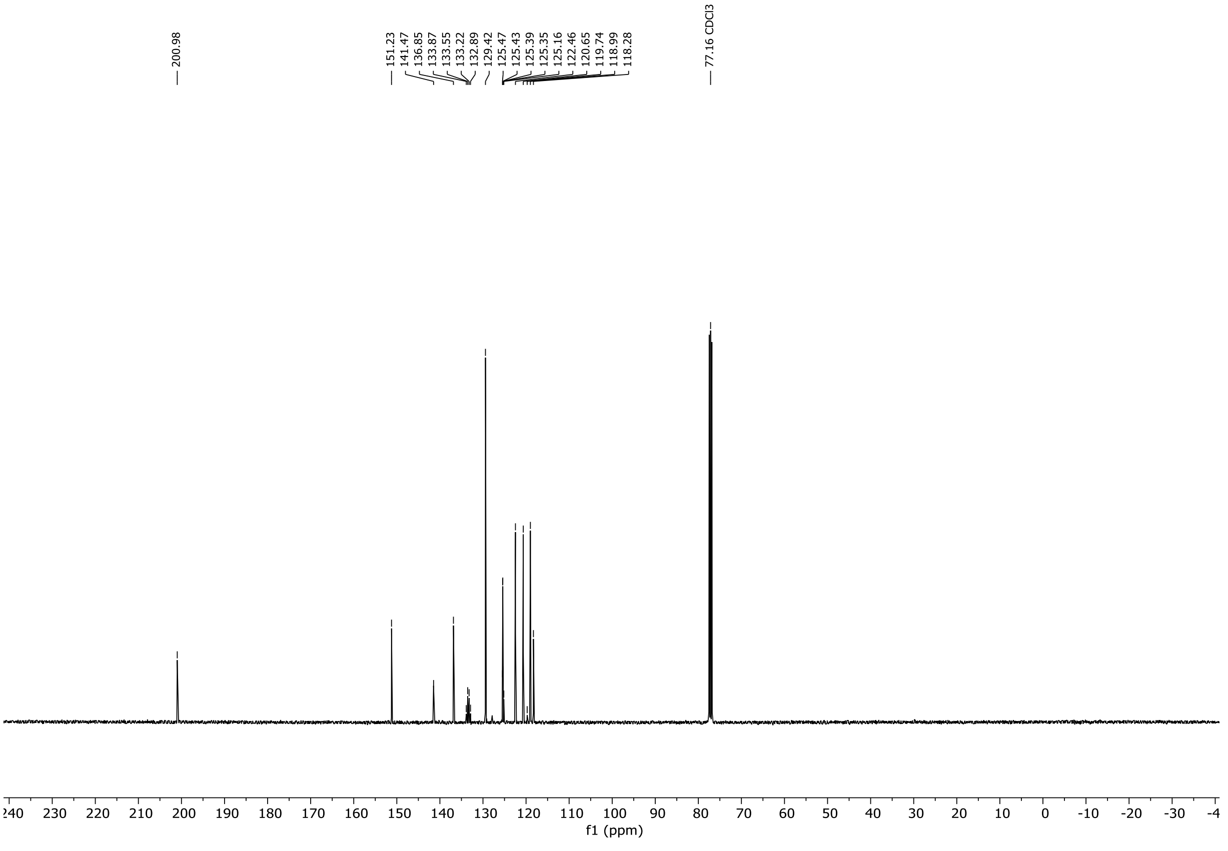

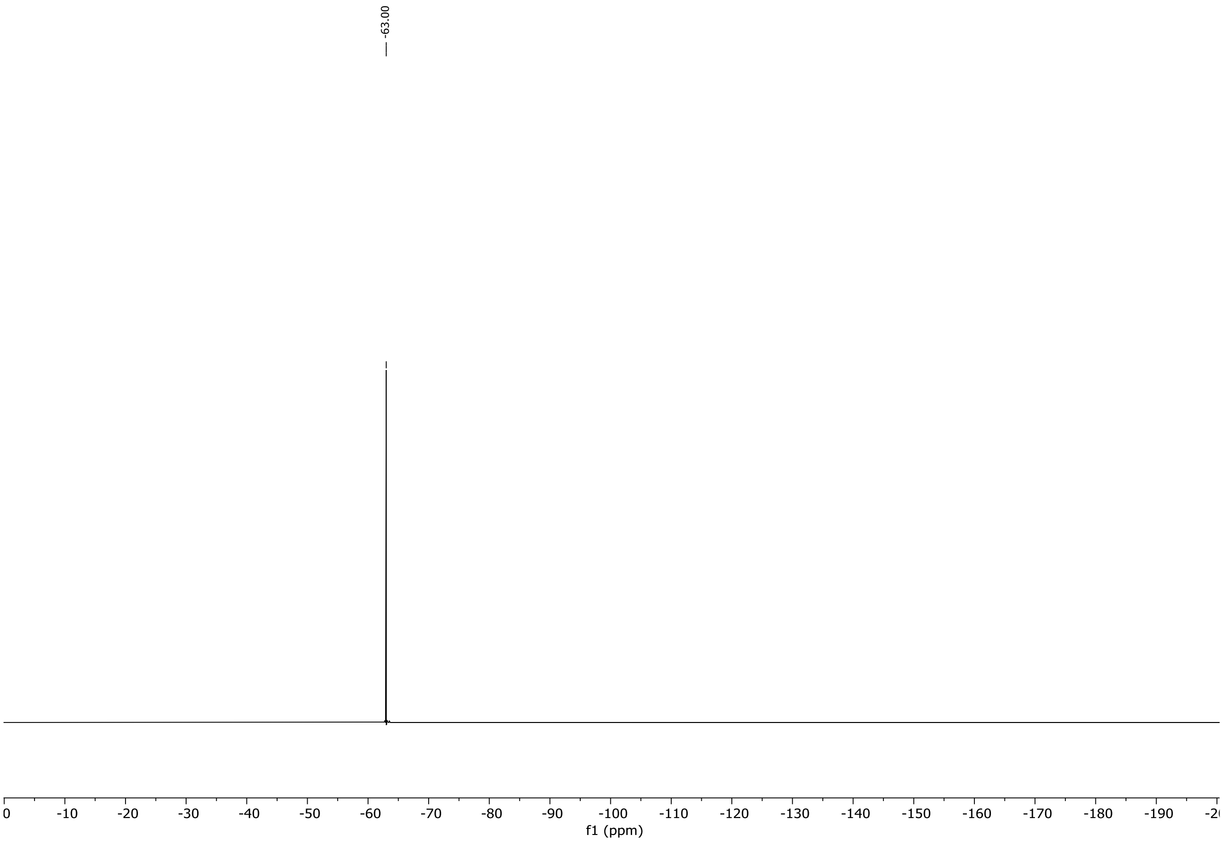
-63.00
0
-10
-20
-30
-40
-50
-60
-70
-80
-90
-100
-110
-120
-130
-140
-150
-160
-170
-180
-190
f1 (ppm)

2-Amino-6-(4-(trifluoromethyl)benzoyl)phenyl (1*SR*,5*RS*,7*RS*)-1,5,7-trimethyl-2-oxo-3-azabicyclo[3.3.1]nonane-7-carboxylate (*rac*-SI-16)

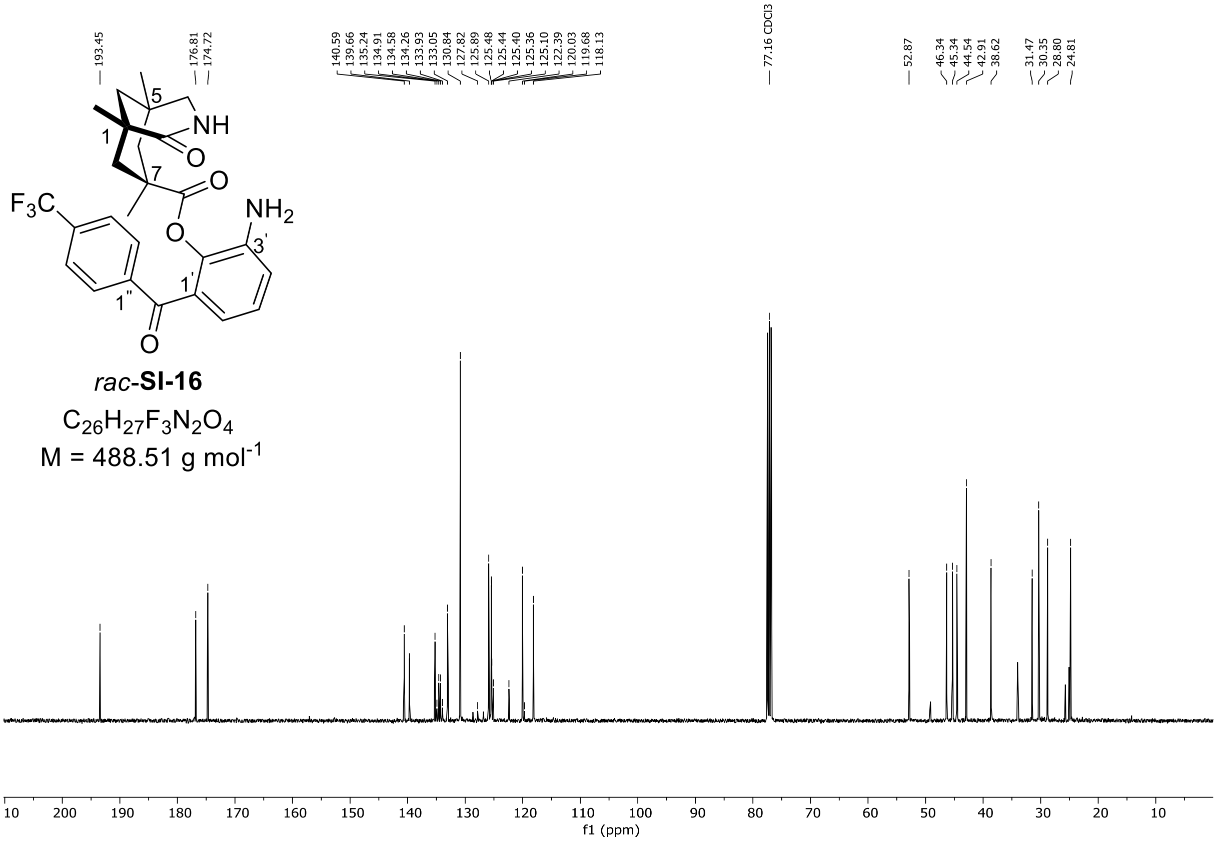

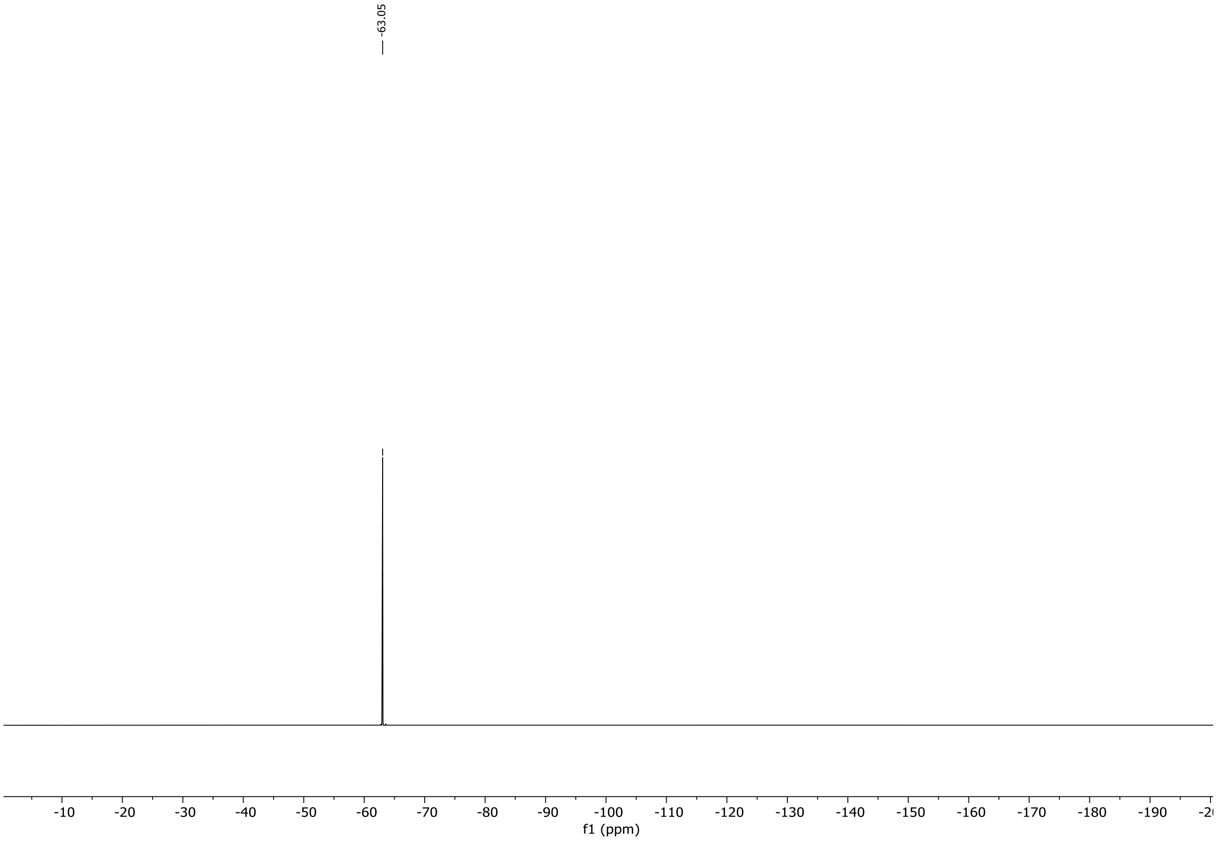

(1*SR*,5*SR*,7*RS*)-1,5,7-Trimethyl-7-(7-(4-(trifluoromethyl)benzoyl)benzo[*d*]oxazol-2-yl)-3-azabicyclo[3.3.1]nonan-2-one (*rac*-2c)

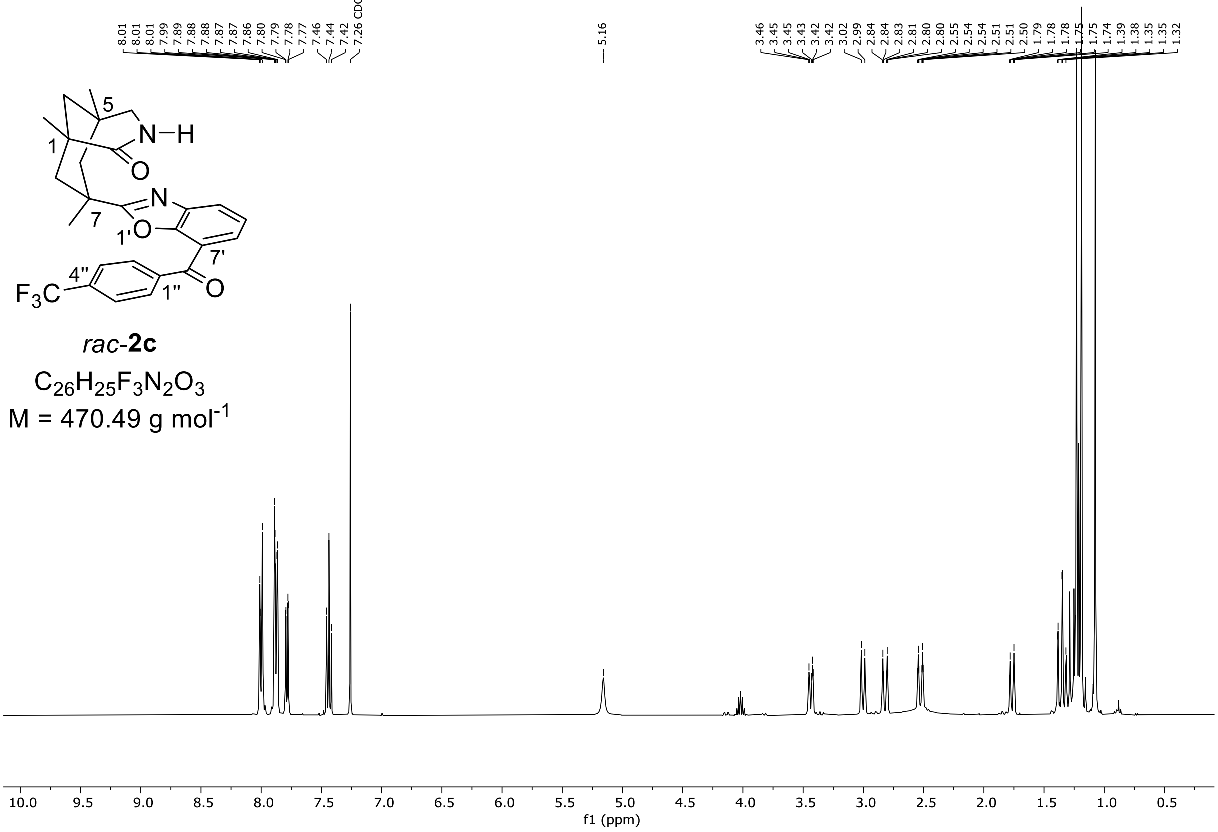

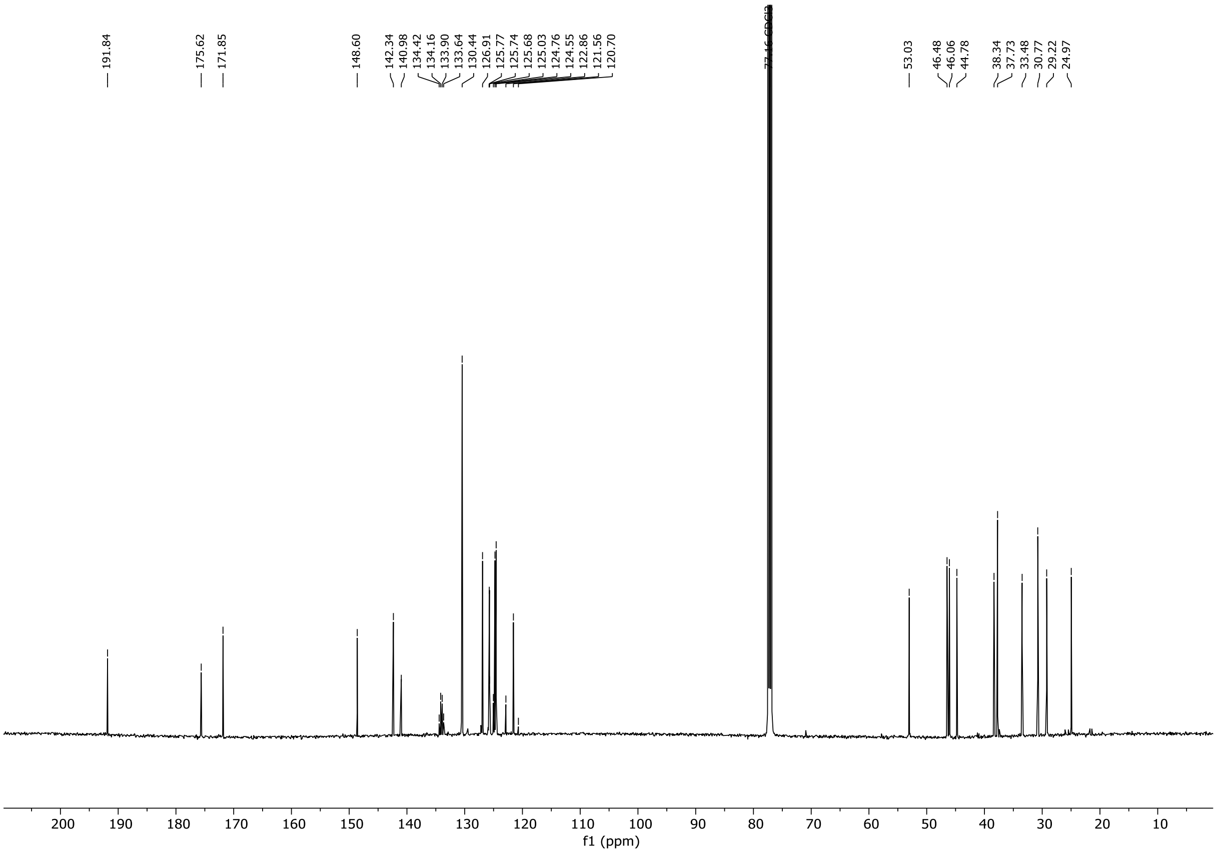

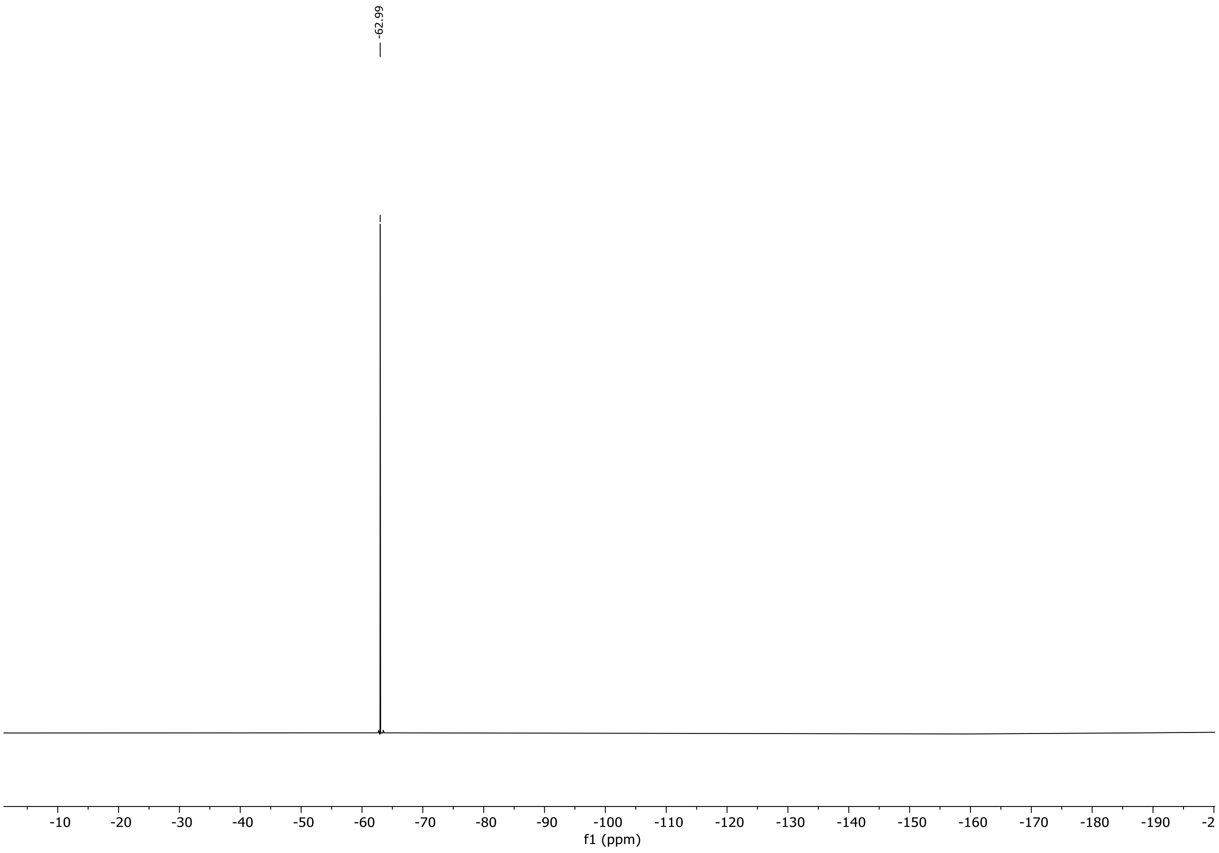
-62.99
-10 -20 -30 -40 -50 -60 -70 -80 -90 -100 -110 -120 -130 -140 -150 -160 -170 -180 -190 -2
f1 (ppm)

Methyl 2-(chloromethyl)nicotinate (SI-2)

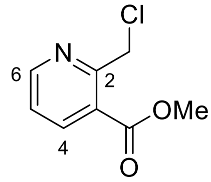

SI-2

$C_8H_8ClNO_2$

M = 185.61 g mol^{-1}

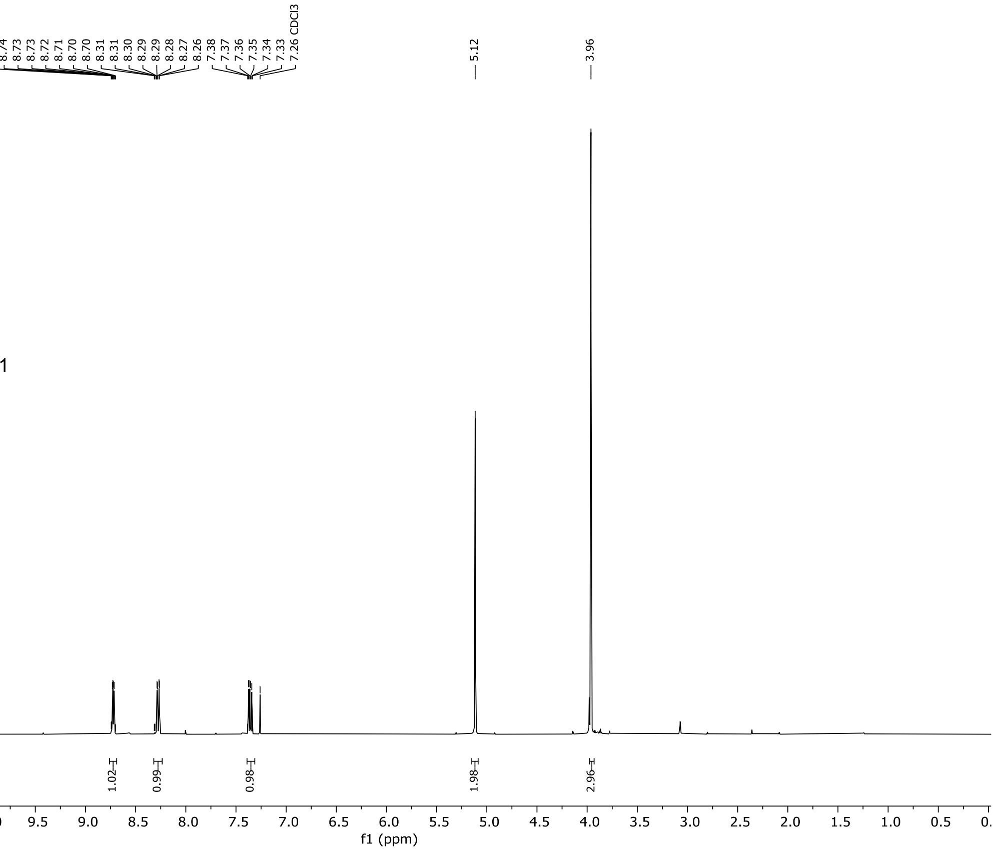

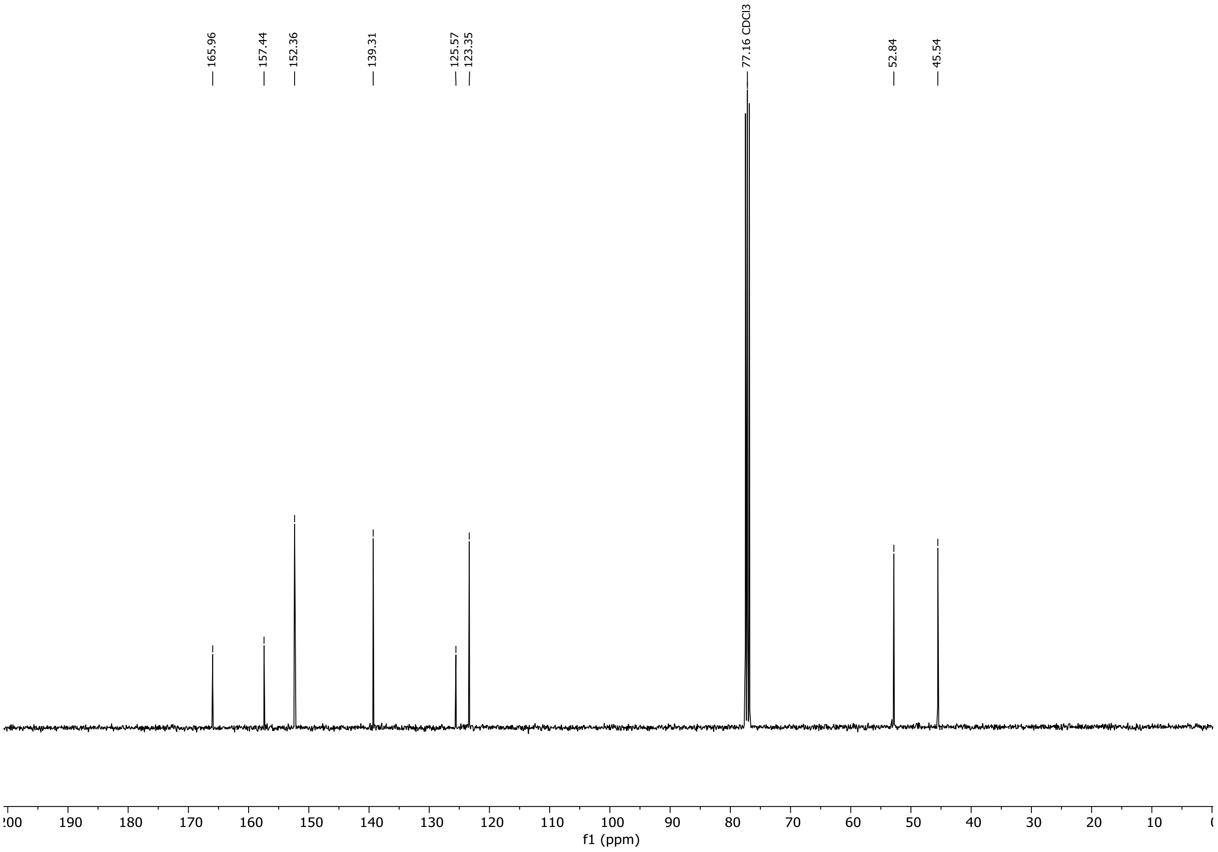

Methyl 6-bromo-2-(bromomethyl)nicotinate (SI-1a)

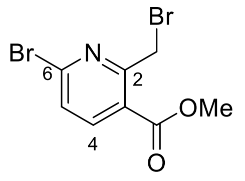

SI-1a

$C_8H_7Br_2NO_2$

M = 308.96 g mol^{-1}

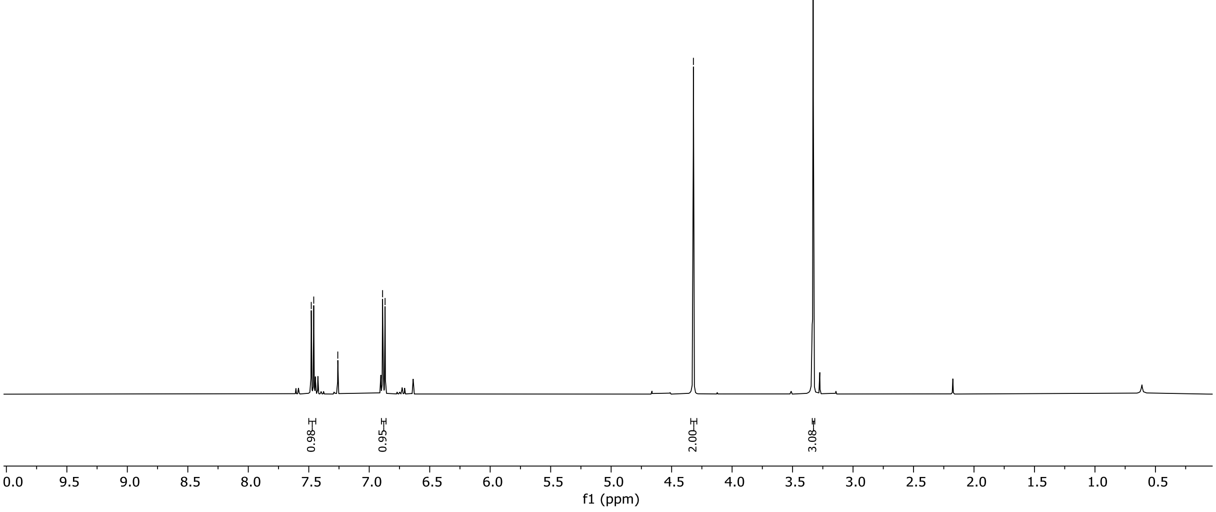

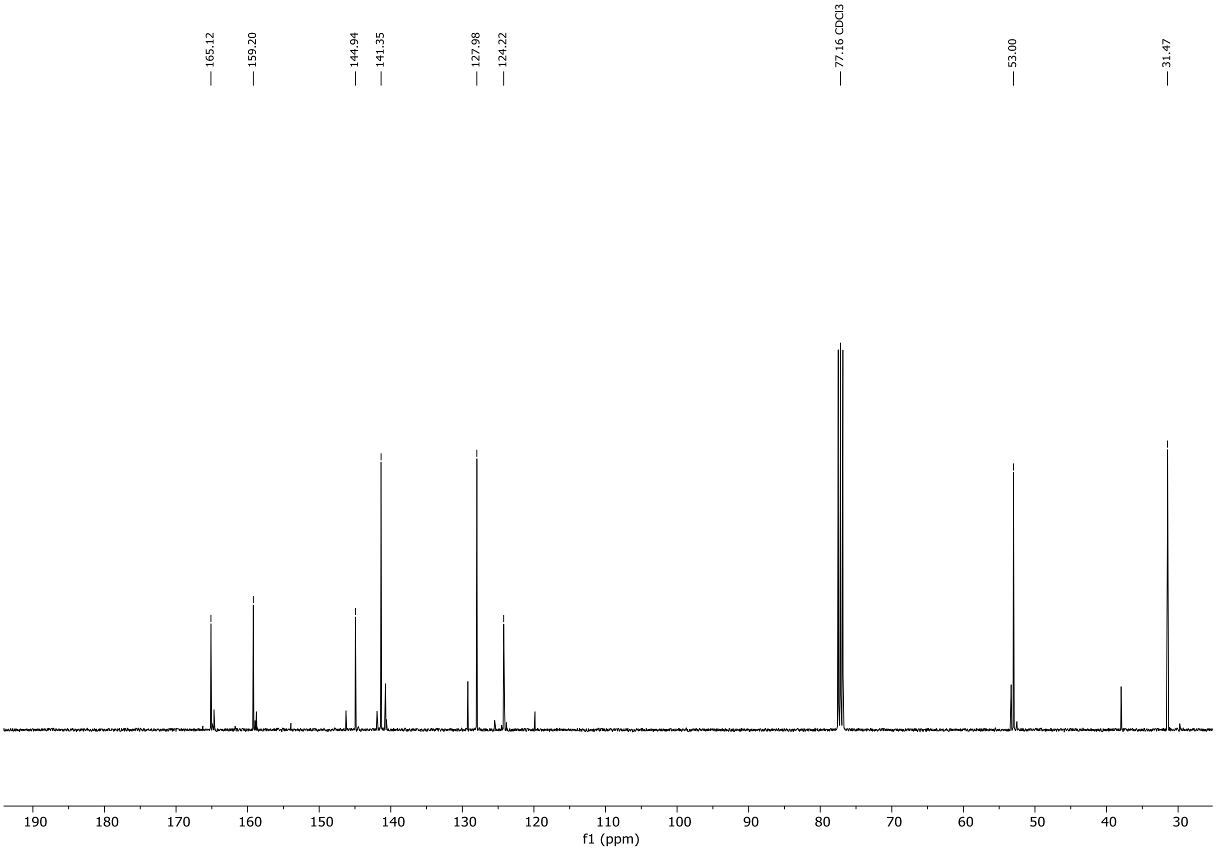

Methyl 2-(bromomethyl)-6-(trifluoromethyl)nicotinate (SI-1b)

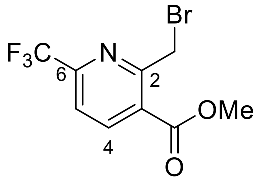

SI-1b

$C_9H_7BrF_3NO_2$

M = 298.06 g mol^{-1}

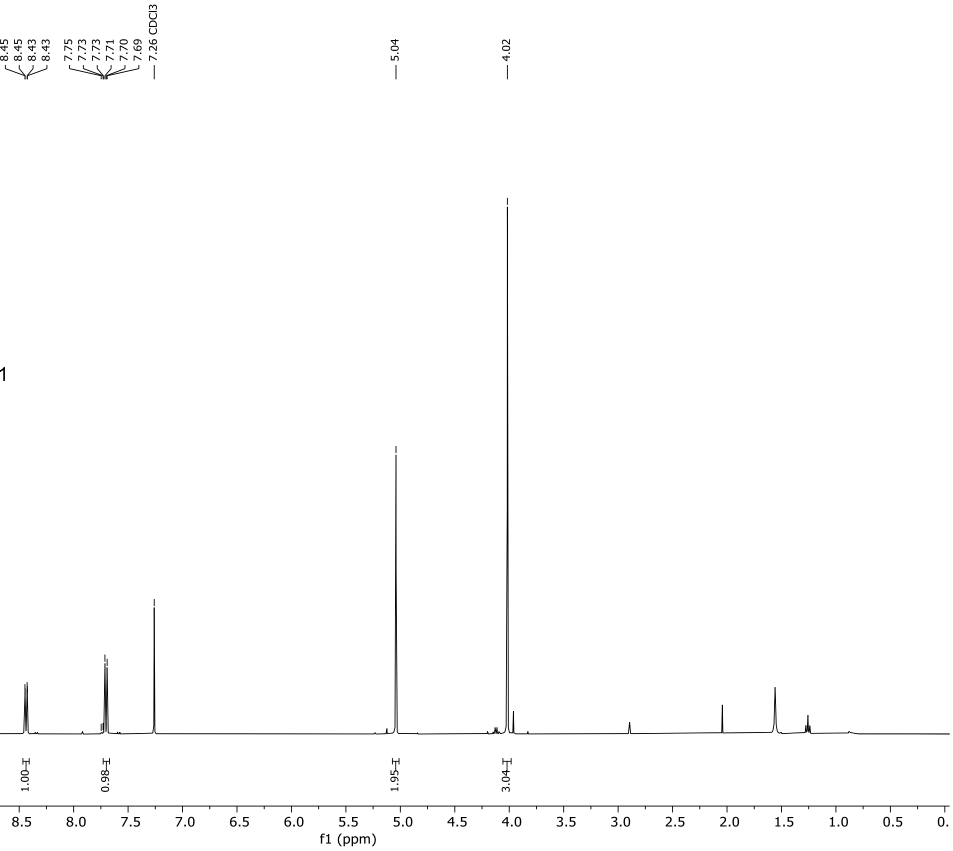

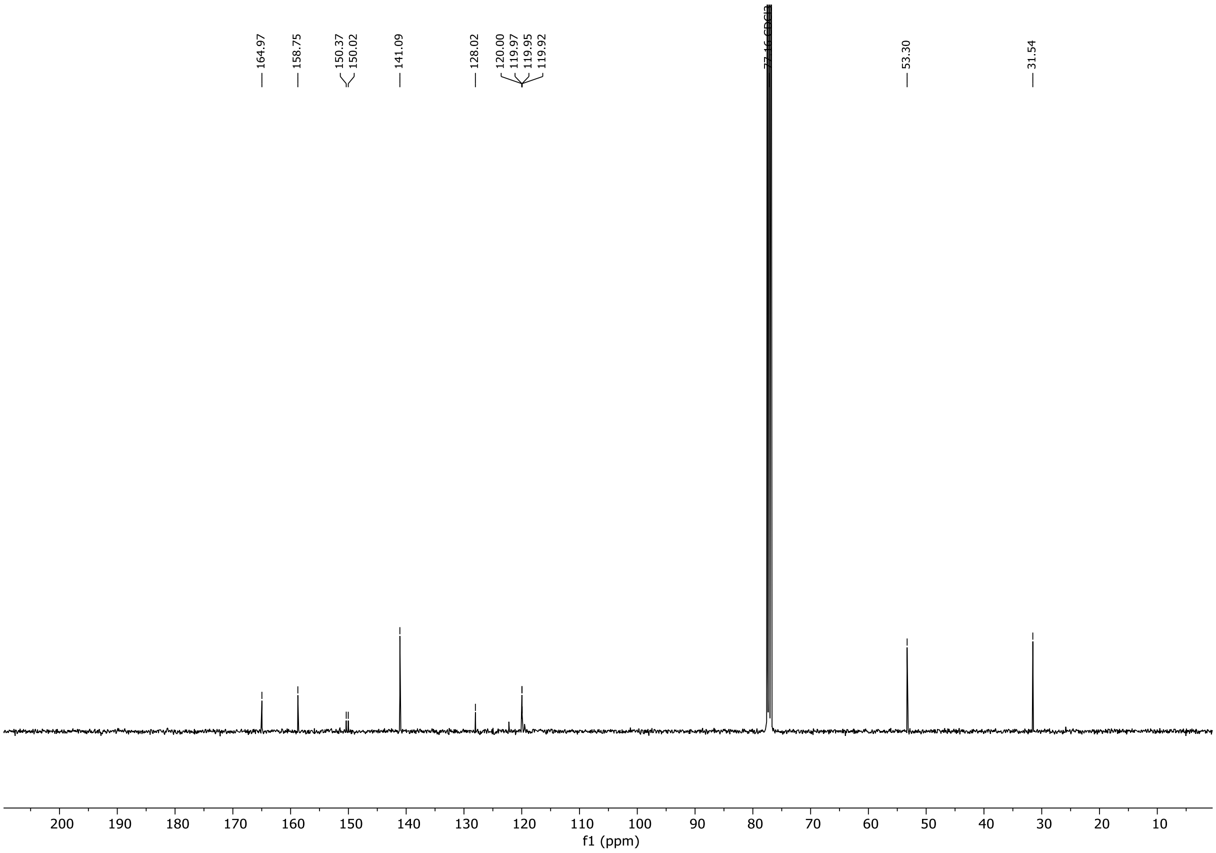

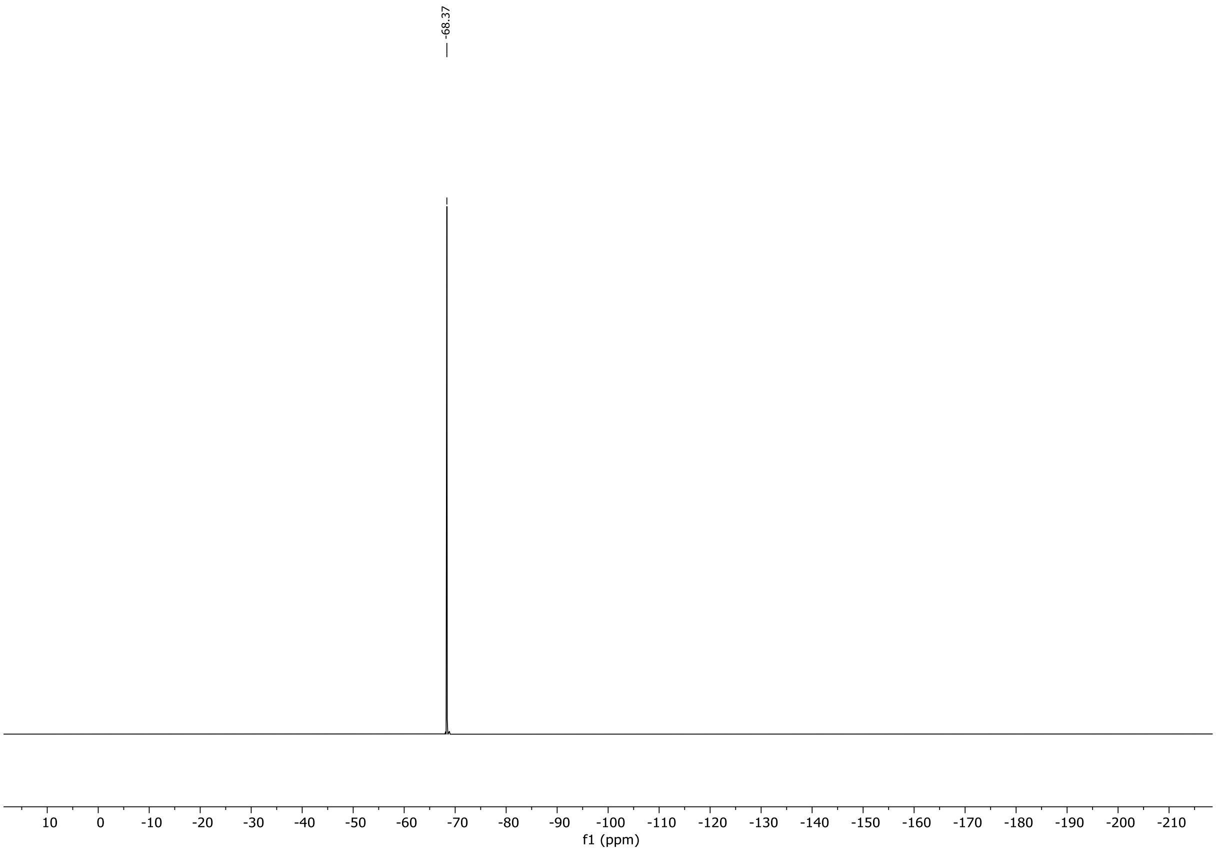
-68.37
10 0 -10 -20 -30 -40 -50 -60 -70 -80 -90 -100 -110 -120 -130 -140 -150 -160 -170 -180 -190 -200 -210
f1 (ppm)

6-(4-Methoxybenzyl)-6,7-dihydro-5*H*-pyrrolo[3,4-*b*]pyridin-5-one (SI-3a)

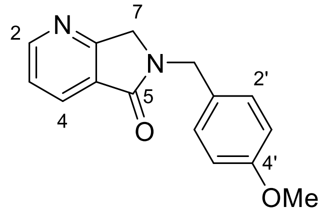

SI-3a

$C_{15}H_{14}N_2O_2$

M = 254.29 g mol^{-1}

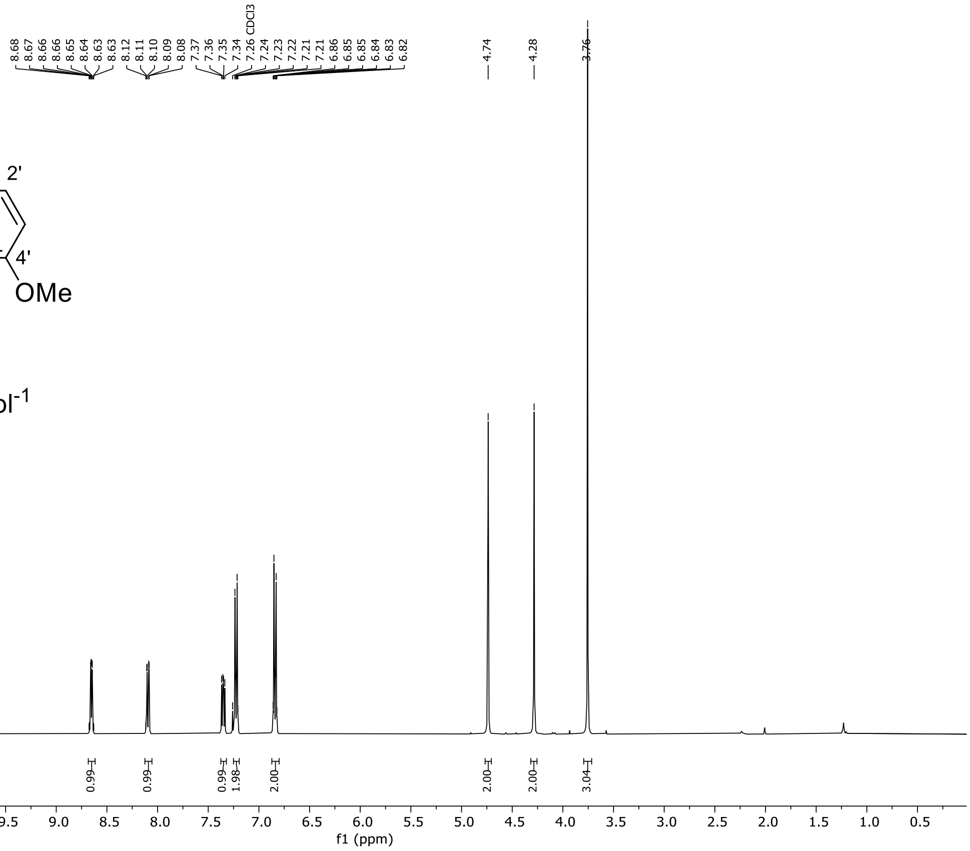

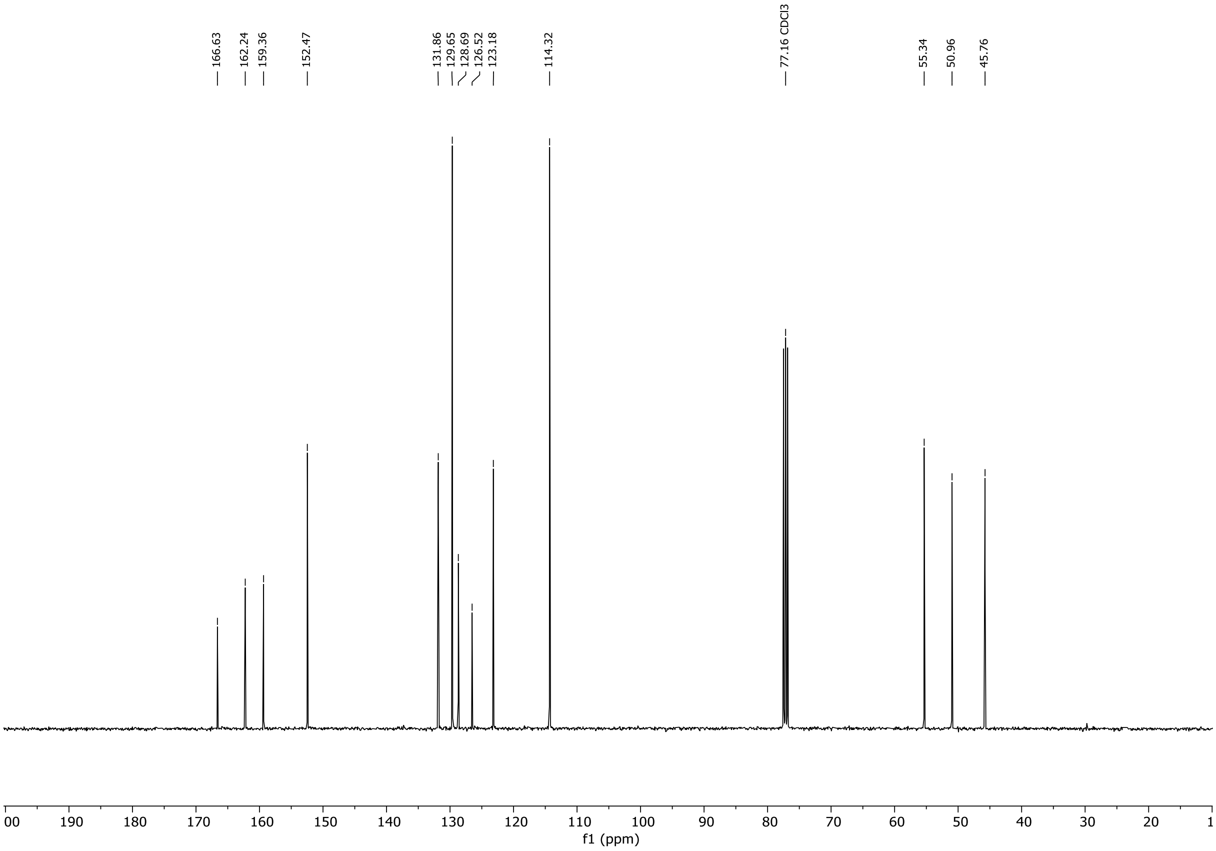

2-Bromo-6-(4-methoxybenzyl)-6,7-dihydro-5*H*-pyrrolo[3,4-*b*]pyridin-5-one (SI-3b)

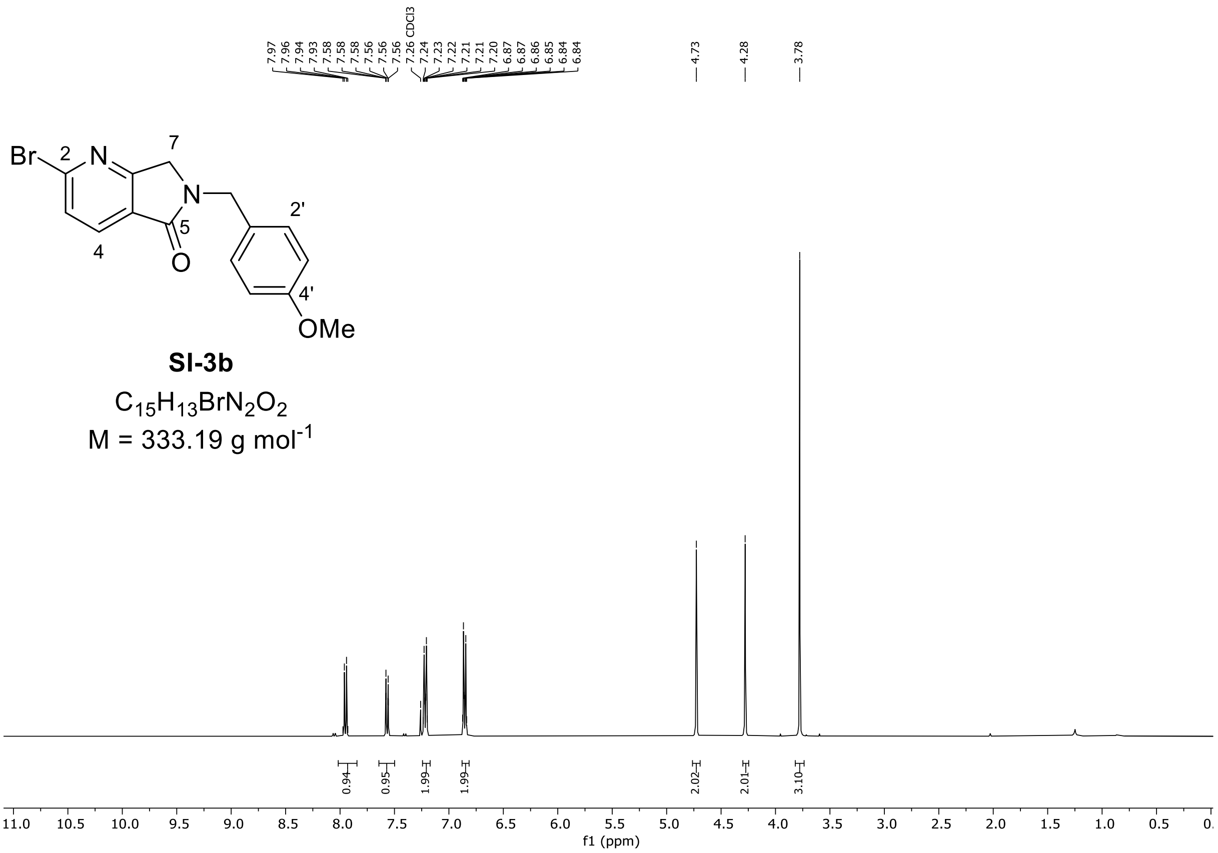

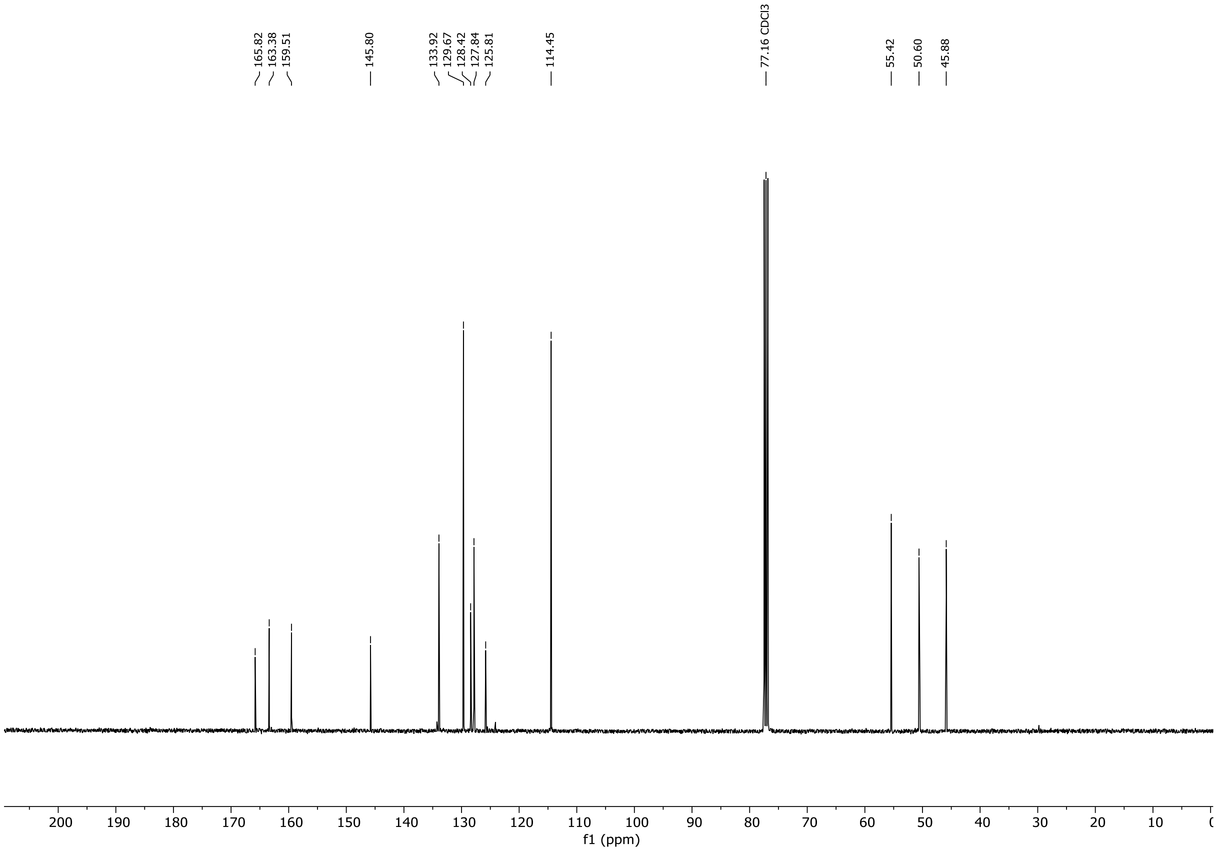

6-(4-Methoxybenzyl)-2-(trifluoromethyl)-6,7-dihydro-5*H*-pyrrolo[3,4-*b*]pyridin-5-one (SI-3c)

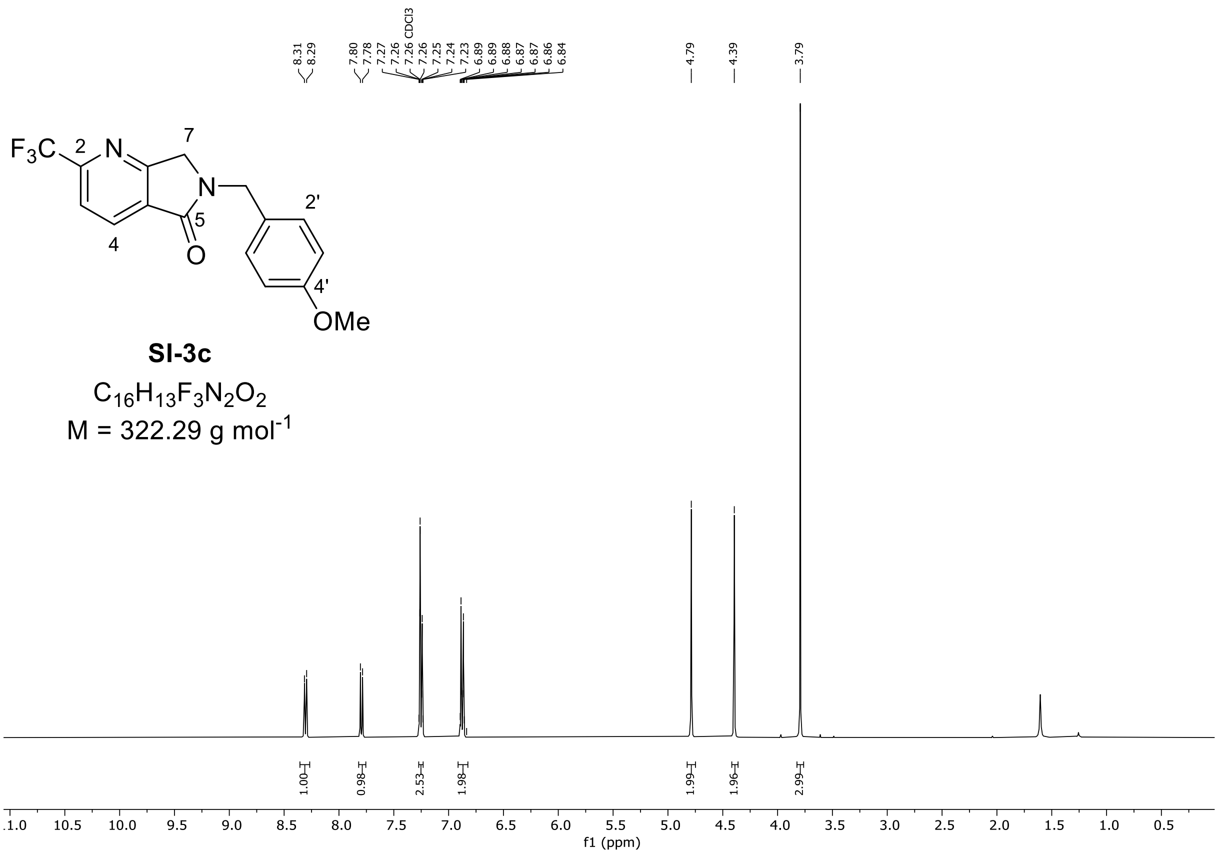

SI-3c
$C_{16}H_{13}F_3N_2O_2$
M = 322.29 g mol^{-1}

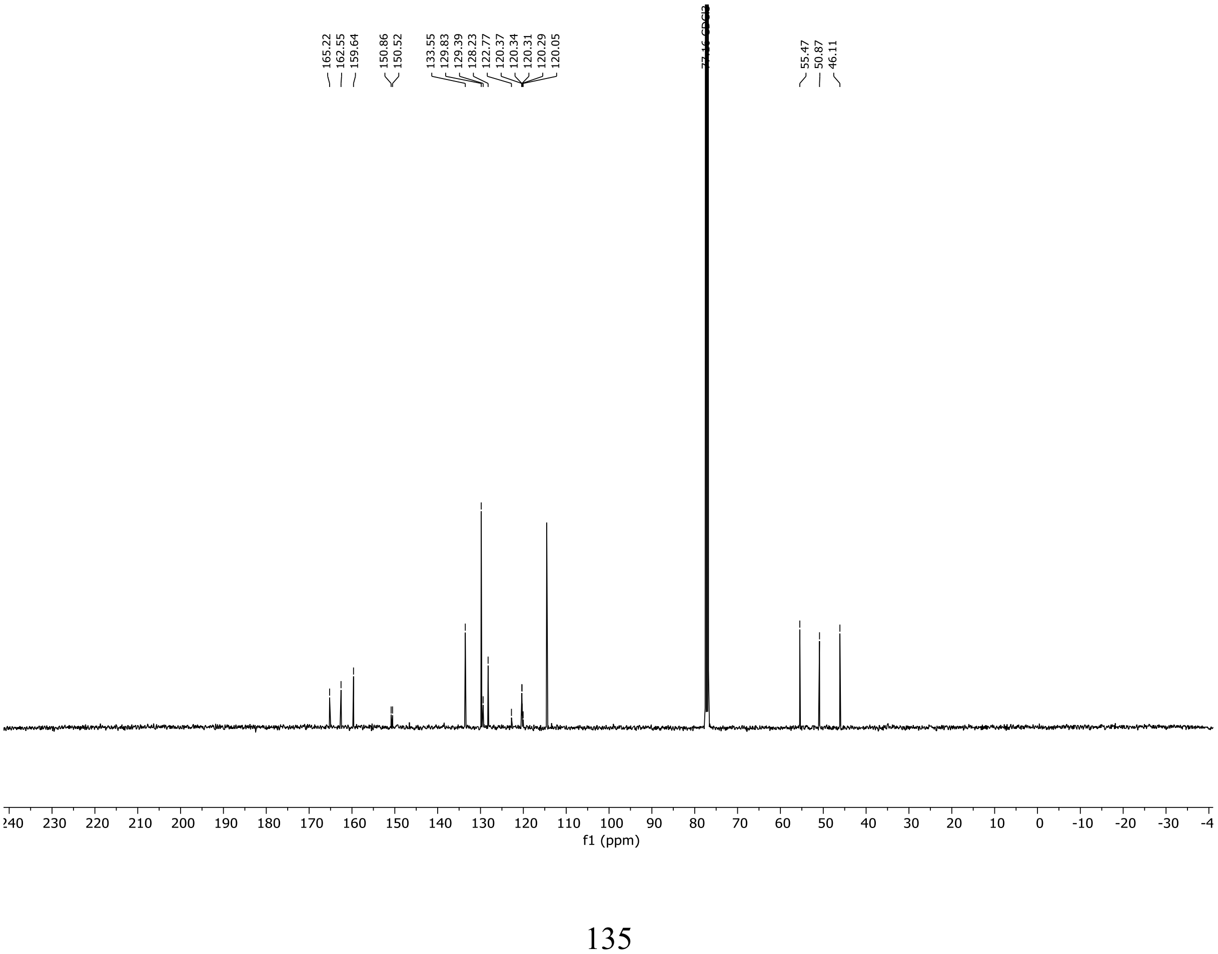

2-Chloro-6-(4-methoxybenzyl)-6,7-dihydro-5*H*-pyrrolo[3,4-*b*]pyridin-5-one (SI-3d)

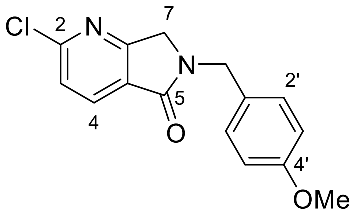

SI-3d

$C_{15}H_{13}ClN_2O_2$

M = 288.73 g mol^{-1}

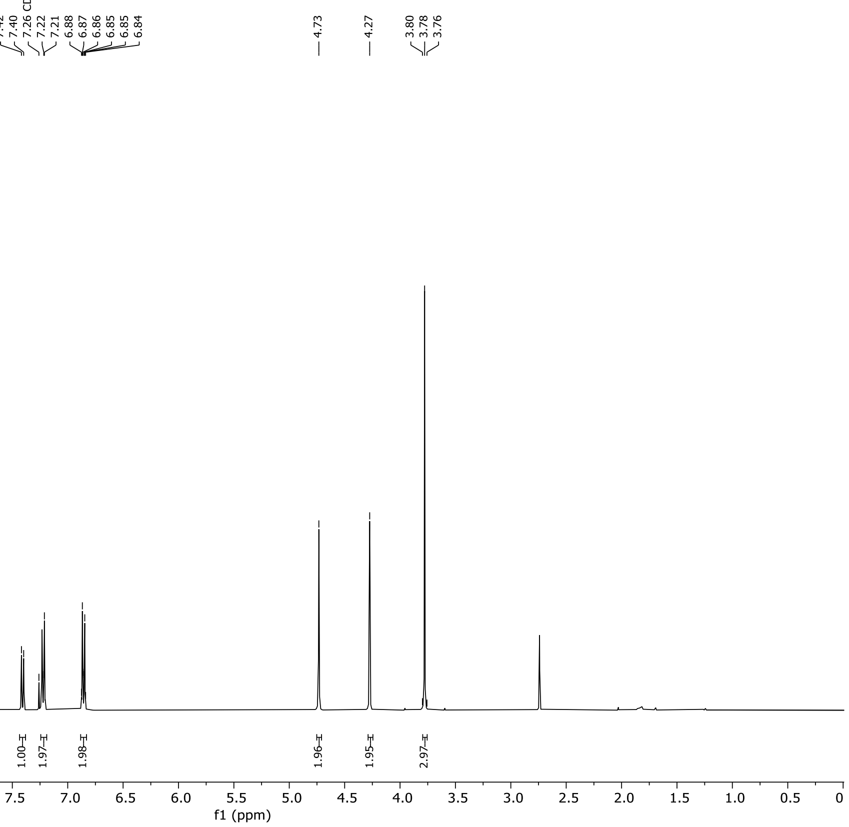

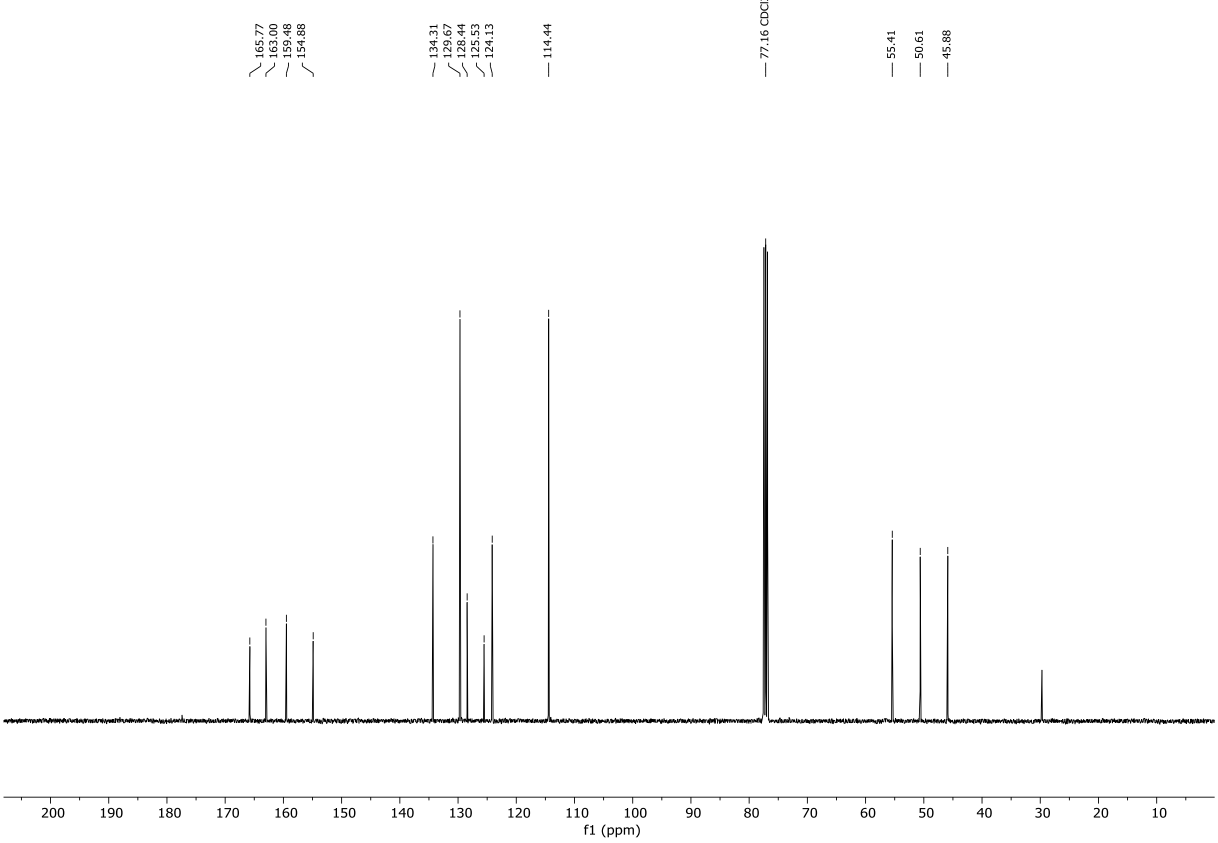

3-Bromo-6-(4-methoxybenzyl)-6,7-dihydro-5*H*-pyrrolo[3,4-*b*]pyridin-5-one (SI-3e)

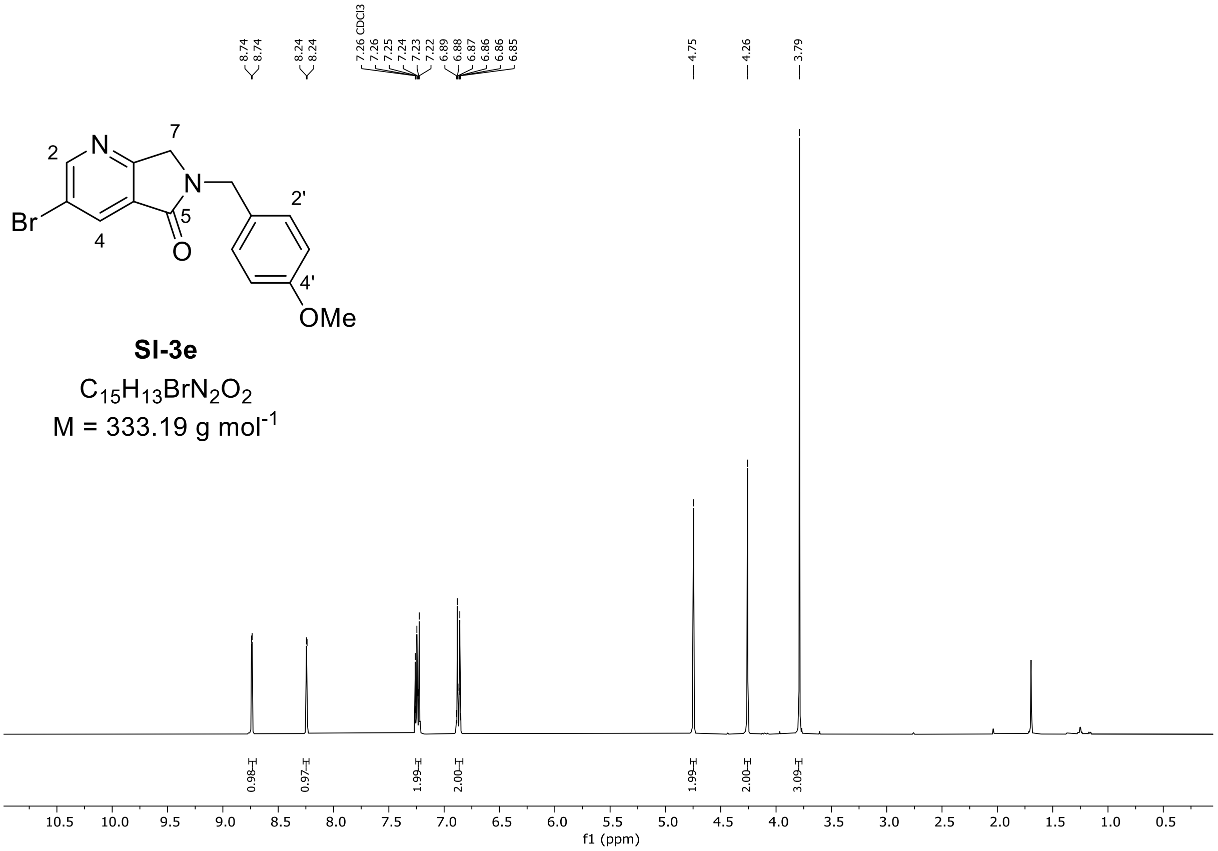

SI-3e

$C_{15}H_{13}BrN_2O_2$

M = 333.19 g mol^{-1}

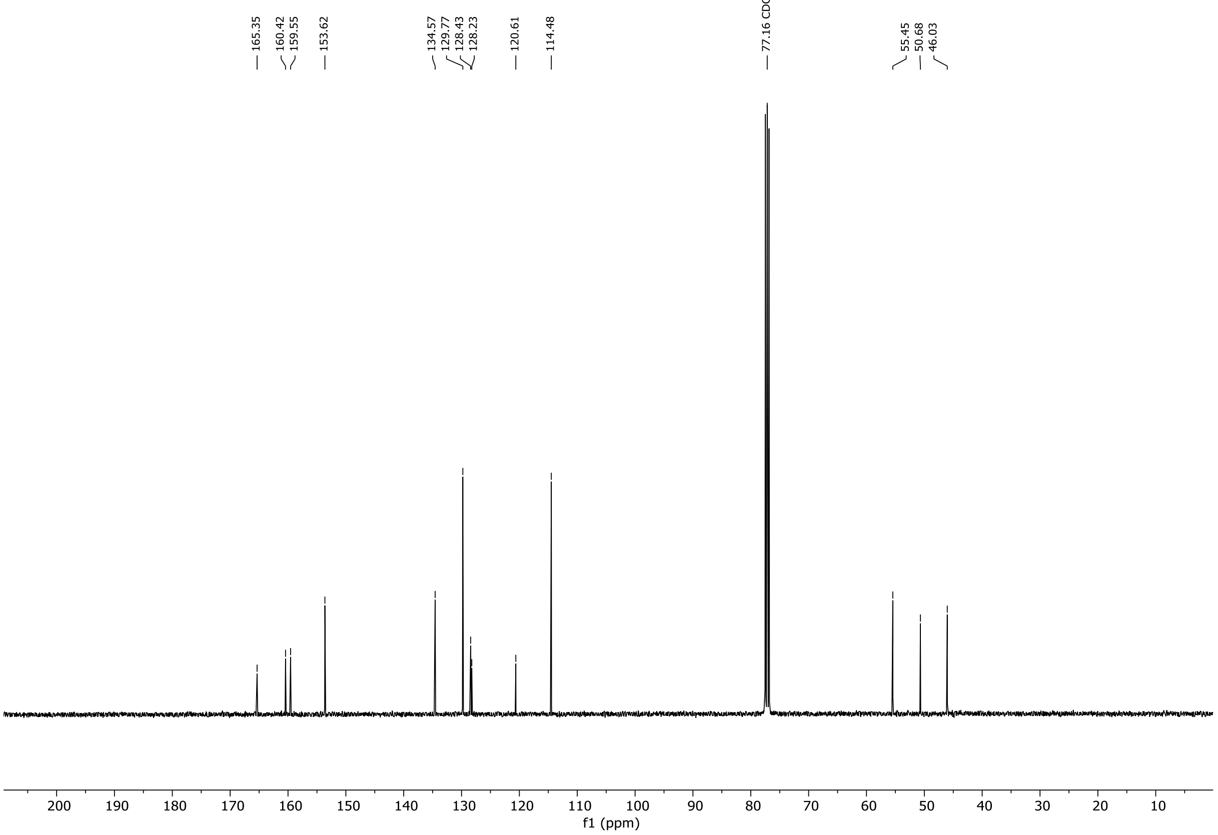

3-Chloro-6-(4-methoxybenzyl)-6,7-dihydro-5*H*-pyrrolo[3,4-*b*]pyridin-5-one (SI-3f)

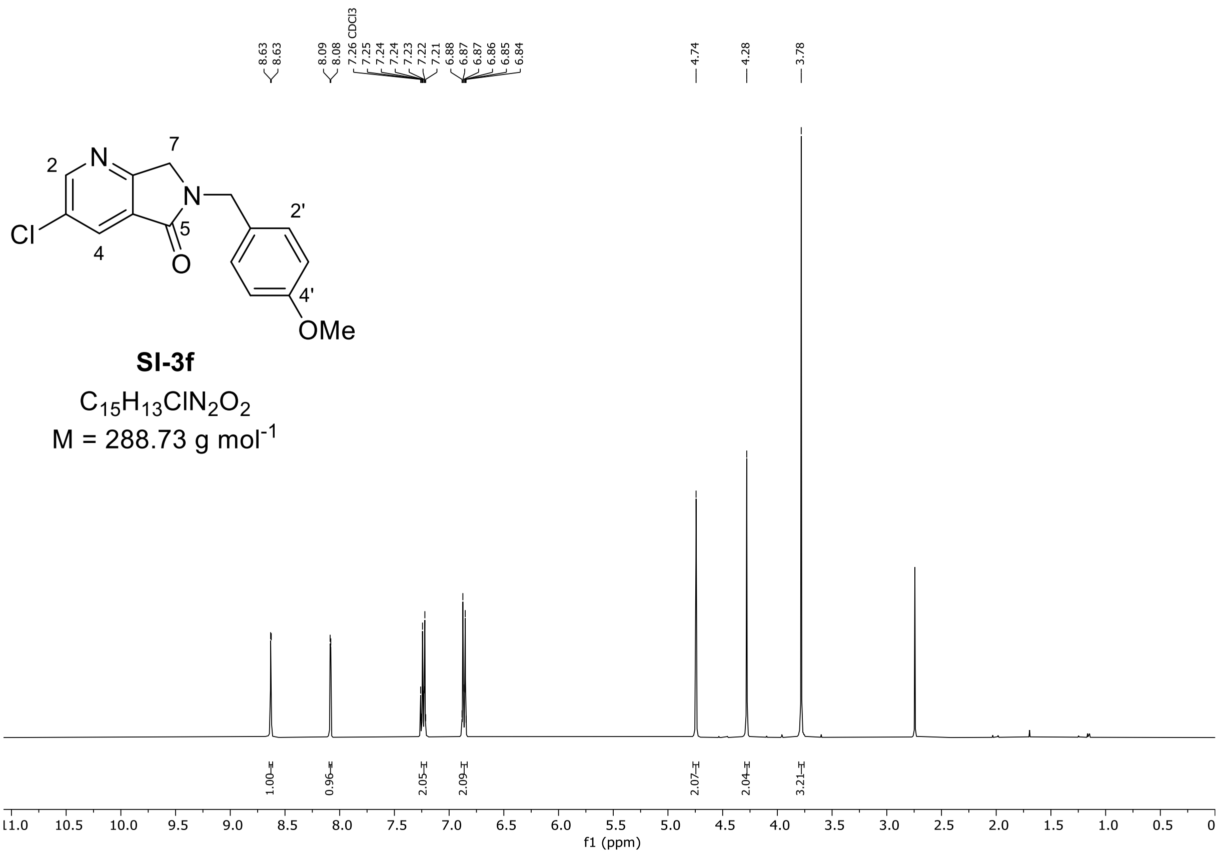

SI-3f

$C_{15}H_{13}ClN_2O_2$

M = 288.73 g mol^{-1}

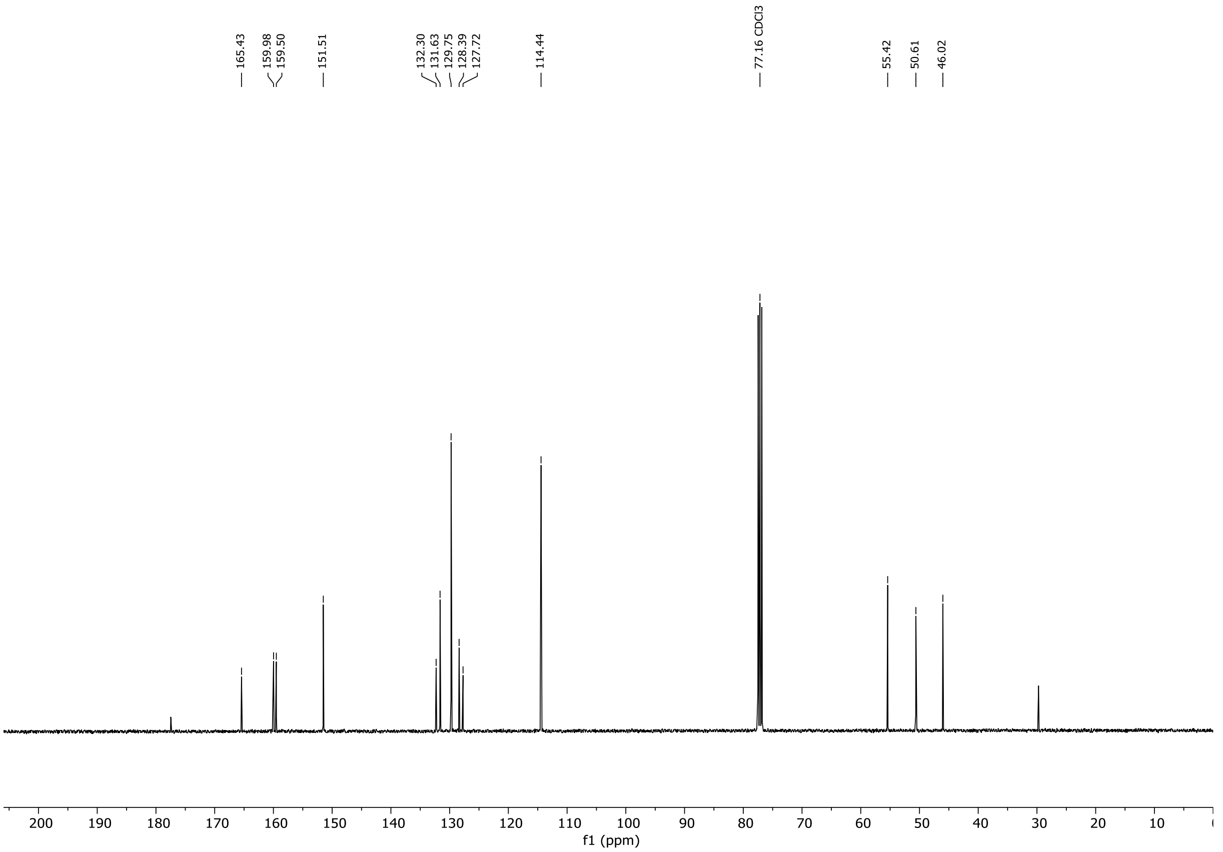

7-Benzyl-6,7-dihydro-5*H*-pyrrolo[3,4-*b*]pyridin-5-one (*rac*-3a)

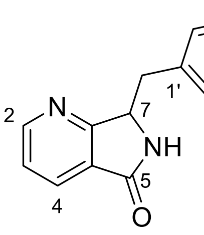

rac-**3a**

$C_{14}H_{12}N_2O$

M = 224.26 g mol^{-1}

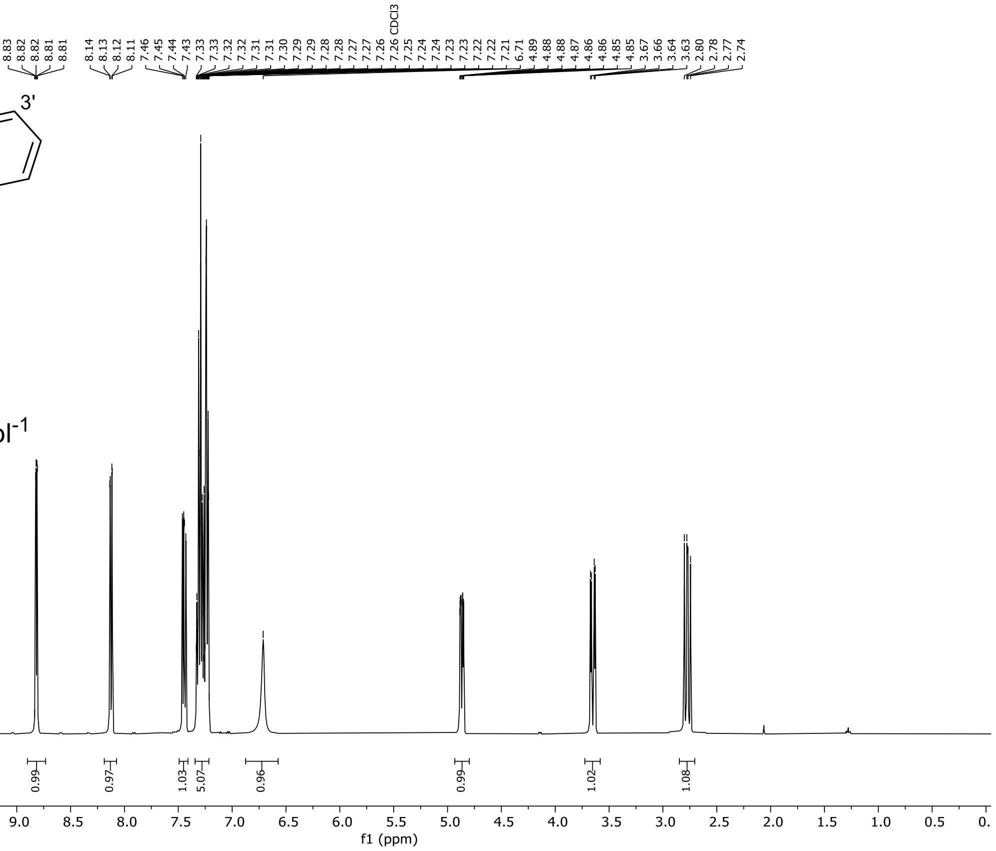

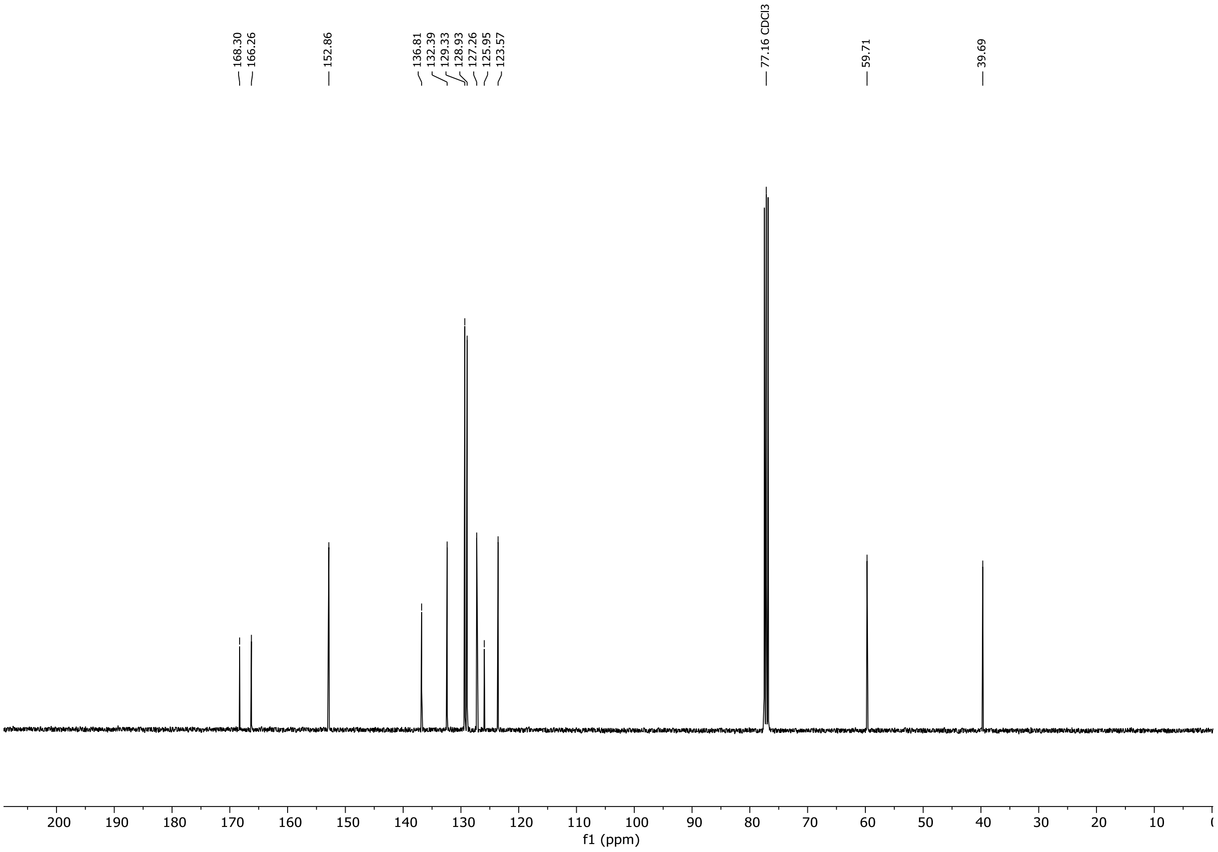

7-(4-(*tert*-Butyl)benzyl)-6,7-dihydro-5*H*-pyrrolo[3,4-*b*]pyridin-5-one (*rac*-3b)

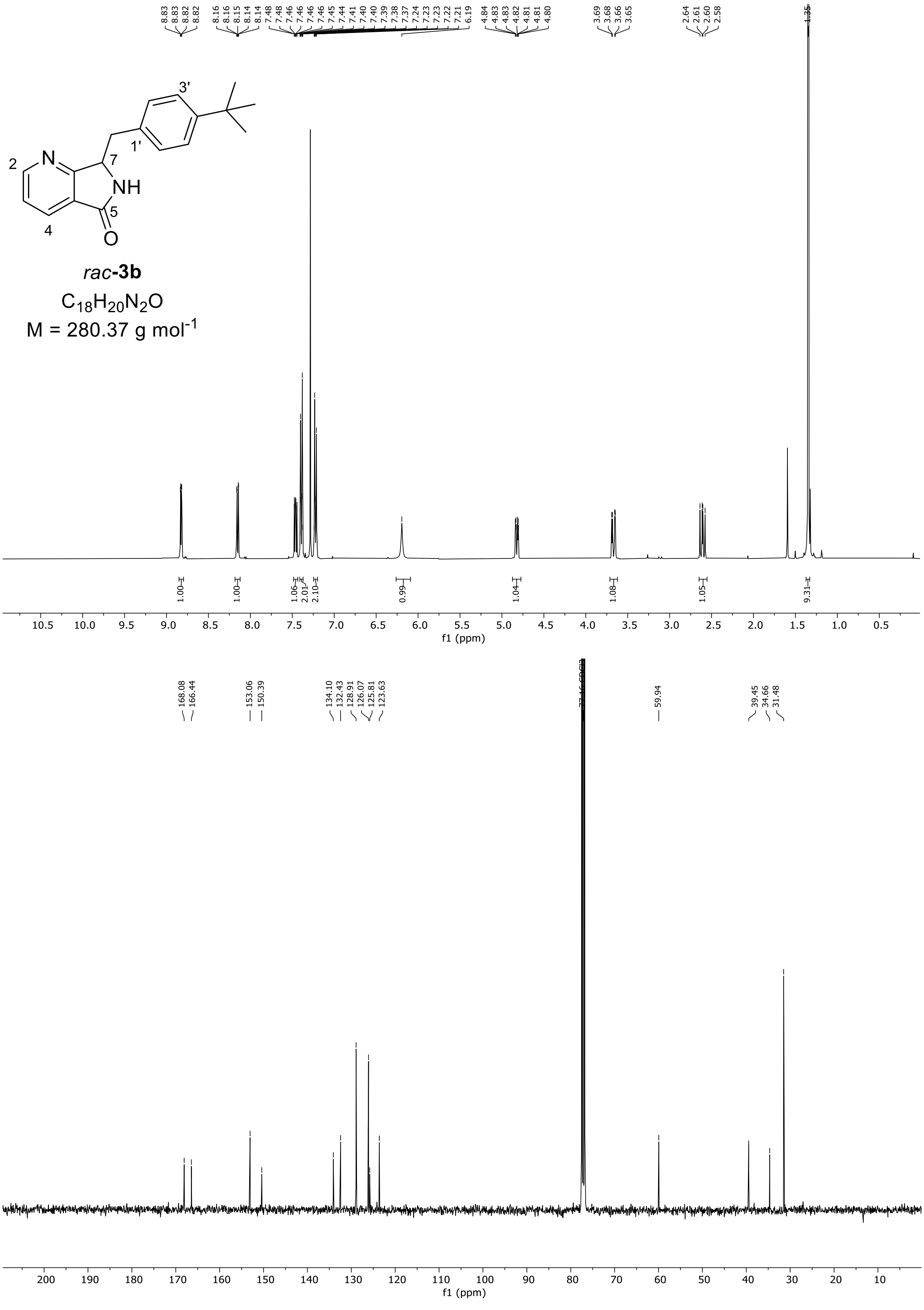

7-(3,5-Dimethylbenzyl)-6,7-dihydro-5*H*-pyrrolo[3,4-*b*]pyridin-5-one (*rac*-3c)

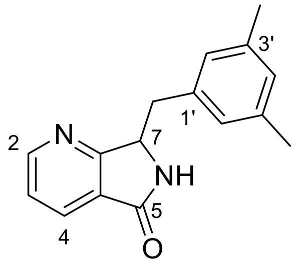

rac-**3c**

$C_{16}H_{16}N_2O$

M = 252.32 g mol^{-1}

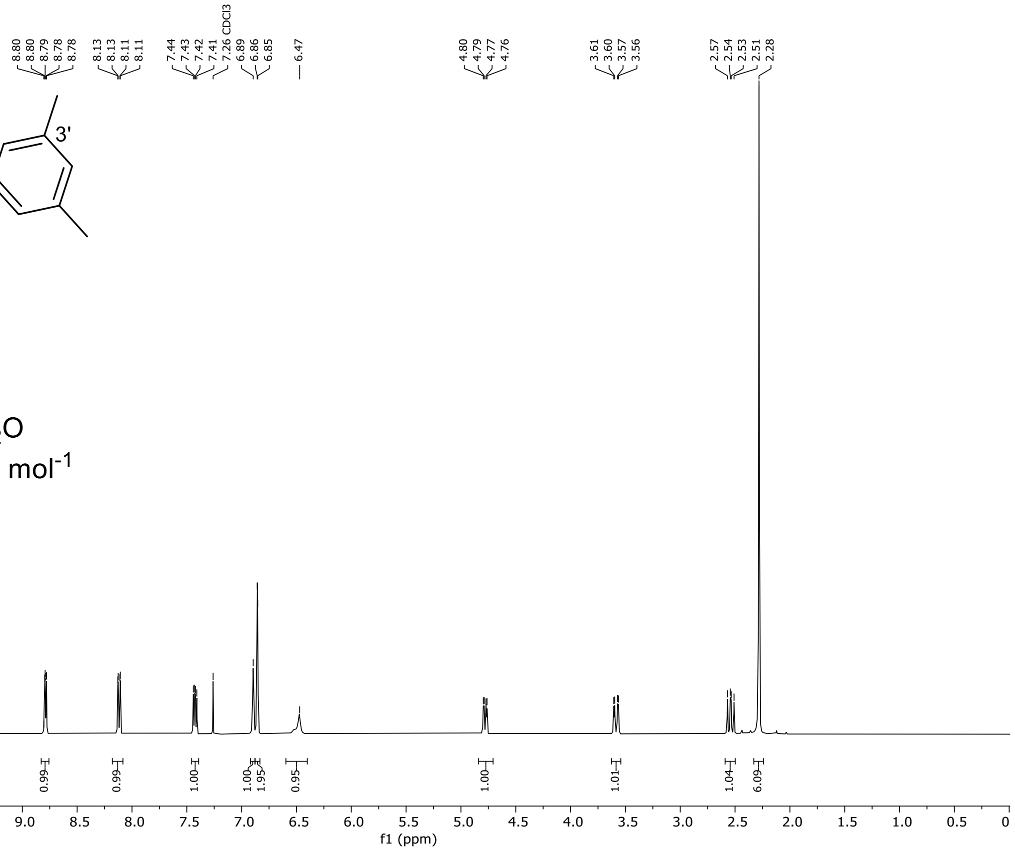

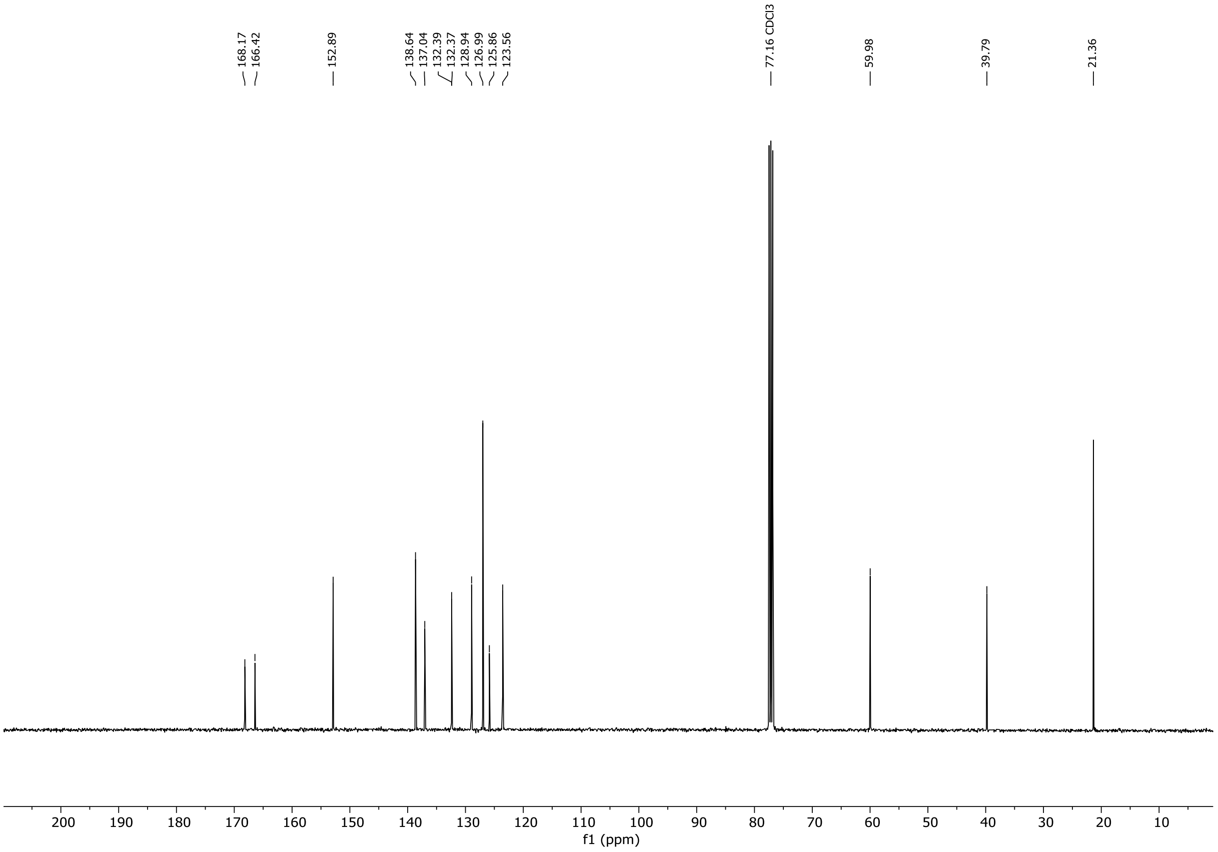

7-(4-Fluorobenzyl)-6,7-dihydro-5*H*-pyrrolo[3,4-*b*]pyridin-5-one (*rac*-3d)

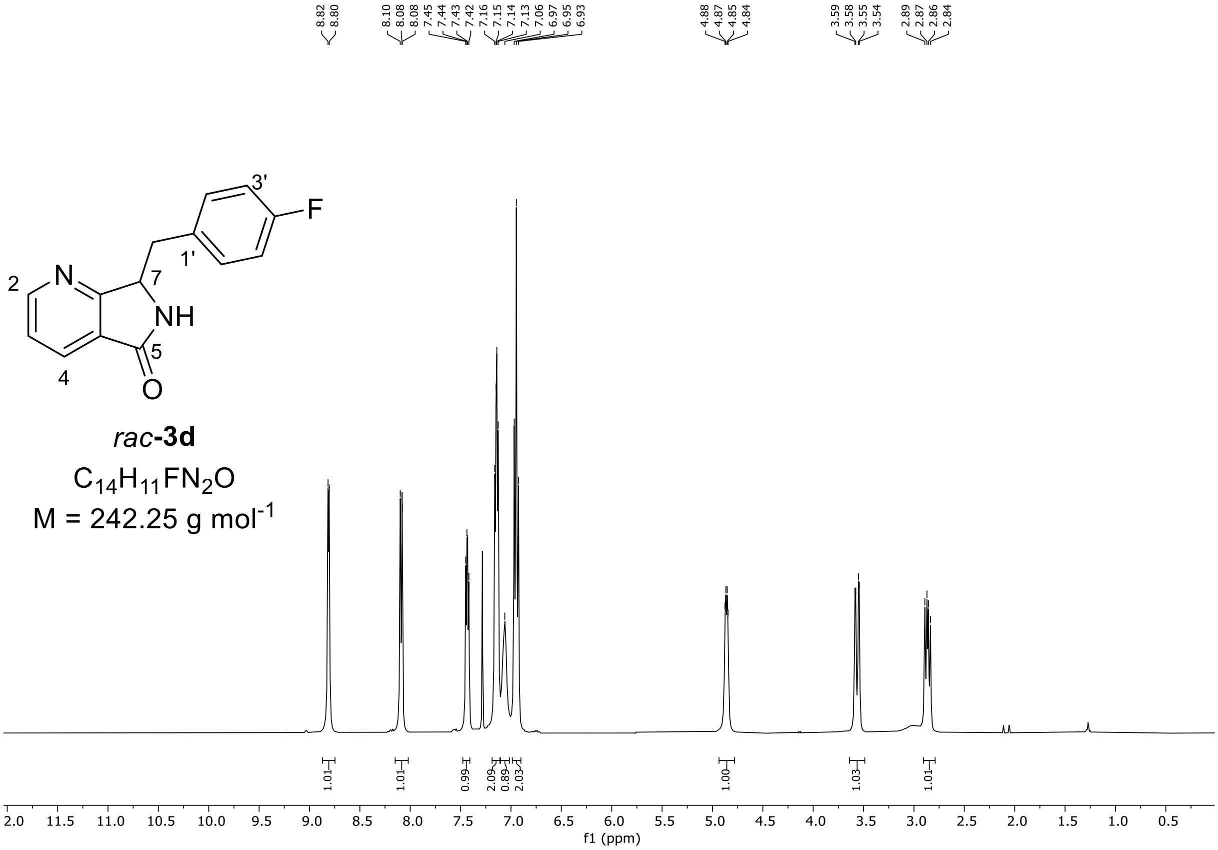

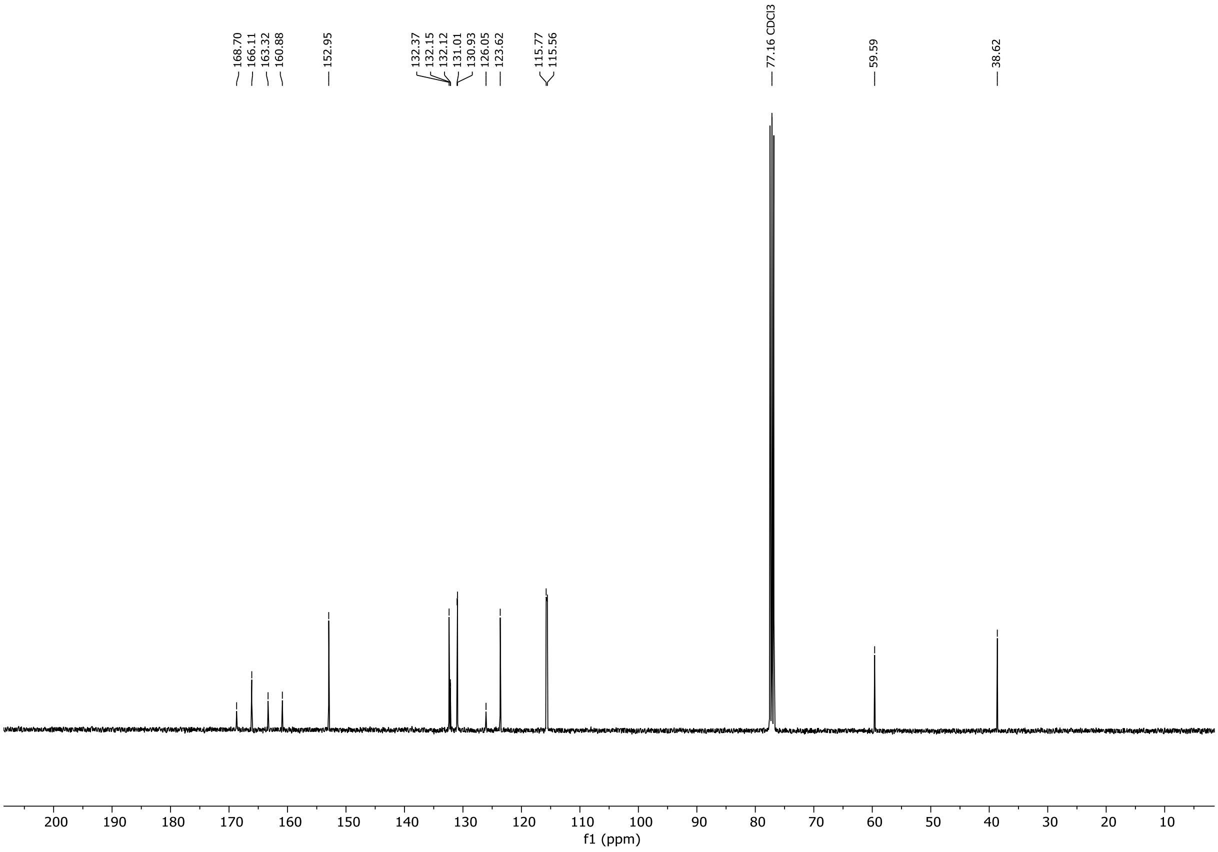

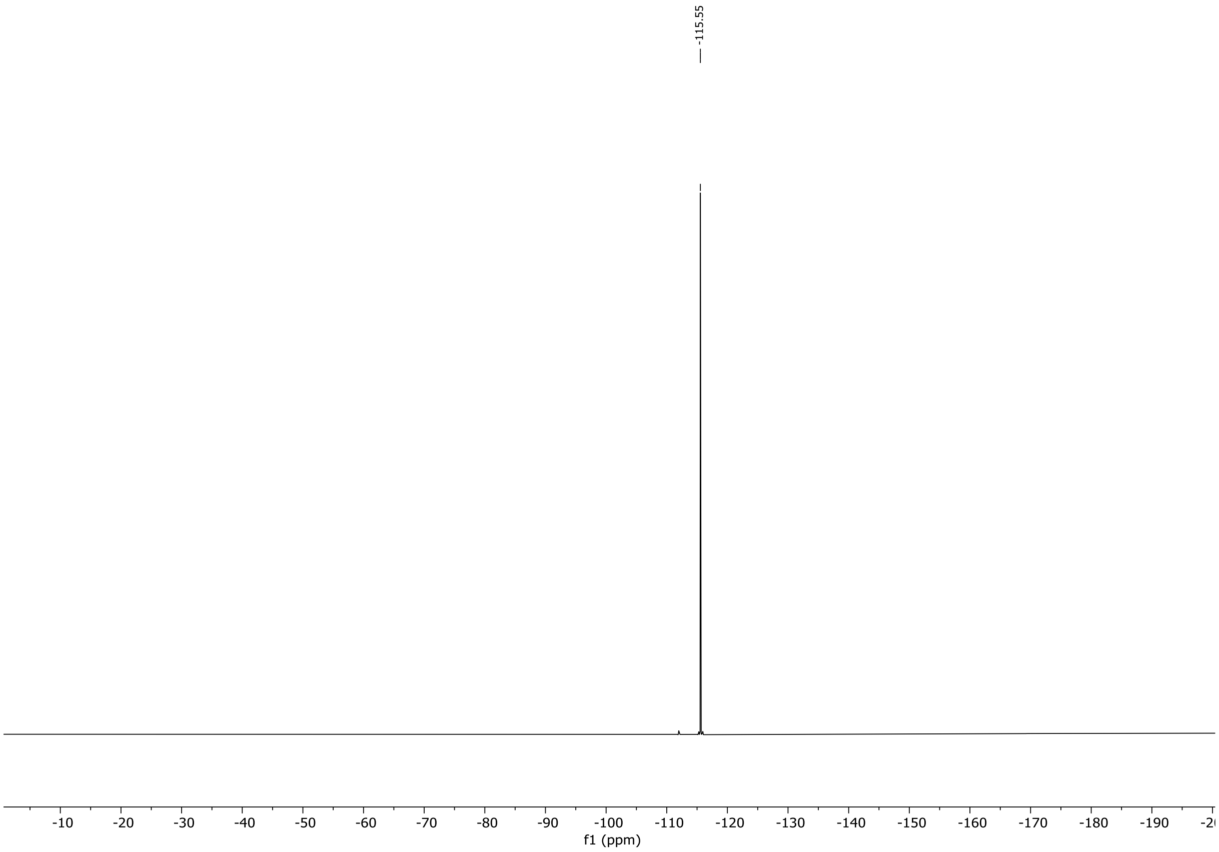
-115.55
-10
-20
-30
-40
-50
-60
-70
-80
-90
-100
-110
-120
-130
-140
-150
-160
-170
-180
-190
f1 (ppm)

7-(4-Bromobenzyl)-6,7-dihydro-5*H*-pyrrolo[3,4-*b*]pyridin-5-one (*rac*-**3e**)

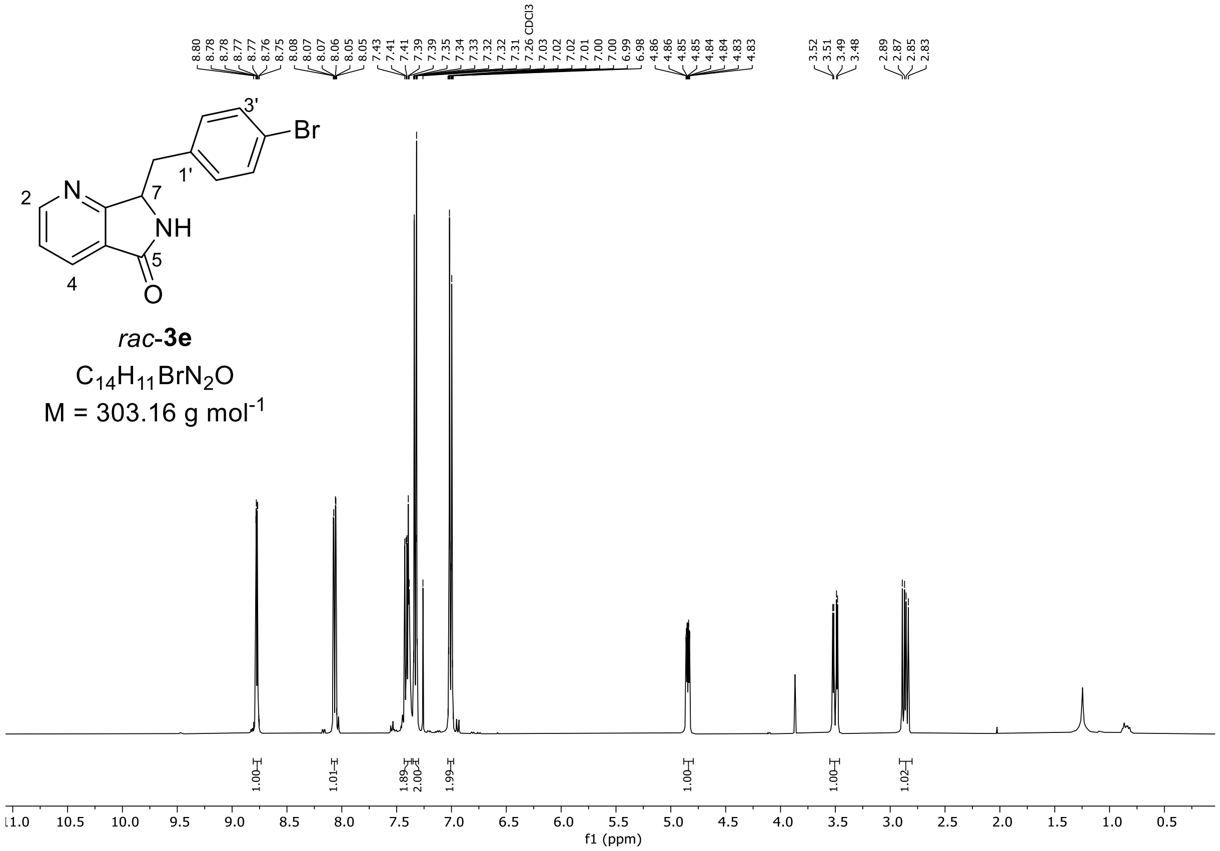

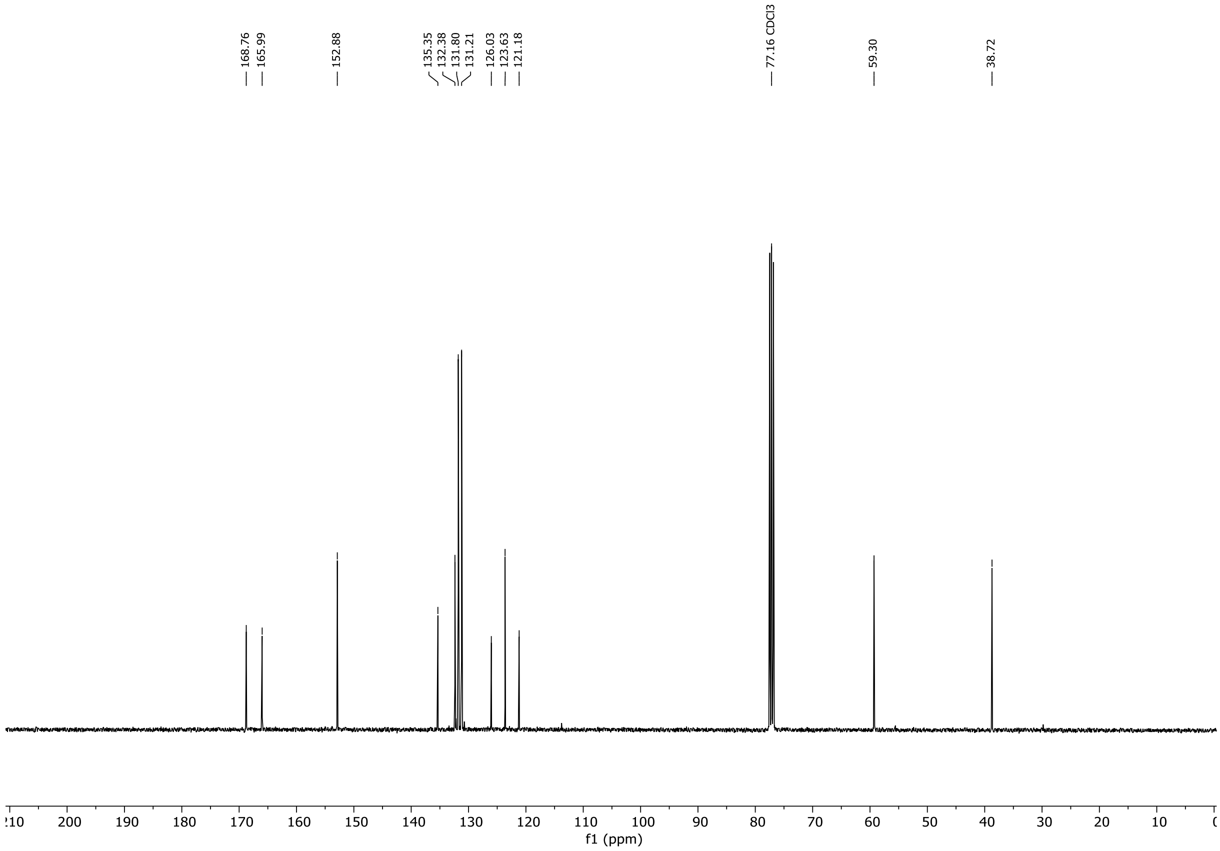

7-(Cyclopropylmethyl)-6,7-dihydro-5*H*-pyrrolo[3,4-*b*]pyridin-5-one (*rac*-3f)

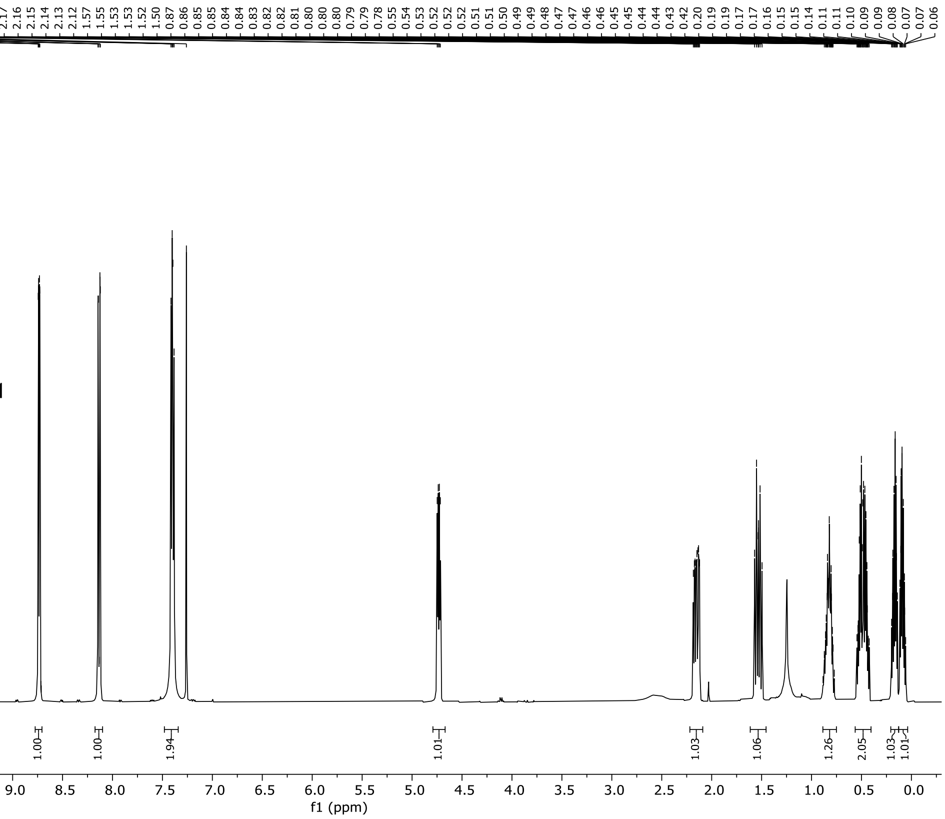

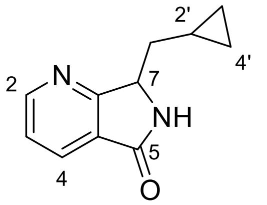

rac-**3f**

$C_{11}H_{12}N_2O$

M = 188.23 g mol^{-1}

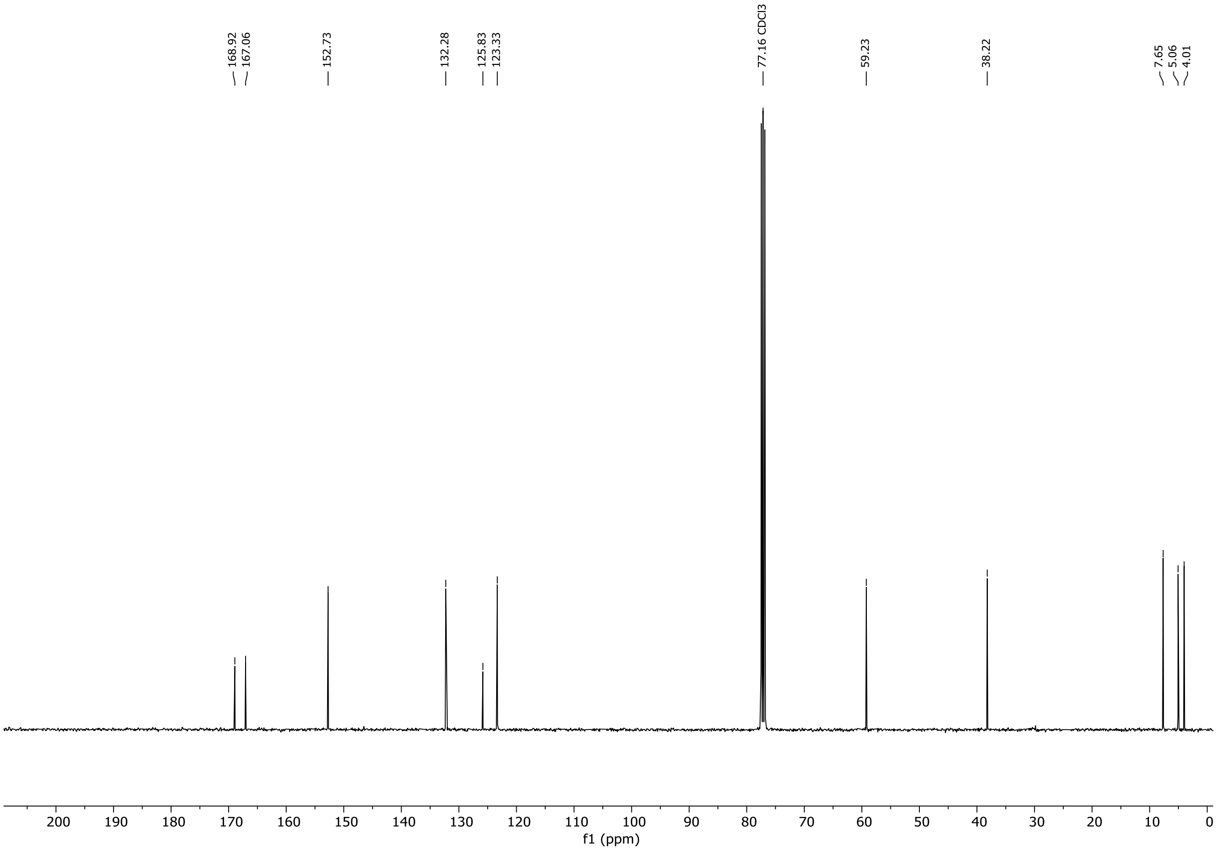

7-(Cyclobutylmethyl)-6,7-dihydro-5*H*-pyrrolo[3,4-*b*]pyridin-5-one (*rac*-3g)

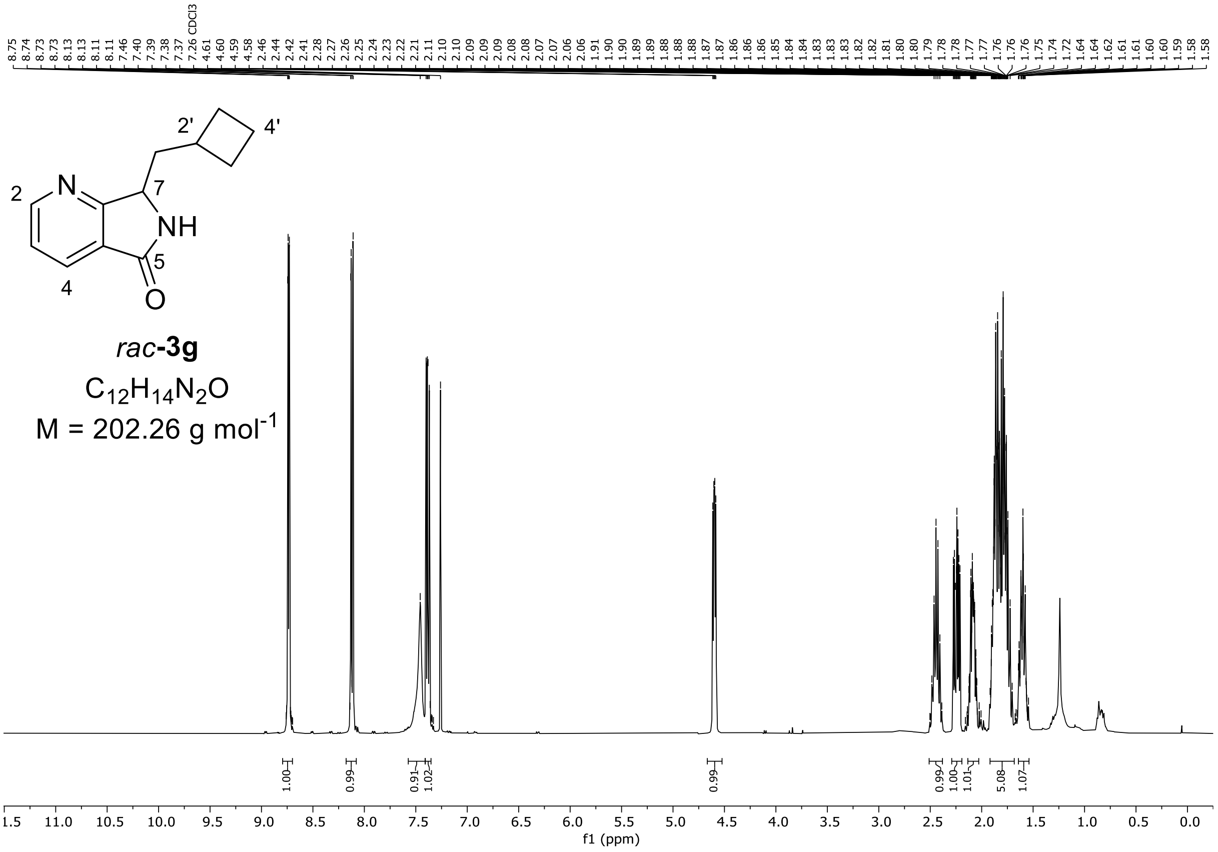

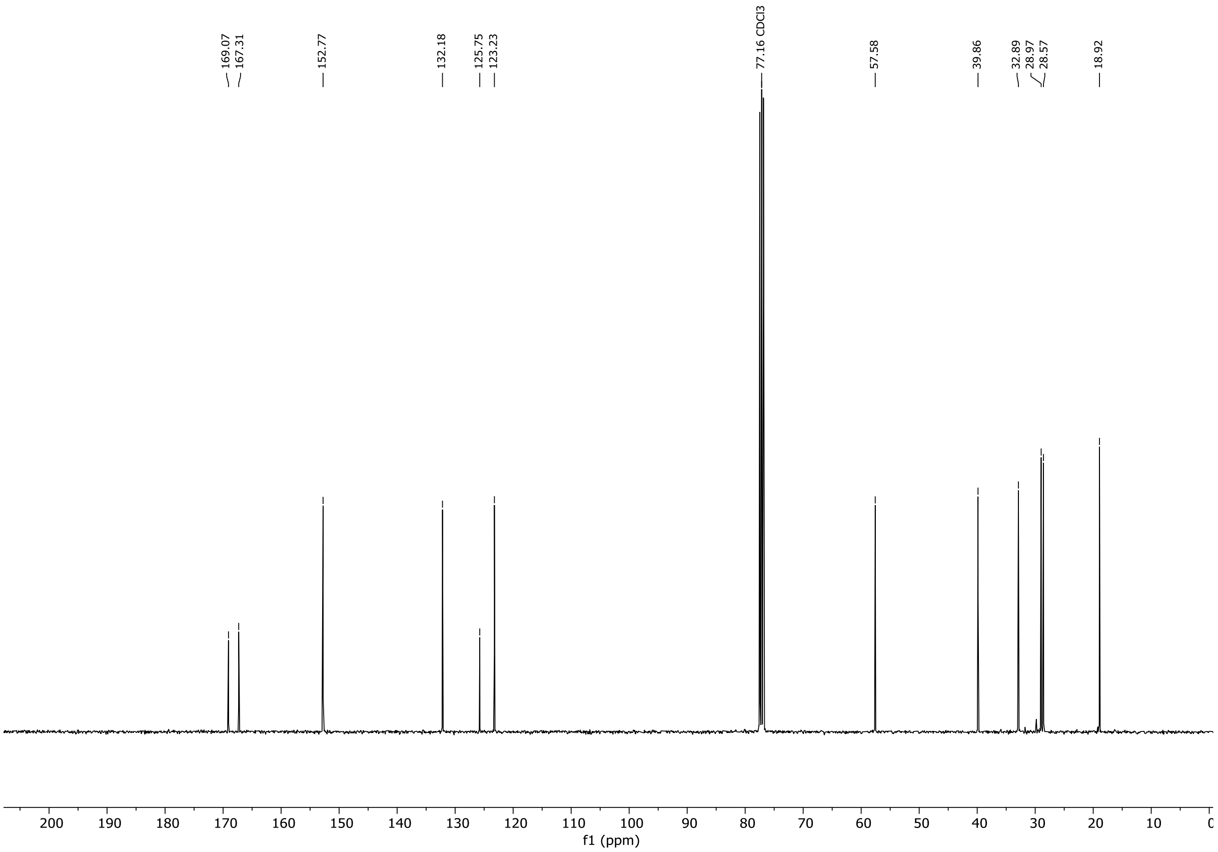

***tert*-Butyl 3-((5-oxo-6,7-dihydro-5*H*-pyrrolo[3,4-*b*]pyridin-7-yl)methyl)azetidine-1-carboxylate (*rac*-3h)**

rac-**3h**

$C_{16}H_{21}N_3O_3$

M = 303.36 g mol^{-1}

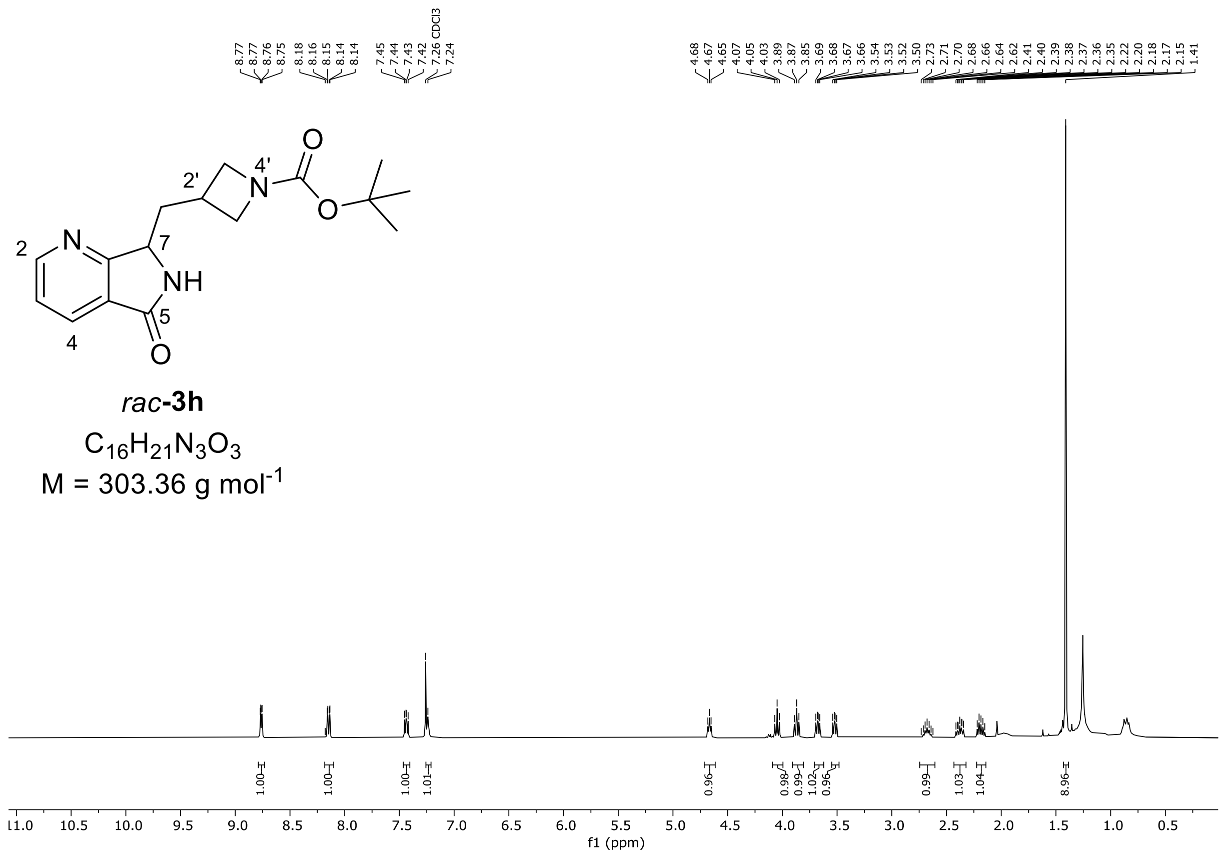

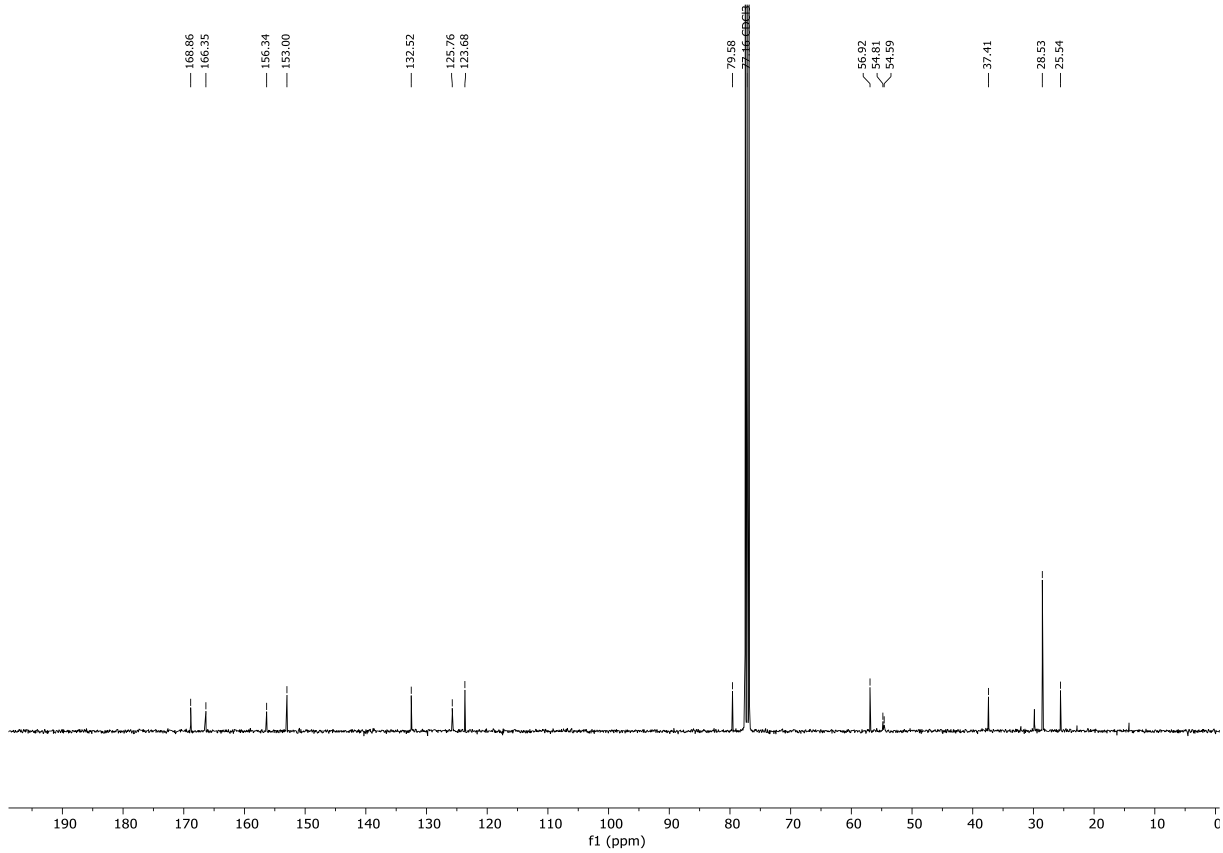

7-Ethyl-6,7-dihydro-5*H*-pyrrolo[3,4-*b*]pyridin-5-one (*rac*-3i)

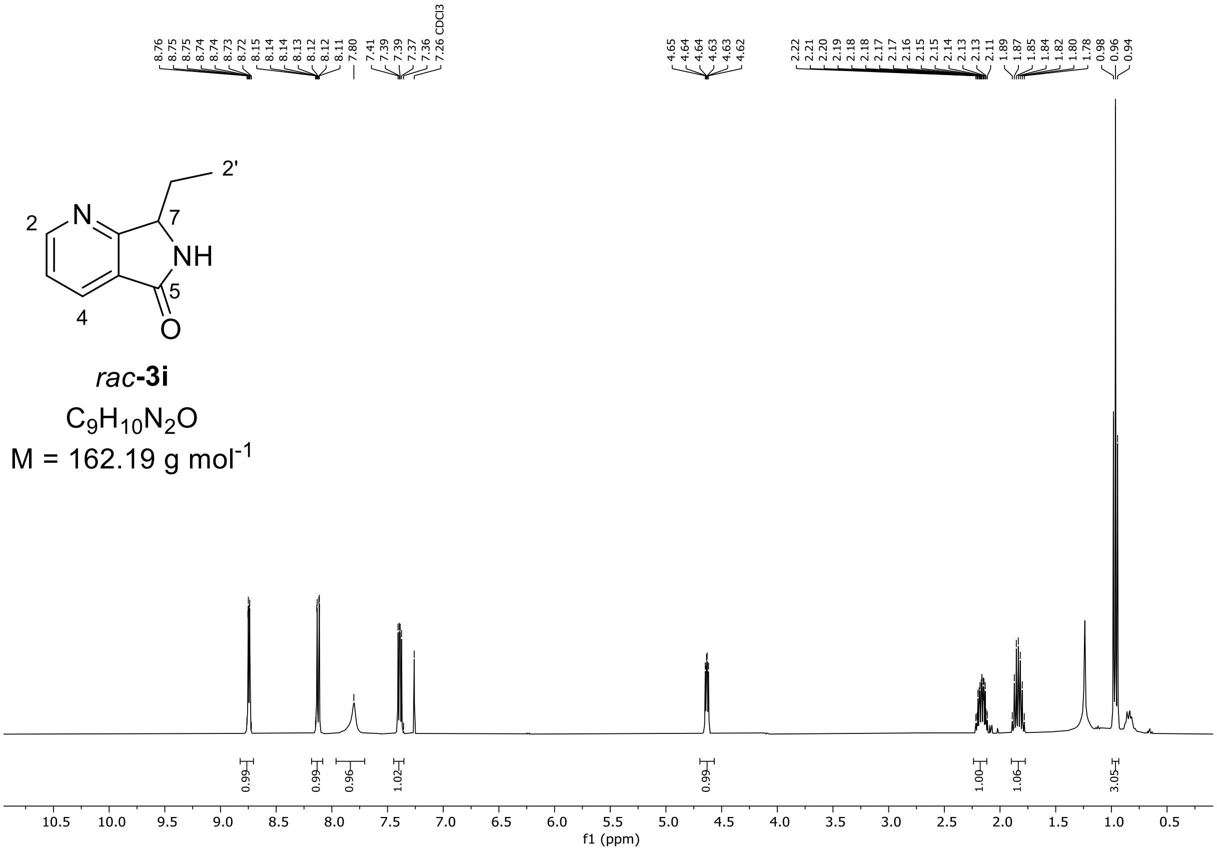

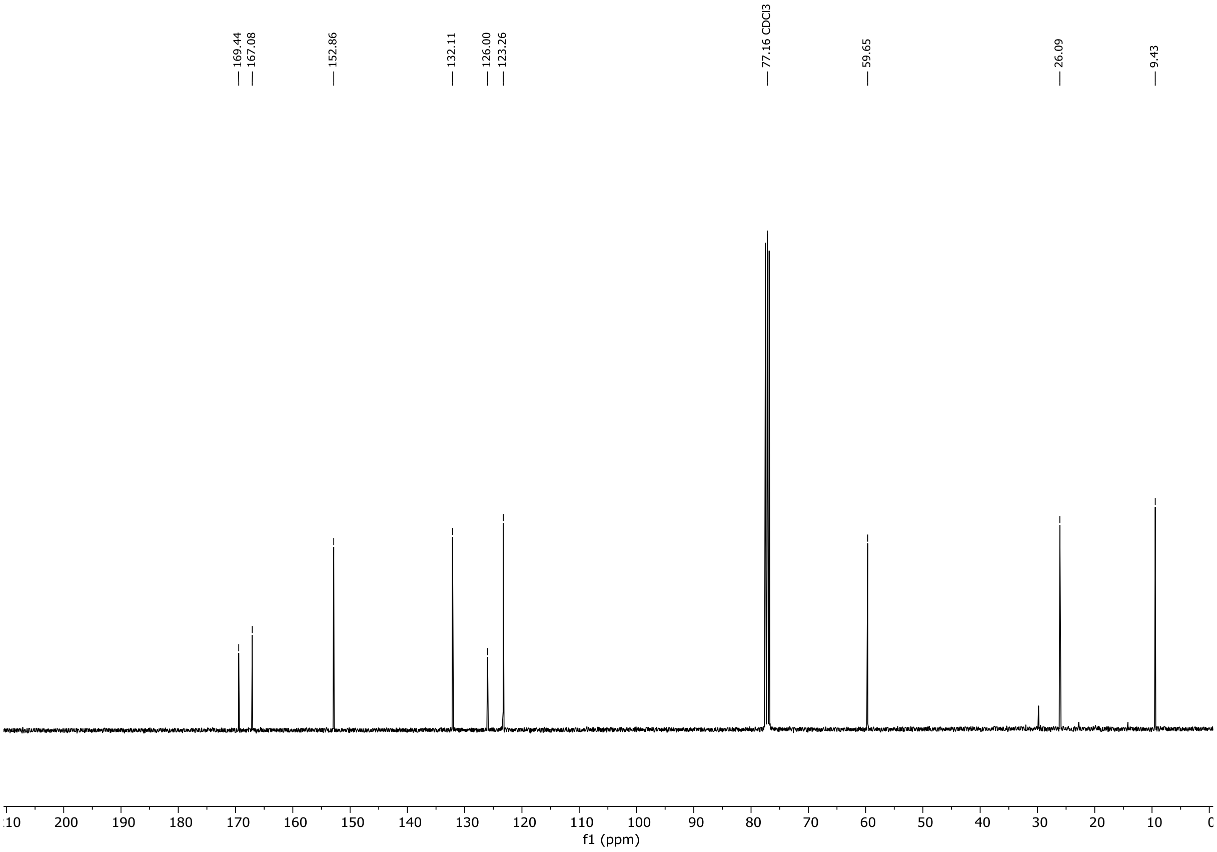

7-Butyl-6,7-dihydro-5*H*-pyrrolo[3,4-*b*]pyridin-5-one (*rac*-3j)

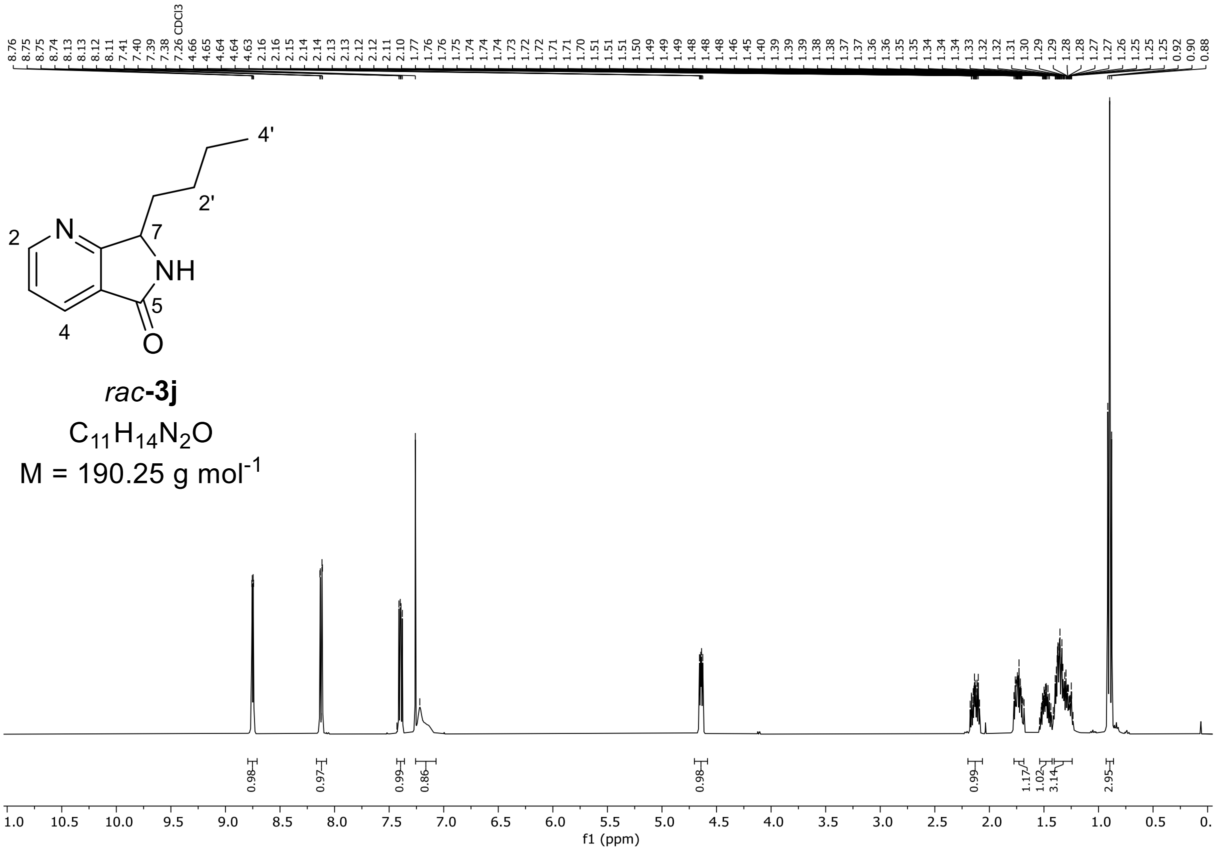

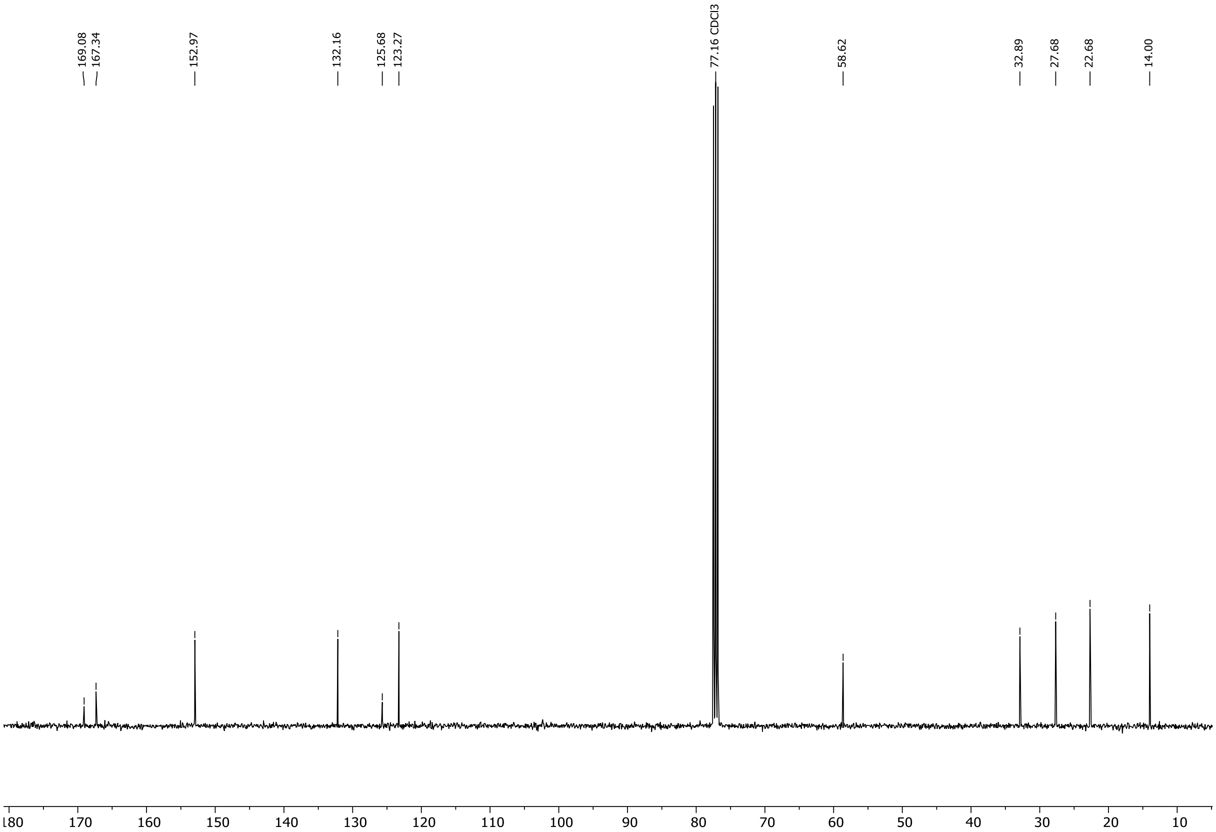

2-Chloro-7-isopentyl-6,7-dihydro-5*H*-pyrrolo[3,4-*b*]pyridin-5-one (*rac*-3k)

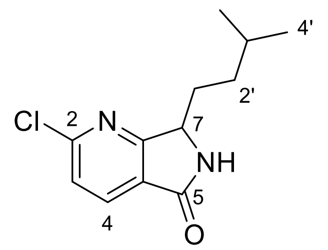

rac-**3k**

$C_{12}H_{15}ClN_2O$

M = 238.72 g mol^{-1}

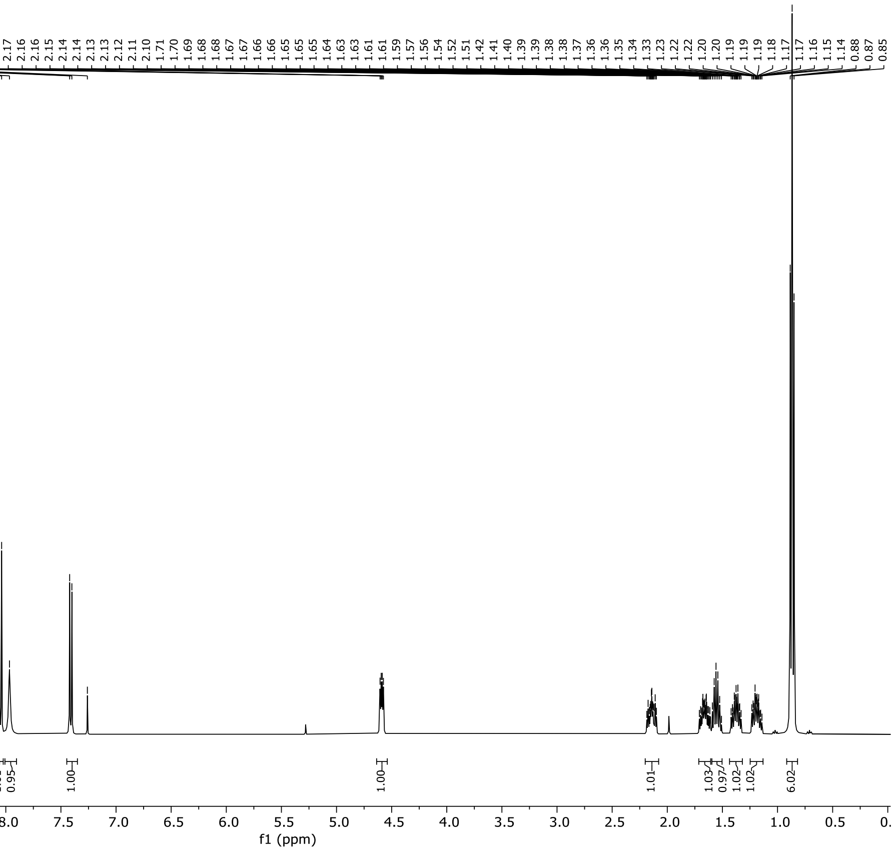

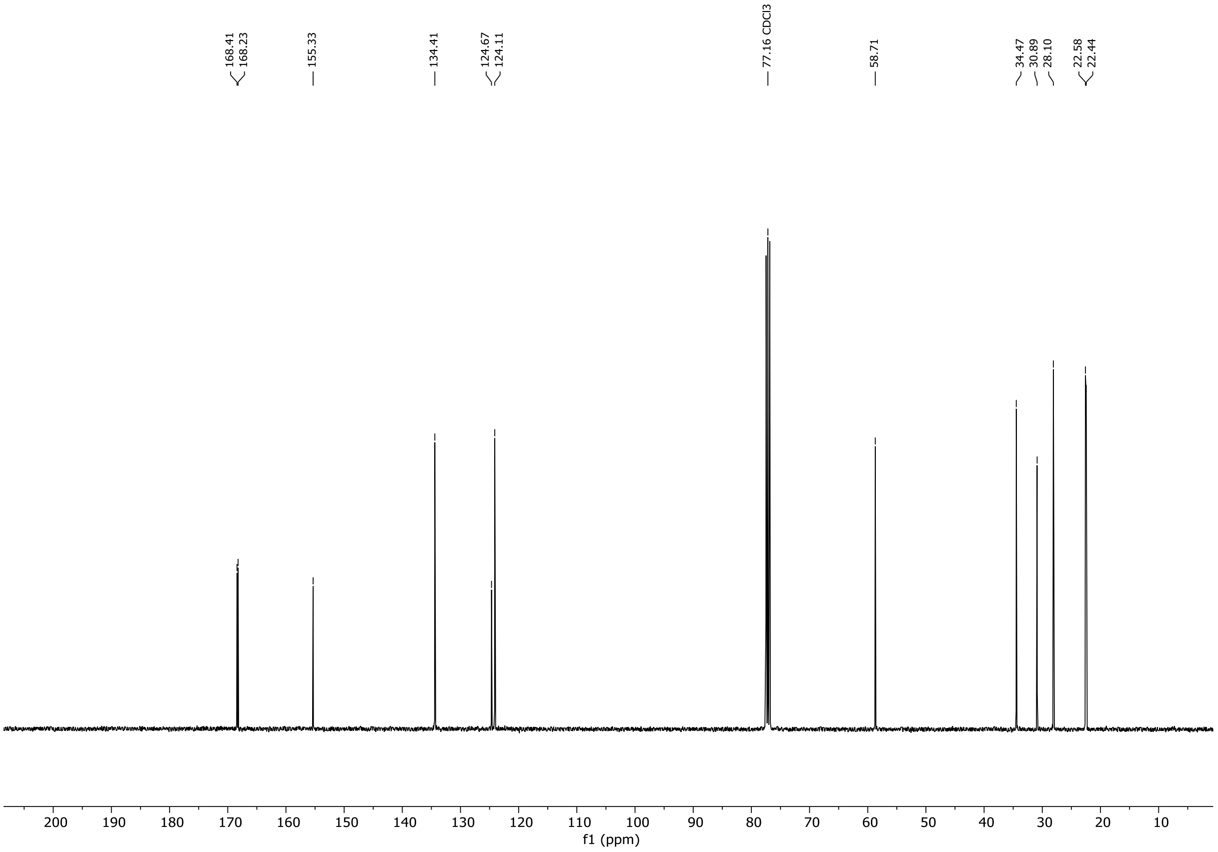

7-Benzyl-2-bromo-6,7-dihydro-5*H*-pyrrolo[3,4-*b*]pyridin-5-one (*rac*-3l)

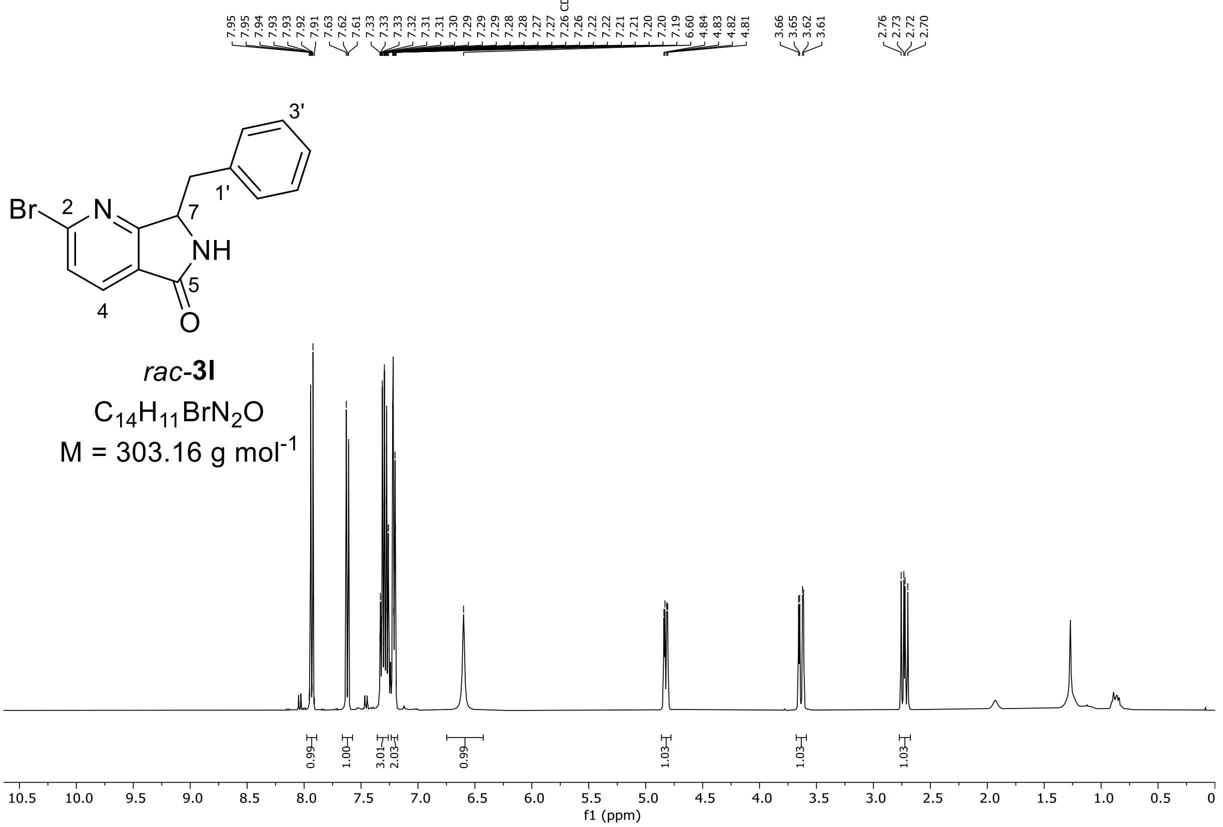

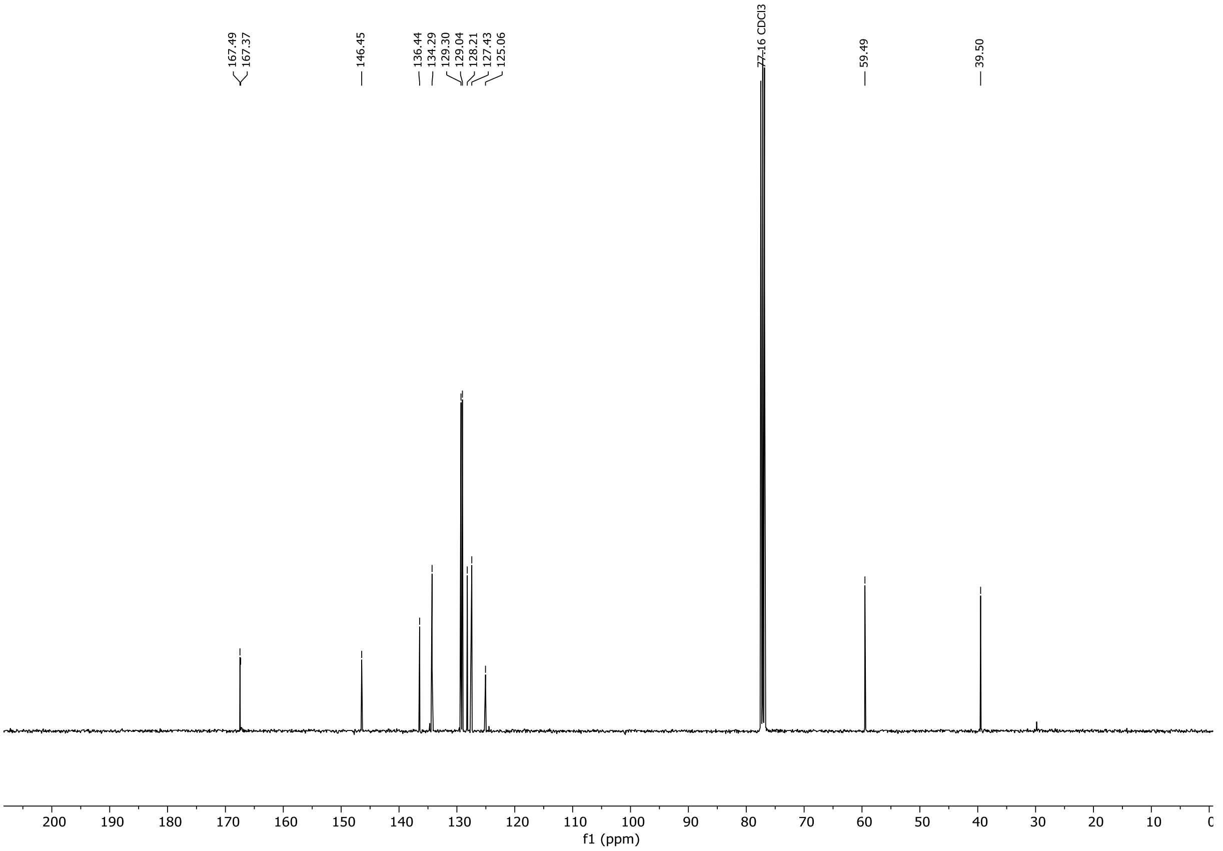

7-Benzyl-2-(trifluoromethyl)-6,7-dihydro-5*H*-pyrrolo[3,4-*b*]pyridin-5-one (*rac*-3m)

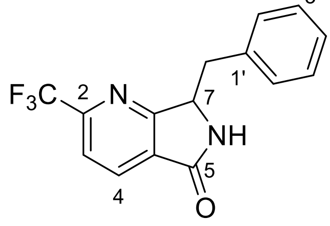

rac-**3m**

$C_{15}H_{11}F_3N_2O$

M = 292.26 g mol^{-1}

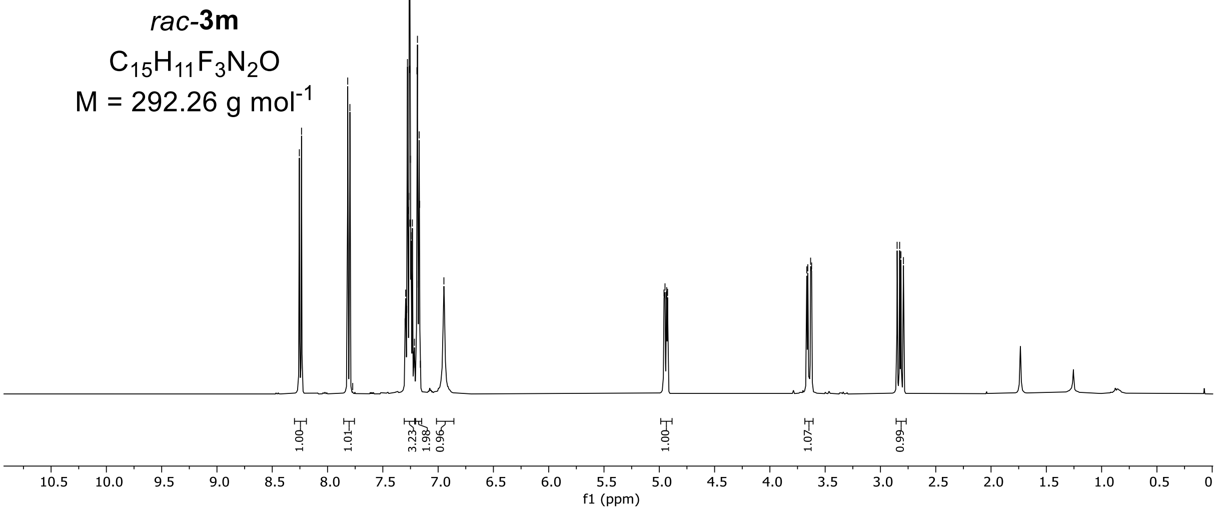

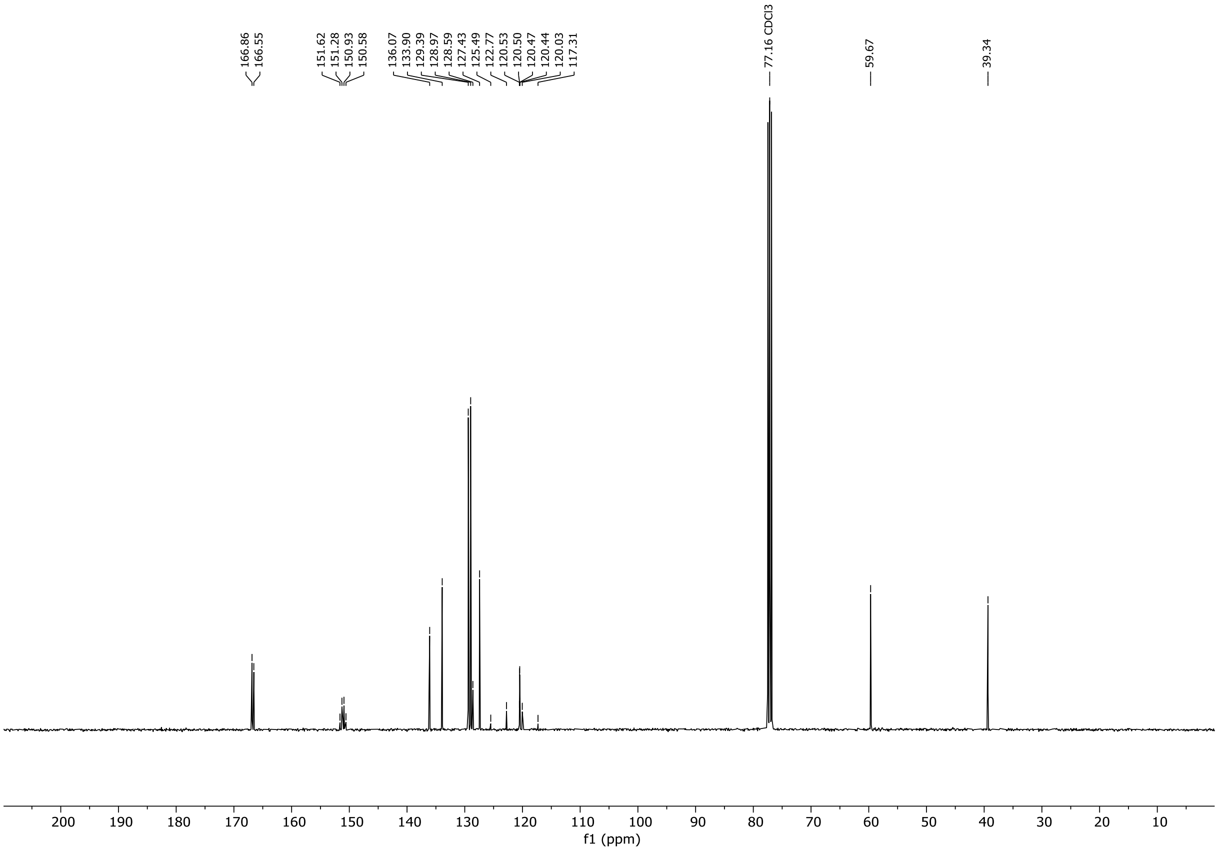

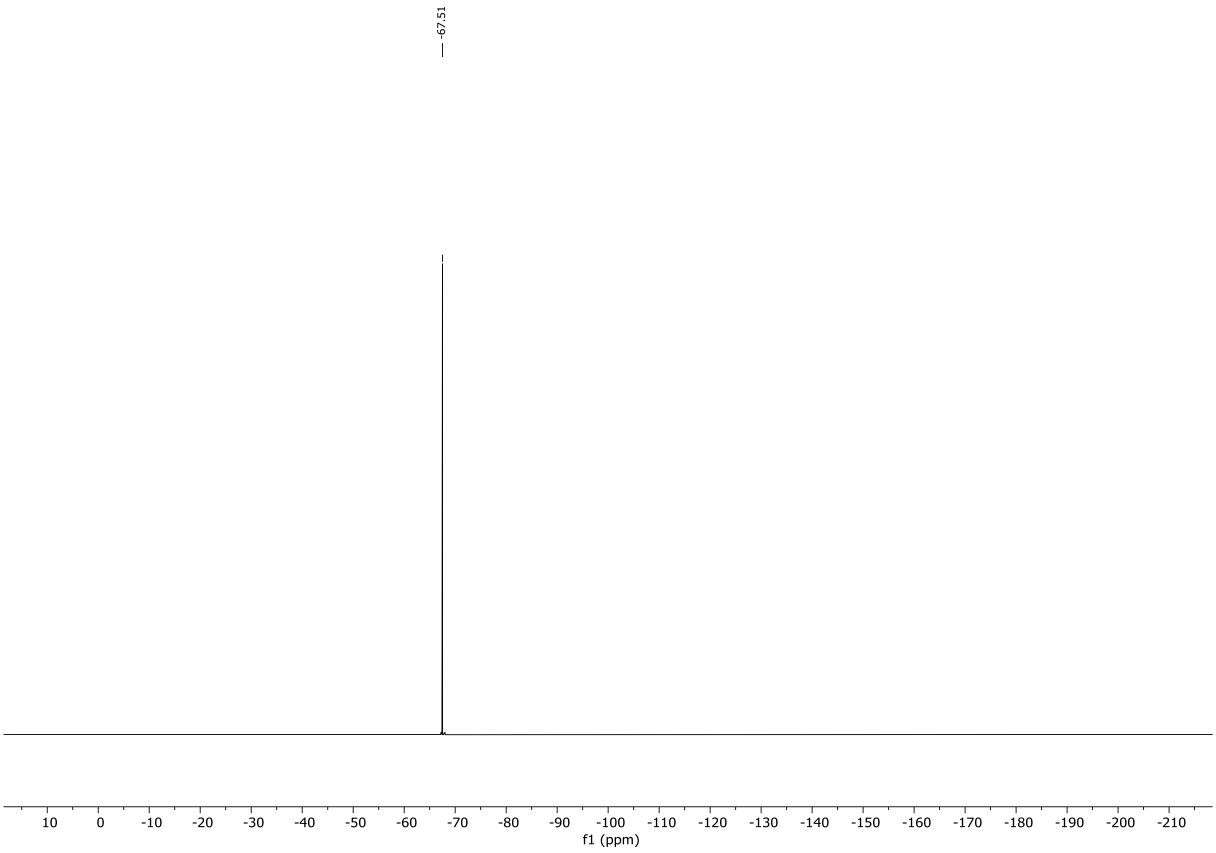
-67.51
10 0 -10 -20 -30 -40 -50 -60 -70 -80 -90 -100 -110 -120 -130 -140 -150 -160 -170 -180 -190 -200 -210
f1 (ppm)

7-Benzyl-2-chloro-6,7-dihydro-5*H*-pyrrolo[3,4-*b*]pyridin-5-one (*rac*-**3n**)

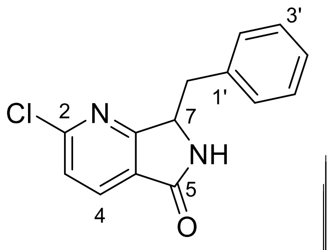

rac-**3n**

$C_{14}H_{11}ClN_2O$

M = 258.71 g mol^{-1}

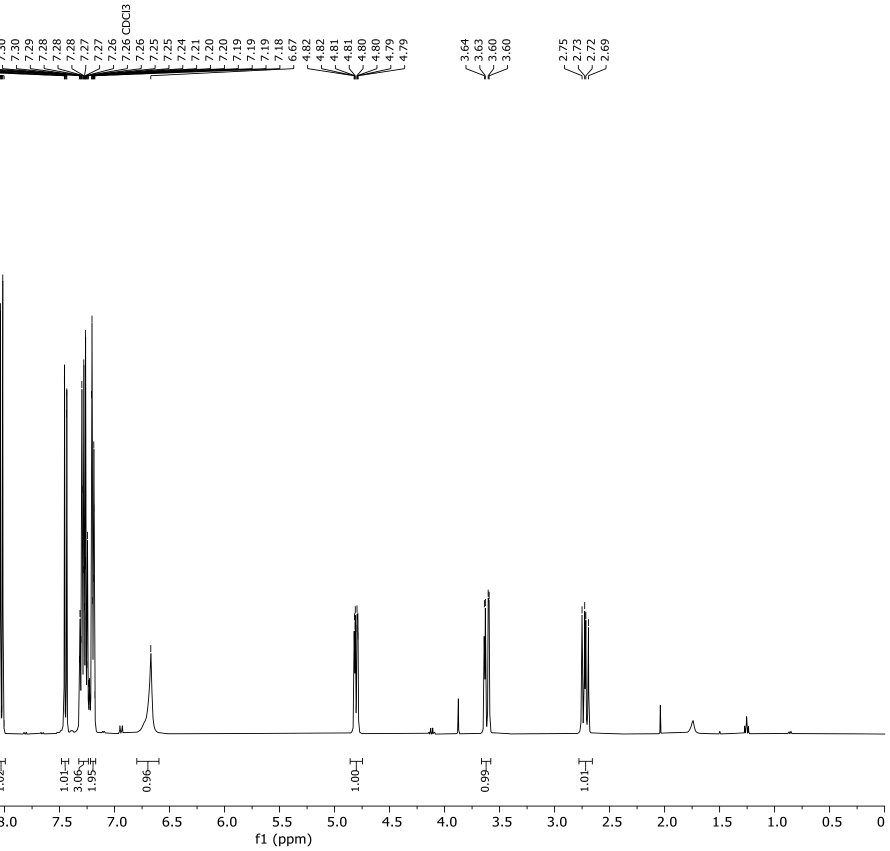

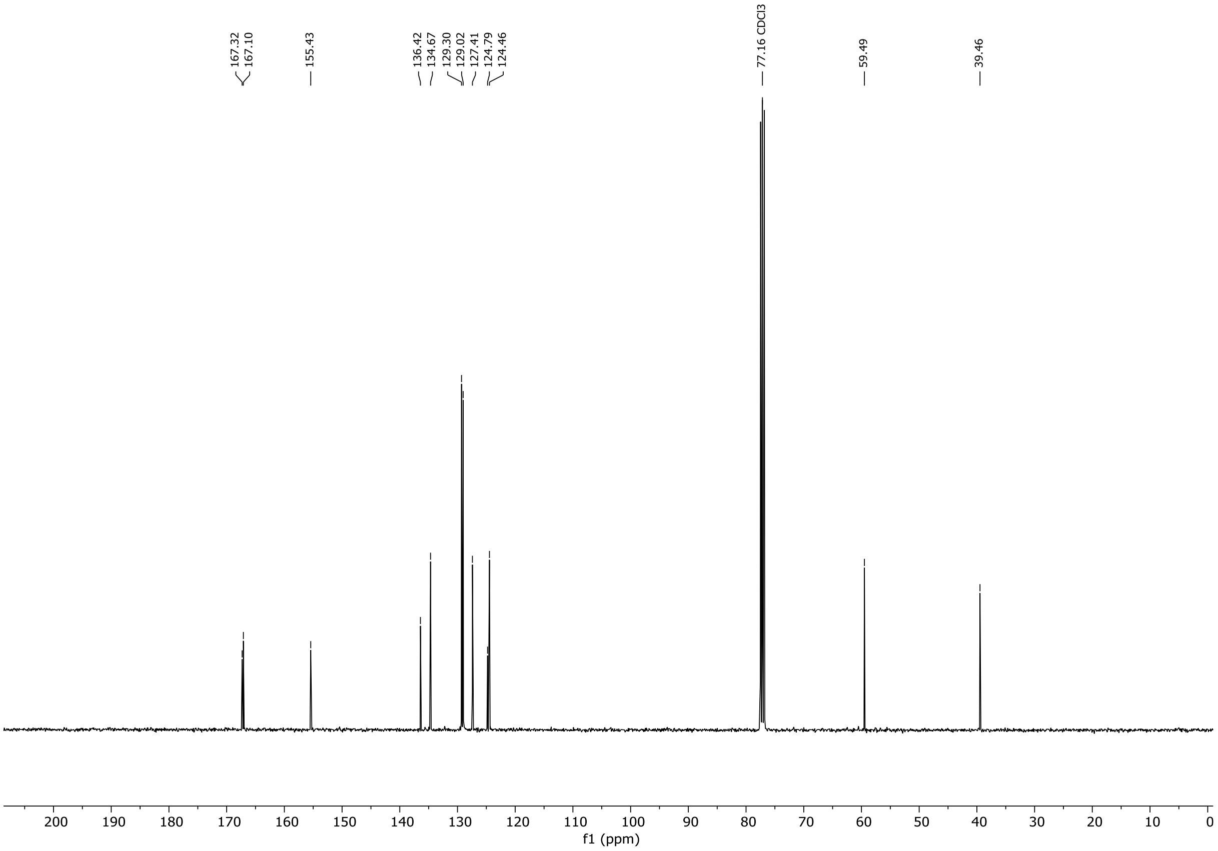

7-Benzyl-3-bromo-6,7-dihydro-5*H*-pyrrolo[3,4-*b*]pyridin-5-one (*rac*-**3o**)

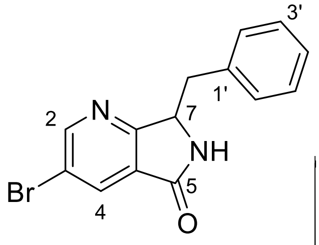

rac-**3o**

$C_{14}H_{11}BrN_2O$

M = 303.16 g mol^{-1}

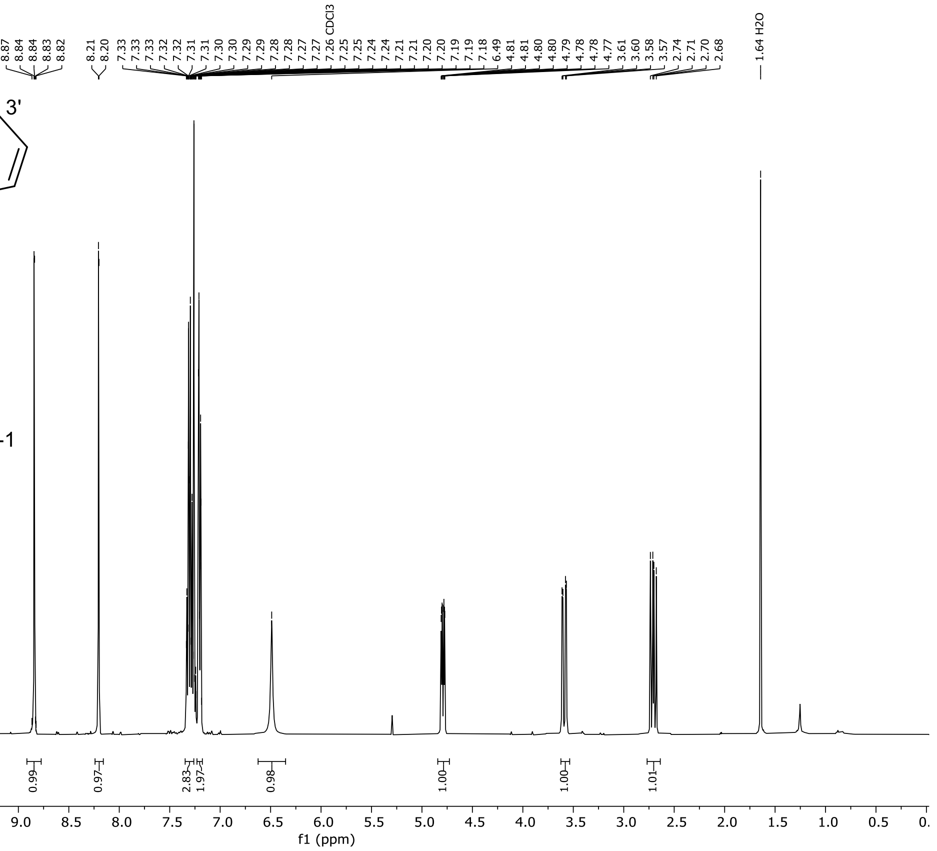

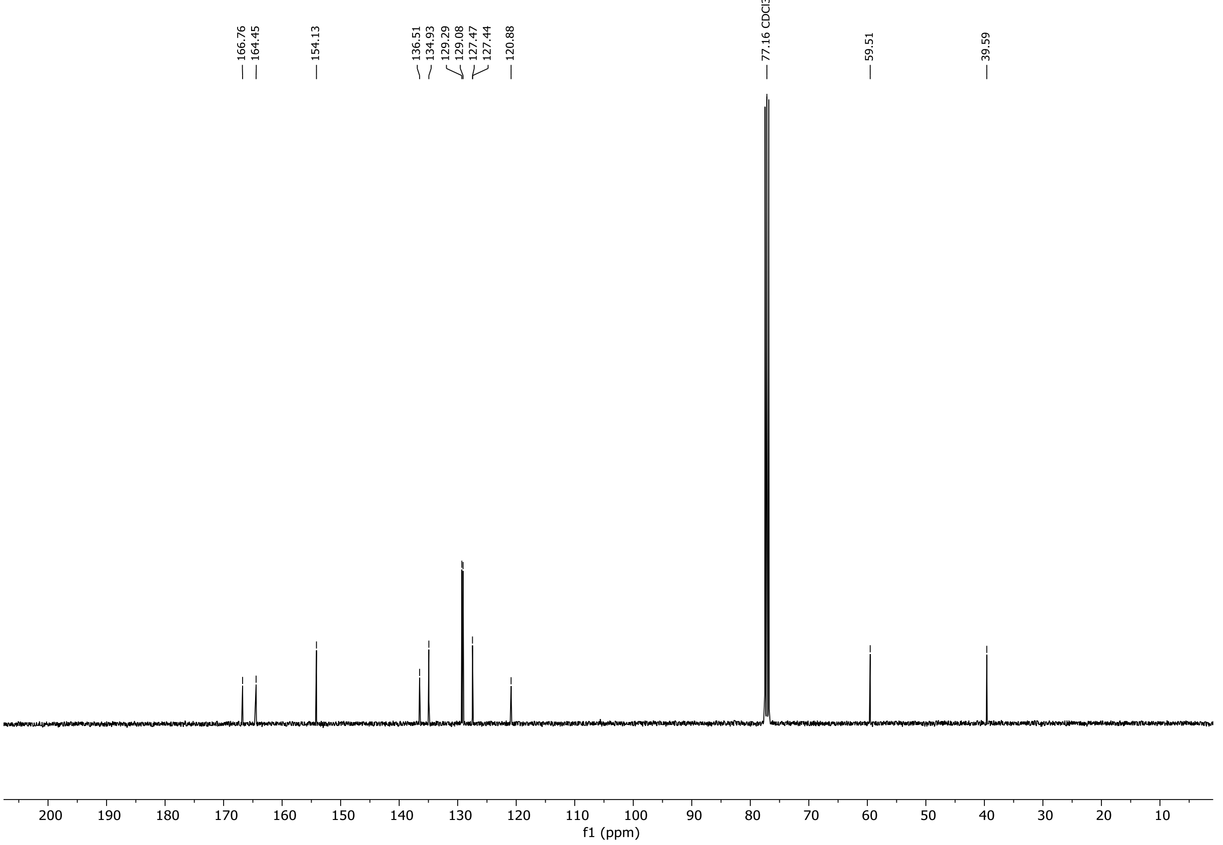

7-Benzyl-3-phenyl-6,7-dihydro-5*H*-pyrrolo[3,4-*b*]pyridin-5-one (*rac*-3q)

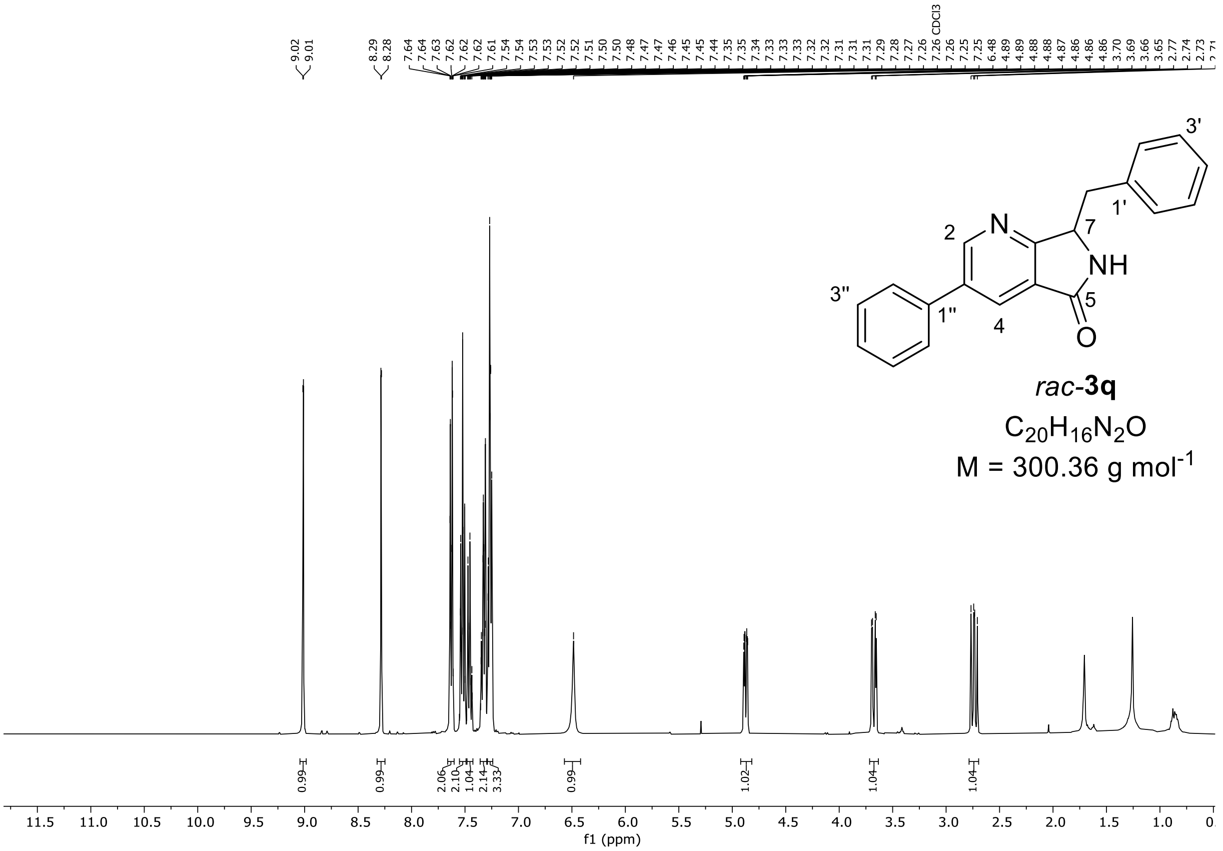

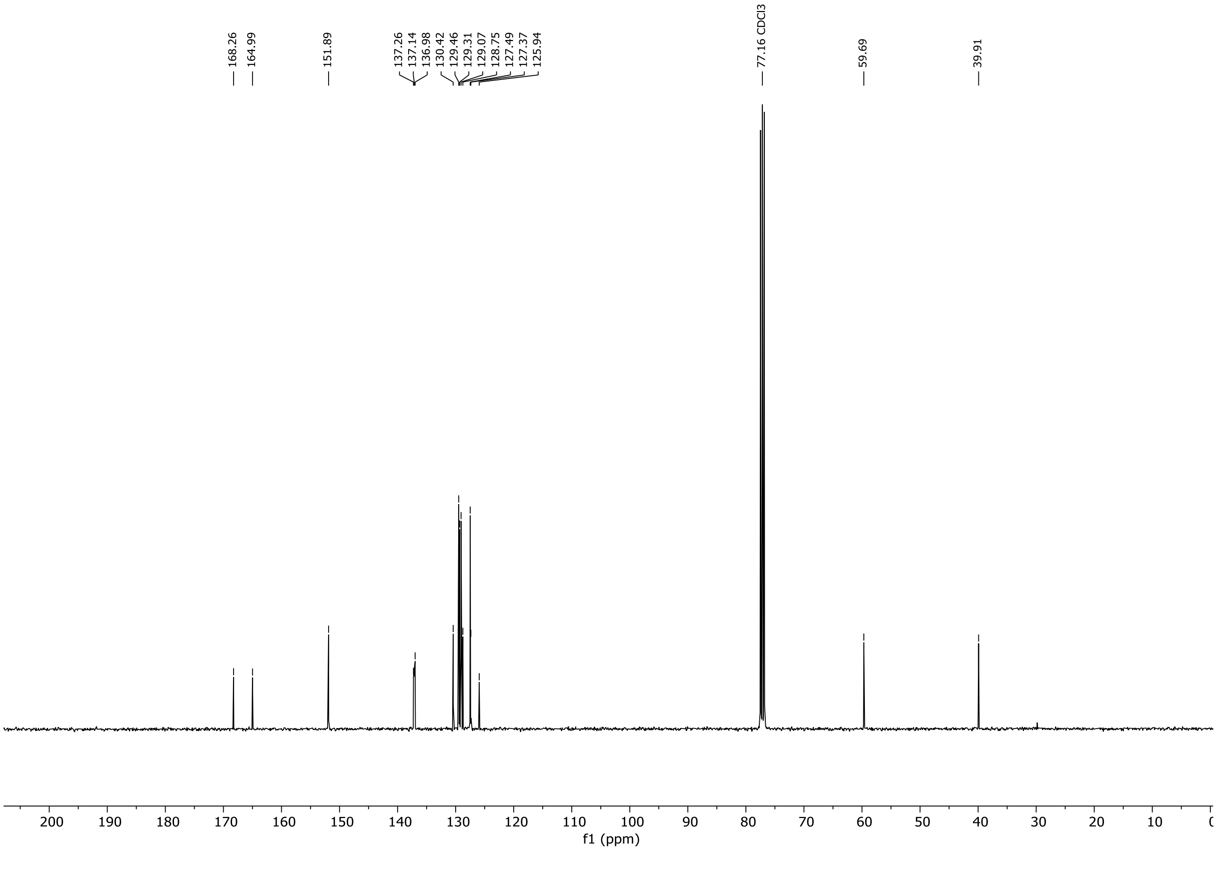

7-Isopropoxy-6,7-dihydro-5*H*-pyrrolo[3,4-*b*]pyridin-5-one (*rac*-3r)

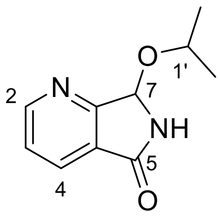

rac-**3r**
$C_{10}H_{12}N_2O_2$
M = 192.22 g mol^{-1}

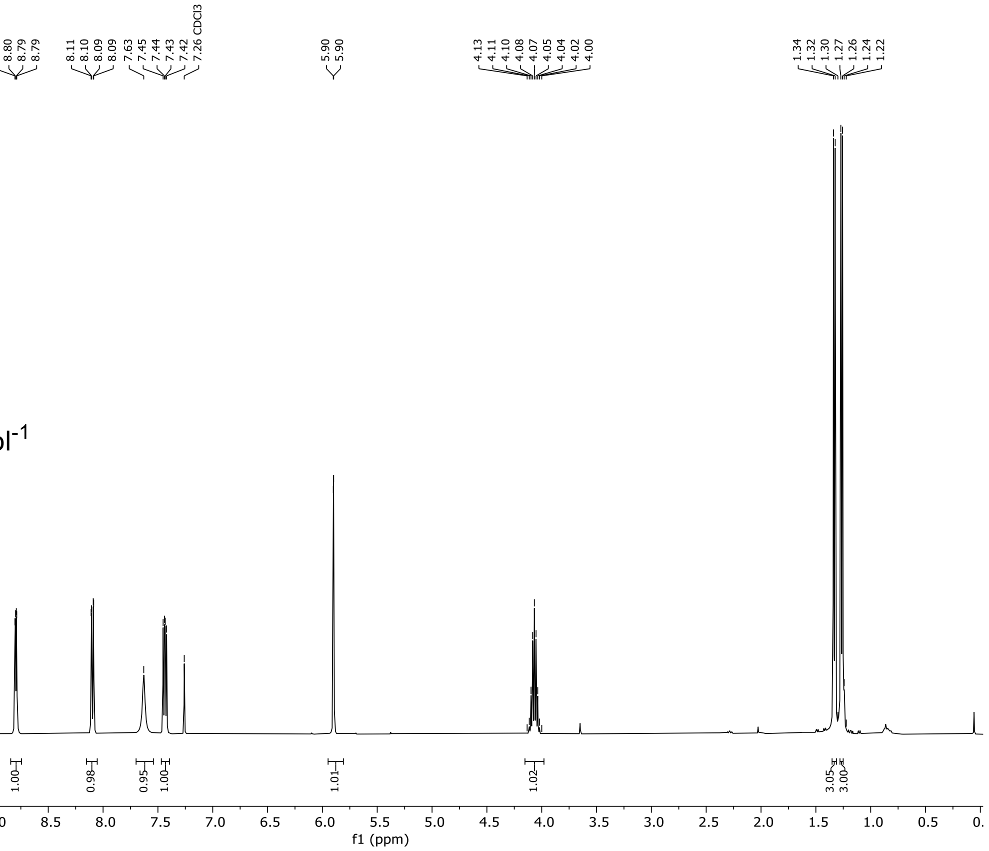

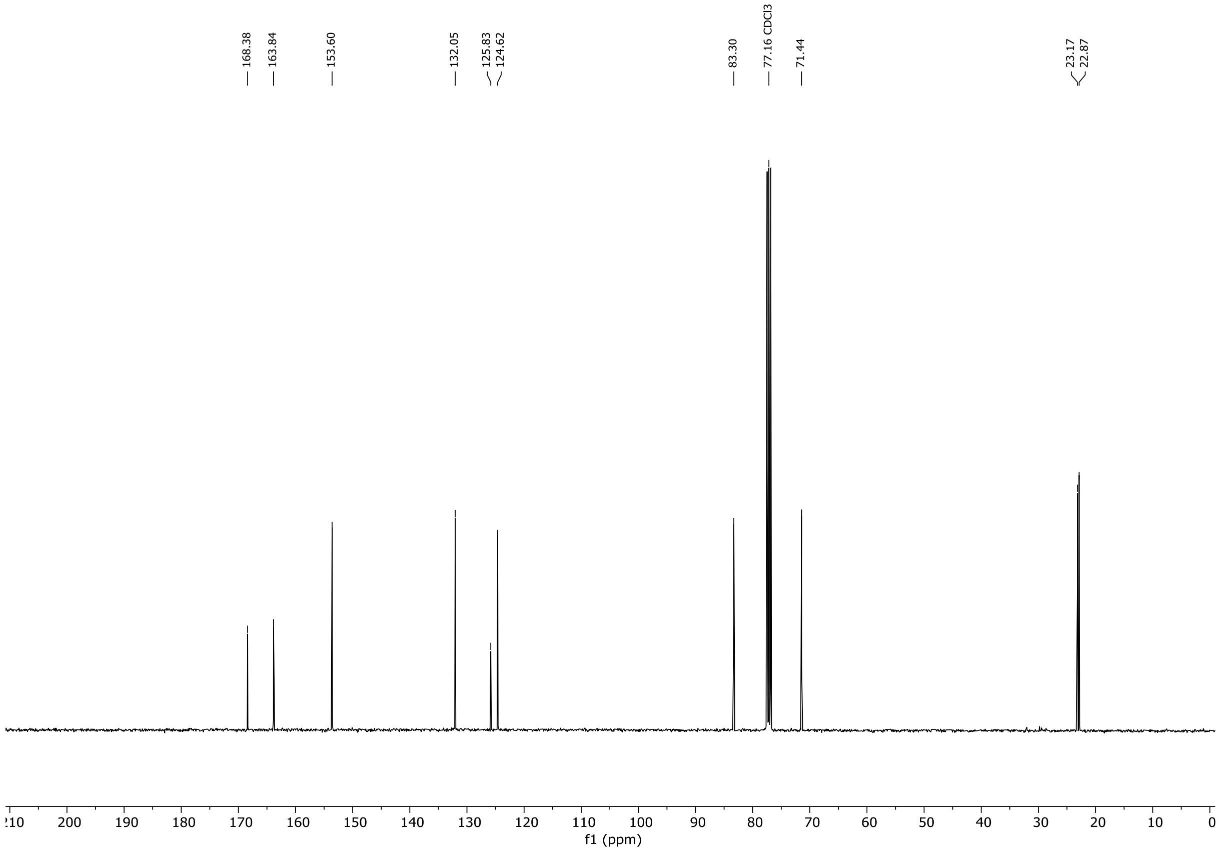

7-(Cyclopentyloxy)-6,7-dihydro-5*H*-pyrrolo[3,4-*b*]pyridin-5-one (*rac*-3s)

rac-**3s**
$C_{12}H_{14}N_2O_2$
M = 218.26 g mol^{-1}

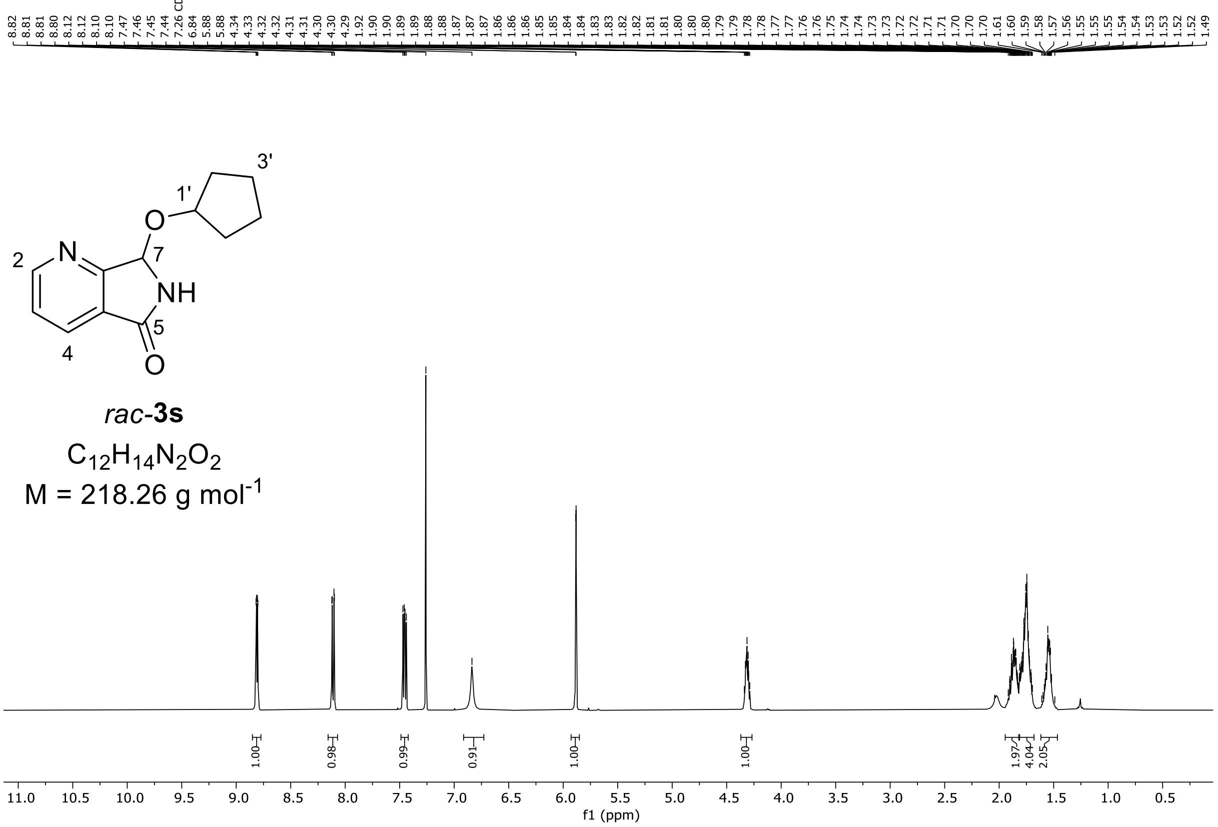

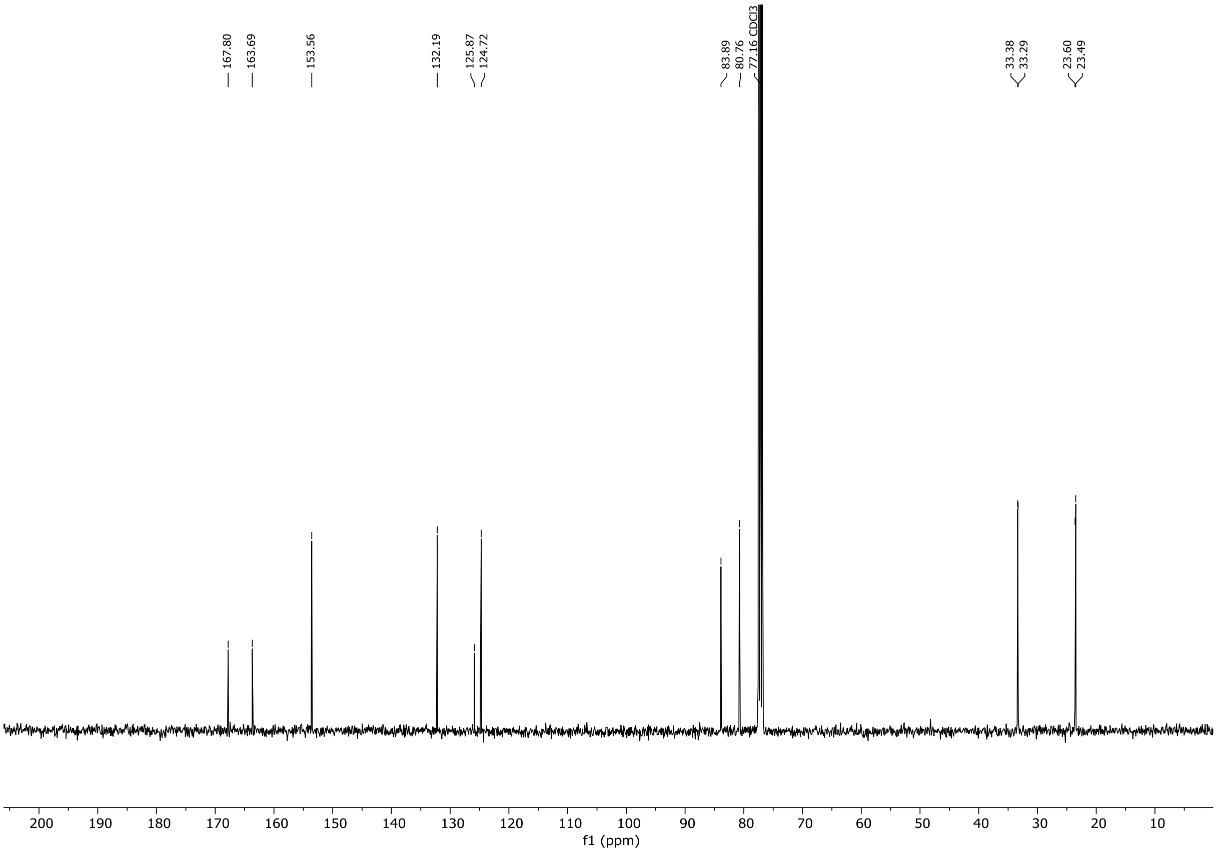

7-(*tert*-Butoxy)-6,7-dihydro-5*H*-pyrrolo[3,4-*b*]pyridin-5-one (*rac*-3t)

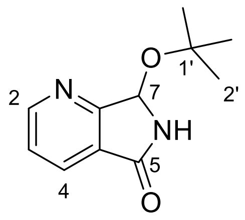

rac-**3t**

$C_{11}H_{14}N_2O_2$

M = 206.25 g mol^{-1}

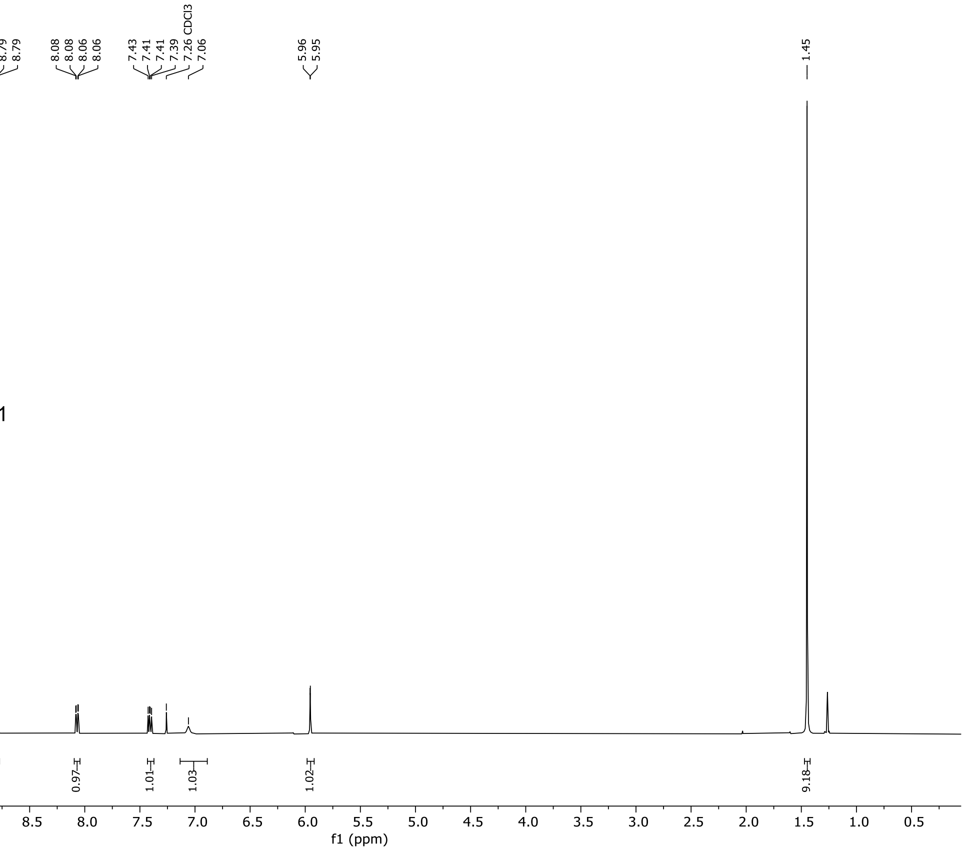

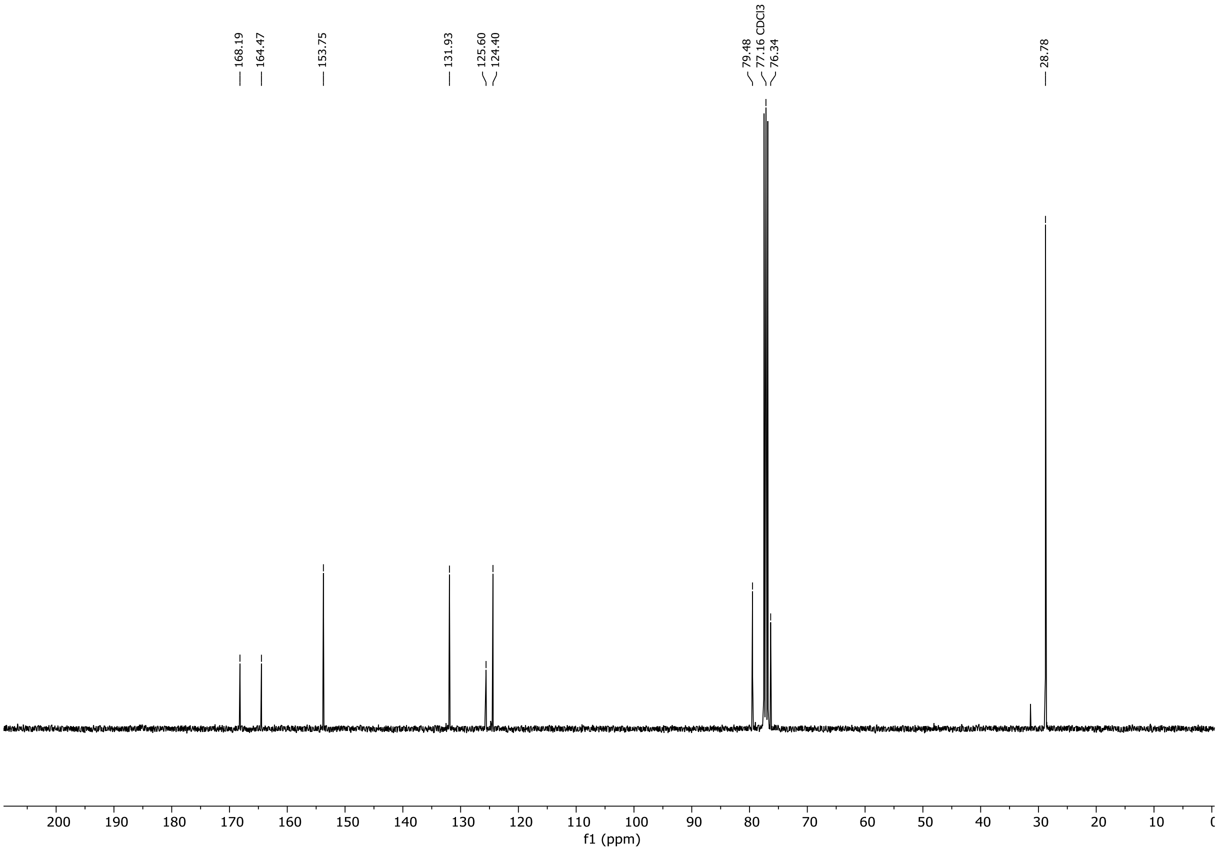

7-(3-Chloro-2,2-dimethylpropoxy)-6,7-dihydro-5*H*-pyrrolo[3,4-*b*]pyridin-5-one (*rac*-3u)

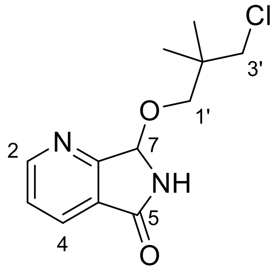

rac-**3u**

$C_{12}H_{15}ClN_2O_2$

M = 254.71 g mol^{-1}

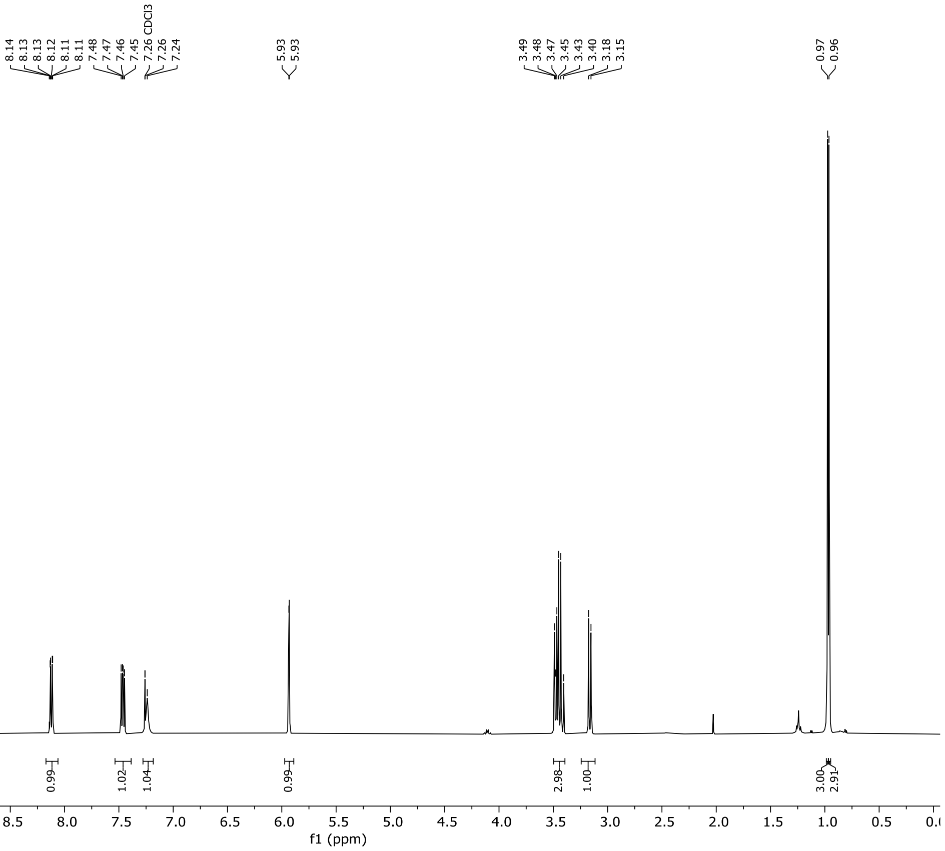

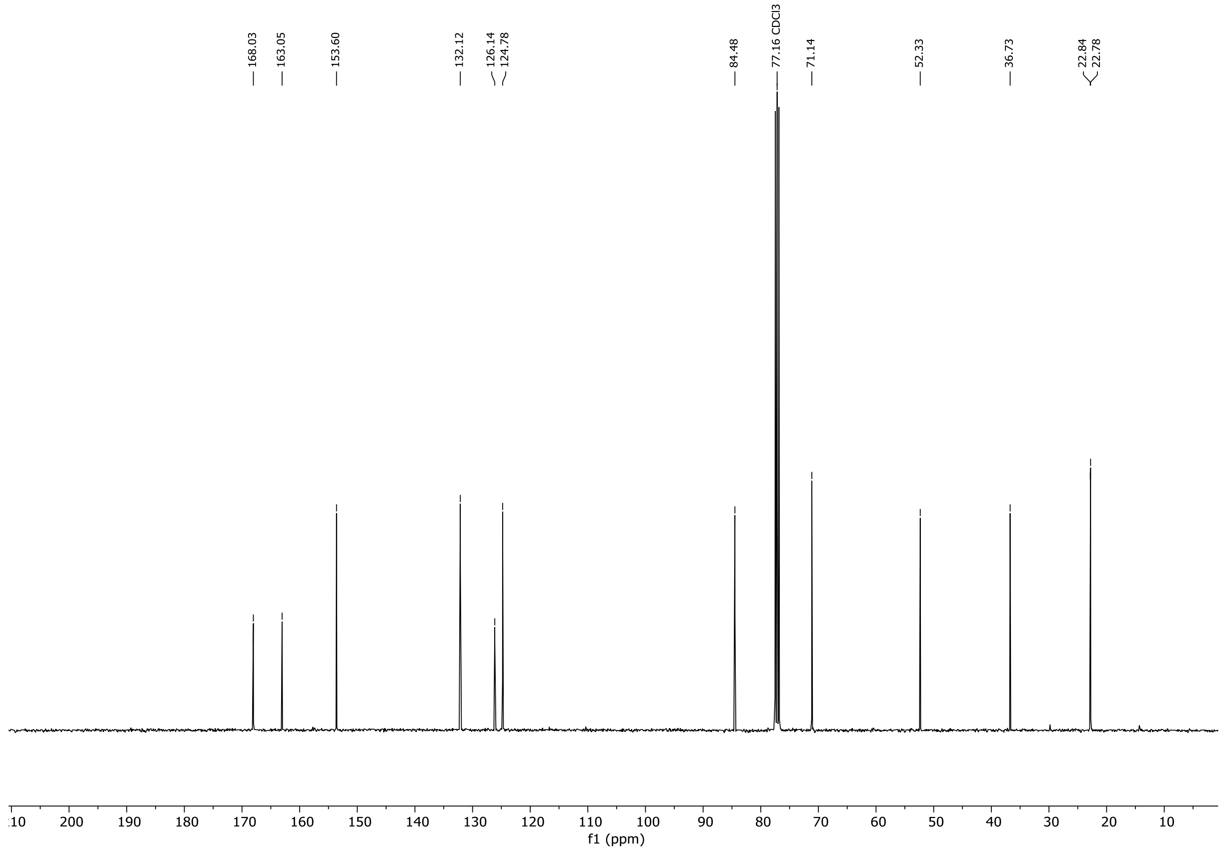

7-((*tert*-Butyldimethylsilyl)oxy)-6,7-dihydro-5*H*-pyrrolo[3,4-*b*]pyridin-5-one (*rac*-3v)

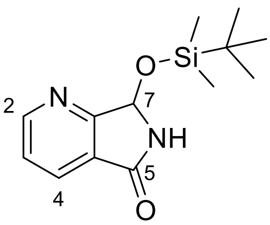

rac-**3v**

$C_{13}H_{20}N_2O_2Si$

M = 264.40 g mol^{-1}

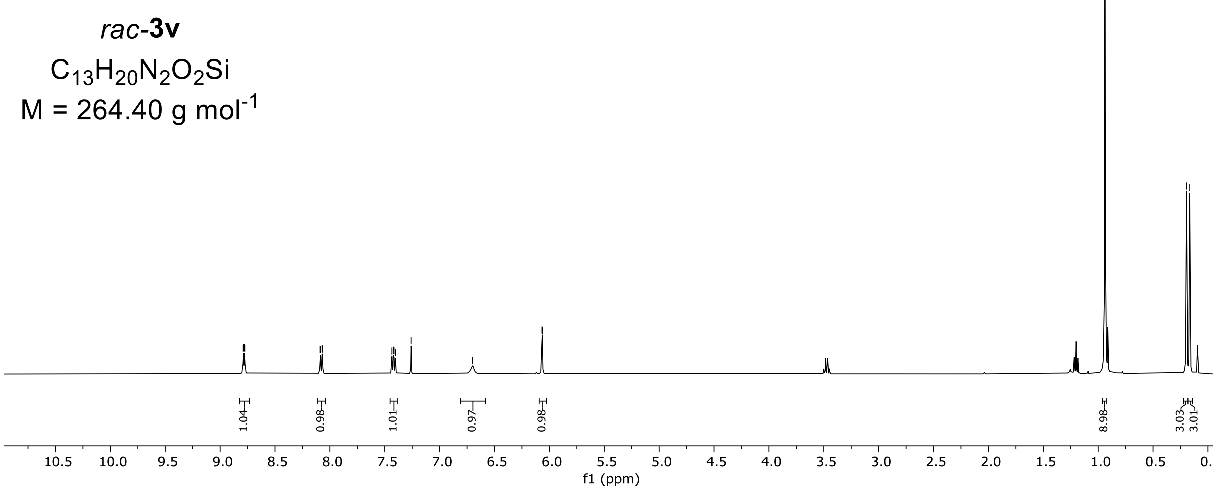

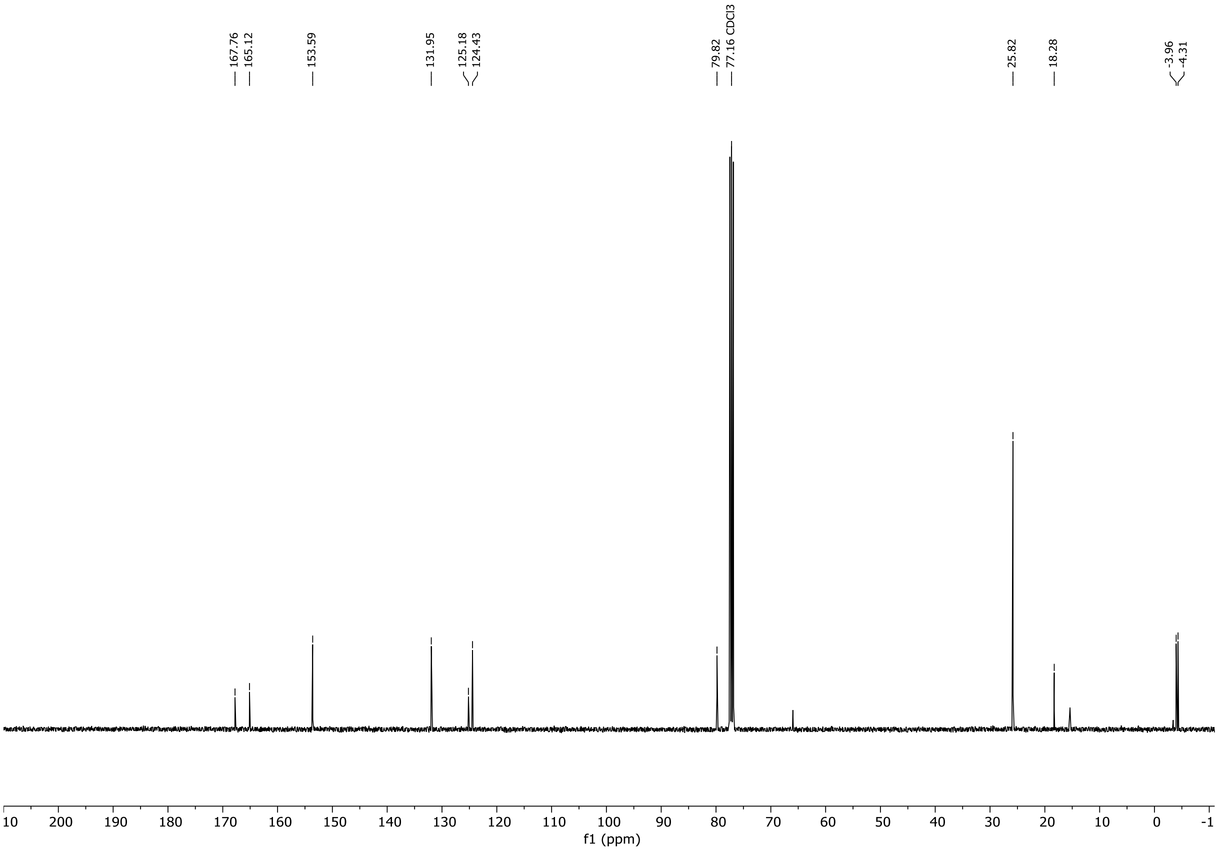

7-((*S*)-2-Methylbutoxy)-6,7-dihydro-5*H*-pyrrolo[3,4-*b*]pyridin-5-one (*3S/3R*-3w)

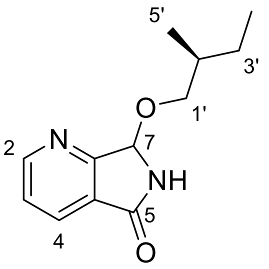

3S/3R-**3w**

$C_{12}H_{16}N_2O_2$

M = 220.27g mol^{-1}

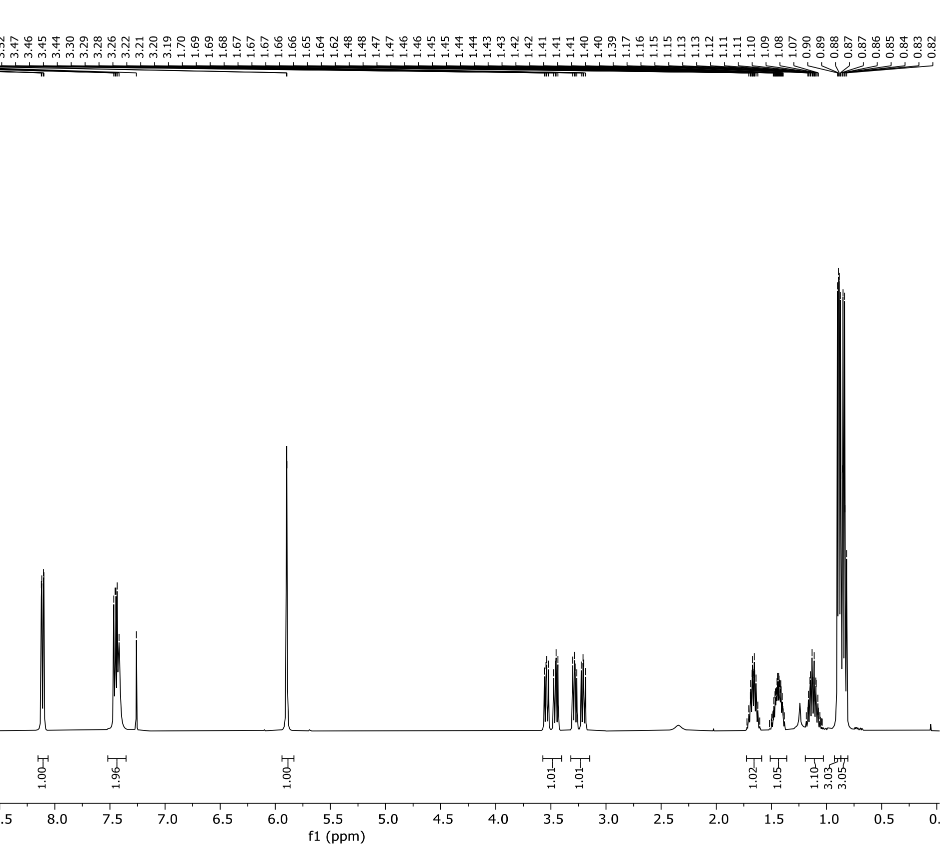

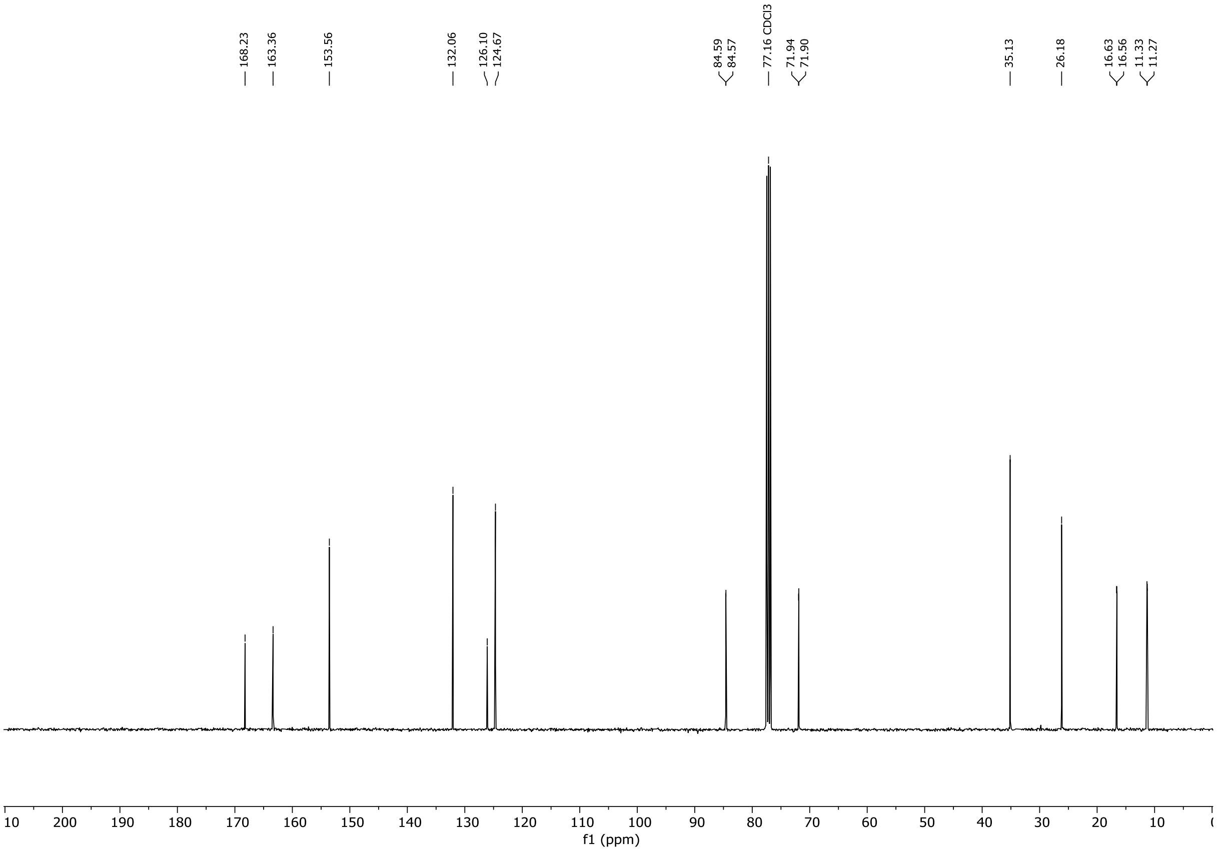

2-(4-Methoxybenzyl)-2,3-dihydro-1*H*-pyrrolo[3,4-*c*]pyridin-1-one (SI-17)

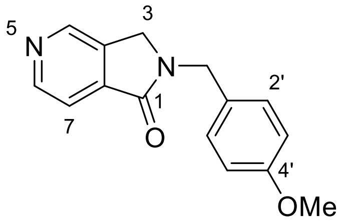

SI-17

$C_{15}H_{14}N_2O_2$

M = 254.29 g mol^{-1}

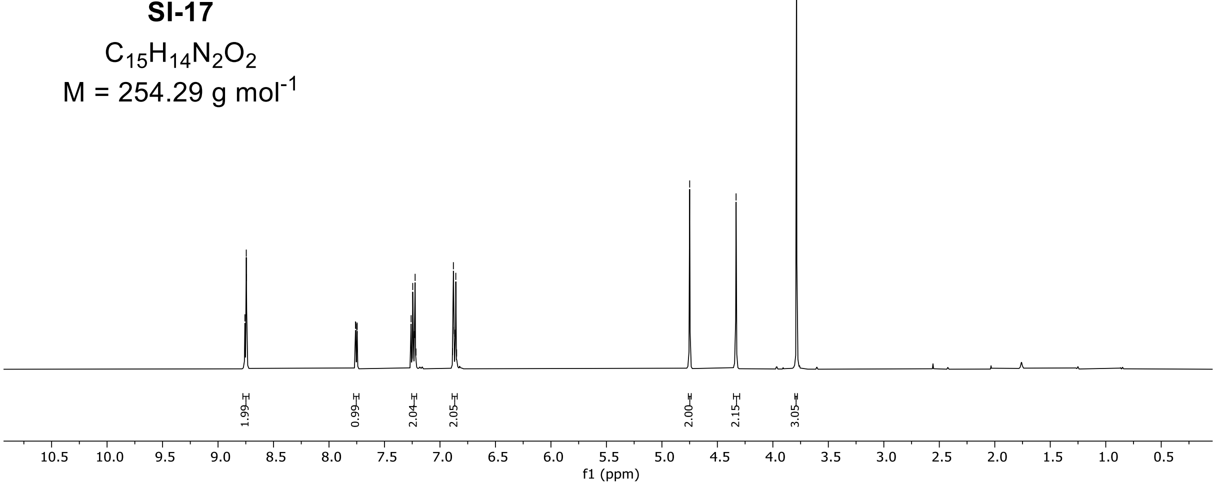

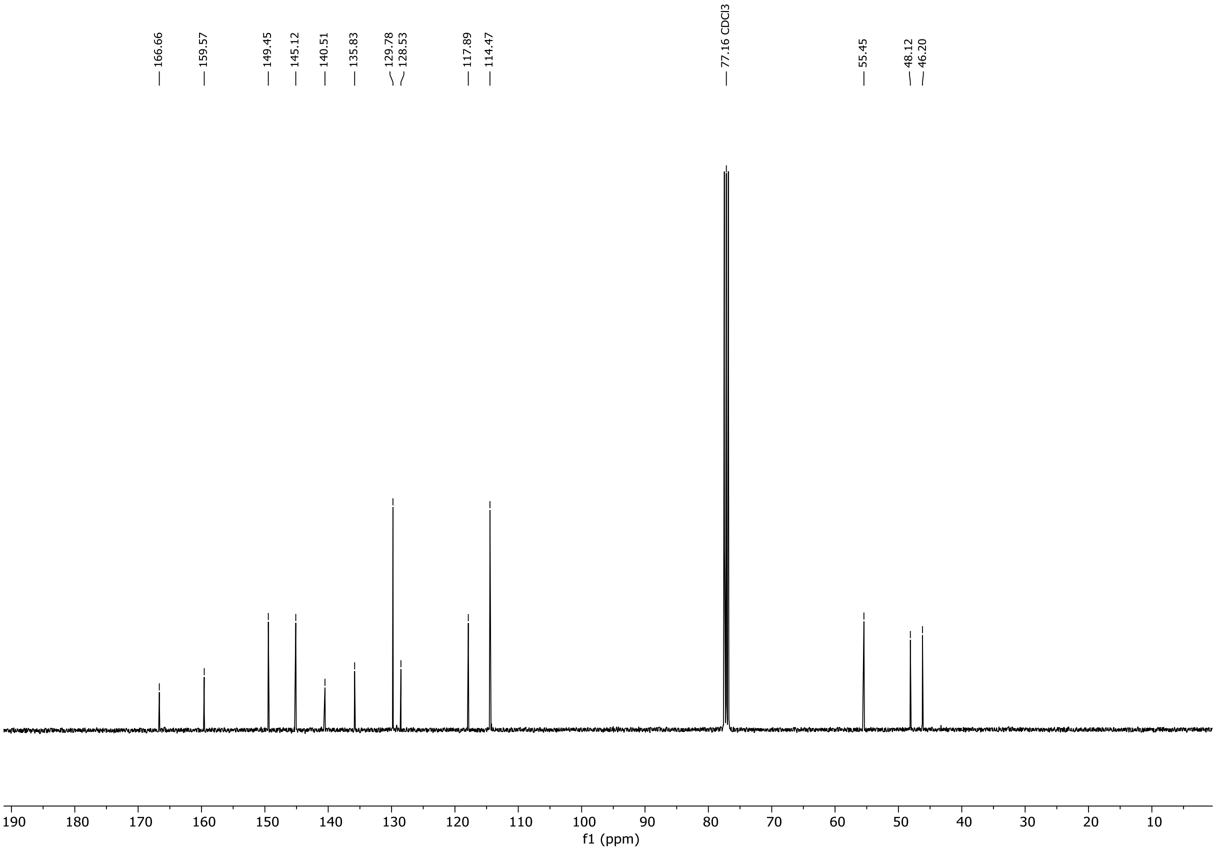

3-Benzyl-2,3-dihydro-1*H*-pyrrolo[3,4-*c*]pyridin-1-one (*rac*-**12a**)

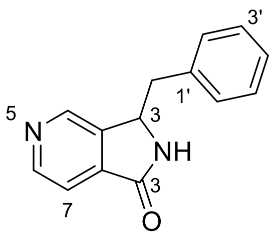

rac-**12a**

$C_{14}H_{12}N_2O$

M = 224.26 g mol^{-1}

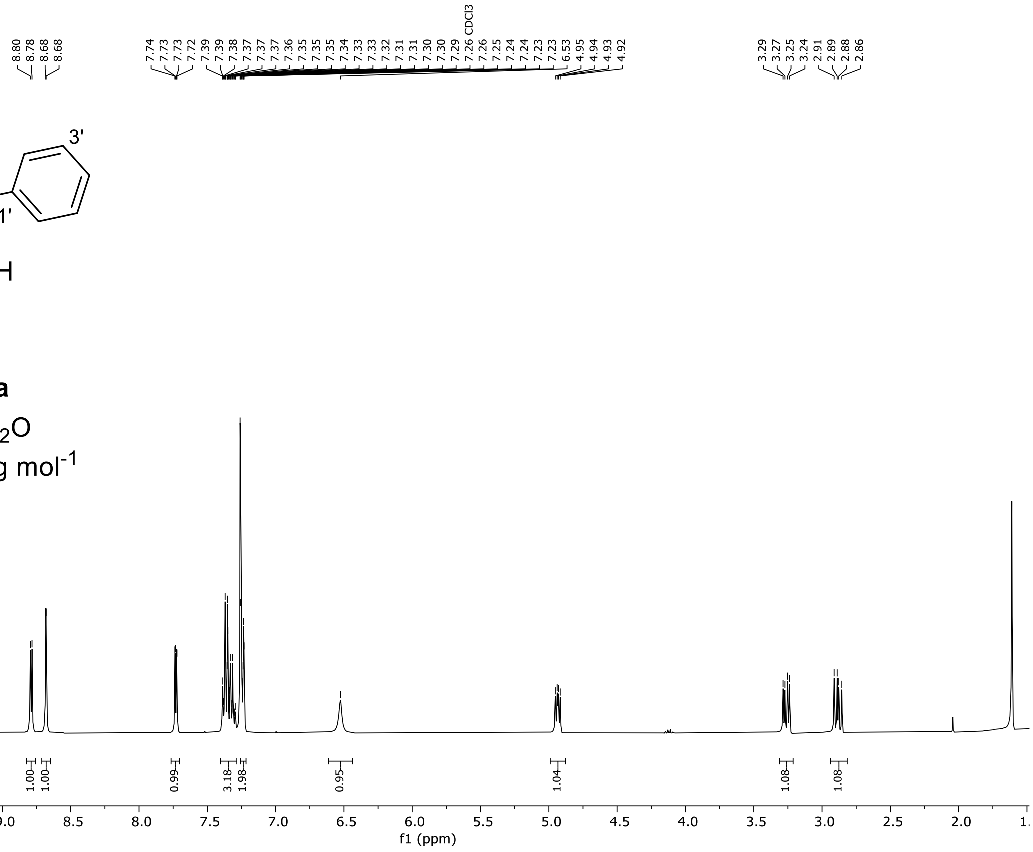

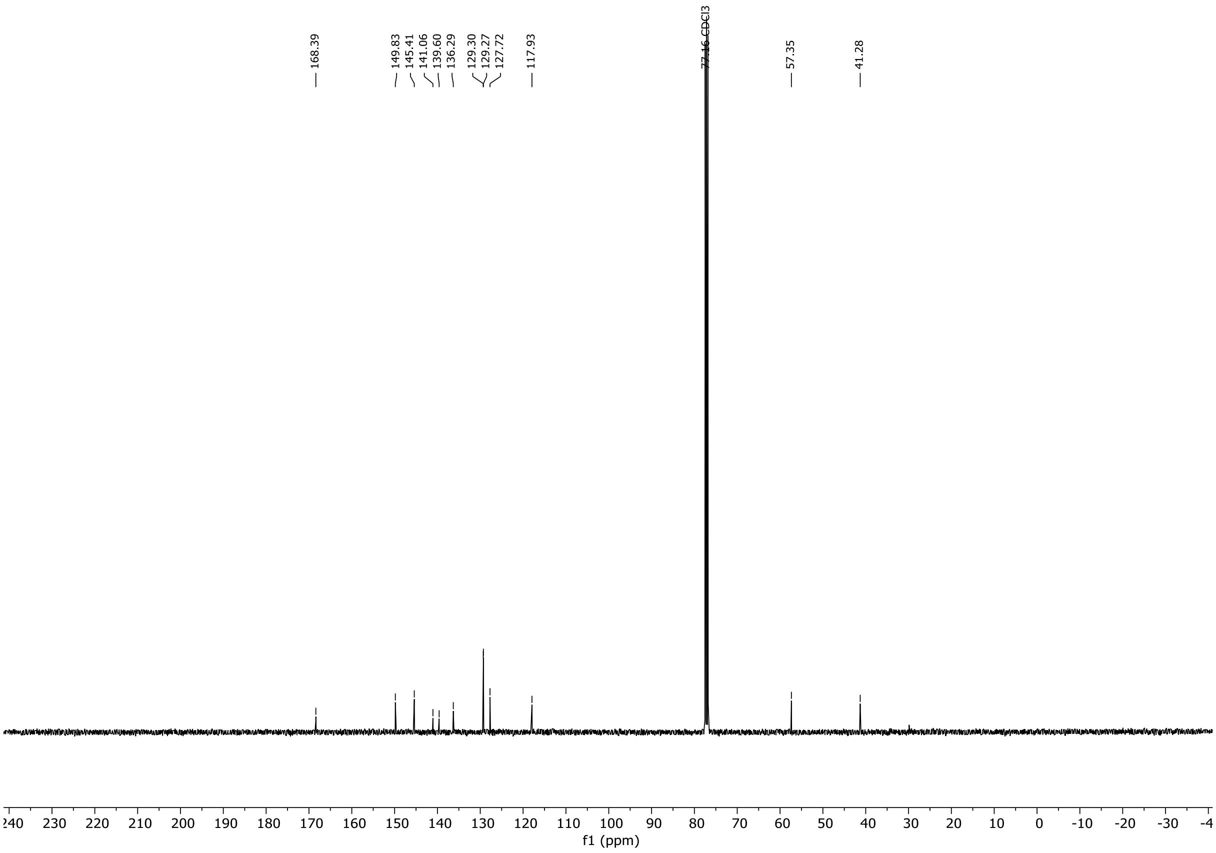

3-(Methoxycarbonyl)-4-methylpyridine 1-oxide (SI-18)

SI-18

$C_8H_9NO_3$

M = 167.16 g mol^{-1}

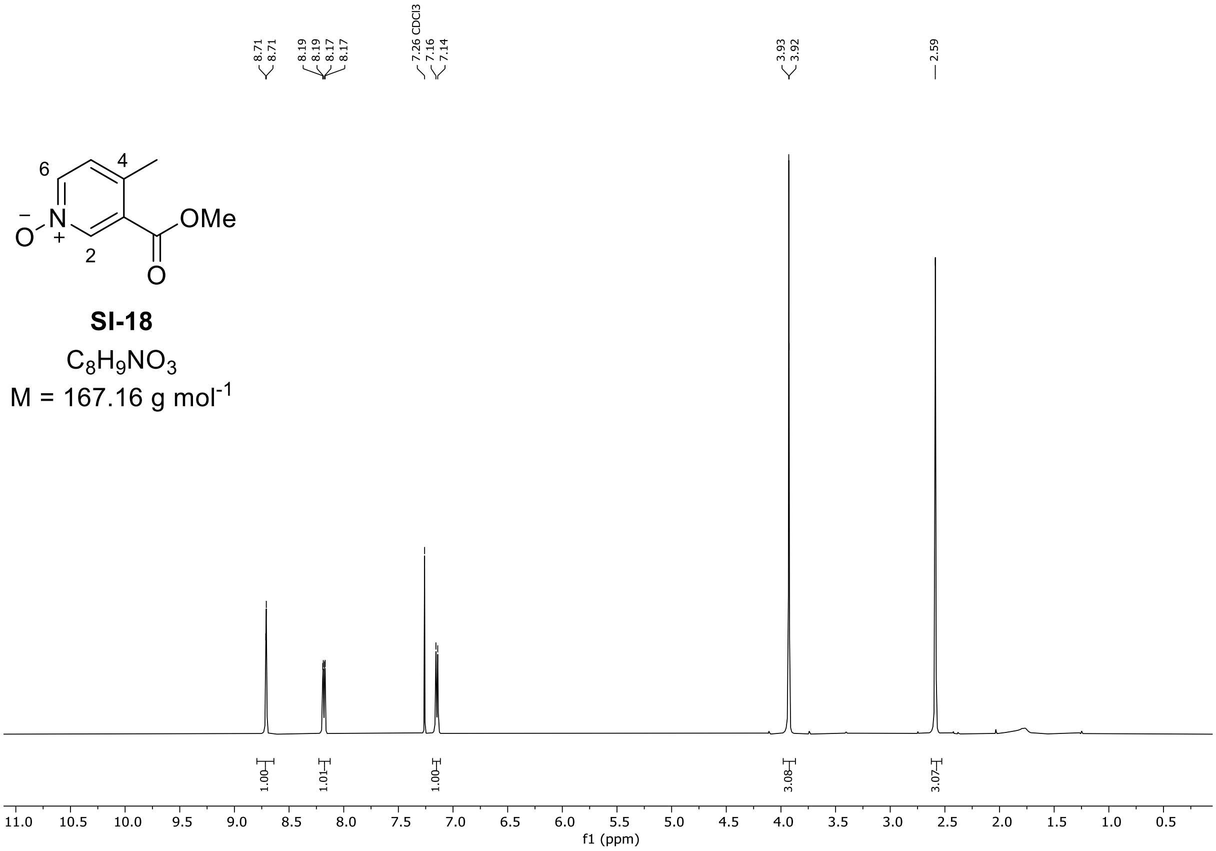

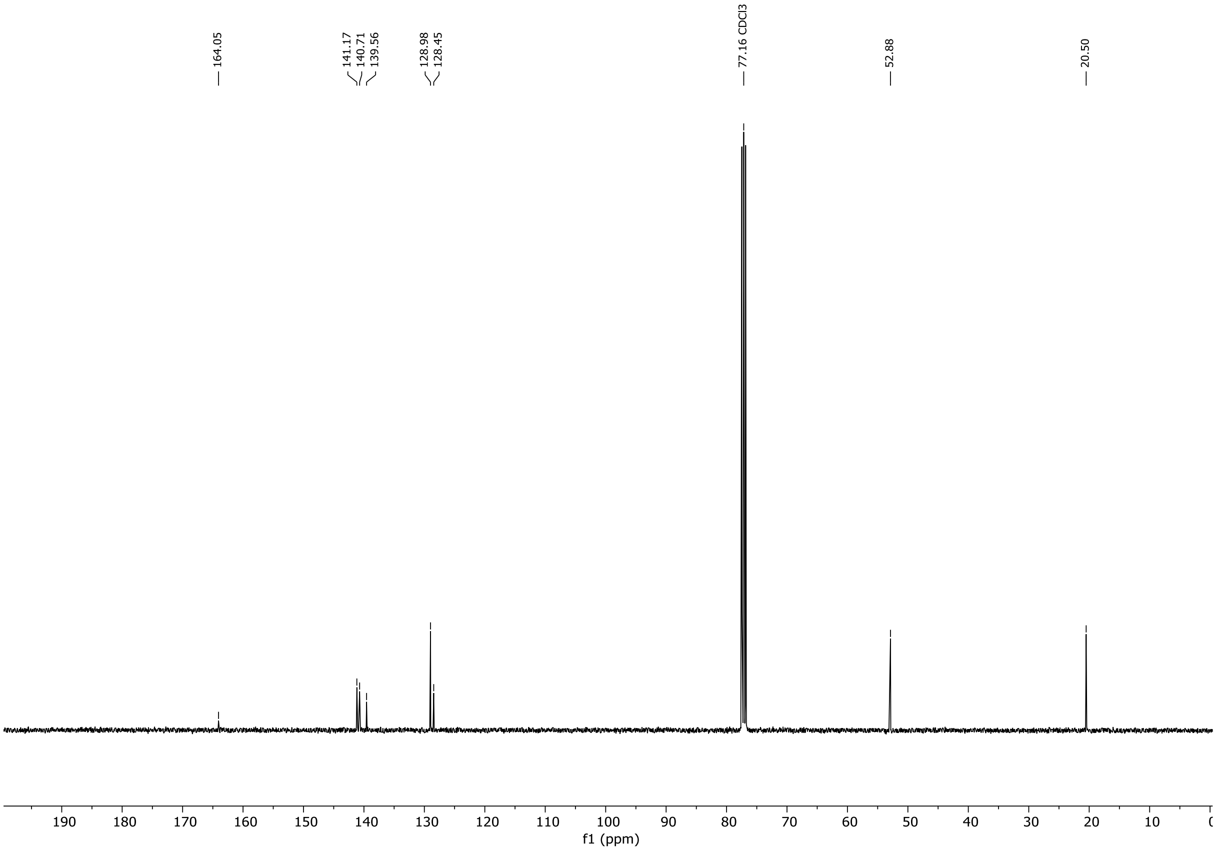

Methyl 4-(chloromethyl)nicotinate (SI-19)

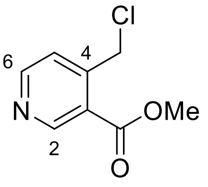

SI-19

$C_8H_8ClNO_2$

M = 185.61 g mol^{-1}

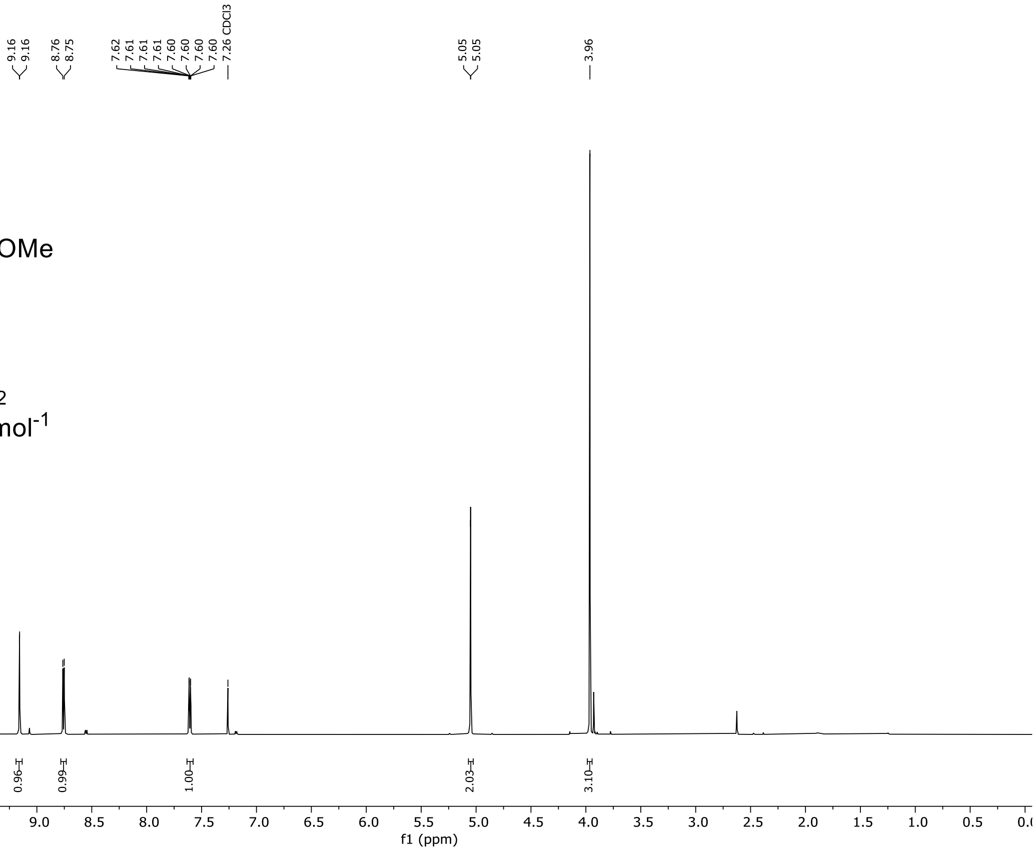

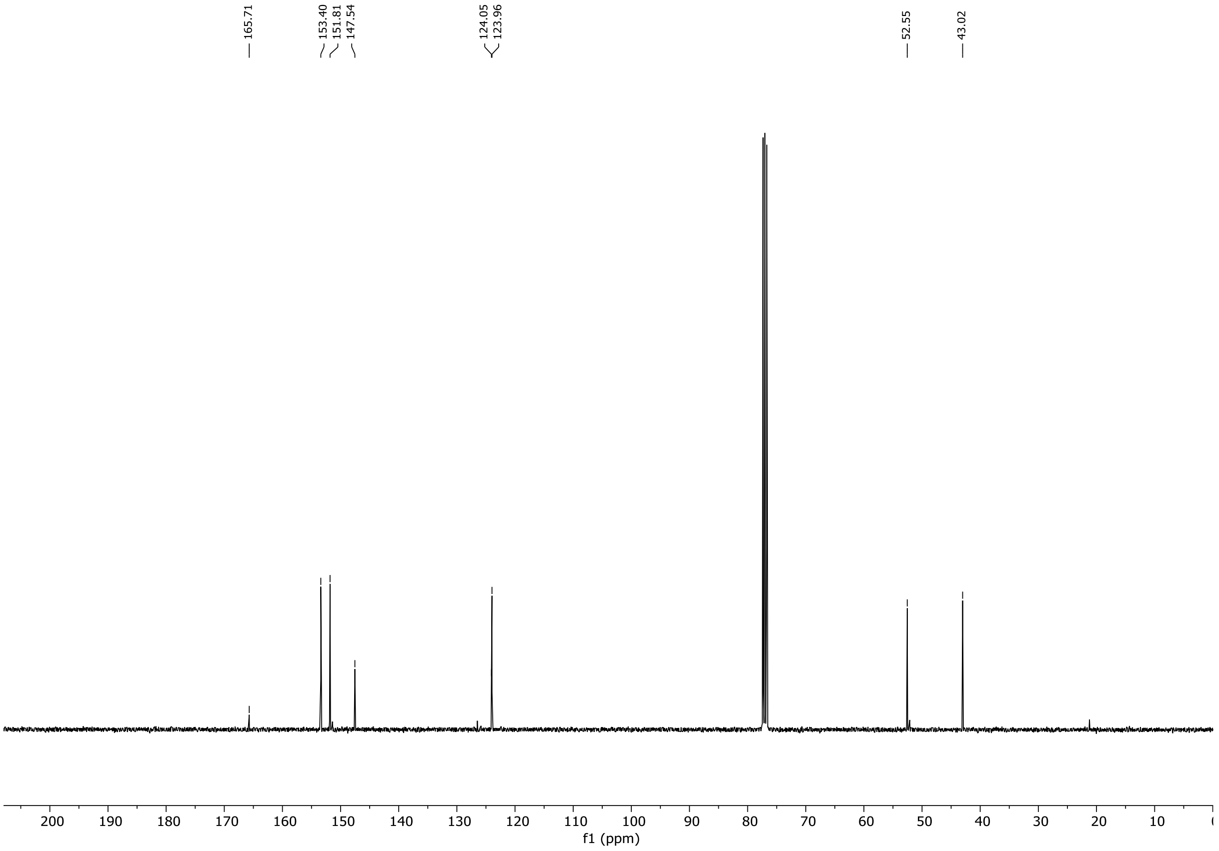

2-(4-Methoxybenzyl)-1,2-dihydro-3*H*-pyrrolo[3,4-*c*]pyridin-3-one (SI-20)

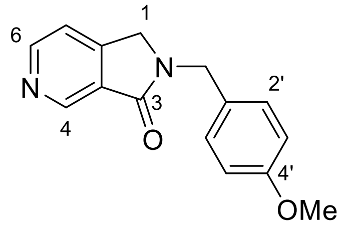

SI-20

$C_{15}H_{14}N_2O_2$

M = 254.29 g mol^{-1}

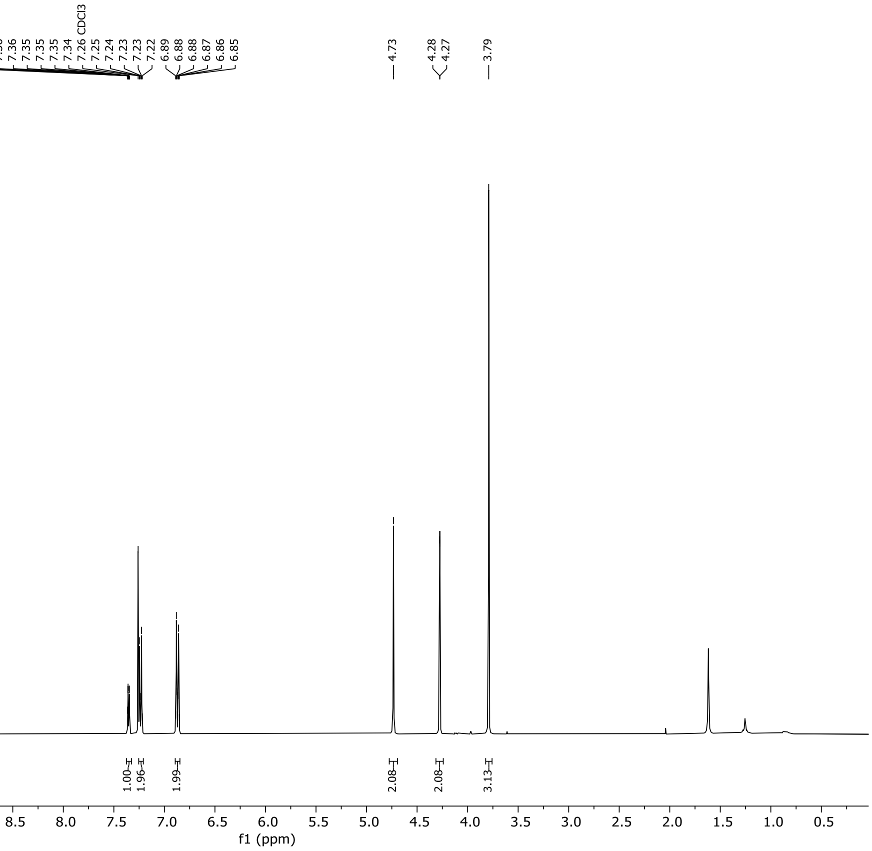

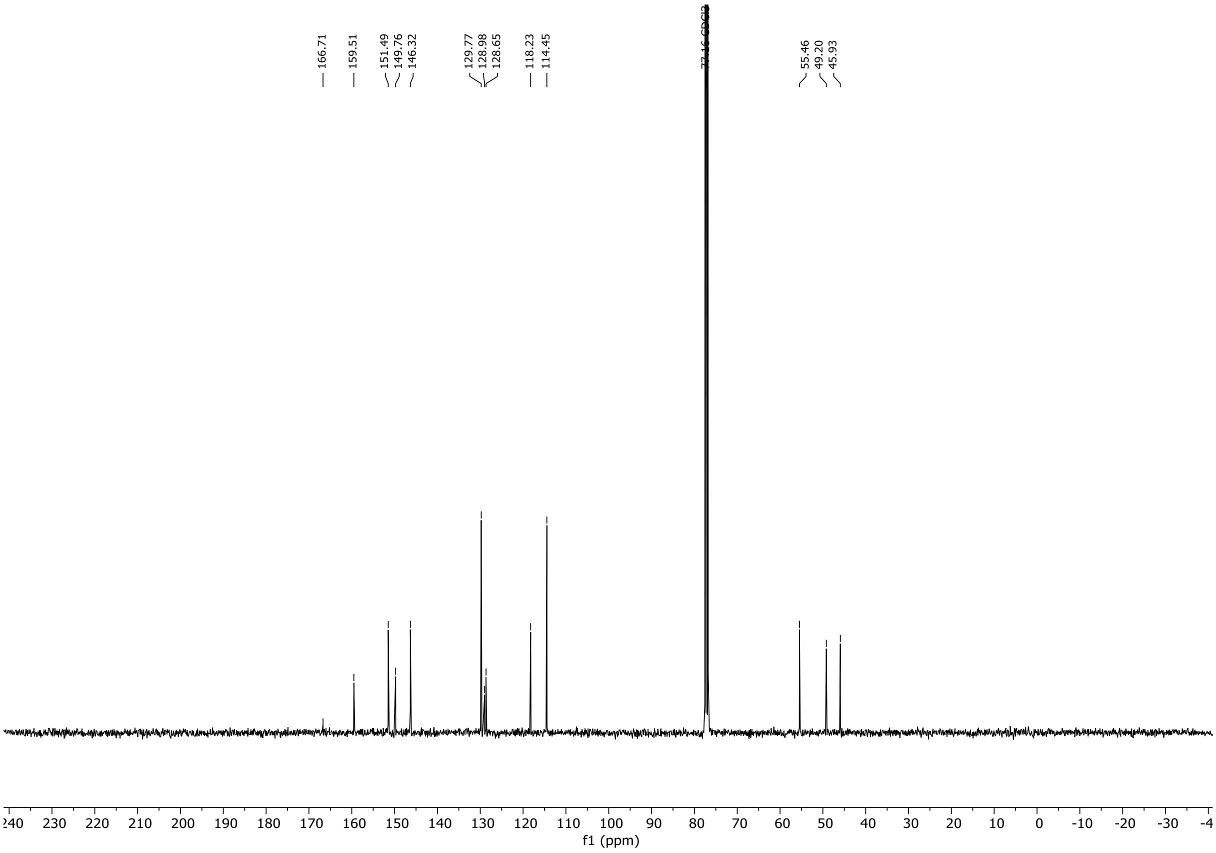

1-Benzyl-1,2-dihydro-3*H*-pyrrolo[3,4-*c*]pyridin-3-one (*rac*-12b)

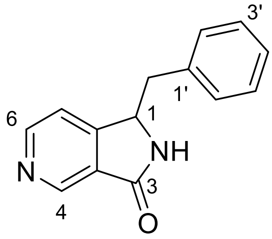

rac-**12b**

$C_{14}H_{12}N_2O$

M = 224.26 g mol^{-1}

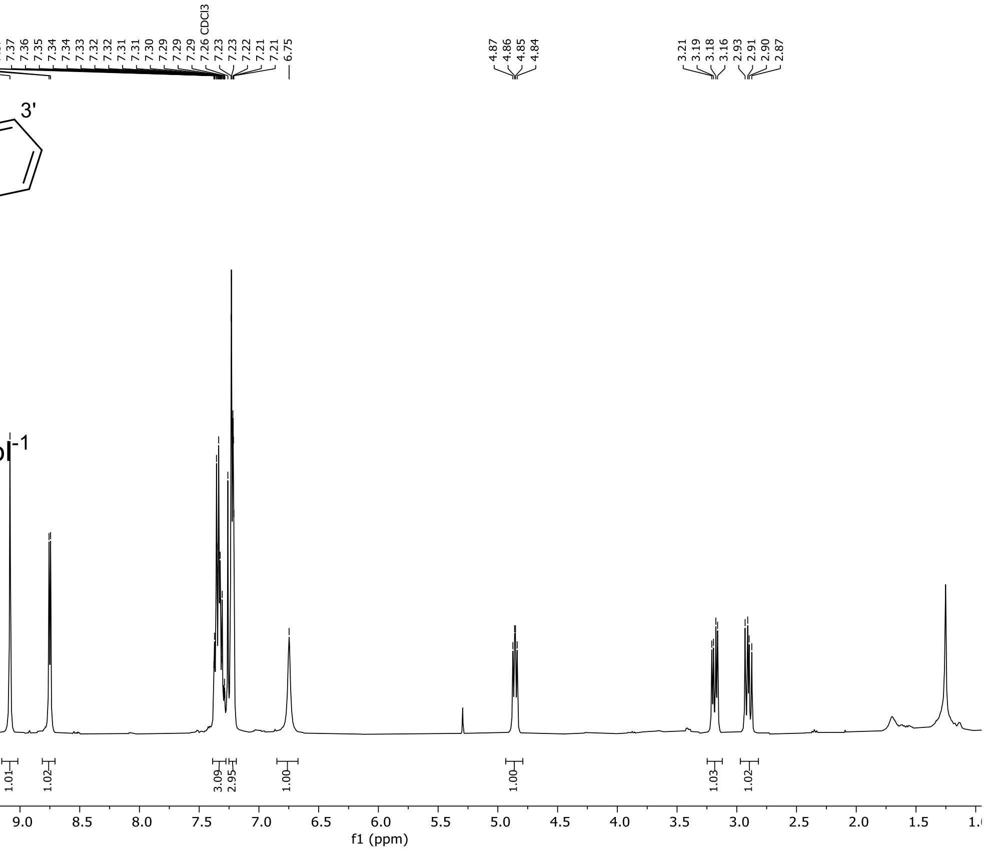

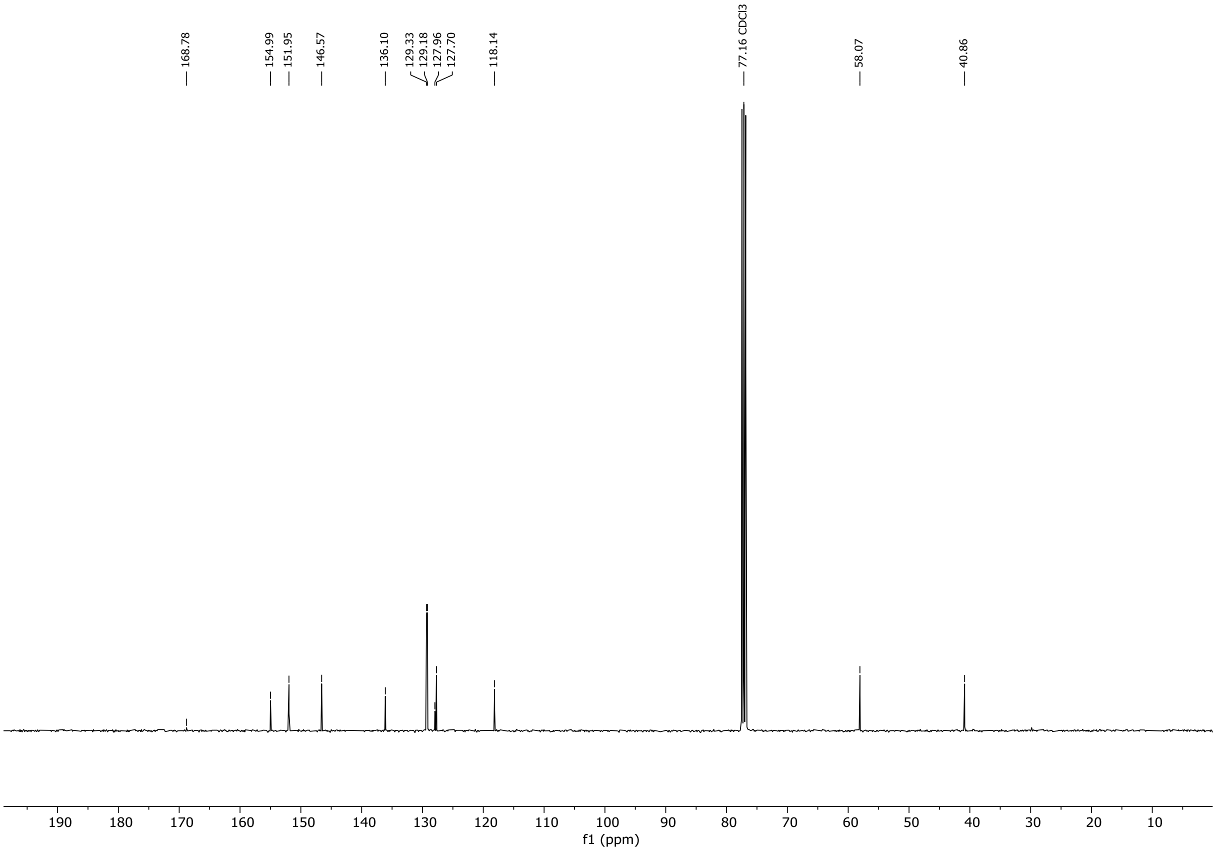

6-(4-Methoxybenzyl)-5,6-dihydro-7*H*-pyrrolo[3,4-*b*]pyridin-7-one (SI-21)

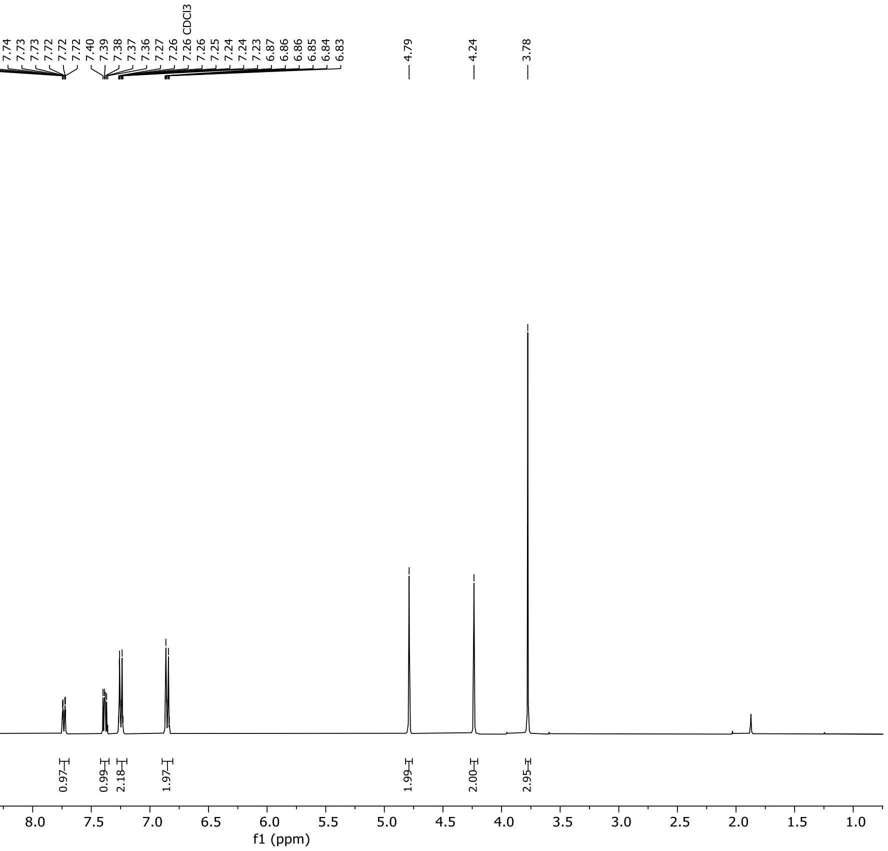

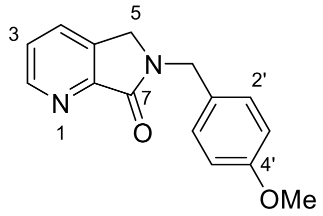

SI-21

$C_{15}H_{14}N_2O_2$

M = 254.29 g mol^{-1}

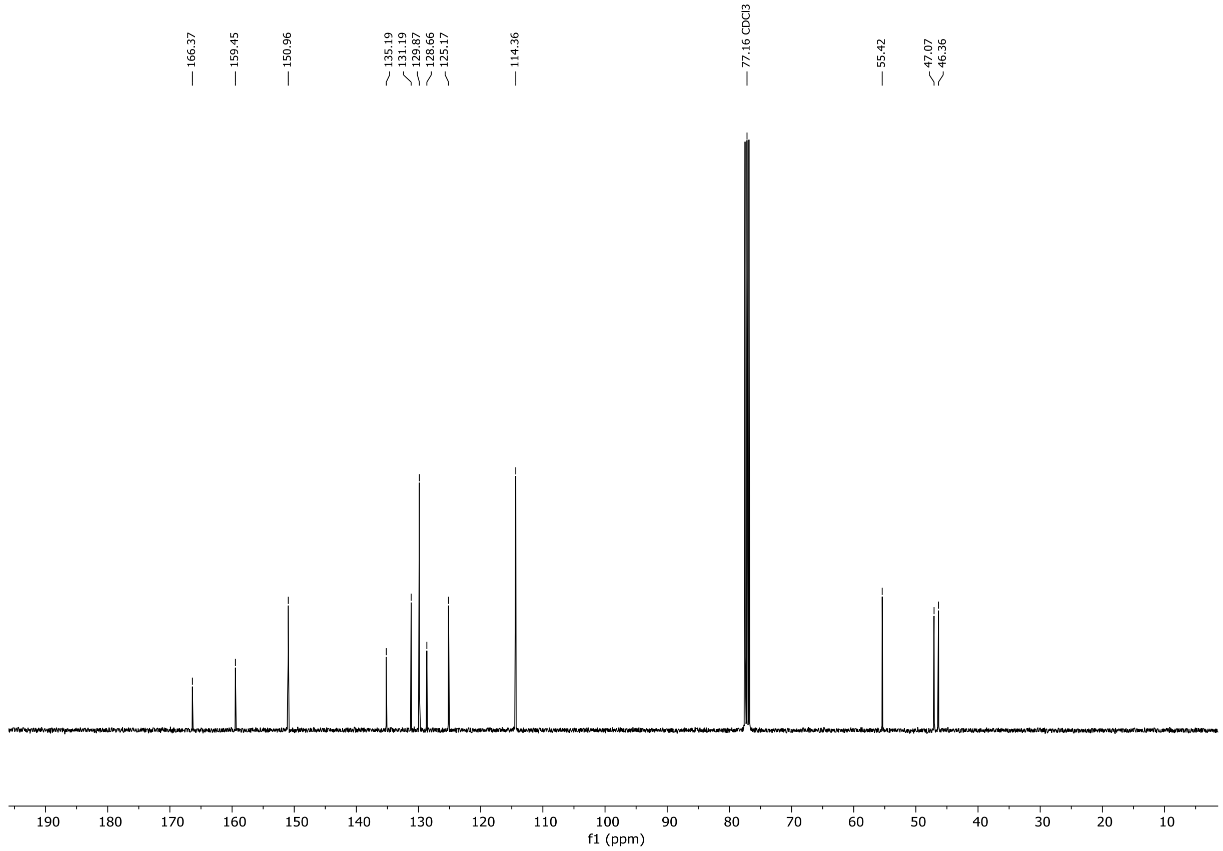

5-Benzyl-5,6-dihydro-7*H*-pyrrolo[3,4-*b*]pyridin-7-one (*rac*-12c)

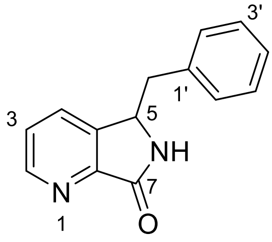

rac-**12c**

$C_{14}H_{12}N_2O$

M = 224.26 g mol^{-1}

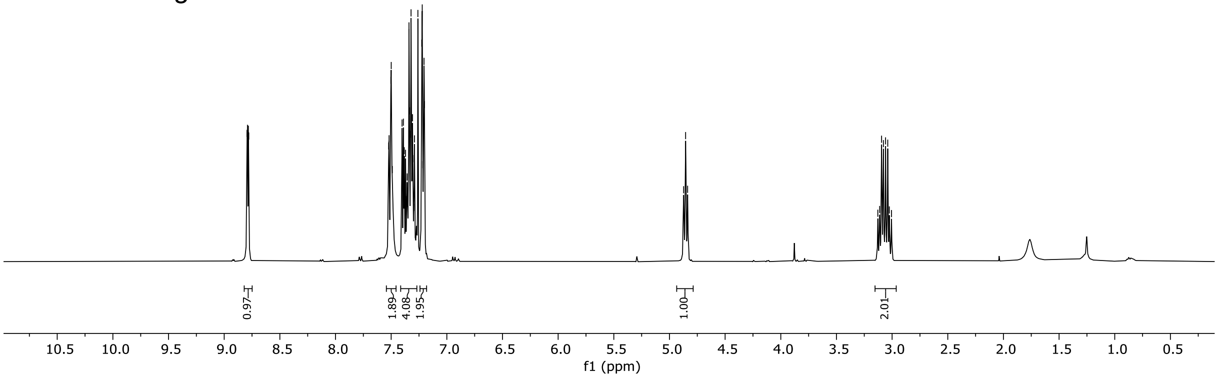

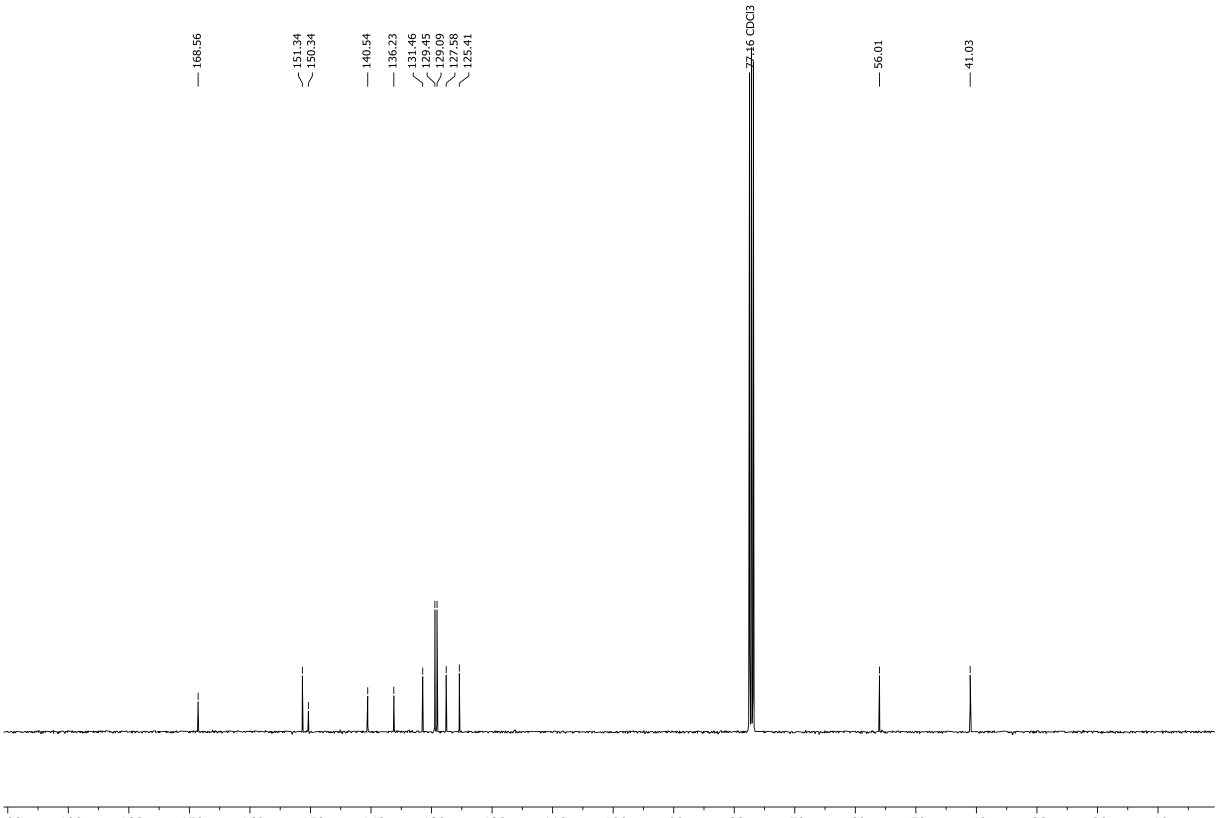

(*S*)-*tert*-Butyl 7-benzyl-5-oxo-5,7-dihydro-6*H*-pyrrolo[3,4-*b*]pyridine-6-carboxylate (4)

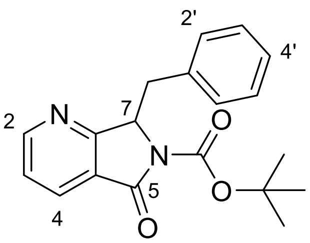

4

$C_{19}H_{20}N_2O_3$

M = 324.38 g mol^{-1}

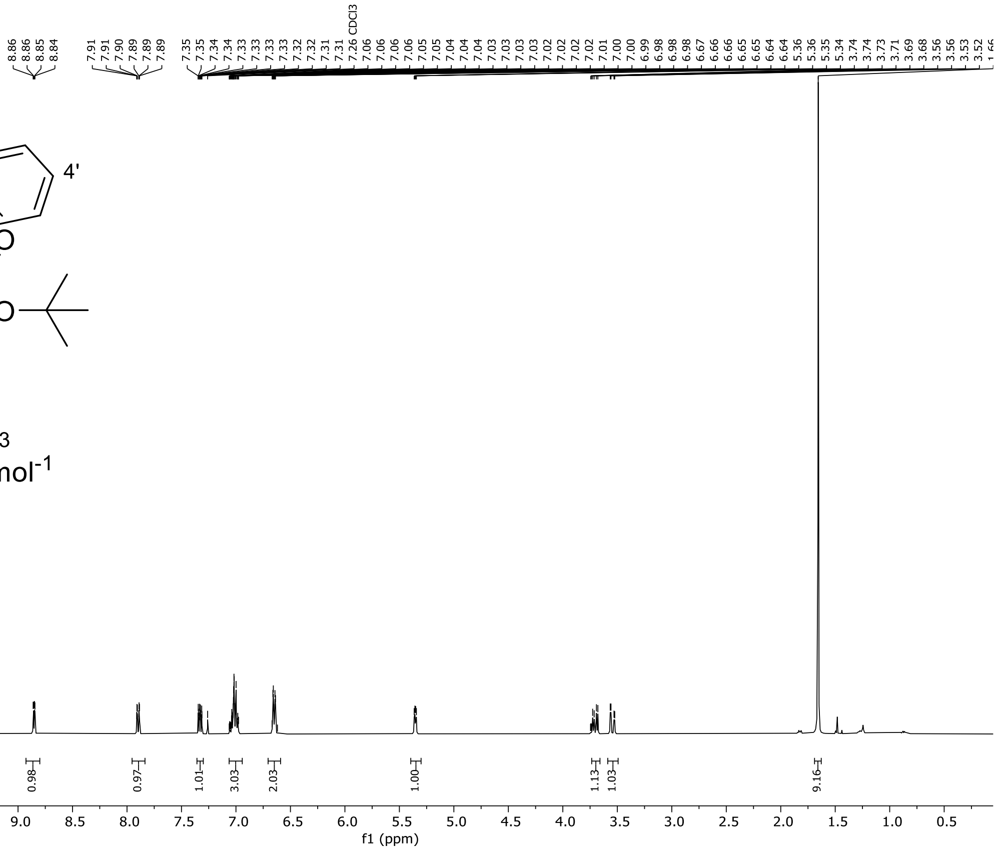

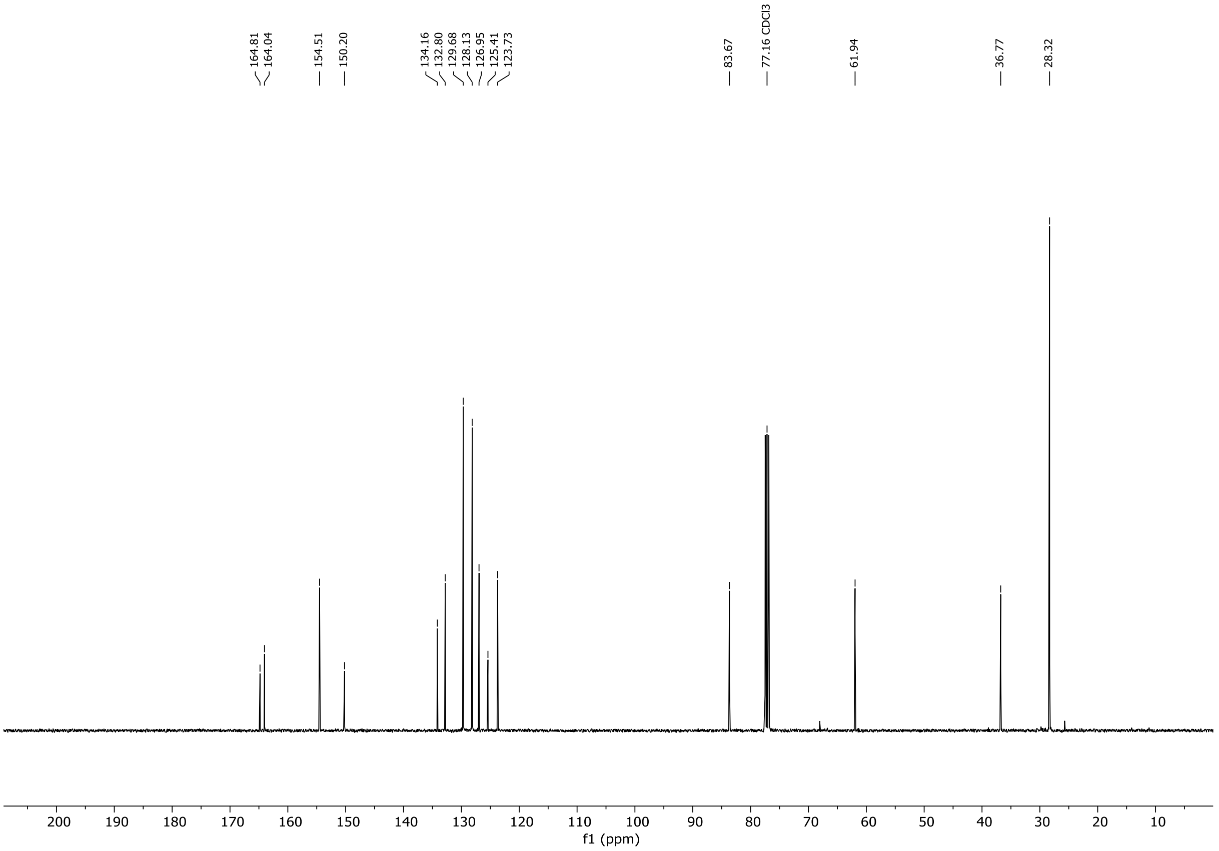

(*S*)-2-(1-((*tert*-Butoxycarbonyl)amino)-2-phenylethyl)nicotinic acid (5)

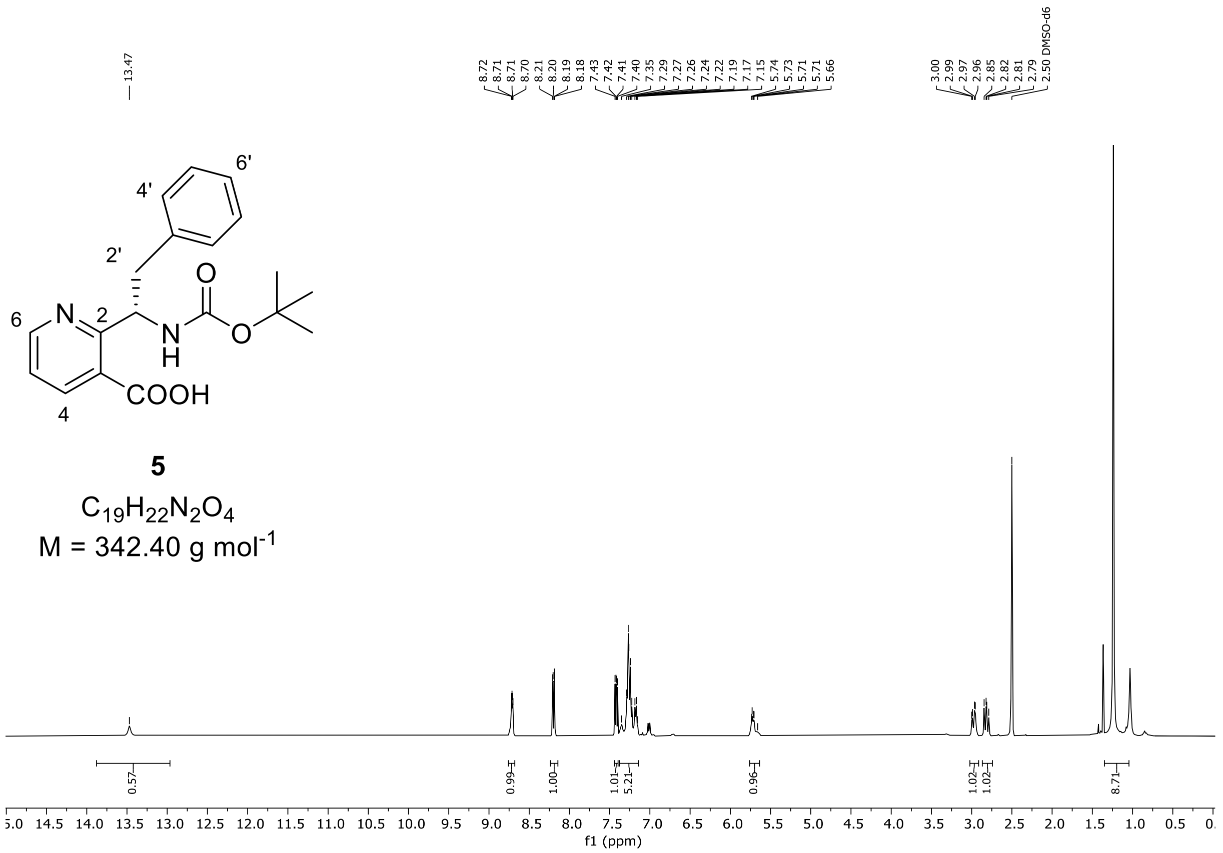

5

$C_{19}H_{22}N_2O_4$

M = 342.40 g mol^{-1}

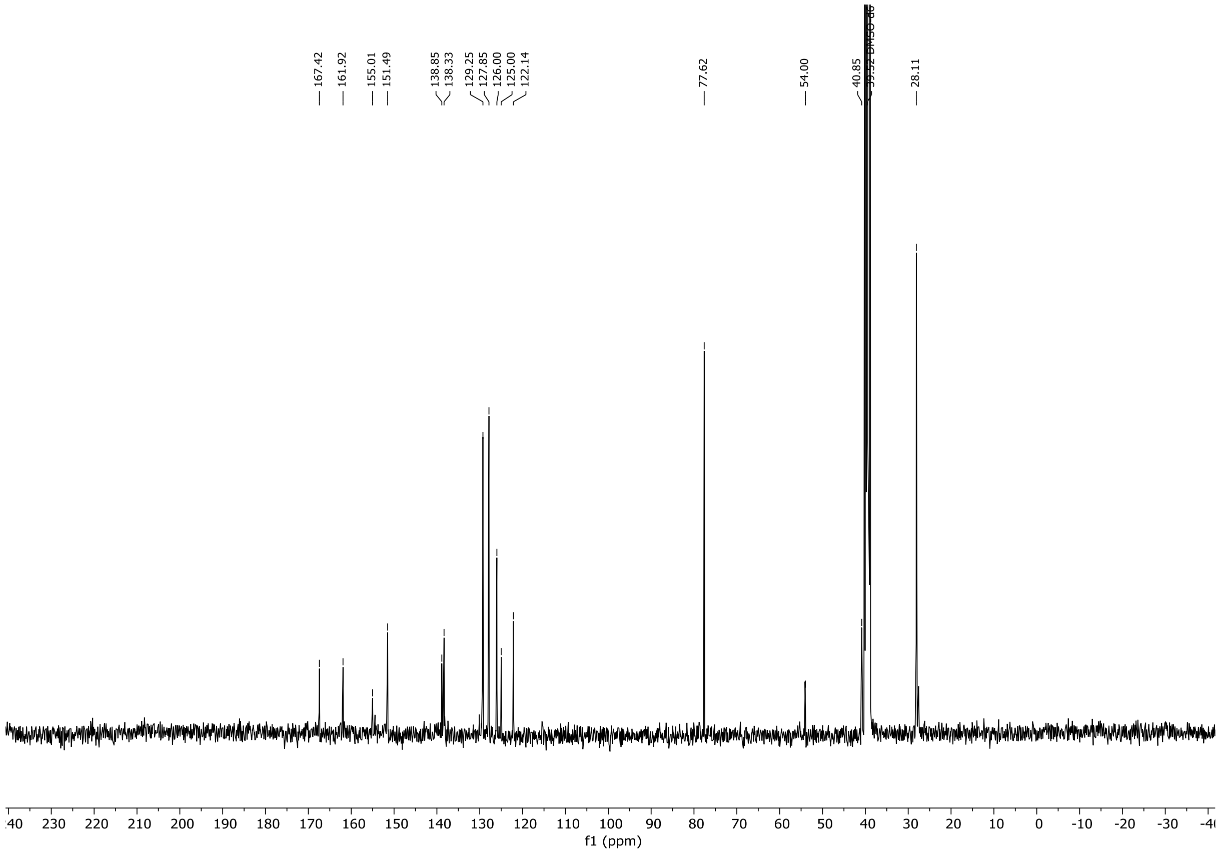

***tert*-Butyl (*S*)-(1-(3-(hydroxymethyl)pyridin-2-yl)-2-phenylethyl)carbamate (6)**

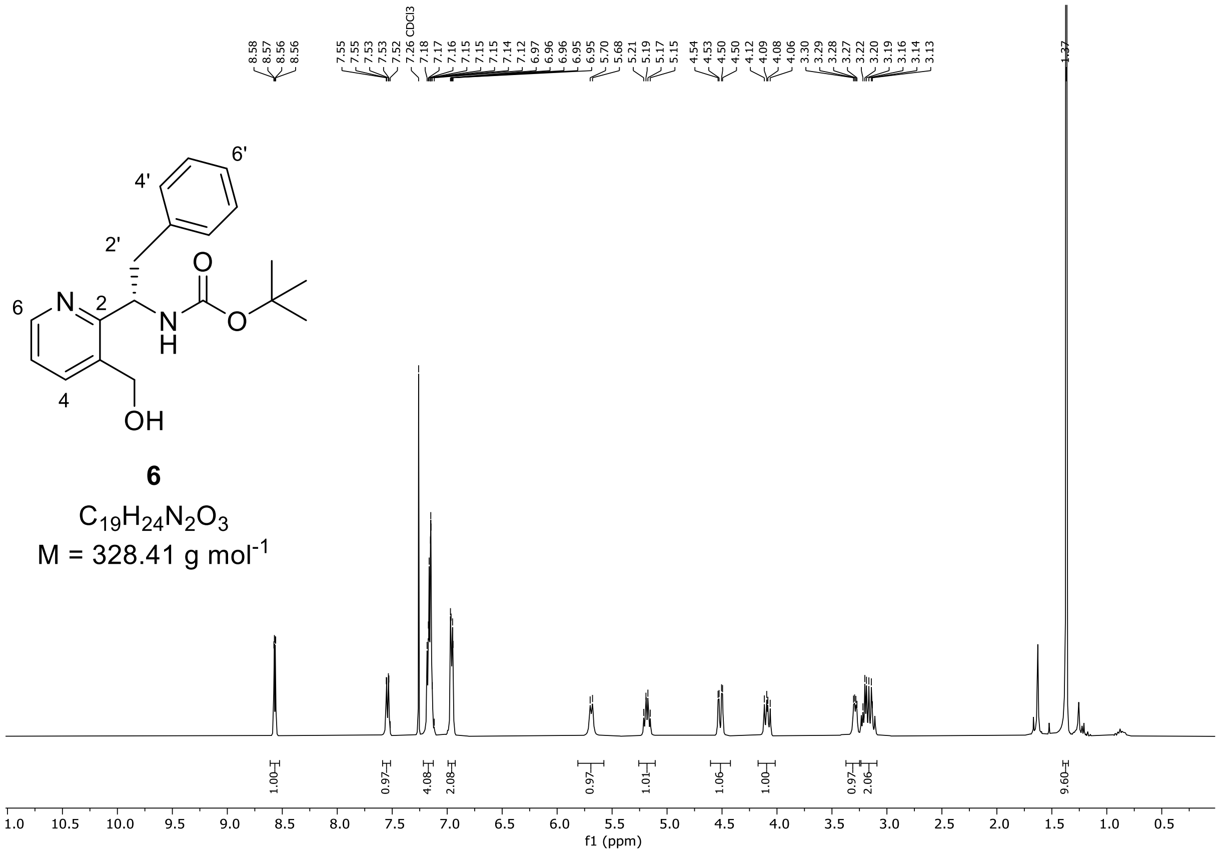

6

$C_{19}H_{24}N_2O_3$

M = 328.41 g mol^{-1}

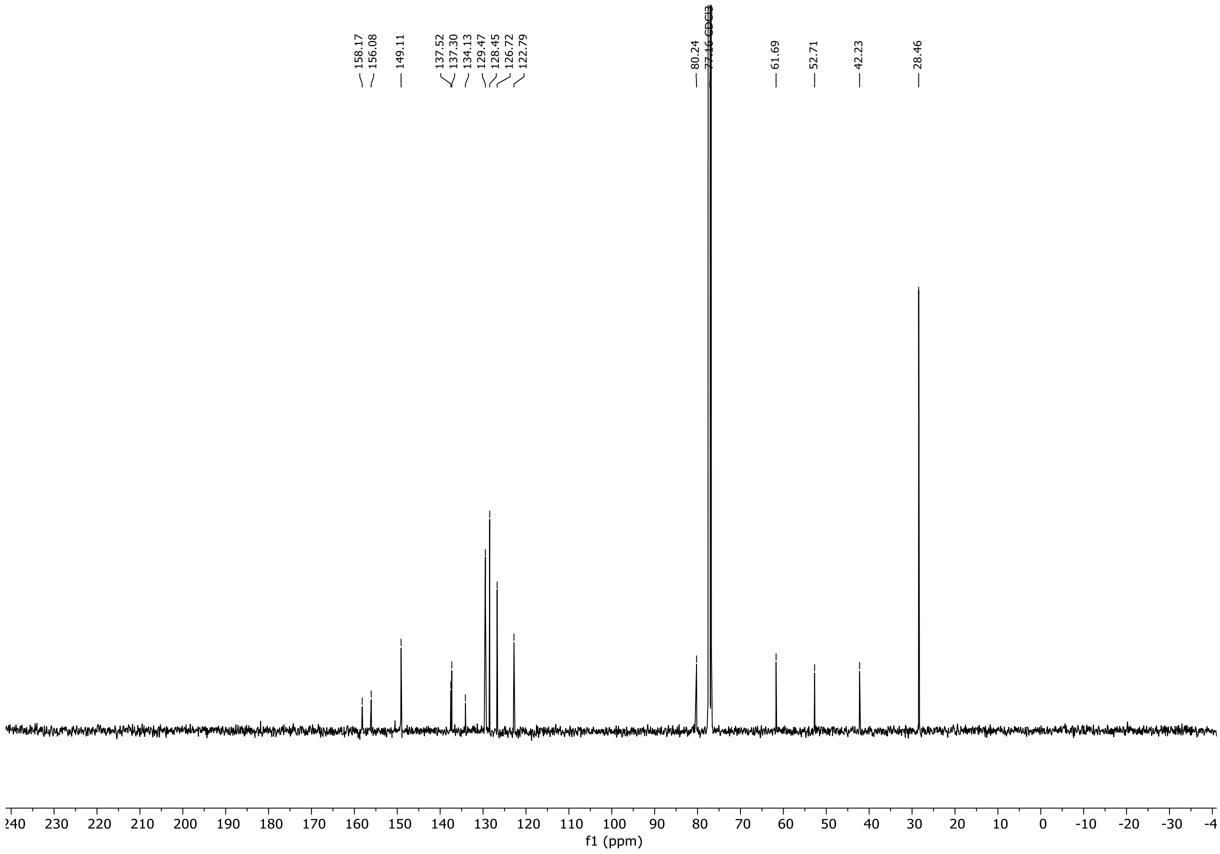

(*S*)-7-(cyclobutylmethyl)-1,2,3,4,6,7-hexahydro-5*H*-pyrrolo[3,4-*b*]pyridin-5-one (7)

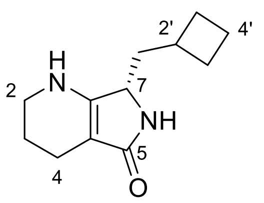

7

$C_{12}H_{18}N_2O$

M = 206.29 g mol^{-1}

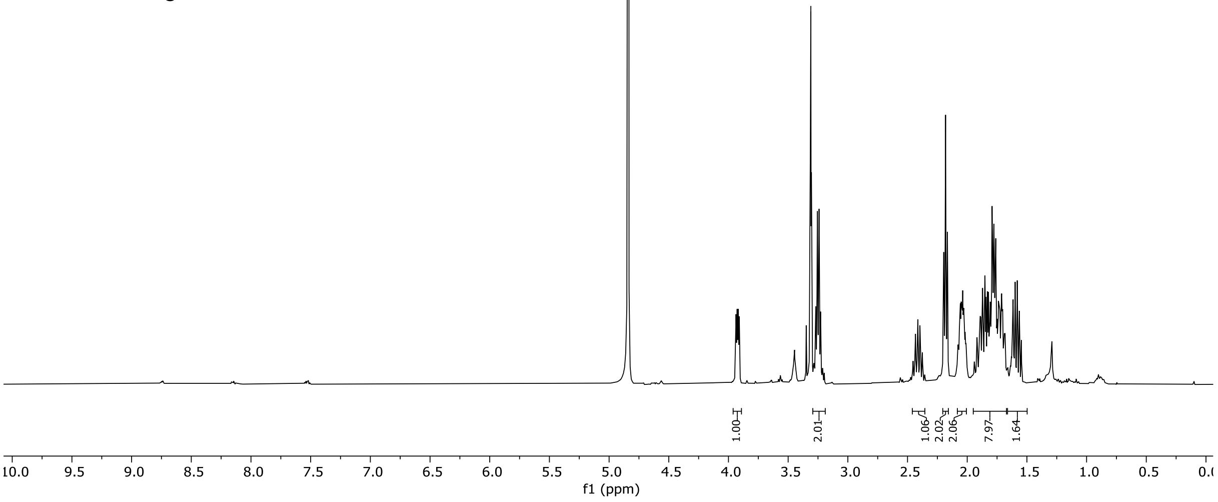

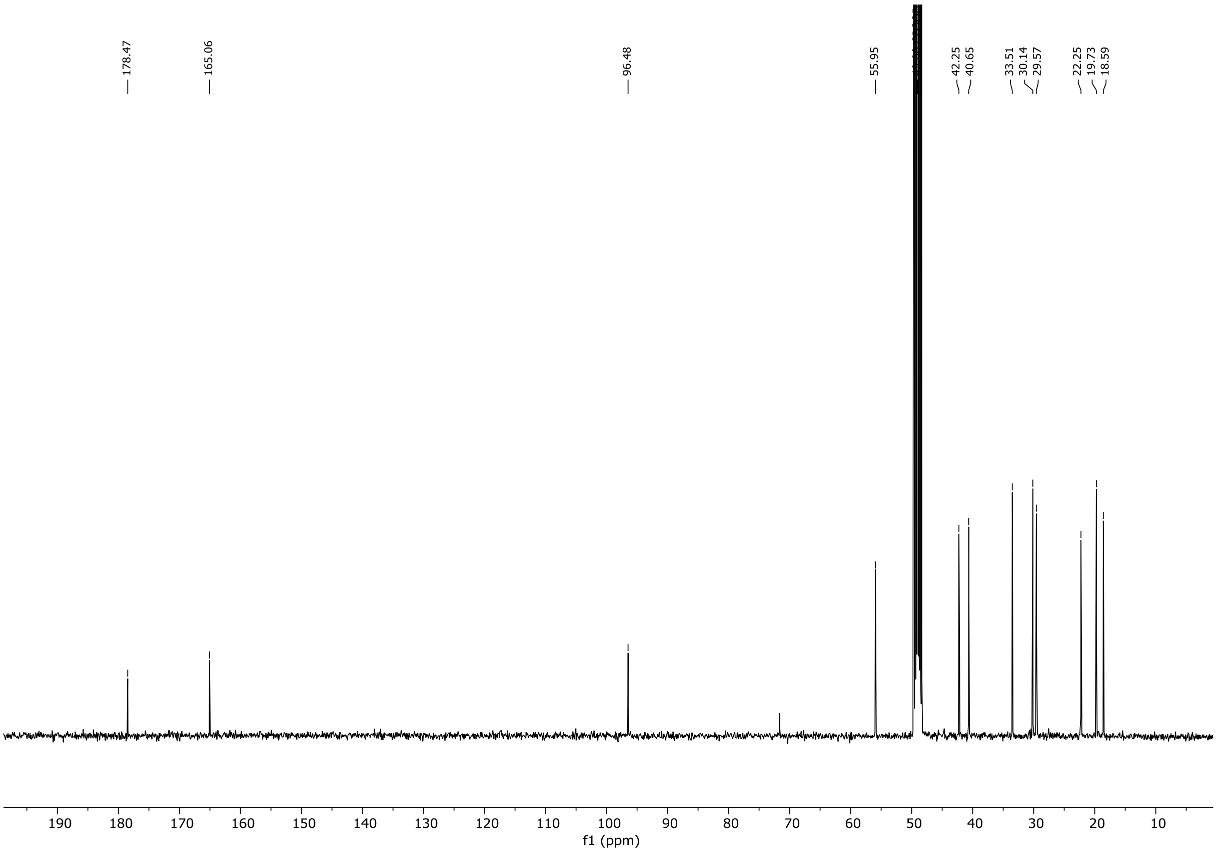

(*S*)-7-Benzyl-2-(cyclohex-1-en-1-yl)-6,7-dihydro-5*H*-pyrrolo[3,4-*b*]pyridin-5-one (3x)

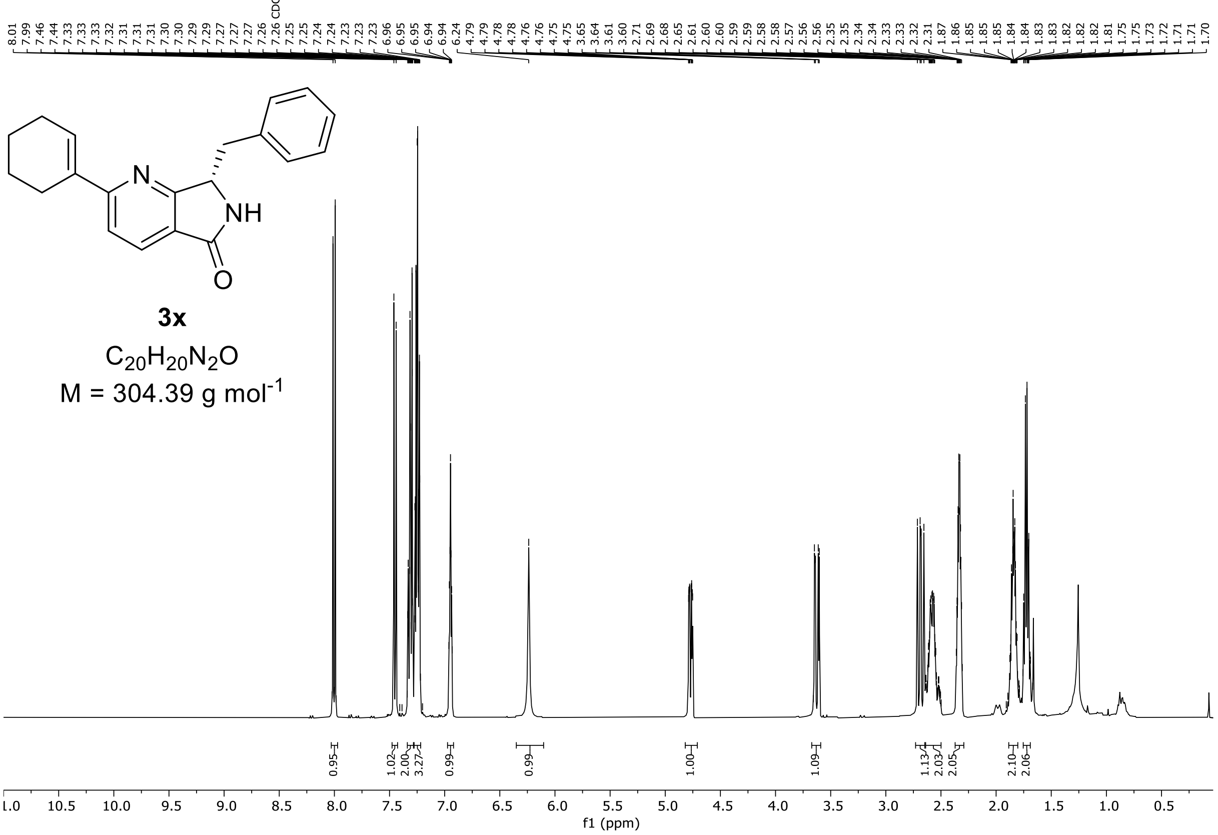

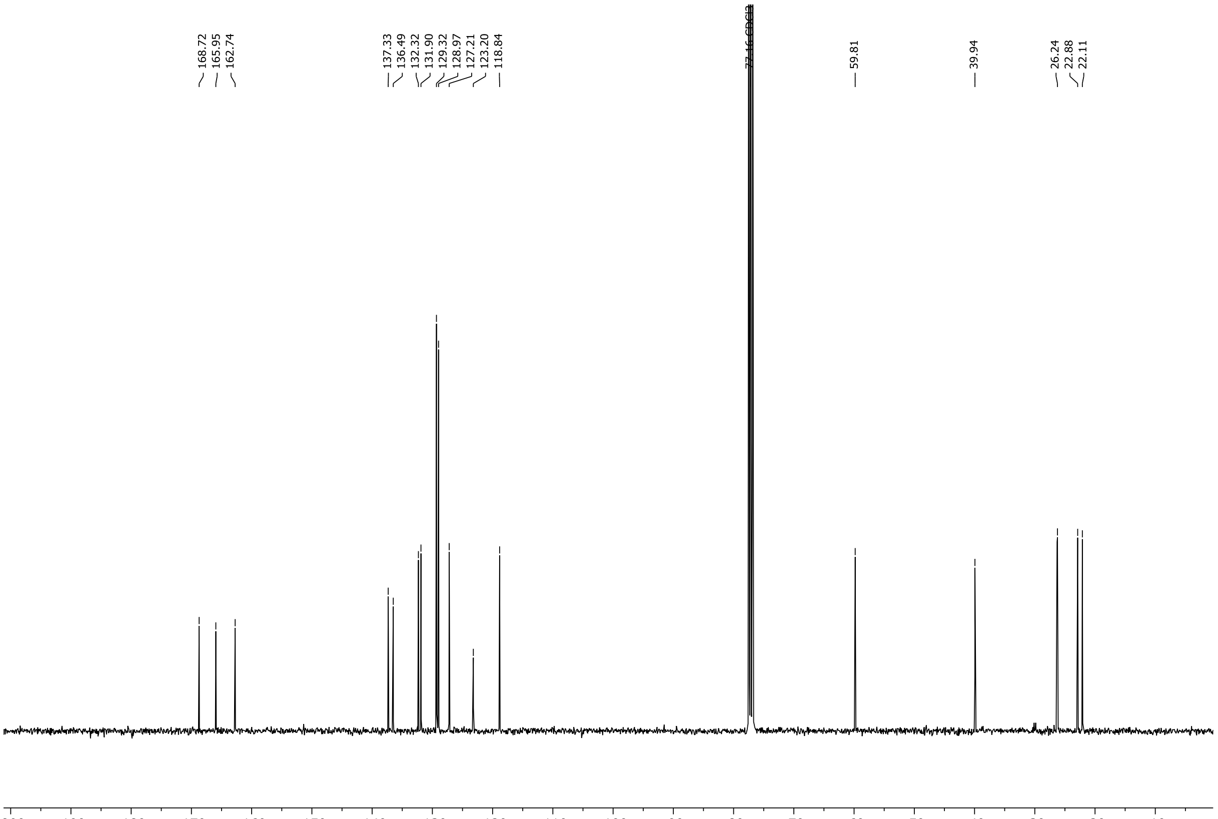

15. Chiral HPLC Traces

(*S*)-7-Benzyl-6,7-dihydro-5*H*-pyrrolo[3,4-*b*]pyridin-5-one (3a)

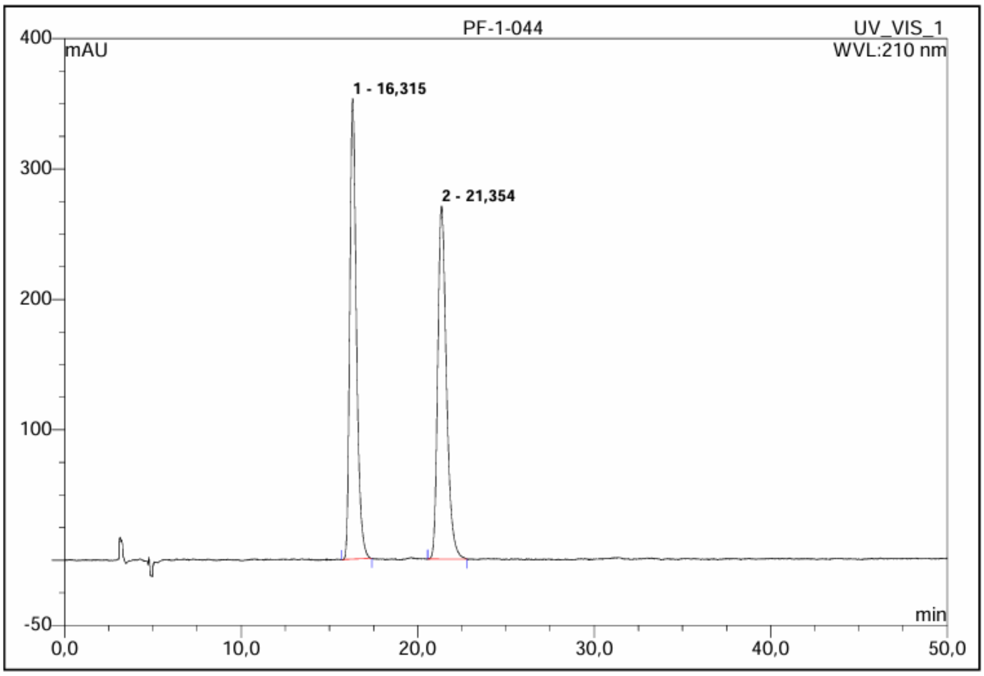

No.	Ret.Time min	Peak Name	Height mAU	Area mAU*min	Rel.Area %	Amount	Type
1	16,31	n.a.	353,152	153,756	49,84	n.a.	BMB*
2	21,35	n.a.	270,797	154,730	50,16	n.a.	BMB*
Total:			623,948	308,485	100,00	0,000	

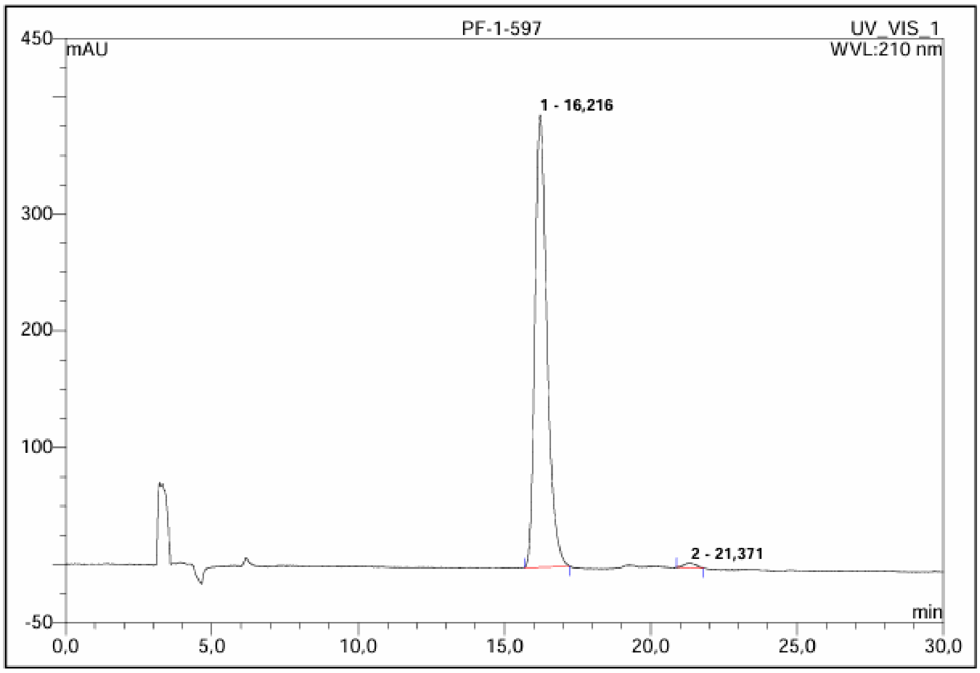

No.	Ret.Time min	Peak Name	Height mAU	Area mAU*min	Rel.Area %	Amount	Type
1	16,22	n.a.	386,675	183,745	99,03	n.a.	BMB
2	21,37	n.a.	3,986	1,792	0,97	n.a.	BMB*
Total:			390,661	185,537	100,00	0,000	

0.5 mmol scale

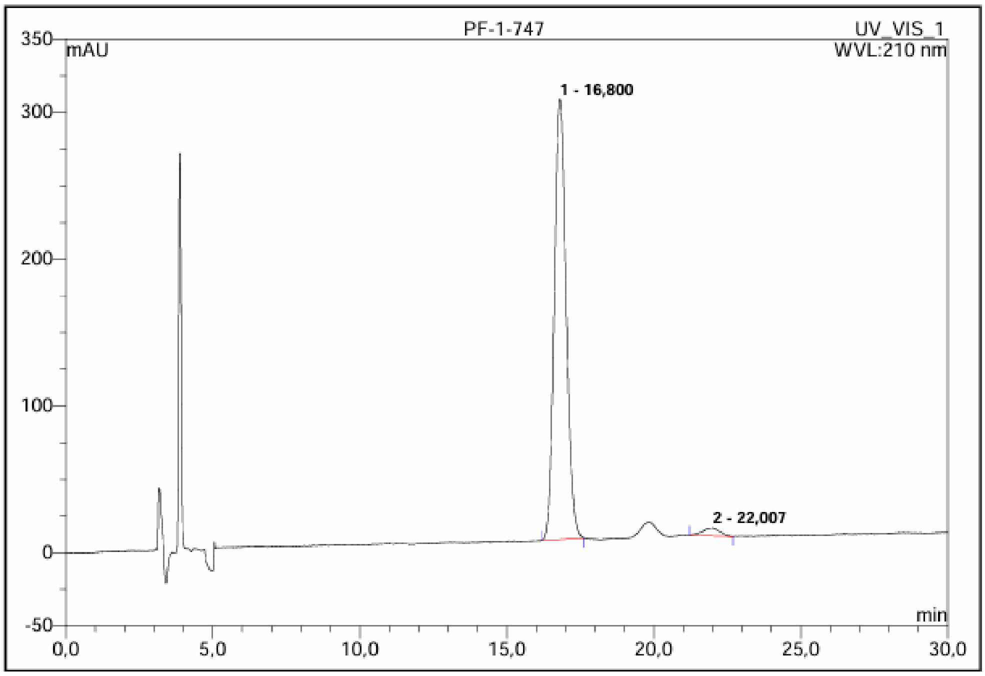

No.	Ret.Time min	Peak Name	Height mAU	Area mAU*min	Rel.Area %	Amount	Type
1	16,80	n.a.	300,147	140,054	97,60	n.a.	BMB
2	22,01	n.a.	5,358	3,449	2,40	n.a.	BMB*
Total:			305,505	143,503	100,00	0,000	

(*S*)-7-(4-(*tert*-Butyl)benzyl)-6,7-dihydro-5*H*-pyrrolo[3,4-*b*]pyridin-5-one (3b)

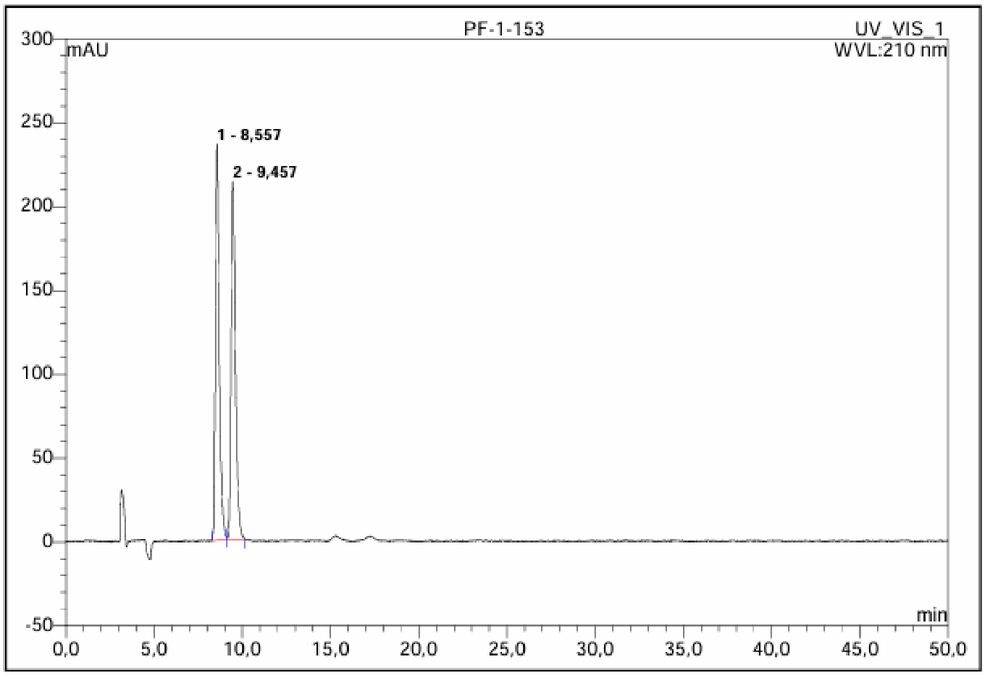

No.	Ret.Time min	Peak Name	Height mAU	Area mAU*min	Rel.Area %	Amount	Type
1	8,56	n.a.	236,370	61,628	49,97	n.a.	BM
2	9,46	n.a.	213,729	61,695	50,03	n.a.	MB
Total:			450,098	123,323	100,00	0,000	

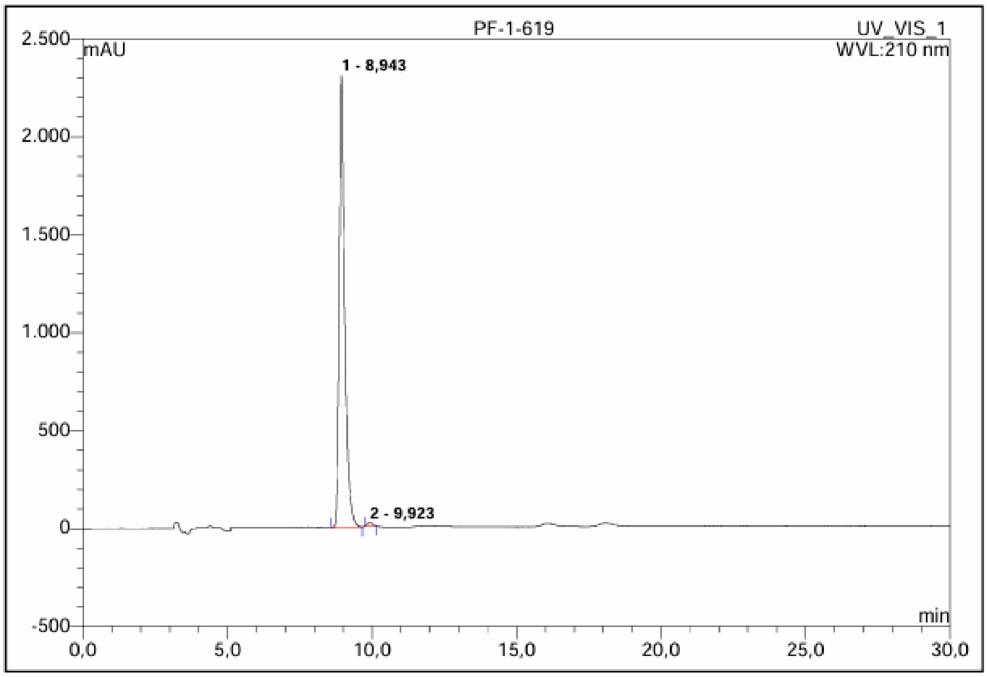

No.	Ret.Time min	Peak Name	Height mAU	Area mAU*min	Rel.Area %	Amount	Type
1	8,94	n.a.	2306,177	502,289	99,27	n.a.	BM *
2	9,92	n.a.	17,336	3,677	0,73	n.a.	BMB*
Total:			2323,513	505,966	100,00	0,000	

(*S*)-7-(3,5-Dimethylbenzyl)-6,7-dihydro-5*H*-pyrrolo[3,4-*b*]pyridin-5-one (3c)

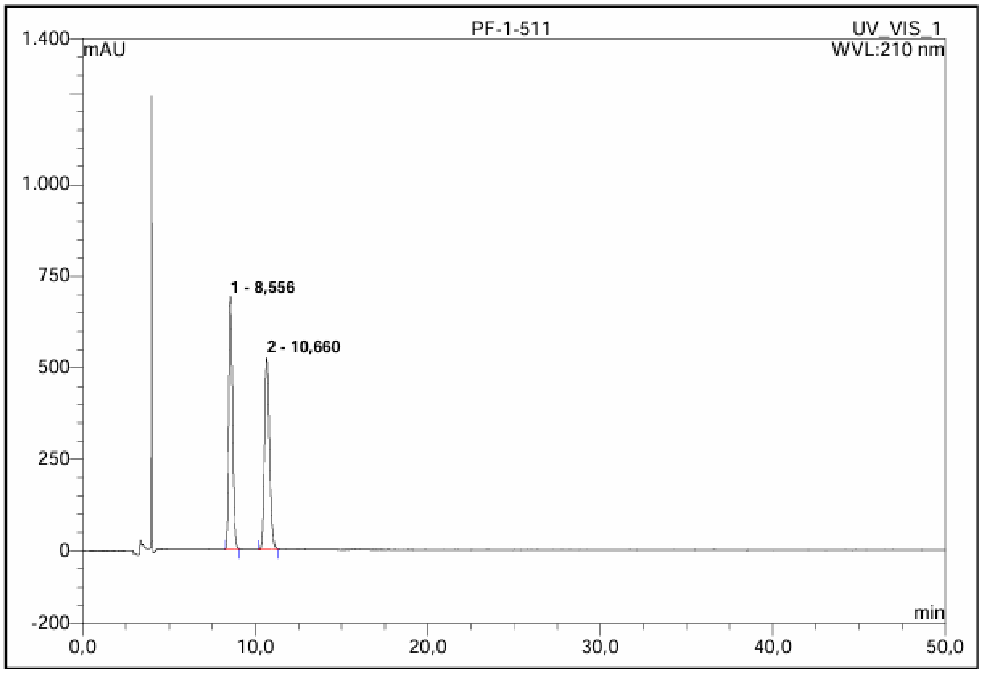

No.	Ret.Time min	Peak Name	Height mAU	Area mAU*min	Rel.Area %	Amount	Type
1	8,56	n.a.	692,681	175,525	49,89	n.a.	BMB
2	10,66	n.a.	526,237	176,272	50,11	n.a.	BMB
Total:			1218,919	351,798	100,00	0,000	

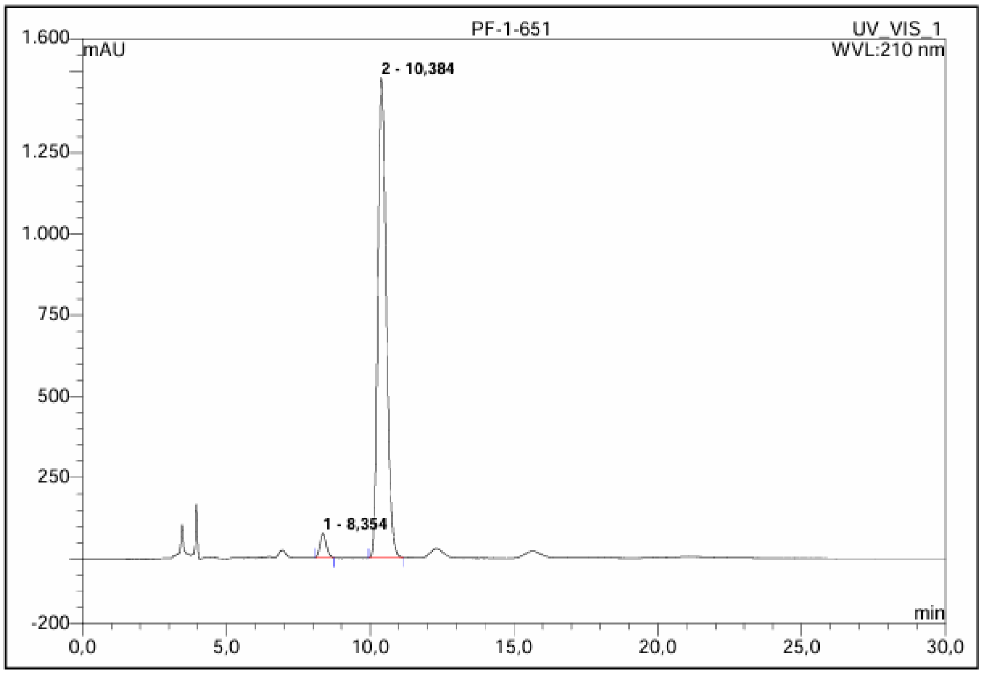

No.	Ret.Time min	Peak Name	Height mAU	Area mAU*min	Rel.Area %	Amount	Type
1	8,35	n.a.	73,792	18,538	3,53	n.a.	BMB*
2	10,38	n.a.	1477,421	506,162	96,47	n.a.	BM *
Total:			1551,212	524,700	100,00	0,000	

(*S*)-7-(4-Fluorobenzyl)-6,7-dihydro-5*H*-pyrrolo[3,4-*b*]pyridin-5-one (3d)

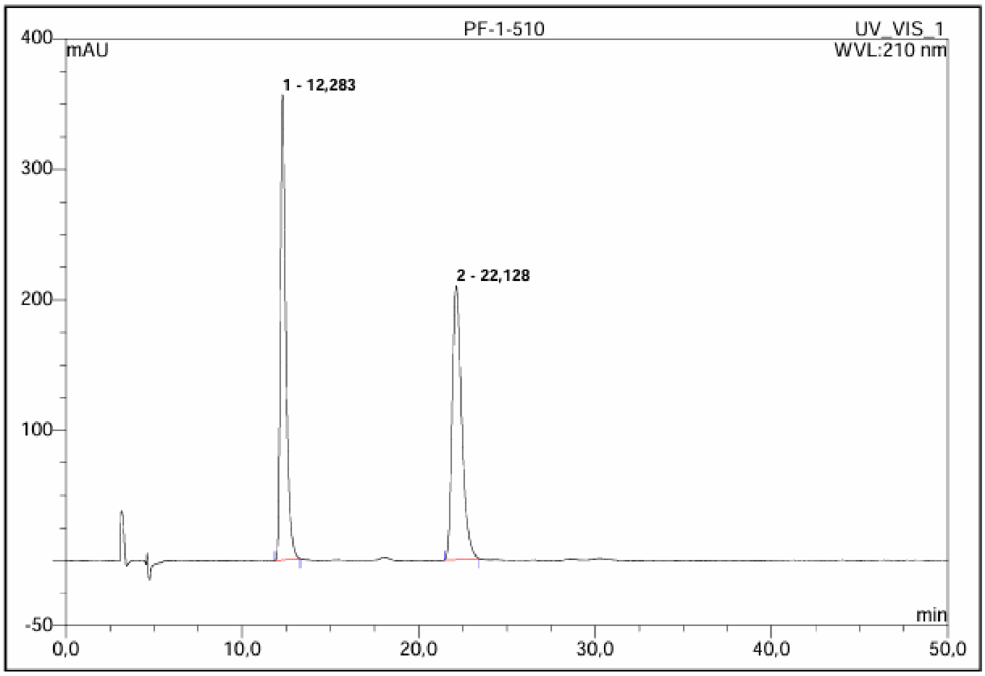

No.	Ret.Time min	Peak Name	Height mAU	Area mAU*min	Rel.Area %	Amount	Type
1	12,28	n.a.	357,059	130,858	50,04	n.a.	BMB
2	22,13	n.a.	210,135	130,646	49,96	n.a.	BMB
Total:			567,193	261,504	100,00	0,000	

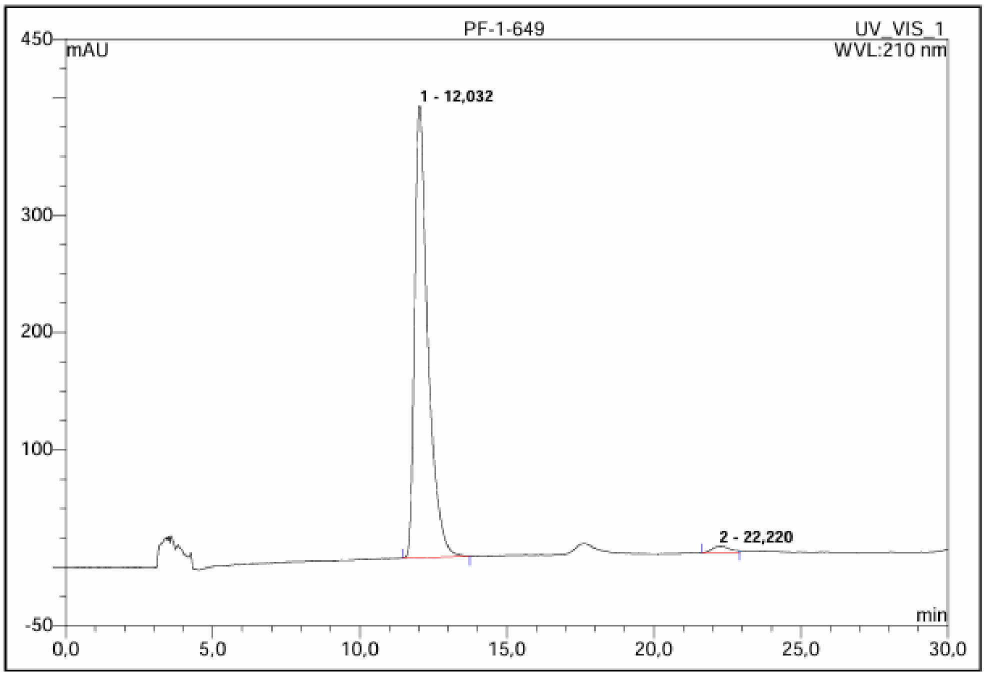

No.	Ret.Time min	Peak Name	Height mAU	Area mAU*min	Rel.Area %	Amount	Type
1	12,03	n.a.	385,196	201,329	97,97	n.a.	BMB
2	22,22	n.a.	5,798	4,176	2,03	n.a.	BM *
Total:			390,994	205,505	100,00	0,000	

(*S*)-7-(4-Bromobenzyl)-6,7-dihydro-5*H*-pyrrolo[3,4-*b*]pyridin-5-one (3e)

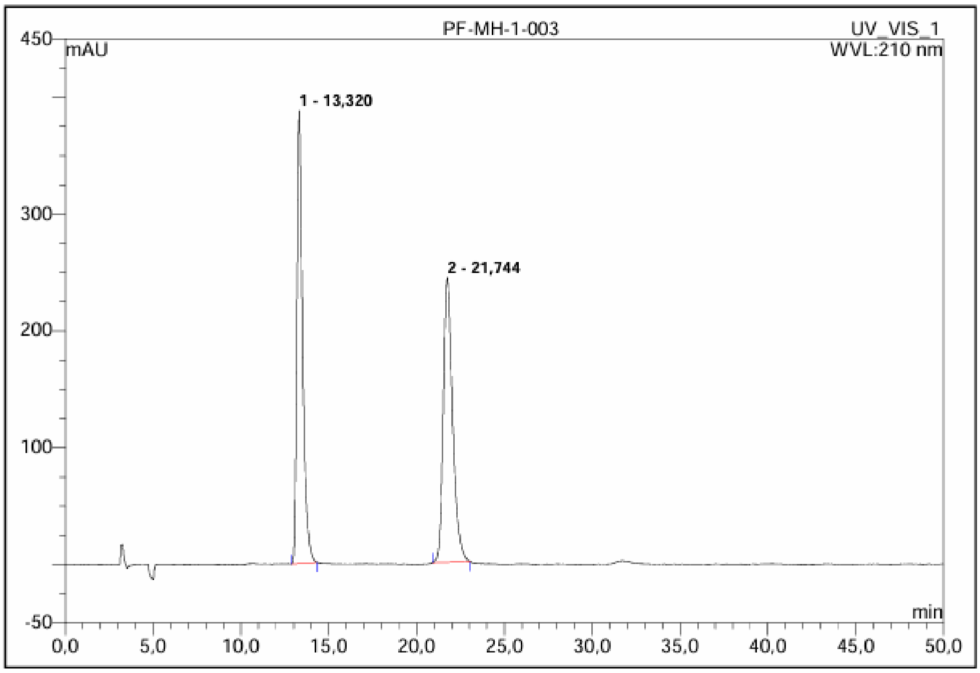

No.	Ret.Time min	Peak Name	Height mAU	Area mAU*min	Rel.Area %	Amount	Type
1	13,32	n.a.	387,682	149,070	49,83	n.a.	BMB
2	21,74	n.a.	243,670	150,116	50,17	n.a.	BMB
Total:			631,352	299,186	100,00	0,000	

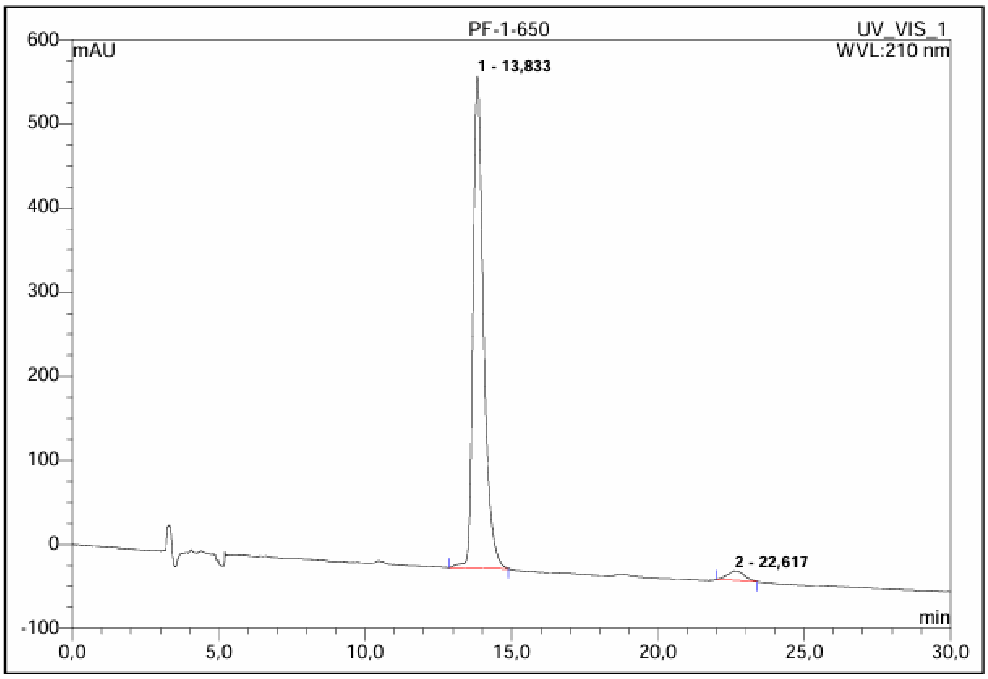

No.	Ret.Time min	Peak Name	Height mAU	Area mAU*min	Rel.Area %	Amount	Type
1	13,83	n.a.	584,542	244,609	97,08	n.a.	BMB
2	22,62	n.a.	11,259	7,364	2,92	n.a.	BMB*
Total:			595,801	251,973	100,00	0,000	

(*S*)-7-(Cyclopropylmethyl)-6,7-dihydro-5*H*-pyrrolo[3,4-*b*]pyridin-5-one (3f)

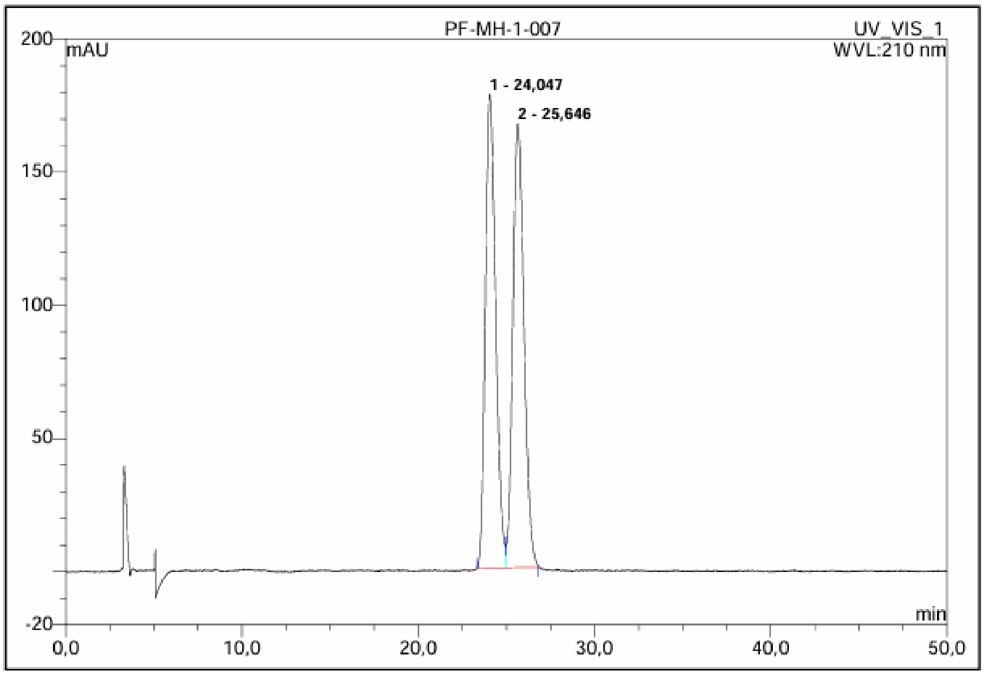

No.	Ret.Time min	Peak Name	Height mAU	Area mAU*min	Rel.Area %	Amount	Type
1	24,05	n.a.	177,978	121,176	49,80	n.a.	BM
2	25,65	n.a.	166,652	122,158	50,20	n.a.	MB
Total:			344,629	243,334	100,00	0,000	

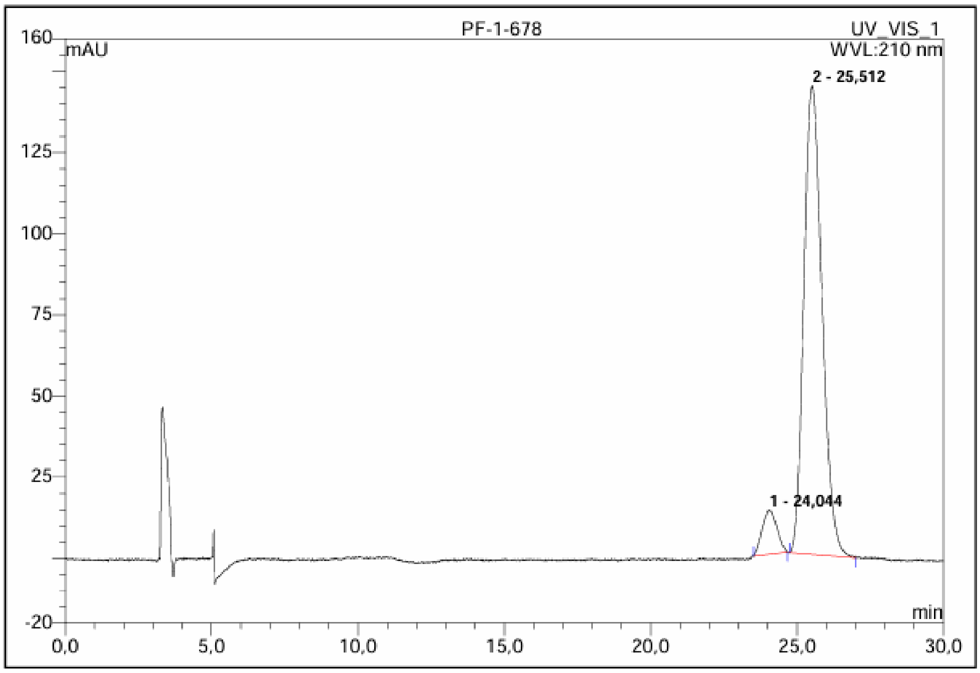

No.	Ret.Time min	Peak Name	Height mAU	Area mAU*min	Rel.Area %	Amount	Type
1	24,04	n.a.	13,629	7,900	7,08	n.a.	BMB*
2	25,51	n.a.	144,250	103,721	92,92	n.a.	BMB*
Total:			157,879	111,621	100,00	0,000	

(*S*)-7-(Cyclobutylmethyl)-6,7-dihydro-5*H*-pyrrolo[3,4-*b*]pyridin-5-one (3g)

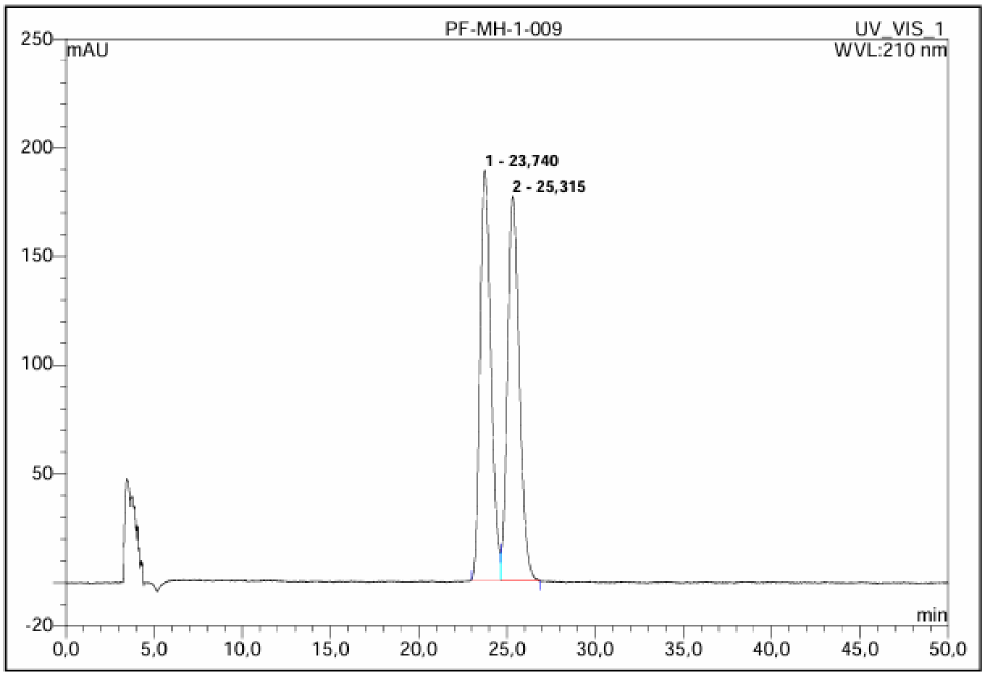

No.	Ret.Time min	Peak Name	Height mAU	Area mAU*min	Rel.Area %	Amount	Type
1	23,74	n.a.	188,400	134,823	49,50	n.a.	BM *
2	25,31	n.a.	176,898	137,540	50,50	n.a.	MB*
Total:			365,298	272,363	100,00	0,000	

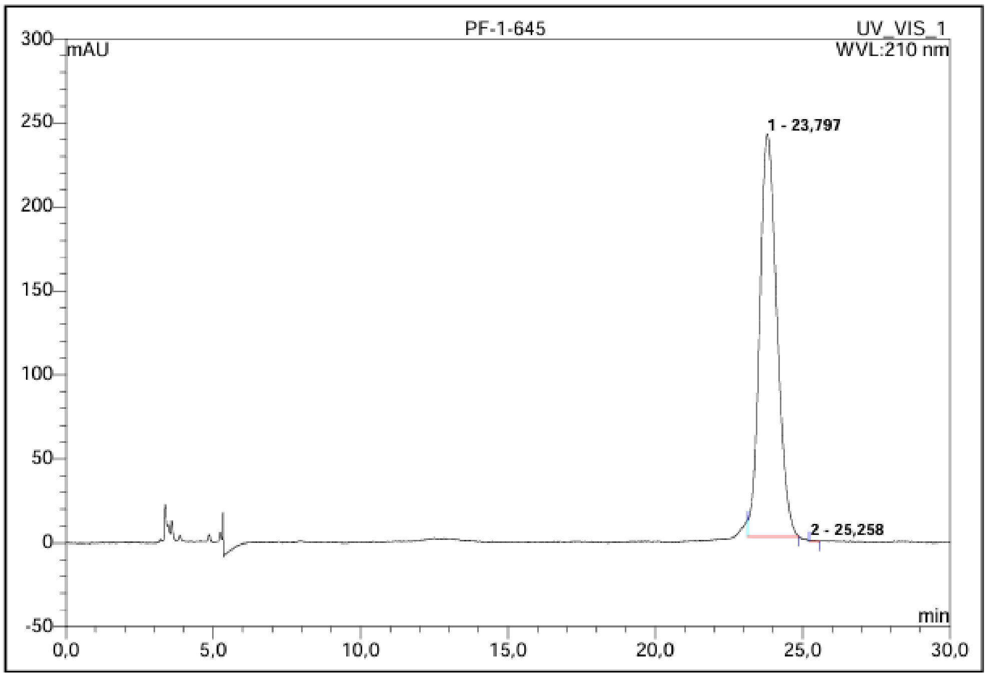

No.	Ret.Time min	Peak Name	Height mAU	Area mAU*min	Rel.Area %	Amount	Type
1	23,80	n.a.	239,426	162,639	99,93	n.a.	MB*
2	25,26	n.a.	0,754	0,107	0,07	n.a.	MB*
Total:			240,180	162,746	100,00	0,000	

0.20 mmol scale

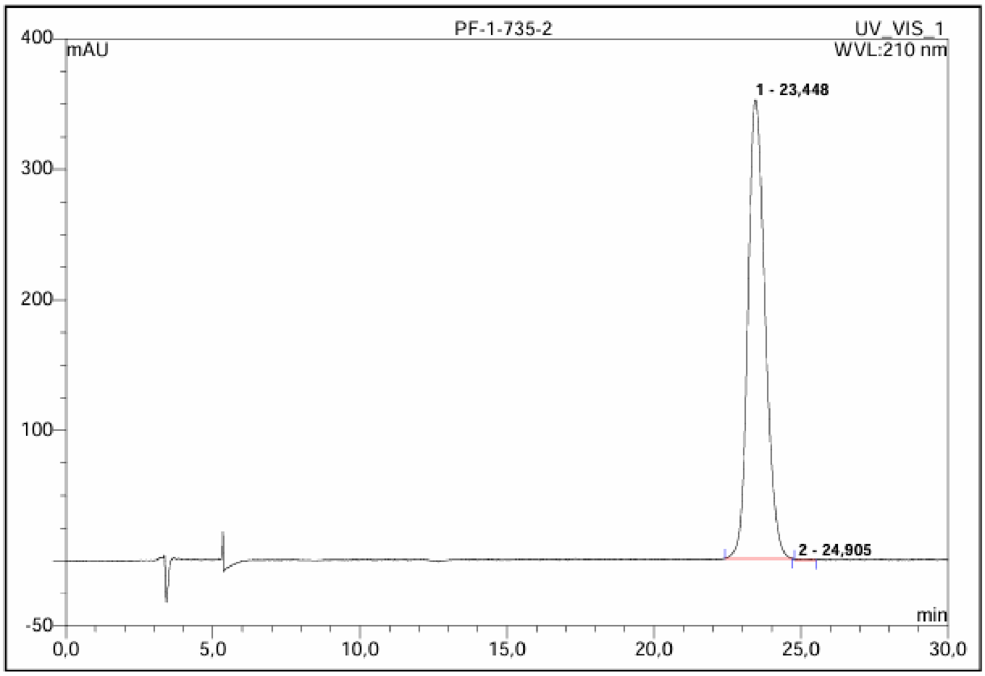

No.	Ret.Time min	Peak Name	Height mAU	Area mAU*min	Rel.Area %	Amount	Type
1	23,45	n.a.	351,778	237,163	99,83	n.a.	BMB*
2	24,90	n.a.	0,938	0,411	0,17	n.a.	M *
Total:			352,716	237,574	100,00	0,000	

(*S*)-*tert*-Butyl 3-((5-oxo-6,7-dihydro-5*H*-pyrrolo[3,4-*b*]pyridin-7-yl)methyl)azetidine-1-carboxylate (3h)

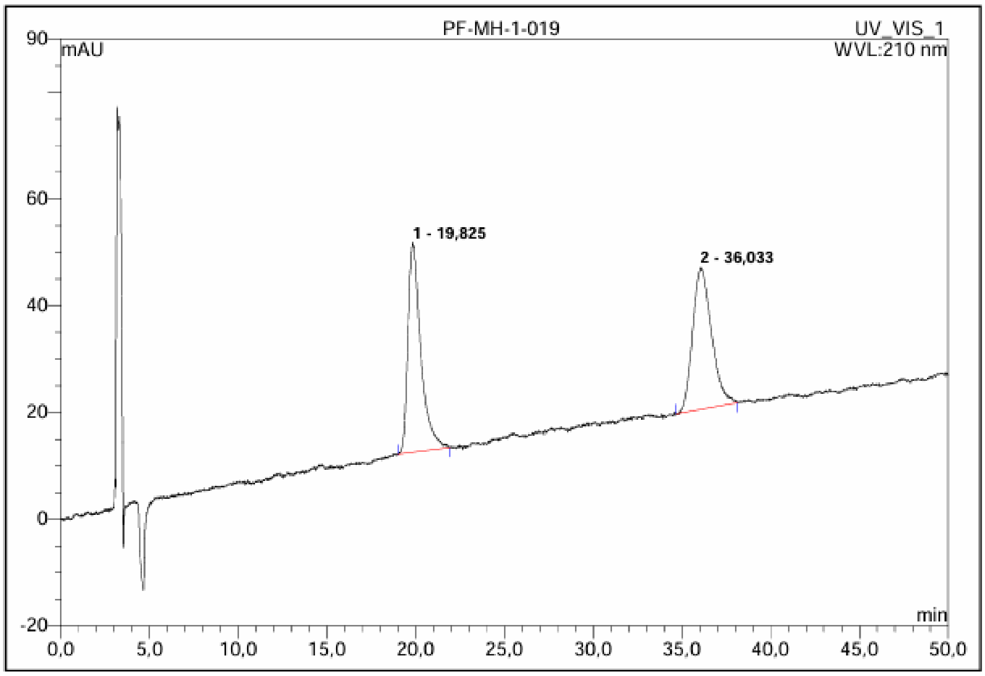

No.	Ret.Time min	Peak Name	Height mAU	Area mAU*min	Rel.Area %	Amount	Type
1	19,82	n.a.	39,319	32,748	49,99	n.a.	BMB*
2	36,03	n.a.	26,539	32,759	50,01	n.a.	BMB*
Total:			65,858	65,507	100,00	0,000	

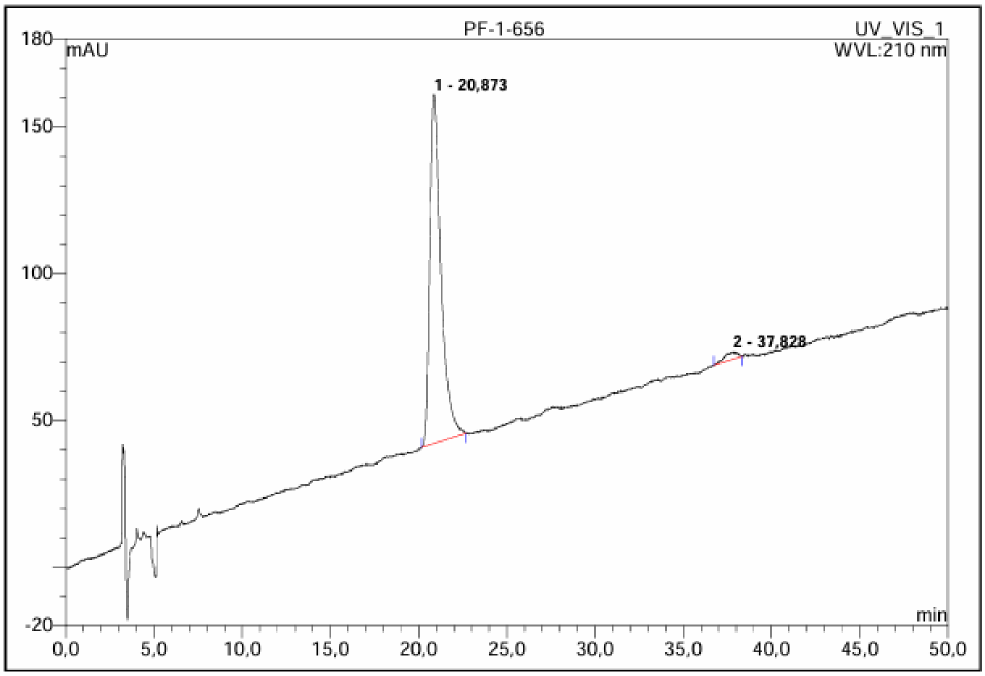

No.	Ret.Time min	Peak Name	Height mAU	Area mAU*min	Rel.Area %	Amount	Type
1	20,87	n.a.	118,939	86,988	97,56	n.a.	BMB*
2	37,83	n.a.	2,541	2,178	2,44	n.a.	BMB*
Total:			121,479	89,167	100,00	0,000	

(***S***)**-7-Ethyl-6,7-dihydro-5*****H*****-pyrrolo[3,4-*****b*****]pyridin-5-one (3i)**

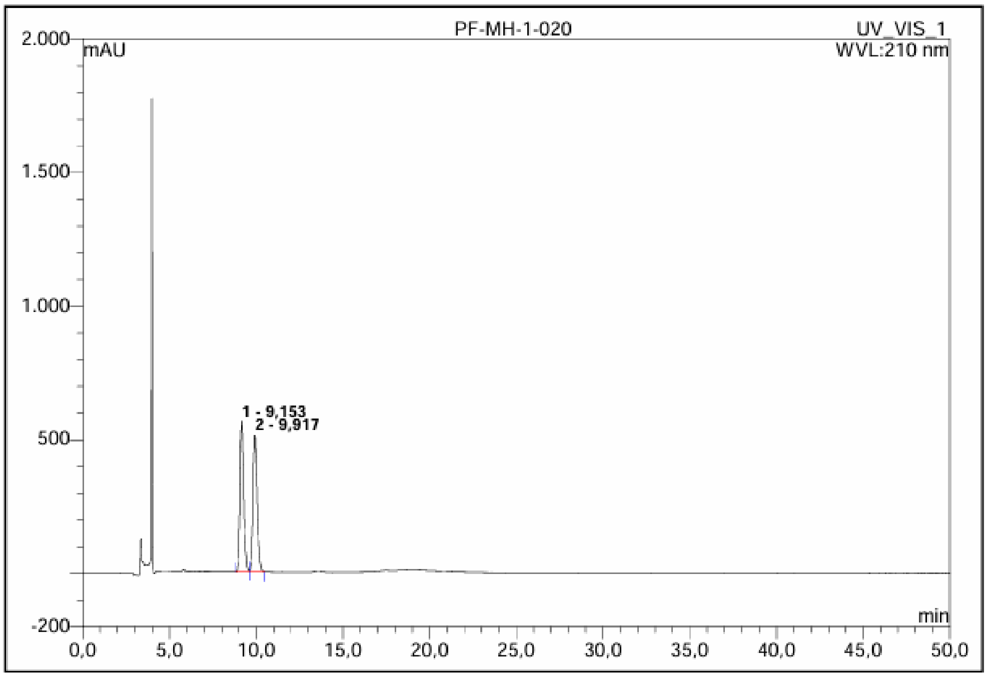

No.	Ret.Time min	Peak Name	Height mAU	Area mAU*min	Rel.Area %	Amount	Type
1	9,15	n.a.	566,026	141,391	50,04	n.a.	BM
2	9,92	n.a.	512,594	141,192	49,96	n.a.	MB
Total:			1078,619	282,583	100,00	0,000	

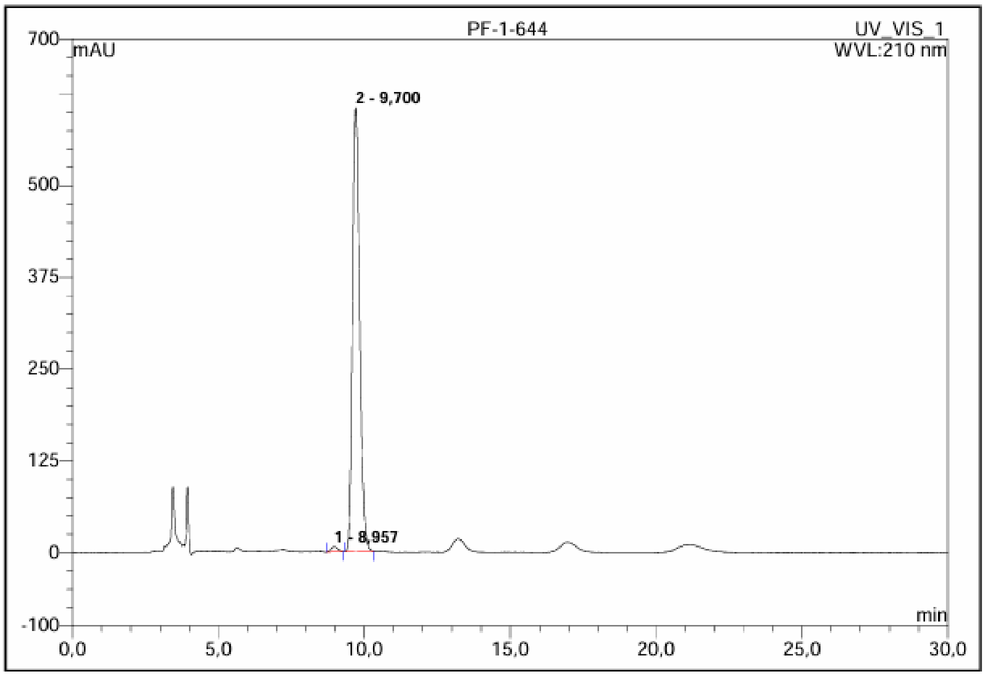

No.	Ret.Time min	Peak Name	Height mAU	Area mAU*min	Rel.Area %	Amount	Type
1	8,96	n.a.	6,874	1,682	0,97	n.a.	BMB*
2	9,70	n.a.	604,589	172,021	99,03	n.a.	BMB
Total:			611,463	173,703	100,00	0,000	

(*S*)-7-Butyl-6,7-dihydro-5*H*-pyrrolo[3,4-*b*]pyridin-5-one (3j)

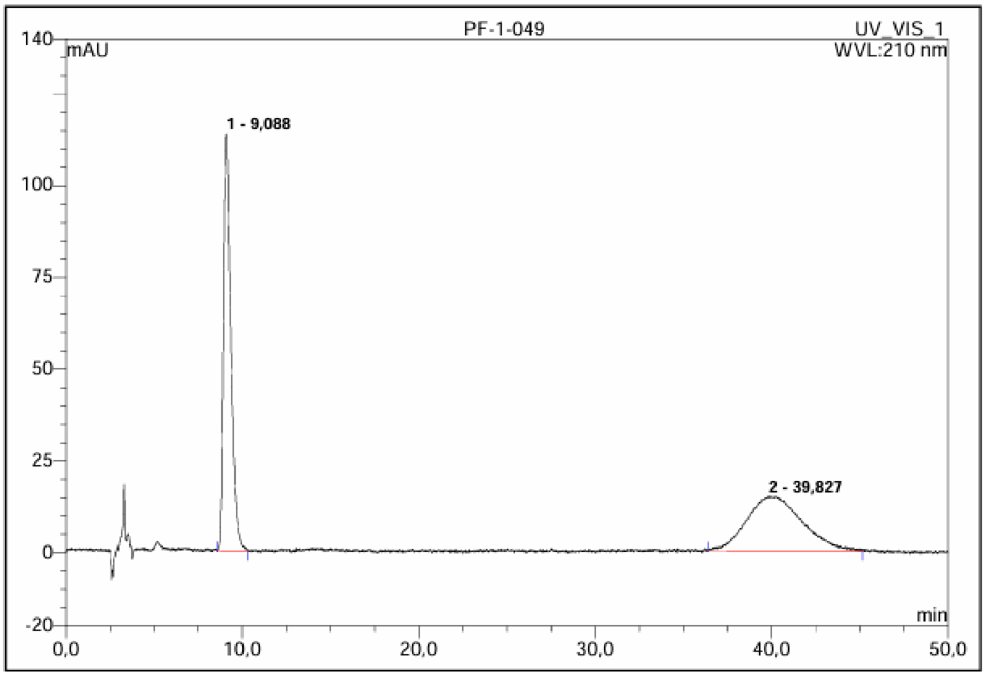

No.	Ret.Time min	Peak Name	Height mAU	Area mAU*min	Rel.Area %	Amount	Type
1	9,09	n.a.	113,739	55,439	50,38	n.a.	BMB*
2	39,83	n.a.	15,070	54,597	49,62	n.a.	BMB*
Total:			128,809	110,036	100,00	0,000	

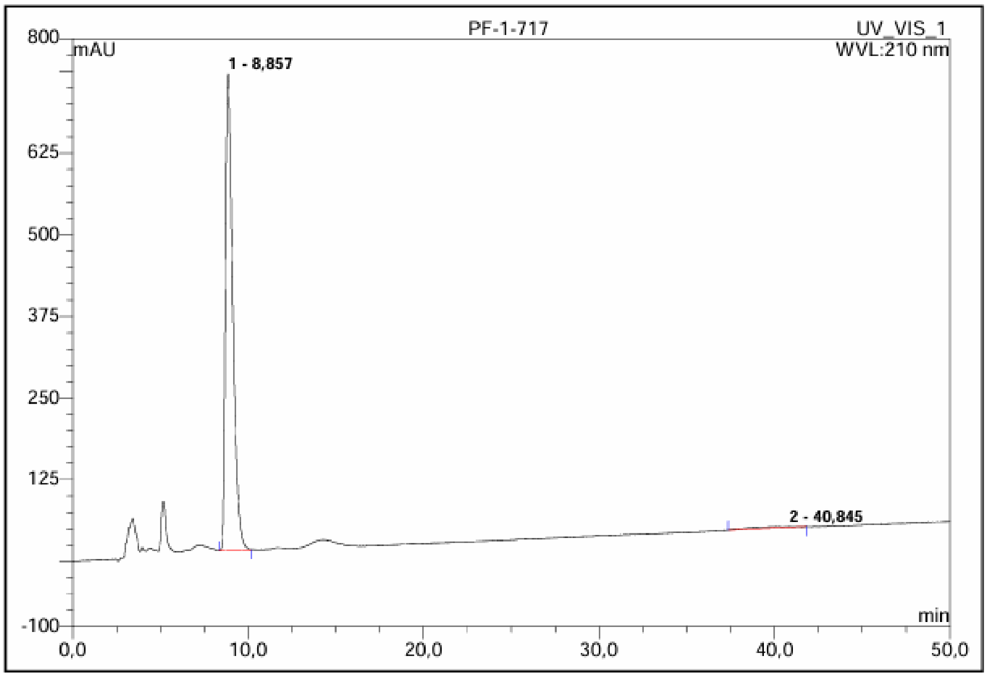

No.	Ret.Time min	Peak Name	Height mAU	Area mAU*min	Rel.Area %	Amount	Type
1	8,86	n.a.	729,393	351,831	98,59	n.a.	BMB*
2	40,84	n.a.	1,272	5,021	1,41	n.a.	BMB*
Total:			730,665	356,852	100,00	0,000	

(*S*)-2-Chloro-7-isopentyl-6,7-dihydro-5*H*-pyrrolo[3,4-*b*]pyridin-5-one (3k)

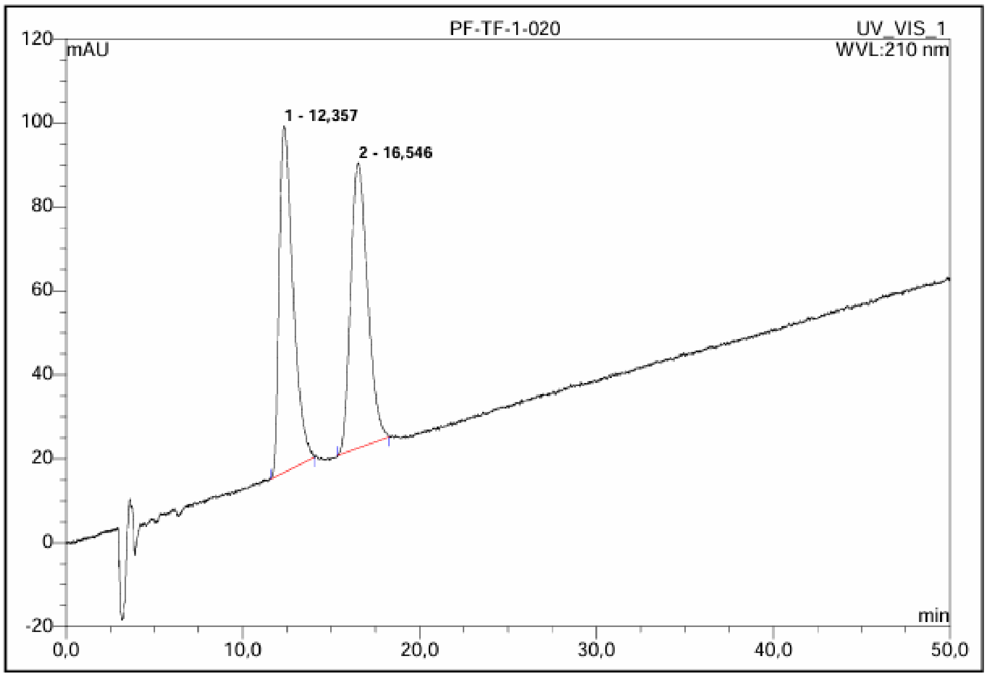

No.	Ret.Time min	Peak Name	Height mAU	Area mAU*min	Rel.Area %	Amount	Type
1	12,36	n.a.	82,451	74,612	49,69	n.a.	BMB*
2	16,55	n.a.	67,902	75,529	50,31	n.a.	BMB*
Total:			150,353	150,141	100,00	0,000	

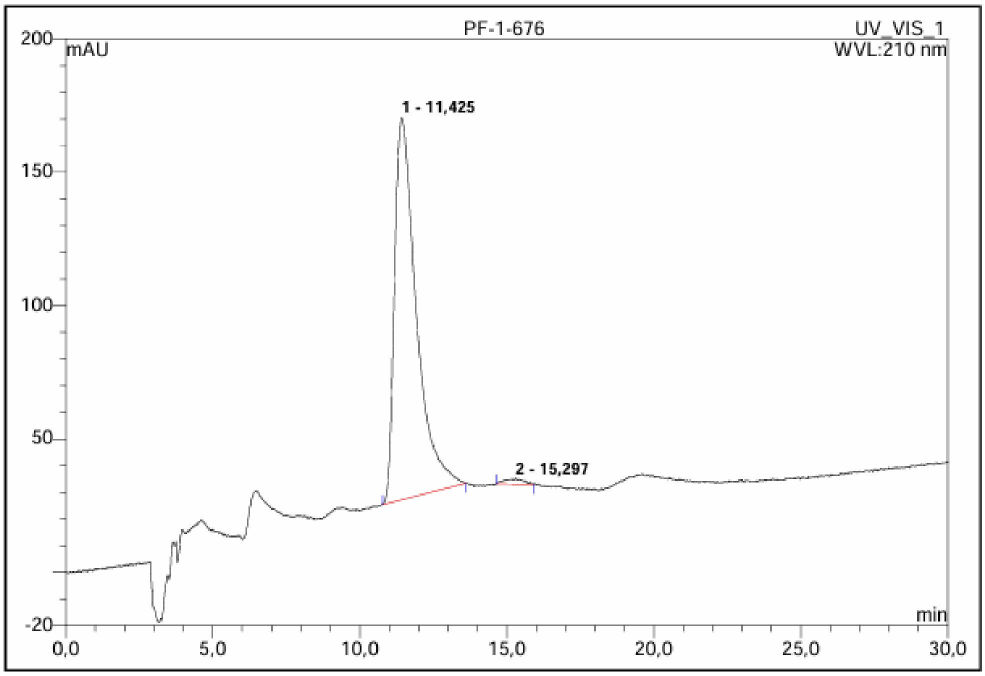

No.	Ret.Time min	Peak Name	Height mAU	Area mAU*min	Rel.Area %	Amount	Type
1	11,43	n.a.	143,235	122,866	98,77	n.a.	BMB*
2	15,30	n.a.	2,189	1,533	1,23	n.a.	BMB*
Total:			145,424	124,399	100,00	0,000	

(*S*)-7-Benzyl-2-bromo-6,7-dihydro-5*H*-pyrrolo[3,4-*b*]pyridin-5-one (3l)

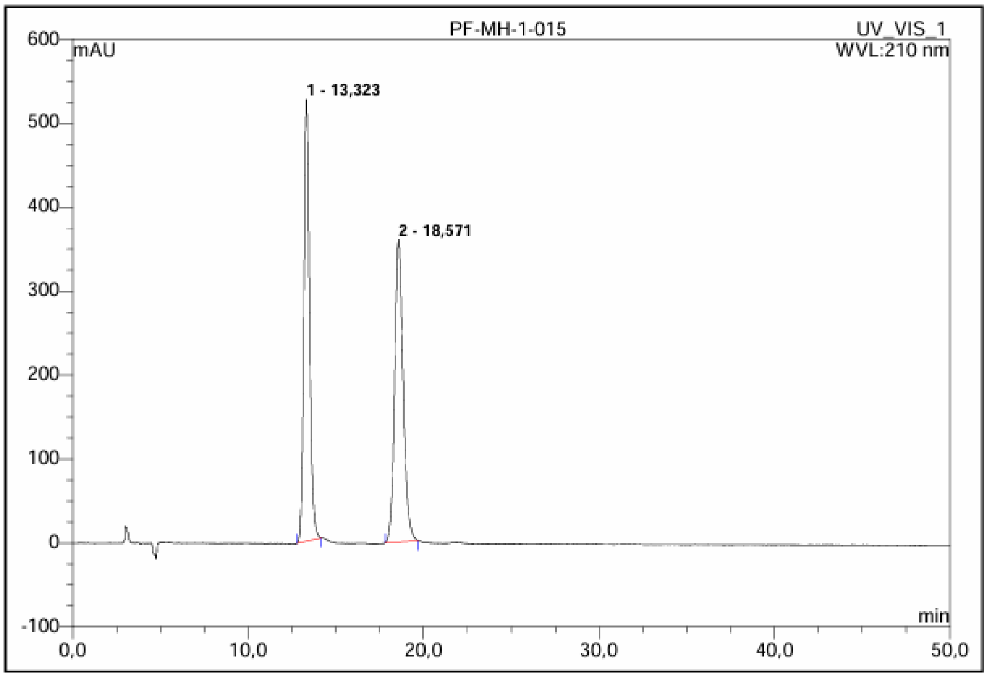

No.	Ret.Time min	Peak Name	Height mAU	Area mAU*min	Rel.Area %	Amount	Type
1	13,32	n.a.	526,168	198,103	49,79	n.a.	BMB
2	18,57	n.a.	359,877	199,791	50,21	n.a.	BMB
Total:			886,045	397,894	100,00	0,000	

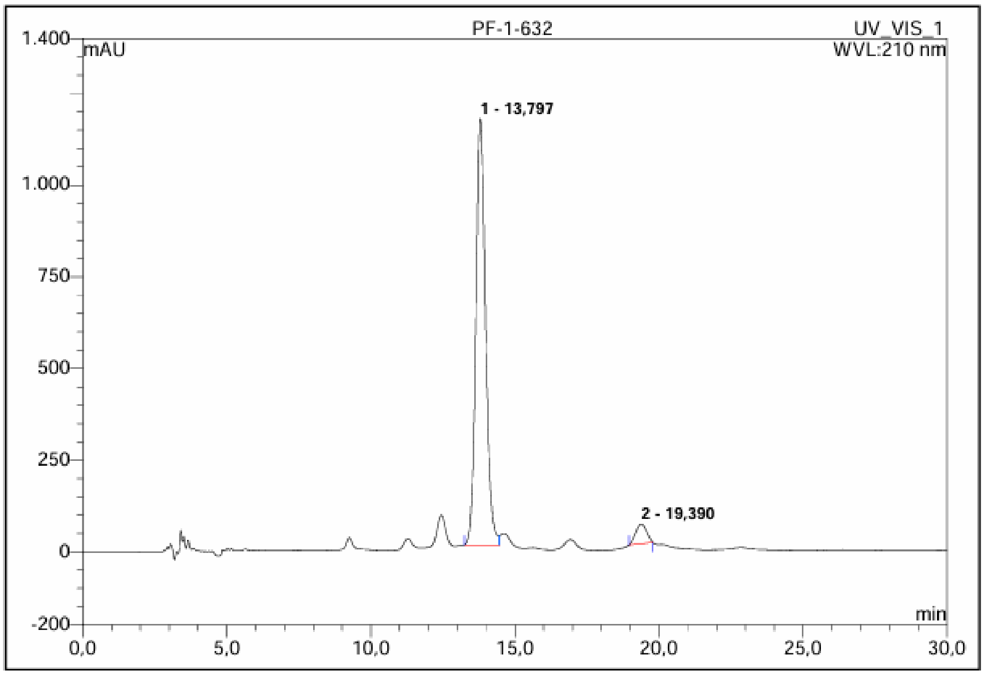

No.	Ret.Time min	Peak Name	Height mAU	Area mAU*min	Rel.Area %	Amount	Type
1	13,80	n.a.	1163,673	449,487	95,07	n.a.	BM *
2	19,39	n.a.	53,447	23,315	4,93	n.a.	BMB*
Total:			1217,120	472,803	100,00	0,000	

0.20 mmol scale

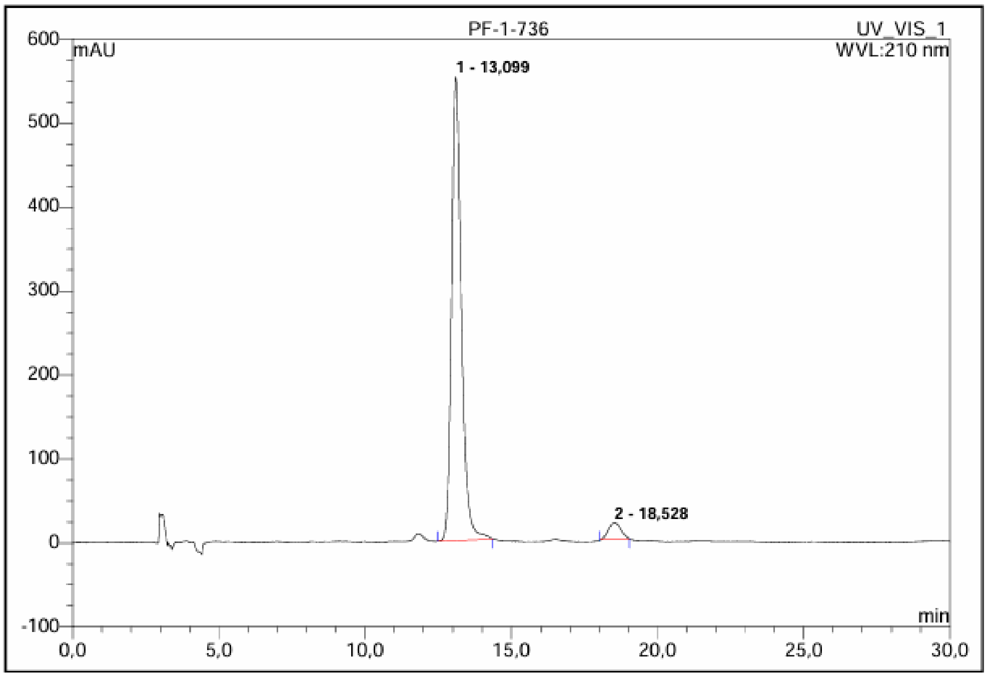

No.	Ret.Time min	Peak Name	Height mAU	Area mAU*min	Rel.Area %	Amount	Type
1	13,10	n.a.	552,130	213,207	95,90	n.a.	BMB*
2	18,53	n.a.	19,457	9,119	4,10	n.a.	MB*
Total:			571,587	222,326	100,00	0,000	

(*S*)-7-Benzyl-2-(trifluoromethyl)-6,7-dihydro-5*H*-pyrrolo[3,4-*b*]pyridin-5-one (3m)

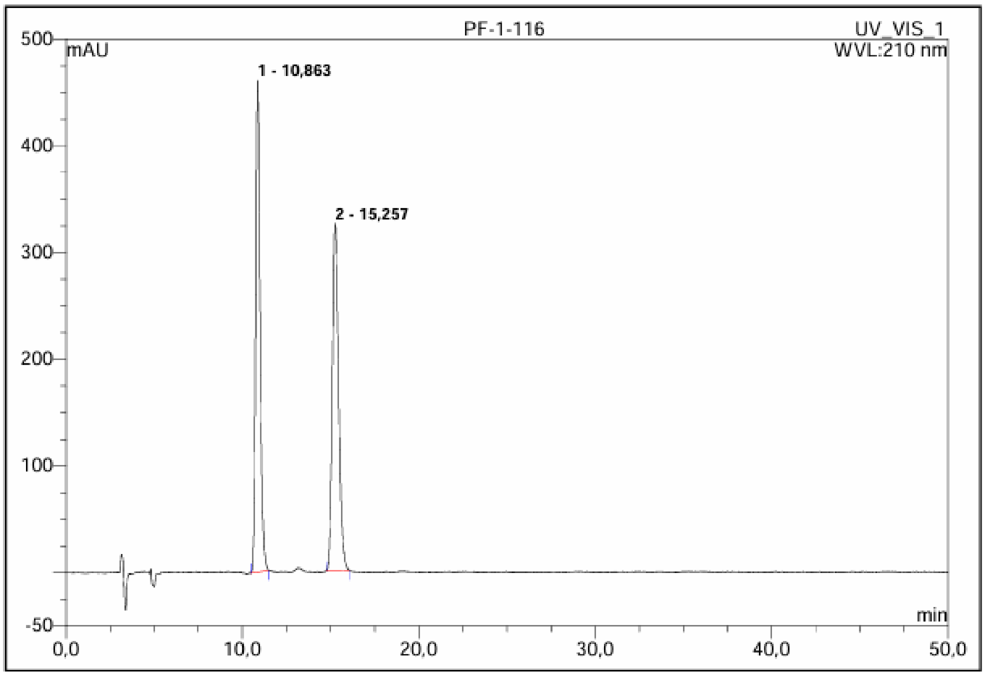

No.	Ret.Time min	Peak Name	Height mAU	Area mAU*min	Rel.Area %	Amount	Type
1	10,86	n.a.	460,557	135,084	50,03	n.a.	BMB
2	15,26	n.a.	325,520	134,920	49,97	n.a.	BMB
Total:			786,077	270,003	100,00	0,000	

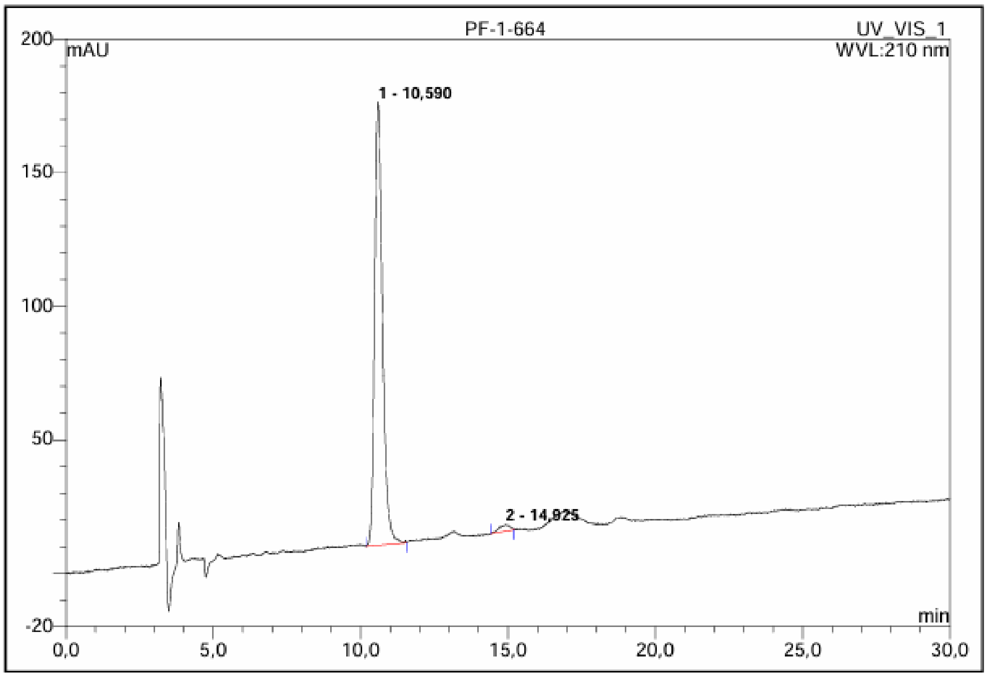

No.	Ret.Time min	Peak Name	Height mAU	Area mAU*min	Rel.Area %	Amount	Type
1	10,59	n.a.	165,867	51,286	98,02	n.a.	BMB*
2	14,92	n.a.	2,396	1,034	1,98	n.a.	BMB*
Total:			168,263	52,321	100,00	0,000	

(*S*)-7-Benzyl-2-chloro-6,7-dihydro-5*H*-pyrrolo[3,4-*b*]pyridin-5-one (3n)

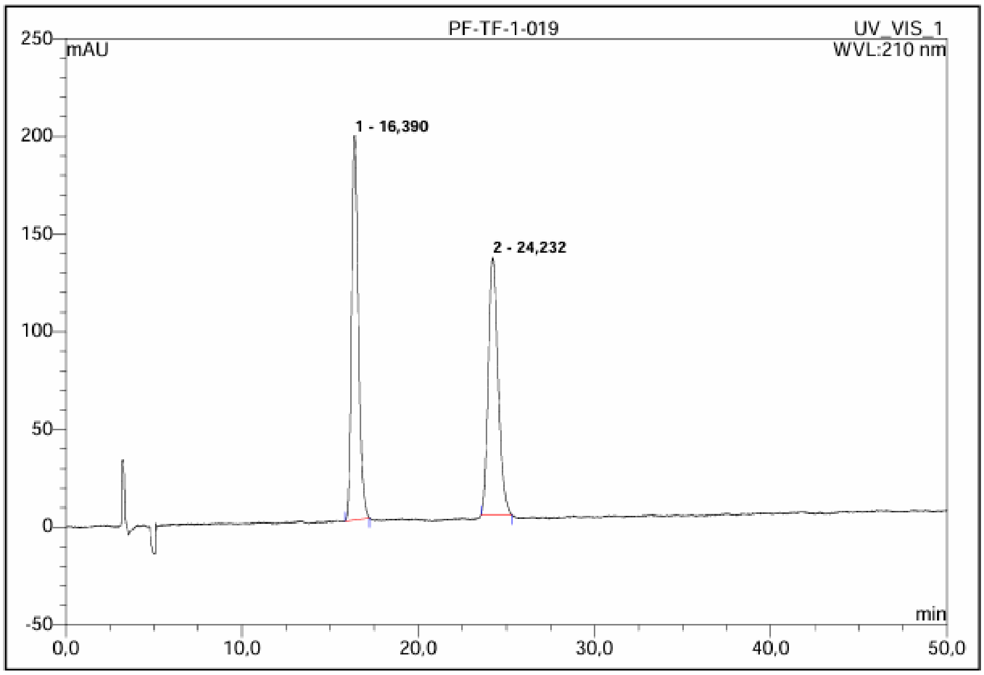

No.	Ret.Time min	Peak Name	Height mAU	Area mAU*min	Rel.Area %	Amount	Type
1	16,39	n.a.	196,825	87,772	50,67	n.a.	BMB
2	24,23	n.a.	131,670	85,434	49,33	n.a.	BMB
Total:			328,495	173,206	100,00	0,000	

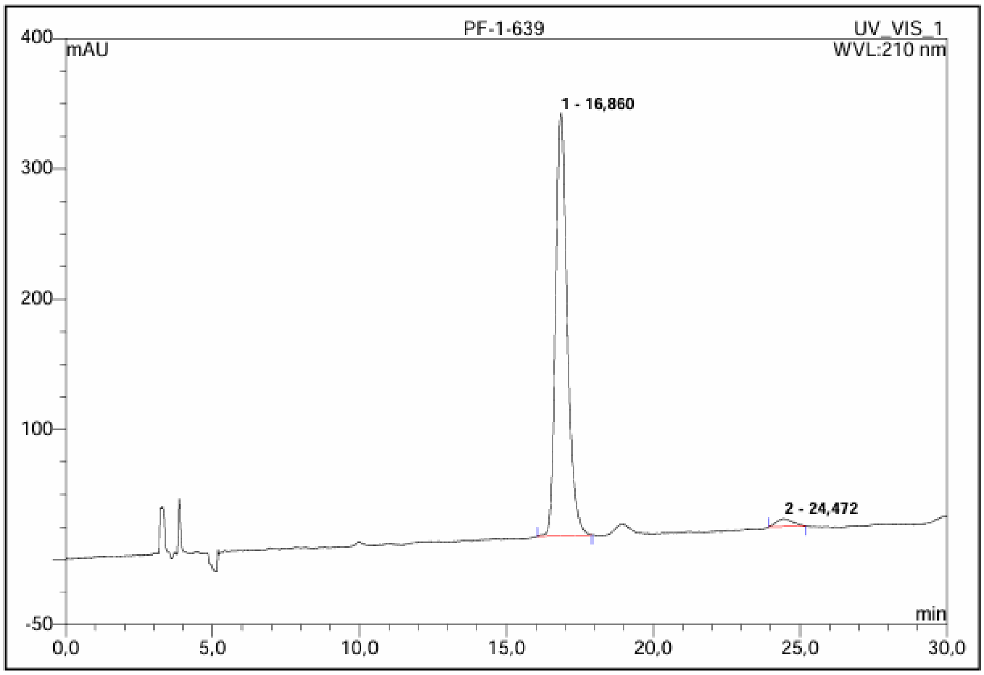

No.	Ret.Time min	Peak Name	Height mAU	Area mAU*min	Rel.Area %	Amount	Type
1	16,86	n.a.	324,443	147,640	97,48	n.a.	BMB*
2	24,47	n.a.	5,964	3,818	2,52	n.a.	BMB*
Total:			330,407	151,458	100,00	0,000	

(*S*)-7-Benzyl-3-bromo-6,7-dihydro-5*H*-pyrrolo[3,4-*b*]pyridin-5-one (3o)

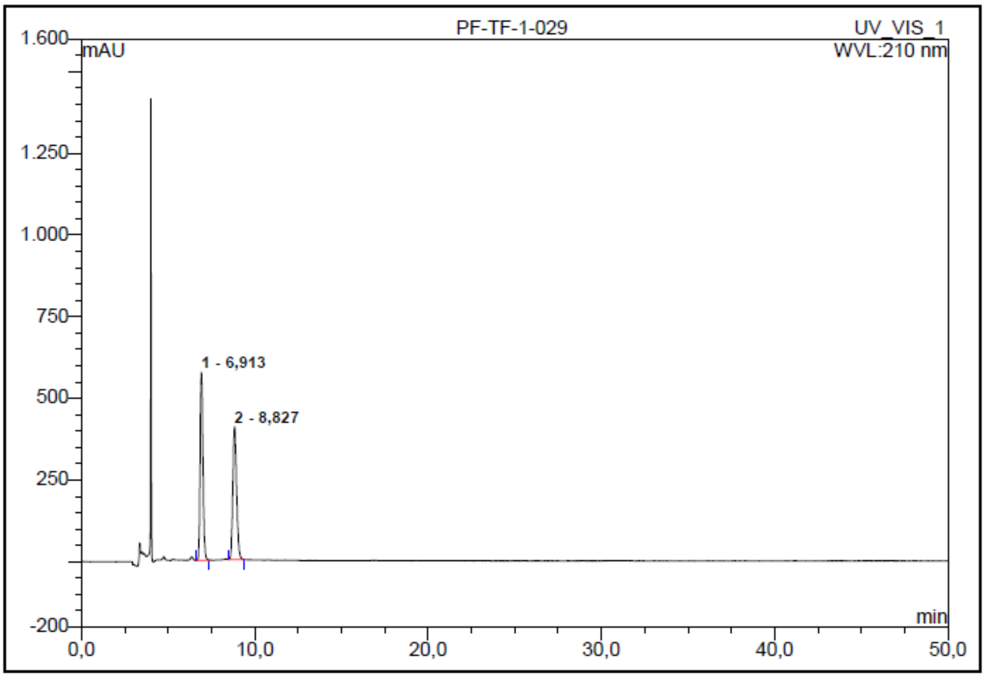

No.	Ret.Time min	Peak Name	Height mAU	Area mAU*min	Rel.Area %	Amount	Type
1	6,91	n.a.	575,889	108,335	51,10	n.a.	BMB
2	8,83	n.a.	406,414	103,683	48,90	n.a.	BMB
Total:			982,303	212,018	100,00	0,000	

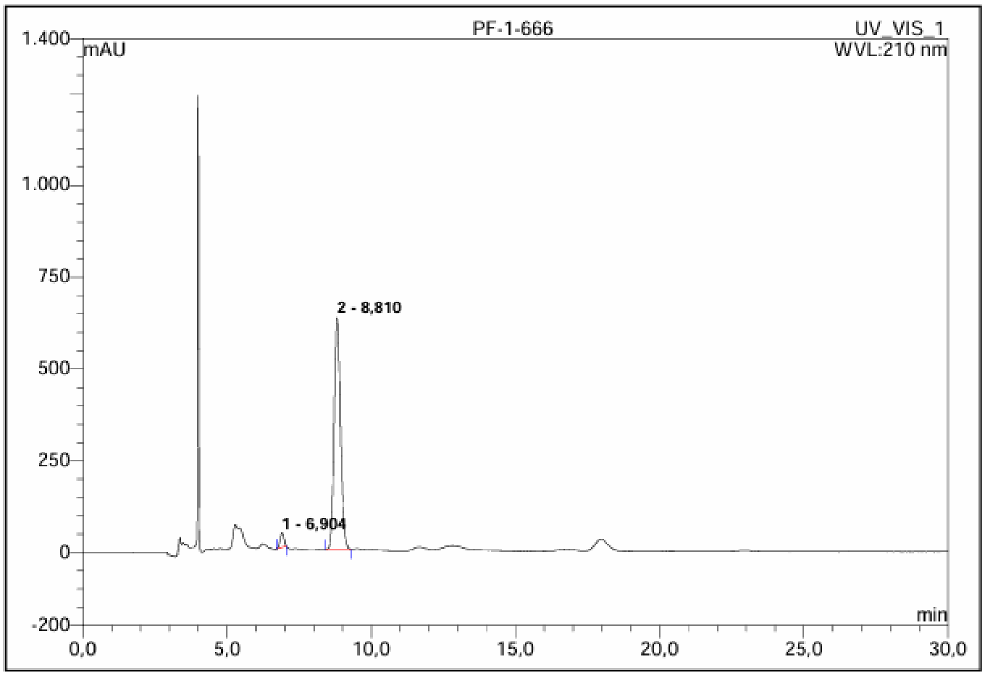

No.	Ret.Time min	Peak Name	Height mAU	Area mAU*min	Rel.Area %	Amount	Type
1	6,90	n.a.	39,235	6,205	3,68	n.a.	BMB*
2	8,81	n.a.	631,475	162,337	96,32	n.a.	BMB
Total:			670,709	168,542	100,00	0,000	

(*S*)-7-Benzyl-3-chloro-6,7-dihydro-5*H*-pyrrolo[3,4-*b*]pyridin-5-one (3p)

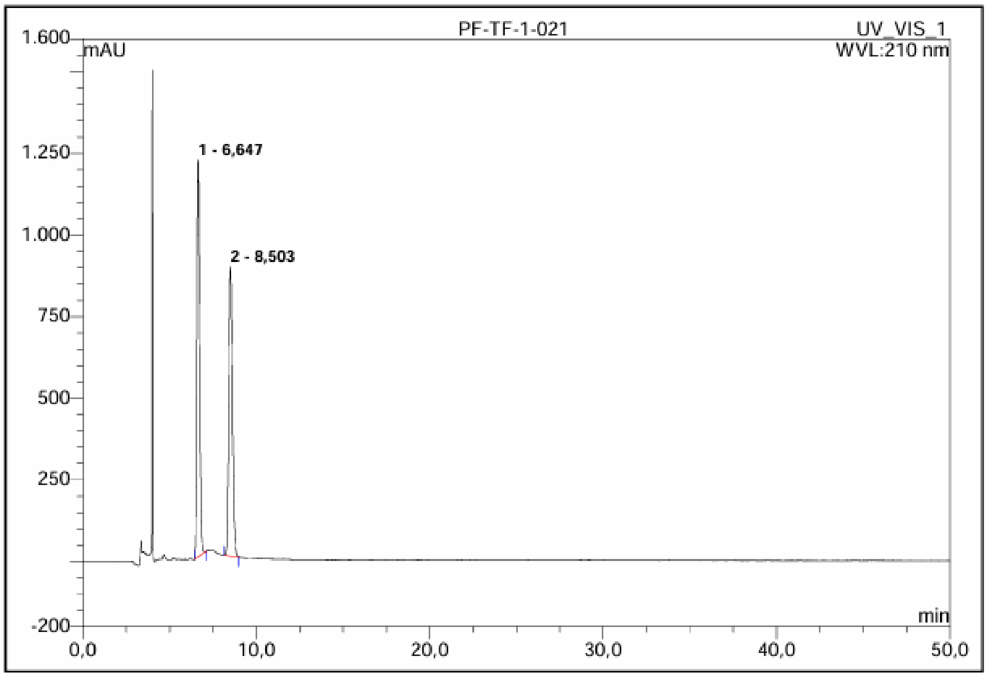

No.	Ret.Time min	Peak Name	Height mAU	Area mAU*min	Rel.Area %	Amount	Type
1	6,65	n.a.	1216,398	213,225	49,87	n.a.	BMB
2	8,50	n.a.	887,009	214,326	50,13	n.a.	BMB
Total:			2103,407	427,551	100,00	0,000	

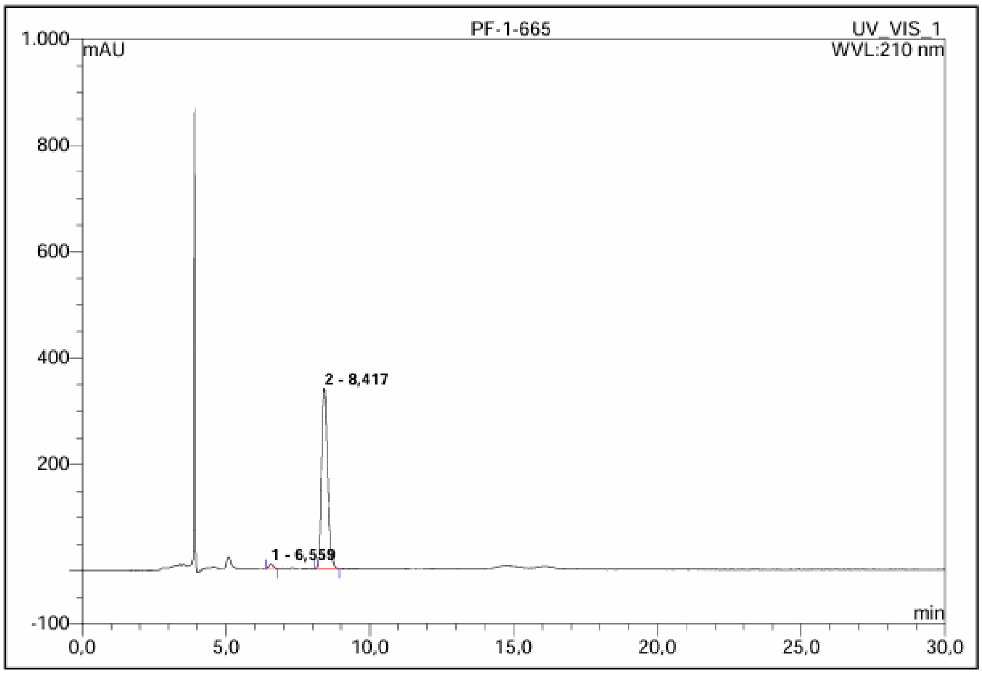

No.	Ret.Time min	Peak Name	Height mAU	Area mAU*min	Rel.Area %	Amount	Type
1	6,56	n.a.	8,776	1,419	1,67	n.a.	BMB*
2	8,42	n.a.	339,620	83,384	98,33	n.a.	BMB
Total:			348,396	84,803	100,00	0,000	

Reaction with 5 mol% of 11a instead of (-)-2b

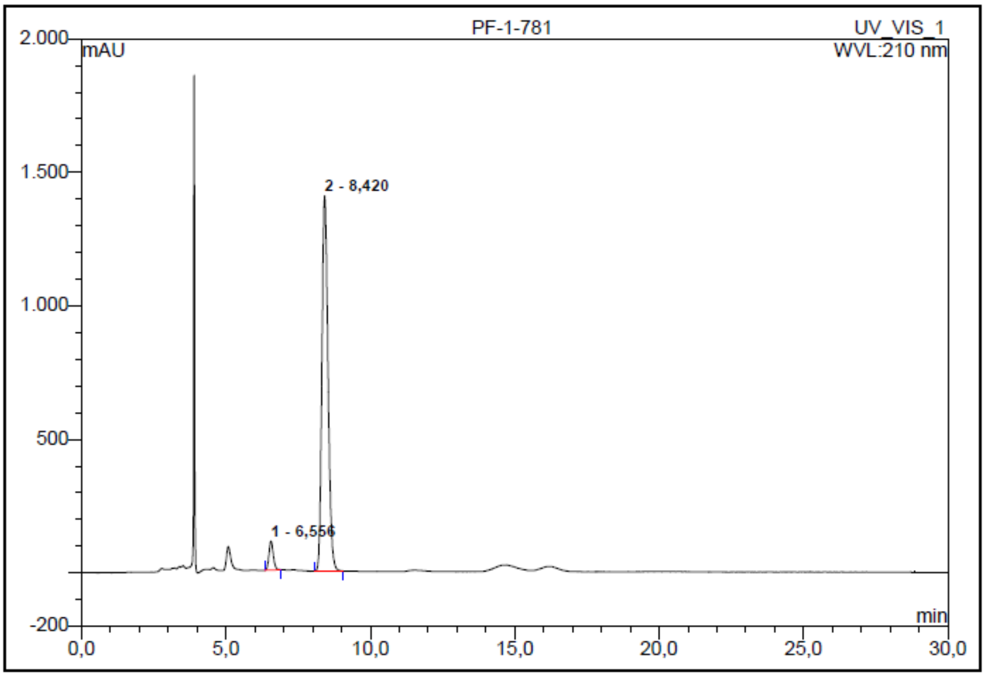

No.	Ret.Time min	Peak Name	Height mAU	Area mAU*min	Rel.Area %	Amount	Type
1	6,56	n.a.	108,361	18,786	5,15	n.a.	BMB
2	8,42	n.a.	1405,620	346,066	94,85	n.a.	BMB
Total:			1513,980	364,852	100,00	0,000	

(*S*)-7-Benzyl-3-phenyl-6,7-dihydro-5*H*-pyrrolo[3,4-*b*]pyridin-5-one (3q)

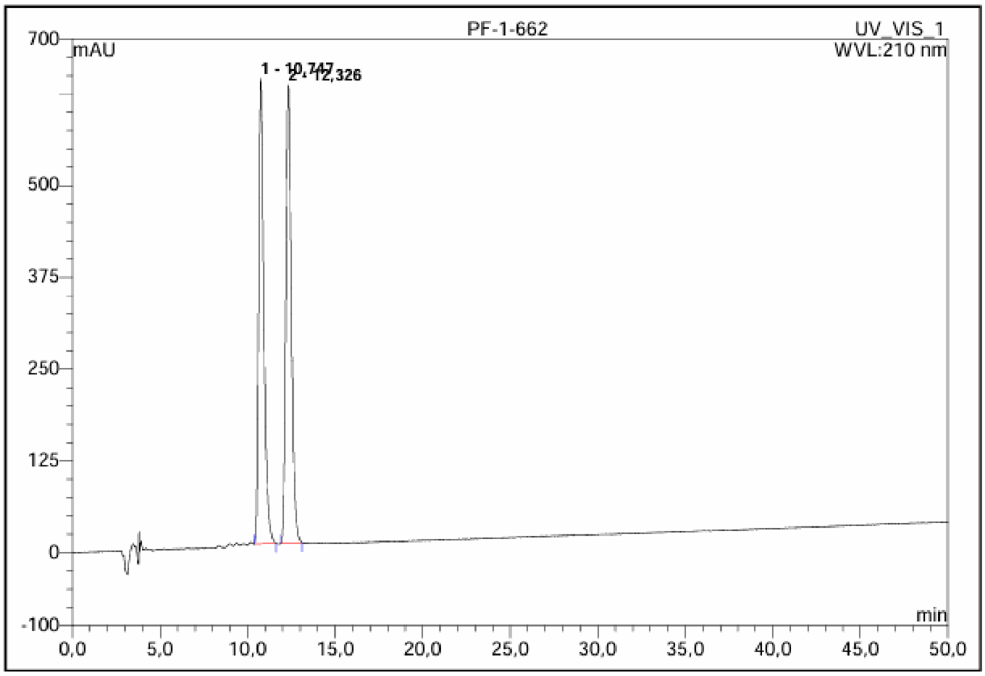

No.	Ret.Time min	Peak Name	Height mAU	Area mAU*min	Rel.Area %	Amount	Type
1	10,75	n.a.	634,466	222,220	49,53	n.a.	BMB
2	12,33	n.a.	624,393	226,449	50,47	n.a.	BMB
Total:			1258,858	448,669	100,00	0,000	

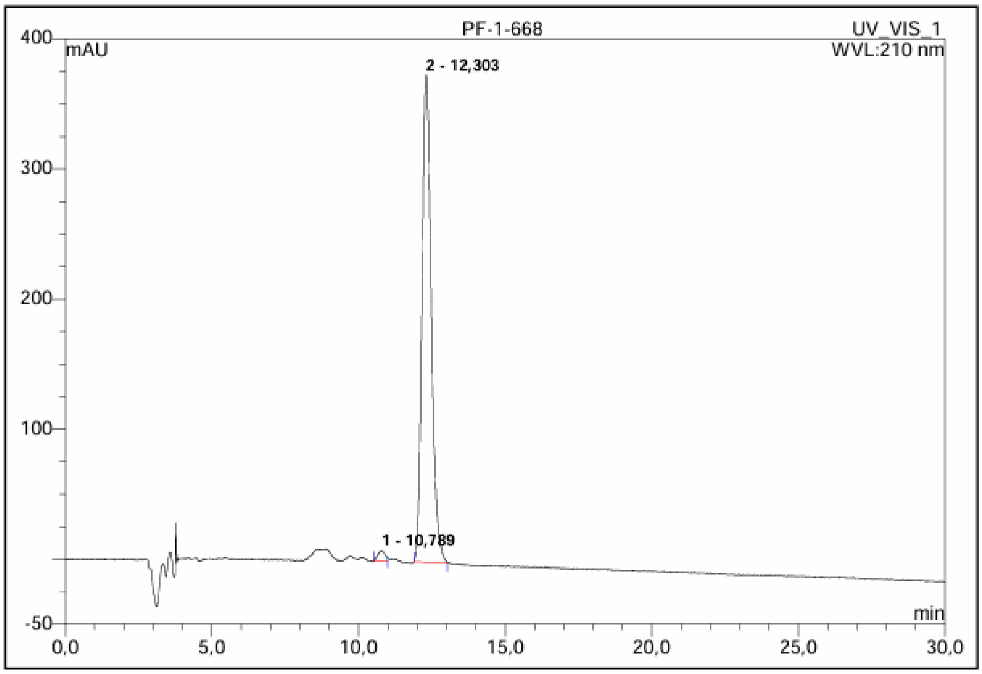

No.	Ret.Time min	Peak Name	Height mAU	Area mAU*min	Rel.Area %	Amount	Type
1	10,79	n.a.	7,759	2,198	1,57	n.a.	BM *
2	12,30	n.a.	374,754	137,769	98,43	n.a.	BMB
Total:			382,514	139,967	100,00	0,000	

(*S*)-7-Isopropoxy-6,7-dihydro-5*H*-pyrrolo[3,4-*b*]pyridin-5-one (3r)

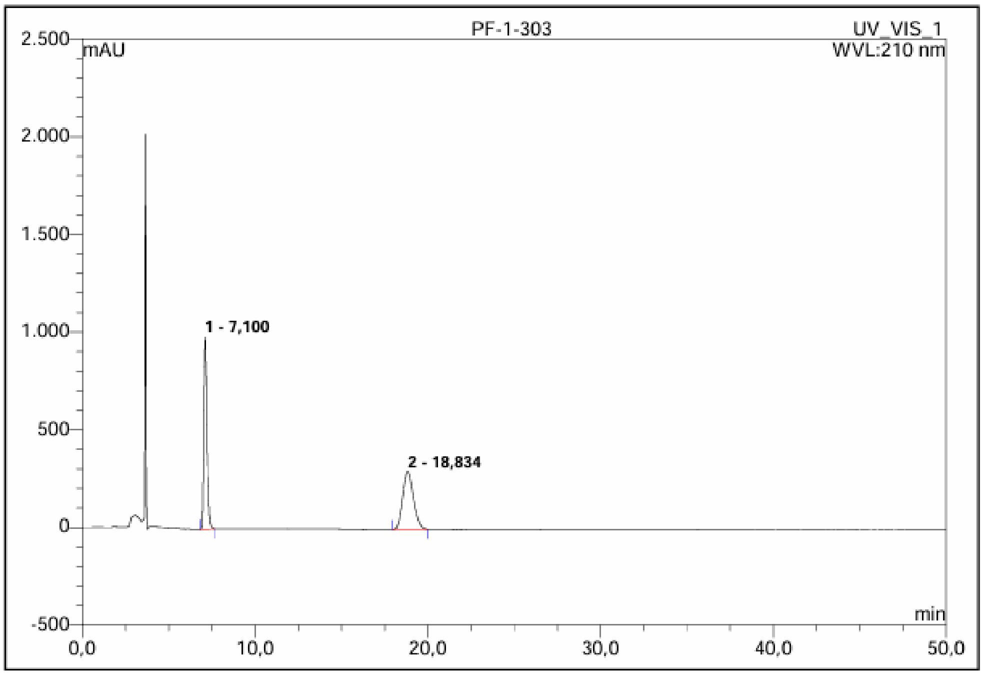

No.	Ret.Time min	Peak Name	Height mAU	Area mAU*min	Rel.Area %	Amount	Type
1	7,10	n.a.	982,527	213,369	49,99	n.a.	BMB
2	18,83	n.a.	296,995	213,446	50,01	n.a.	BMB
Total:			1279,522	426,815	100,00	0,000	

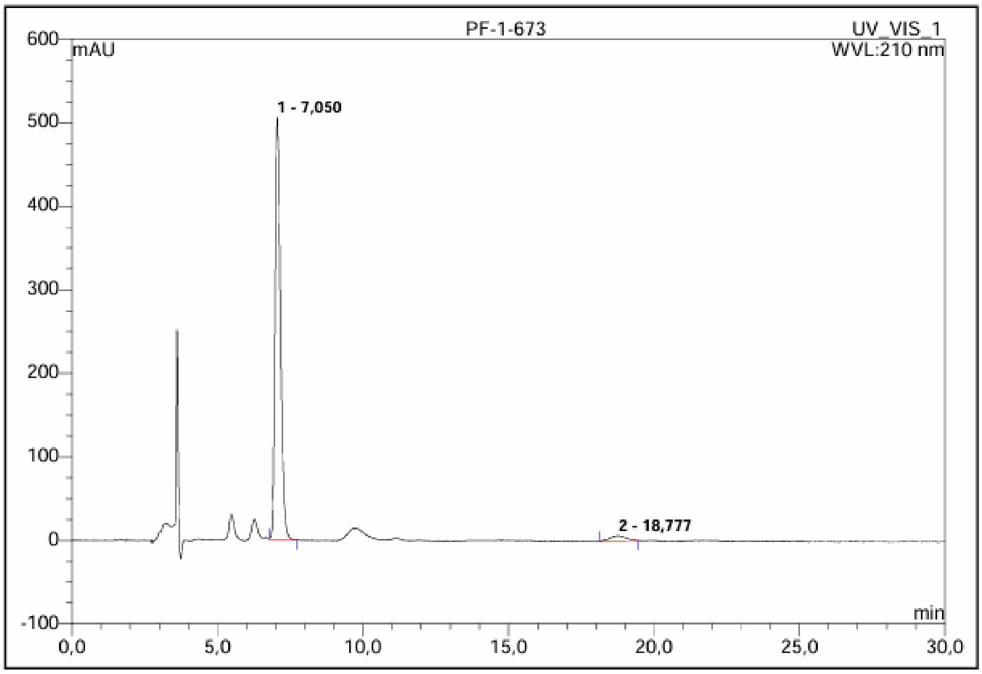

No.	Ret.Time min	Peak Name	Height mAU	Area mAU*min	Rel.Area %	Amount	Type
1	7,05	n.a.	506,159	111,487	96,83	n.a.	MB*
2	18,78	n.a.	6,025	3,653	3,17	n.a.	BMB*
Total:			512,184	115,141	100,00	0,000	

(*S*)-7-(Cyclopentyloxy)-6,7-dihydro-5*H*-pyrrolo[3,4-*b*]pyridin-5-one (3s)

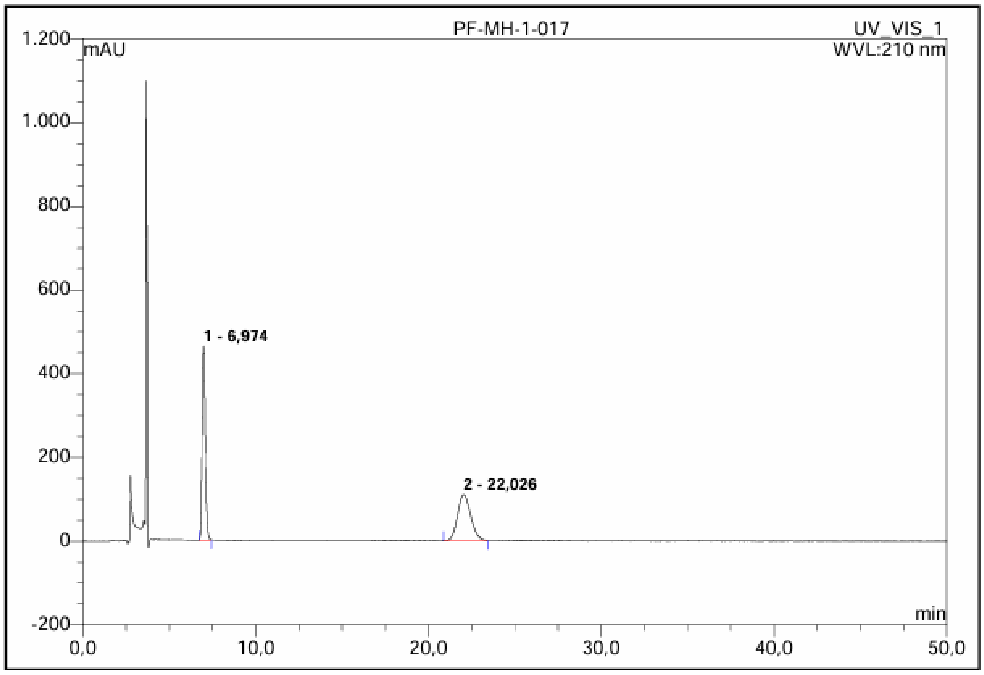

No.	Ret.Time min	Peak Name	Height mAU	Area mAU*min	Rel.Area %	Amount	Type
1	6,97	n.a.	462,715	95,759	49,82	n.a.	BMB
2	22,03	n.a.	110,297	96,434	50,18	n.a.	BMB*
Total:			573,011	192,192	100,00	0,000	

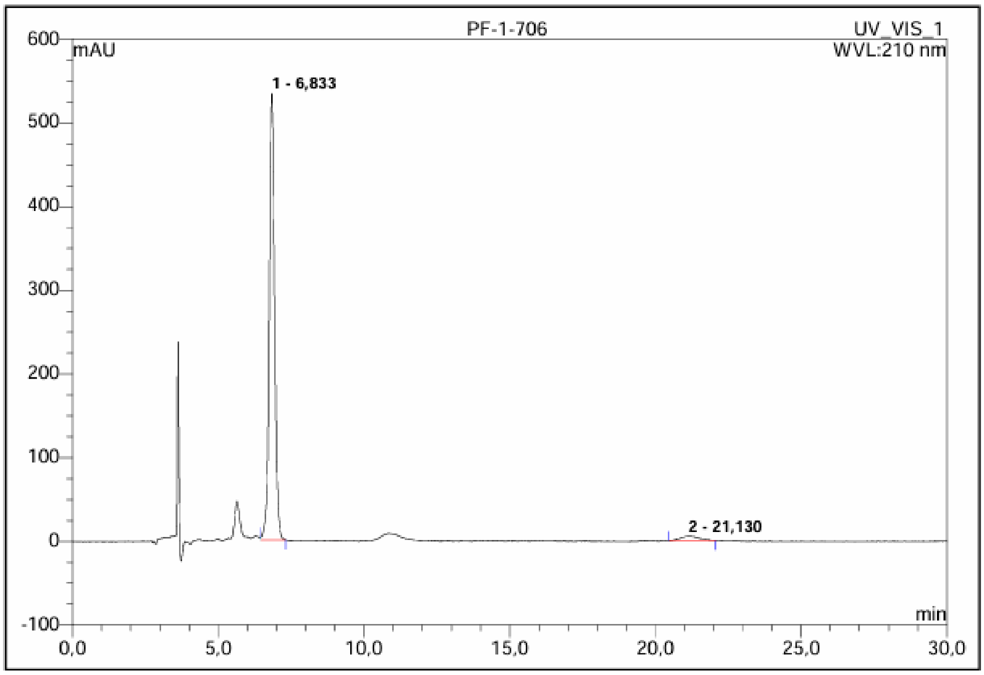

No.	Ret.Time min	Peak Name	Height mAU	Area mAU*min	Rel.Area %	Amount	Type
1	6,83	n.a.	533,308	115,558	96,83	n.a.	MB*
2	21,13	n.a.	5,112	3,778	3,17	n.a.	BMB*
Total:			538,420	119,335	100,00	0,000	

(*S*)-7-(*tert*-butoxy)-6,7-dihydro-5*H*-pyrrolo[3,4-*b*]pyridin-5-one (3t)

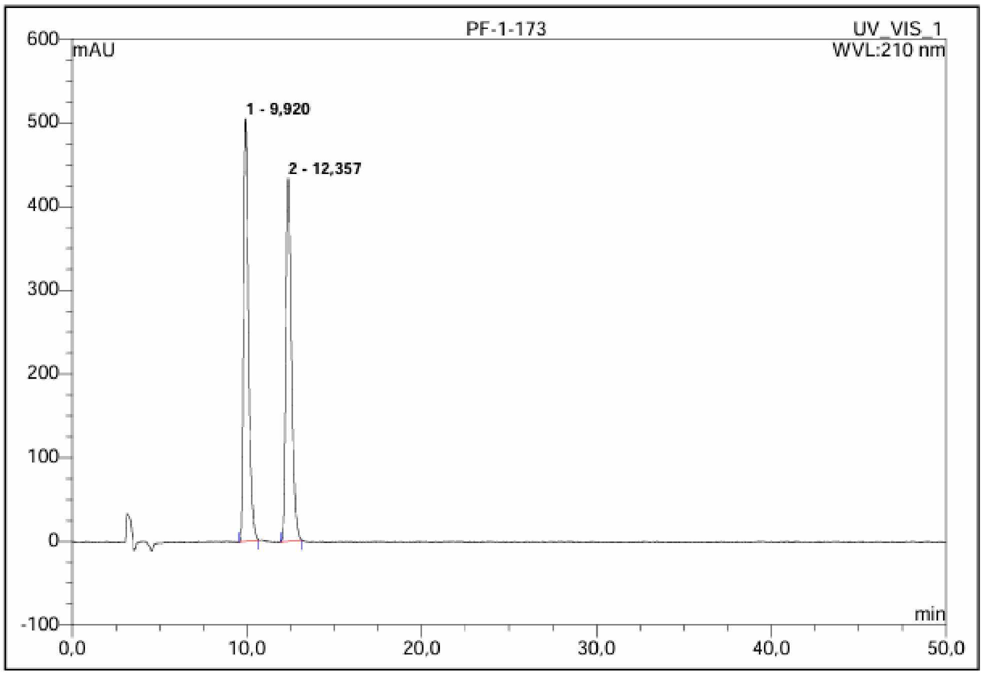

No.	Ret.Time min	Peak Name	Height mAU	Area mAU*min	Rel.Area %	Amount	Type
1	9,92	n.a.	504,622	161,889	50,03	n.a.	BMB
2	12,36	n.a.	434,031	161,685	49,97	n.a.	BMB
Total:			938,653	323,574	100,00	0,000	

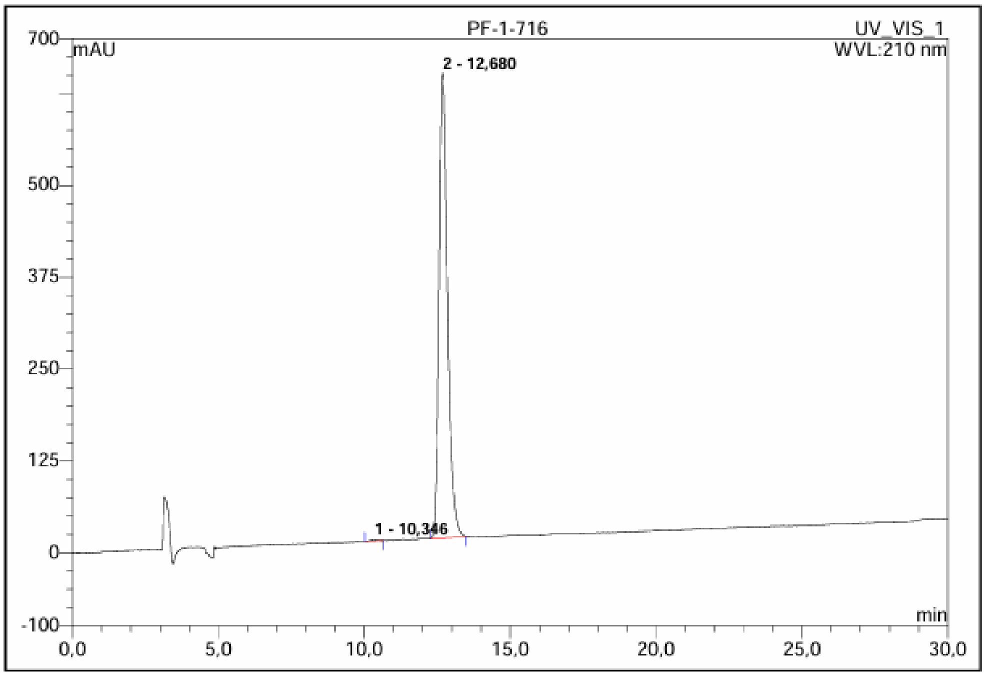

No.	Ret.Time min	Peak Name	Height mAU	Area mAU*min	Rel.Area %	Amount	Type
1	10,35	n.a.	2,190	0,556	0,26	n.a.	BMB*
2	12,68	n.a.	633,081	213,934	99,74	n.a.	BMB
Total:			635,271	214,489	100,00	0,000	

(*S*)-7-(3-Chloro-2,2-dimethylpropoxy)-6,7-dihydro-5*H*-pyrrolo[3,4-*b*]pyridin-5-one (3u)

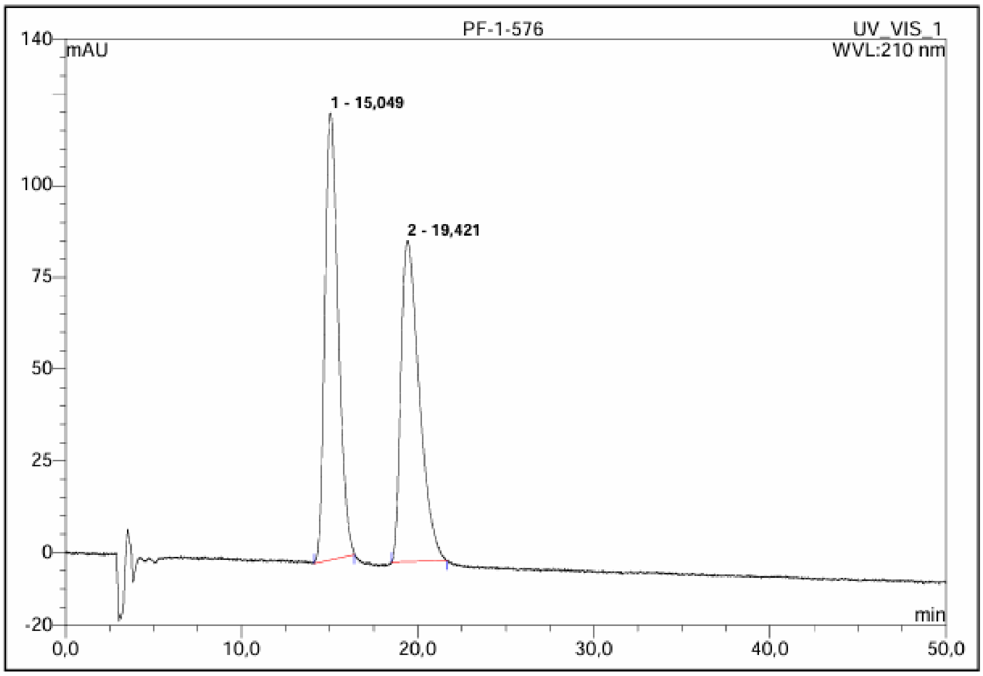

No.	Ret.Time min	Peak Name	Height mAU	Area mAU*min	Rel.Area %	Amount	Type
1	15,05	n.a.	121,934	107,375	50,46	n.a.	BMB*
2	19,42	n.a.	87,536	105,418	49,54	n.a.	BMB*
Total:			209,471	212,793	100,00	0,000	

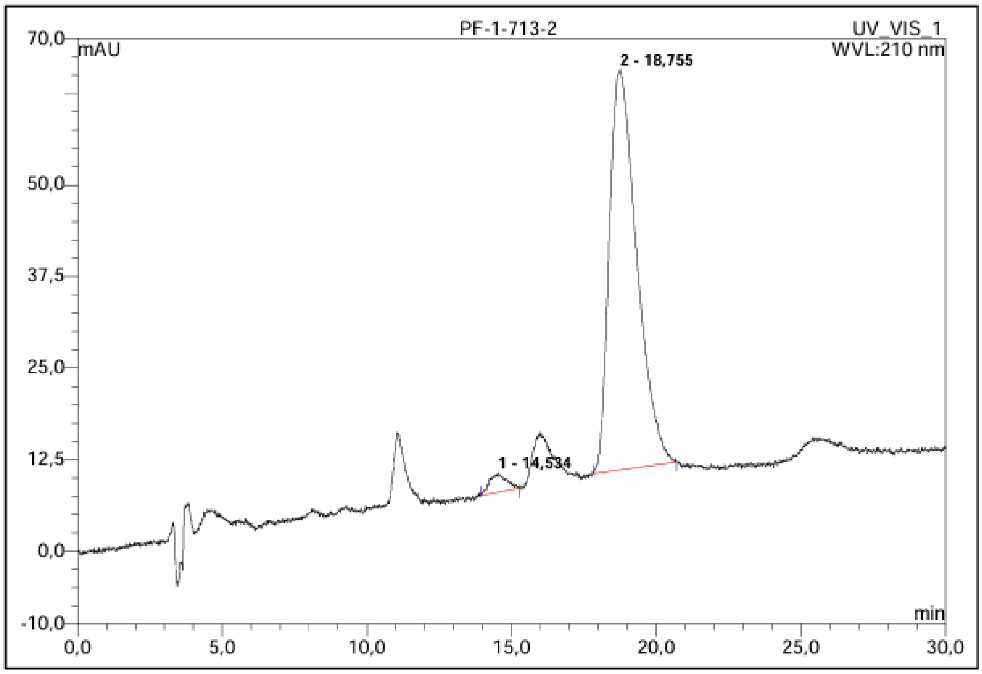

No.	Ret.Time min	Peak Name	Height mAU	Area mAU*min	Rel.Area %	Amount	Type
1	14,53	n.a.	2,652	1,767	2,86	n.a.	BMB*
2	18,76	n.a.	54,684	60,053	97,14	n.a.	BMB*
Total:			57,336	61,820	100,00	0,000	

(*S*)-7-((*tert*-Butyldimethylsilyl)oxy)-6,7-dihydro-5*H*-pyrrolo[3,4-*b*]pyridin-5-one (3v)

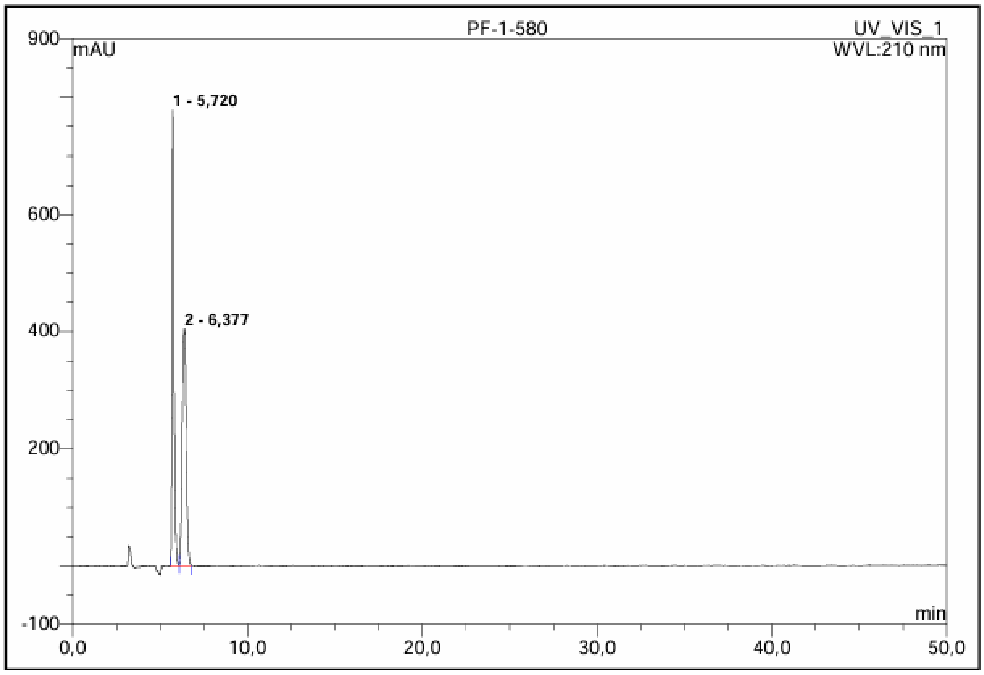

No.	Ret.Time min	Peak Name	Height mAU	Area mAU*min	Rel.Area %	Amount	Type
1	5,72	n.a.	778,042	106,105	49,85	n.a.	BM
2	6,38	n.a.	404,087	106,760	50,15	n.a.	MB
Total:			1182,128	212,865	100,00	0,000	

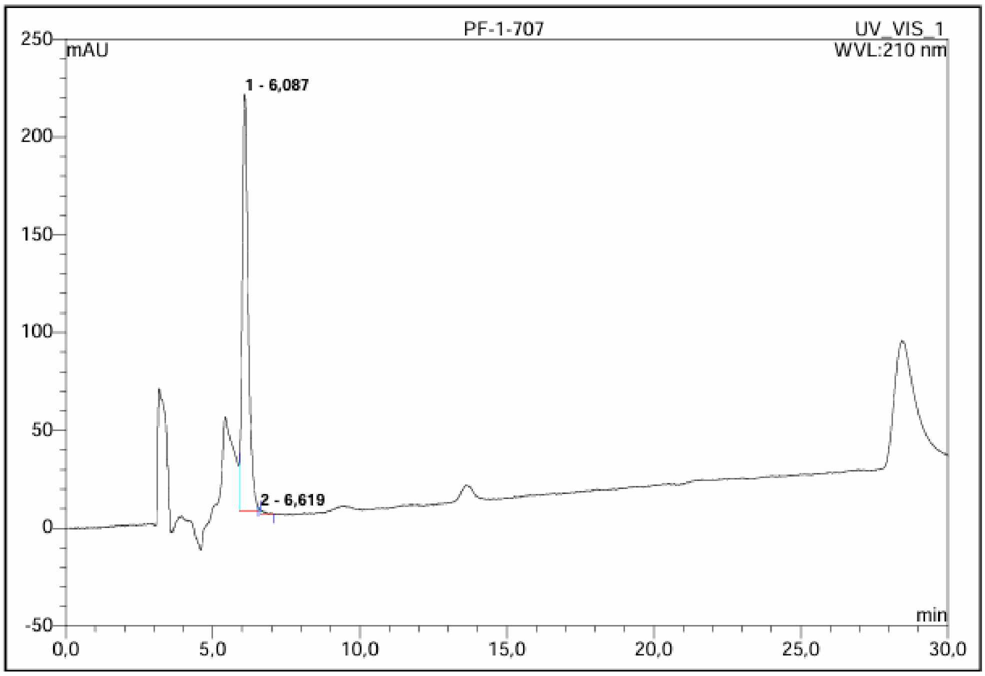

No.	Ret.Time min	Peak Name	Height mAU	Area mAU*min	Rel.Area %	Amount	Type
1	6,09	n.a.	212,703	49,934	99,30	n.a.	M *
2	6,62	n.a.	1,932	0,354	0,70	n.a.	MB*
Total:			214,635	50,288	100,00	0,000	

(*S*)-7-((*S*)-2-methylbutoxy)-6,7-dihydro-5*H*-pyrrolo[3,4-*b*]pyridin-5-one [(3*S*)-3w]

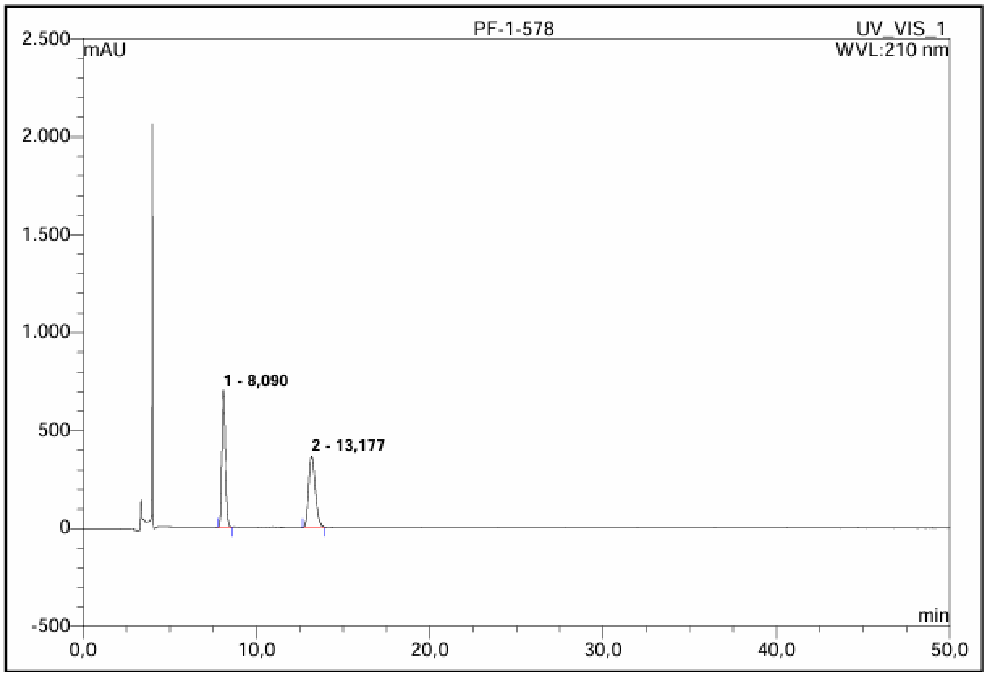

No.	Ret.Time min	Peak Name	Height mAU	Area mAU*min	Rel.Area %	Amount	Type
1	8,09	n.a.	702,120	163,481	50,20	n.a.	BMB
2	13,18	n.a.	365,894	162,148	49,80	n.a.	BMB
Total:			1068,014	325,629	100,00	0,000	

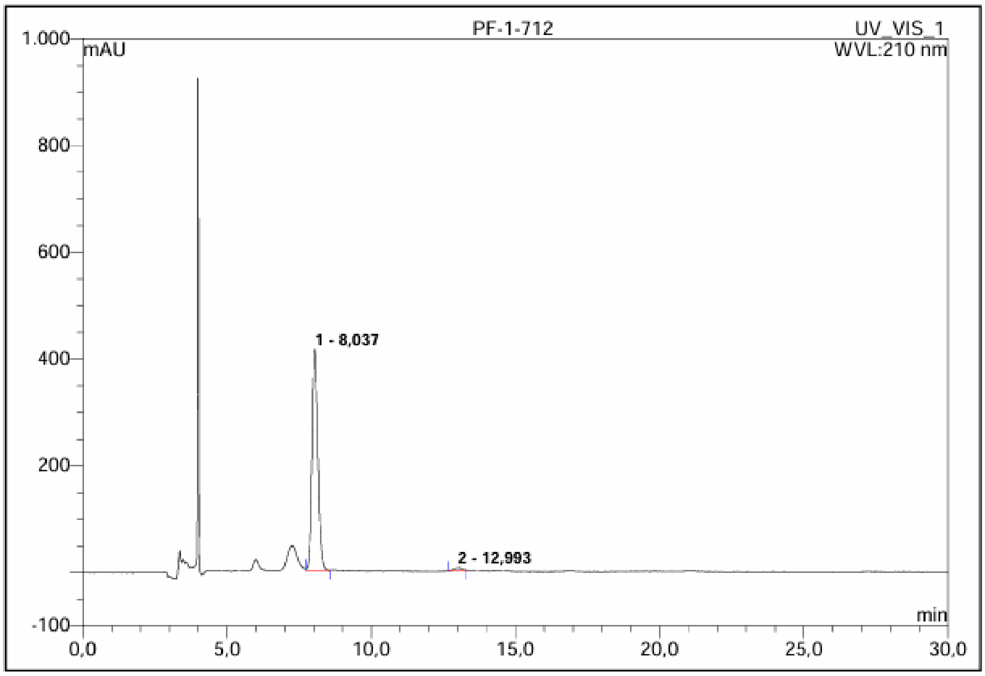

No.	Ret.Time min	Peak Name	Height mAU	Area mAU*min	Rel.Area %	Amount	Type
1	8,04	n.a.	413,401	96,145	97,96	n.a.	MB*
2	12,99	n.a.	5,541	2,000	2,04	n.a.	BM *
Total:			418,941	98,144	100,00	0,000	

(*R*)-7-((*S*)-2-methylbutoxy)-6,7-dihydro-5*H*-pyrrolo[3,4-*b*]pyridin-5-one [(*3R*)-3w]

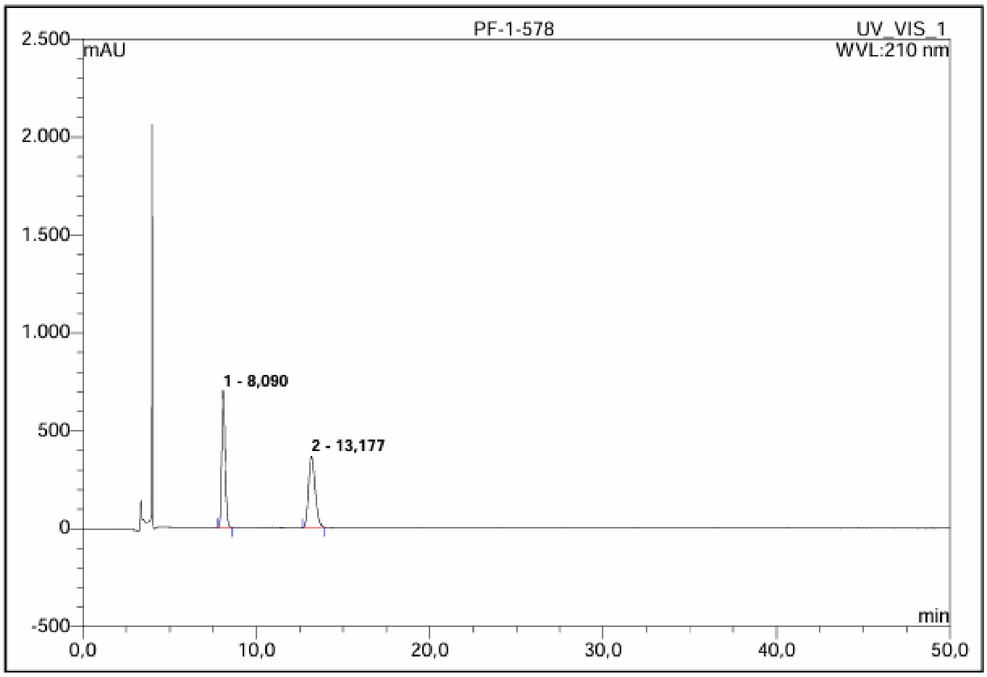

No.	Ret.Time min	Peak Name	Height mAU	Area mAU*min	Rel.Area %	Amount	Type
1	8,09	n.a.	702,120	163,481	50,20	n.a.	BMB
2	13,18	n.a.	365,894	162,148	49,80	n.a.	BMB
Total:			1068,014	325,629	100,00	0,000	

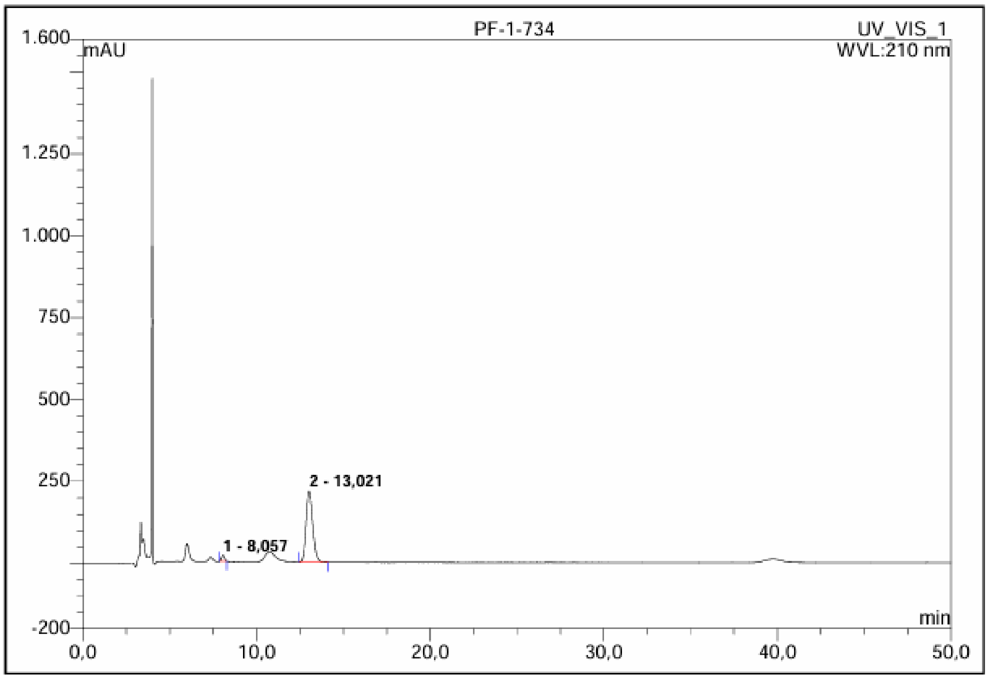

No.	Ret.Time min	Peak Name	Height mAU	Area mAU*min	Rel.Area %	Amount	Type
1	8,06	n.a.	17,088	3,452	3,53	n.a.	MB*
2	13,02	n.a.	215,673	94,211	96,47	n.a.	BMB*
Total:			232,761	97,663	100,00	0,000	

3-Benzyl-2,3-dihydro-1*H*-pyrrolo[3,4-*c*]pyridin-1-one (*rac*-**12a**)

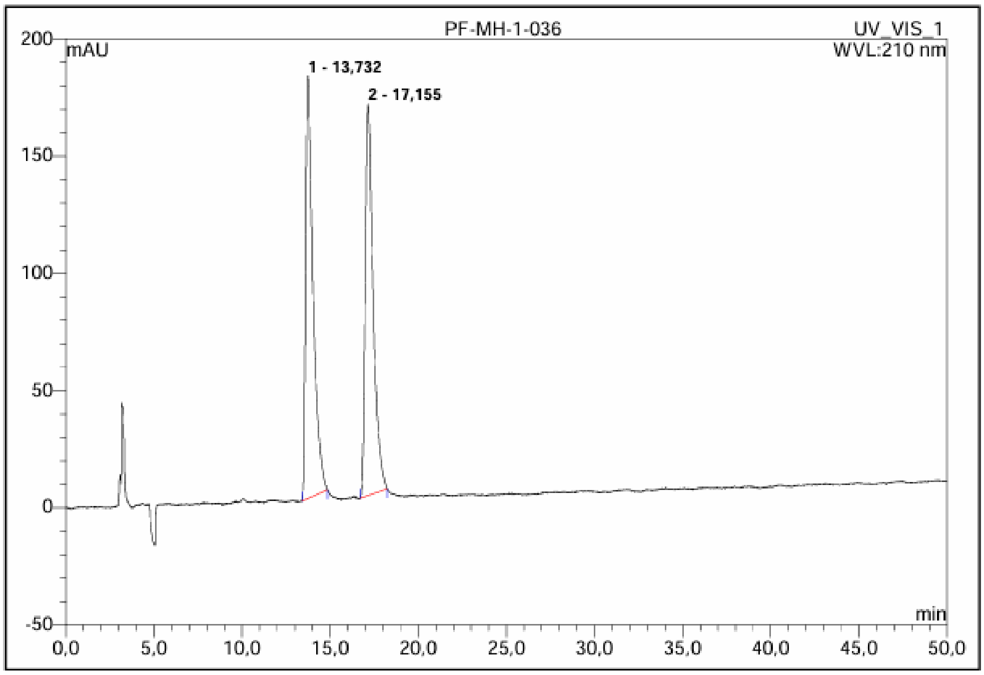

No.	Ret.Time min	Peak Name	Height mAU	Area mAU*min	Rel.Area %	Amount	Type
1	13,73	n.a.	180,222	87,580	50,07	n.a.	BMB
2	17,16	n.a.	166,873	87,328	49,93	n.a.	BMB
Total:			347,096	174,908	100,00	0,000	

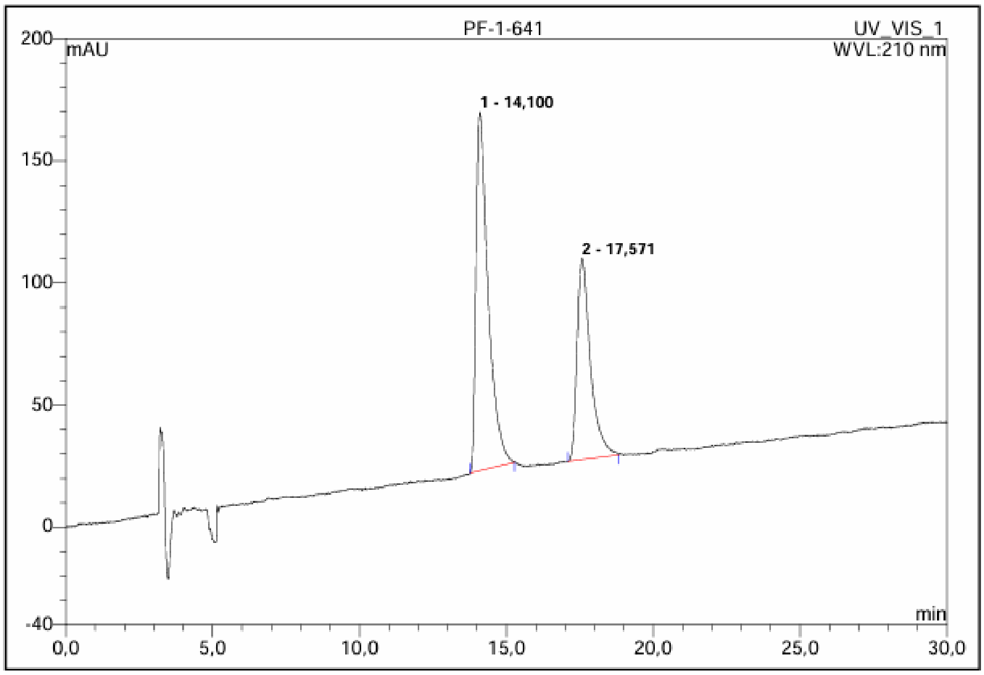

No.	Ret.Time min	Peak Name	Height mAU	Area mAU*min	Rel.Area %	Amount	Type
1	14,10	n.a.	146,648	69,988	61,68	n.a.	BMB
2	17,57	n.a.	82,386	43,477	38,32	n.a.	BMB
Total:			229,034	113,464	100,00	0,000	

1-Benzyl-1,2-dihydro-3*H*-pyrrolo[3,4-*c*]pyridin-3-one (*rac*-**12b**)

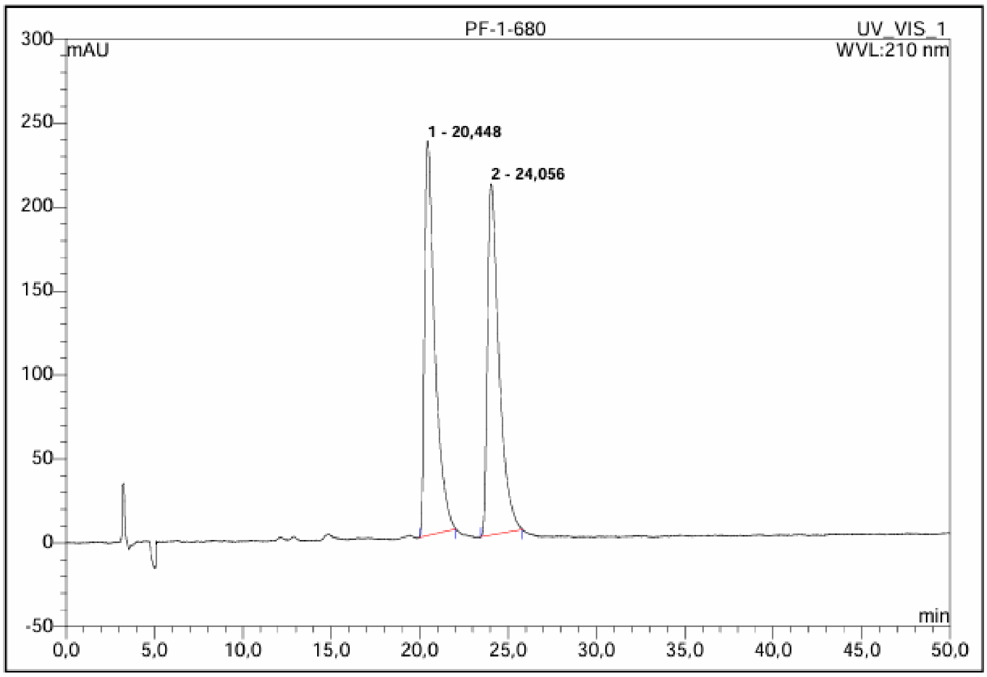

No.	Ret.Time min	Peak Name	Height mAU	Area mAU*min	Rel.Area %	Amount	Type
1	20,45	n.a.	234,997	159,319	50,06	n.a.	BMB
2	24,06	n.a.	208,907	158,948	49,94	n.a.	BMB
Total:			443,904	318,267	100,00	0,000	

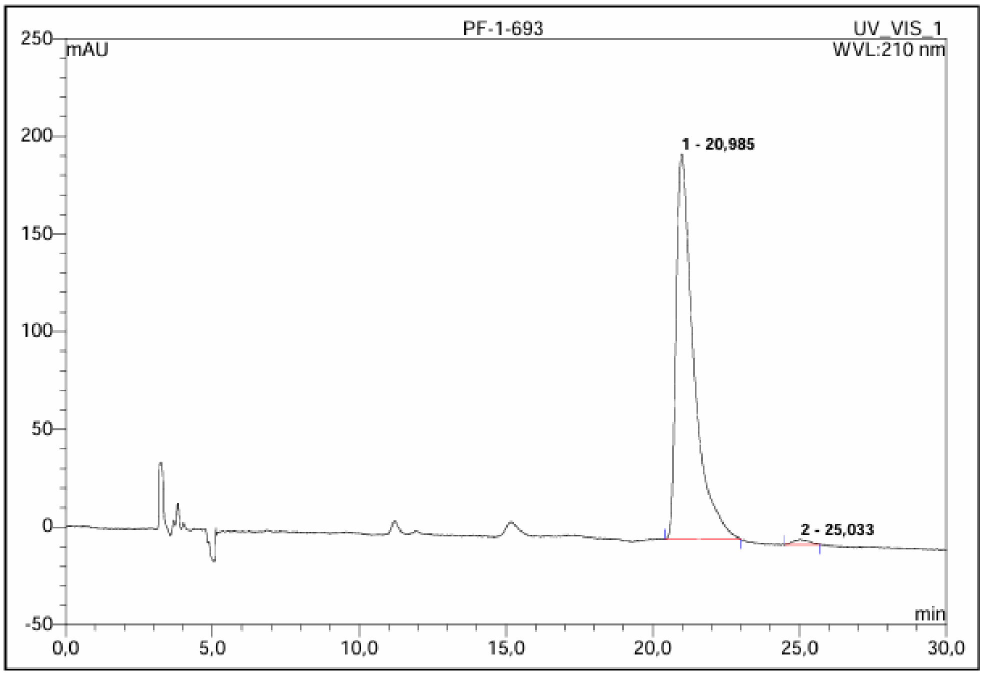

No.	Ret.Time min	Peak Name	Height mAU	Area mAU*min	Rel.Area %	Amount	Type
1	20,98	n.a.	196,929	137,597	98,86	n.a.	BMB*
2	25,03	n.a.	2,766	1,585	1,14	n.a.	BMB*
Total:			199,694	139,182	100,00	0,000	

5-Benzyl-5,6-dihydro-7*H*-pyrrolo[3,4-*b*]pyridin-7-one (*rac*-**12c**)

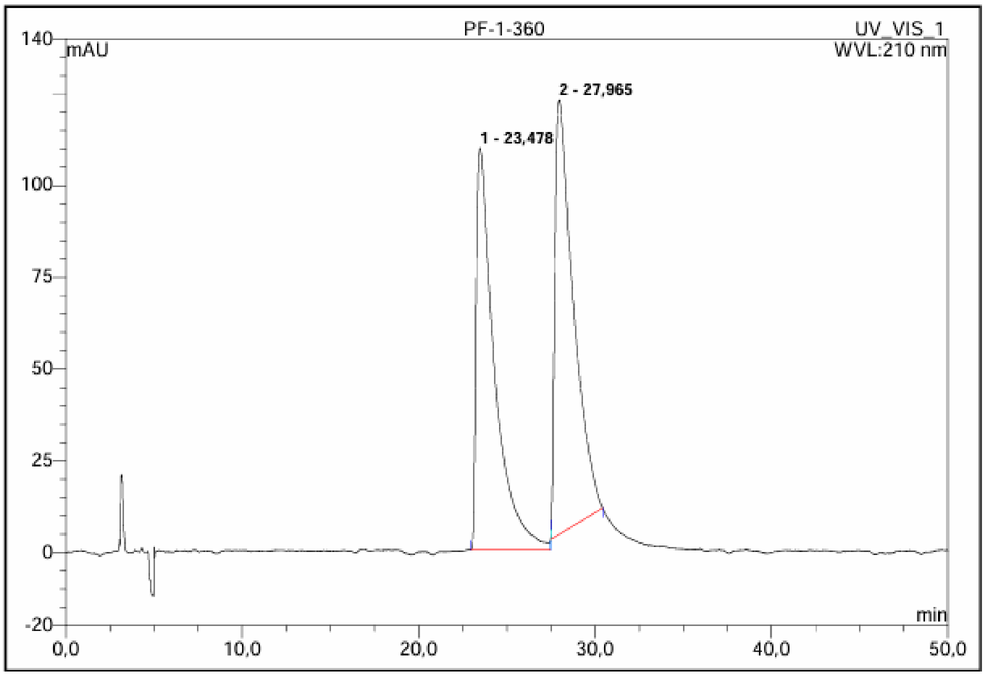

No.	Ret.Time min	Peak Name	Height mAU	Area mAU*min	Rel.Area %	Amount	Type
1	23,48	n.a.	109,552	131,836	47,90	n.a.	BM *
2	27,96	n.a.	118,426	143,397	52,10	n.a.	MB*
Total:			227,978	275,233	100,00	0,000	

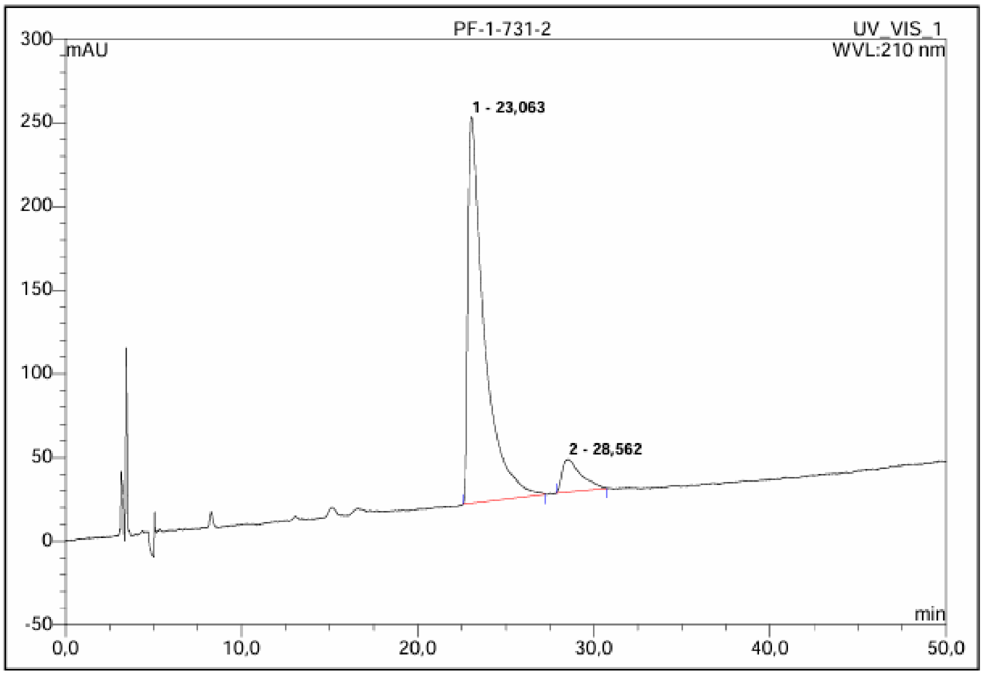

No.	Ret.Time min	Peak Name	Height mAU	Area mAU*min	Rel.Area %	Amount	Type
1	23,06	n.a.	230,792	249,457	91,11	n.a.	BMB*
2	28,56	n.a.	19,448	24,336	8,89	n.a.	BMB*
Total:			250,240	273,793	100,00	0,000	

(*S*)-*tert*-Butyl 7-benzyl-5-oxo-5,7-dihydro-6*H*-pyrrolo[3,4-*b*]pyridine-6-carboxylate (4)

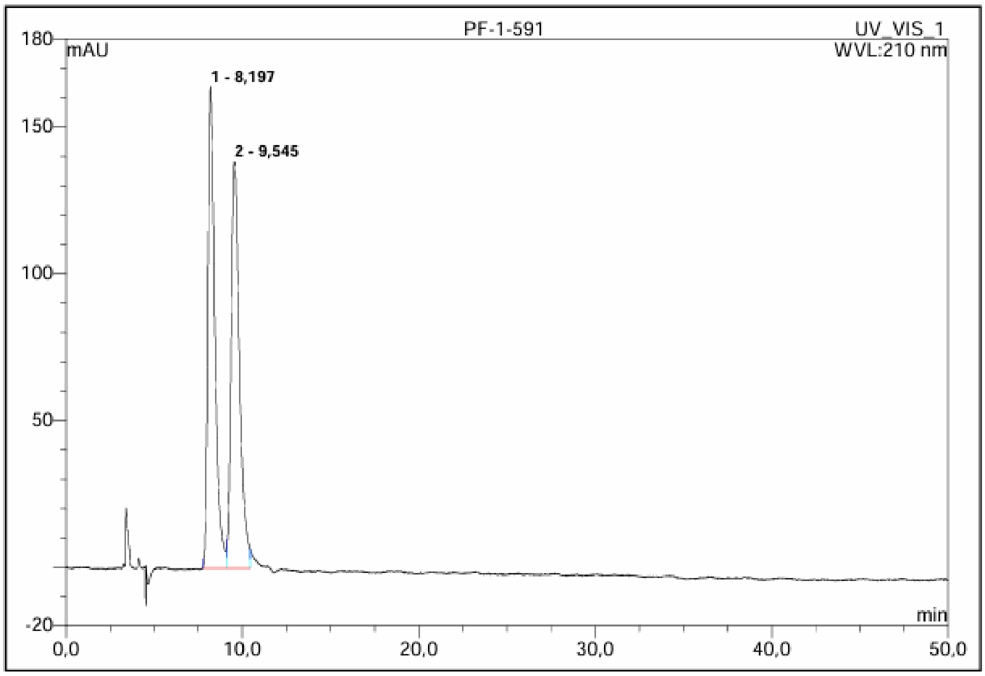

No.	Ret.Time min	Peak Name	Height mAU	Area mAU*min	Rel.Area %	Amount	Type
1	8,20	n.a.	164,089	75,455	49,65	n.a.	BM *
2	9,54	n.a.	138,614	76,534	50,35	n.a.	M *
Total:			302,704	151,989	100,00	0,000	

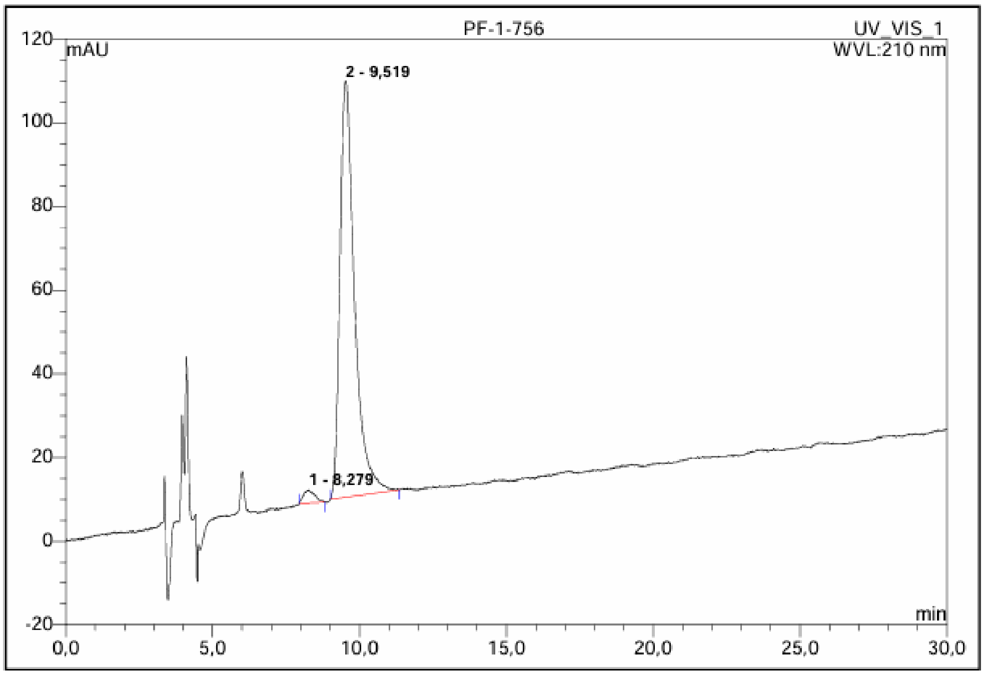

No.	Ret.Time min	Peak Name	Height mAU	Area mAU*min	Rel.Area %	Amount	Type
1	8,28	n.a.	3,109	1,373	2,35	n.a.	BMB*
2	9,52	n.a.	99,467	57,150	97,65	n.a.	BMB*
Total:			102,576	58,523	100,00	0,000	

(*S*)-*tert*-Butyl (*S*)-(1-(3-(hydroxymethyl)pyridin-2-yl)-2-phenylethyl)carbamate (6)

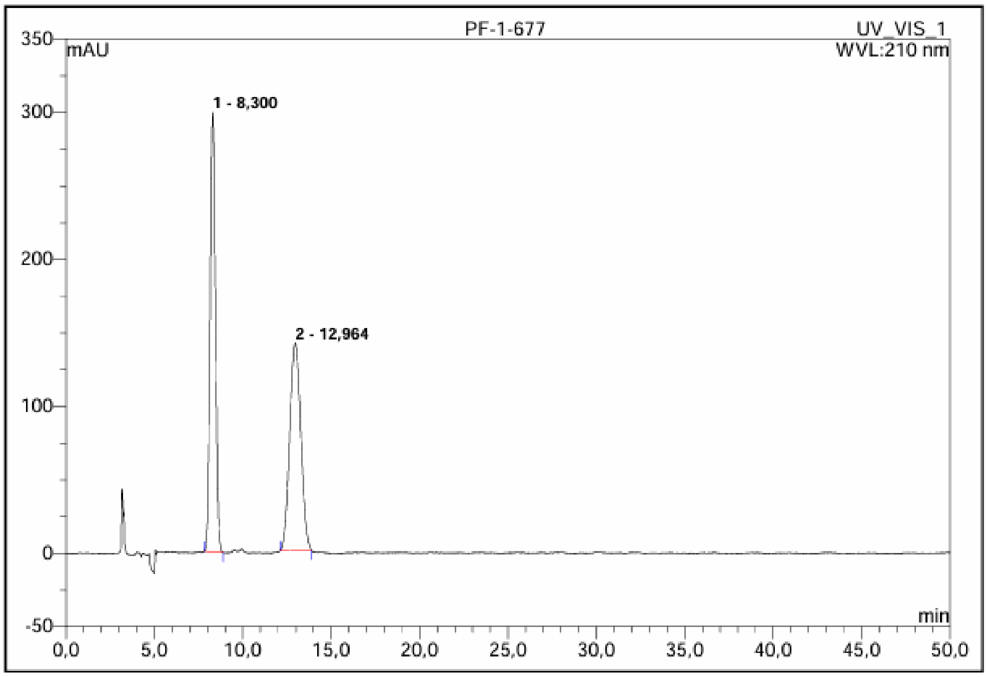

No.	Ret.Time min	Peak Name	Height mAU	Area mAU*min	Rel.Area %	Amount	Type
1	8,30	n.a.	299,071	104,846	50,53	n.a.	BMB
2	12,96	n.a.	141,037	102,633	49,47	n.a.	BMB
Total:			440,108	207,479	100,00	0,000	

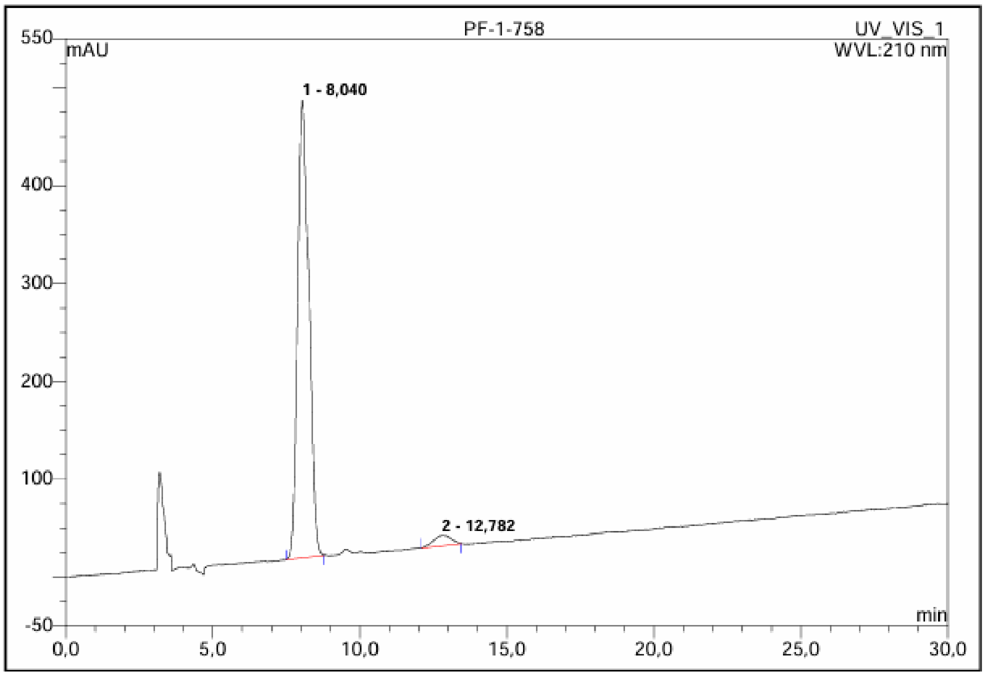

No.	Ret.Time min	Peak Name	Height mAU	Area mAU*min	Rel.Area %	Amount	Type
1	8,04	n.a.	467,657	208,694	96,57	n.a.	BMB
2	12,78	n.a.	10,677	7,417	3,43	n.a.	BMB*
Total:			478,334	216,111	100,00	0,000	

(4a*R*,7*S*,7a*S*)-7-(Cyclobutylmethyl)octahydro-5*H*-pyrrolo[3,4-*b*]pyridin-5-one (7)

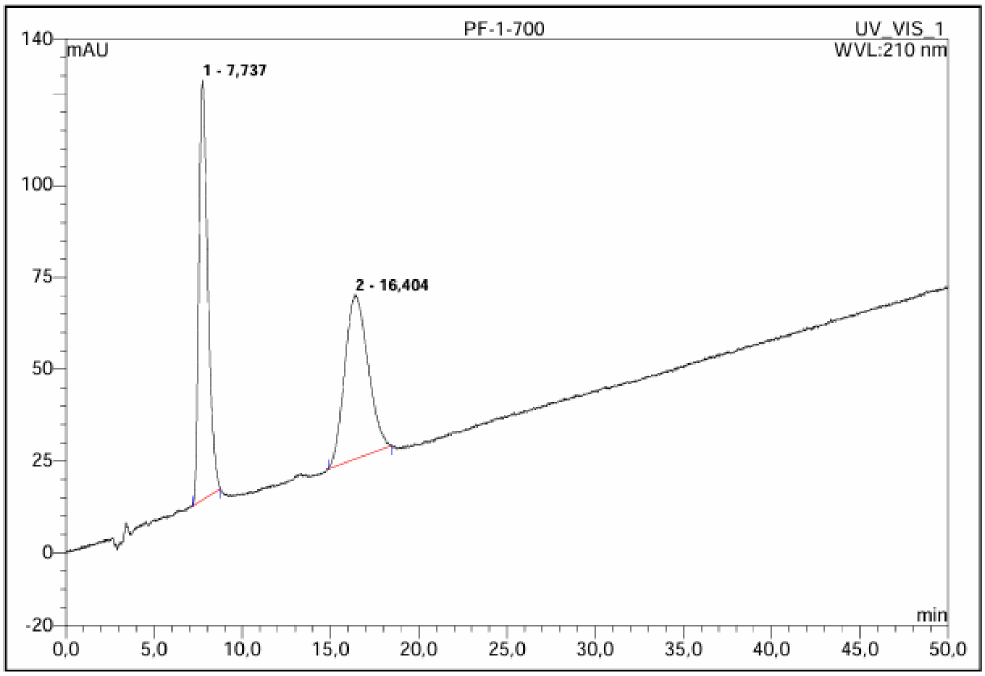

No.	Ret.Time min	Peak Name	Height mAU	Area mAU*min	Rel.Area %	Amount	Type
1	7,74	n.a.	114,391	67,329	49,61	n.a.	BMB
2	16,40	n.a.	44,824	68,398	50,39	n.a.	BMB*
Total:			159,215	135,727	100,00	0,000	

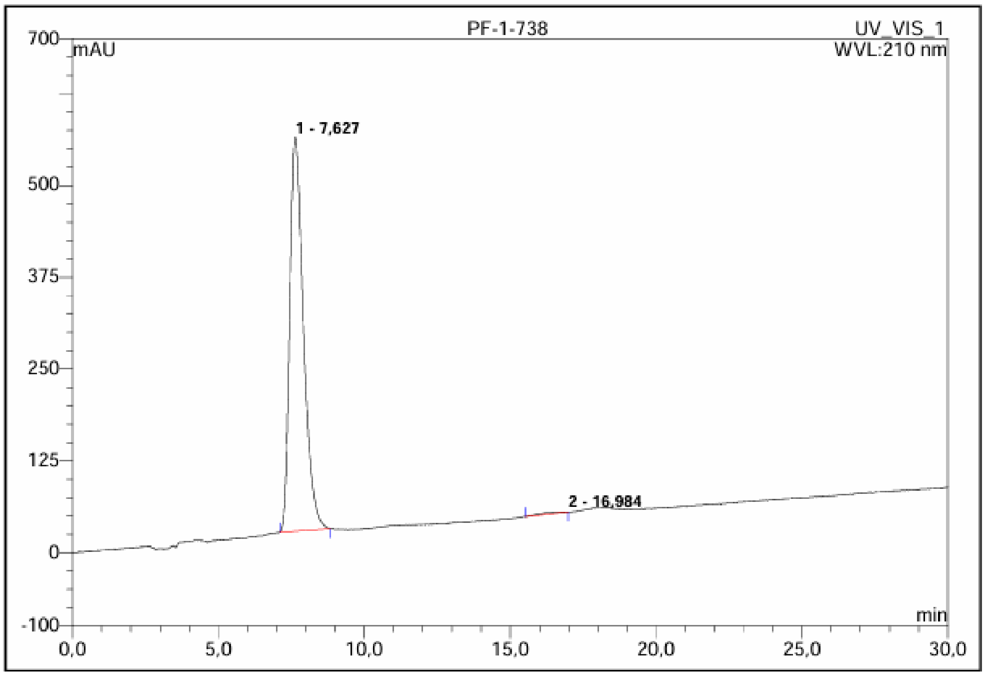

No.	Ret.Time min	Peak Name	Height mAU	Area mAU*min	Rel.Area %	Amount	Type
1	7,63	n.a.	536,408	290,979	99,52	n.a.	BMB
2	16,98	n.a.	0,000	1,403	0,48	n.a.	BMB*
Total:			536,408	292,382	100,00	0,000	

(*S*)-7-Benzyl-2-(cyclohex-1-en-1-yl)-6,7-dihydro-5*H*-pyrrolo[3,4-*b*]pyridin-5-one (3x)

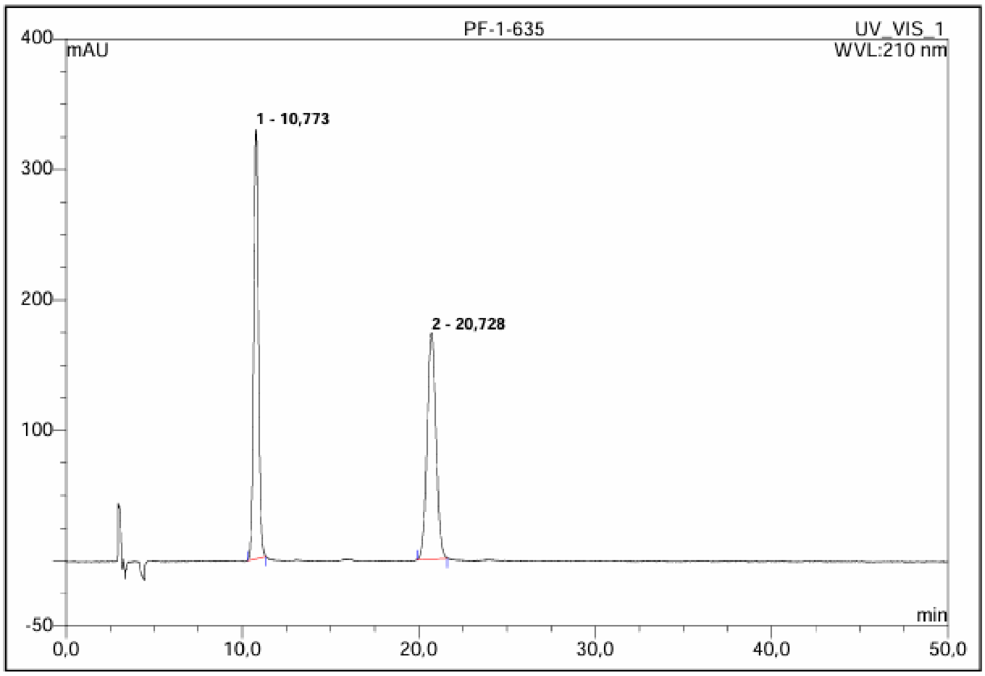

No.	Ret.Time min	Peak Name	Height mAU	Area mAU*min	Rel.Area %	Amount	Type
1	10,77	n.a.	329,377	101,076	50,05	n.a.	BMB
2	20,73	n.a.	173,331	100,858	49,95	n.a.	BMB
Total:			502,708	201,934	100,00	0,000	

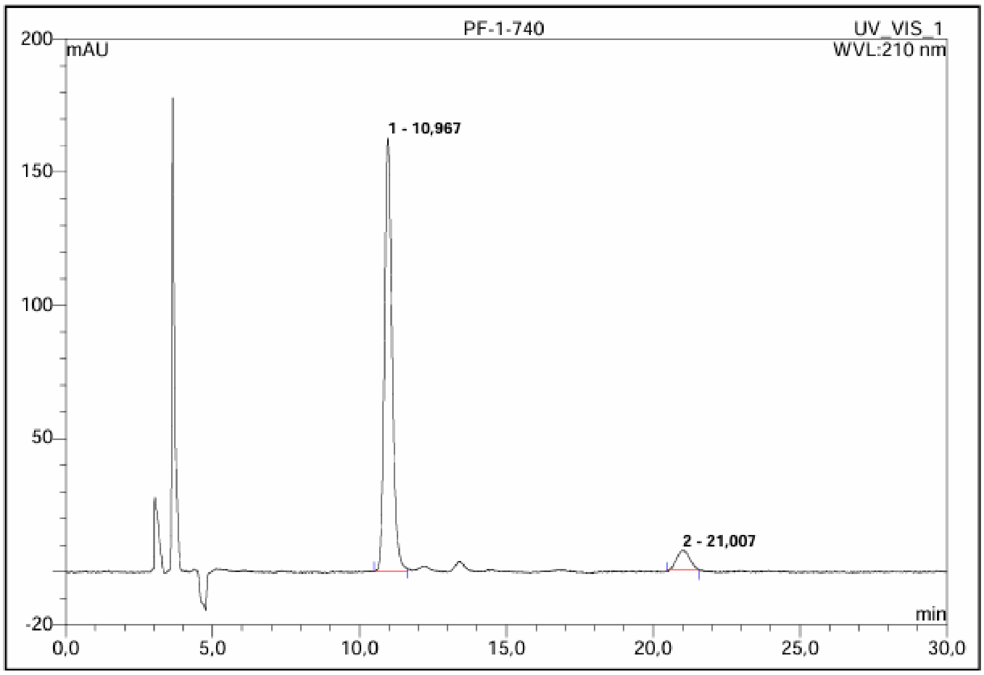

No.	Ret.Time min	Peak Name	Height mAU	Area mAU*min	Rel.Area %	Amount	Type
1	10,97	n.a.	162,524	48,630	92,74	n.a.	BM *
2	21,01	n.a.	7,381	3,807	7,26	n.a.	MB*
Total:			169,904	52,436	100,00	0,000	

16. References

[1] Pagare, P. P.; Ghatge, M. S.; Chen, Q.; Musayev, F. N.; Venitz, J.; Abdulmalik, O.; Zhang, Y.; Safo, M. K. Exploration of Structure–Activity Relationship of Aromatic Aldehydes Bearing Pyridinylmethoxy-Methyl Esters as Novel Antisickling Agents. *J. Med. Chem.* **2020**, *63*, 14724–14739.

[2] Freund, P.; Pauls, M.; Babushkina, D.; Pickl, T.; Bannwarth, C.; Bach, T. Photochemical Deracemization of 4,7-Diaza-1-isoindolinones by Unidirectional Hydrogen Atom Shuttling. *J. Am. Chem. Soc.* **2025**, *147*, 1434–1439.

[3] Galenko, E. E.; Kryukova, M. A.; Novikov, M. S.; Khlebnikov, A. F. Synthesis of Bi-, Ter-, and Quaterpyridinecarboxylates via Propargylisoxazole–Pyridine Rearrangement. *J. Org. Chem.* **2020**, *85*, 6109–6122.

[4] Vitaku, E.; Njardarson, J. T. A Mild meta-Selective C–H Alkylation of Catechol Mono-Ethers. *Eur. J. Org. Chem.* **2016**, 3679–3683.

[5] Makarov, A. S.; Kekhvaeva, A. E.; Chalikidi, P. N.; Abaev, V. T.; Trushkov, I. V.; Uchuskin, M. G. A Simple Synthesis of Densely Substituted Benzofurans by Domino Reaction of 2-Hydroxybenzyl Alcohols with 2-Substituted Furans. *Synthesis* **2019**, *51*, 3747–3757.

[6] Legouin, B.; Gayral, M.; Uriac, P.; Tomasi, S.; van de Weghe, P. Molecular Tweezers: Synthesis and Formation of Host–Guest Complexes. *Eur. J. Org. Chem.* **2010**, 5503–5508.

[7] Kitamura, M.; Ohmori, K.; Kawase, T.; Suzuki, K. Total Synthesis of Pradimicinone, the Common Aglycon of the Pradimicin–Benanomicin Antibiotics. *Angew. Chem., Int. Ed.* **1999**, *38*, 1229–1232.

[8] Müller, C.; Bauer, A.; Bach, T. Light-Driven Enantioselective Organocatalysis. *Angew. Chem. Int. Ed* **2009**, *48*, 6640–6642.

[9] APEX4 Suite of Crystallographic Software, Version 2021-10.0, Bruker AXS Inc., Madison, Wisconsin, USA, 2021.

[10] Bruker, SAINT, V8.41, Bruker AXS Inc., Madison, Wisconsin, USA.

[11] L. Krause, R. Herbst-Irmer, G. M. Sheldrick, D. Stalke, J. Appl. Cryst. 2015, 48, 3–10, doi:10.1107/S1600576714022985.

[12] G. M. Sheldrick, Acta Cryst. 2015, A71, 3–8, doi:10.1107/S2053273314026370.

[13] G. M. Sheldrick, Acta Cryst. 2015, C71, 3–8, doi:10.1107/S2053229614024218.

[14] C. B. Huebschle, G. M. Sheldrick, B. Dittrich, J. Appl. Cryst. 2011, 44, 1281–1284, doi:10.1107/S0021889811043202.

[15] Ed. E. Prince, International Tables for Crystallography Volume C, Mathematical, Physical and Chemical Tables, International Union of Crystallography, Chester, England, 2006, 500–502; 219–222; 193–199.

[16] C. R. Groom, I. J. Bruno, M. P. Lightfoot, S. C. Ward, Acta Cryst. 2016, B72, 171–179, doi:10.1107/S2052520616003954.

[17] D. Kratzert, FinalCif, V153, https://dkratzert.de/finalcif.html.

[18] Spitale, R. C.; Flynn, R. A.; Zhang, Q. C.; Crisalli, P.; Lee, B.; Jung, J. W.; Kuchelmeister, H. Y.; Batista, P. J.; Torre, E. A.; Kool, E. T.; Chang, H. Y. Structural imprints *in vivo* decode RNA regulatory mechanisms. *Nature* **2015**, *519*, 486–490.

[19] Goto, T.; Saito, M.; Sato, R. Magnesium Ion Assisted Highly Regio- and Chemoselective Reduction of 5H-Pyrrolo[3,4-b]pyridine-5,7(6H)-diones with Sodium Borohydride. A Convenient Synthesis of 6,7-Dihydro7-hydroxy-5H-pyrrolo[3,4-b]pyridin-5-ones. Bull. Chem. Soc. J. **1989**, 62, 1205–1240.

[20] Goto, T.; Utsunomiya, S.; Aiba, H.; Hayasaka, H.; Endo, M.; Watanabe, R.; Ishizaki, T.; Sato, R.; Saito, M. Synthesis of 7-Substituted 6,7-Dihydro-5H-pyrrolo[3,4-b]pyridin-5 ones. Reaction of 7-Hydroxy Derivatives with Nucleophiles. *Bull. Chem. Soc. J.* **1991**, *64*, 1901-1910.

[21] Clarke, K.; Goulding, J.; Scrowston, R. Preparation of some thiopyranopyridine derivatives. *J. Chem. Soc., Perkin Trans. 1*, **1984**, 1501-1505.

[22] Metcalf, B et al., Discovery of GBT440, an Orally Bioavailable R-State Stabilizer of Sickle Cell Hemoglobian. *ACS Med. Chem. Lett*. **2017**, *8*, 321-326.

[23] Schiwek, C. H.; Jandl, C.; Bach, T., Diastereoselective Rhodium-Catalyzed Hydrogenation of 2-Oxindoles and 3,4-Dihydroquinolones. *Org. Lett.* **2020**, *22*, 9468-9472.

[24] Ai, J. J.; Liu, B. B.; Li, J.; Wang, F.; Huang, C. M.; Rao, W.; Wang, S. Y., Fe–S Catalyst Generated In Situ from Fe(III)- and $S_3^{\bullet-}$-Promoted Aerobic Oxidation of Terminal Alkenes. *Org. Lett.* **2021**, *23*, 4705-4709.

[25] Xu, Y.; Mewes, L.; Thyrhaug, E.; Sláma, V.; Šanda, F. e.; Langhals, H.; Hauer, J., Isolating pure donor and acceptor signals by polarization-controlled transient absorption spectroscopy. *J. Phys. Chem. Lett.* **2023,** *14*, 5390-5396.

[26] Xu, Y.; Peschel, M.; Jänchen, M.; Foja, R.; Storch, G.; Thyrhaug, E.; de Vivie-Riedle, R.; Hauer, J., Determining Excited-State Absorption Properties of a Quinoid Flavin by Polarization-Resolved Transient Spectroscopy. *J. Phys. Chem. A* **2024,** *128*, 3830-3839.

[27] Riedle, E.; Beutter, M.; Lochbrunner, S.; Piel, J.; Schenkl, S.; Spörlein, S.; Zinth, W., Generation of 10 to 50 fs pulses tunable through all of the visible and the NIR. *Appl. Phys. B* **2000,** *71*, 457-465.

[28] Slavov, C.; Hartmann, H.; Wachtveitl, J., Implementation and evaluation of data analysis strategies for time-resolved optical spectroscopy. *Anal. Chem.* **2015,** *87* (4), 2328-2336.

[29] A. Kumar *et al.*, Transient absorption spectroscopy based on uncompressed hollow core fiber white light proves pre-association between a radical ion photocatalyst and substrate. *J. Chem. Phys.* **2023**, *158*, 144201.

S17 Appendix: Computational Studies

All relevant inputs/outputs of the quantum chemical calculations are provided in the esi.zip file. In addition, Table S9 and S10 contain energy contributions for all species discussed in this work.

S17.1 Structure Generation and Workflow

We investigate the series of benzyl-substituted aza-1-isoindolinones, namely 4-, 5-, 6- and 7-aza-1-isoindolinone as shown in Figure S15 next to the two chiral photocatalysts (−)-**2a** and (−)-**2b**.

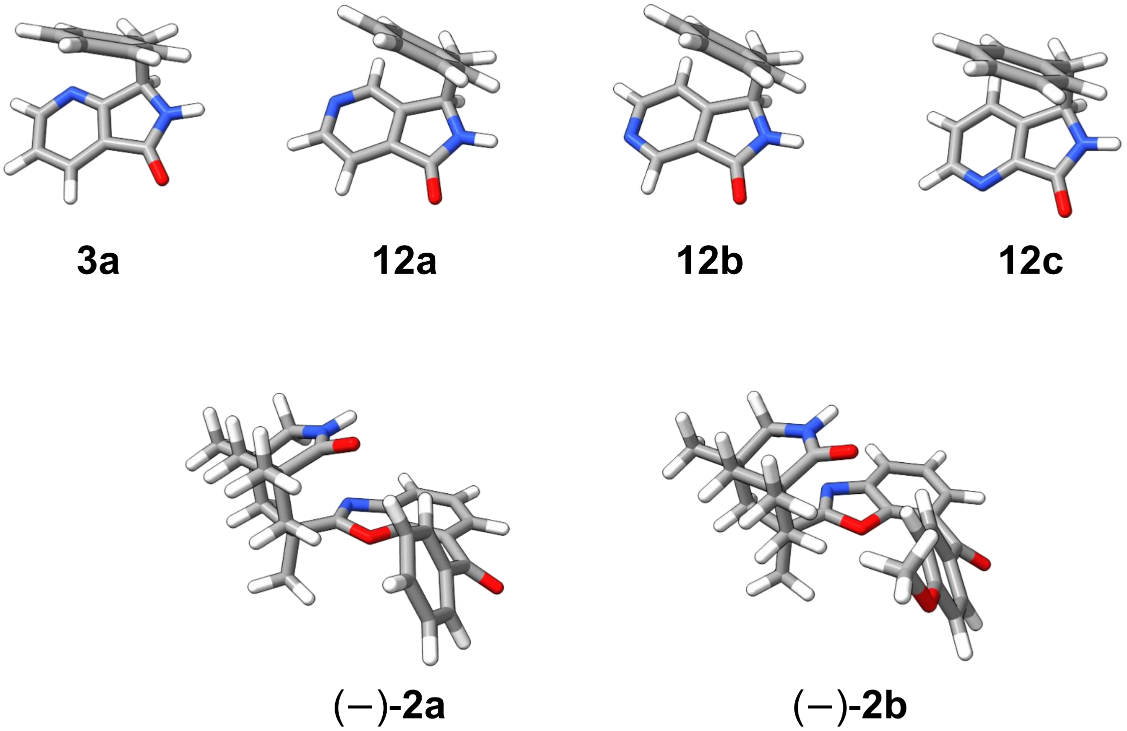

Figure S15: Minimum geometries (PBEh-3c+CPCM(DCM)) of the studied aza-indolinones **3a**, **12a**, **12b**, **12c**, and the photocatalysts (−)-**2a** and (−)-**2b**.

All structures are first optimized using the semiempirical electronic structure theory method GFN2-xTB[30] as available in the `xtb` program (version 6.7.0)[31] and the analytical linearized Poisson–Boltzmann (ALPB)[32] implicit solvation model for dichloromethane (DCM). DCM

is chosen to replace the experimentally used solvent α, α, α-trifluorotoluene due to its similar dielectric constant and because the latter is not parametrized for the ALPB model. For all species illustrated in Figure S15, we perform conformational sampling with the global optimization algorithm (GOAT)[33] in the ORCA quantum chemistry program (version 6.1.1).[34–36] We use default settings and GFN2-xTB+ALPB as electronic structure theory level for these calculations.

For each conformer ensemble (CE), we reoptimize all identified conformers with the PBEh-3c[37] composite density functional theory (DFT) method and the conductor-like polarizable continuum solvation model (CPCM)[38,39] in ORCA. As a member of the 3c family of methods, PBEh-3c offers a good description of geometries and noncovalent interactions at moderate computational cost. This composite method features a modified def2-SV(P) basis set[40] ('def2-mSVP'), D3(BJ) dispersion correction[41,42], a geometric counterpoise correction[43], and uses a modified functional based on that of Perdew, Burke and Ernzerhof[44,45] with a fixed fraction of 42% Fock exchange.[37]

To generate noncovalent complexes of substrate and photocatalyst, we employ the automated interaction site screening (aISS) algorithm[46] of the `xtb` program. This procedure is based on the intermolecular force-field approach xTB-iFF[47], which associates rigid fragments based on localized molecular orbital centers. As before, the default settings of aISS combined with GFN2-xTB+ALPB are used for this step.

Key features of further steps in the multilevel computational protocol employed in the present work are illustrated in Figure S16 and Table S7, and will be explained in the following.

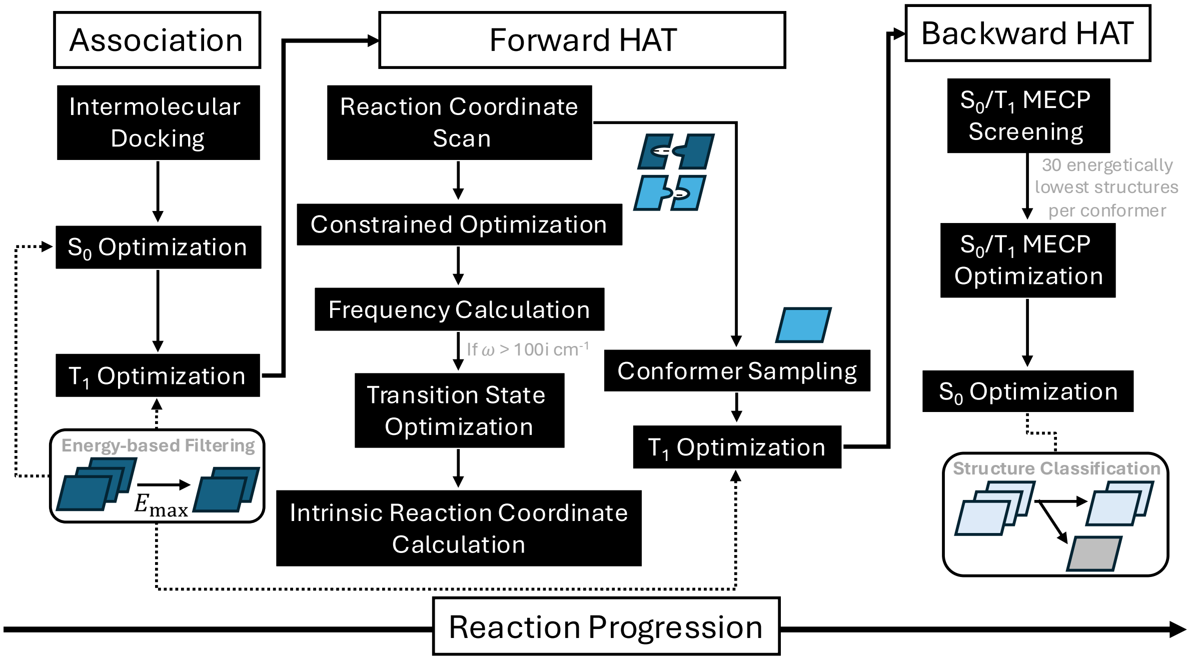

Figure S16: Schematic computational workflow used to study the photochemical deracemization of aza-isoindolinones in the present work. Methodological details to this scheme are collected in Table S7 below.

Table S7: Technical and methodological details of the employed multilevel workflow starting from dimeric noncovalent complex geometries. os-GFN2-xTB: Refers to an open-shell electronic configuration with two unpaired electrons throughout this work (keyword "--uhf 2" in `xtb`) and is used to approximate the T_1 excited-state with GFN2-xTB. $d_{\text{O-H}}$: Describes the interfragment coordinate for forward hydrogen atom transfer (fHAT) as exemplified in Fig. S17. TS: Transition State, IRC: Intrinsic Reaction Coordinate. [a]: Density functional theory calculations are performed with the ORCA quantum chemistry program. [b]: Calculations are carried out with a development version of the TeraChem program (based on version 1.9).[48,49] [c]: These steps are relevant for automatization and to manage the overall computational cost, due to the number of investigated conformers.

Workflow Step	Methodological Details	Notes
Intermolecular Docking	aISS with xTB-IFF	Based on GFN2-xTB+ALPB(DCM) electronic structure.
S_0 or T_1 Optimization[a]	RKS- or UKS-DFT with PBEh-3c+CPCM(DCM)	–
Reaction Coordinate Scan	os-GFN2-xTB+ALPB	Scan along $d_{\text{O}-\text{H}}$.
Constrained Optimization	os-GFN2-xTB+ALPB	Using the external optimizer functionality of ORCA and constraining $d_{\text{O-H}}$.
Frequency Calculation	GFN2-xTB+ALPB	–
TS Optimization[50]	PBEh-3c+CPCM	Using the `defgrid3` integration grid.
IRC Calculation[51]	"	Using the Hessian at the TS as input.
Conformer Sampling	GOAT with GFN2-xTB+ALPB	Based on the fHAT product obtained from the os-GFN2-xTB scan.
MECP Screening[52]	CREST (version 3.0.2)[53,54] with GFN2-xTB+ALPB	–
MECP Optimization	CREST at the UKS-PBEh-3c+CPCM level of theory[b]	–
	Additional Steps[c]	
Structure Identification	MolBar (version 1.2.0b)[55,56]	Identification and grouping of distinct molecular configurations via the `ensplit` tool.
Energy-based Filtering	CREST CREGEN routine	Remove structures $E_{\text{max}} = 3\ \text{kcal} \cdot \text{mol}^{-1}$ with respect to the lowest energy conformation (PBEh-3c+CPCM electronic energies) from a CE.

S17.2 Analysis of the Hydrogen Atom Transfer Steps

Similar to our previous work[2,57], we perform a minimum energy crossing point (MECP) screening[52] as available in the CREST program to identify configurations of the complexes after forward hydrogen atom transfer (fHAT) suitable for a back hydrogen atom transfer (bHAT) step. It is sensible to note here that the 30 energetically lowest MECP geometries (GFN2-xTB+ALPB) of each MECP ensemble are considered for reoptimization to a S_0/T_1 MECP at the UKS-PBEh-3c+CPCM(DCM) level of theory. MECP optimizations in CREST allow addressing Terachem for gradient computations via the `generic` runmode. Successive S_0 optimizations starting from these MECP geometries are performed with restricted Kohn–Sham (RKS)-DFT in order to identify stable intermediates or products. In Figure S17, we exemplarily present the complex geometries relevant for the photochemical deracemization reaction, involving substrate **3a** and catalyst (−)-**2b**.

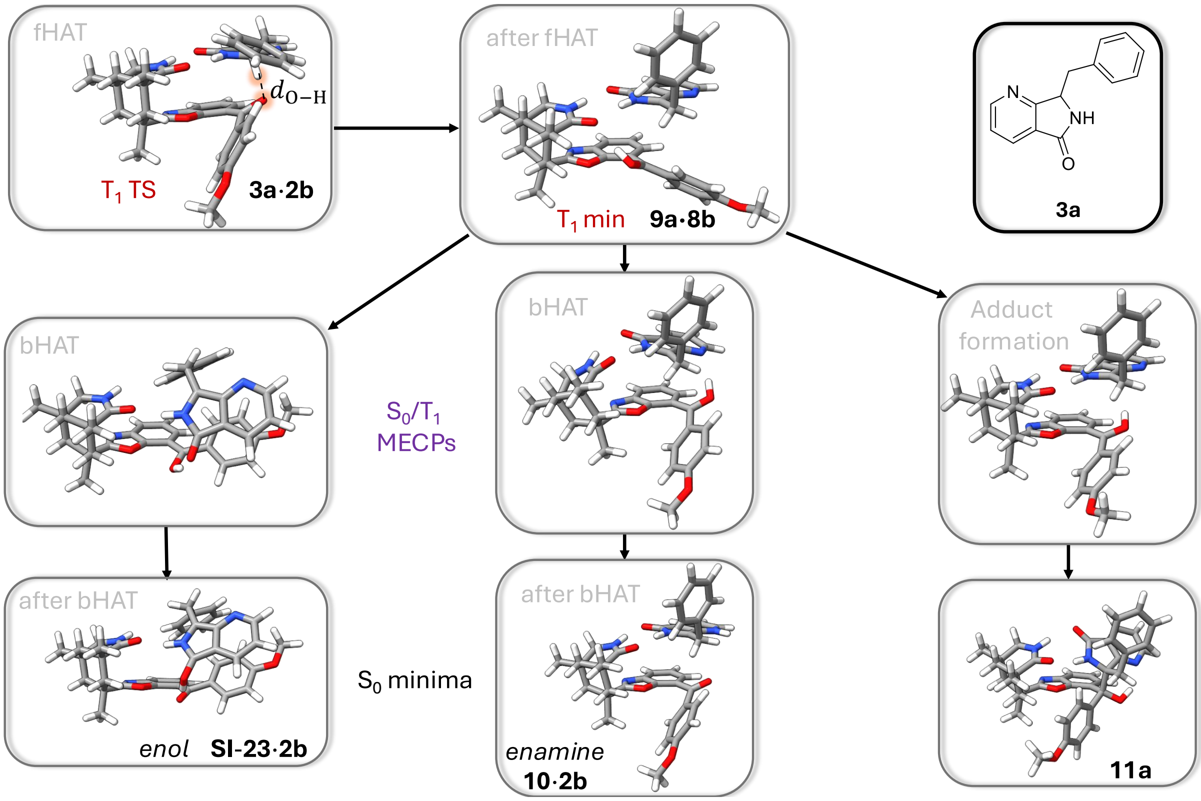

Figure S17: Relevant geometries for the photochemical deracemization of **3a** with photocatalyst (−)-**2b** before and after the forward and backward HAT steps. Black arrows show the reaction process starting from the fHAT via a T_1 transition state. For bHAT, three distinct intermediates/products **SI-23**, **10**, and **11a** are shown with the respective S_0/T_1 MECPs that lead to these species.

Employing the described sampling protocol, we are able to identify both putative bHAT sites (carbonyl oxygen, nitrogen in 4-position), as well as the experimentally confirmed adduct **11a**, where a C–C bond is formed between the former stereogenic center of **3a** and the carbon atom proximate to the benzophenone oxygen of (−)-**2b**. Analogous products and intermediates are also identified for the complexes involving 6-aza-1-isoindolinone **12b**.

MD Analysis of possible bHAT Pathways

To gain further insight about the structural rearrangement, we conduct a series of molecular dynamics (MD) simulations at the os-GFN2-xTB+ALPB level of theory. As described above, the T_1 potential energy surface is approximated using an open-shell electronic configuration

with two unpaired electrons.

NVT equilibrations at $T = 303.15$ K for a total simulation time $t_{\text{tot}} = 1$ ps with propagation steps $\Delta t_{\text{step}} = 1$ fs and snapshots extracted every $\Delta t_{\text{sample}} = 100$ fs are performed for all identified S_0/T_1 MECPs (337 geometries for **12b** as substrate). The productive runs (NVT, $t_{\text{tot}} = 10$ ps, $\Delta t_{\text{step}} = 1$ fs $\Delta t_{\text{sample}} = 100$ fs) are performed in the same way using the equilibration data for initial velocities. From these 33700 structures, we determine interactions between the nitrogen in 6-position and the transferred H atom ($I_{\text{6N...H}}$), as well as the substrates carbonyl oxygen with this H atom ($I_{\text{O...H}}$). The interfragment interaction $I_{A...B}$ between two atoms A and B is identified by a simple distance criterion including covalent bond radii $r_{\text{cov},A}, r_{\text{cov},B}$, and the Eucledian distance between the atoms $|\mathbf{R}_{AB}|$:

$$I(\mathbf{R}_{AB}, r_{\text{cov},A}, r_{\text{cov},B})_{A...B} = \begin{cases} 1 \text{ , if } |\mathbf{R}_{AB}| \leq 2(r_{\text{cov},A} + r_{\text{cov},B}) \\ 0 \text{ , otherwise} \end{cases} \quad . \tag{1}$$

We use $r_{\text{cov,H}} = 0.31$ Å, $r_{\text{cov,O}} = 0.66$ Å, $r_{\text{cov,N}} = 0.71$ Å.

Figure S18 shows the interactions over all MD trajectories, where at least one interaction is found according to Eq. 1.

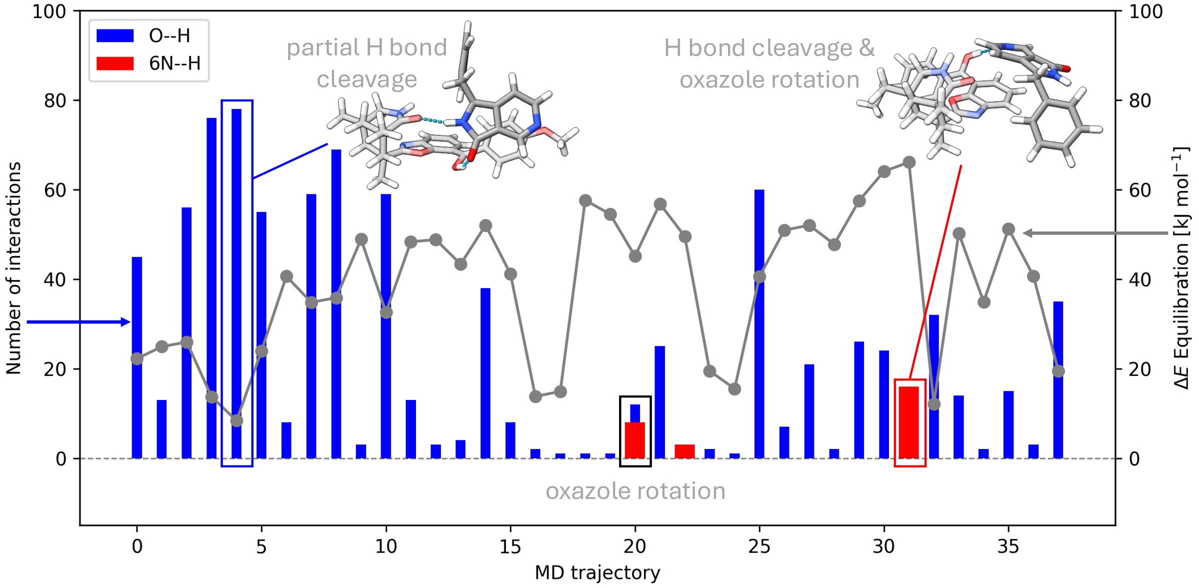

Figure S18: Distribution of the $I_{\text{O...H}}$ (blue) and $I_{\text{6N...H}}$ (red) interfragment interactions determined over all MD trajectories showing at least one interaction (Eq. 1). The right ordinate contains the GFN2-xTB energies of the equilibration runs to exemplify how far from equilibrium a trajectory propagated. Representative snapshots of the **13b**·**8b** complexes, i.e. the achiral radical intermediate of 6-aza-isoindolinone with the protonated ketyl radical of catalyst (−)-**2b**, are shown to clarify the necessary molecular reorientation.

Among the sampled structures, most trajectories indicate interactions between the **13b** carbonyl group and the hydrogen atom involved in the bHAT. As Figure S18 shows, this interaction requires, at least, cleavage of one hydrogen bond between **13b** and **8b** to allow for reorientation towards the benzophenone unit, where the H atom is situated after fHAT. An alternative mechanism—as is also found for the illustrated snapshot showing 6N–H interactions—would involve rotation of the catalyst oxazole moiety.

Checking Feasibility of other bHAT Coordinates through Oxazole Rotation

Given that the calculated barrier for the oxazole rotation in the T_1 state of isolated (−)-**2b** (*cf.* Fig. S19) amounts to only $\Delta G^{\ddagger} = 50\ \text{kJ} \cdot \text{mol}^{-1}$ (half life $t_{1/2} \approx 56\ \mu\text{s}$), we expect that this conformational change is feasible during the course of the photochemical reaction

in complex **13b·8b** as well. This rotation would facilitate bHAT to the thermodynamically favored enamine intermediate **14b** (*cf*. Scheme 8 in the main article).

Aside from the two productive pathways (bHAT to carbonyl oxygen or 6-N), formation of the resting state complex **SI-24** via carbon-carbon bond formation is feasible and observed among the sampled MECP geometries. This is particularly relevant, since the calculated barrier for oxazole rotation in **SI-24** amounts to $\Delta G^{\ddagger} = 69\ \mathrm{kJ\cdot mol^{-1}}$ ($t_{1/2} \approx 114$ ms).

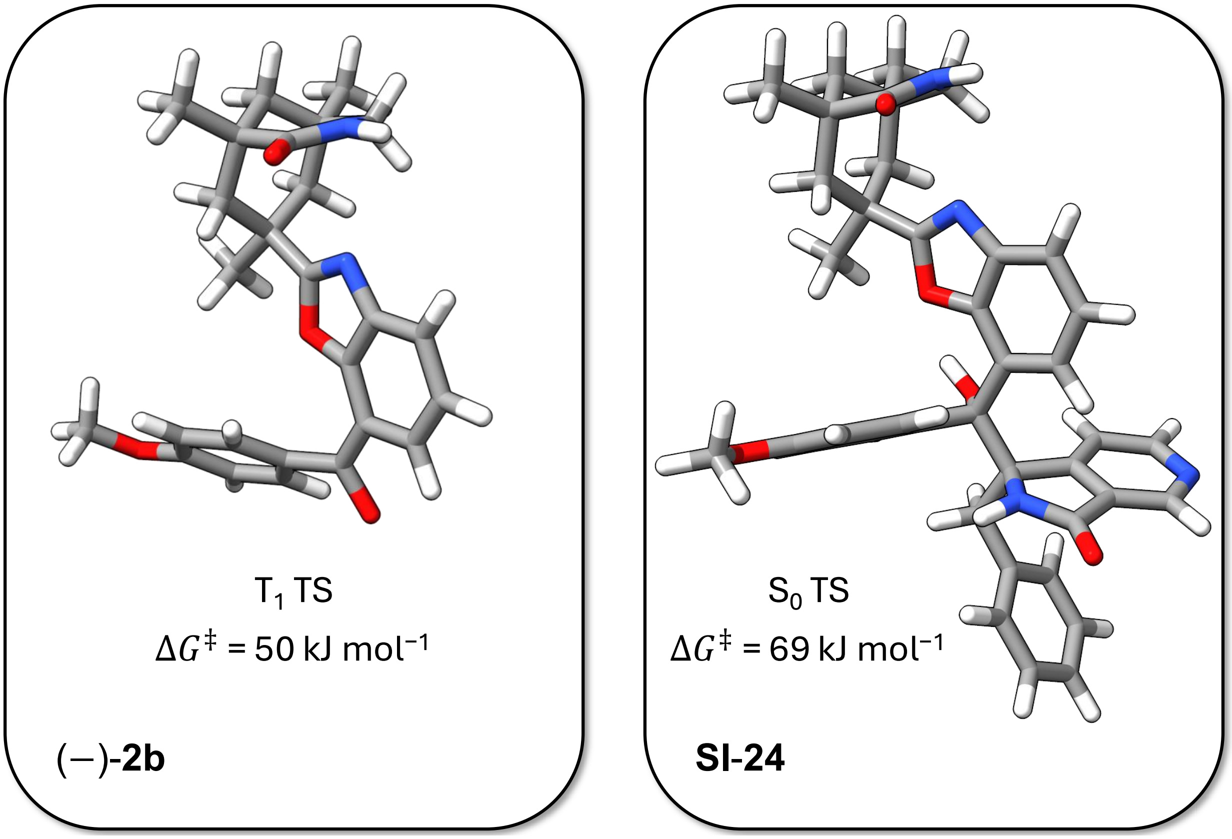

Figure S19: Transition states for the rotation of the oxazole moiety of (−)-**2b** (T_1 state, $\Delta G^{\ddagger} = 50\ \mathrm{kJ\cdot mol^{-1}}$) and of **SI-24** ($S_0$, $\Delta G^{\ddagger} = 69\ \mathrm{kJ\cdot mol^{-1}}$) computed at the PBEh-3c+CPCM level of theory. Contributions to the Gibbs free energy are explained in chapter S17.4 below.

While an increased rotational barrier in **SI-24** compared to (−)-**2b** is to be expected for steric reasons, rotation via **SI-24** in its electronic ground-state (S_0) appears to be suitable, and does not need to compete with the lifetime of the excited triplet state.

To examine how **SI-24** and adducts involving the other aza-isoindolinone subtrates are rein-

troduced in the photocatalytic cycle, exploratory calculations are carried out with hole-hole Tamm-Dancoff-approximated density functional theory (hh-TDA)[58,59] in combination with the PBEh-3c method in Terachem.[49] Here, we focus again on the adduct discussed in the main article **11a** that relates to **3a**. For **11a**, C–C bond scission upon optimization to the S_1 excited-state is revealed, and as the potential energy curve in Fig. S20 shows, this bond dissociation does not involve a barrier on the S_1 excited-state.

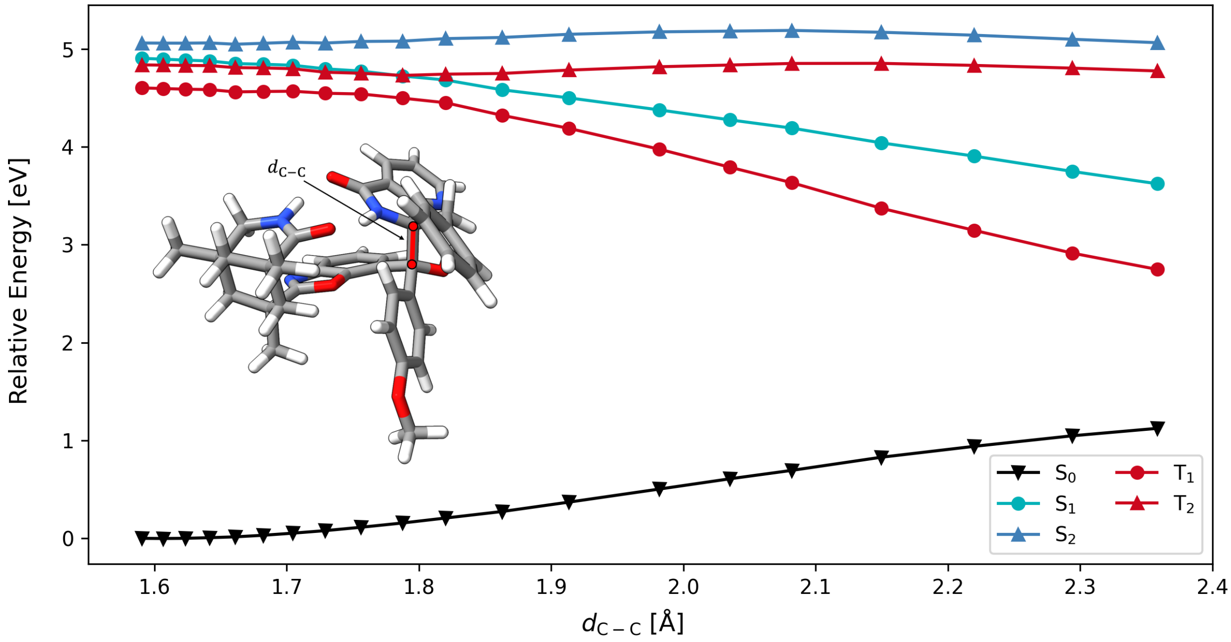

Figure S20: hh-TDA-PBEh-3c//GFN2-xTB+ALPB(DCM) potential energy curve along the carbon-carbon bond formed in **11a**. The scan coordinate d_{C-C} is highlighted in the structure. The GFN2-xTB geometries are obtained using an elevated electronic temperature (T_{el} = 4000 K).

Notably, the T_1 state also does not involve a significant barrier along d_{C-C} and the energetic profile is similar to that of the S_1 state.

To clarify whether bond scission may be feasible via a ground-state mechanism, we further compute the barrier to the associated TS with UKS-DFT (PBEh-3c+CPCM). With $\Delta G^{\ddagger}$ = 103 kJ · mol^{-1}, **11a** is thermally stable (half life of $t_{1/2} \approx 1.1$ d), and thus, photochemical bond scission seems to be the more relevant pathway to convert the resting state complex back into the reactive biradical intermediate **8b·9a**. Photoexcitation (*cf.* Tab. S8) causes C–

C bond scission in the adduct and continously leads to reformation of a biradical intermediate that, after structural rearrangement, gets access to a suitable bHAT coordinate. The same conclusion can be drawn for the related adducts.

S17.3 Photophysical Characterization

To compute vertical excitation energies, we use an extension of the density functional theory/multireference configuration interaction (DFT/MRCI)[60,61] method, p-DFT/MRCI as proposed by Schuurman and Neville.[62] Different from only the energy-based selection criterion employed in DFT/MRCI[60,61], p-DFT/MRCI further reduces the number of configuration state functions employing Epstein–Nesbet perturbation theory, therby achieving impressive computational speed-ups. Aside from the advanced configuration selection, p-DFT/MRCI introduces a set of other technical modifications in contrast to the parent method DFT/MRCI, like the reference space selection and perturbative energy corrections. The interested reader is referred to Ref. 62 for further details about this method. We use the publicly available GRaCI[63] code (version 1.2.0) by the Schuurman group to carry out these calculations. As common for most Hamiltonian parametrizations of DFT/MRCI, the anchor configuration is computed using the BHLYP[64] exchange-correlation functional (50% Fock exchange) and a def2-SVP basis set. The configuration selection in p-DFT/MRCI is effectively driven by the pruning threshold α, which we set to 0.98 in our calculations to resemble DFT/MRCI energies more closely. Initial tests suggest that the differences to DFT/MRCI amount to $\simeq$ 0.10 eV, which is roughly on the order of the intrinsic error of DFT/MRCI and sufficient for comparing relative differences across different molecules.

The short parametrization and the associated energy cutoff (0.8 E_h) of the R2018[65] Hamiltonian by Heil *et al.* is chosen. The lowest eight eigenvalues are requested in our calculations and otherwise, default settings are employed. GRaCI uses the open-source python framework PySCF[66], which, in turn, uses LIBXC[67] for the exchange-correlation components on the BHLYP reference calculation.

Table S8 lists selected singlet vertical excitation energies of the ground-state complexes and adducts of **3a** or **12b** with (−)-**2b** and (−)-**2a**.

Table S8: Absolute energy E, vertical excitation energy $\Delta E_{0\mathrm{n}}$ and electric dipole oscillator strength f_{0n} of complexes **3a**·(−)-**2a**, **3a**·(−)-**2b**, and **12b**·(−)-**2a**, **12b**·(−)-**2b** as well as the associated adducts **SI-25**, **11a** and **SI-26**, **SI-24**.

State	E [$\mathrm{E_h}$]	$\Delta E_{0\mathrm{n}}$ [eV]	f_{0n} [a.u.]
	**3a**·(−)-**2a**		
S_0	−2026.395084	-	-
S_1	−2026.258083	3.73	0.00
S_2	−2026.239648	4.23	0.11
S_3	−2026.227192	4.57	0.00
	**3a**·(−)-**2b**		
S_0	−2140.770197	-	-
S_1	−2140.635048	3.68	0.01
S_2	−2140.612121	4.30	0.11
S_3	−2140.608851	4.39	0.31
	**SI-25**		
S_0	−2026.385736	-	-
S_1	−2026.219138	4.53	0.00
S_2	−2026.210346	4.77	0.00
S_3	−2026.207655	4.85	0.12
	**11a**		
S_0	−2140.759243	-	-
S_1	−2140.592375	4.54	0.00
S_2	−2140.584569	4.75	0.02
S_3	−2140.582767	4.80	0.03
	**12b**·(−)-**2a**		
S_0	−2026.390656	-	-
S_1	−2026.256010	3.66	0.00
S_2	−2026.231826	4.32	0.10
S_3	−2026.220256	4.64	0.02
	**12b**·(−)-**2b**		
S_0	−2140.767687	-	-
S_1	−2140.629614	3.76	0.01
S_2	−2140.610250	4.28	0.08
S_3	−2140.605031	4.43	0.52
	**SI-26**		
S_0	−2026.374232	-	-
S_1	−2026.201602	4.70	0.01
S_2	−2026.199810	4.75	0.07
S_3	−2026.193381	4.92	0.00
	**SI-24**		
S_0	−2140.605625	-	-
S_1	−2140.592333	4.65	0.01
S_2	−2140.587801	4.70	0.06
S_3	−2140.586576	4.79	0.06

Comparing first complexes **3a**·(−)-**2a** and **3a**·(−)-**2b**, we find an increase in the combined oscillator strengths of the S_1–S_3, in particular for the S_3 state with 0.31 a.u. for **3a**·(−)-**2b** compared to 0.00 a.u. in **3a**·(−)-**2a**. Given that we expect population of the S_1 of **11a** to be feasible under experimental irradiation conditions (λ = 350 nm $\approx$ 3.54 eV), it appears likely that the S_3 state of the noncovalent complex **3a**·**2b** will be populated primarily at the beginning of the photochemical reaction. It is important to stress here that only the intensity maximum of the employed UV lamp (*cf*. Fig. S2) is centered at 350 nm, but that photons of higher energy may allow for population of energetically higher-lying excited-states. These should be statistically relevant due to the Gaussian-like spectral range the lamp covers.[2]
Ultimately, the S_1 state of complex **3a**·(−)-**2b** may be reached after internal conversion (IC) from a higher-lying excited singlet state (S_2, S_3). Following intersystem crossing (ISC), the benzophenone T_1 state will be populated, which serves as starting point for the photochemical deracemization reaction. The experimentally observed general increase in *ee*'s upon i) a decreased maximum irradiation wavelength from 366 nm to 350 nm and ii) an increased absorption coefficient of the low-energy shoulder from (−)-**2a** to (−)-**2b**, qualitatively aligns with the calculated $\Delta E_{0\mathrm{n}}$ and $f_{0\mathrm{n}}$.
Moving to the noncovalent catalyst complexes involving **12b**, we find a similar situation. In fact, the ratio of the combined S_1–S_3 oscillator strength $F = \sum_{i=1}^{3} f_{0i}^{\mathrm{X \cdot 2b}} / f_{0i}^{\mathrm{X \cdot 2a}}$ is even more in favor of the MeO-substituted noncovalent complex **12b**·(−)-**2b** (F = 5.0) than observed for complexes involving **3a** (F = 3.7).
A comparison of complexes **3a**·(−)-**2a** and **3a**·(−)-**2b** with the respective adducts reveals that population of even the S_1 state of these "resting-state" species requires higher excitation energies of 4.53 eV (**SI-25**) and 4.54 eV (**11a**), respectively. The pronounced blue-shift of, e.g. 0.86 eV for **11a** compared to **3a**·(−)-**2b**, accompanied with the overall low oscillator strenghts suggests that a photochemically induced C–C bond scission is more difficult to achieve under experimental conditions than photoexcitation of the bimolecular complexes at the beginning of the reaction. As alluded to above, this reaction step should generally

proceed more efficiently probing the resting-state species with a light of lower λ.

S17.4 Computation of Gibbs Free Energies

All (free) energy differences reported in the present work refer to the minimum free energy conformation exclusively. If not stated otherwise, the Gibbs free energy of species i is computed according to Eq. 2:

$$G_i = E_{\mathrm{gas},i} + G_{\mathrm{TRV},i} + \delta G_{\mathrm{solv},i}\,. \tag{2}$$

$E_{\mathrm{gas},i}$ is the electronic energy (PW6B95[68]-D4[69]/def2-QZVPP[40]) computed in gas phase at the PBEh-3c+CPCM minimum geometry. Here, the `defgrid3` numerical integration grid, the resolution-of-the-identity (RI-J)[70,71] for Coulomb integrals with the def2/J[72] auxiliary basis, and chain-of-spheres for exchange (COSX)[73,74] integral approximations are employed. Thermostatistical contributions due to the translational, rotational, and vibrational (TRV) degrees of freedom of the nuclei at finite temperature (T = 303.15 K) are described using a particle-in-a-box, rigid-rotor, and modified harmonic oscillator (mRRHO)[75] model. Zero-point vibrational energy contributions are included as well. The harmonic frequencies (PBEh-3c) are scaled by 0.95 as proposed in the literature.[76] A standard-state solvation correction $\delta G_{\mathrm{solv},i} = G_{\mathrm{solv},i} - E_{\mathrm{gas},i}$ is computed at the GFN2-xTB+ALPB//PBEh-3c+CPCM level of theory for the conversion of 1 mol gas at 1 bar to a 1 M solution (keyword "bar1M") with `xtb`.

Product Stability for Different Solvents

To investigate the impact of solvent polarity on the products (enamines **14**, enols **15**) stability relative to the respective substrate, we modified the free energy expression (Eq. 2) to Eq. 3:

$$G_i = E_{\mathrm{solv},i} + G_{\mathrm{TRV},i}\,. \tag{3}$$

$E_{\mathrm{solv},i}$ here includes the CPCM solvation contribution to the electronic energy ([PW6B95-D4/def2-QZVPP//PBEh-3c]+CPCM), which is why we omit $\delta G_{\mathrm{solv},i}$ (used in combination with a gas-phase $E_{\mathrm{gas},i}$ in Eq. 2). $G_{\mathrm{TRV},i}$ refers to the same contribution as declared above. For different solvents, all geometries are reoptimized at the PBEh-3c level of theory including CPCM for the respective solvent. The resulting heatmaps for three different solvents (Toluene, DCM, and MeCN) are collected in Figure S21.

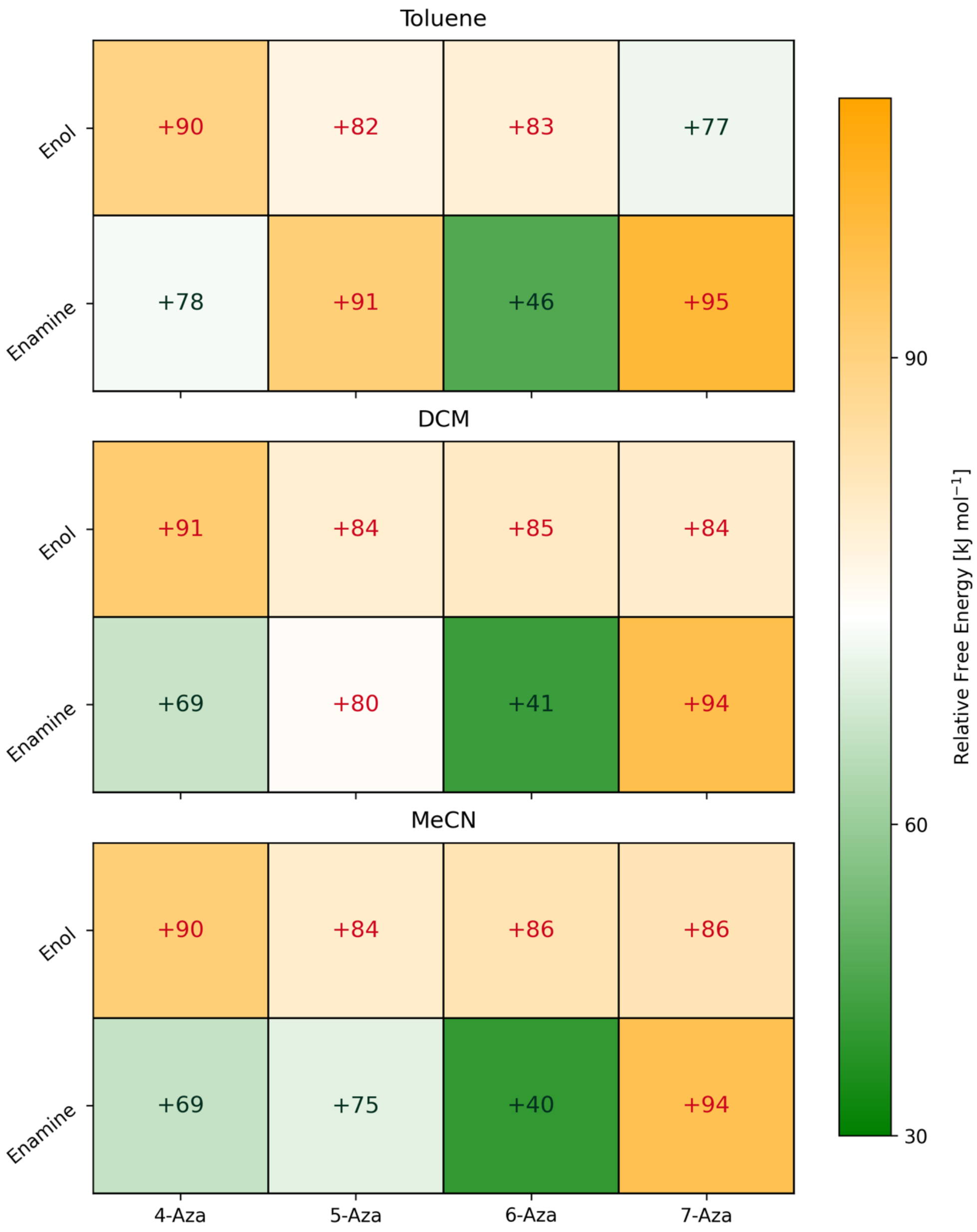

Figure S21: Heatmaps (see Eq. 3 for the Gibbs free energy contributions) for the relative product stabilities of the enamine and enol products of **3a**, **12a**, **12b**, and **12c**.

Comparing first Scheme 8 in the main article with the above Figure, we see that the relative trends in DCM are not substantially altered moving from Eq. 2 to 3. Having verified the similarity between the employed relative free energies, we turn to the changes that became clear from Figure S21. We find a stabilization of **10** by 9 $kJ \cdot mol^{-1}$ moving from Toluene to DCM and MeCN. The associated enol product is not stabilized for **3a**. The most drastic

changes are observed for the bHAT products of the 5-Aza compound **12a**: the enamine **14a** is stabilized by 16 $kJ \cdot mol^{-1}$ compared to **12a**. This finding nicely shows that the solvation stabilizes the zwitterionic character in the 5-Aza enamine (**14a**), which agrees with the experimental observation of increased yield and *ee*, when conducting the photochemical reaction in MeCN instead of DCM (see main article).

Table S9: Contributions to the Gibbs free energy of all relevant species computed according to Eq. 2. See the text for details on the theory level. a: Refers to the oxazole rotation. b: Refers to the fHAT step. c: Refers to the carbon-carbon bond cleavage, $d_{\mathrm{C-C}}$. d: Electronic energy refering to the PBEh-3c+CPCM level of theory.

Species	State	E_{el} [$\mathrm{E_h}$]	G_{TRV} [$\mathrm{E_h}$]	δG_{solv} [$\mathrm{E_h}$]	G [$\mathrm{E_h}$]
**3a**	S_0	−726.746442	0.186803	−0.022281	−726.581920
**10**	S_0	−726.714765	0.185848	−0.024931	−726.553848
**SI-23**	S_0	−726.708619	0.185085	−0.019476	−726.543010
**12a**	S_0	−726.741227	0.186823	−0.022539	−726.576943
**14a**	S_0	−726.699461	0.188318	−0.028850	−726.539993
**15a**	S_0	−726.706396	0.185478	−0.019901	−726.540819
**12b**	S_0	−726.741882	0.186810	−0.022860	−726.577932
**14b**	S_0	−726.720261	0.186935	−0.025759	−726.559085
**15b**	S_0	−726.707633	0.185823	−0.019931	−726.541741
**12c**	S_0	−726.738295	0.186942	−0.023791	−726.575144
**14c**	S_0	−726.699542	0.186731	−0.025578	−726.538389
**15c**	S_0	−726.711774	0.185571	−0.019147	−726.545350
(−)-**2a**	S_0	−1305.838109	0.403432	−0.037312	−1305.471989
(−)-**2b**	S_0	−1420.555107	0.432756	−0.040479	−1420.162830
	T_1	−1420.450260	0.427878	−0.043107	−1420.065489
	T_1 (TS)^a	−1420.429899	0.428603	−0.044546	−1420.045842
**3a**·(−)-**2b**	S_0	−2147.331951	0.644660	−0.062327	−2146.749618
	T_1	−2147.229981	0.639494	−0.062690	−2146.653177
	T_1 (TS)^b	−2147.220034	0.637684	−0.061551	−2146.643901
**9a**·**8b**	T_1	−2147.278983	0.641562	−0.059963	−2146.697384
	T_1^d	−2138.927365	-	-	-
	S_0/T_1 MECP^d	−2138.925859	-	-	-
**10**·(−)-**2b**	S_0	−2147.304074	0.645385	−0.058710	−2146.717399
**13b**·**8b**	T_1^d	−2138.925815	-	-	-
	S_0/T_1 MECP^d	−2138.917861	-	-	-
**13c**·**8b**	T_1^d	−2138.929416	-	-	-
	S_0/T_1 MECP^d	−2138.920603	-	-	-
**SI-24**	S_0	−2147.317044	0.649577	−0.056550	−2146.724017
	S_0 (TS)^b	−2147.284412	0.648953	−0.062328	−2146.697787
	S_0 (TS)^c	−2147.272776	0.645545	−0.057700	−2146.684931

Table S10: Contributions to the Gibbs free energy of all aza-isoindolinones computed according to Eq. 3 for Toluene, DCM and MeCN. See the text for details on the theory level.

Species	State	E_{solv} [$\mathrm{E_h}$]	G_{TRV} [$\mathrm{E_h}$]	G [$\mathrm{E_h}$]
		Toluene		
**3a**	S_0	−726.756917	0.186945	−726.569972
**10**	S_0	−726.717336	0.186625	−726.530711
**SI-23**	S_0	−726.720227	0.185787	−726.534440
**12a**	S_0	−726.727874	0.187847	−726.540027
**14a**	S_0	−726.718790	0.184773	−726.534017
**15a**	S_0	−726.751635	0.186966	−726.564669
**12b**	S_0	−726.720275	0.184469	−726.535806
**14b**	S_0	−726.753325	0.186959	−726.566366
**15b**	S_0	−726.714669	0.186594	−726.528075
**12c**	S_0	−726.752418	0.186945	−726.565473
**14c**	S_0	−726.735266	0.187026	−726.548240
**15c**	S_0	−726.721558	0.186213	−726.535345
		DCM		
**3a**	S_0	−726.763193	0.186803	−726.576390
**10**	S_0	−726.728981	0.187239	−726.541742
**SI-23**	S_0	−726.727439	0.185823	−726.541616
**12a**	S_0	−726.736334	0.185848	−726.550486
**14a**	S_0	−726.726132	0.185478	−726.540654
**15a**	S_0	−726.760446	0.186942	−726.573504
**12b**	S_0	−726.726929	0.185085	−726.541844
**14b**	S_0	−726.760660	0.186810	−726.573850
**15b**	S_0	−726.724381	0.186796	−726.537585
**12c**	S_0	−726.759296	0.186823	−726.572473
**14c**	S_0	−726.744974	0.186935	−726.558039
**15c**	S_0	−726.727213	0.185571	−726.541642
		MeCN		
**3a**	S_0	−726.765986	0.187548	−726.578438
**10**	S_0	−726.732934	0.186853	−726.546081
**SI-23**	S_0	−726.729692	0.186102	−726.543590
**12a**	S_0	−726.738816	0.186413	−726.552403
**14a**	S_0	−726.728448	0.185794	−726.542654
**15a**	S_0	−726.763989	0.187636	−726.576353
**12b**	S_0	−726.728993	0.184918	−726.544075
**14b**	S_0	−726.763688	0.187595	−726.576093
**15b**	S_0	−726.727643	0.186938	−726.540705
**12c**	S_0	−726.762219	0.187623	−726.574596
**14c**	S_0	−726.747873	0.187209	−726.560664
**15c**	S_0	−726.728873	0.185075	−726.543798

References

[30] Bannwarth, C.; Ehlert, S.; Grimme, S. GFN2-xTB—An Accurate and Broadly Parametrized Self-Consistent Tight-Binding Quantum Chemical Method with Multipole Electrostatics and Density-Dependent Dispersion Contributions. *J. Chem. Theory Comput.* **2019**, *15*, 1652–1671.

[31] Semiempirical Extended Tight-Binding Program Package. (https://github.com/grimme-lab/xtb).

[32] Ehlert, S.; Stahn, M.; Spicher, S.; Grimme, S. Robust and Efficient Implicit Solvation Model for Fast Semiempirical Methods. *J. Chem. Theory Comput.* **2021**, *17*, 4250–4261.

[33] de Souza, B. GOAT: A Global Optimization Algorithm for Molecules and Atomic Clusters. *Angew. Chem. Int. Ed.* **2025**, *64*, e202500393.

[34] Neese, F. The ORCA Program System. *WIREs Comput. Mol. Sci.* **2012**, *2*, 73–78.

[35] Neese, F.; Wennmohs, F.; Becker, U.; Riplinger, C. The ORCA Quantum Chemistry Program Package. *J. Chem. Phys.* **2020**, *152*, 224108.

[36] Neese, F. Software Update: The ORCA Program System—Version 6.0. *WIREs Comput. Mol. Sci.* **2025**, *15*, e70019.

[37] Grimme, S.; Brandenburg, J. G.; Bannwarth, C.; Hansen, A. Consistent Structures and Interactions by Density Functional Theory with Small Atomic Orbital Basis Sets. *J. Chem. Phys.* **2015**, *143*, 054107.

[38] Barone, V.; Cossi, M. Quantum Calculation of Molecular Energies and Energy Gradients in Solution by a Conductor Solvent Model. *J. Phys. Chem. A* **1998**, *102*, 1995–2001.

[39] Garcia-Ratés, M.; Neese, F. Effect of the Solute Cavity on the Solvation Energy and Its Derivatives within the Framework of the Gaussian Charge Scheme. *J. Comput. Chem.* **2020**, *41*, 922–939.

[40] Weigend, F.; Ahlrichs, R. Balanced Basis Sets of Split Valence, Triple Zeta Valence and Quadruple Zeta Valence Quality for H to Rn: Design and Assessment of Accuracy. *Phys. Chem. Chem. Phys.* **2005**, *7*, 3297–3305.

[41] Grimme, S.; Antony, J.; Ehrlich, S.; Krieg, H. A Consistent and Accurate Ab Initio Parametrization of Density Functional Dispersion Correction (DFT-D) for the 94 Elements H-Pu. *J. Chem. Phys.* **2010**, *132*, 154104.

[42] Grimme, S.; Ehrlich, S.; Goerigk, L. Effect of the Damping Function in Dispersion Corrected Density Functional Theory. *J. Comput. Chem.* **2011**, *32*, 1456–1465.

[43] Kruse, H.; Grimme, S. A Geometrical Correction for the Inter- and Intra-Molecular Basis Set Superposition Error in Hartree-Fock and Density Functional Theory Calculations for Large Systems. *J. Chem. Phys.* **2012**, *136*, 154101.

[44] Perdew, J. P.; Burke, K.; Ernzerhof, M. Generalized Gradient Approximation Made Simple. *Phys. Rev. Lett.* **1996**, *77*, 3865–3868.

[45] Adamo, C.; Barone, V. Toward Reliable Density Functional Methods without Adjustable Parameters: The PBE0 Model. *J. Chem. Phys.* **1999**, *110*, 6158–6170.

[46] Plett, C.; Grimme, S. Automated and Efficient Generation of General Molecular Aggregate Structures. *Angew. Chem. Int. Ed.* **2023**, *62*, e202214477.

[47] Grimme, S.; Bannwarth, C.; Caldeweyher, E.; Pisarek, J.; Hansen, A. A General Intermolecular Force Field Based on Tight-Binding Quantum Chemical Calculations. *J. Chem. Phys.* **2017**, *147*, 161708.

[48] Seritan, S.; Bannwarth, C.; Fales, B. S.; Hohenstein, E. G.; Kokkila-Schumacher, S. I. L.; Luehr, N.; Snyder, J. W., Jr.; Song, C.; Titov, A. V.; Ufimtsev, I. S.; Martínez, T. J. TeraChem: Accelerating Electronic Structure and Ab Initio Molecular Dynamics with Graphical Processing Units. *J. Chem. Phys.* **2020**, *152*, 224110.

[49] Steinbach, P.; Bannwarth, C. Combining Low-Cost Electronic Structure Theory and Low-Cost Parallel Computing Architecture. *Phys. Chem. Chem. Phys.* **2024**, *26*, 16567–16578.

[50] Baker, J. An Algorithm for the Location of Transition States. *J. Comput. Chem.* **1986**, *7*, 385–395.

[51] Ishida, K.; Morokuma, K.; Komornicki, A. The Intrinsic Reaction Coordinate. An Ab Initio Calculation for HNC→HCN and H-+CH4→CH4+H-. *J. Chem. Phys.* **1977**, *66*, 2153–2156.

[52] Pracht, P.; Bannwarth, C. Fast Screening of Minimum Energy Crossing Points with Semiempirical Tight-Binding Methods. *J. Chem. Theory Comput.* **2022**, *18*, 6370–6385.

[53] Pracht, P.; Grimme, S.; Bannwarth, C.; Bohle, F.; Ehlert, S.; Feldmann, G.; Gorges, J.; Müller, M.; Neudecker, T.; Plett, C.; Spicher, S.; Steinbach, P.; Wesołowski, P. A.; Zeller, F. CREST—A Program for the Exploration of Low-Energy Molecular Chemical Space. *J. Chem. Phys.* **2024**, *160*, 114110.

[54] CREST - A Program for the Automated Exploration of Low-Energy Molecular Chemical Space. (https://github.com/crest-lab/crest).

[55] van Staalduinen, N.; Bannwarth, C. MolBar: A Molecular Identifier for Inorganic and Organic Molecules with Full Support of Stereoisomerism. *Digit. Discov.* **2024**, *3*, 2298–2319.

[56] MolBar: A Molecular Identifier for Inorganic and Organic Molecules with Full Support of Stereoisomerism. (https://git.rwth-aachen.de/bannwarthlab/molbar).

[57] Ghosh, B.; Iglhaut, M.; Babushkina, D.; Pauls, M.; Bannwarth, C.; Bach, T. Photochemical Deracemization of Chromanes and Its Application to the Synthesis of Enantiopure Bioactive Compounds. *Angew. Chem. Int. Ed.* **2026**, *65*, e21436.

[58] Bannwarth, C.; Yu, J. K.; Hohenstein, E. G.; Martínez, T. J. Hole–Hole Tamm–Dancoff-approximated Density Functional Theory: A Highly Efficient Electronic Structure Method Incorporating Dynamic and Static Correlation. *J. Chem. Phys.* **2020**, *153*, 024110.

[59] Yu, J. K.; Bannwarth, C.; Hohenstein, E. G.; Martínez, T. J. Ab Initio Nonadiabatic Molecular Dynamics with Hole–Hole Tamm–Dancoff Approximated Density Functional Theory. *J. Chem. Theory Comput.* **2020**, *16*, 5499–5511.

[60] Grimme, S.; Waletzke, M. A Combination of Kohn–Sham Density Functional Theory and Multi-Reference Configuration Interaction Methods. *J. Chem. Phys.* **1999**, *111*, 5645–5655.

[61] Marian, C. M.; Heil, A.; Kleinschmidt, M. The DFT/MRCI Method. *WIREs Comput. Mol. Sci.* **2019**, *9*, e1394.

[62] Neville, S. P.; Schuurman, M. S. Removing the Deadwood from DFT/MRCI Wave Functions: The p-DFT/MRCI Method. *J. Chem. Theory Comput.* **2021**, *17*, 7657–7665.

[63] GRaCI: General Reference Configuration Interaction. (https://github.com/schuurman-group/graci).

[64] Becke, A. D. A New Mixing of Hartree–Fock and Local Density-functional Theories. *J. Chem. Phys.* **1993**, *98*, 1372–1377.

[65] Heil, A.; Kleinschmidt, M.; Marian, C. M. On the Performance of DFT/MRCI Hamiltonians for Electronic Excitations in Transition Metal Complexes: The Role of the Damping Function. *J. Chem. Phys.* **2018**, *149*, 164106.

[66] Sun, Q.; Berkelbach, T. C.; Blunt, N. S.; Booth, G. H.; Guo, S.; Li, Z.; Liu, J.; McClain, J. D.; Sayfutyarova, E. R.; Sharma, S.; Wouters, S.; Chan, G. K.-L. PySCF: The

Python-based Simulations of Chemistry Framework. *WIREs Comput. Mol. Sci.* **2018**, *8*, e1340.

[67] Lehtola, S.; Steigemann, C.; Oliveira, M. J. T.; Marques, M. A. L. Recent Developments in Libxc — A Comprehensive Library of Functionals for Density Functional Theory. *SoftwareX* **2018**, *7*, 1–5.

[68] Zhao, Y.; Truhlar, D. G. Design of Density Functionals That Are Broadly Accurate for Thermochemistry, Thermochemical Kinetics, and Nonbonded Interactions. *J. Phys. Chem. A* **2005**, *109*, 5656–5667.

[69] Caldeweyher, E.; Ehlert, S.; Hansen, A.; Neugebauer, H.; Spicher, S.; Bannwarth, C.; Grimme, S. A Generally Applicable Atomic-Charge Dependent London Dispersion Correction. *J. Chem. Phys.* **2019**, *150*, 154122.

[70] Neese, F. An Improvement of the Resolution of the Identity Approximation for the Formation of the Coulomb Matrix. *J. Comput. Chem.* **2003**, *24*, 1740–1747.

[71] Vahtras, O.; Almlöf, J.; Feyereisen, M. W. Integral Approximations for LCAO-SCF Calculations. *Chem. Phys. Lett.* **1993**, *213*, 514–518.

[72] Weigend, F. Accurate Coulomb-fitting Basis Sets for H to Rn. *Phys. Chem. Chem. Phys.* **2006**, *8*, 1057–1065.

[73] Neese, F.; Wennmohs, F.; Hansen, A.; Becker, U. Efficient, Approximate and Parallel Hartree–Fock and Hybrid DFT Calculations. A 'Chain-of-Spheres' Algorithm for the Hartree–Fock Exchange. *Chem. Phys.* **2009**, *356*, 98–109.

[74] Helmich-Paris, B.; de Souza, B.; Neese, F.; Izsák, R. An Improved Chain of Spheres for Exchange Algorithm. *J. Chem. Phys.* **2021**, *155*, 104109.

[75] Grimme, S. Supramolecular Binding Thermodynamics by Dispersion-Corrected Density Functional Theory. *Chem. Eur. J.* **2012**, *18*, 9955–9964.

[76] Spicher, S.; Grimme, S. Efficient Computation of Free Energy Contributions for Association Reactions of Large Molecules. *J. Phys. Chem. Lett.* **2020**, *11*, 6606–6611.
